# CXCL3 promotes liver cancer progression by modulating the tumor microenvironment via the PI3K/AKT/mTOR pathway

**DOI:** 10.1371/journal.pone.0334639

**Published:** 2025-11-19

**Authors:** Yue Li, Tao Liu, Ziteng Cai, Chaonan Peng, Zhilei He, Lichun Liang, Bo Han, Ran Bi, Lei Liu, Weiqun Wang

**Affiliations:** 1 Basic Medical College, Jiamusi University, Jiamusi, Heilongjiang, China; 2 Key laboratory of Microecology-immune Regulatory Network and Related Diseases School of Basic Medicine, Jiamusi University, Jiamusi, Heilongjiang Province, China; 3 Department of Surgery, First College of Medicine, Jiamusi University, Jiamusi, Heilongjiang, China; 4 School of Public Health, Jiamusi University, Jiamusi, Heilongjiang, China; 5 State Key Laboratory of Neurology and Oncology Drug Development, Nanjing, Jiangsu, China; University of Toronto, CANADA

## Abstract

CXCL3, a member of the CXC chemokine family, has been increasingly implicated in the progression of various cancers, including hepatocellular carcinoma, due to its role in immune and inflammatory responses within the tumor microenvironment. This study aimed to investigate the expression and function of CXCL3 in liver cancer and to elucidate its underlying mechanisms. A combination of bioinformatics analysis, ELISA, RT-qPCR, immunohistochemistry, in vitro cell assays, and in vivo nude mouse models was employed to assess CXCL3 expression and function. The results showed that CXCL3 was significantly upregulated in hepatocellular carcinoma tissues and associated with reduced overall survival in patients. It promoted the proliferation, colony formation, and migration of liver cancer cells (Bel-7402, HepG2, and SMMC-7721) via exogenous, autocrine, and paracrine mechanisms, and recruited tumor-associated macrophages, neutrophils, and fibroblasts into the tumor microenvironment. Mechanistically, CXCL3 activated the PI3K/AKT/mTOR pathway by upregulating PI3K, p-PI3K, AKT, p-AKT, mTOR, and p-mTOR, while the mTOR inhibitor Torin 1 reversed these effects. Gene set enrichment analysis showed enrichment in immune-related pathways, including Toll-like receptor and chemokine signaling. In vivo, CXCL3 overexpression significantly promoted tumor growth in nude mice. These findings suggest CXCL3 facilitates liver cancer progression through tumor microenvironment modulation and PI3K/AKT/mTOR pathway activation.

## 1 Introduction

Liver cancer is one of the most common malignant tumors worldwide. It ranks sixth in global cancer incidence and third in cancer-related mortality [1]. The burden of liver cancer varies across geographic regions and ethnic populations, with nearly 50% of new cases diagnosed in China alone [2]. Hepatocellular carcinoma (HCC), the predominant subtype, accounts for approximately 80% of all primary liver cancers [3]. Current treatment strategies—such as surgical resection, radiofrequency ablation, interventional embolization, and liver transplantation—can yield favourable outcomes, particularly in early-stage patients [4,5]. However, challenges remain in managing advanced disease, highlighting the importance of uncovering molecular mechanisms underlying tumor progression and identifying novel prognostic and therapeutic biomarkers.

Chemokines are a group of small, secreted proteins in the cytokine superfamily, with molecular weights ranging from 8 to 12 kDa. The chemokine family includes approximately 50 ligands, 20 classical G protein-coupled receptors, and 4 atypical chemokine receptors [6]. These ligands and receptors often exhibit overlapping interactions: 14 of the 19 typical receptors can bind multiple ligands, and vice versa [7]. This complex chemokine network enables precise cellular responses to microenvironmental signals and plays a central role in regulating immune cell activation, chemotaxis, and migration during inflammation and immune surveillance [8]. Increasing evidence suggests that chemokines and their receptors are frequently mutated or aberrantly expressed in tumors and are functionally linked to cancer progression. Their involvement spans immune evasion, cell proliferation, angiogenesis, maintenance of stemness, and metastatic behaviour [9]. By directing immune cell recruitment into the tumor microenvironment, chemokines help shape local immune responses and influence intercellular interactions that govern tumor dynamics [8,10].

CXCL3 is a member of the CXC chemokine subfamily and is classified as an ELR+ chemokine (Glu-Leu-Arg motif) [11]. Recent studies indicate that CXCL3 is overexpressed in multiple malignancies and may contribute to tumor initiation and progression by acting within the tumor microenvironment [12]. Its biological effects are primarily mediated via the CXCR2 receptor [12], which also binds other ligands such as CXCL1, CXCL5, and CXCL8 [7]. Our previous work demonstrated that CXCL5 enhances liver cancer cell migration and proliferation through both autocrine and paracrine mechanisms. Notably, CXCL5 derived from stromal cells exerts a similarly strong pro-tumorigenic effect [13]. Since both CXCL3 and CXCL5 signal through CXCR2, it is plausible that CXCL3 may also promote liver cancer progression via CXCR2-dependent pathways.

## 2 Materials and Methods

### 2.1 Bioinformatics analysis

RNA sequencing data for CXCL3 and corresponding clinical information were obtained from The Cancer Genome Atlas (TCGA) database (https://portal.gdc.cancer.gov/). The mRNA expression levels of CXCL3 in normal and hepatocellular carcinoma (HCC) tissues were compared. The association between CXCL3 expression and patient survival was assessed using Kaplan–Meier survival analysis. The correlation between CXCL3 expression and immune cell infiltration was analyzed using the TIMER database (https://cistrome.shinyapps.io/timer/). Kyoto Encyclopedia of Genes and Genomes (KEGG) pathway enrichment was conducted using Gene Set Enrichment Analysis (GSEA), and the pathways with the highest enrichment scores were selected for further investigation.

### 2.2 Clinical specimens and immunohistochemistry

From 1st February 2023 to 1st April 2024, samples were collected at Jiamusi Medical College. On 27th May 2024, the organization microarray processing was entrusted to Zhongke Guanghua (Xi’an) Intelligent Biotechnology Co., Ltd. for scientific research purposes. The constructed tissue microarray contains 96 tissue samples, comprising 48 liver cancer tissues and 48 normal liver tissues. The liver cancer tissues were categorized according to tumor infiltration stage: 6 cases were classified as T1, 9 as T2, and 33 as T3. In terms of sex distribution, there were 11 female and 37 male cases. Age-based stratification included 24 patients aged ≤55 years and 24 aged >55 years.

The tissue microarray was processed through a standard immunohistochemistry (IHC) protocol. Briefly, sections were deparaffinized using xylene, treated with methanol-H₂O₂ to eliminate endogenous peroxidase activity, and passed through graded alcohols. Antigen retrieval was performed by incubating the slides in 10 mM citrate buffer (pH 6.0). Following PBS washes, the slides were blocked with BSA solution and incubated overnight at 4°C with a primary polyclonal antibody against CXCL3 (cat. no. YT2075; dilution 1:100; ImmunoWay). The following day, the slides were washed with PBS, incubated with IgG-biotin and SABC complex (Beyotime Institute of Biotechnology), and washed again. 3,3′-Diaminobenzidine (DAB) was used for color development. The stained slides were examined under a microscope, and images were captured. ImageJ 1.52i software was used to analyse the average optical density of the staining images [14].

### 2.3 Cell lines, culture and transfection

The human liver cancer cell lines HepG2, Bel-7402, and SMMC-7721 were obtained from the American Type Culture Collection and cultured in RPMI-1640 medium (Corning, Inc.) supplemented with 10% fetal bovine serum (FBS; NQBB, Australia) and penicillin/streptomycin. Cells were maintained in a humidified incubator at 37°C with 5% CO₂. Lentiviral vectors were constructed, including an empty control vector, a CXCL3 overexpression vector, and a vector carrying a CXCL3-targeting interfering sequence (sh-CXCL3). Pseudovirus particles containing these constructs were produced by GeneCopoeia Inc. (Rockville, MD, USA). To establish CXCL3-overexpressing, CXCL3-silenced (sh-CXCL3), and corresponding mock control cell lines, the liver cancer cells were infected with the respective pseudoviruses following the manufacturer’s instructions [13]. HepG2, Bel-7402, and SMMC-7721 cell lines were identified through short tandem repeat (STR) analysis. The STR results showed that the DNA of the cell line matched perfectly with HepG2, Bel-7402, and SMMC-7721, and no cross contamination of human cells was detected (S1 Appendix in S1 File. STR analysis.).

### 2.4 Reverse transcription-quantitative PCR (RT-qPCR)

CXCL3-overexpressing cells, sh-CXCL3 cells, CXCL3-overexpressing LX-2 cells, and their respective mock control cells were seeded in 6-well plates at a density of 3 × 10⁶ cells per well in 1 mL RPMI-1640 medium supplemented with 10% FBS. After 24 hours of incubation, cells were trypsinized, and the supernatant was discarded. The resulting cell pellet was transferred to a 1.5 mL centrifuge tube. Total RNA was extracted from hepatocellular carcinoma and liver stromal cells using TRIzol® reagent (Sangon Biotech, Shanghai, China) according to the manufacturer’s instructions. Reverse transcription of 2 µg of total RNA into cDNA was performed using a cDNA synthesis kit (Sangon Biotech, Shanghai, China). The primer sequences of CXCL3 and β - actin are listed in Table 1. Quantitative PCR was subsequently carried out using the SuperReal PreMix Plus kit [15].

**Table 1:** Primers sequences used for qRT-PCR.

Gene	Primers	Sequence (5’ to 3’)
CXCL3	F1	tga atg taa ggt ccc ccg ga
	R1	cac cct gca gga agt gtc aa
β-actin	F1	cct tcc ttc ctg ggc atg g
	R1	tct tca ttg tgc tgg gtg cc

### 2.5 Enzyme-linked immunosorbent assay (ELISA)

Cells were seeded in 6-well plates at a density of 3 × 10⁶ cells per well in 1 mL RPMI-1640 medium supplemented with 10% FBS. After 24 hours of culture, the culture medium from each well was collected and centrifuged to isolate the supernatant. The supernatant was then added to an ELISA plate following the manufacturer’s instructions for the Human CXCL3 ELISA Kit (Mskbio). A microplate reader was used to measure optical density at a wavelength of 450 nm. Experimental results were zeroed against the blank well, and CXCL3 concentrations were quantified using a standard curve. The amount of CXCL3 secreted by the cells in each sample was calculated accordingly [14].

### 2.6 Cell Counting Kit-8 (CCK-8) assay

a) For the exogenous stimulation study, liver cancer cells were seeded in 96-well plates at a density of 3 × 10³ cells per well in 100 µL of medium containing various concentrations of recombinant CXCL3 (0, 2, 5, 10, 20, or 30 ng/mL), with or without 0.775 ng/mL of the mTOR inhibitor Torin 1 (APeXBIO Technology LLC). b) For the autocrine study, stably transfected liver cancer cells were seeded in 96-well plates (3 × 10³ cells/well) in 100 µL of medium, with or without 0.775 ng/mL Torin 1. c) For the paracrine study, liver cancer cells were cultured in 96-well plates (3 × 10³ cells/well) in 100 µL of conditioned medium collected from LX-2 cells. After 48 hours of incubation, cell proliferation was assessed using the Cell Counting Kit-8 (CCK-8) assay [16]. The inhibitory effect of Torin 1 on cell proliferation was calculated using the following formula:

Inhibition rate of proliferation (%) = [(OD value in the DMSO-treated group − OD value in the Torin 1-treated group)/ OD value in the DMSO-treated group] × 100%.

### 2.7 5-Ethynyl-2´-deoxyuridine (EdU) cell proliferation assay

This assay was conducted for both exogenous and autocrine studies as follows:

a) In the exogenous study, liver cancer cells were seeded in 24-well plates at a density of 5 × 10⁴ cells per well in complete RPMI-1640 medium supplemented with 10% FBS and 100 U/mL penicillin/streptomycin. After cell adherence, the culture medium was replaced with fresh medium containing 10% FBS and varying concentrations of recombinant CXCL3 (0, 5, or 10 ng/mL).
b) In the autocrine study, log-phase stably transfected hepatocellular carcinoma cells (transfected with empty vector, CXCL3, scrambled shRNA, or CXCL3-shRNA) were seeded in 24-well plates at a density of 5 × 10⁴ cells per well in 500 µL RPMI-1640 medium containing 10% FBS.

Cell proliferation was assessed using the Click-iT™ EdU Cell Proliferation Kit (cat. no. C0075S; Beyotime Institute of Biotechnology), following the manufacturer’s instructions. After nuclear staining with Hoechst 33342, EdU-positive cells were visualized using a fluorescence microscope. The proliferation rate was calculated as follows:

EdU-positive rate (%) = (Number of cells with red fluorescence/ Total number of cells with blue fluorescence) × 100% [17].

### 2.8 Cell cloning assay

Liver cancer cells were seeded at a density of 3 × 10² cells per well in 24-well plates containing 500 µL of complete RPMI-1640 medium. The culture medium was refreshed every 3 days. After 12 days of incubation at 37°C in a humidified incubator, cells were fixed with fixative solution and stained with crystal violet at room temperature for 30 minutes. Colonies were then imaged and counted under a microscope [18].

### 2.9 Transwell migration assay

Cell migration was assessed using 24-well Transwell chambers (Biofil, China) with 8.0 µm pore membranes.

1). Exogenous study: Liver cancer cells were seeded into the upper chamber at a density of 2 × 10⁴ cells per well in serum-free medium. The lower chamber was filled with 600 µL of medium containing 15% FBS and various concentrations of recombinant CXCL3 (0, 2, 5, 10, 20, or 30 ng/mL), with or without 0.775 ng/mL Torin 1.
2). Autocrine study: Stably transfected liver cancer cells were seeded in the upper chamber (2 × 10⁴ cells/chamber) in serum-free medium, while the lower chamber contained 600 µL of medium with 15% FBS, with or without Torin 1.
3). Paracrine study: Liver cancer cells were seeded in the upper chamber (2 × 10⁴ cells/chamber) in serum-free medium, and the lower chamber was filled with 600 µL of conditioned medium from LX-2 cells at varying concentrations (0%, 20%, 40%, 60%, or 80%).

After 48 hours of incubation at 37°C, non-migratory cells on the upper surface of the membrane were removed with a sterile cotton swab. The migrated cells on the lower surface were fixed and stained with crystal violet/ethanol solution for 30 minutes at room temperature. Cells were imaged and counted under a bright-field microscope [19]. The inhibition rate of migration by Torin 1 was calculated using the formula:

Inhibition rate of migration (%) = [(Number of migratory cells in the DMSO-treated group − Number in the Torin 1-treated group)/ Number in the DMSO-treated group] × 100%.

### 2.10 Cell scratch assay

CXCL3-overexpressing cells, sh-CXCL3 cells, and their respective mock control cells were seeded into 6-well plates at a density of 4 × 10⁵ cells per well in 1 mL of complete medium supplemented with 10% FBS. Once the cells reached full confluence, a sterile 10 µL pipette tip was used to create a scratch wound across the cell monolayer. Detached cells were gently removed by rinsing with serum-free medium.

Images of the scratch area were captured immediately after wounding (0 h). Cells were then incubated at 37°C in medium containing FBS. After 24 and 48 hours, additional images were taken under a fluorescence microscope to record the migration of cells into the scratch area. The scratch closure (migration distance) was measured using ImageJ 1.52i software [20].

### 2.11 Xenotransplantation of tumor cells in nude mice

This study was conducted in accordance with the guidelines of the Declaration of Helsinki and was approved by the Medical Ethics Committee at Jiamusi University (approval no. JDJCYXY 20240007). Log-phase HepG2 mock control cells and CXCL3-overexpressing HepG2 cells were harvested and resuspended in 100 µL of serum- and antibiotic-free RPMI-1640 medium at a concentration of 3 × 10⁶ cells per injection. Each suspension was subcutaneously injected into the dorsal region of immunodeficient (nude) mice. Nude mice were divided into overexpression group and mock group, with 8 mice in each group. The mice were monitored for tumor growth over a period of 45 days. At the endpoint, all mice were humanely euthanized. The xenograft tumors were carefully excised, weighed, photographed, and stored at −80°C for subsequent analysis [21].

### 2.12 Western blot assay

Western blot analysis was performed following standard procedures. Equal amounts of total protein (30 µg) were separated on 12% SDS-PAGE gels and transferred to 0.45-µm polyvinylidene difluoride (PVDF) membranes (MilliporeSigma). After blocking with 5% skimmed milk, membranes were incubated overnight at 4°C with primary antibodies, including mTOR (cat. no. YT2913; 1:1,000; ImmunoWay), p-mTOR (cat. no. YP0176; 1:1,000; ImmunoWay), AKT (cat. no. CY5551; 1:1,000; Shanghai Abways), p-AKT (cat. no. CY6569; 1:1,000; Shanghai Abways), PI3K (cat. no. CY5355; 1:1,000; Shanghai Abways), and p-PI3K (cat. no. AF3241; 1:1,000; Affinity). After washing with PBST (PBS with Tween-20, 1:2,000), membranes were incubated with HRP-conjugated goat anti-rabbit (cat. no. TA373083; 1:10,000; OriGene) or goat anti-mouse secondary antibodies (cat. no. TA373082; 1:10,000; OriGene). Bands were visualized using an enhanced chemiluminescence (ECL) kit (Thermo Fisher Scientific, Inc.) and detected according to the manufacturer’s protocol [22].

### 2.13 Statistical analysis

Statistical analysis was conducted with SPSS 29.0 software (IBM Corp.). The data are presented as the mean ± SD. Comparisons between two groups were performed using a two-tailed unpaired Student’s t-test, comparisons among multiple groups were performed by one-way ANOVA, followed by a post-hoc test using Tukey’s Honestly Significant Difference Test. P<0.05 was considered to indicate a statistically significant difference.

## 3 Results

### 3.1 Expression profile and clinical relevance of CXCL3 in liver cancer

**Fig 1:**
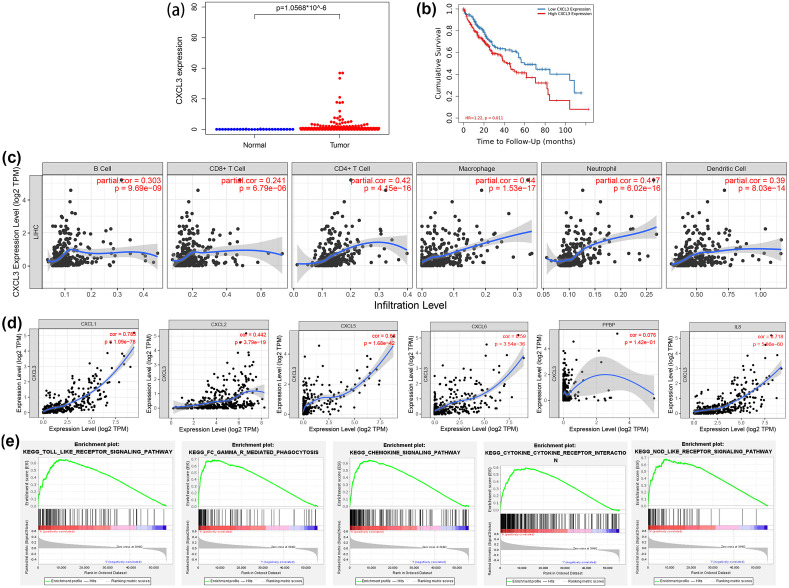
Comprehensive bioinformatics analyses of CXCL3 expression patterns and functional roles in liver cancer. **(a)** CXCL3 RNA expression obtained from the TCGA database. **(b)** Kaplan-Meier survival analysis of liver cancer patients. **(c)** The association between CXCL3 expression and infiltration of immune cells. **(d)** The expression correlation between CXCL3 and CXCR2-associated ligands. **(e)** Enrichment analysis of CXCL3 expression.

RNA sequencing data revealed that CXCL3 expression was significantly upregulated in liver cancer tissues compared to normal liver tissues (Fig 1a). Kaplan–Meier survival analysis indicated that patients with high CXCL3 expression had markedly shorter overall survival, suggesting its prognostic value (Fig 1b). Analysis using the TIMER database demonstrated a positive correlation between CXCL3 expression and immune cell infiltration, including macrophages, neutrophils, B cells, CD4⁺ T cells, CD8⁺ T cells, and dendritic cells within the tumor microenvironment (Fig 1c).

Given that CXCR2 ligands include CXCL1, CXCL2, CXCL3, CXCL5, CXCL6, CXCL7 [pro-platelet basic protein (PPBP)], and CXCL8 (IL-8), the expression correlation between CXCL3 and other CXCR2 ligands was assessed. CXCL3 expression was positively correlated with several ligands, particularly CXCL1, CXCL5, and CXCL8, but not with CXCL7 (Fig 1d). Furthermore, gene set enrichment analysis (GSEA) revealed that high CXCL3 expression was significantly associated with several immune and inflammation-related pathways, including Toll-like receptor signaling, FcγR-mediated phagocytosis, chemokine signaling, cytokine–cytokine receptor interaction, and NOD-like receptor signaling pathways (Fig 1e). Collectively, these findings suggest that elevated CXCL3 expression in liver cancer is linked to poor prognosis, immune cell recruitment, CXCR2 ligand co-expression, and activation of tumor-related immune pathways.

### 3.2 CXCL3 expression in liver cancer

**Fig 2:**
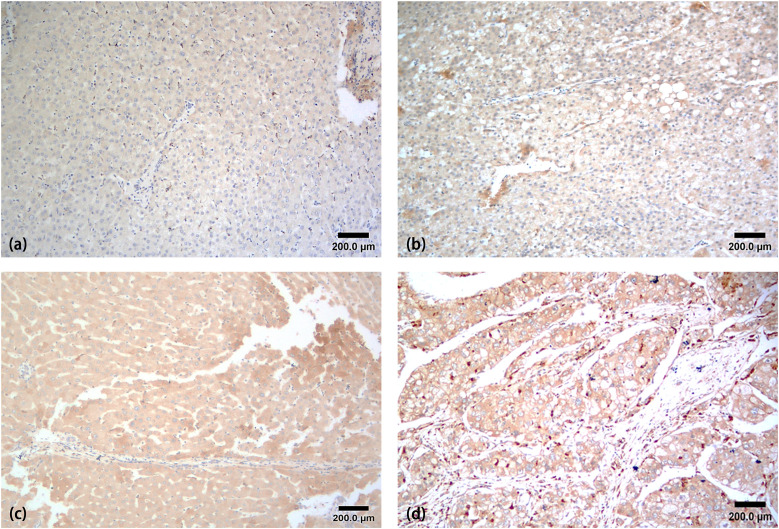
Immunohistochemical assessment of CXCL3 expression in a tissue microarray. **(a)** Normal liver tissues with weak CXCL3 staining. **(b-d)** Liver cancer tissues (Grades I-III) with strong CXCL3 staining.

Immunohistochemistry results demonstrated notably higher CXCL3 expression in liver cancer tissues compared to that in normal liver tissues (P < 0.01). Furthermore, CXCL3 expression positively correlated with TNM staging (P < 0.01), which unrelated to gender or age. (Table 2 and Fig 2).

**Table 2:** Expression of CXCL3 in liver tissue microarray ( –x ± s).

		Mean optical density	*P*
**Cancer tissue**		0.150 ± 0.020	<0.01^*^
**Paracancer tissue**		0.130 ± 0.024	
**Clinical stages**	I	0.125 ± 0.005	<0.01^&^
	II	0.147 ± 0.014	
	III	0.166 ± 0.020	
**Gender**	Man	0.145 ± 0.023	0.961^#^
	Woman	0.145 ± 0.020	
**Age**	≤55	0.136 ± 0.027	0.114^△^
	>55	0.144 ± 0.020	

optical density of cytoplasm; * vs. Paracancer tissue; ^&^ vs. I; ^#^ vs. Man; ^△^ vs. ≤55.

### 3.3 Exogenous CXCL3 promotes malignant behavior of liver cancer cells

**Fig 3:**
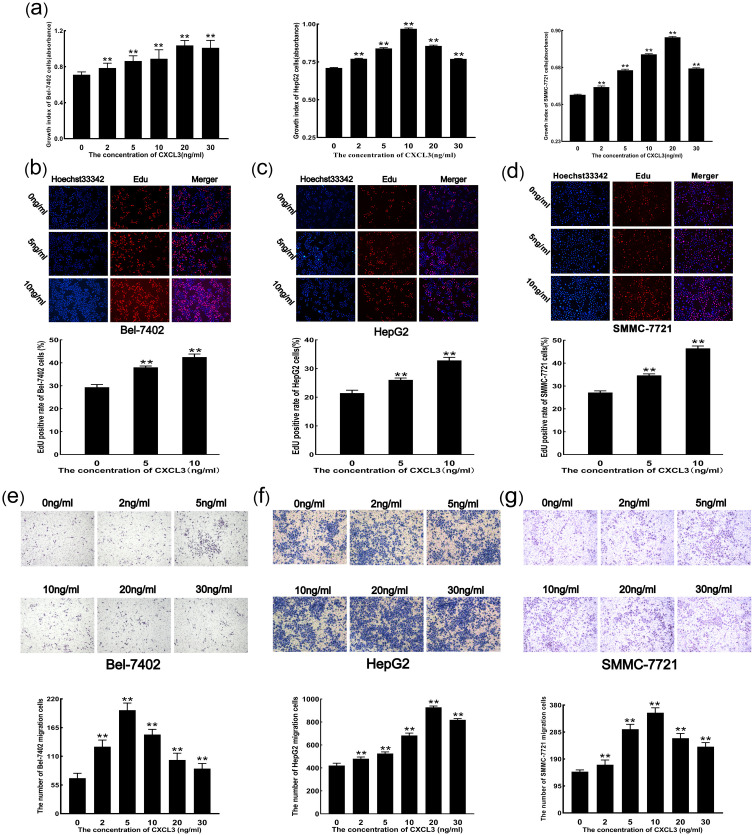
Effects of exogenous CXCL3 on liver cancer cell malignancy. **(a)** Cell proliferation measured by CCK-8 assay. **(b-d)** Cell viability analyzed by EdU staining. **(e-g)** Cell migration assessed by Transwell assay. **p < 0.01 vs. 0 ng/ml CXCL3.

The CCK-8 assay demonstrated that treatment with exogenous CXCL3 at concentrations of 2, 5, 10, 20, and 30 ng/mL significantly promoted the proliferation of Bel-7402, HepG2, and SMMC-7721 cells (Fig 3a). Consistently, the EdU assay showed increased cell proliferation in response to 5 and 10 ng/mL CXCL3, confirming its stimulatory effect on cell viability (Fig 3b–3d). Moreover, Transwell migration assays revealed that exogenous CXCL3 at the same concentration range (2–30 ng/mL) enhanced the migratory capacity of all three liver cancer cell lines (Fig 3e–3g).

### 3.4 CXCL3 overexpression promotes malignant behavior of liver cancer cells

**Fig 4:**
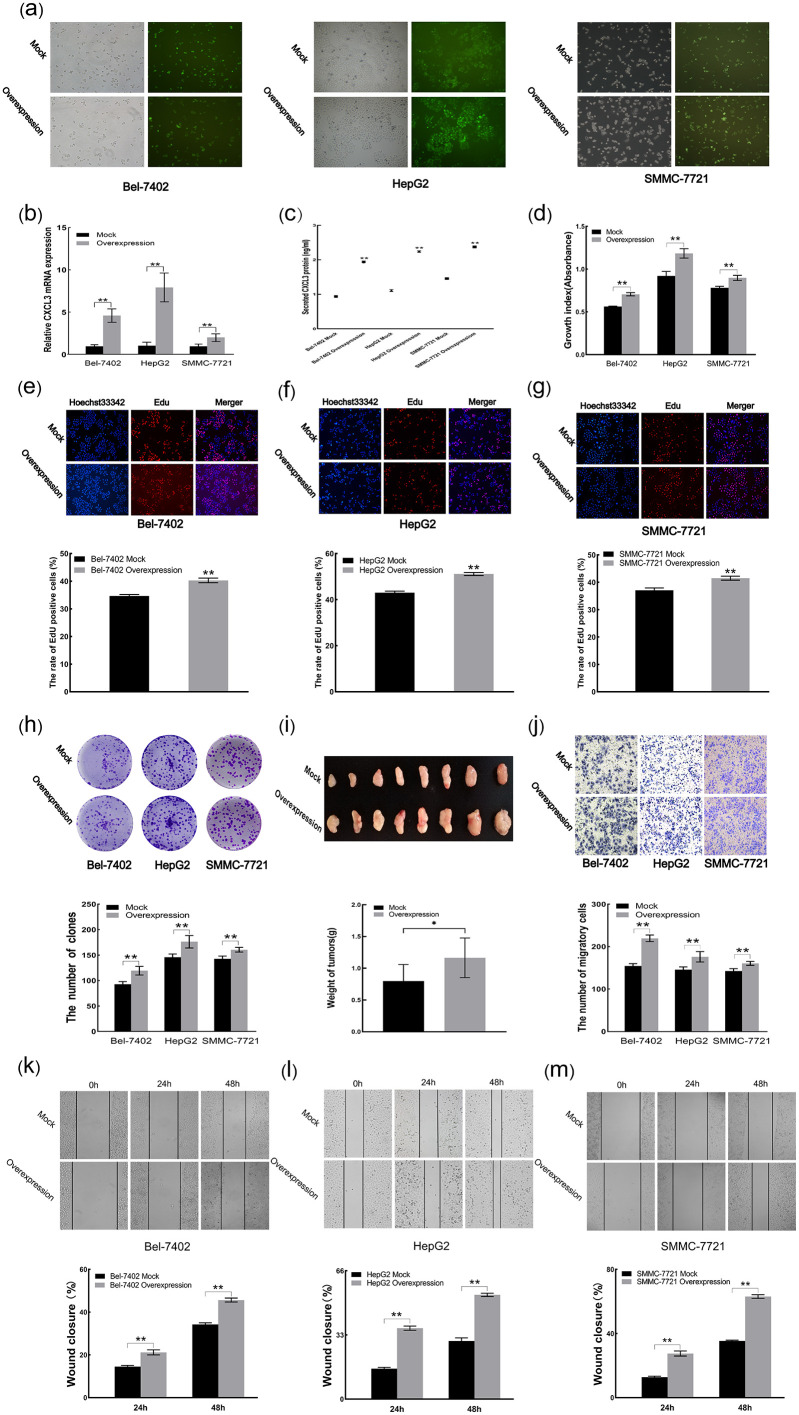
Effects of CXCL3 overexpression on liver cancer cell malignancy in vitro and in vivo. **(a)** Transfection efficiency visualized by fluorescence. **(b)** CXCL3 mRNA levels quantified by RT-qPCR. **(c)** Secreted CXCL3 protein measured by ELISA. **(d)** Cell proliferation assessed by CCK-8 assay. **(e-g)** Cell viability analyzed by EdU staining. **(h)** Cell colony formation evaluated by clonogenic assay **(i)** Tumors in the overexpression group compared to the mock group in nude mice. **(j-l)** Cell migration determined by scratch analysis. *p < 0.05, **p < 0.01.

Following transfection of Bel-7402, HepG2, and SMMC-7721 cells, fluorescence microscopy confirmed a transfection efficiency exceeding 80% (Fig 4a). RT-qPCR and ELISA analyses further verified successful CXCL3 overexpression in these cell lines (Fig 4b and 4c). Functional assays, including CCK-8, EdU, and colony formation assays, revealed that CXCL3 overexpression significantly promoted cell proliferation, viability, and clonogenic capacity (Fig 4d–4h). In vivo, mice injected with CXCL3-overexpressing HepG2 cells developed tumors with notably larger volumes compared to controls (Fig 4i). Moreover, both Transwell and wound healing (scratch) assays demonstrated that CXCL3 overexpression enhanced the migratory ability of the liver cancer cells (Fig 4j–4m).

### 3.5 Knockdown of CXCL3 suppresses malignant behavior of liver cancer cells

**Fig 5:**
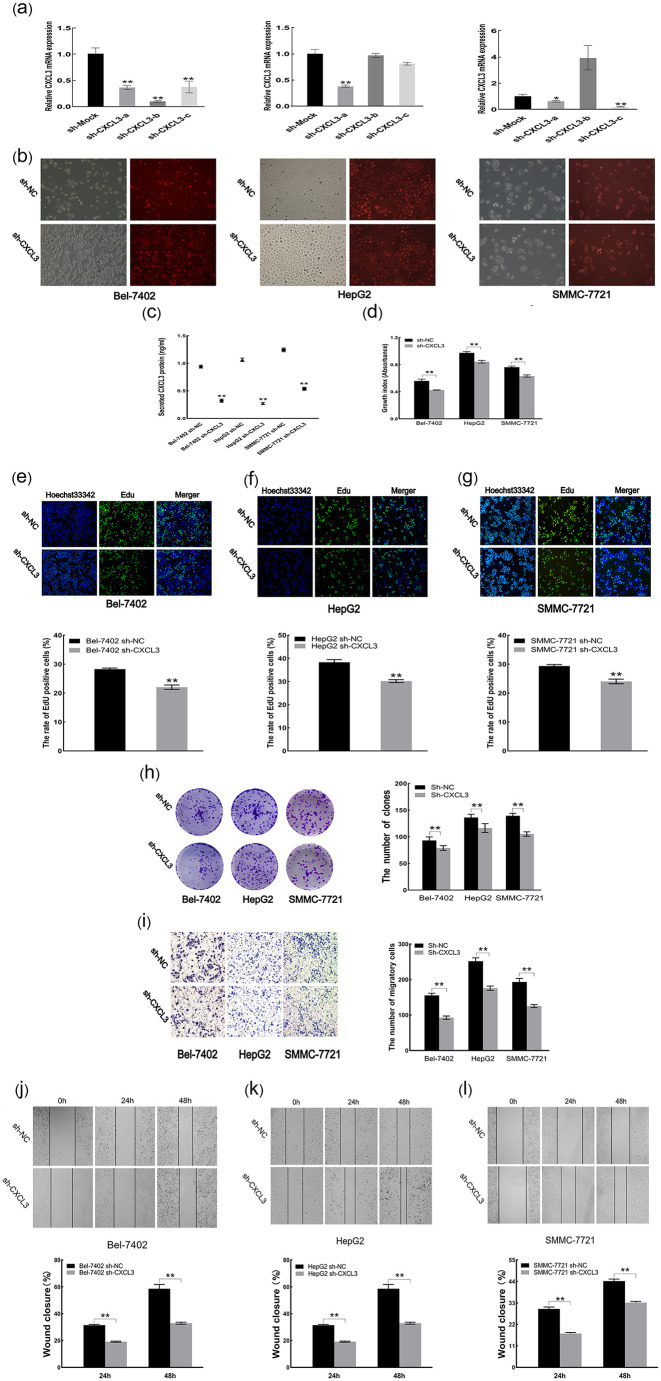
Effects of CXCL3 low expression on liver cancer cell malignancy. **(a)** CXCL3 mRNA levels quantified by RT-qPCR. **(b)** Transfection efficiency visualized by fluorescence. **(c)** Secreted CXCL3 protein measured by ELISA. **(d)** Cell proliferation assessed by CCK-8 assay. **(e-g)** Cell viability analyzed by EdU staining. **(h)** Cell colony formation evaluated by clonogenic assay. **(i)** Cell migration estimated by Transwell assay. **(j-l)** Cell migration determined by scratch analysis. **p < 0.01.

To achieve effective knockdown of CXCL3, three distinct shRNA sequences targeting CXCL3 were designed. RT-qPCR analysis confirmed that at least one of the sequences significantly reduced CXCL3 expression in Bel-7402, HepG2, and SMMC-7721 cells (Fig 5a). Fluorescence microscopy showed a transfection efficiency exceeding 80% (Fig 5b). ELISA results further demonstrated a reduction in secreted CXCL3 levels in the culture supernatants following knockdown (Fig 5c). Functional assays, including CCK-8, EdU, and colony formation, revealed that CXCL3 downregulation significantly suppressed cell proliferation, viability, and clonogenic capacity (Fig 5d–5h). Moreover, migration assays showed that silencing of CXCL3 led to a marked decrease in the migratory ability of the liver cancer cells (Fig 5i–5l).

### 3.6 CXCL3 promotes malignant behaviors of liver cancer cells via the paracrine pathway

**Fig 6:**
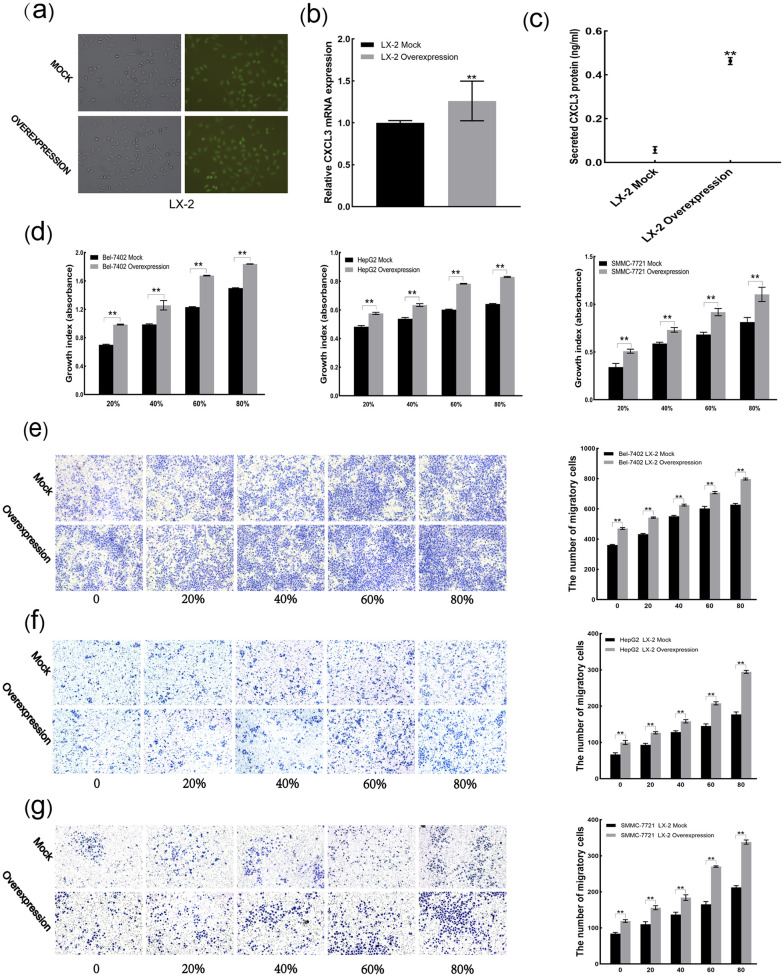
Effects of paracrine CXCL3 on liver cancer cell malignancy. **(a)** Transfection efficiency visualized by fluorescence. **(b)** CXCL3 mRNA levels quantified by RT-qPCR. **(c)** Secreted CXCL3 protein measured by ELISA. **(d)** Cell proliferation assessed by CCK-8 assay. **(e-g)** Cell migration estimated by Transwell assay. **p < 0.01.

Upon transfection of LX-2 cells, a transfection efficiency of over 80% was observed under fluorescence microscopy (Fig 6a). RT-qPCR and ELISA assays demonstrated a significant increase in CXCL3 expression in LX-2 cells overexpressing CXCL3 (Fig 6b and 6c). CCK-8 assay demonstrated that 20%, 40%, 60% and 80% conditioned medium from CXCL3-overexpressing LX-2 cells enhanced proliferation of Bel-7402, HepG2 and SMMC-7721 cells (Fig 6d). Transwell assay also showed that 20, 40, 60 and 80% conditioned medium from CXCL3-overexpressing LX-2 cells promoted migration of these cells (Fig 6e-6g).

### 3.7 Effect of exogenous CXCL3 on the mTOR pathway

**Fig 7:**
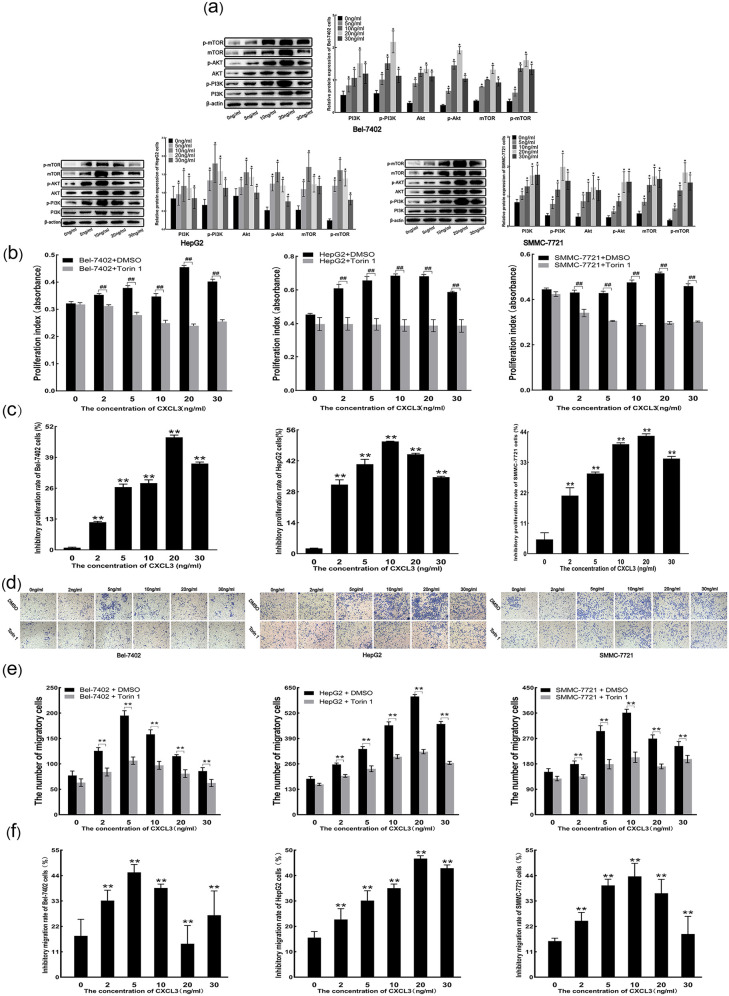
Exogenous CXCL3 promotes liver cancer cell malignancy via the mTOR pathway. **(a)** PI3K, p-PI3K, AKT, p-AKT, mTOR and p-mTOR protein levels quantified by Western blot analysis. **(b)** Cell proliferation assessed by CCK-8 assay. **(c)** Cell proliferative inhibition rate evaluated by CCK-8 assay. **(d)** Representative images of Transwell analysis **(e)** Cell migration estimated by Transwell assay. **(f)** Cell migratory inhibition rate determined Transwell assay. # #p < 0.01, *p < 0.05 vs. 0 ng/ml CXCL3, **p < 0.01 vs. 0 ng/ml CXCL3.

Western blot analysis demonstrated that treatment with exogenous CXCL3 at concentrations of 5, 10, 20, and 30 ng/mL significantly increased the protein expression levels of PI3K, p-PI3K, AKT, p-AKT, mTOR, and p-mTOR in Bel-7402, HepG2, and SMMC-7721 cells compared to cells treated with 0 ng/mL CXCL3 (Fig 7a). CCK-8 assays showed that CXCL3 at these concentrations enhanced cell proliferation, even following treatment with the mTOR inhibitor Torin 1 (Fig 7b). Notably, the proliferation inhibition rates [14] induced by Torin 1 were greater in the CXCL3-treated groups than in the 0 ng/mL CXCL3 control group (Fig 7c). Similarly, Transwell assays indicated that CXCL3 continued to promote cell migration after Torin 1 treatment (Fig 7d and 7e), although the migratory inhibition rates [14] were also significantly increased relative to the untreated control (Fig 7f). These findings suggest that exogenous CXCL3 promotes the malignant behavior of liver cancer cells through activation of the mTOR signaling pathway.

### 3.8 CXCL3 overexpression promotes malignant behavior via the mTOR pathway

**Fig 8:**
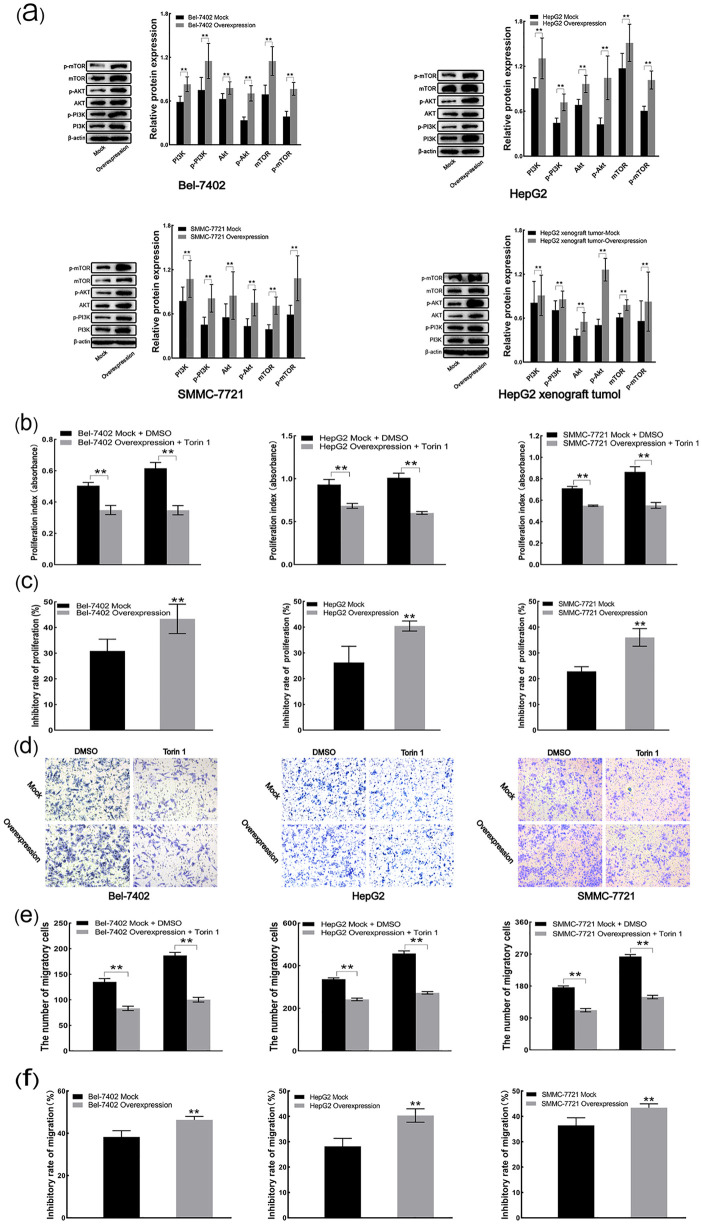
CXCL3 overexpression facilitates liver cancer cell malignancy via the mTOR pathway. **(a)** PI3K, p-PI3K, AKT, p-AKT, mTOR and p-mTOR protein levels quantified by Western blot analysis. **(b)** Cell proliferation assessed by CCK-8 assay. **(c)** Cell proliferative inhibition rate evaluated by CCK-8 assay. **(d)** Representative images of Transwell analysis **(e)** Cell migration estimated by Transwell assay. **(f)** Cell migratory inhibition rate determined Transwell assay. **p < 0.01.

In both *in vitro* cell experiments and *in vivo* xenograft tumor models, CXCL3 overexpression led to elevated protein levels of PI3K, p-PI3K, AKT, p-AKT, mTOR, and p-mTOR compared to mock controls (Fig 8a). CCK-8 assays showed that, even after treatment with the mTOR inhibitor Torin 1, proliferation remained higher in CXCL3-overexpressing cells than in mock cells (Fig 8b). However, the proliferation inhibition rate induced by Torin 1 was greater in CXCL3-overexpressing cells compared to mock controls (Fig 8c). Similarly, although migration ability remained elevated in CXCL3-overexpressing cells following Torin 1 treatment, the migratory inhibition rate was also higher in these cells than in mock cells (Fig 8d–8f). These findings suggest that CXCL3 overexpression promotes the malignant behaviour of liver cancer cells by activating the mTOR

### 3.9 Low expression of CXCL3 suppresses the malignant behavior via the mTOR pathway

**Fig 9:**
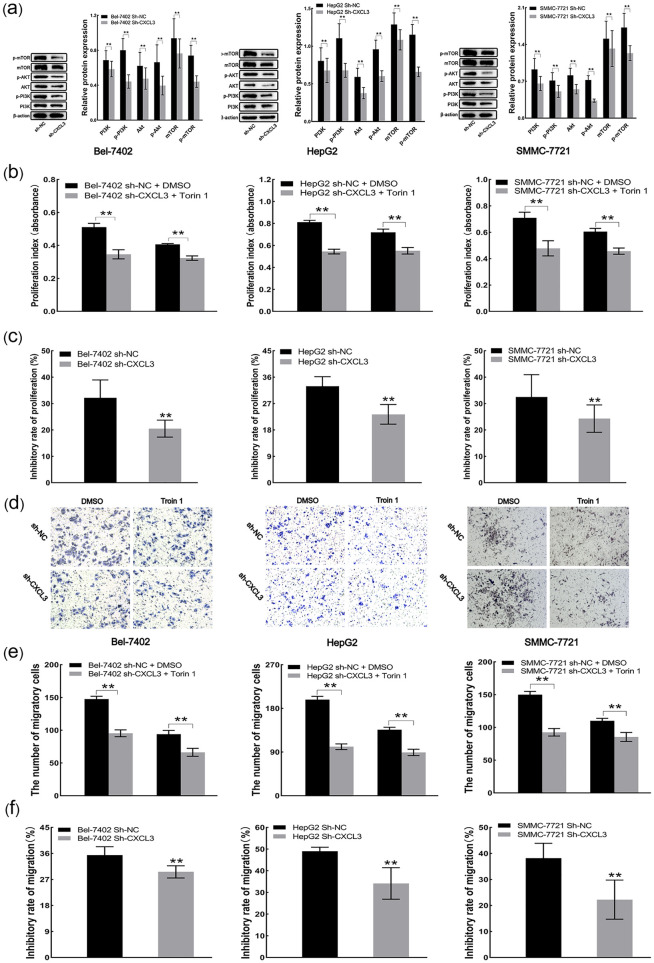
CXCL3 low expression inhibits liver cancer cell malignancy via the mTOR pathway. **(a)** PI3K, p-PI3K, AKT, p-AKT, mTOR and p-mTOR protein levels quantified by Western blot analysis. **(b)** Cell proliferation assessed by CCK-8 assay. **(c)** Cell proliferative inhibition rate evaluated by CCK-8 assay. **(d)** Representative images of Transwell analysis **(e)** Cell migration estimated by Transwell assay. **(f)** Cell migratory inhibition rate determined Transwell assay. **p < 0.01.

Western blot results revealed that sh-CXCL3 cells exhibited significant reductions in the levels of PI3K, p-PI3K, AKT, p-AKT, mTOR and p-mTOR proteins (Fig 9a). CCK-8 assays demonstrated that the proliferation of sh-CXCL3 cells remained suppressed even after administration of Torin 1 (Fig 9b). However, the proliferative inhibition rate exhibited by Torin 1 on sh-NC (negative control) cells was higher than that on sh-CXCL3 cells (Fig 9c). Similarly to the proliferation experiments, Torin 1 displayed a consistent pattern in both inhibiting migration and reducing migratory inhibition rate in sh-CXCL3 cells (Fig 9d-9f).

### 3.10 CXCL3 derived from LX-2 cell regulates the expression of mTOR pathway proteins

**Fig 10:**
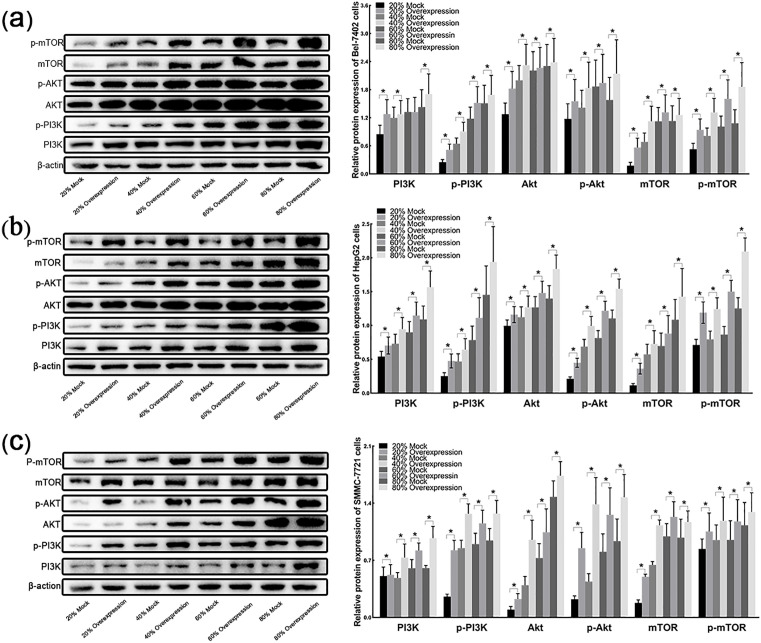
LX-2-derived CXCL3 regulates the expression of mTOR pathway proteins in liver cancer cells. **(a)** Protein levels of PI3K, p-PI3K, AKT, p-AKT, mTOR and p-mTOR in BeL-7402 cells quantified by Western blot analysis. **(b)** Same quantification performed in HepG2 cells. **(c)** Same quantification performed in SMMC-7721 cells. **p < 0.01.

Western blot experiments revealed that, compared to the Bel-7402, HepG2 and SMMC-7721 cells cultured with 20, 40, 60 and 80% of the LX-2 mock-derived conditioned medium, the levels of PI3K, p-PI3K, AKT, p-AKT, mTOR and p-mTOR proteins were significantly upregulated in the cells cultured with LX-2-overexpression-derived conditioned medium (Fig 10a-10c).

## 4 Discussion

CXCL3 belongs to the CXC chemokine family and was initially identified for its expression in human malignant melanoma cells; it is also referred to as melanoma growth-stimulatory activity alpha [23]. It is now widely accepted that CXCL3 is upregulated in various human tumors, and its high expression has been associated with adverse clinical and pathological characteristics in cancer patients [12]. For example, Ruan et al. reported that high CXCL3 expression may serve as a diagnostic and prognostic biomarker in colon cancer, being linked to increased mortality, tumor thrombus formation, and reduced overall survival [24]. Moreover, CXCL3 has been shown to be enriched in the plasma of colon cancer patients, with elevated levels correlating with aggressive clinical features such as advanced stage, poor differentiation, and lymph node metastasis [25]. Our previous study also demonstrated a direct association between CXCL3 upregulation and improved diagnostic accuracy in colon cancer [14]. Consistent with these findings, other studies have reported similar associations between high CXCL3 expression and tumor progression or poor prognosis in various cancer types [26,27]. In alignment with these observations, the present study revealed that CXCL3 expression is significantly elevated in liver cancer tissues and is associated with more advanced clinical staging and reduced overall survival, indicating that CXCL3 may contribute to liver cancer development.

The tumor microenvironment is a complex and dynamic system composed of tumour cells, immune cells, and stromal cells. Communication among these cellular components is mediated by various soluble factors, including cytokines, chemokines, growth factors, and matrix-modifying enzymes [12]. Numerous studies have shown that chemokines play an essential role in attracting and guiding the migration of various immune cells into the tumor microenvironment [10]. Several chemokines have been reported to induce the infiltration of immune cell populations such as myeloid-derived suppressor cells, tumor-associated macrophages, tumor-associated neutrophils, and regulatory T cells, which can contribute to immune evasion and enhance the malignant potential of tumors [8]. Conversely, chemokines may also participate in anti-tumor immune responses by recruiting CD4-positive T cells, CD8-positive T cells, and natural killer cells, enabling these effector cells to suppress tumor growth [8]. In our study, we observed a positive correlation between CXCL3 expression and infiltration of macrophages, neutrophils, B cells, CD4-positive T cells, CD8-positive T cells, and dendritic cells in liver cancer tissues. It has been proposed that many chemokines attract monocytes and neutrophils into the tumor microenvironment, where they differentiate into tumour-associated macrophages and tumor-associated neutrophils, respectively, and thereby regulate tumor immune responses [28]. Tumor-associated macrophages are often the most abundant immune cells within the tumor microenvironment, sometimes accounting for over 50% of the total tumor volume, and they are typically associated with poorer clinical outcomes across a range of cancers [29]. CXC chemokines secreted by tumor-associated macrophages have been shown to facilitate cancer progression and metastasis [20].

In pancreatic ductal adenocarcinoma, Sun et al. reported a CXCL3- and CXCR2-dependent interaction between tumor-associated macrophages and cancer-associated fibroblasts. They found that macrophages, as primary producers of CXCL3, increased CXCL3 expression in response to interleukin-33 stimulation. Meanwhile, the receptor CXCR2 was predominantly expressed by cancer-associated fibroblasts. Activation of CXCR2 by CXCL3 promoted a phenotypic transition from fibroblasts to myofibroblast-like cancer-associated fibroblasts, marked by α-smooth muscle actin expression, ultimately promoting tumor metastasis [30]. In addition, our current study revealed that CXCL3 expression is positively correlated with the expression of other CXCR2-binding chemokines, including CXCL1, CXCL5, and CXCL8. These chemokines are frequently overexpressed in human cancers and have been implicated in promoting tumor growth, cell migration, and angiogenesis [31]. Taken together, these findings suggest that CXCL3 may facilitate the malignant progression of liver cancer by regulating the expression of CXCR2 ligands and coordinating immune cell recruitment within the tumor microenvironment.

In addition to immune cells and tumor cells, the tumor microenvironment contains specialised connective tissue cells such as fibroblasts and mesenchymal stromal cells [32]. It is increasingly recognised that both tumor and stromal cells contribute to local chemokine production, and that crosstalk between these cells through chemokines plays a critical role in cancer progression. This interaction supports tumor cell proliferation, survival, migration, angiogenesis, and resistance to therapy, operating through both autocrine and paracrine mechanisms [21]. Our previous study in cervical cancer revealed that CXCL3 secreted by both tumor and stromal cells can enhance malignant behaviours through both autocrine and paracrine signalling [33]. Similarly, Kogan-Sakin et al. showed that CXCL3, together with CXCL1 and CXCL2, was secreted by prostatic stromal cells in response to interleukin-1 from epithelial cells, thereby promoting prostate inflammation and facilitating tumor initiation [33]. In line with these findings, our present study demonstrated that both exogenous CXCL3 administration and its overexpression significantly promoted liver cancer cell proliferation and migration. Moreover, CXCL3 secreted by hepatic stromal LX-2 cells was also shown to enhance tumor cell malignancy.

Interactions between chemokines and their corresponding receptors in the tumor microenvironment can activate several key signalling cascades, including the phosphoinositide 3-kinase/protein kinase B/mechanistic target of rapamycin (PI3K/AKT/mTOR) pathway, the extracellular signal-regulated kinase 1 and 2 (ERK1/2) pathway, and the nuclear factor-kappa B (NF-κB) pathway [7,33,34]. Among these, the PI3K/AKT/mTOR pathway plays a crucial role in regulating tumor cell survival, proliferation, metabolism, and migration [27]. Moreover, activation of this pathway has also been linked to resistance to anticancer therapies, further underlining its importance [35]. In our study, both in vitro and in vivo experiments demonstrated that CXCL3 upregulation led to increased expression of PI3K/AKT/mTOR signalling proteins through both autocrine and paracrine mechanisms. Importantly, the tumor-promoting effects of CXCL3 were significantly suppressed by treatment with the mechanistic target of rapamycin (mTOR) inhibitor Torin 1, supporting the notion that targeting this pathway may provide therapeutic benefit in liver cancer.

## 5 Conclusions

**Fig 11:**
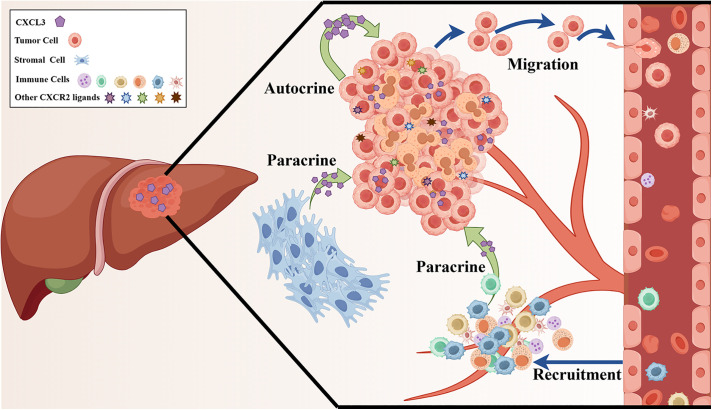
CXCL3 contributes to the malignant progression of liver cancer through its ability to shape the tumor microenvironment.

In summary, this study demonstrates a strong association between elevated CXCL3 expression and the accumulation of immune cells and CXCR2-associated chemokines within the tumor microenvironment of liver cancer. Moreover, CXCL3 derived from both tumor cells and stromal cells was shown to promote the malignant behaviour of tumor cells through activation of the PI3K/AKT/mTOR signalling pathway. These findings suggest that CXCL3 plays a central role in shaping the tumor microenvironment by mediating chemokine-driven autocrine and paracrine interactions among tumor cells, stromal cells, and various types of immune cells (Fig 11).

## Supporting information

S1 File STR analysis. (ZIP)

S2 File Western Blot. (ZIP)

S3 Dataset Bioinformatics data. (ZIP)

S4 File Original data. (ZIP)

S5 File Fig 3 Original images. (ZIP)

S6 File Fig 4 Original images. (ZIP)

S7 File Fig 5 Original images. (ZIP)

S8 File Fig 6 Original images. (ZIP)

S9 File Fig 7 Original images. (ZIP)

S10 File Fig 8 Original images. (ZIP)

S11 File Fig 9 Original images. (ZIP)

## Abbreviations

CXCL3: CXC chemokine ligand 3
HCC: Hepatocellular carcinoma
TCGA: The Cancer Genome Atlas
FBS: fetal bovine serum
RT-qPCR: reverse transcription-quantitative
CCK-8: Cell Counting Kit-8;
EdU: 5-Ethynyl-2´-deoxyuridine
PPBP: pro-platelet basic protein
NC: sh-negative control
CAFs: cancer-associated fibroblasts
myoCAFs: myofibroblast-like CAFs
ERK 1/2: extracellular signal-regulated kinases 1 and 2
NF-κB: nuclear factor-κB
