# Supplementary material for: CXCL3 promotes liver cancer progression by modulating the tumor microenvironment via the PI3K/AKT/mTOR pathway
Source: PLoS One. 2025 Nov 19;20(11):e0334639. doi: 10.1371/journal.pone.0334639 (PMC12629499; doi:10.1371/journal.pone.0334639)
Supplement: S1 File — (ZIP) [file pone.0334639.s001.zip › STR analysis/STR BEL-7402.pdf]

碧云天
Beyotime
碧云天网站 微信公众号
碧云天生物技术/Beyotime Biotechnology
订货热线：400-168-3301或800-8283301
订货e-mail：order@beyotime.com
技术咨询：info@beyotime.com
网址：http://www.beyotime.com

上海碧云天生物技术股份有限公司
质检报告
Certificate of Analysis

产品名称：BEL-7402 (人肝癌细胞)

产品编号：C6109

产品批号：N/A

质检项目	质检标准和要求	质检结果
内外包装	内外包装完整、标签正确、产品包装和产品数量准确	通过
细菌、真菌检测	细胞培养3天后，显微镜下观察，无细菌、真菌污染	通过
支原体检测	细胞培养3天后，培养液上清使用Myco-Lumi™发光法支原体检测试剂盒(C0298)检测，为阴性	通过
STR检测	符合Cellosaurus STR数据，详细见说明书。	通过

检验员：检 21

日期：2024.03.22

上海碧云天生物技术股份有限公司质量部

上海碧云天生物技术股份有限公司 质量部

EV	Cell No.	Cell name	Locus names								
			D5S818	D13S317	D7S820	D16S539	VWA	TH01	AM	TPOX	CSF1PO
	Query (Your Cell)		12,12,	13.3,13.3,	12,12,	9,10,11	16,18,	7,7,	X,X,	12,12,	10,10,
0.97(35/36)	CVCL_5492	BEL-7402	['12', '12']	['13.3', '13.3']	['12', '12']	['9', '10']	['16', '18']	['7', '7']	['X', 'X']	['12', '12']	['10', '10']

https://www.cellosaurus.org/CVCL_5492

STR profile

Source(s): CCRID

Markers:

Amelogenin	X
CSF1PO	10
D2S1338	17
D3S1358	15,18
D5S818	12
D7S820	12
D8S1179	12
D13S317	13.3
D16S539	9,10
D18S51	16
D19S433	13
D21S11	27,28
FGA	18,21
TH01	7
TPOX	12
vWA	16,18

Run an STR similarity search on this cell line
