# Supplementary material for: CXCL3 promotes liver cancer progression by modulating the tumor microenvironment via the PI3K/AKT/mTOR pathway
Source: PLoS One. 2025 Nov 19;20(11):e0334639. doi: 10.1371/journal.pone.0334639 (PMC12629499; doi:10.1371/journal.pone.0334639)
Supplement: S1 File — (ZIP) [file pone.0334639.s001.zip › STR analysis/STR HEPG2.pdf]

人肝癌细胞 Hep G2 [HEPG2]说明书

目录号：SCSP-510

细胞名称：Hep G2 [HEPG2]

细胞描述：人肝癌细胞 Hep G2 [HEPG2]来源于一个 15 岁的白人男性肝癌患者。该细胞表达 3-羟基-3-甲基戊二酰还原酶（3-hydroxy-3-methylglutaryl-CoA）和肝脏三酰甘油脂肪酶（hepatic triglyceride lipase）活性。此细胞不携带乙肝病毒基因组。

物种：白人，男性，15 岁

组织：肝

细胞来源：资源库留存

生物安全等级：BSL-1

完全培养液配方：见下方备注

批次/冻存日期：详见 冻存管/培养瓶 标识

参考传代比例：1:3

参考传代周期：4-6 天

参考换液频率：3-4 天

冻存液配方：完全培养液 95%，DMSO 5%

细胞形态：贴壁生长

支原体检测结果：阴性

STR 鉴定结果：

D5S818: 11,12
D13S317: 9,13
D7S820: 10,10
D16S539: 12,12
vWA: 17,17
THO1: 9,9
Amelogenin: X,Y
TPOX: 8,9
CSF1PO: 10,11

Hep G2 [HEPG2]细胞照片：

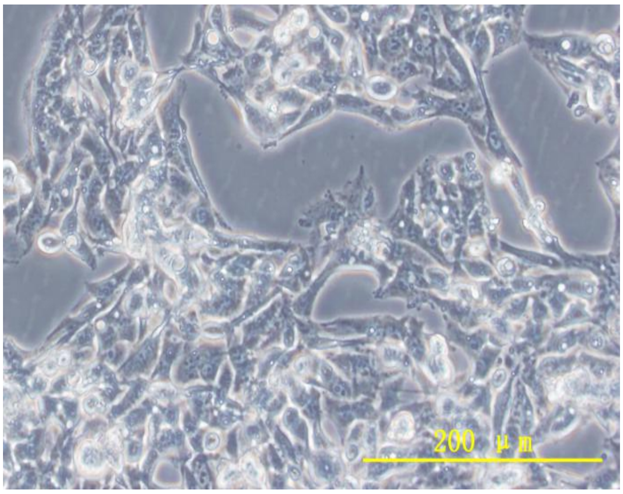

参考文献：

Knowles BB, Aden DP. Human hepatoma derived cell line, process for preparation thereof, and uses therefor. US Patent 4,393,133 dated Jul 12 1983

Schardt C, et al. Characterization of insulin-like growth factor II receptors in human small cell lung cancer cell lines. Exp. Cell Res. 204: 22-29, 1993. PubMed: 8380141

Cuthbert C, et al. Regulation of human apolipoprotein A-I gene expression by gramoxone. J. Biol. Chem. 272: 14954-14960, 1997. PubMed: 9169468

Deleersnyder V, et al. Formation of native hepatitis C virus glycoprotein complexes. J. Virol. 71: 697-704, 1997. PubMed: 8985401

Benn J, et al. Hepatitis B virus HBx protein induces transcription factor AP-1 by activation of extracellular signal-regulated and c-Jun N-terminal mitogen-activated protein kinases. J. Virol. 70: 4978-4985, 1996. PubMed: 8764004

Goodrum FD, et al. Adenovirus early region 4 34-kilodalton protein directs the nuclear localization of the early region 1B 55-kilodalton protein in primate cells. J. Virol. 70: 6323-6335, 1996. PubMed: 8709260

Kolanus W, et al. alphaLbeta2 integrin/LFA-1 binding to ICAM-1 induced by cytohesin-1 a cytoplasmic regulatory molecule. Cell 86: 233-242, 1996. PubMed: 8706128

Lewis W, et al. Fialuridine and its metabolites inhibit DNA polymerase gamma at sites of multiple adjacent analog incorporation, decrease mtDNA abundance, and cause mitochondrial structural defects in cultured hepatoblasts. Proc. Natl. Acad. Sci. USA 93: 3592-3597, 1996. PubMed: 8622980

Jang SI, et al. Activator protein 1 activity is involved in the regulation of the cell type-specific expression from the proximal promoter of the human profilaggrin gene. J. Biol. Chem. 271: 24105-24114, 1996. PubMed: 8798649

Roesler WJ, et al. The alpha-isoform of the CCAAT/enhancer-binding protein is required for mediating cAMP responsiveness of the phosphoenolpyruvate carboxykinase promoter in hepatoma cells. J. Biol. Chem. 271: 8068-8074, 1996. PubMed: 8626491

Lee JH, et al. The proximal promoter of the human transglutaminase 3 gene. J. Biol. Chem. 271: 4561-4568, 1996. PubMed: 8626812

Lieber A, et al. Recombinant adenoviruses with large deletions geneerated by cre-mediated excision exhibit different biological properties compared with first-generation vectors in vitro and in vivo. J. Virol. 70: 8944-8960, 1996. PubMed: 8971024

Dubuisson J, Rice CM. Hepatitis C virus glycoprotein folding: disulfide bond formation and association with calnexin. J. Virol. 70: 778-786, 1996. PubMed: 8551615

Yamaguchi Y, et al. Biochemical characterization and intracellular localization of the Menkes disease protein. Proc. Natl. Acad. Sci. USA 93: 14030-14035, 1996. PubMed: 8943055

Kounas MZ, et al. Cellular internalization and degradation of antithrombin III-thrombin, heparin cofactor II-thrombin, and alpha1-antitrypsin-trypsin complexes is mediated by the low density lipoprotein receptor-related protein. J. Biol. Chem. 271: 6523-6529, 1996. PubMed: 8626456

Klemm DJ, et al. Adenovirus E1A proteins regulate phosphoenolpyruvate carboxykinase gene transcription through multiple mechanisms. J. Biol. Chem. 271: 8082-8088, 1996. PubMed: 8626493

Wu X, et al. Demonstration of a physical interaction between microsomal triglyceride transfer protein and apolipoprotein B during the assembly of ApoB-containing lipoproteins. J. Biol. Chem. 271: 10277-10281, 1996. PubMed: 8626595

Knowles BB, et al. Human hepatocellular carcinoma cell lines secrete the major plasma proteins and hepatitis B surface antigen. Science 209: 497-499, 1980. PubMed: 6248960

Ostlund RE Jr., et al. A sterospecific myo-inositol/D-chiro-inositol transporter in HepG2 liver cells. J. Biol. Chem. 271: 10073-10078, 1996. PubMed: 8626564

Busch SJ, et al. Differential regulation of hepatic triglyceride lipase and 3-hydroxy-3-methylglutaryl-CoA reductase gene expression in a human hepatoma cell line, HepG2. J. Biol. Chem. 265: 22474-22479, 1990. PubMed: 2176219

Darlington GJ, et al. Growth and hepatospecific gene expression of human hepatoma cells in a defined medium. In Vitro Cell. Dev. Biol. 23: 349-354, 1987. PubMed: 3034851

Aden DP, et al. Controlled synthesis of HBsAg in a differentiated human liver carcinoma-derived cell line. Nature 282: 615-616, 1979. PubMed: 233137

Knowles BB, Aden DP. Human hepatoma derived cell line, process for preparation thereof, and uses therefor. US Patent 4,393,133 dated Jul 12 1983

备注：

1. 人肝癌细胞 Hep G2 [HEPG2]完全培养液 配方（100 ml）：

MEM (Invitrogen, 11090081)	87 ml
FBS (Gibco)	10 ml
Glutamax (Invitrogen 35050061)	1 ml
Non-essential Amino Acids, 100× (Invitrogen, 11140050)	1 ml
Sodium Pyruvate 100 mM Solution (Invitrogen 11360070)	1 ml

2. Hep G2 [HEPG2]对培养条件要求较严格，特别是 pH 值和血清质量，否则可能影响细胞凝集数量，应使用高质量低内毒素，未灭火的胎牛血清，可以帮助圆形

的细胞丛簇更好的贴壁及形成单层细胞。细胞内容易有空泡，特别是在融合时。

3.该细胞复苏后可能存在形态不典型的情况，经几次传代后，细胞形态会更加典型。

4.该细胞对血清质量较为敏感，我库建议您使用**优质胎牛血清**进行培养或选择订购我库配套 MEM 完全培养液，货号为 SCSP-651。

5.我库冻存时，每支冻存管约含 7×105 细胞量，体积为 500 μl，预期存活率 70%， 建议复苏至 1 个 T25 培养瓶中。

中国科学院典型培养物保藏委员会细胞库/干细胞库
