# Supplementary material for: CXCL3 promotes liver cancer progression by modulating the tumor microenvironment via the PI3K/AKT/mTOR pathway
Source: PLoS One. 2025 Nov 19;20(11):e0334639. doi: 10.1371/journal.pone.0334639 (PMC12629499; doi:10.1371/journal.pone.0334639)
Supplement: S1 File — (ZIP) [file pone.0334639.s001.zip › STR analysis/STR SMMC-7721.pdf]

碧云天
Beyotime
碧云天网站 微信公众号
碧云天生物技术/Beyotime Biotechnology
订货热线：400-168-3301或800-8283301
订货e-mail：order@beyotime.com
技术咨询：info@beyotime.com
网址：http://www.beyotime.com

上海碧云天生物技术股份有限公司
质 检 报 告
Certificate of Analysis

产品名称：SMMC-7721 (人肝癌细胞)

产品编号：C6865

产品批号：N/A

质检项目	质检标准和要求	质检结果
内外包装	内外包装完整、标签正确、产品包装和产品数量准确	通过
细菌、真菌检测	细胞培养3天后，显微镜下观察，无细菌、真菌污染	通过
支原体检测	细胞培养3天后，培养液上清使用Myco-Lumi™发光法支原体检测试剂盒(C0298)检测，为阴性	通过
STR检测	符合Cellosaurus STR数据，详细见说明书。	通过

检验员：检 86

日期：2024.03.22

上海碧云天生物技术股份有限公司质量部

上海碧云天生物技术股份有限公司 质量部

比对数据库：https://www.cellosaurus.org/CVCL_0534

STR profile

Source(s): CCRID; PubMed=26116706

Markers:

Amelogenin	X
CSF1PO	10
D2S1338	17
D3S1358	15,18
D5S818	11,12
D6S1043	18
D7S820	12
D8S1179	12
D12S391	20,25,26
D13S317	13.3
D16S539	9,10
D18S51	16
D19S433	13
D21S11	27,28
FGA	18,21
Penta D	8,15
Penta E	7,17
TH01	7
TPOX	12
vWA	16,18

Run an STR similarity search on this cell line
