# Supplementary material for: CXCL3 promotes liver cancer progression by modulating the tumor microenvironment via the PI3K/AKT/mTOR pathway
Source: PLoS One. 2025 Nov 19;20(11):e0334639. doi: 10.1371/journal.pone.0334639 (PMC12629499; doi:10.1371/journal.pone.0334639)

Figure 10.(a) Bel-7402, β-actin, original figure

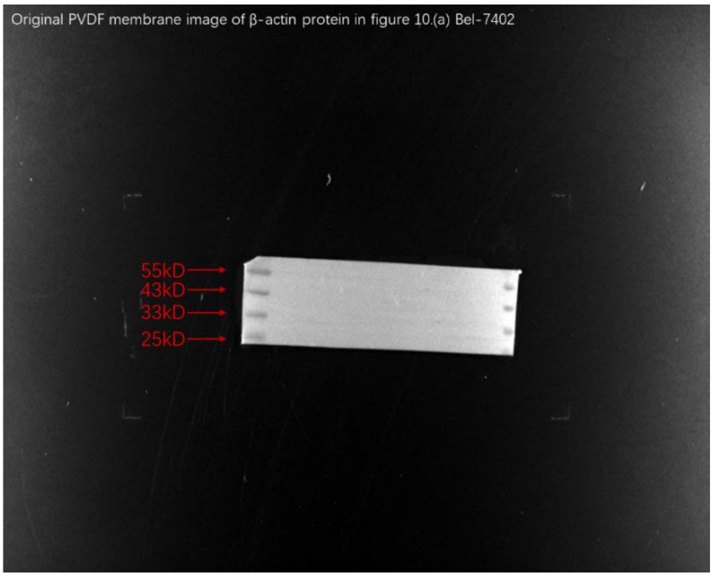

Figure 10.(a) Bel-7402, β-actin, exposure picture 1

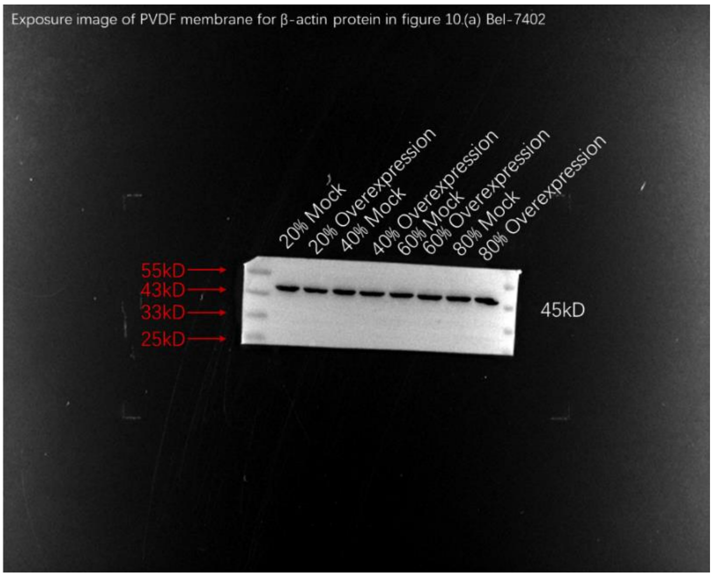

Figure 10.(a) Bel-7402, β-actin, exposure picture 2

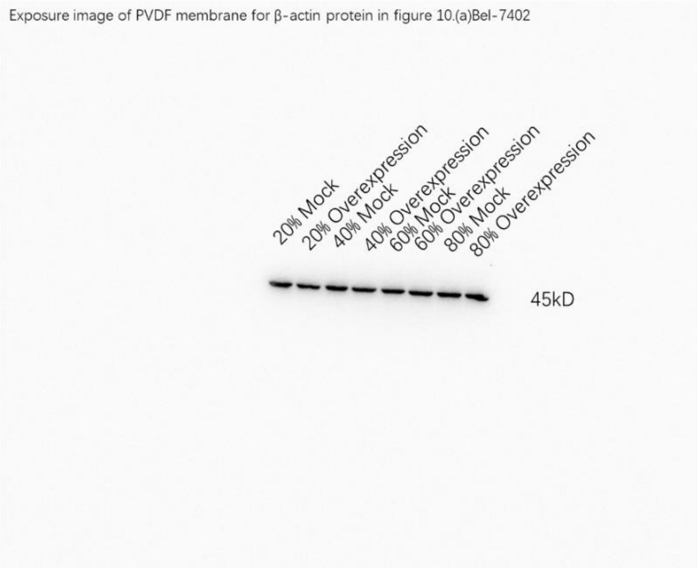

Figure 10.(a) Bel-7402, AKT, original figure

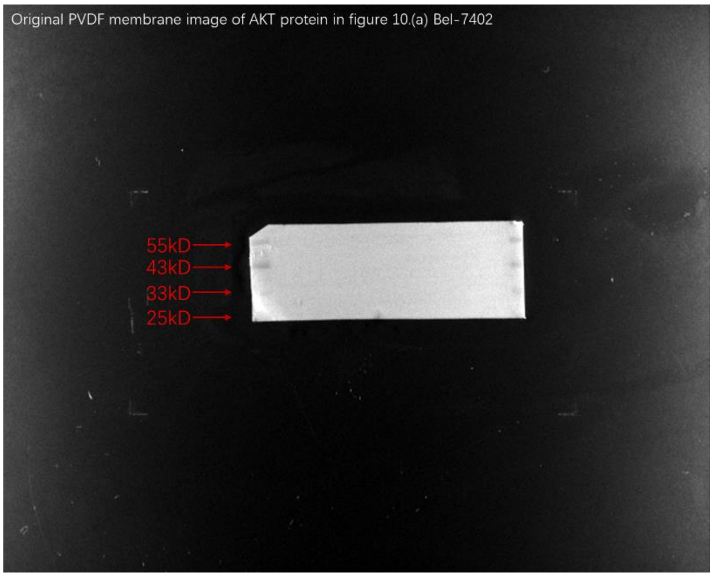

Figure 10.(a) Bel-7402, AKT, exposure picture 1

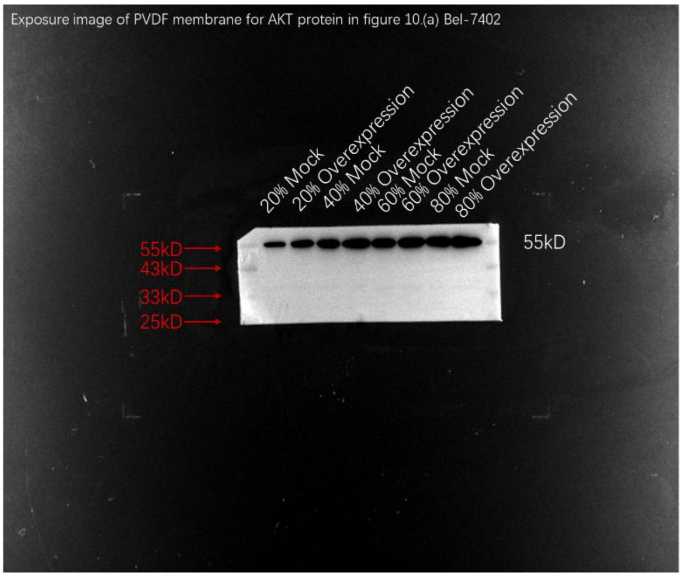

Figure 10.(a) Bel-7402, AKT, exposure picture 2

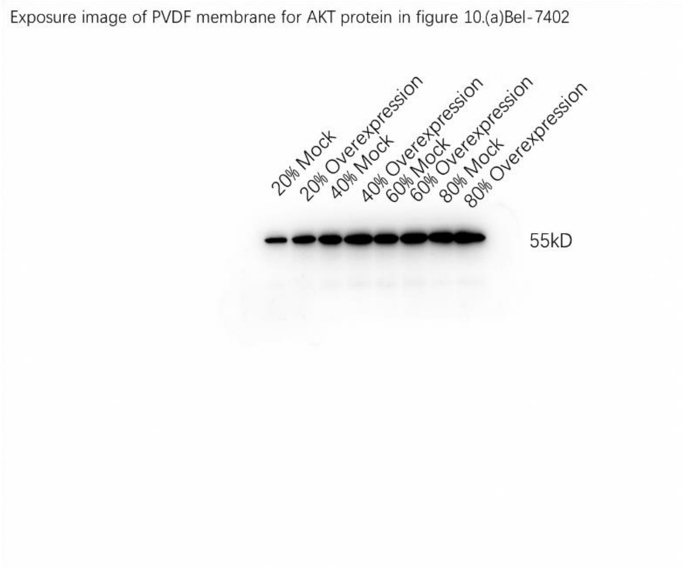

Figure 10.(a) Bel-7402, p-AKT, exposure picture 1

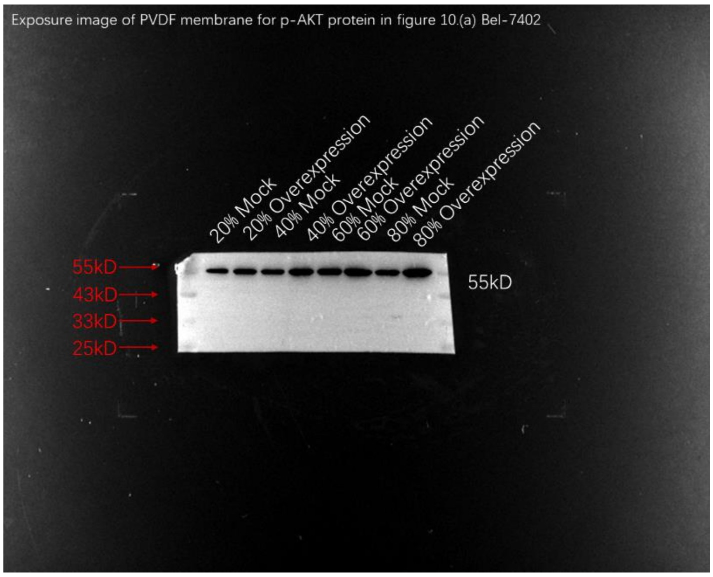

Figure 10.(a) Bel-7402, p-AKT, exposure picture 2

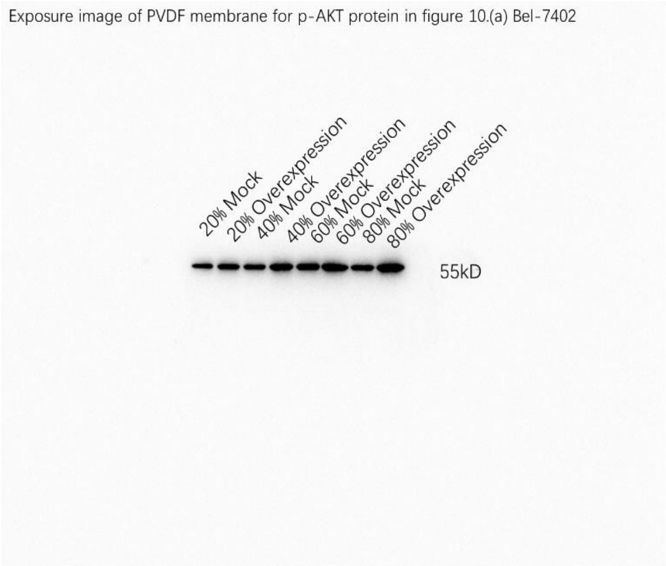

Figure 10.(a) Bel-7402, mTOR, original figure

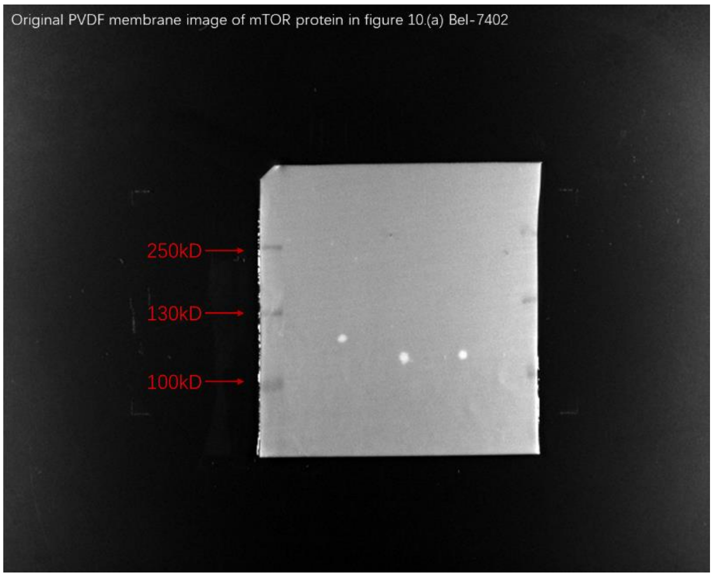

Figure 10.(a) Bel-7402, mTOR, exposure picture 1

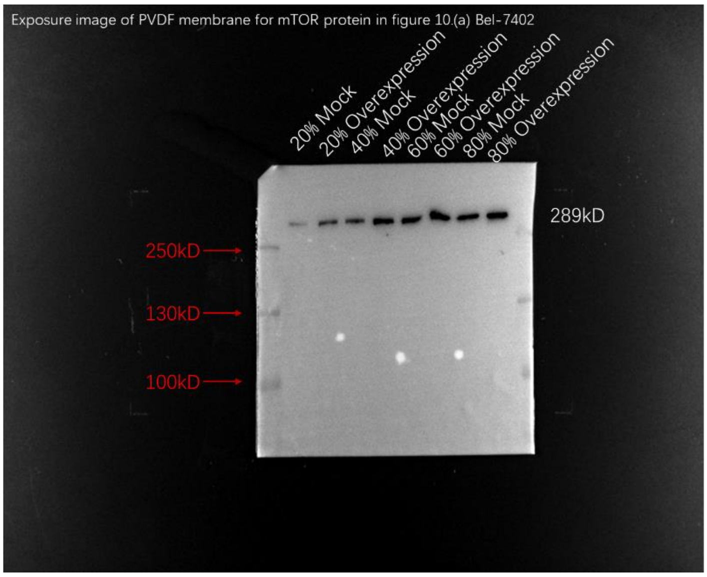

Figure 10.(a) Bel-7402, mTOR, exposure picture 2

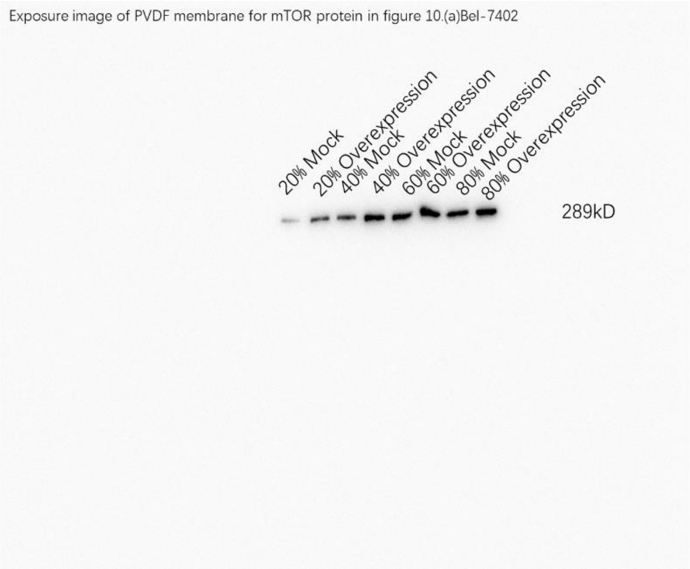

Figure 10.(a) Bel-7402, p-mTOR, original figure

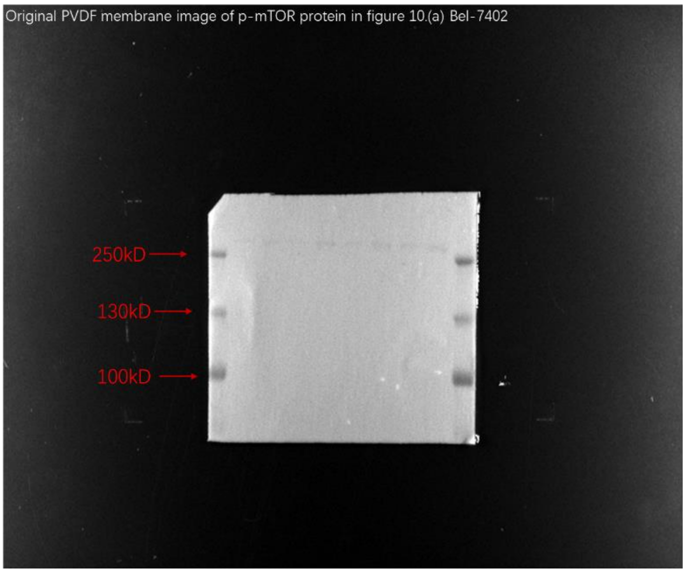

Figure 10.(a) Bel-7402, p-mTOR, exposure picture 1

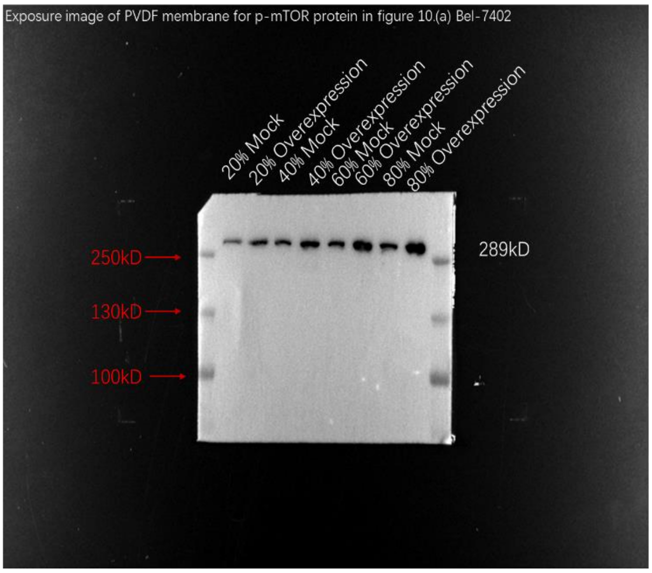

Figure 10.(a) Bel-7402, p-mTOR, exposure picture 2

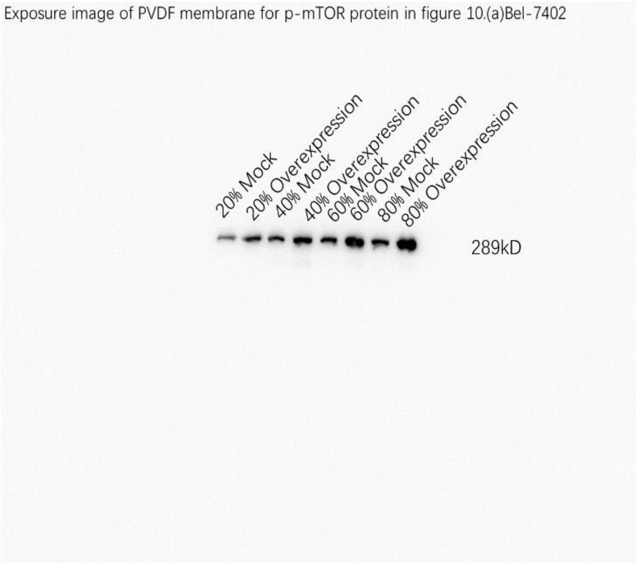

Figure 10.(a) Bel-7402, PI3K, original figure

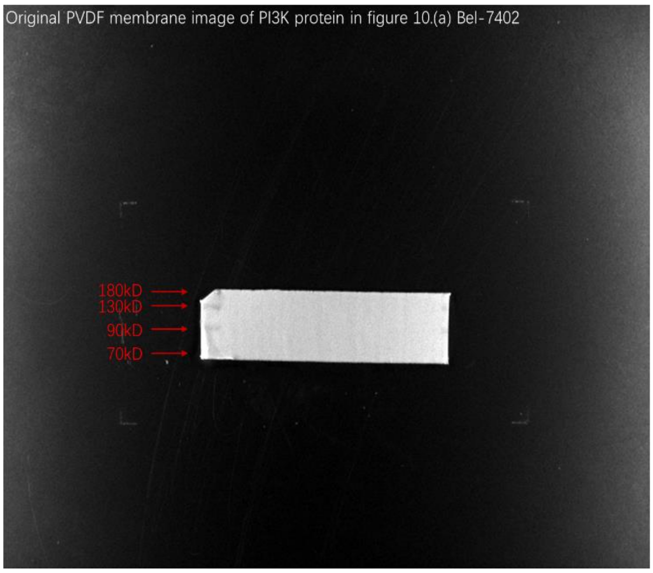

Figure 10.(a)   Bel-7402, PI3K, exposure picture 1

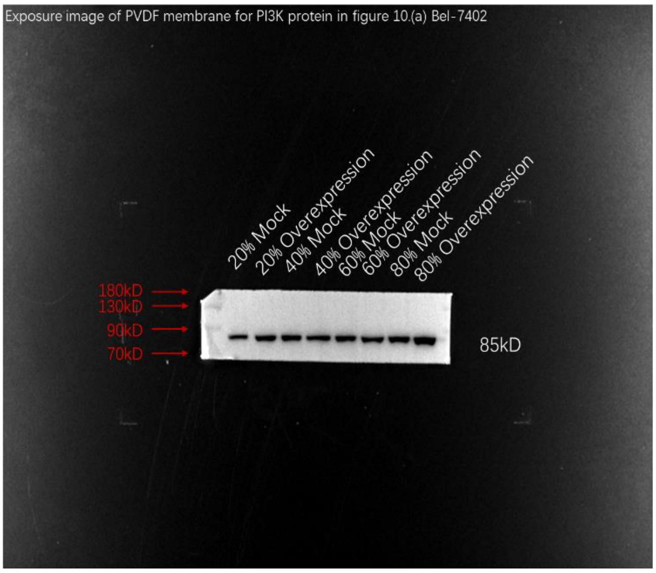

Figure 10.(a)   Bel-7402, PI3K, exposure picture 2

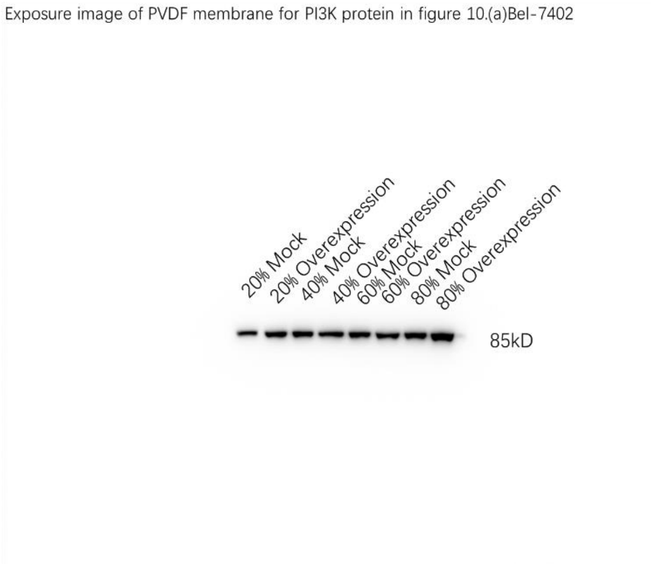

Figure 10.(a)   Bel-7402, p-PI3K, original figure

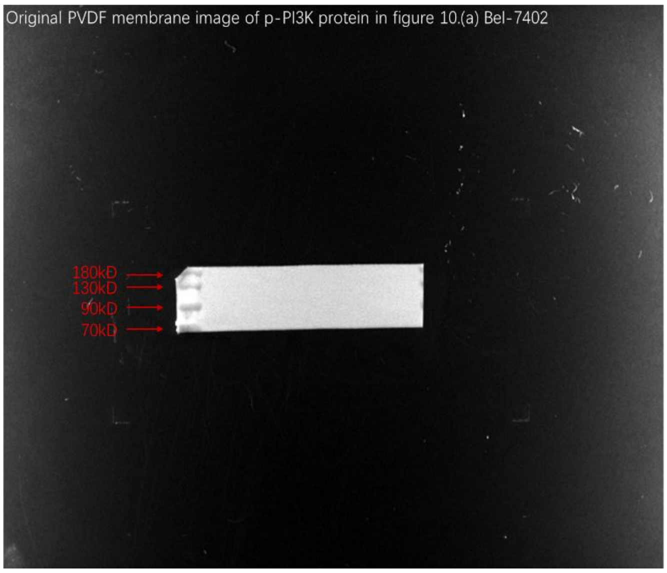

Figure 10.(a)　　　Bel-7402, p-PI3K, exposure picture 1

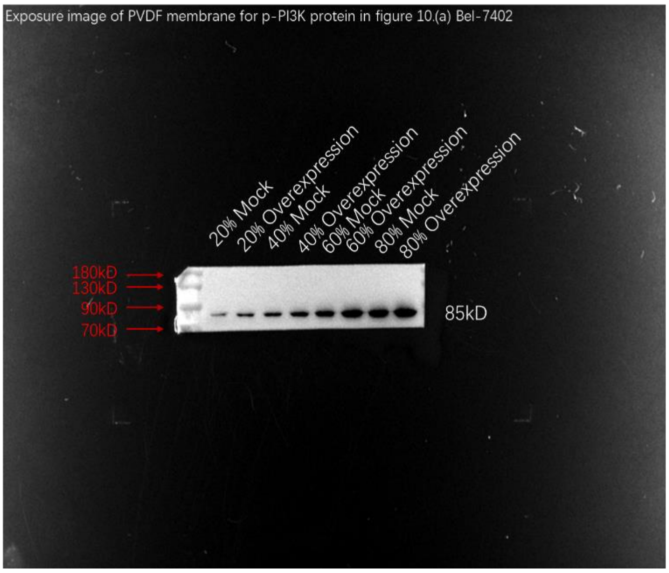

Figure 10.(a)　　　Bel-7402, p-PI3K, exposure picture 2

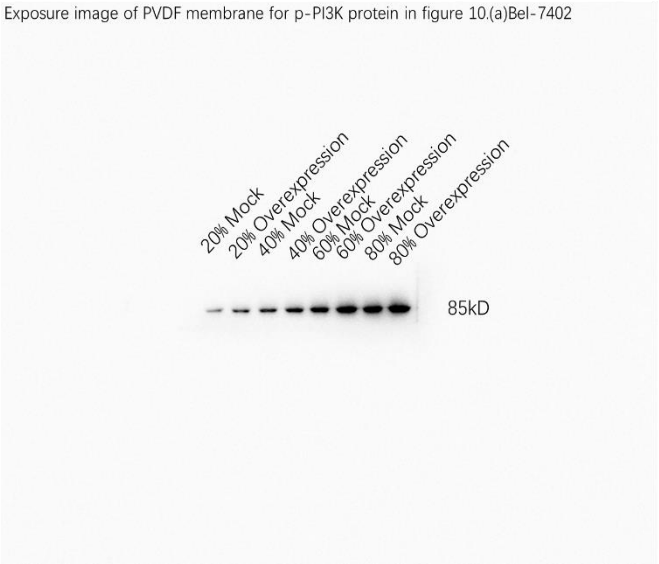

Figure 10.(b)   HepG2, β-actin, original figure

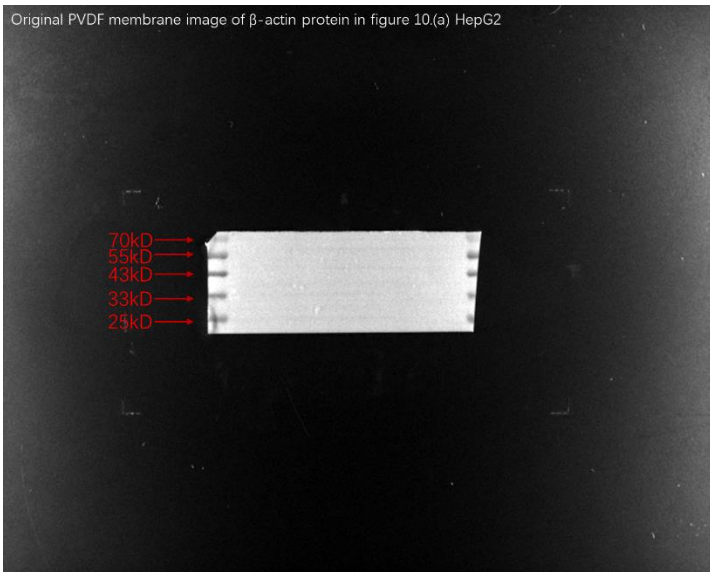

Figure 10.(b)   HepG2, β-actin, exposure picture 1

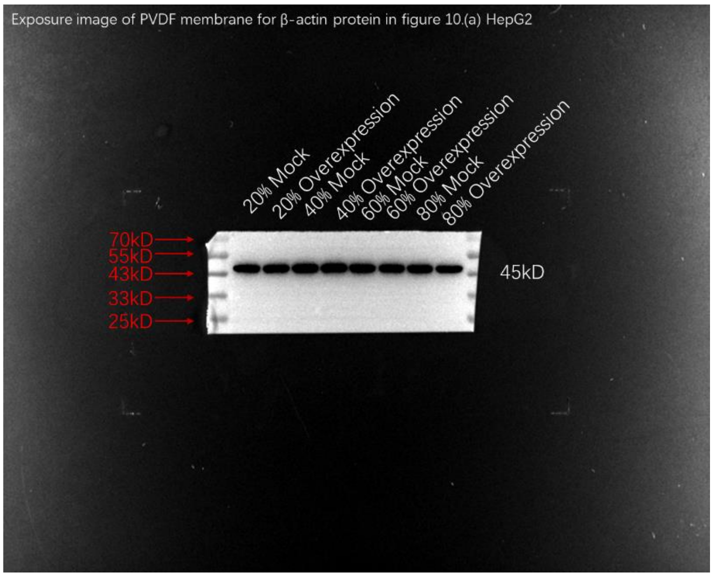

Figure 10.(b)   HepG2, β-actin, exposure picture 2

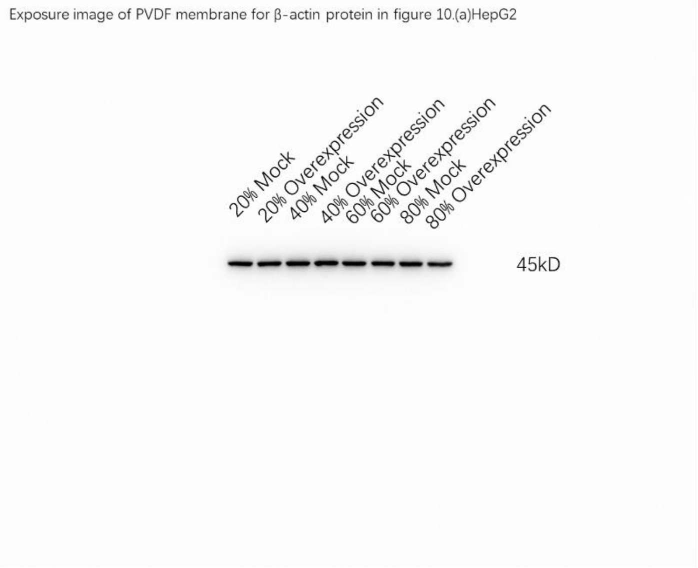

Figure 10.(b) HepG2, AKT, original figure

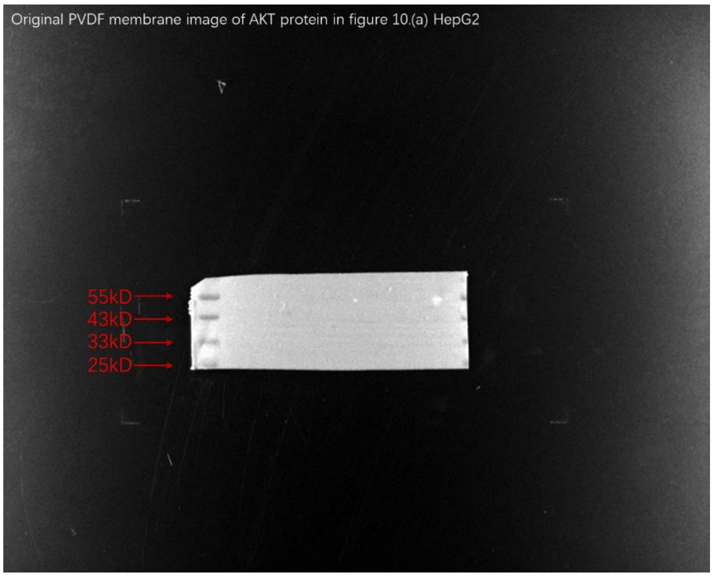

Figure 10.(b) HepG2, AKT, exposure picture 1

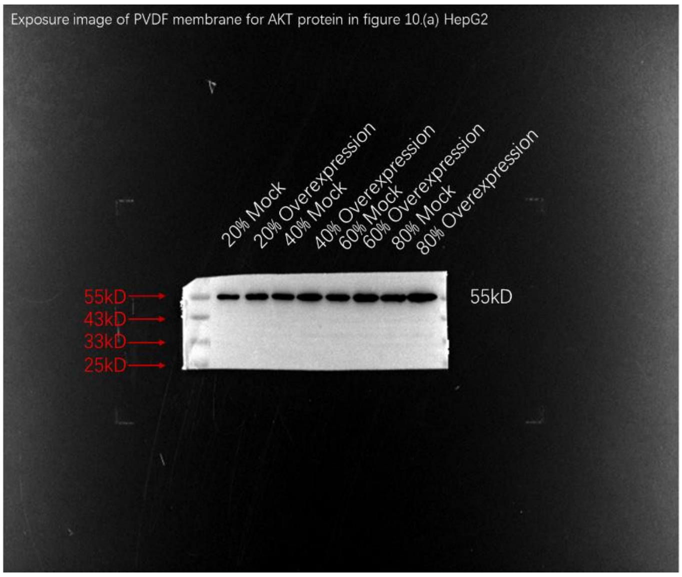

Figure 10.(b) HepG2, AKT, exposure picture 2

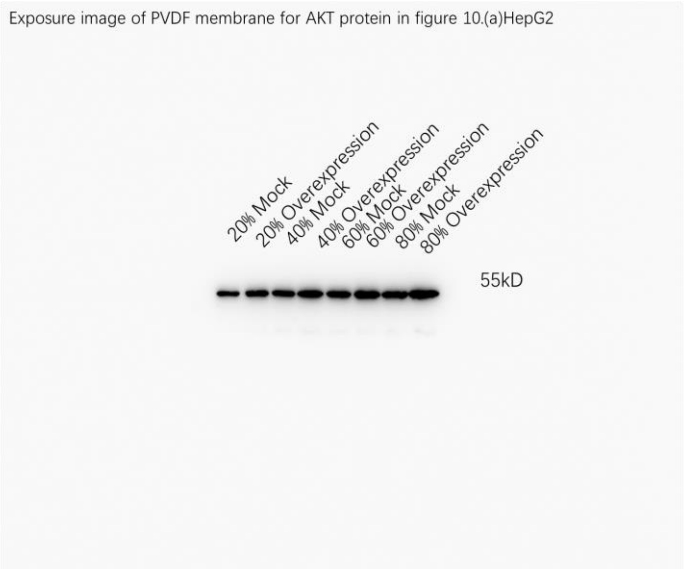

Figure 10.(b) HepG2, p-AKT, original figure

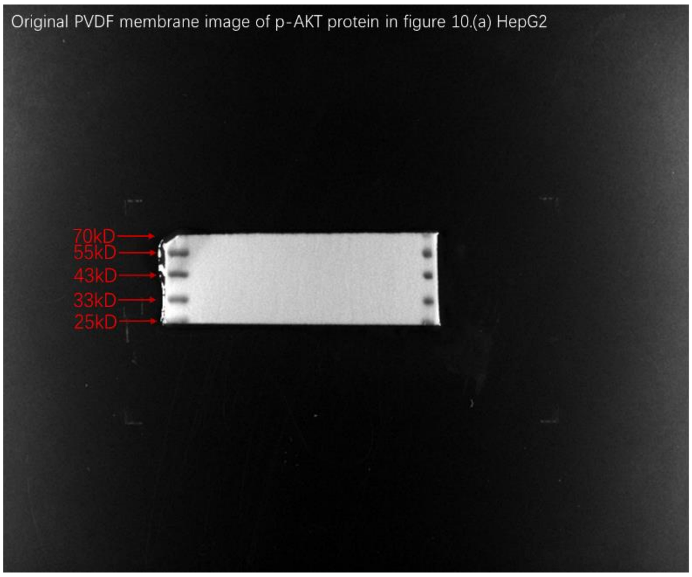

Figure 10.(b) HepG2, p-AKT, exposure picture 1

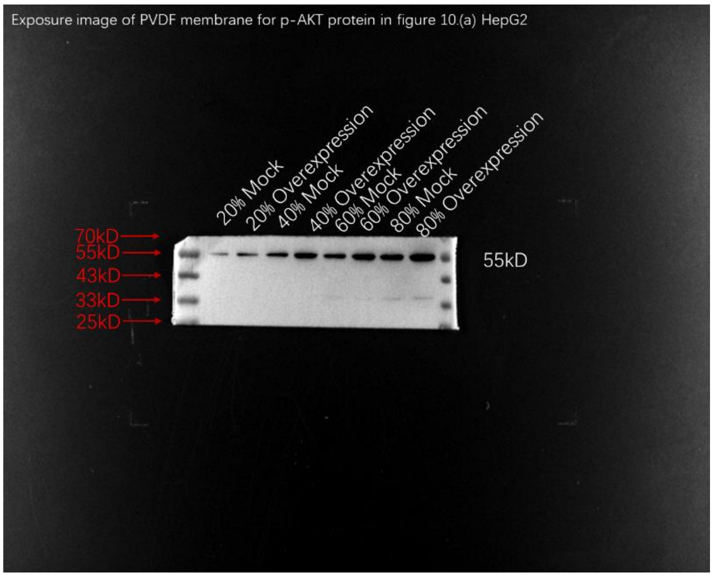

Figure 10.(b)　　HepG2, p-AKT, exposure picture 2

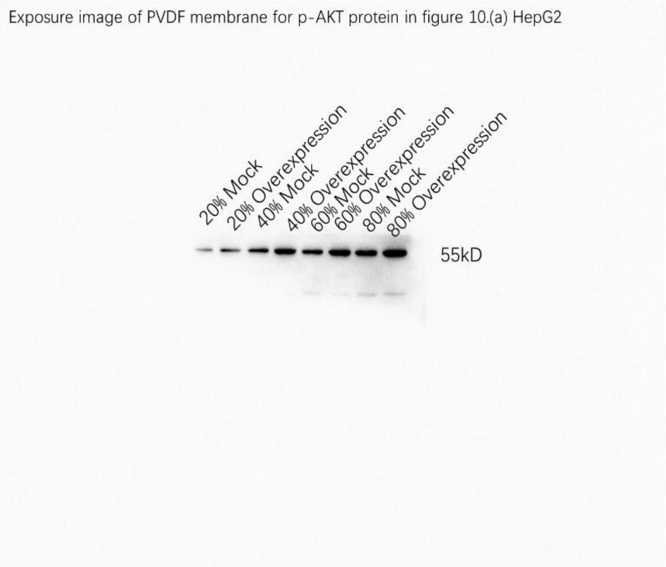

Figure 10.(b)　　HepG2, mTOR, original figure

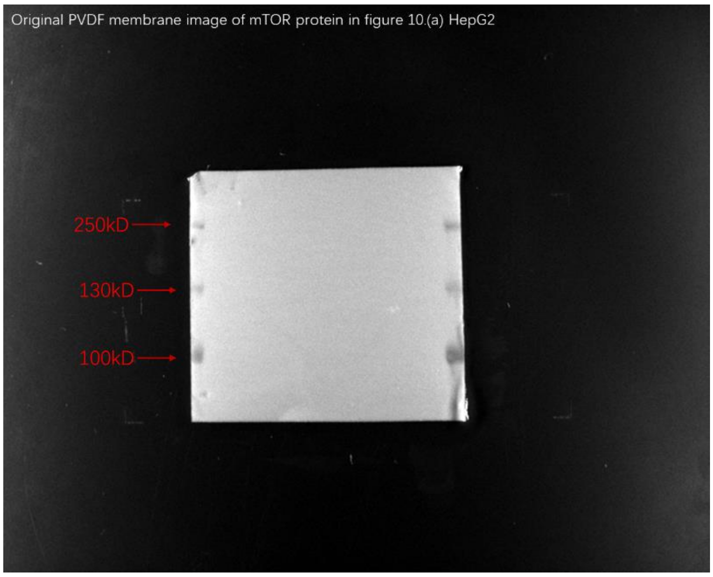

Figure 10.(b) HepG2, mTOR, exposure picture 1

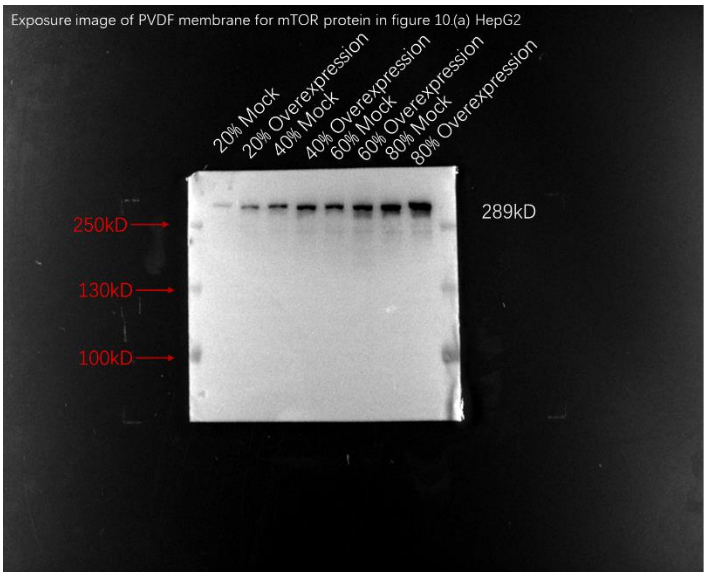

Figure 10.(b) HepG2, mTOR, exposure picture 2

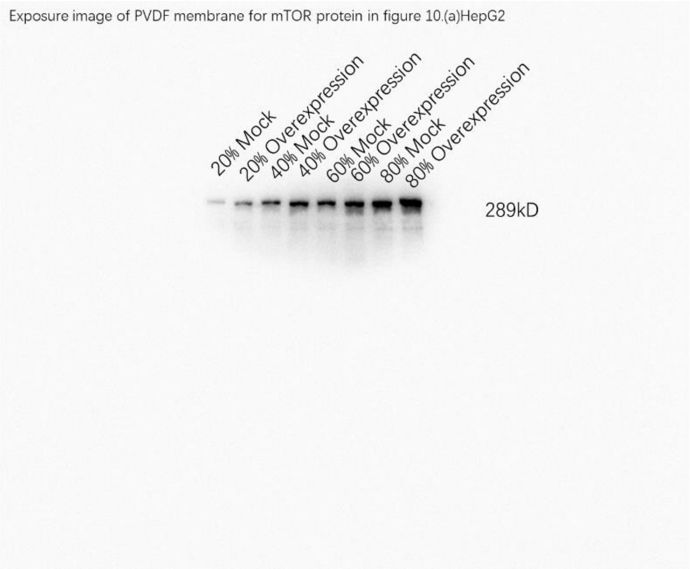

Figure 10.(b) HepG2, p-mTOR, original figure

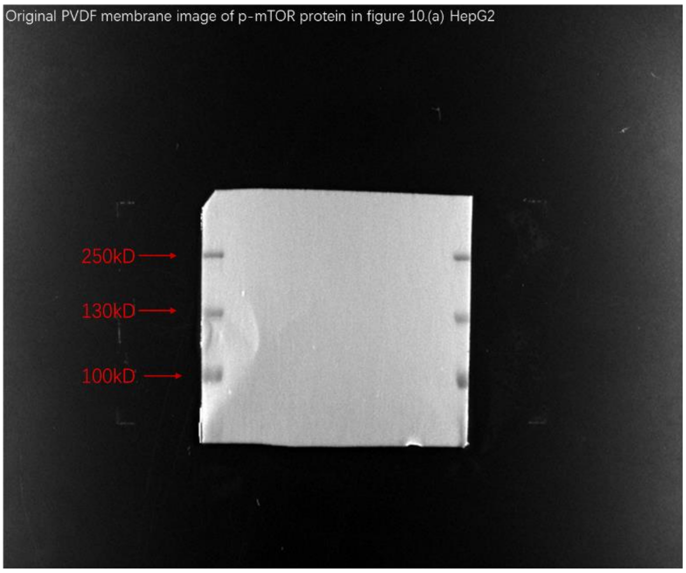

Figure 10.(b) HepG2, p-mTOR, exposure picture 1

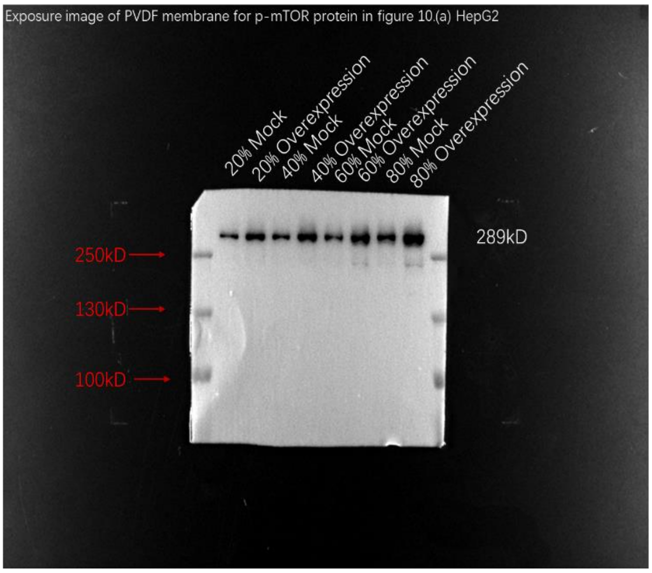

Figure 10.(b) HepG2, p-mTOR, exposure picture 2

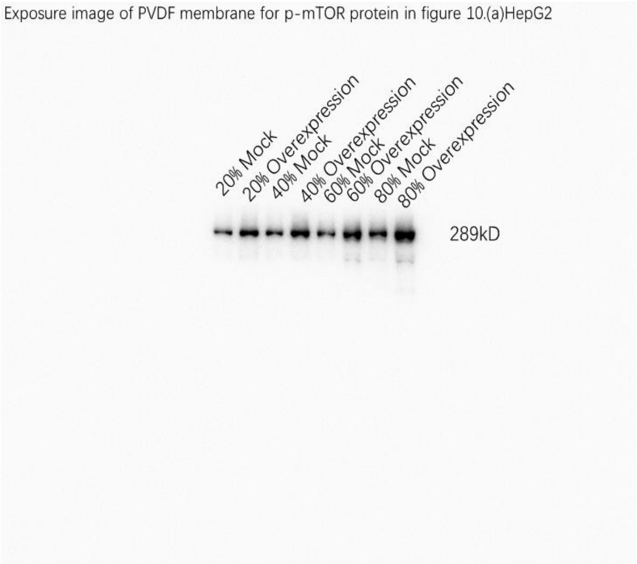

Figure 10.(b) HepG2, PI3K, original figure

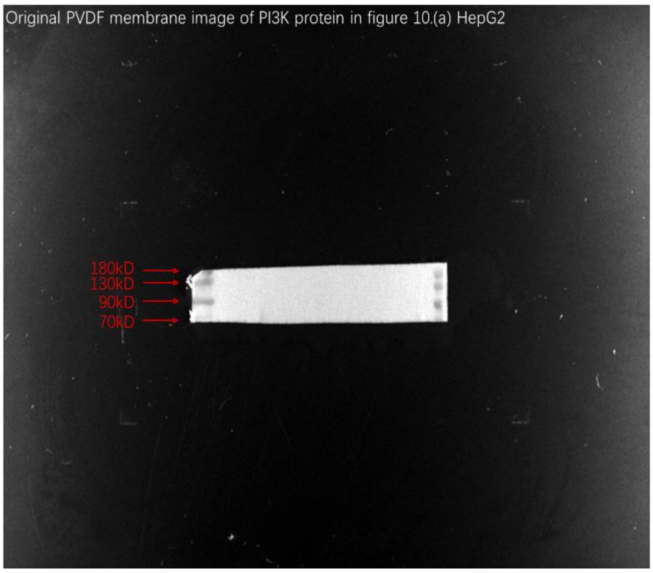

Figure 10.(b) HepG2, PI3K, exposure picture 1

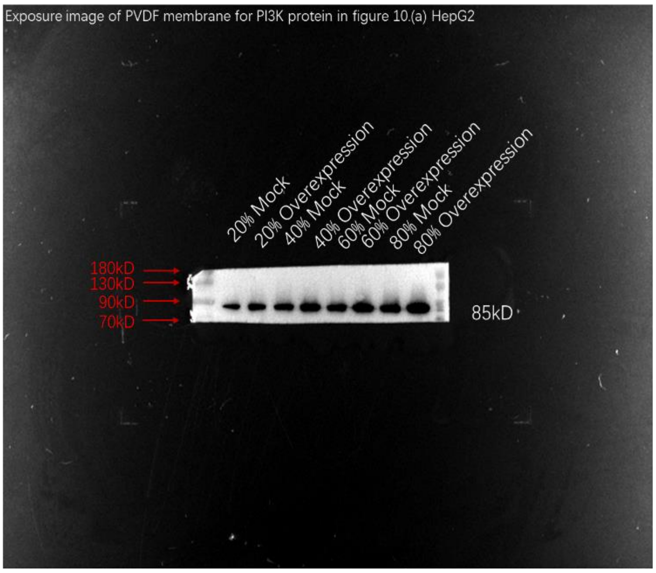

Figure 10.(b) HepG2, PI3K, exposure picture 2

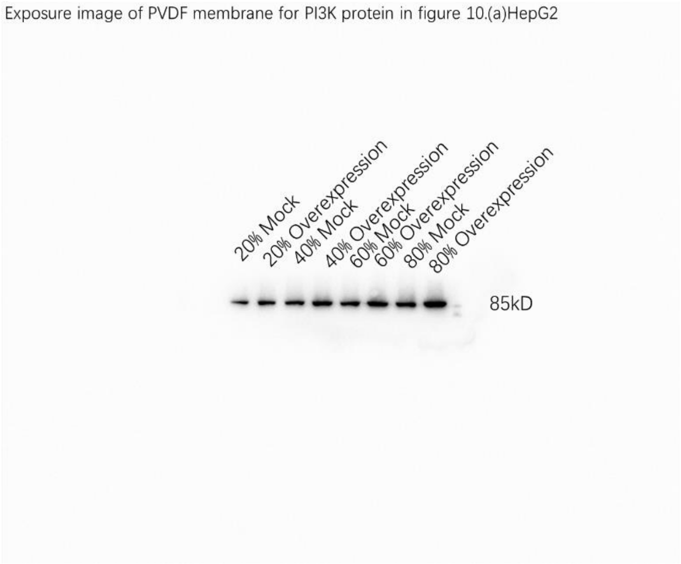

Figure 10.(b) HepG2, p-PI3K, original figure

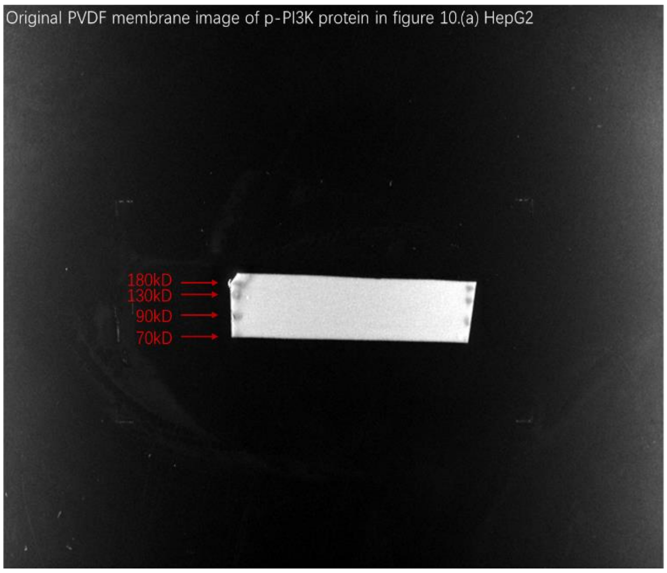

Figure 10.(b) HepG2, p-PI3K, exposure picture 1

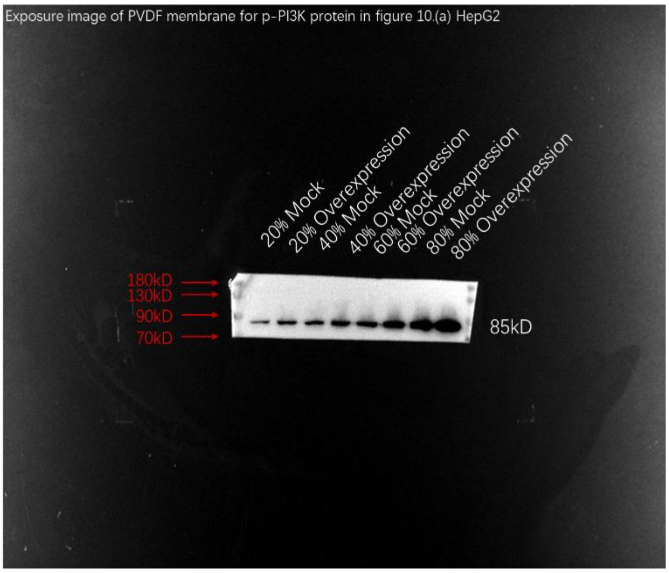

Figure 10.(b) HepG2, p-PI3K, exposure picture 2

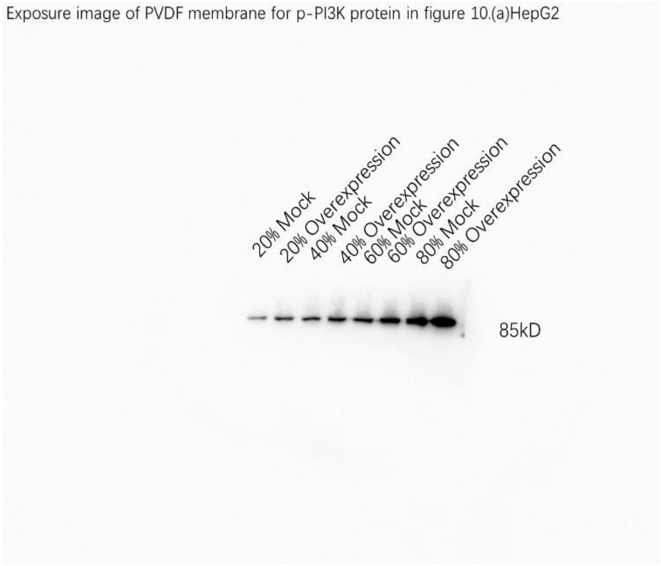

Figure 10.(c) SMMC-7721, β-actin, original figure

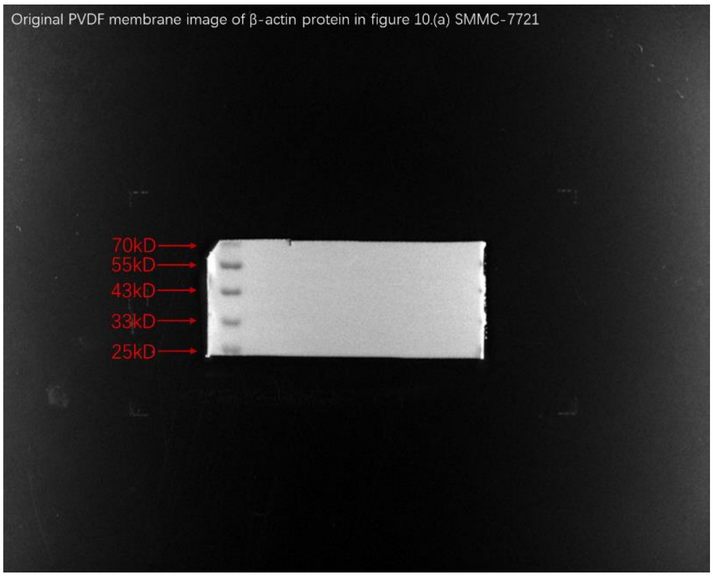

Figure 10.(c) SMMC-7721, β-actin, exposure picture 1

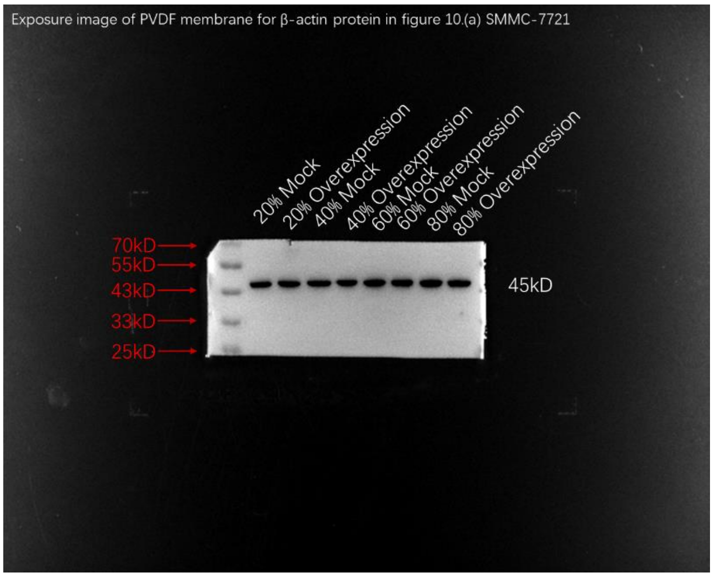

Figure 10.(c) SMMC-7721, β-actin, exposure picture 2

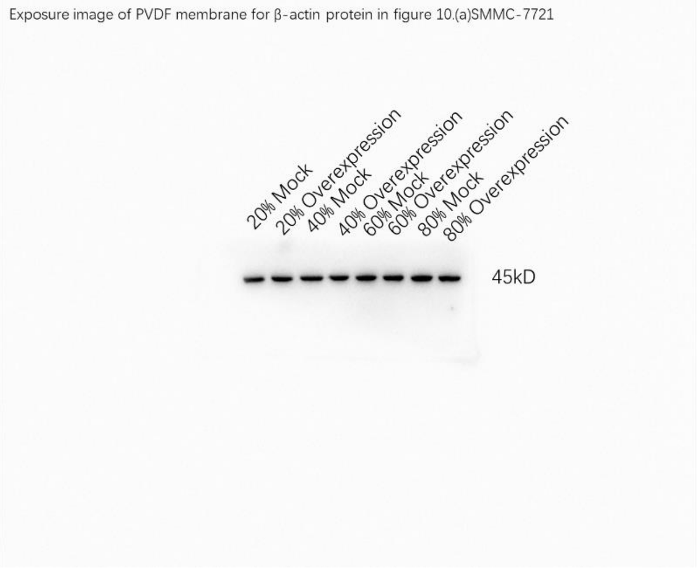

Figure 10.(c) SMMC-7721, AKT, original figure

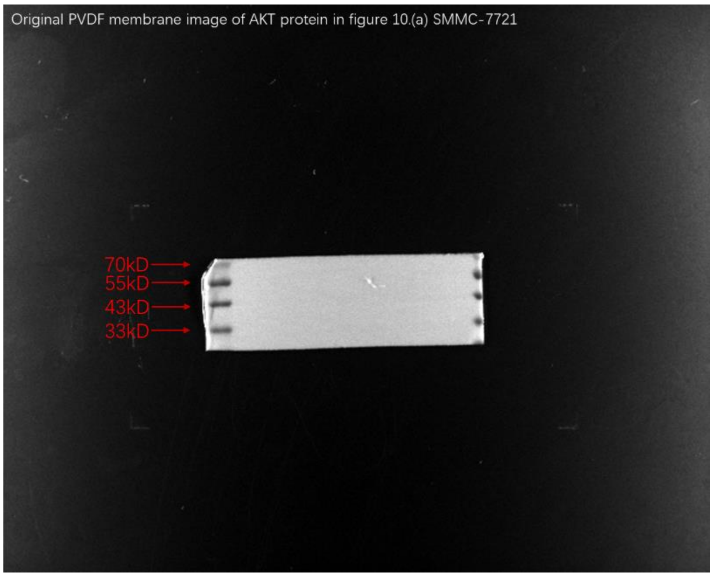

Figure 10.(c) SMMC-7721, AKT, exposure picture 1

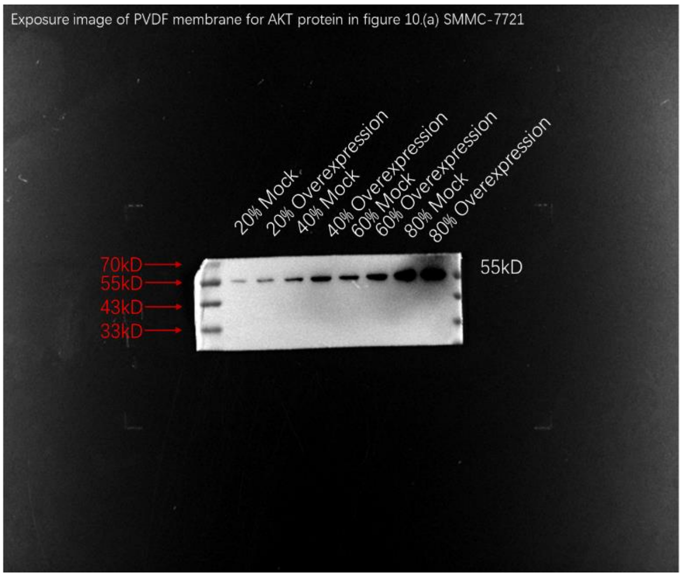

Figure 10.(c) SMMC-7721, AKT, exposure picture 2

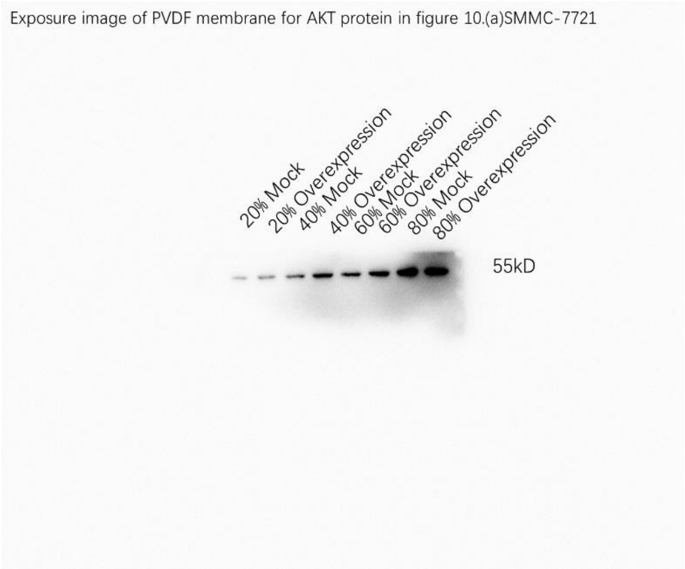

Figure 10.(c) SMMC-7721, p-AKT, original figure

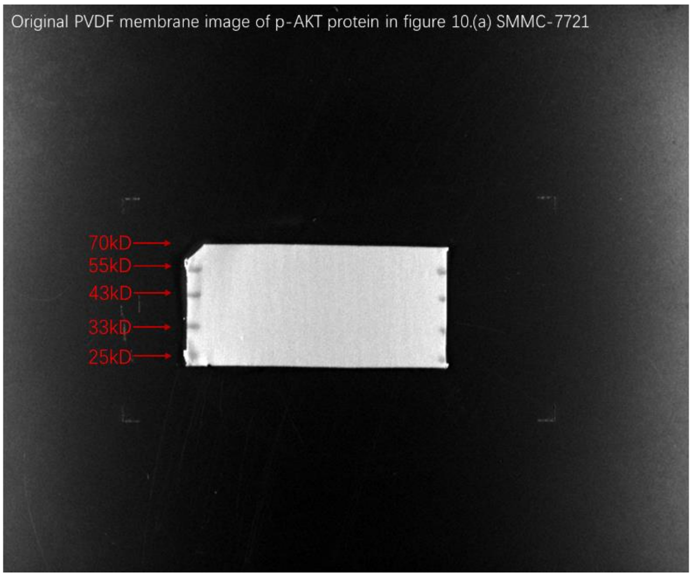

Figure 10.(c) SMMC-7721, p-AKT, exposure picture 1

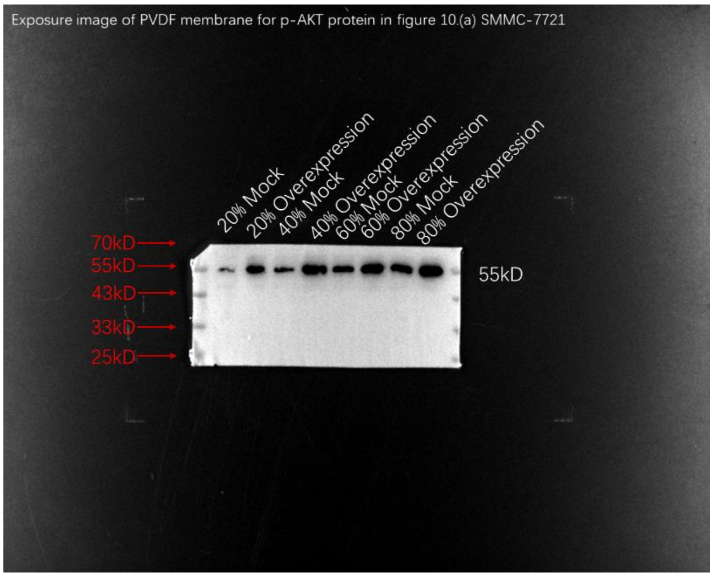

Figure 10.(c) SMMC-7721, p-AKT, exposure picture 2

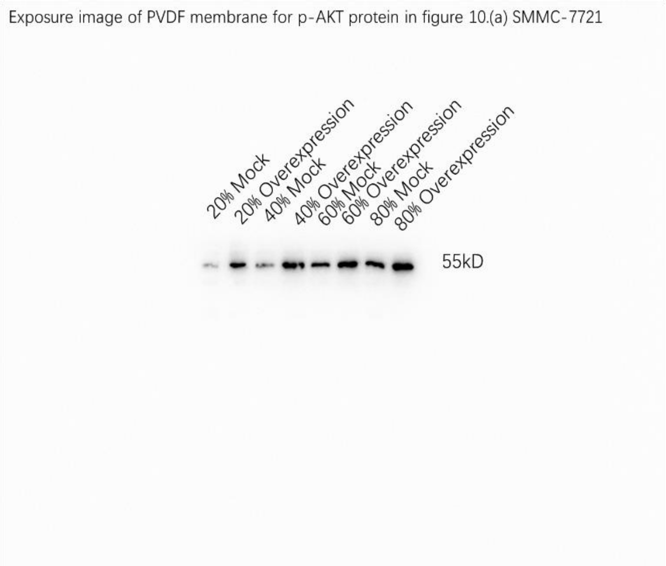

Figure 10.(c) SMMC-7721, mTOR, original figure

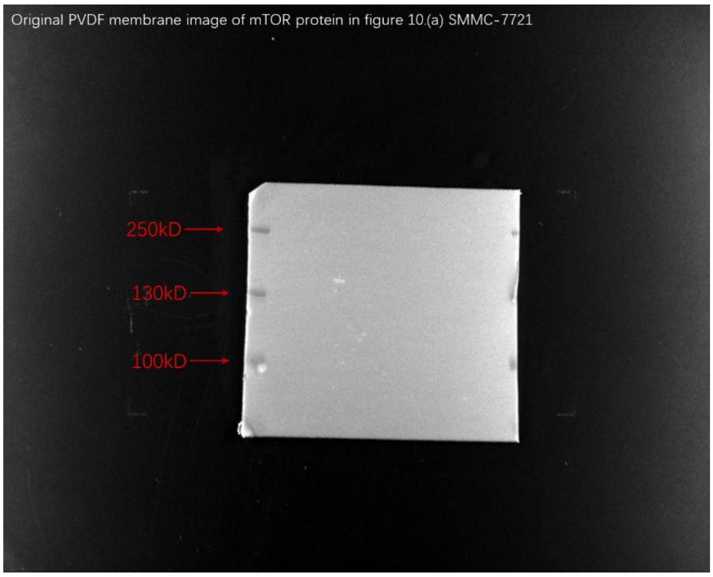

Figure 10.(c) SMMC-7721, mTOR, exposure picture 1

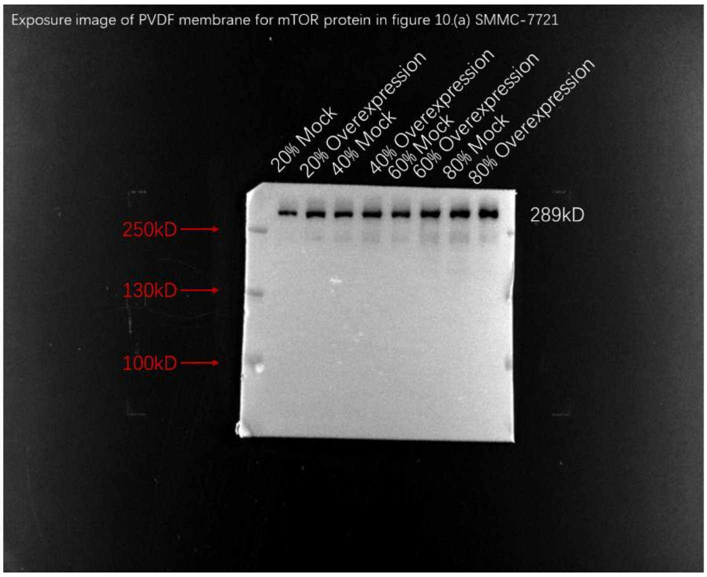

Figure 10.(c) SMMC-7721, mTOR, exposure picture 2

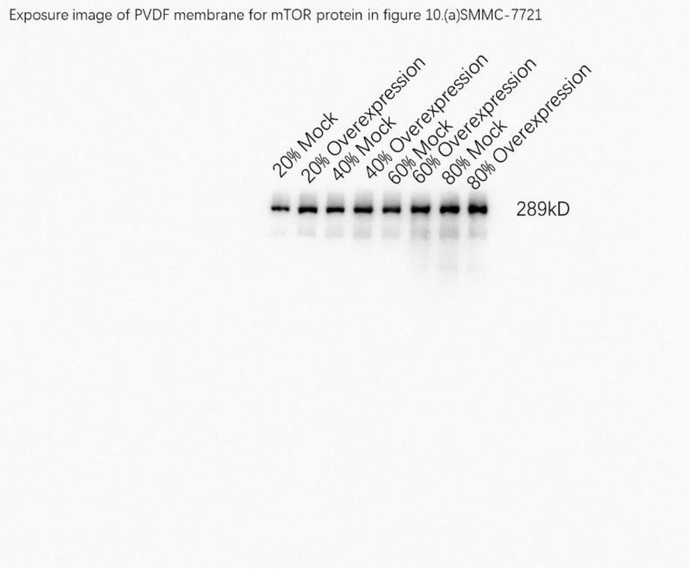

Figure 10.(c) SMMC-7721, p-mTOR, original figure

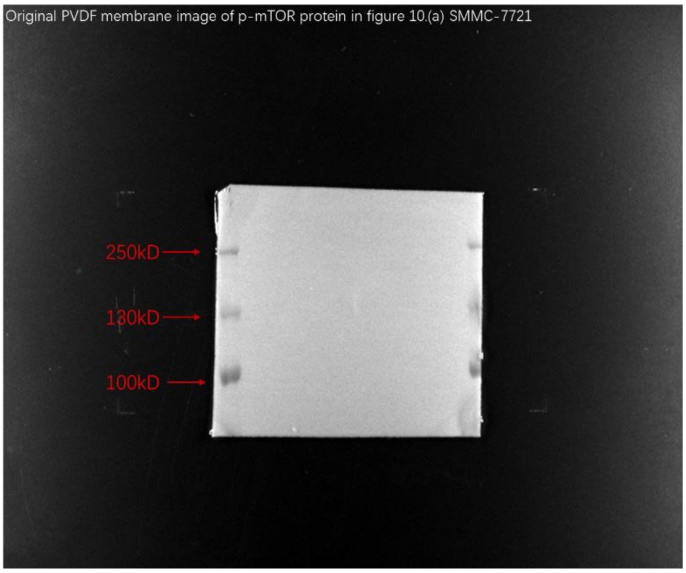

Figure 10.(c)   SMMC-7721, p-mTOR, exposure picture 1

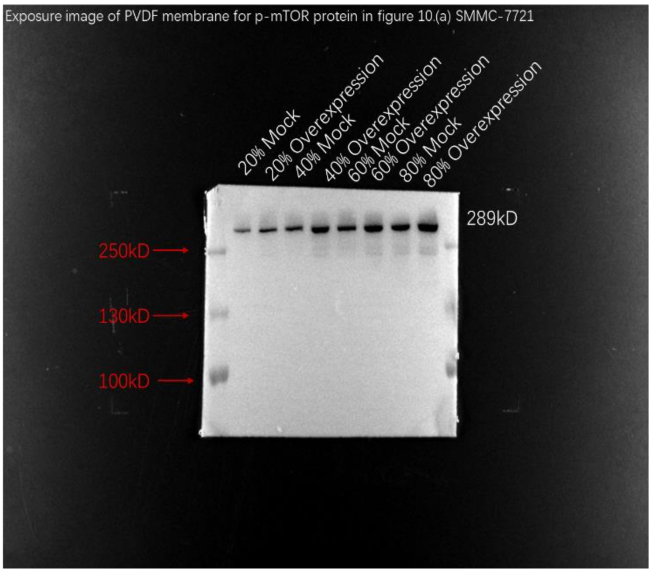

Figure 10.(c)   SMMC-7721, p-mTOR, exposure picture 2

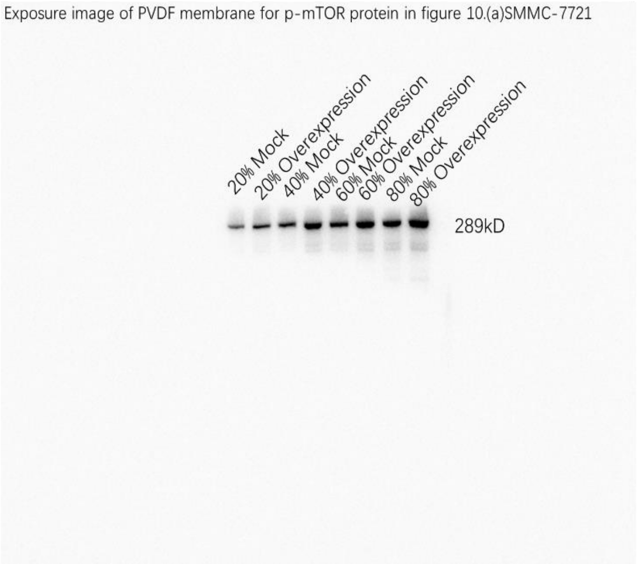

Figure 10.(c) SMMC-7721, PI3K, original figure

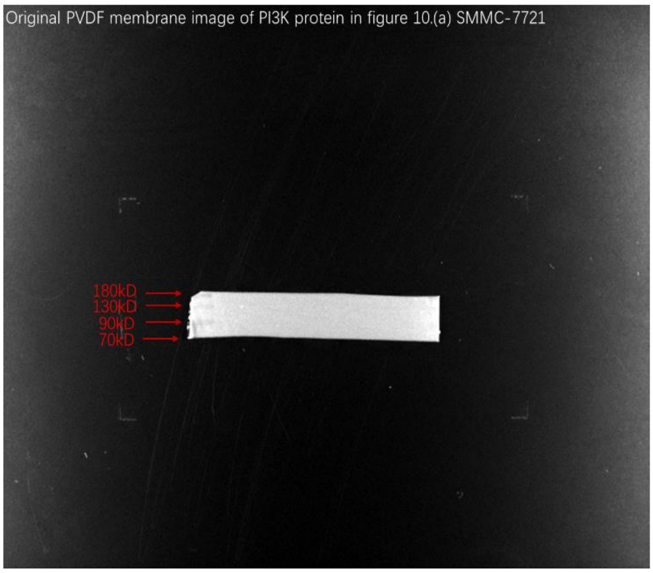

Figure 10.(c) SMMC-7721, PI3K, exposure picture 1

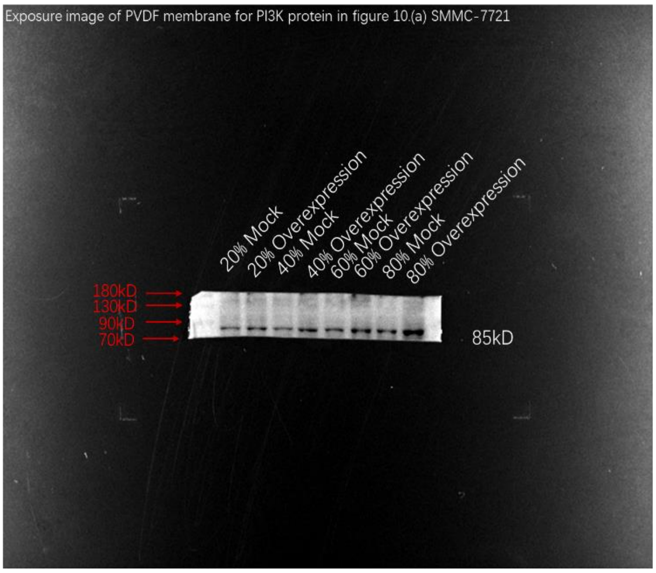

Figure 10.(c) SMMC-7721, PI3K, exposure picture 2

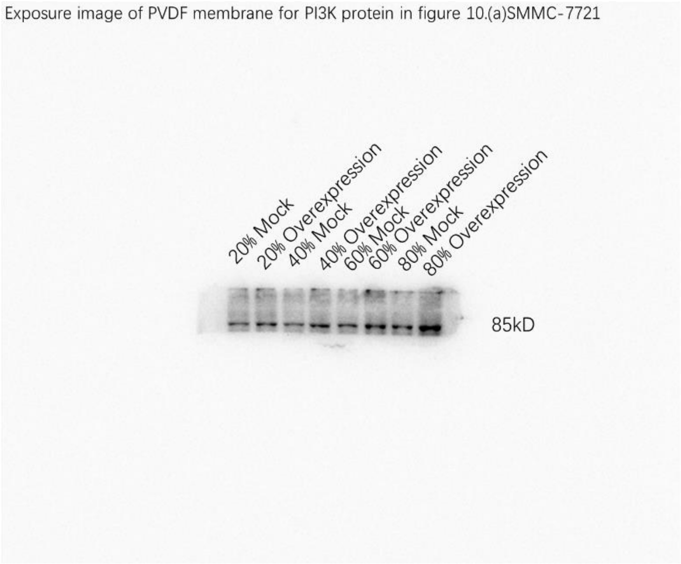

Figure 10.(c) SMMC-7721, p-PI3K, original figure

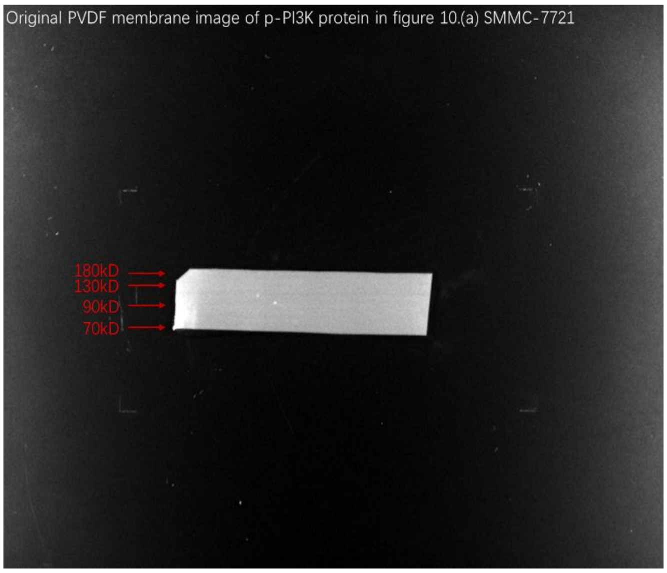

Figure 10.(c) SMMC-7721, p-PI3K, exposure picture 1

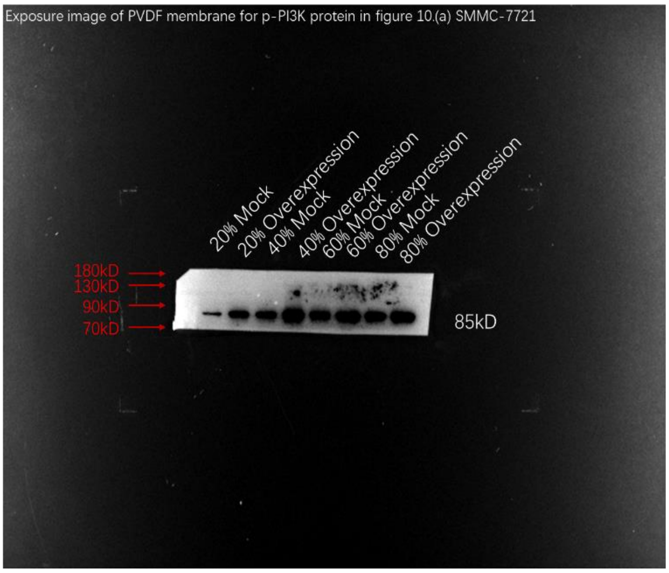

Figure 10.(c) SMMC-7721, p-PI3K, exposure picture 2

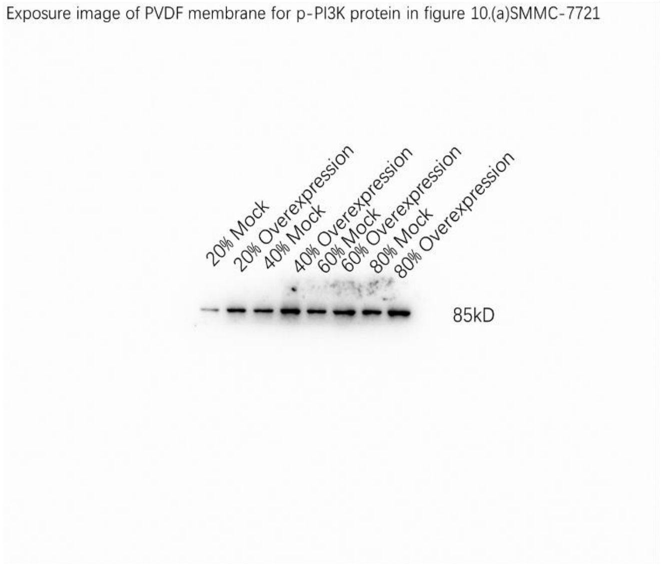

Supplement: S2 File — (ZIP) [file pone.0334639.s002.zip › Western Blot/Figure 10.pdf]
