# Supplementary figures and images for: CXCL3 promotes liver cancer progression by modulating the tumor microenvironment via the PI3K/AKT/mTOR pathway (part 1 of 3)
Source: PLoS One. 2025 Nov 19;20(11):e0334639. doi: 10.1371/journal.pone.0334639 (PMC12629499; doi:10.1371/journal.pone.0334639)

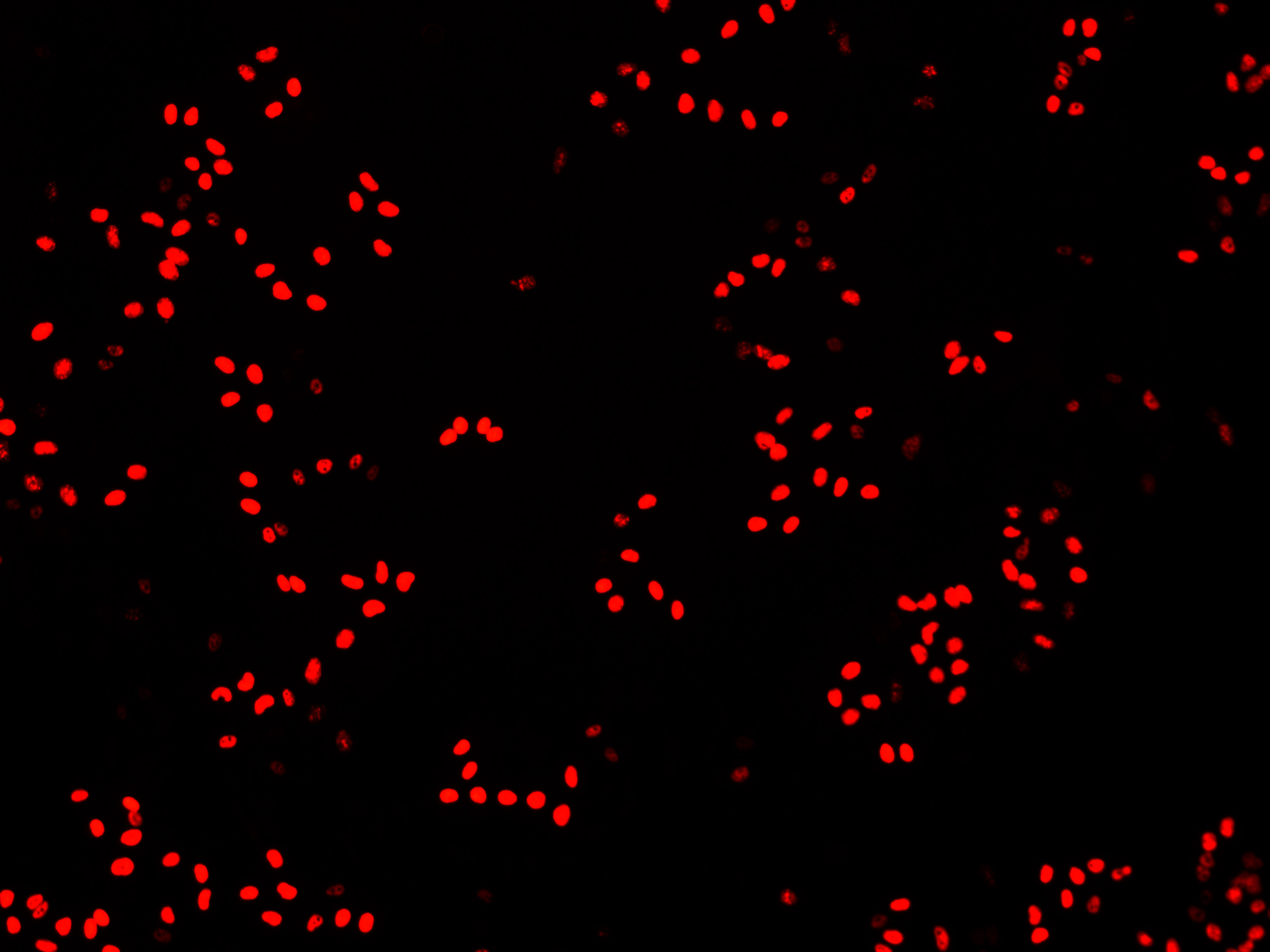

Supplement: S5 File — (ZIP) [file pone.0334639.s005.zip › S 10. File. Original FIgures. Fig.3/3b Bel-7402/0ngml-E.jpg]

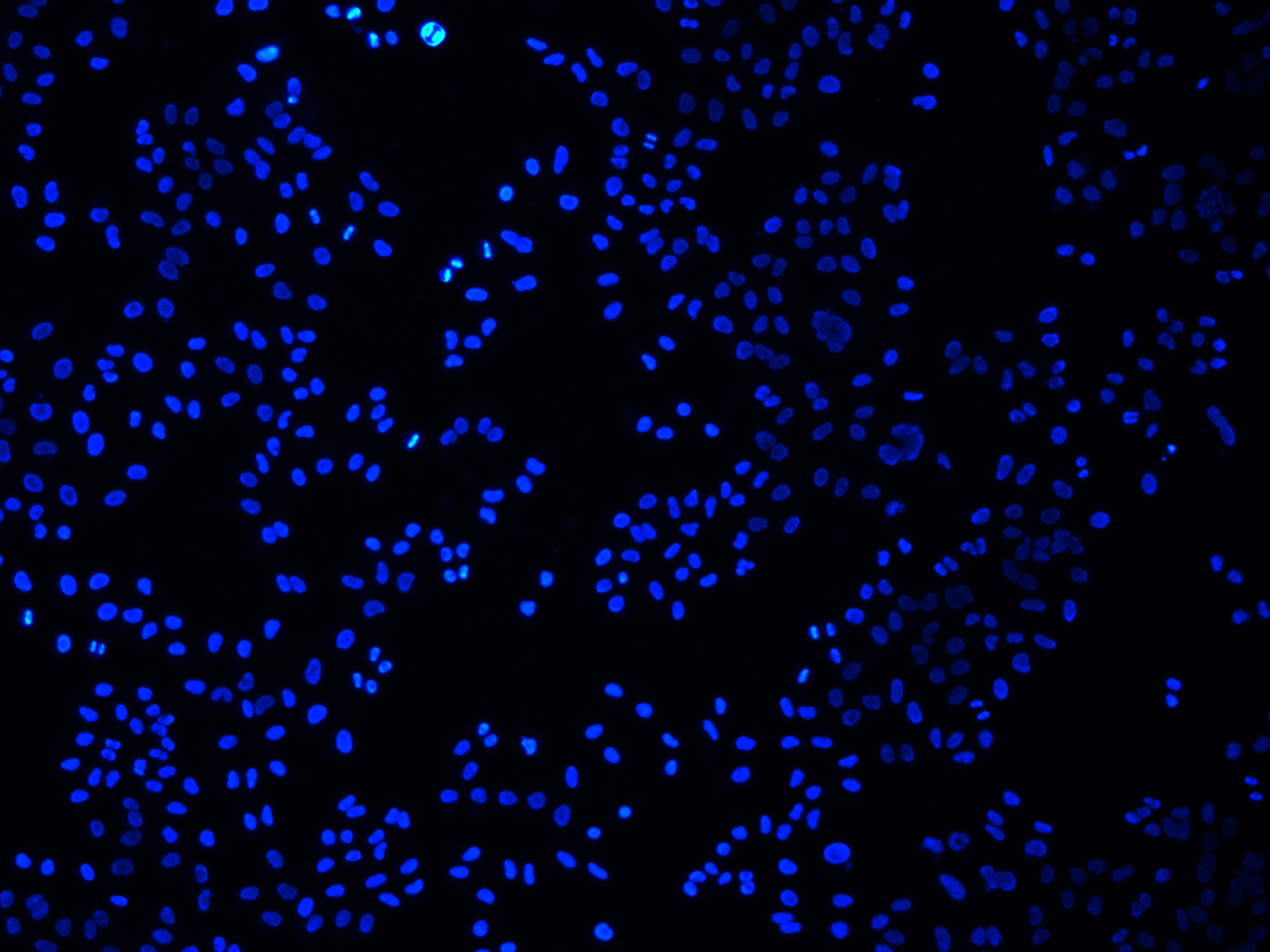

Supplement: S5 File — (ZIP) [file pone.0334639.s005.zip › S 10. File. Original FIgures. Fig.3/3b Bel-7402/0ngml-H.jpg]

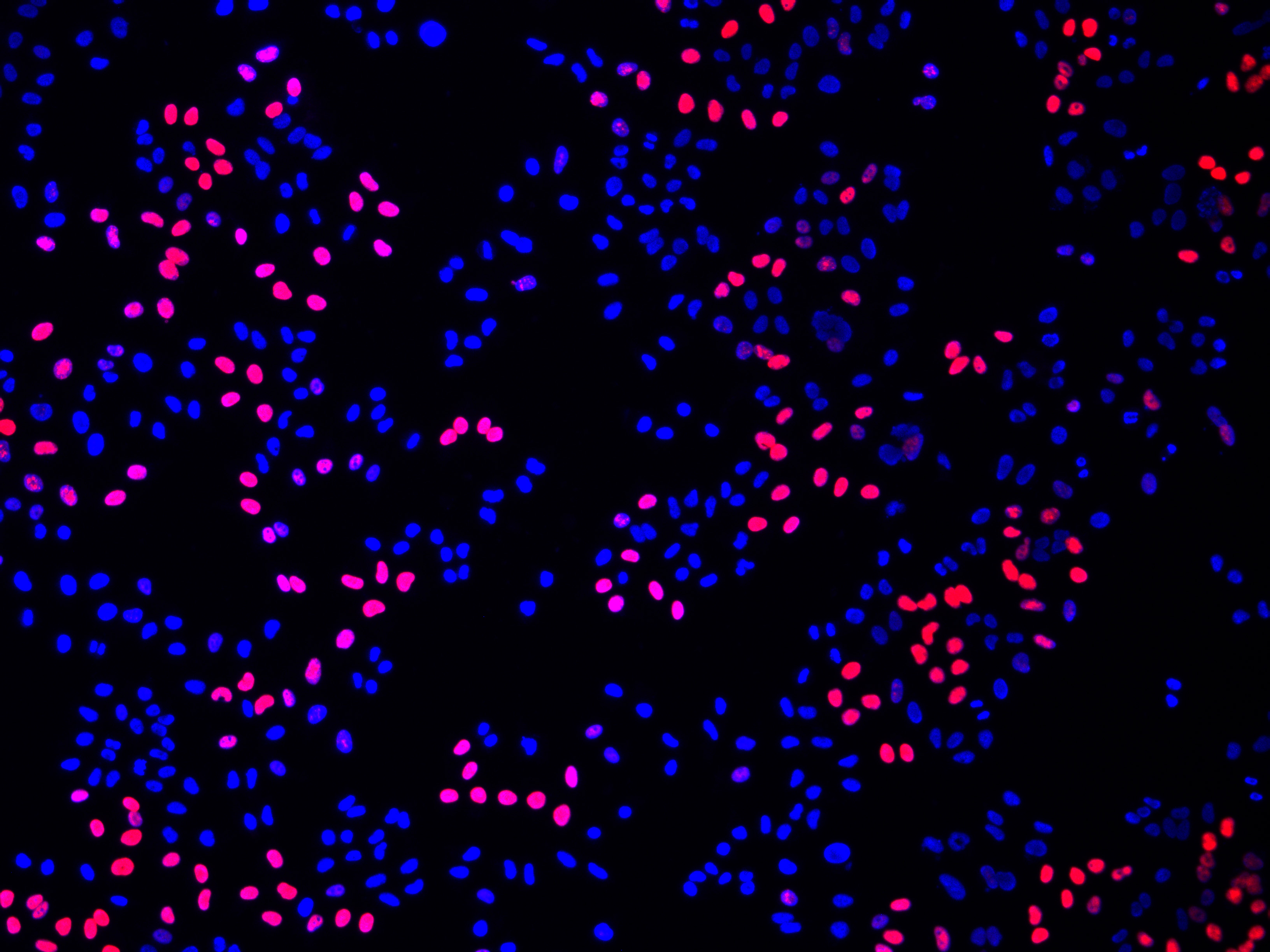

Supplement: S5 File — (ZIP) [file pone.0334639.s005.zip › S 10. File. Original FIgures. Fig.3/3b Bel-7402/0ngml-M.jpg]

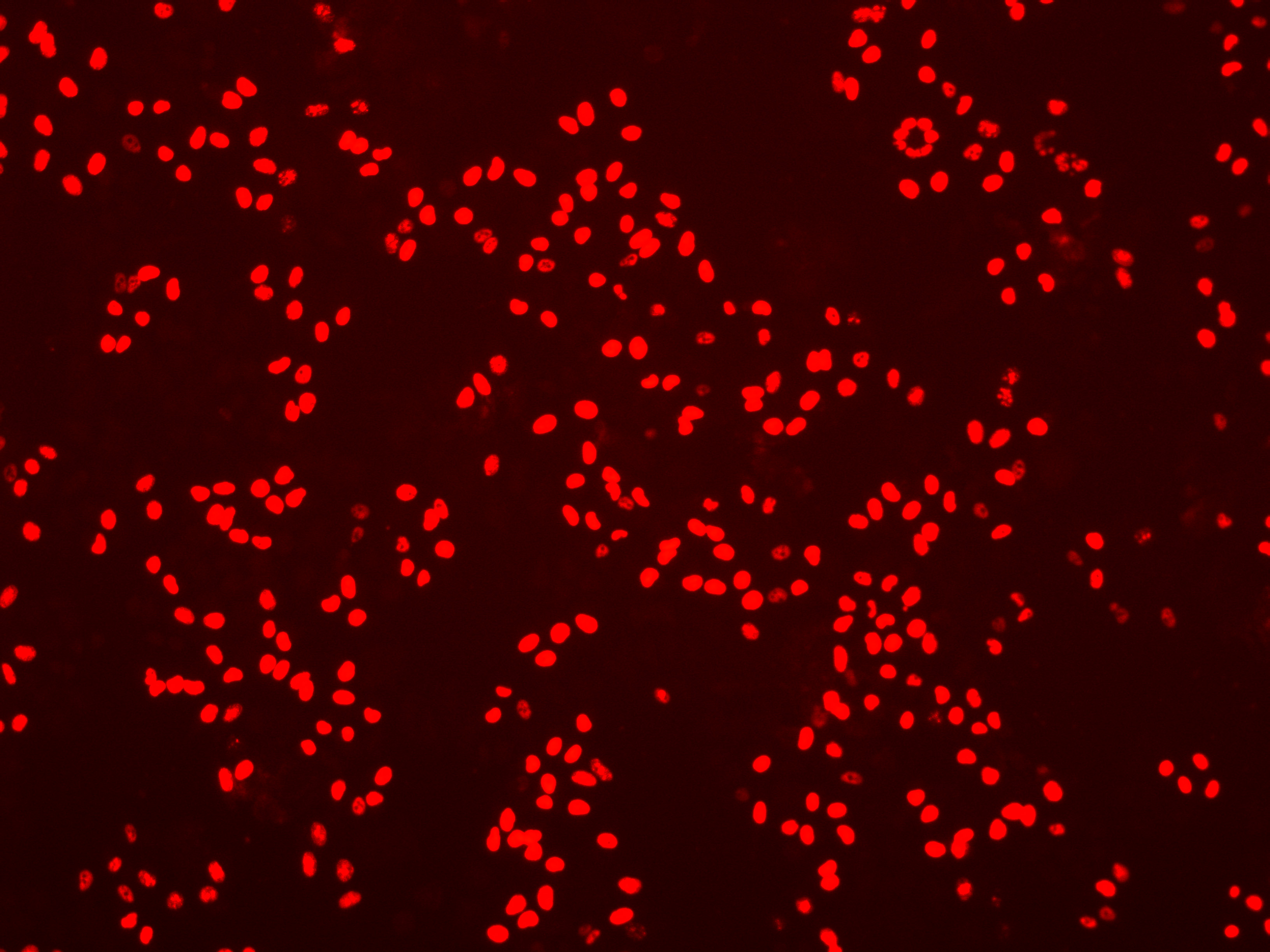

Supplement: S5 File — (ZIP) [file pone.0334639.s005.zip › S 10. File. Original FIgures. Fig.3/3b Bel-7402/10ngml-E.jpg]

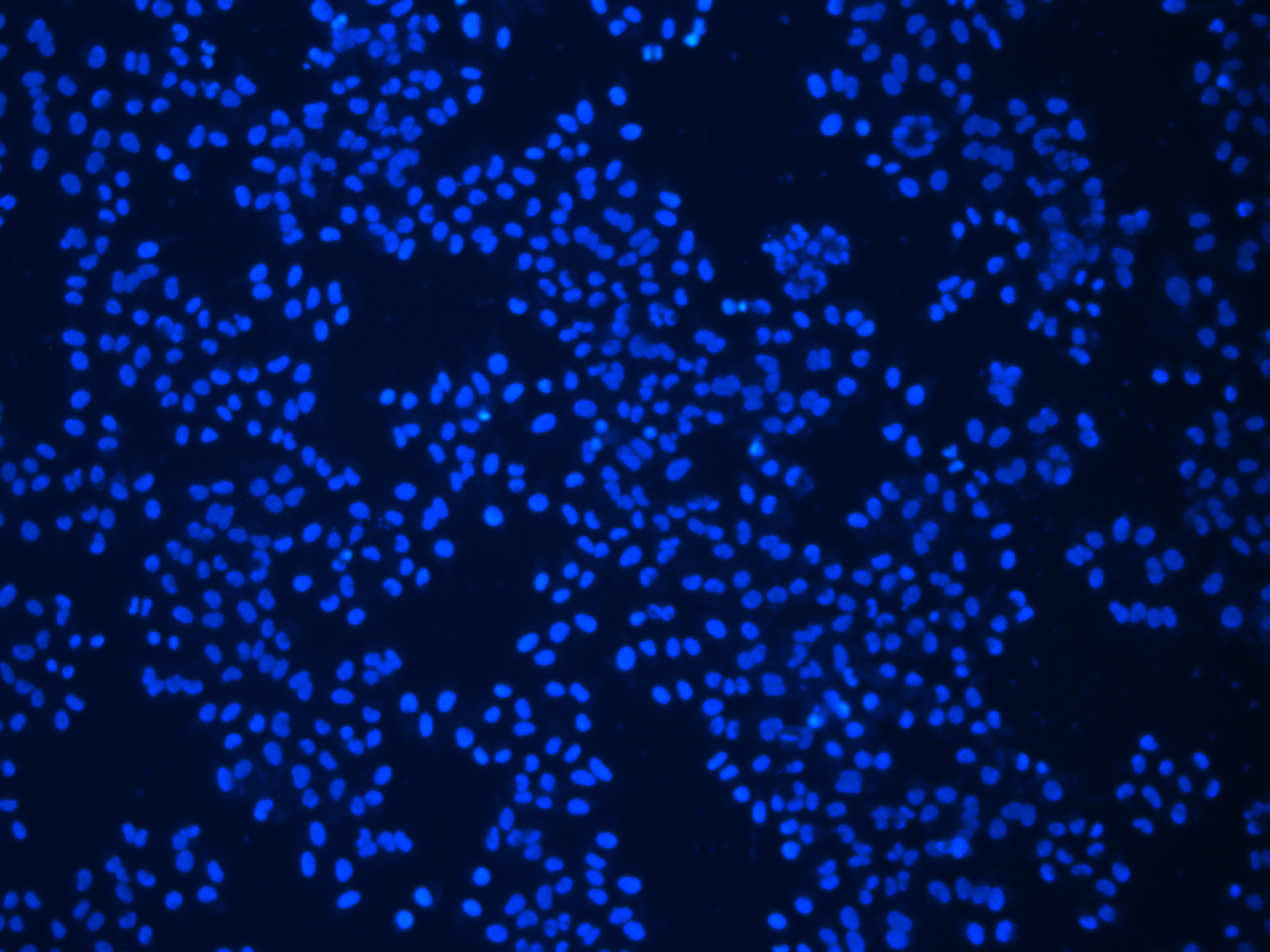

Supplement: S5 File — (ZIP) [file pone.0334639.s005.zip › S 10. File. Original FIgures. Fig.3/3b Bel-7402/10ngml-H.jpg]

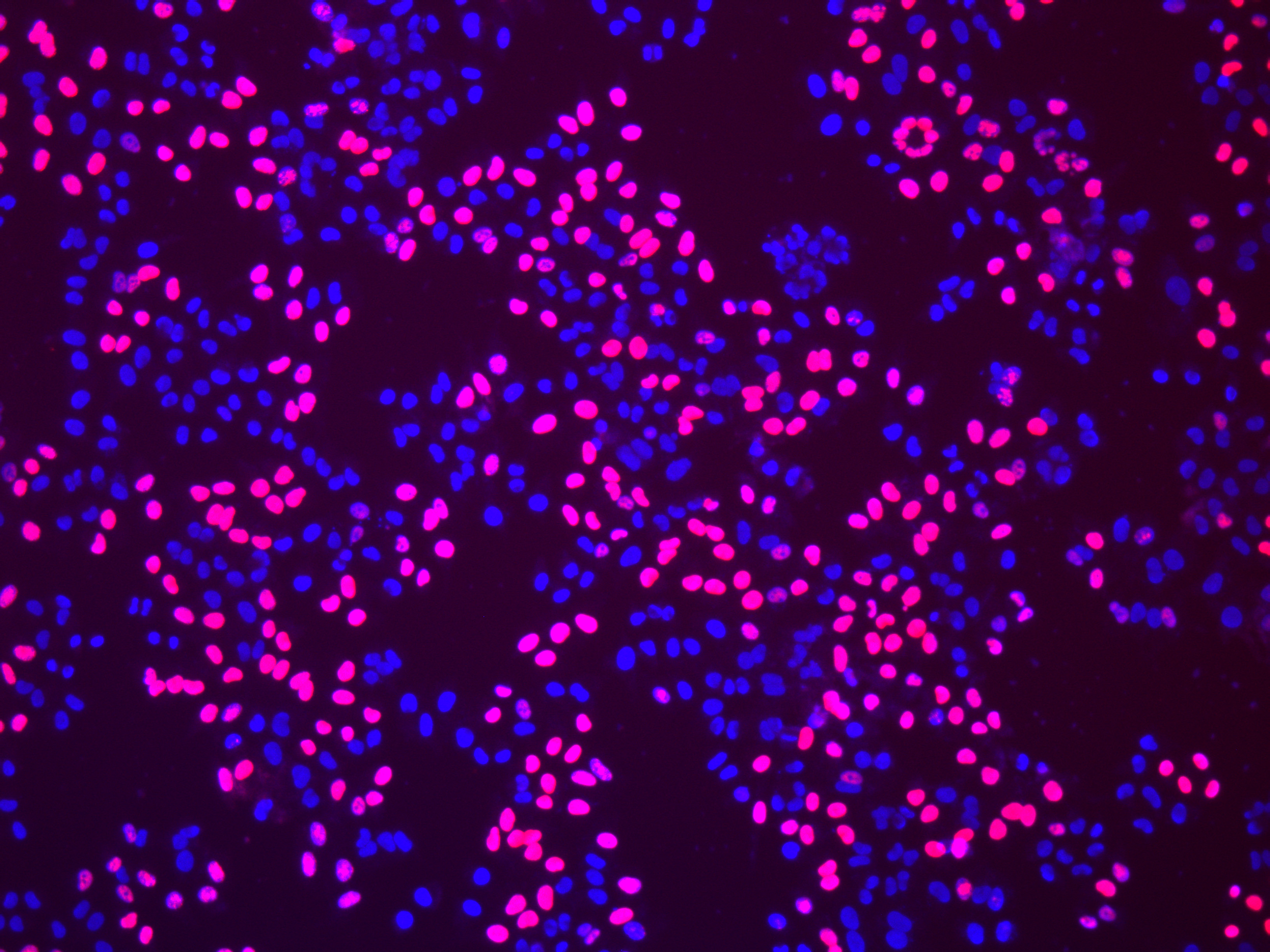

Supplement: S5 File — (ZIP) [file pone.0334639.s005.zip › S 10. File. Original FIgures. Fig.3/3b Bel-7402/10ngml-M.jpg]

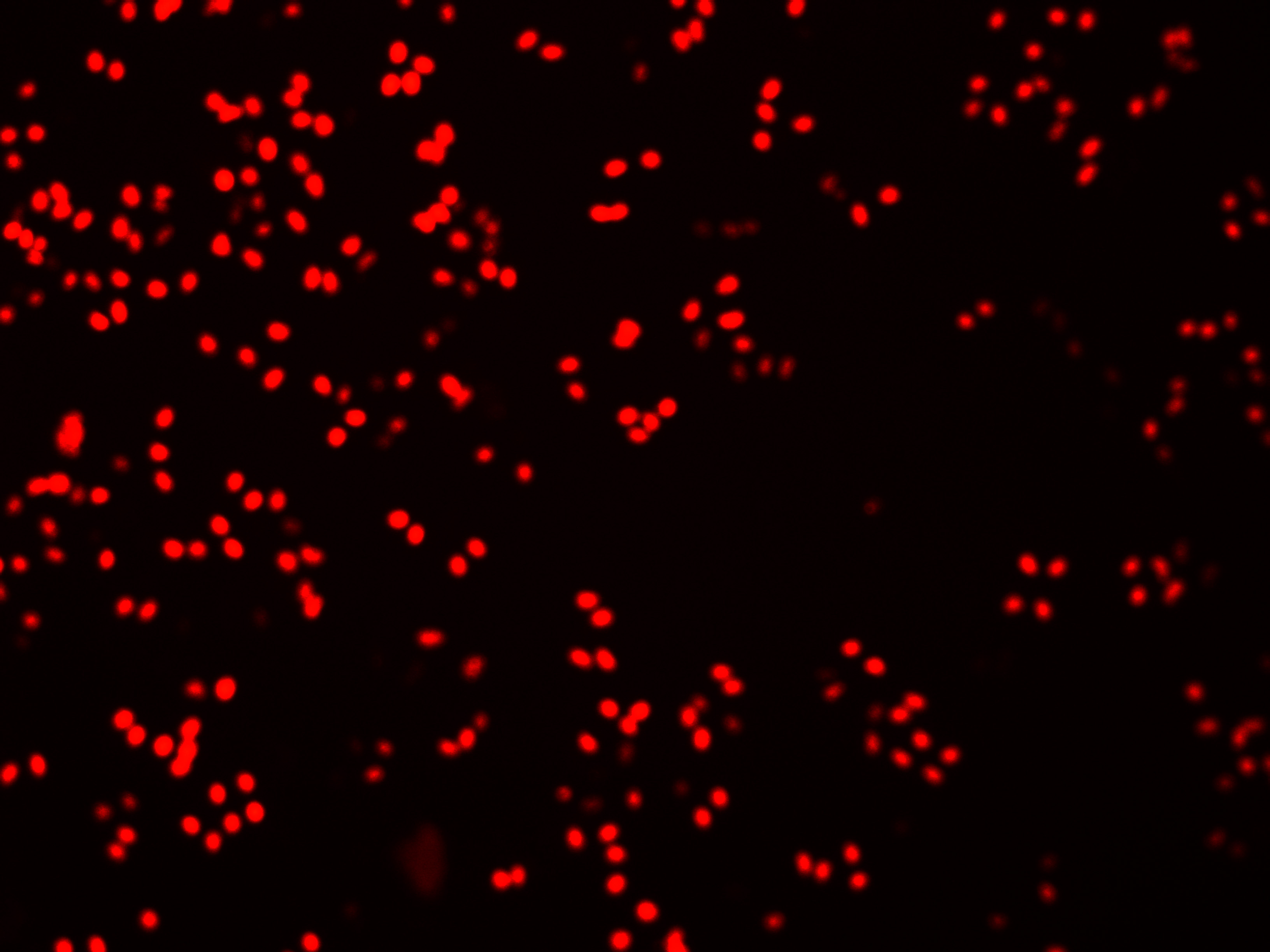

Supplement: S5 File — (ZIP) [file pone.0334639.s005.zip › S 10. File. Original FIgures. Fig.3/3b Bel-7402/5ngml-E.jpg]

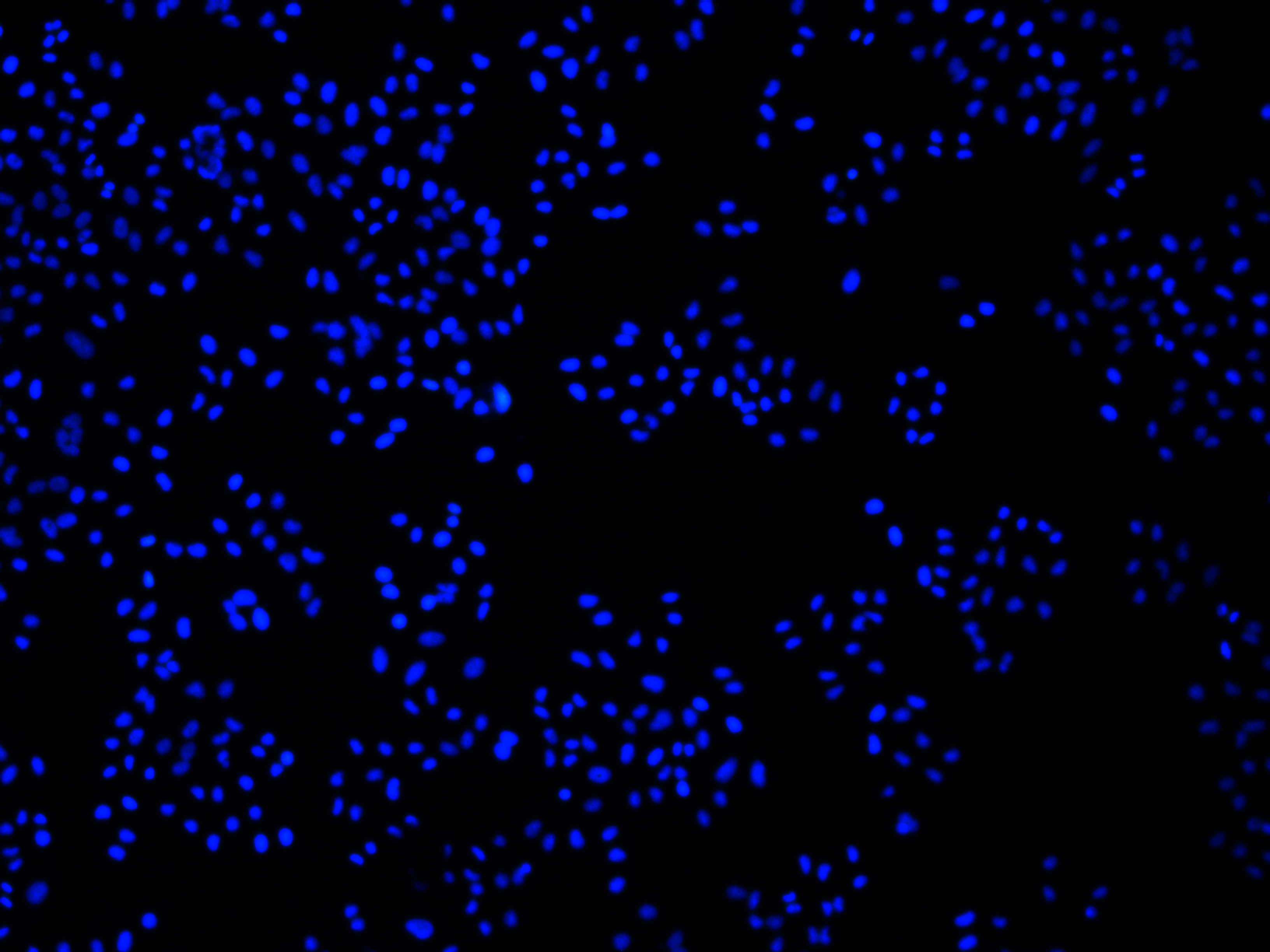

Supplement: S5 File — (ZIP) [file pone.0334639.s005.zip › S 10. File. Original FIgures. Fig.3/3b Bel-7402/5ngml-H.jpg]

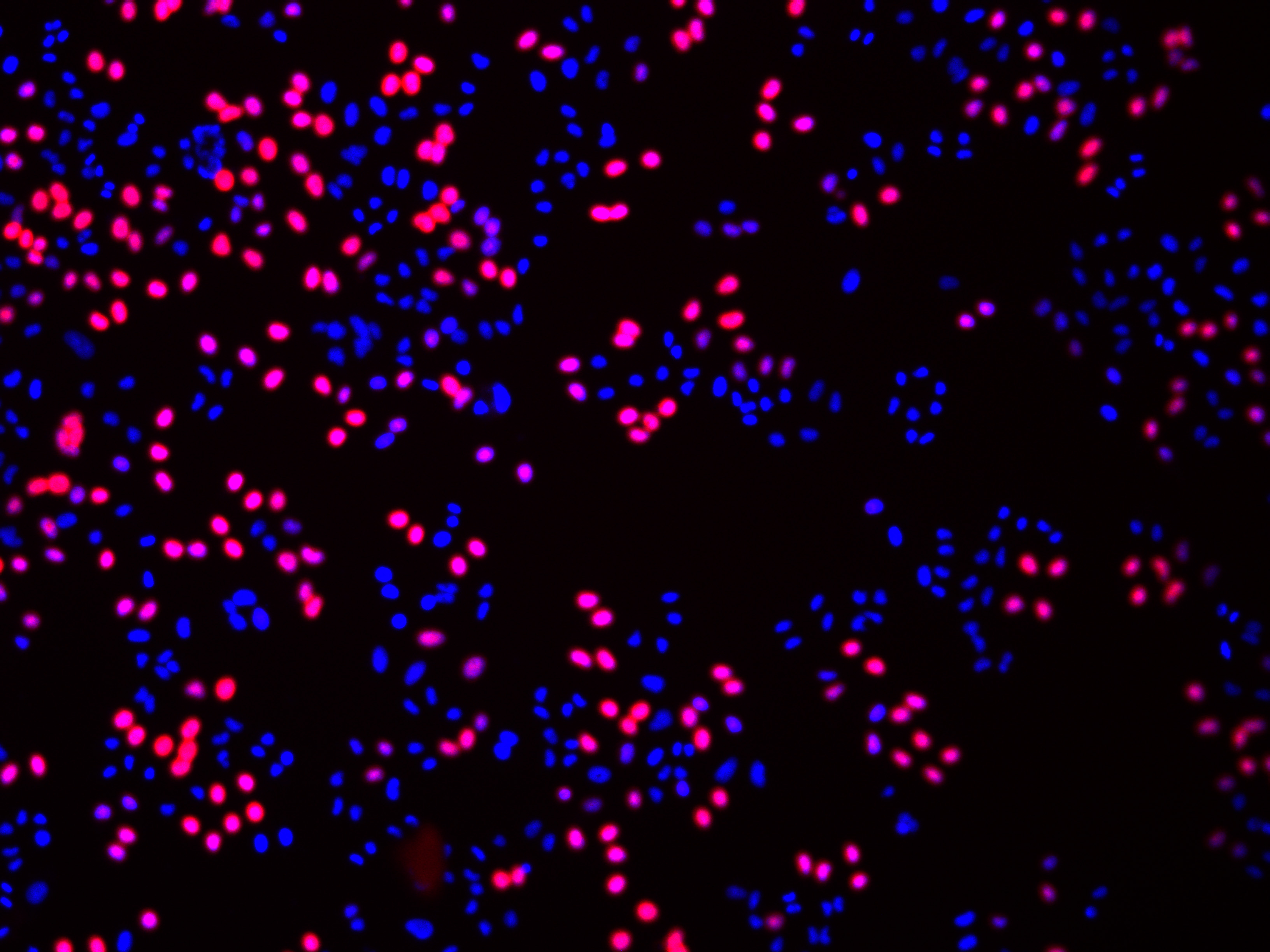

Supplement: S5 File — (ZIP) [file pone.0334639.s005.zip › S 10. File. Original FIgures. Fig.3/3b Bel-7402/5ngml-M.jpg]

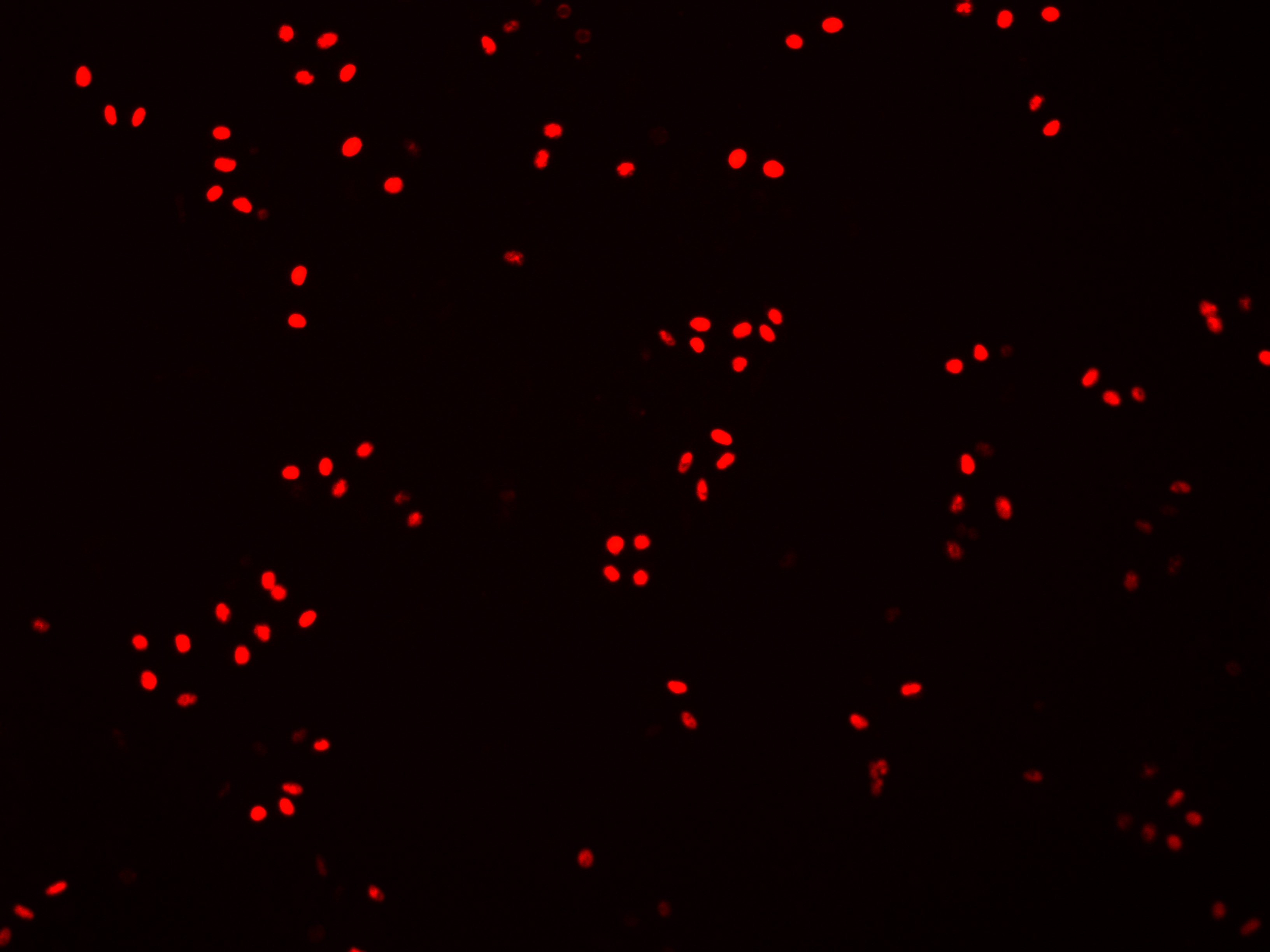

Supplement: S5 File — (ZIP) [file pone.0334639.s005.zip › S 10. File. Original FIgures. Fig.3/3c HepG2/0ngml-E.jpg]

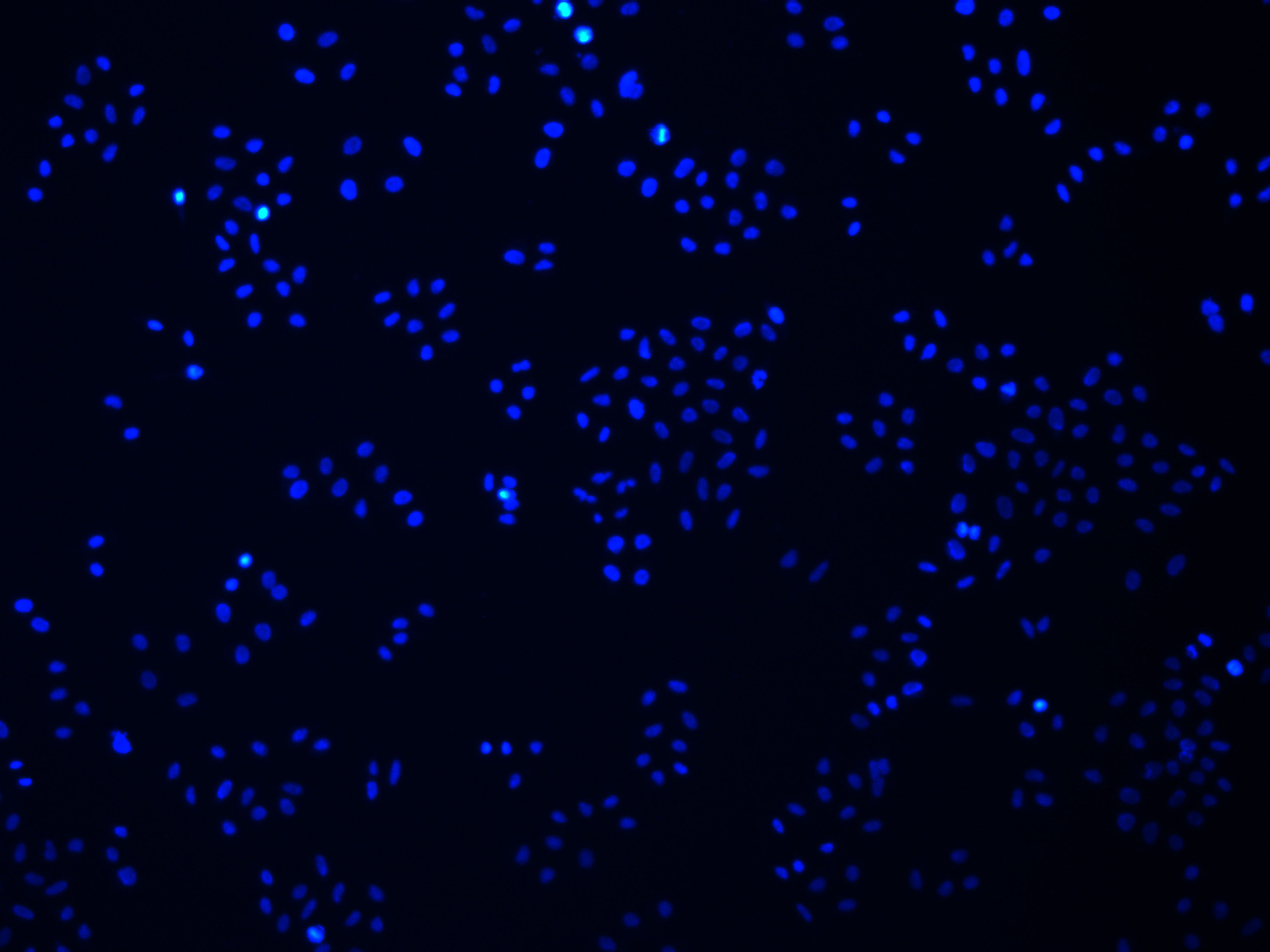

Supplement: S5 File — (ZIP) [file pone.0334639.s005.zip › S 10. File. Original FIgures. Fig.3/3c HepG2/0ngml-H.jpg]

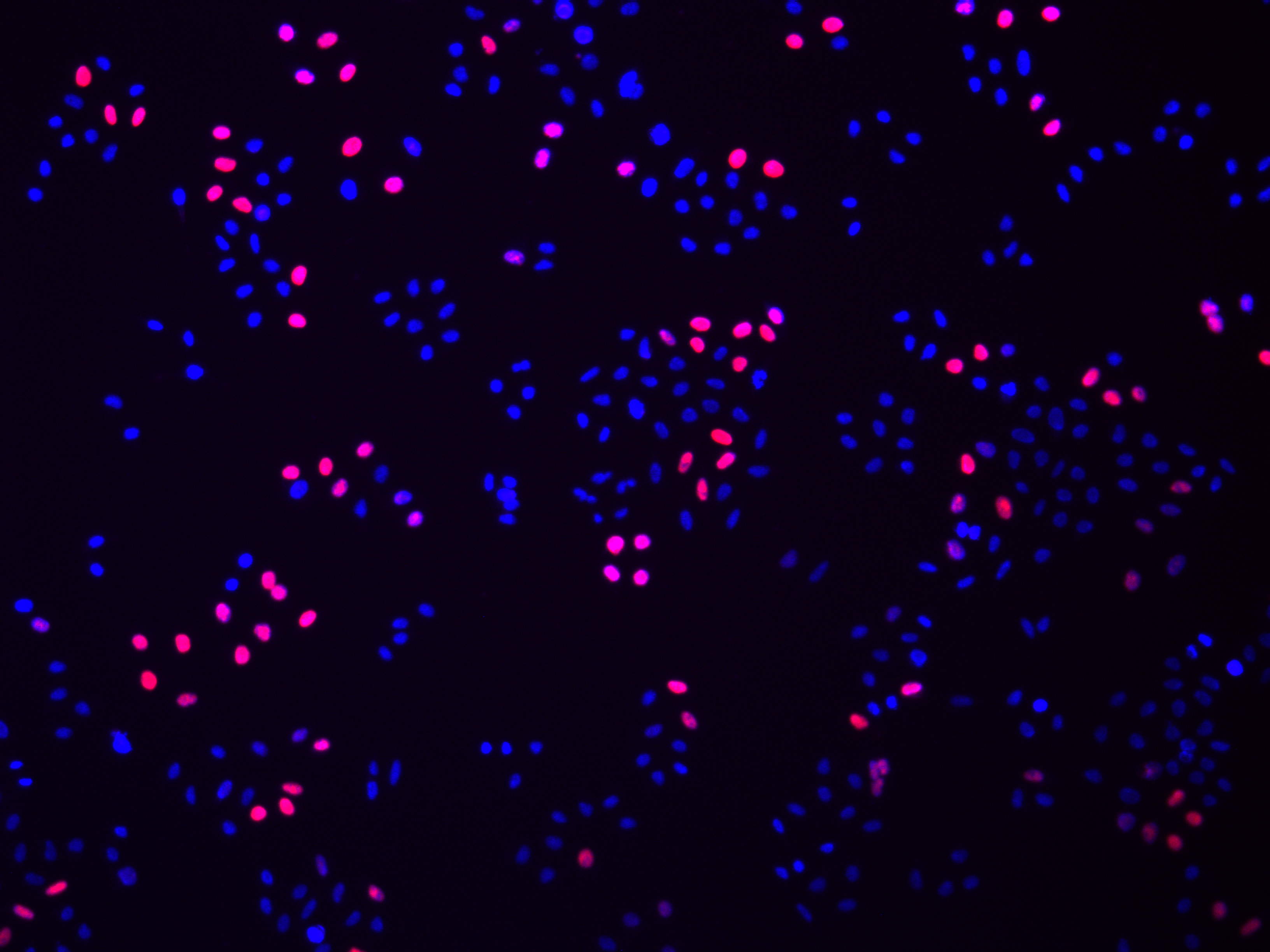

Supplement: S5 File — (ZIP) [file pone.0334639.s005.zip › S 10. File. Original FIgures. Fig.3/3c HepG2/0ngml-M.jpg]

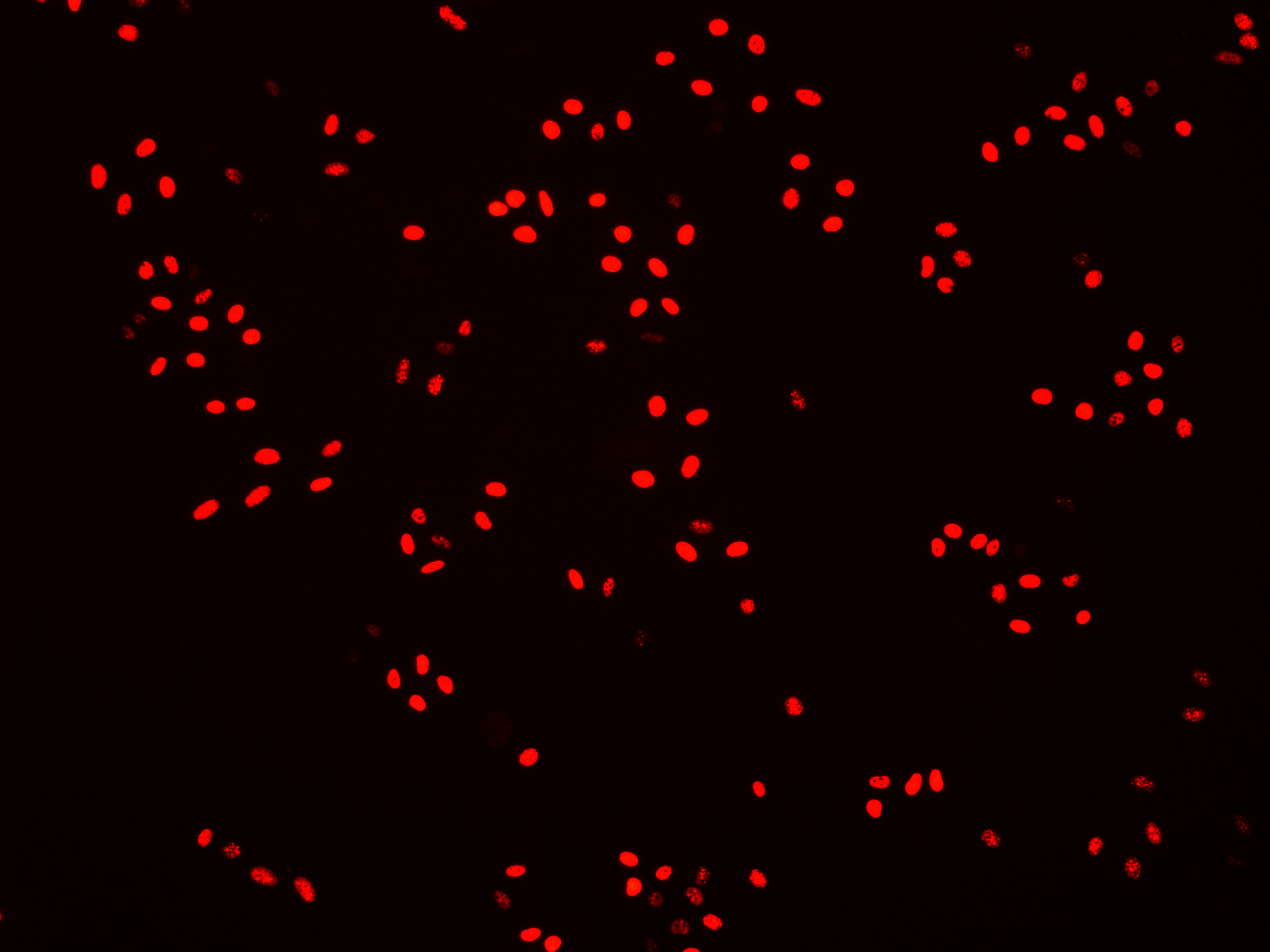

Supplement: S5 File — (ZIP) [file pone.0334639.s005.zip › S 10. File. Original FIgures. Fig.3/3c HepG2/10ngml-E.jpg]

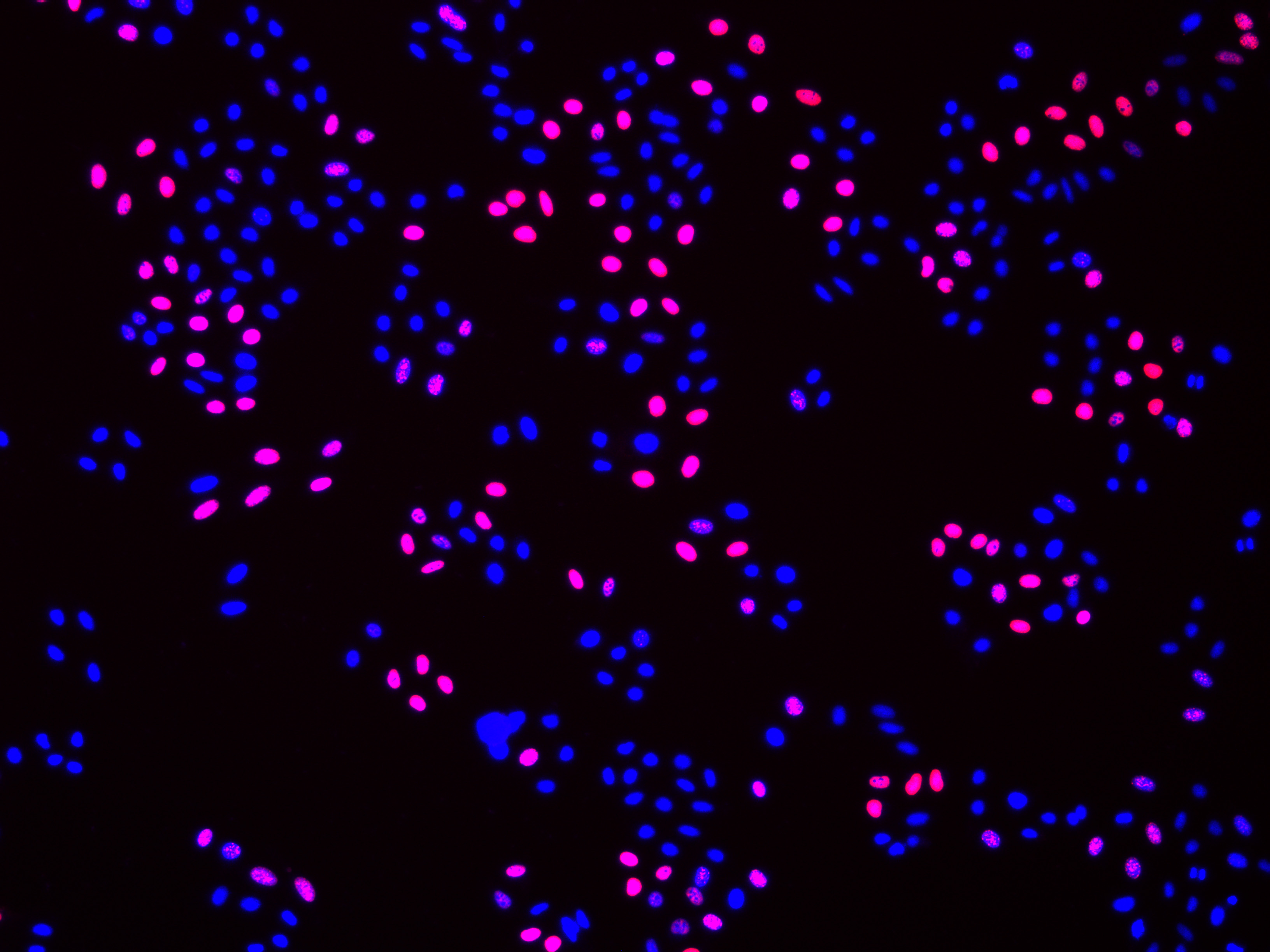

Supplement: S5 File — (ZIP) [file pone.0334639.s005.zip › S 10. File. Original FIgures. Fig.3/3c HepG2/10ngml-M.jpg]

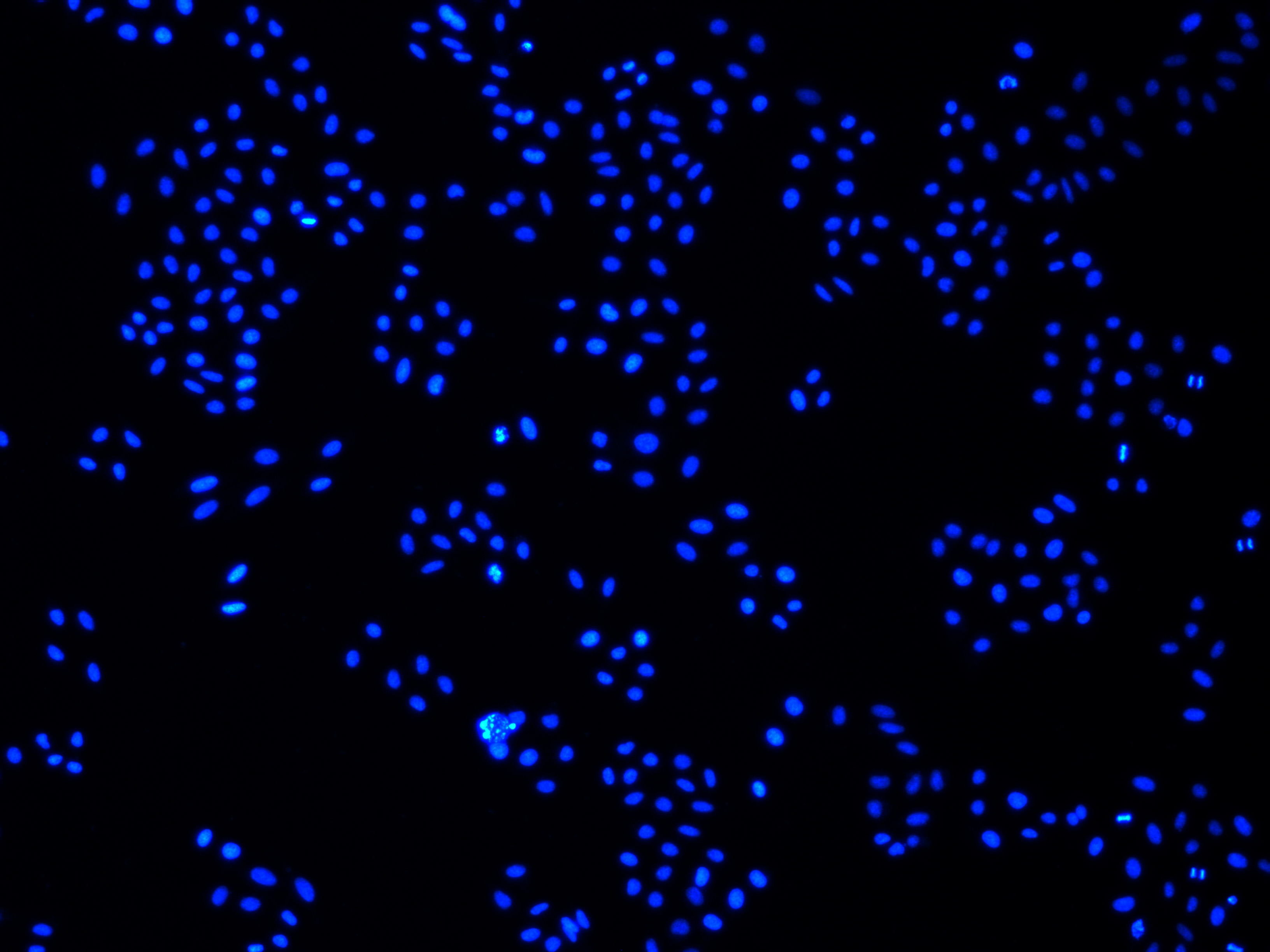

Supplement: S5 File — (ZIP) [file pone.0334639.s005.zip › S 10. File. Original FIgures. Fig.3/3c HepG2/10ngml.jpg]

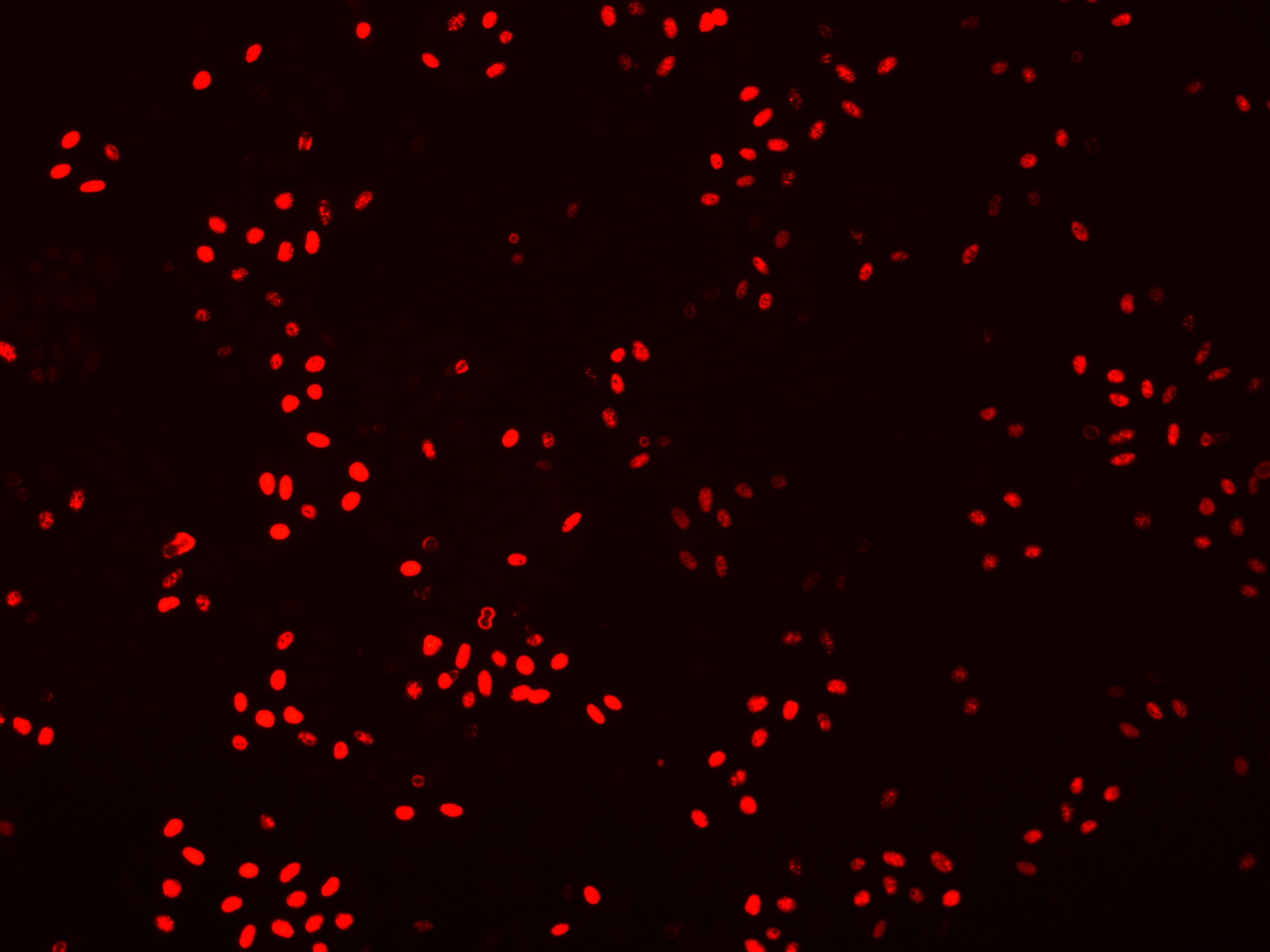

Supplement: S5 File — (ZIP) [file pone.0334639.s005.zip › S 10. File. Original FIgures. Fig.3/3c HepG2/5ngml-E.jpg]

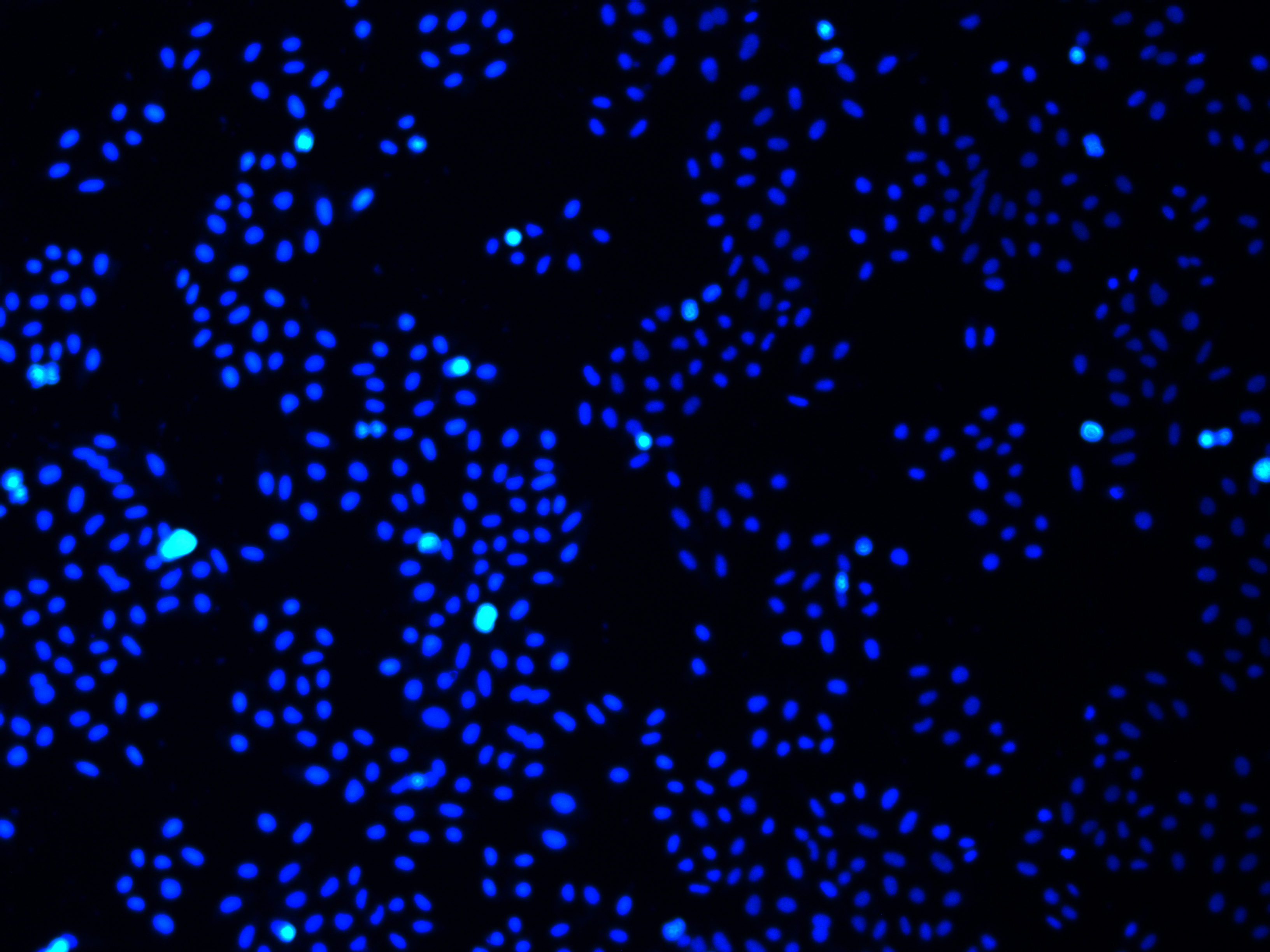

Supplement: S5 File — (ZIP) [file pone.0334639.s005.zip › S 10. File. Original FIgures. Fig.3/3c HepG2/5ngml-H.jpg]

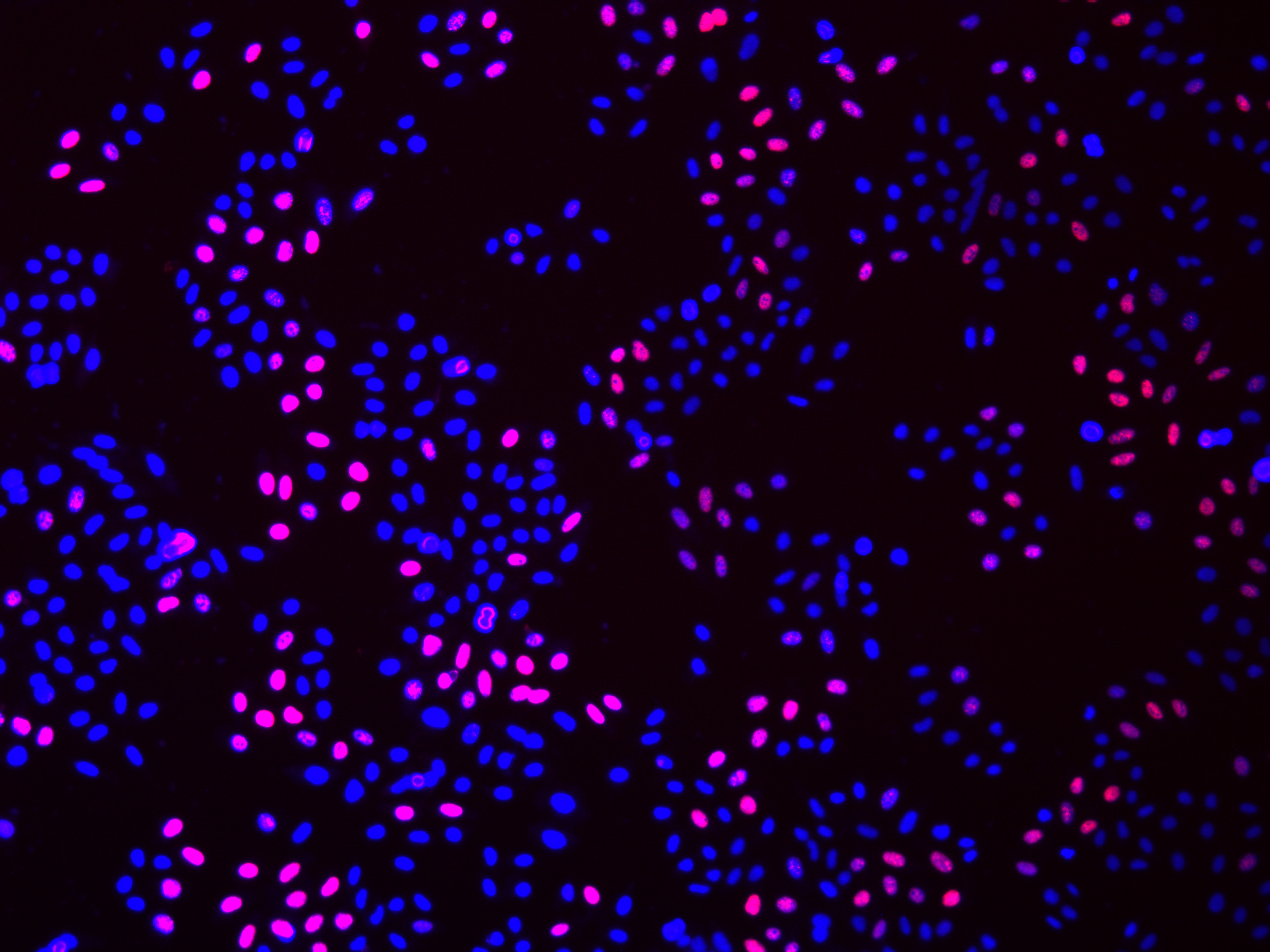

Supplement: S5 File — (ZIP) [file pone.0334639.s005.zip › S 10. File. Original FIgures. Fig.3/3c HepG2/5ngml-M.jpg]

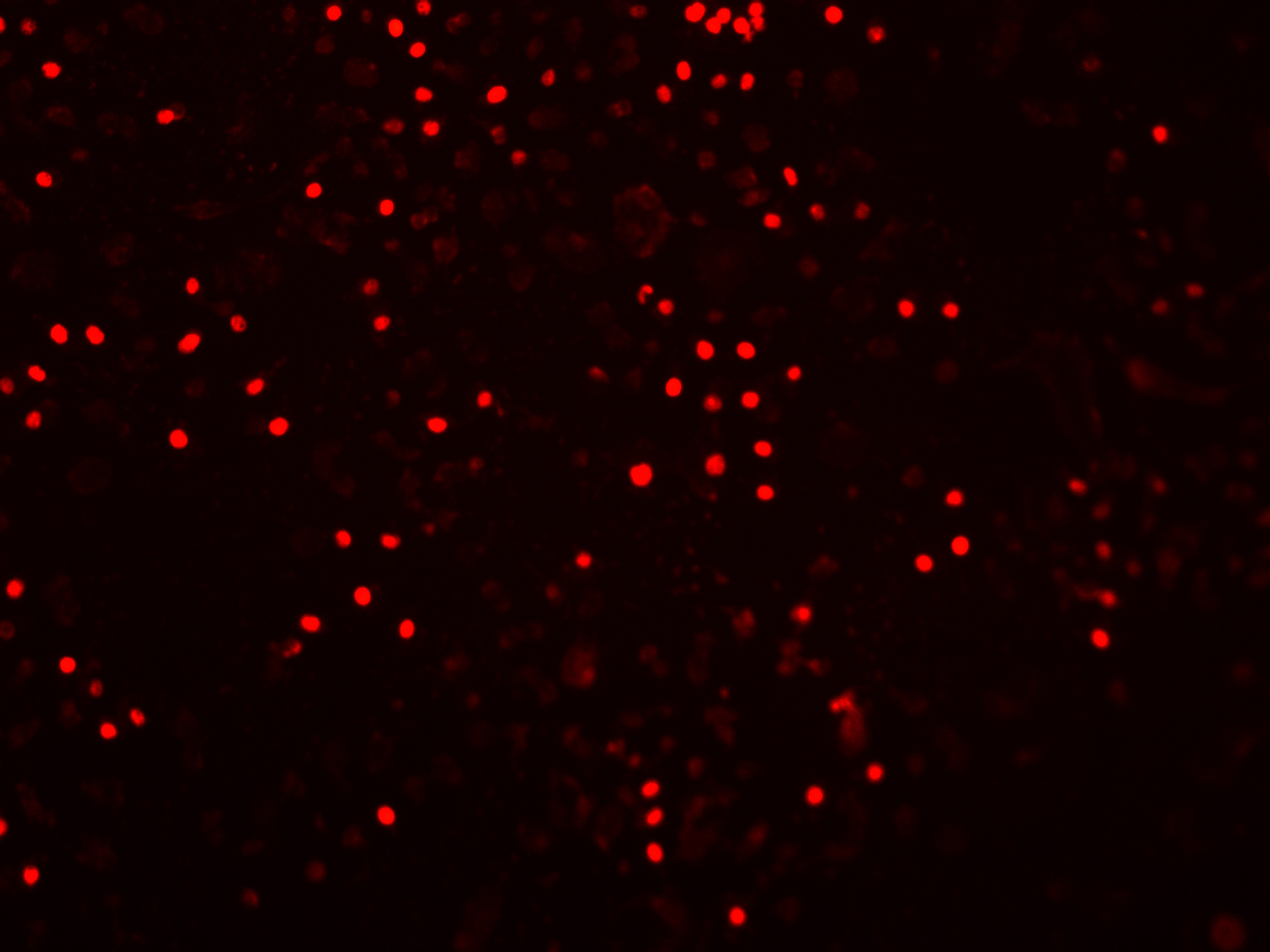

Supplement: S5 File — (ZIP) [file pone.0334639.s005.zip › S 10. File. Original FIgures. Fig.3/3d SMMC-7721/0ngml-E.jpg]

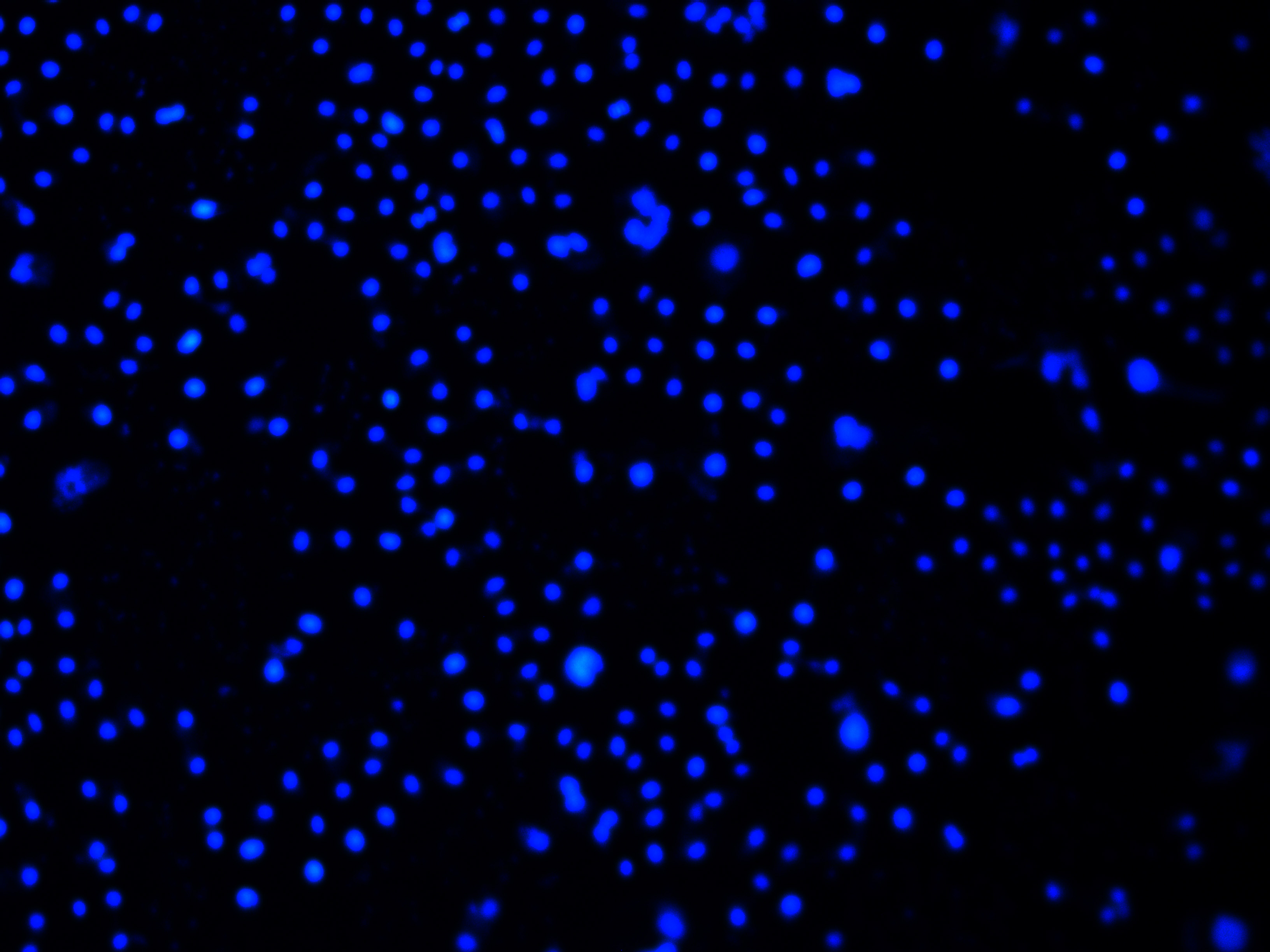

Supplement: S5 File — (ZIP) [file pone.0334639.s005.zip › S 10. File. Original FIgures. Fig.3/3d SMMC-7721/0ngml-H.jpg]

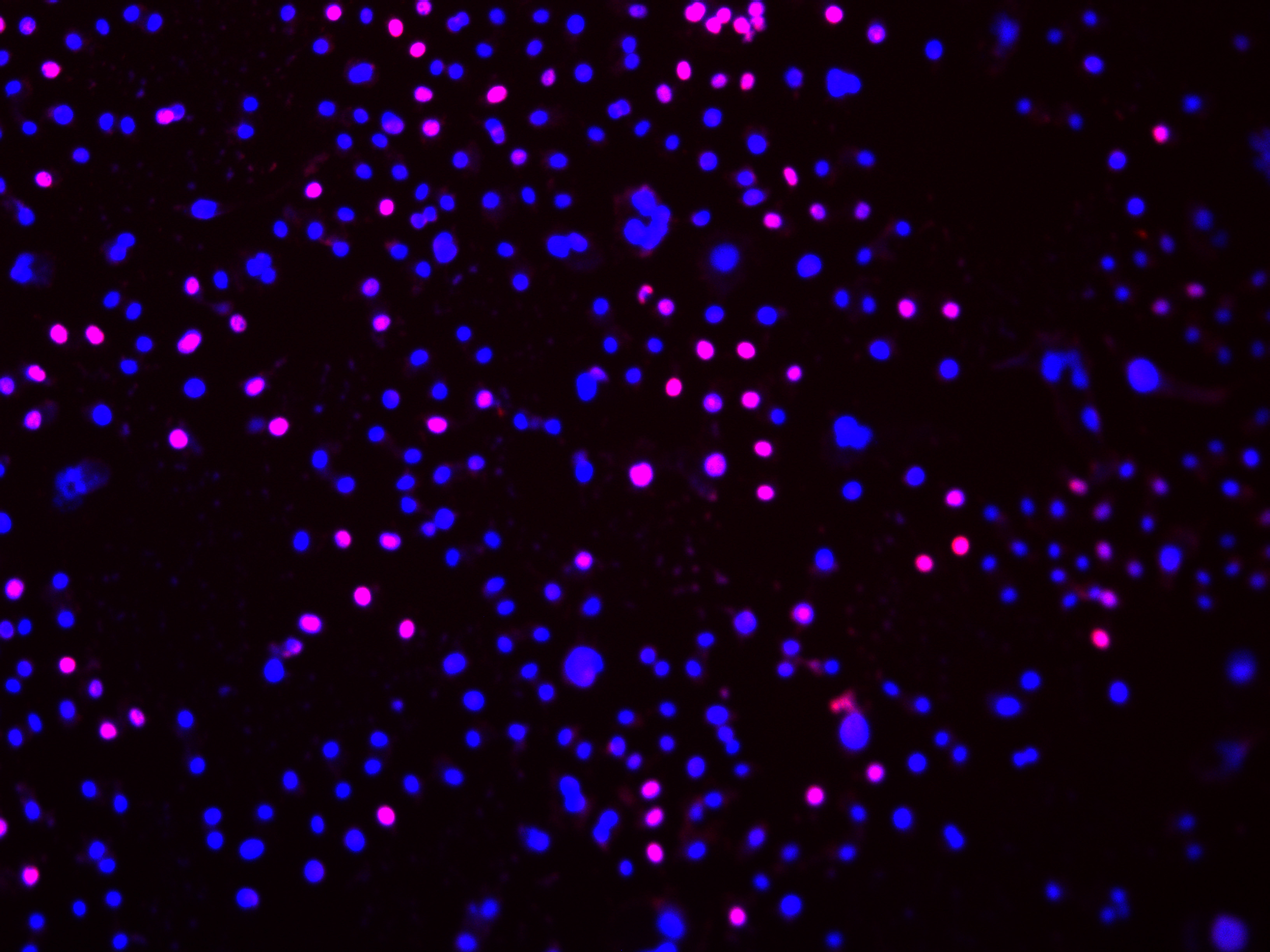

Supplement: S5 File — (ZIP) [file pone.0334639.s005.zip › S 10. File. Original FIgures. Fig.3/3d SMMC-7721/0ngml-M.jpg]

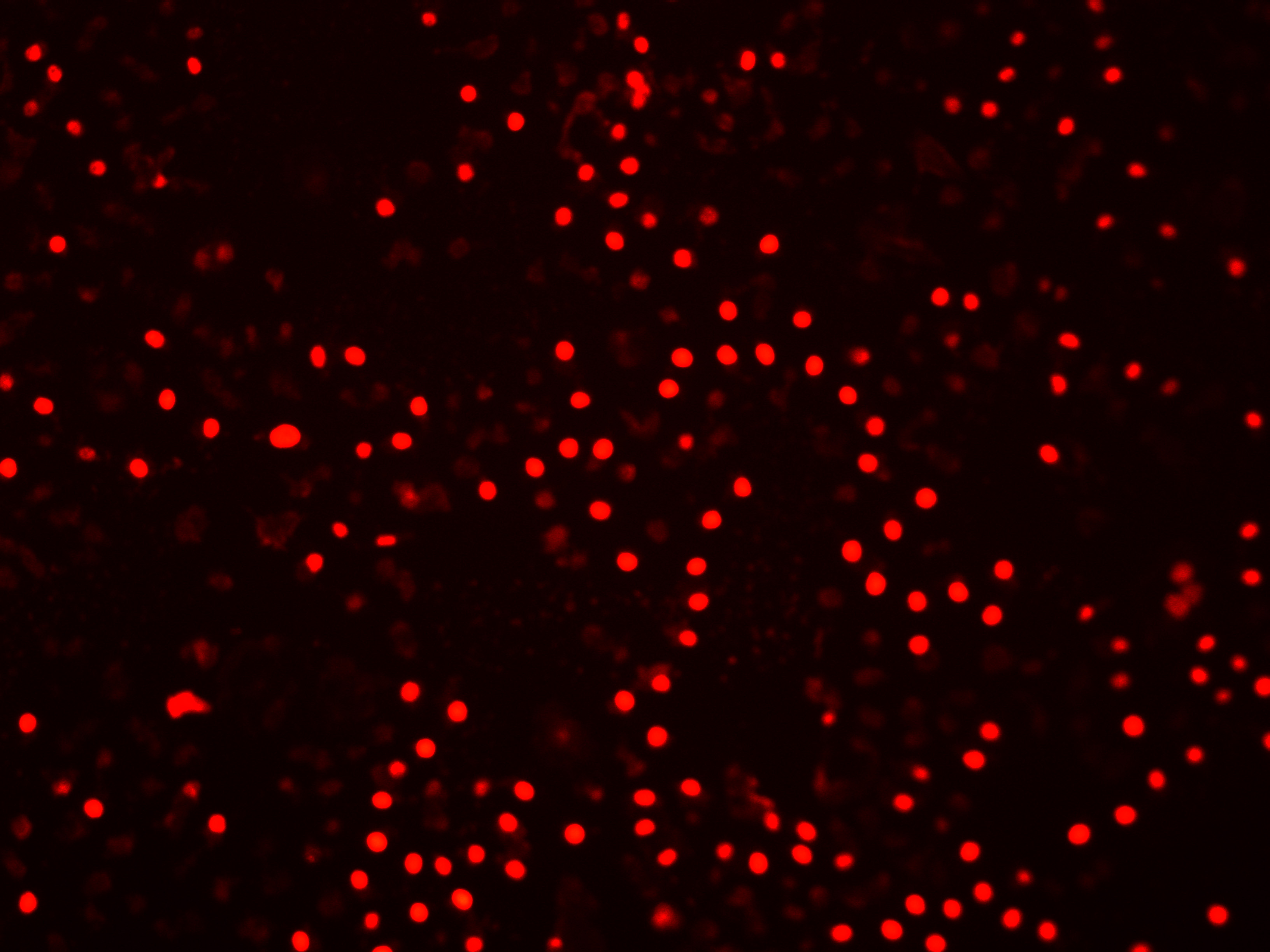

Supplement: S5 File — (ZIP) [file pone.0334639.s005.zip › S 10. File. Original FIgures. Fig.3/3d SMMC-7721/10ngml-E.jpg]

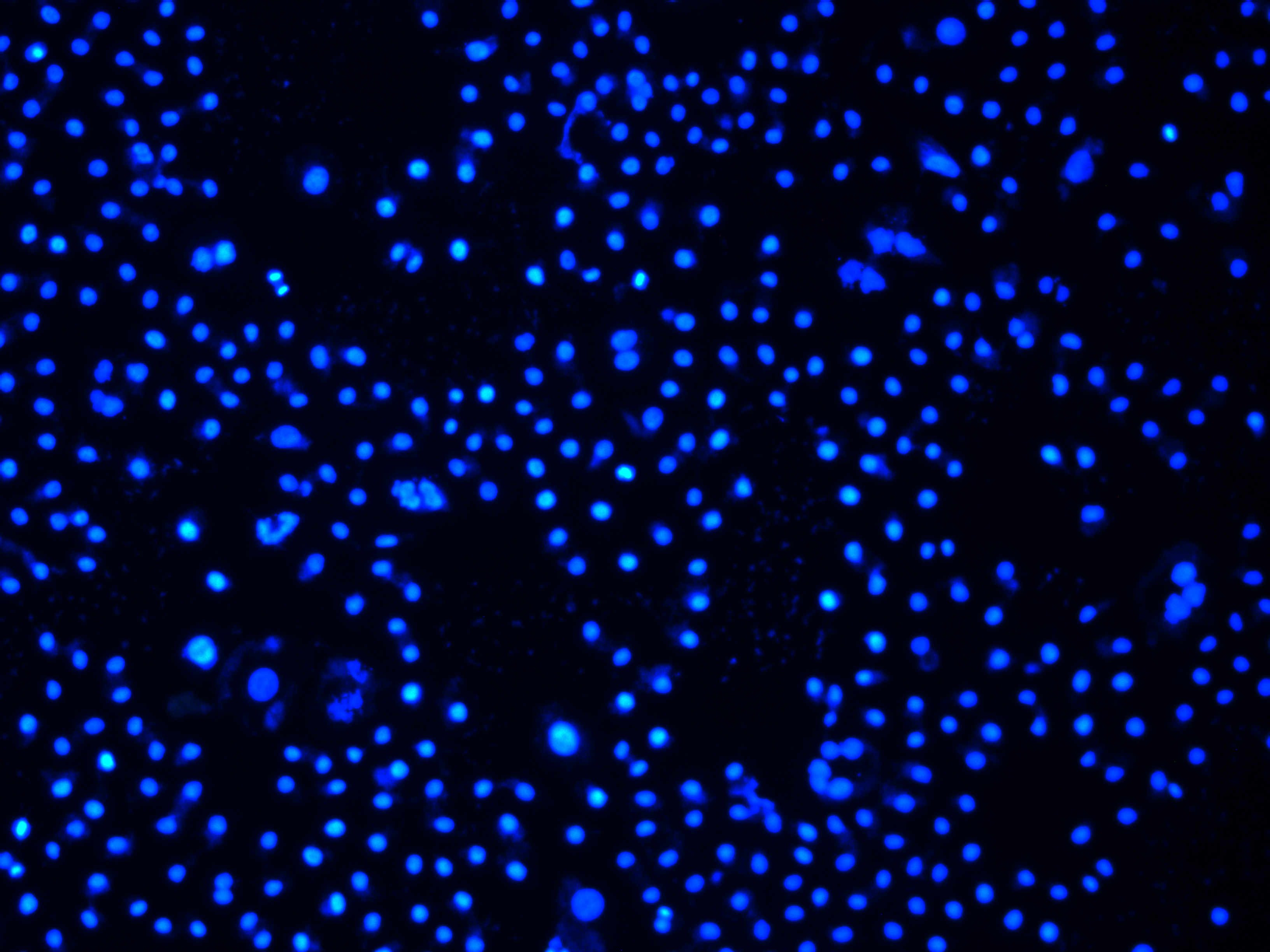

Supplement: S5 File — (ZIP) [file pone.0334639.s005.zip › S 10. File. Original FIgures. Fig.3/3d SMMC-7721/10ngml-H.jpg]

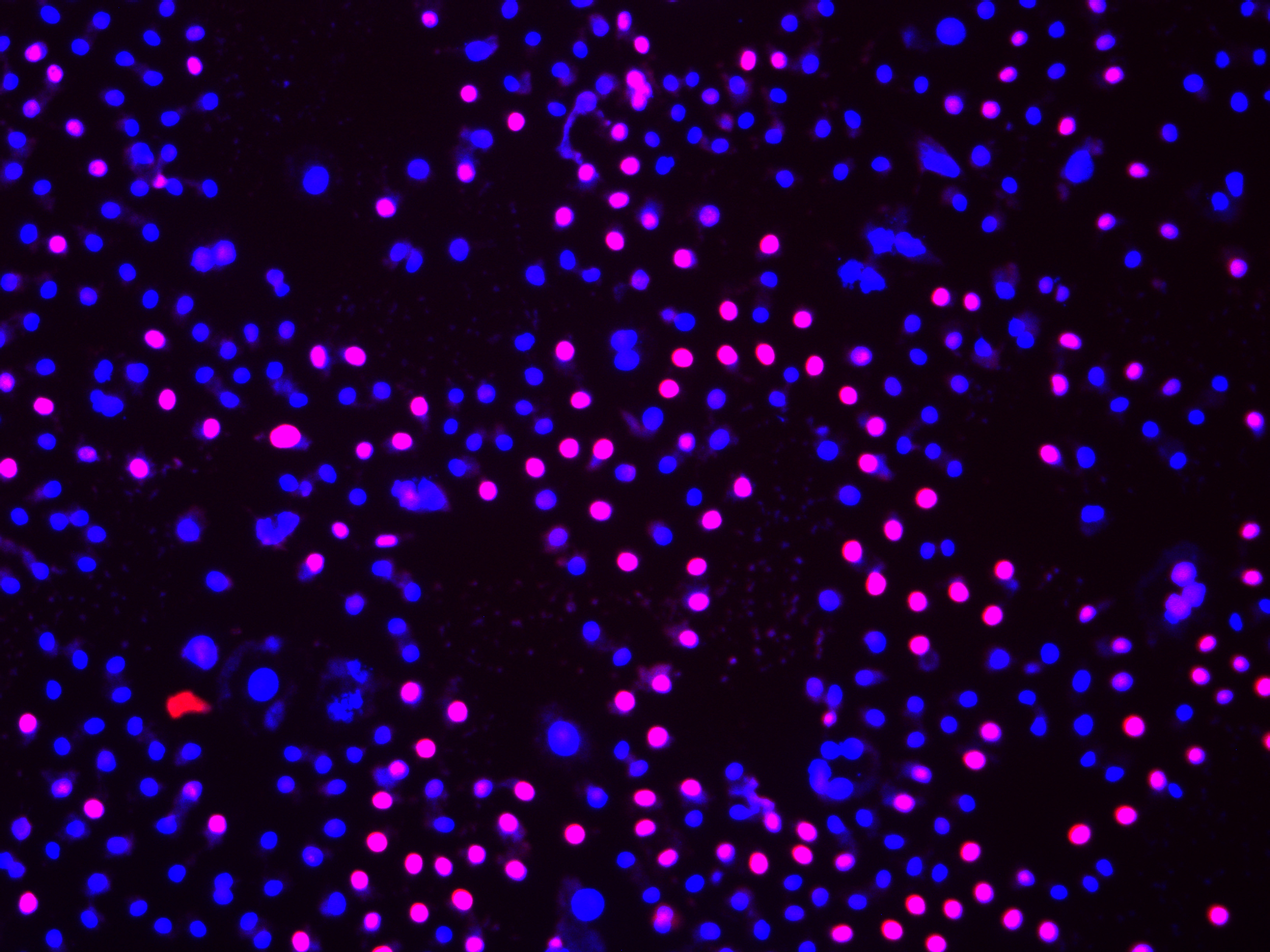

Supplement: S5 File — (ZIP) [file pone.0334639.s005.zip › S 10. File. Original FIgures. Fig.3/3d SMMC-7721/10ngml-M.jpg]

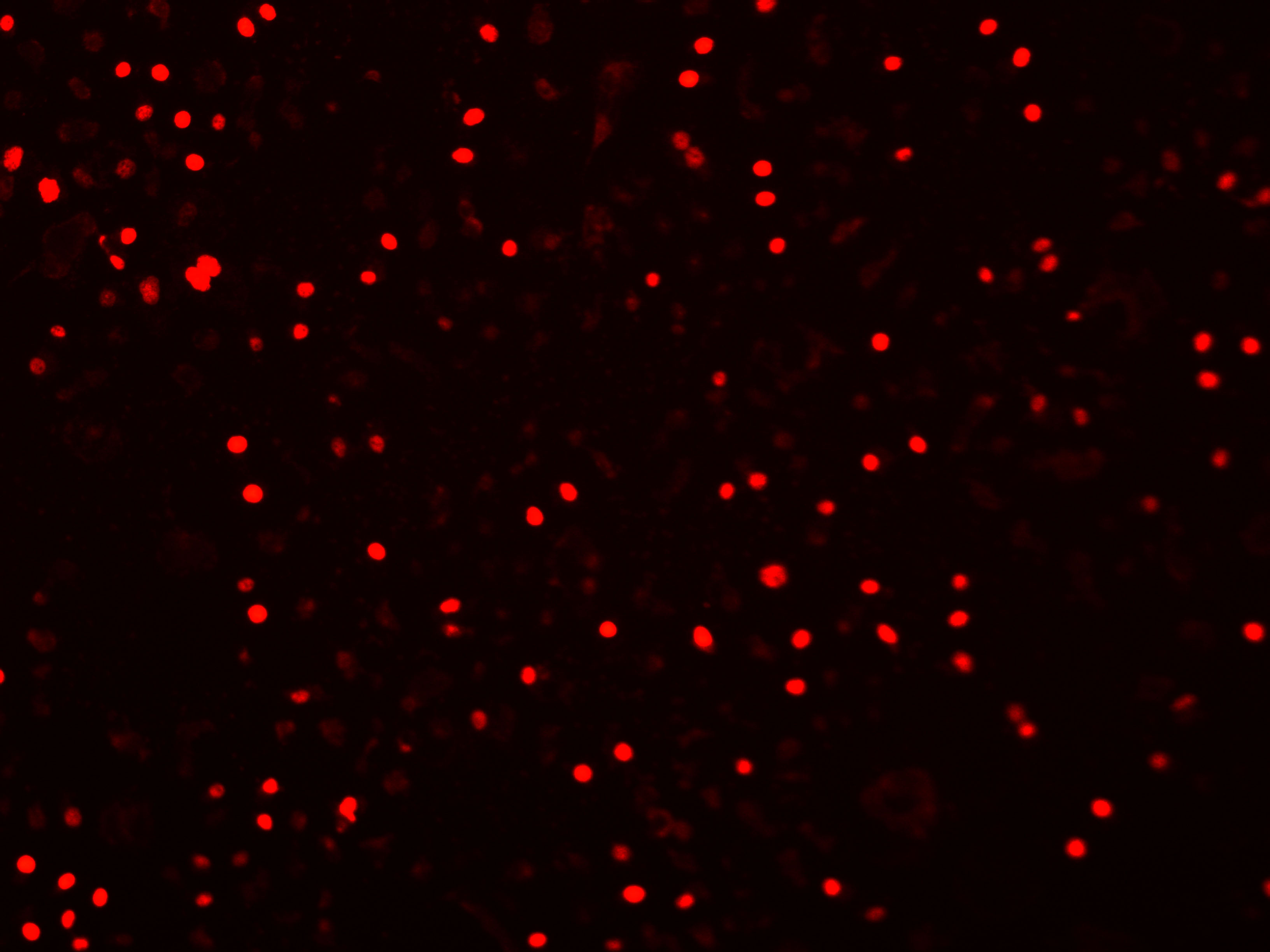

Supplement: S5 File — (ZIP) [file pone.0334639.s005.zip › S 10. File. Original FIgures. Fig.3/3d SMMC-7721/5ngml-E.jpg]

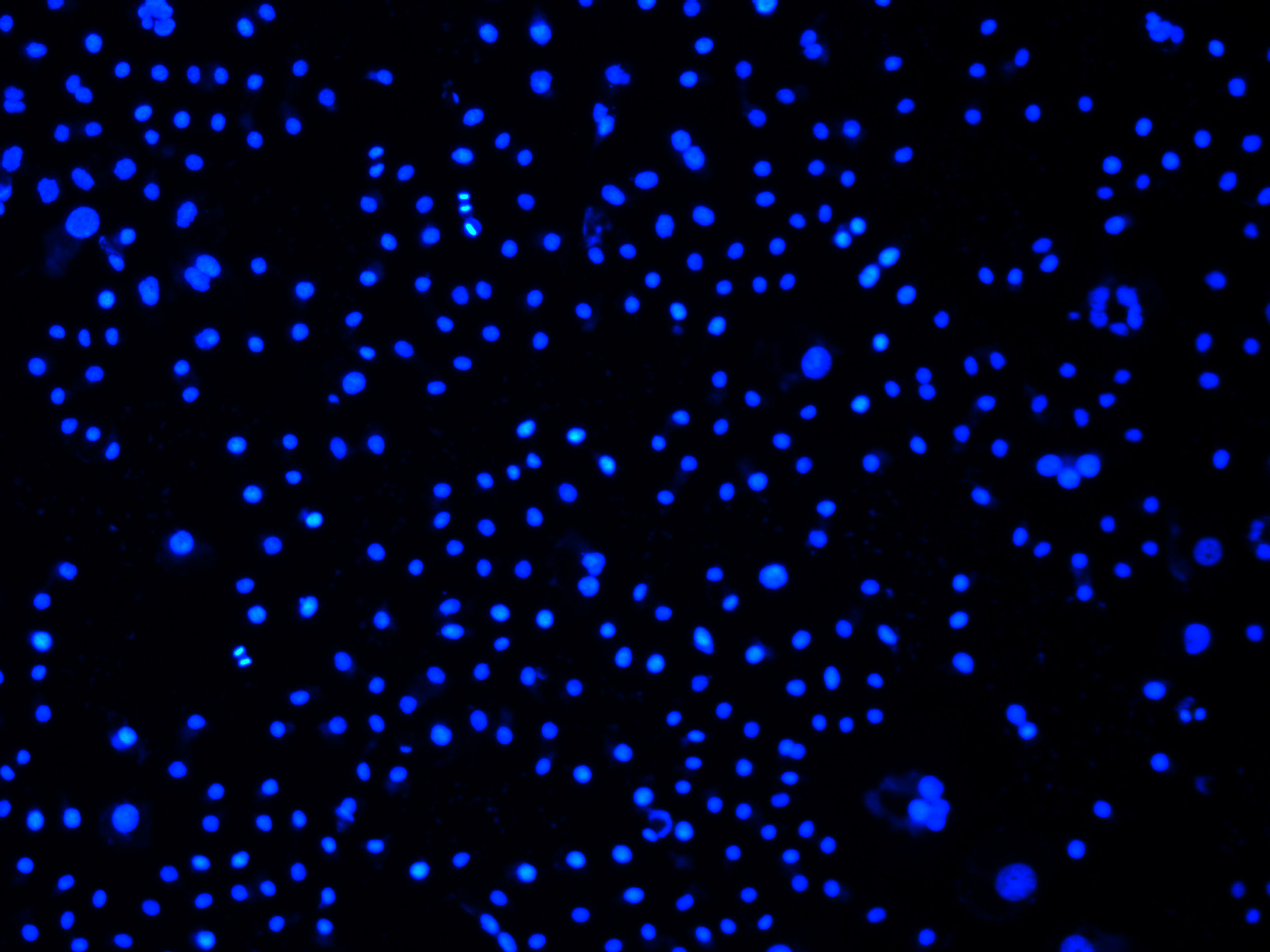

Supplement: S5 File — (ZIP) [file pone.0334639.s005.zip › S 10. File. Original FIgures. Fig.3/3d SMMC-7721/5ngml-H.jpg]

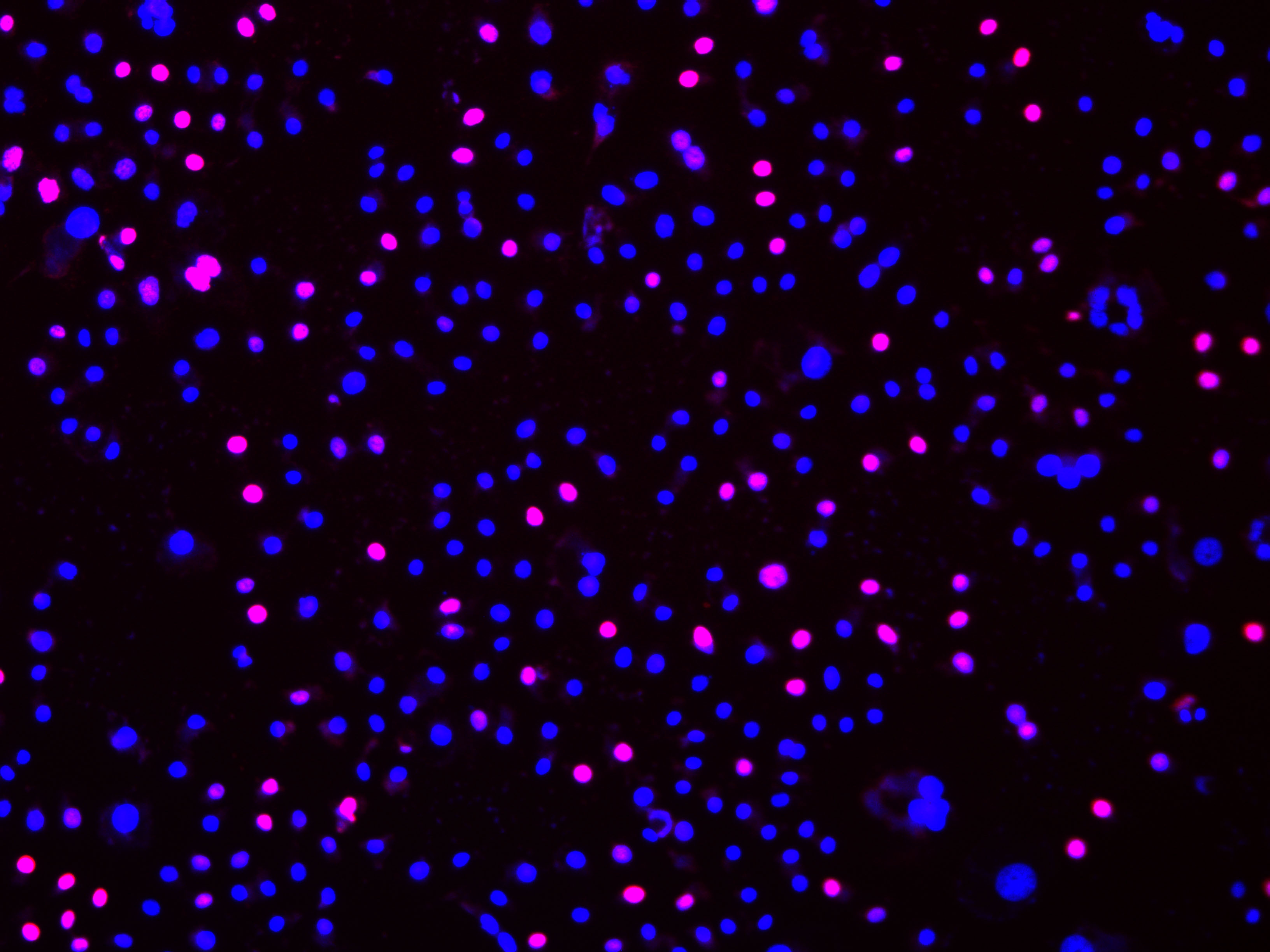

Supplement: S5 File — (ZIP) [file pone.0334639.s005.zip › S 10. File. Original FIgures. Fig.3/3d SMMC-7721/5ngml-M.jpg]

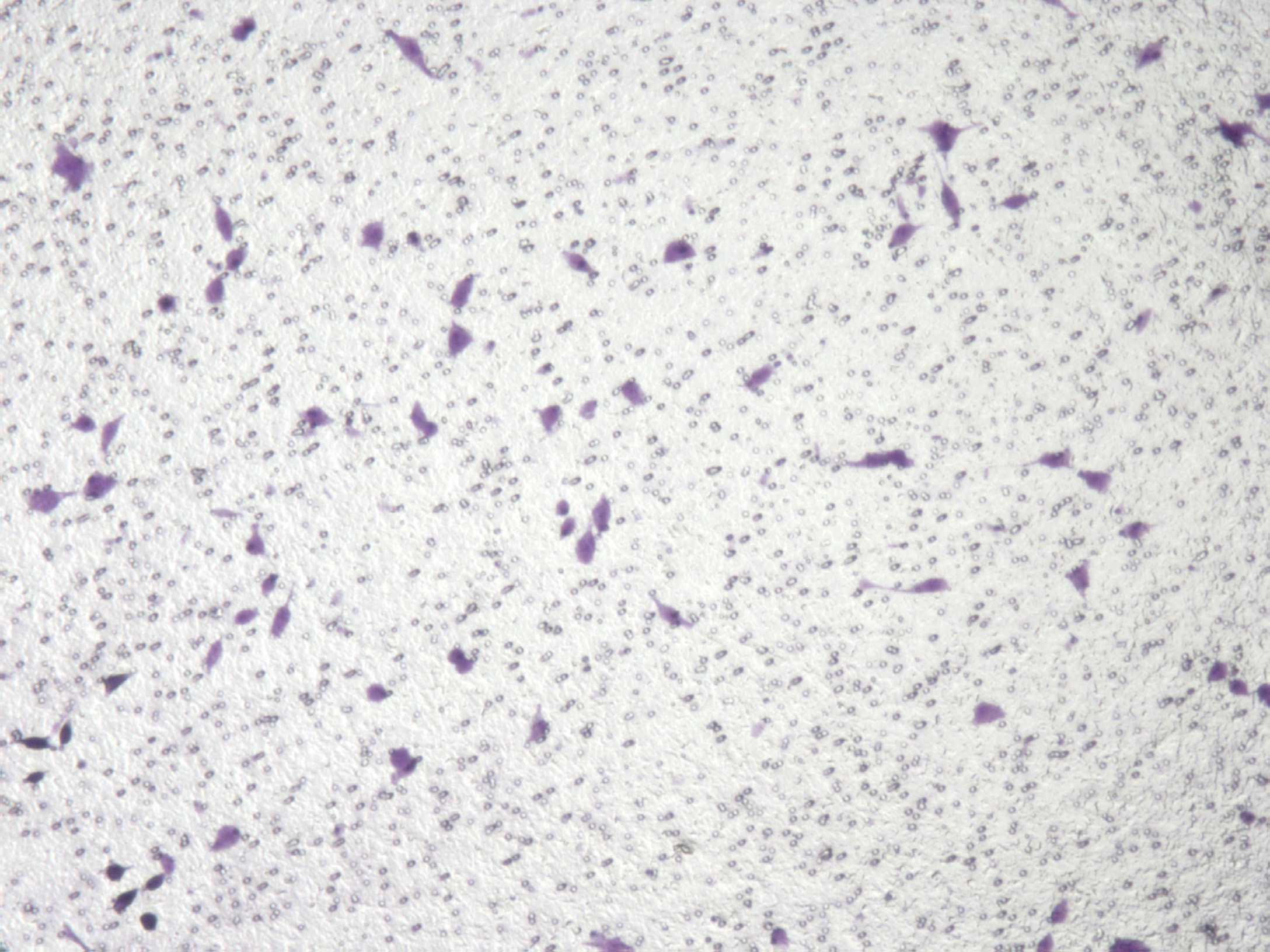

Supplement: S5 File — (ZIP) [file pone.0334639.s005.zip › S 10. File. Original FIgures. Fig.3/3e/bel-7402-0ngml.jpg]

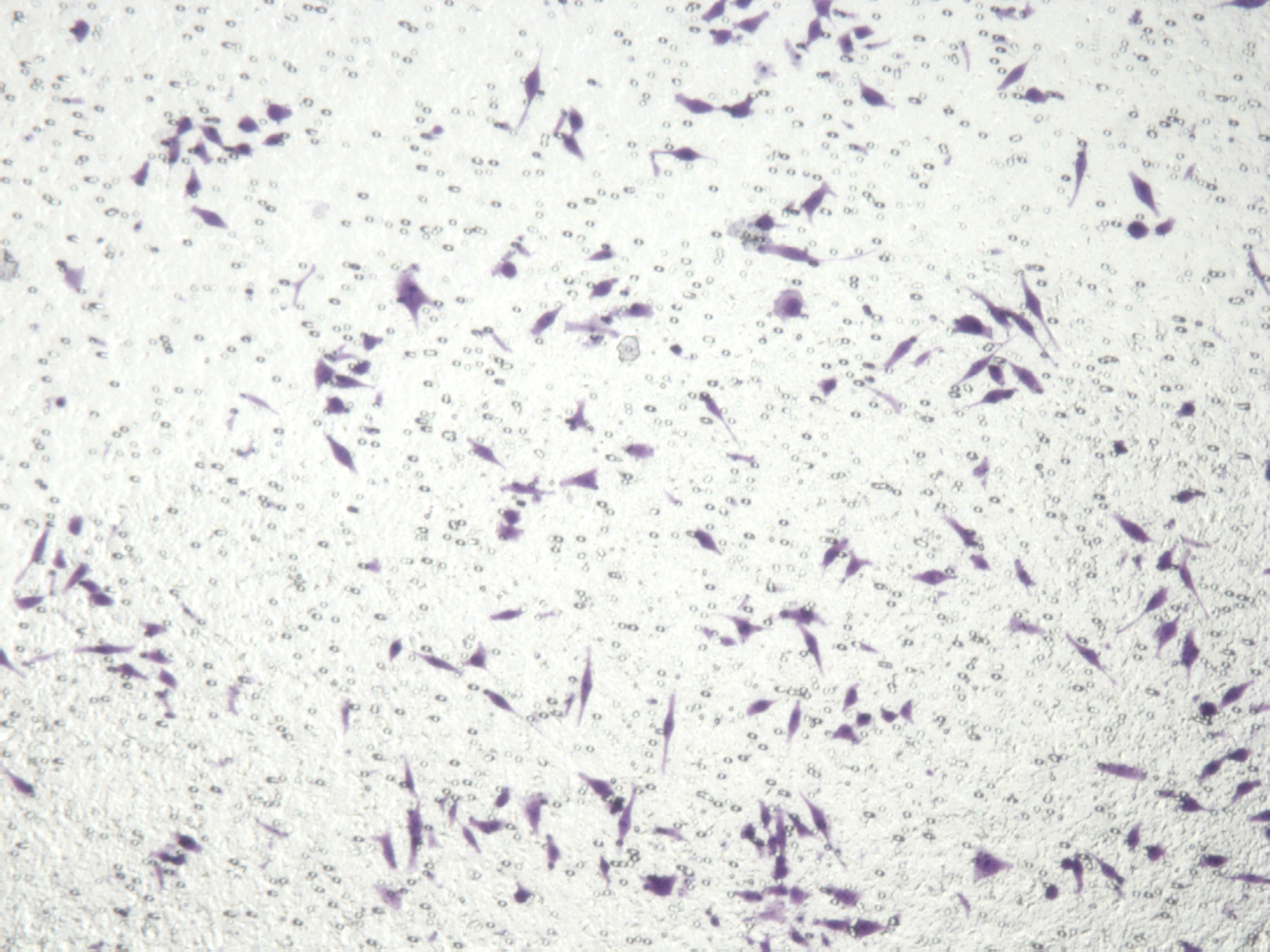

Supplement: S5 File — (ZIP) [file pone.0334639.s005.zip › S 10. File. Original FIgures. Fig.3/3e/bel-7402-10ngml .jpg]

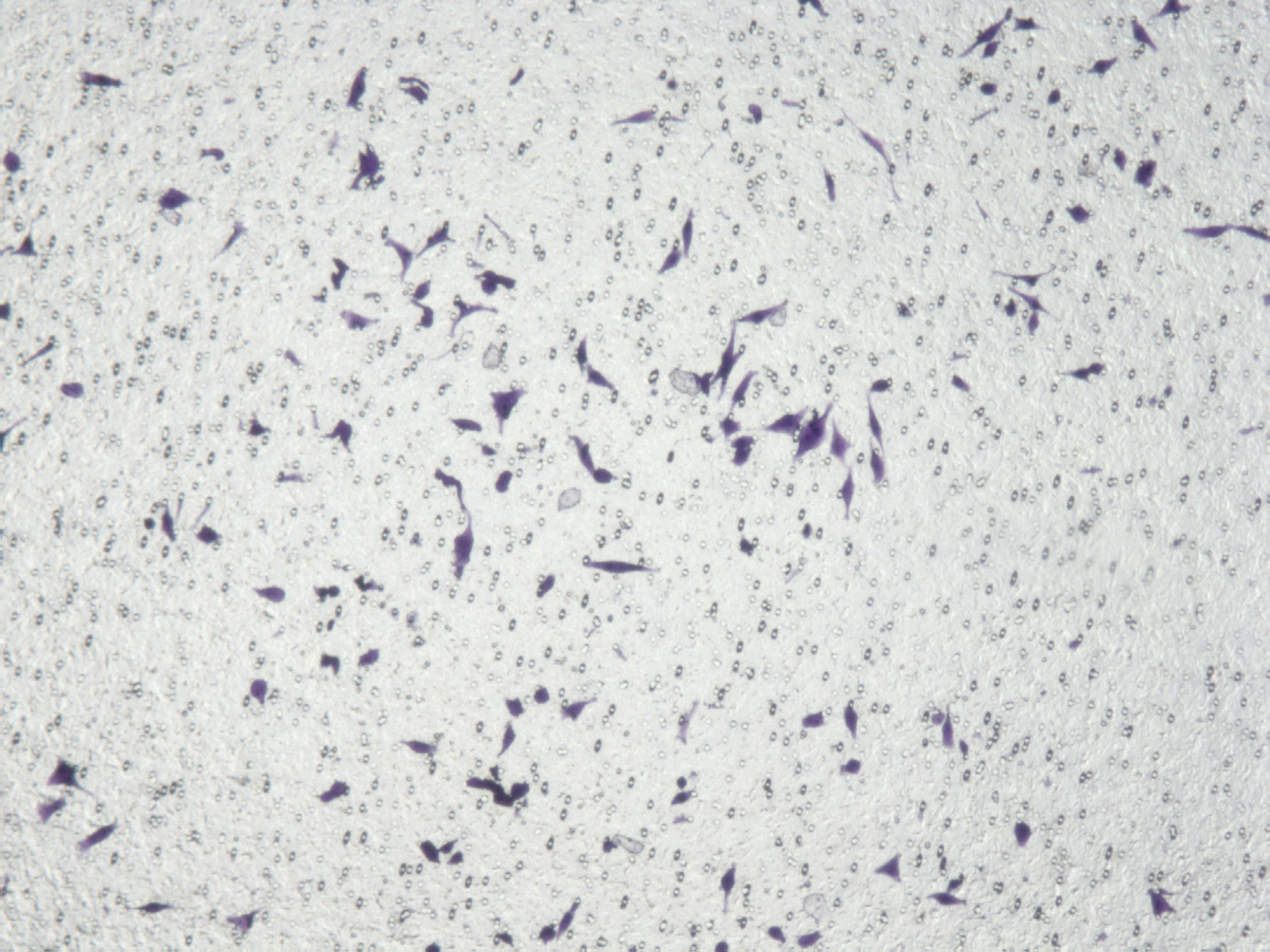

Supplement: S5 File — (ZIP) [file pone.0334639.s005.zip › S 10. File. Original FIgures. Fig.3/3e/bel-7402-20ngml.jpg]

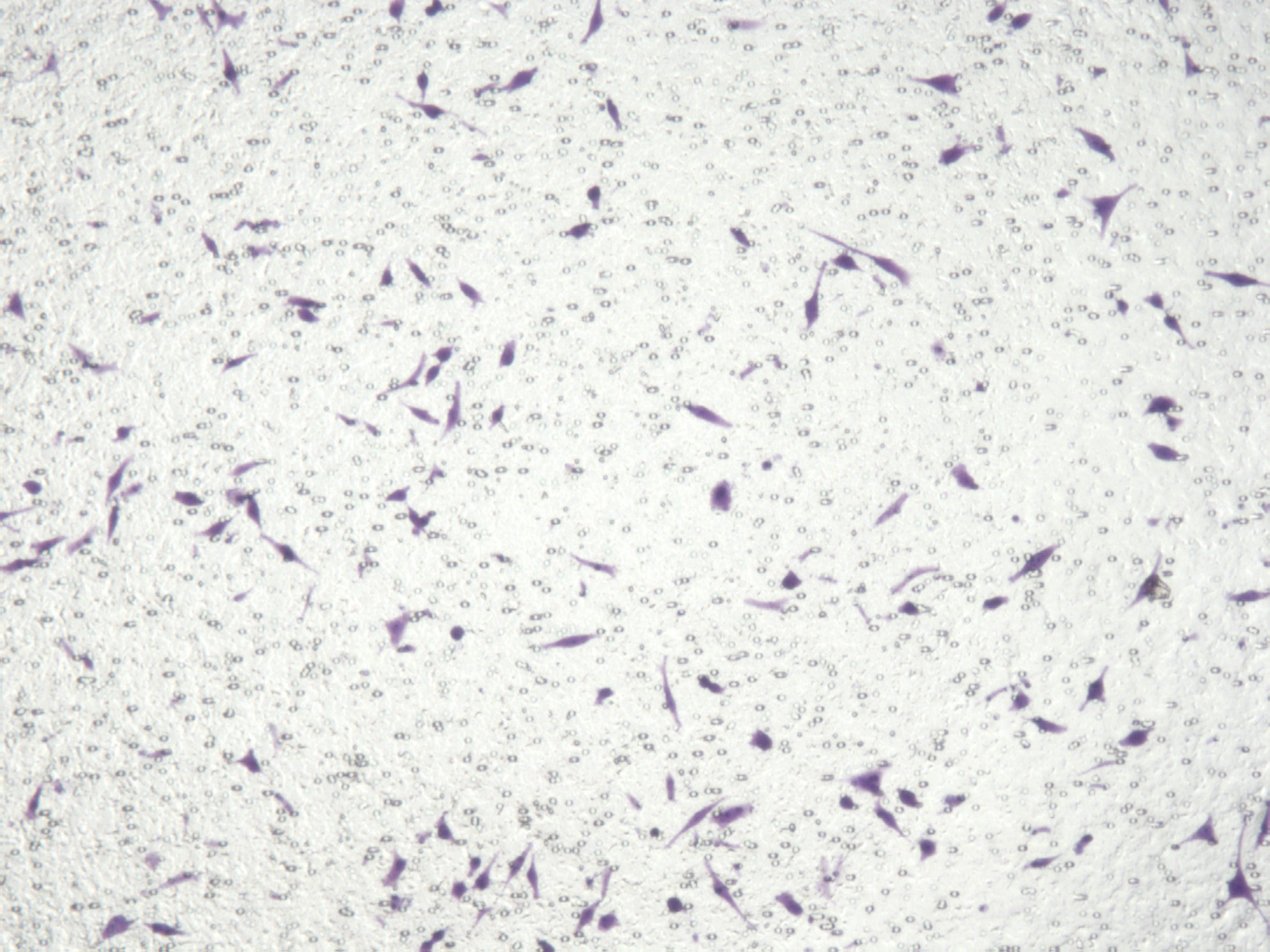

Supplement: S5 File — (ZIP) [file pone.0334639.s005.zip › S 10. File. Original FIgures. Fig.3/3e/bel-7402-2ngml.jpg]

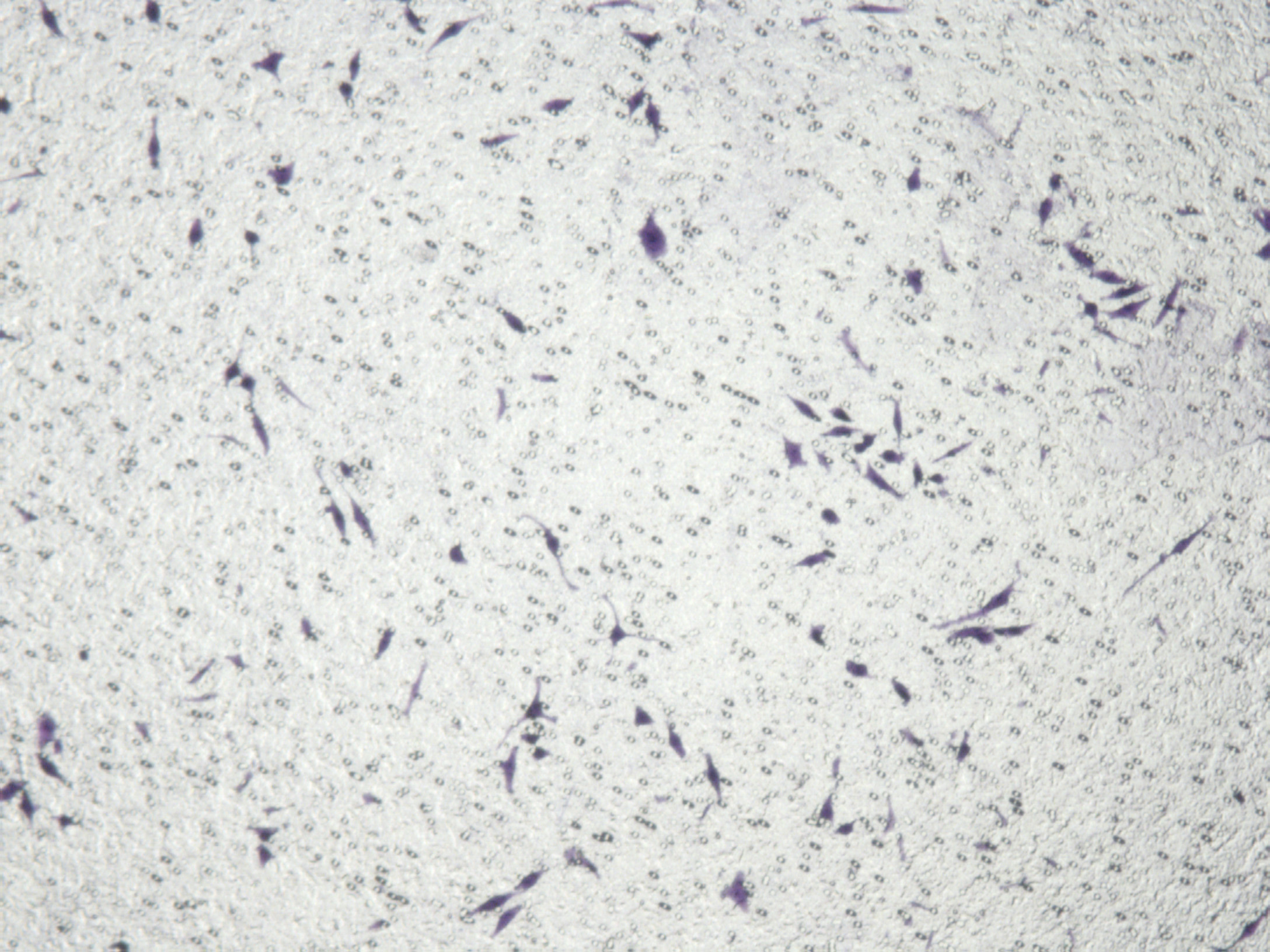

Supplement: S5 File — (ZIP) [file pone.0334639.s005.zip › S 10. File. Original FIgures. Fig.3/3e/bel-7402-30ngml.jpg]

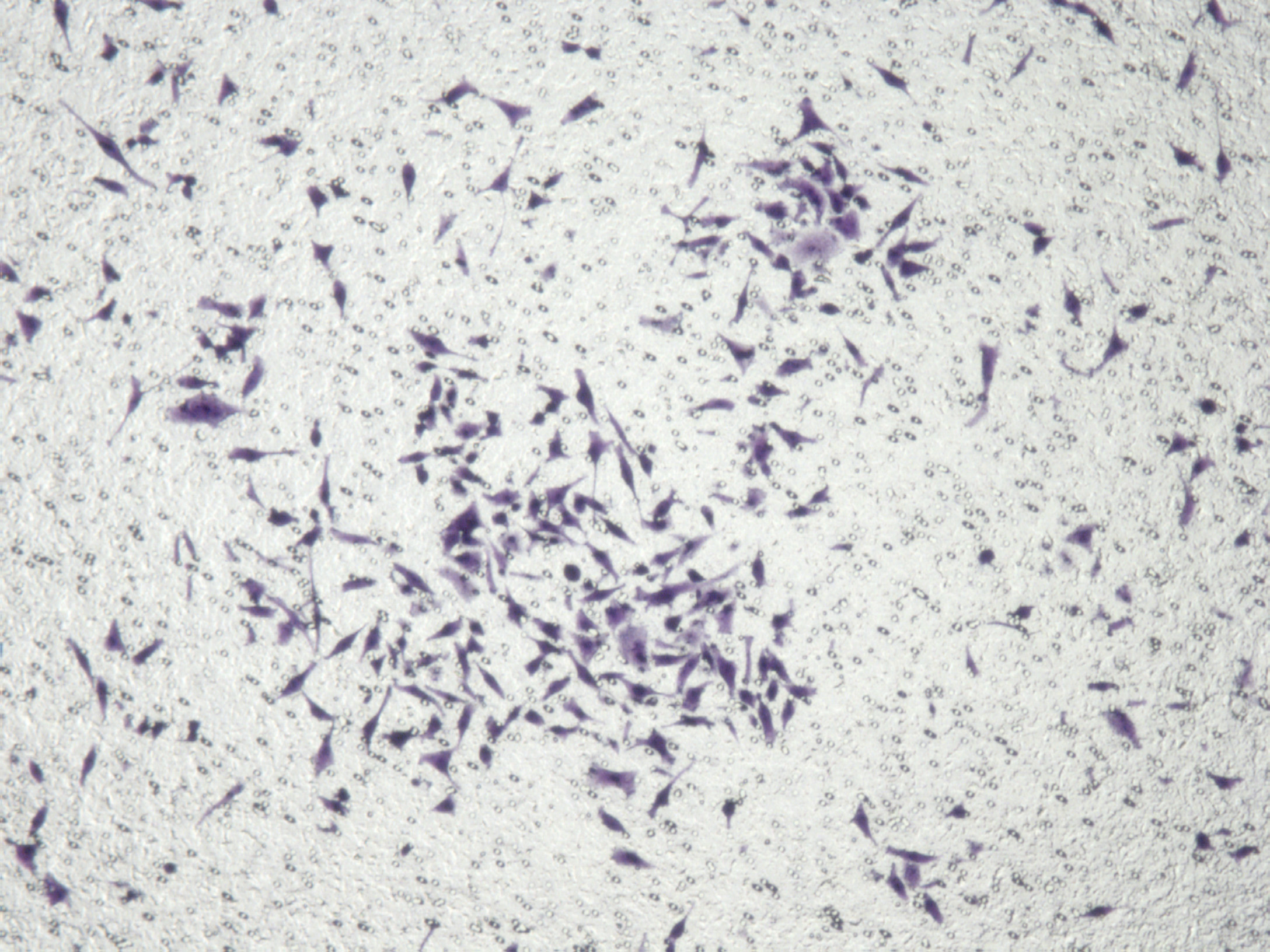

Supplement: S5 File — (ZIP) [file pone.0334639.s005.zip › S 10. File. Original FIgures. Fig.3/3e/bel-7402-5ngml.jpg]

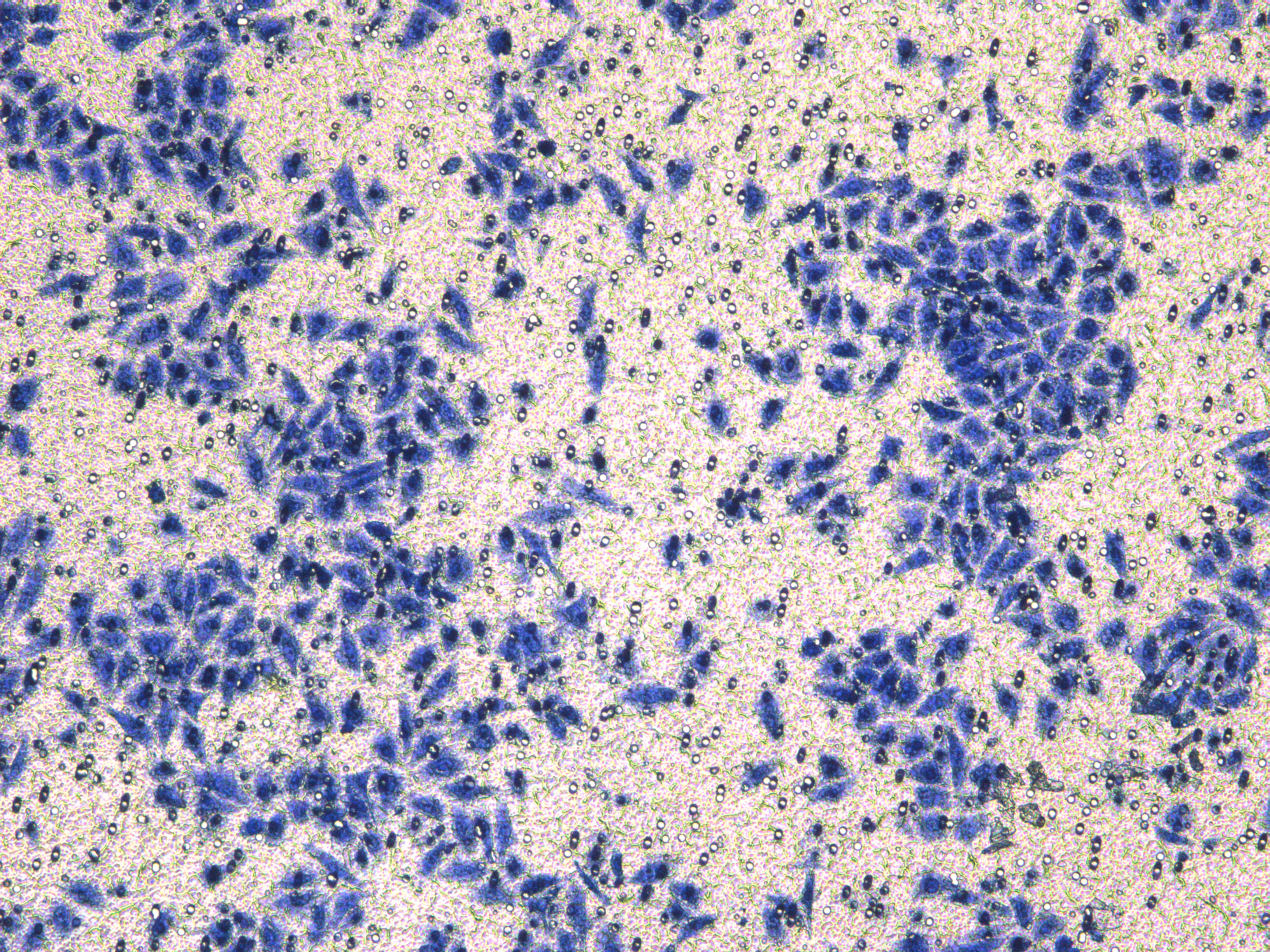

Supplement: S5 File — (ZIP) [file pone.0334639.s005.zip › S 10. File. Original FIgures. Fig.3/3f/Hepg2 0ngml.jpg]

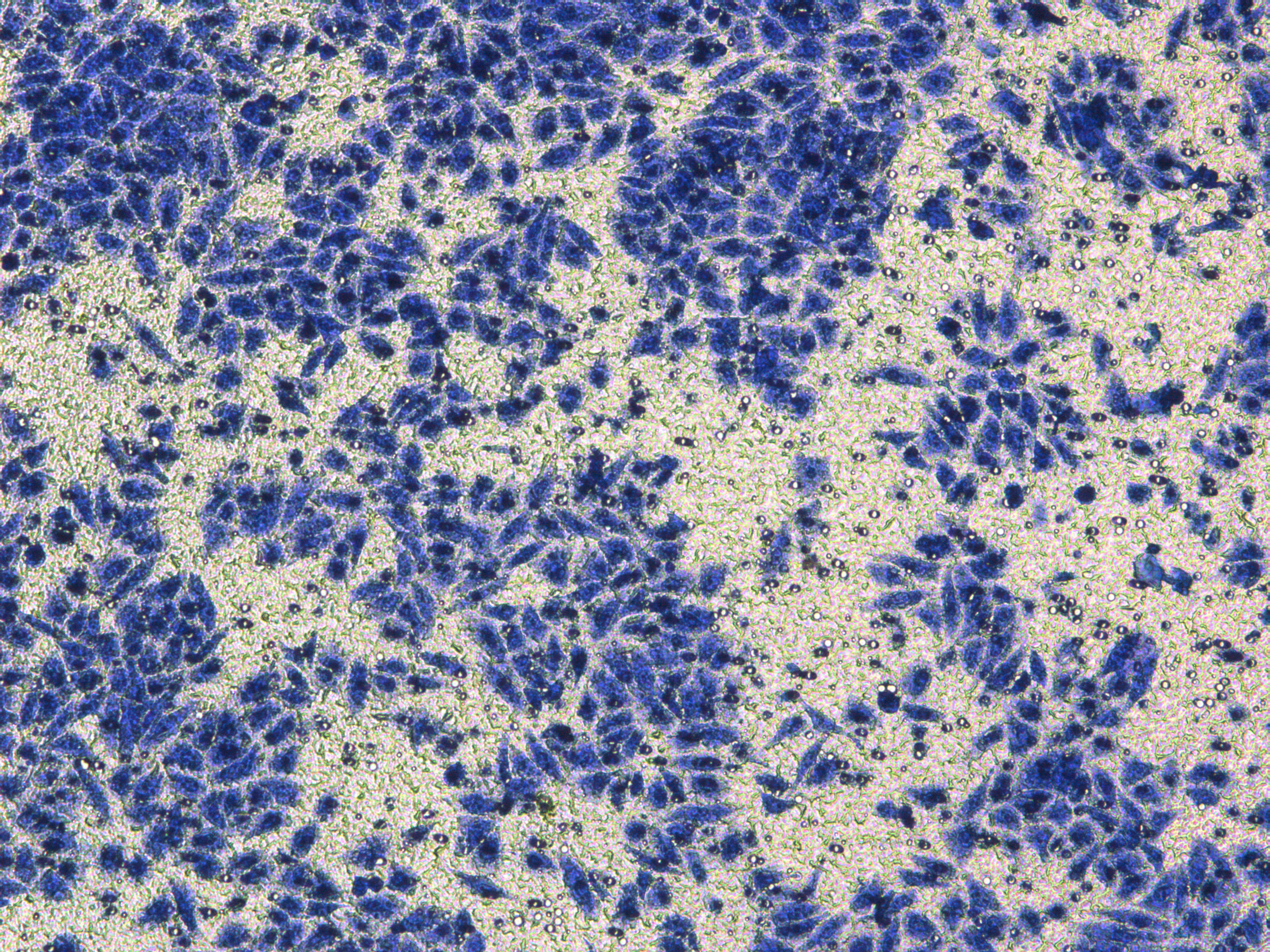

Supplement: S5 File — (ZIP) [file pone.0334639.s005.zip › S 10. File. Original FIgures. Fig.3/3f/Hepg2 10ngml.jpg]

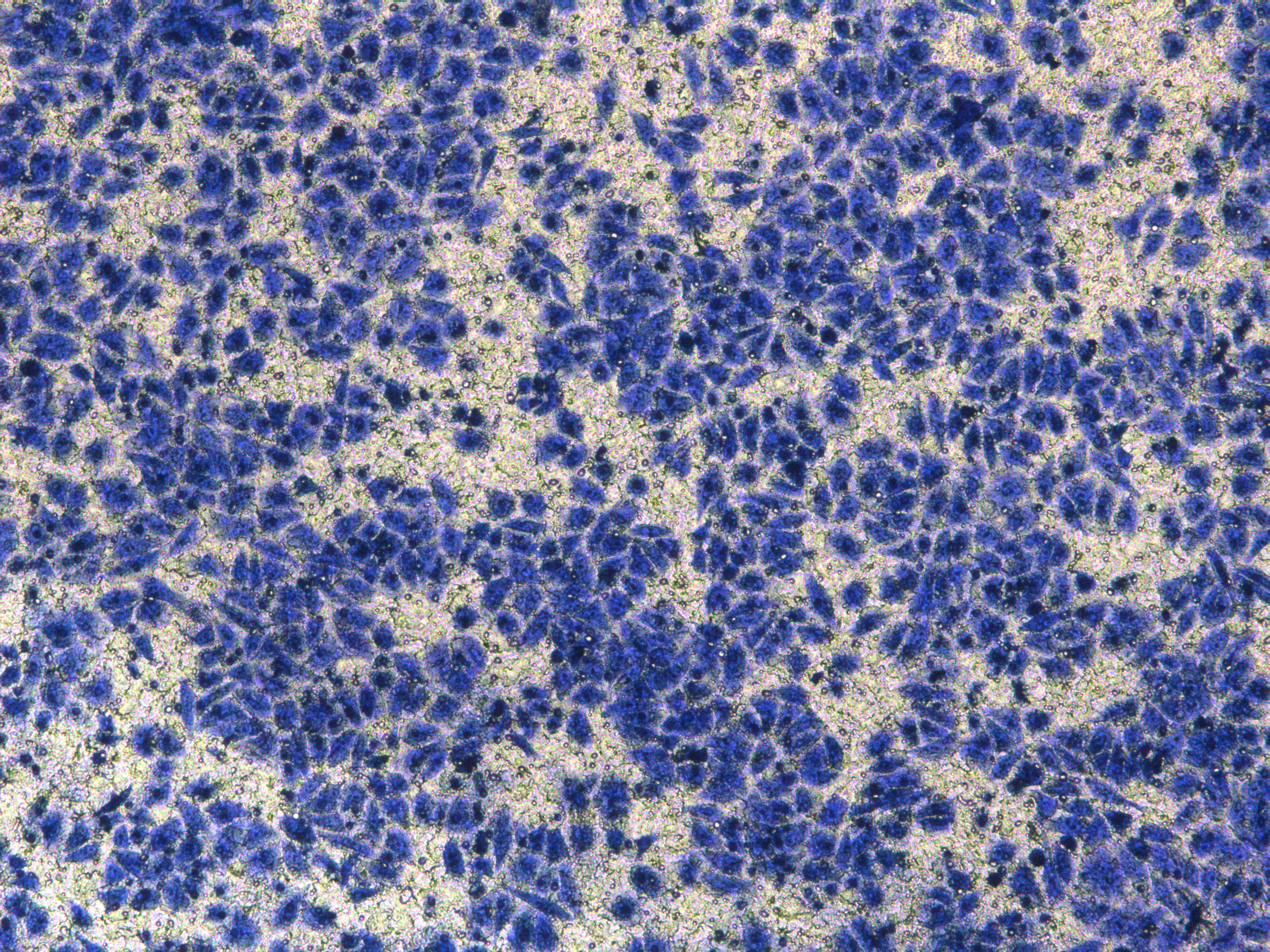

Supplement: S5 File — (ZIP) [file pone.0334639.s005.zip › S 10. File. Original FIgures. Fig.3/3f/Hepg2 20ngml.jpg]

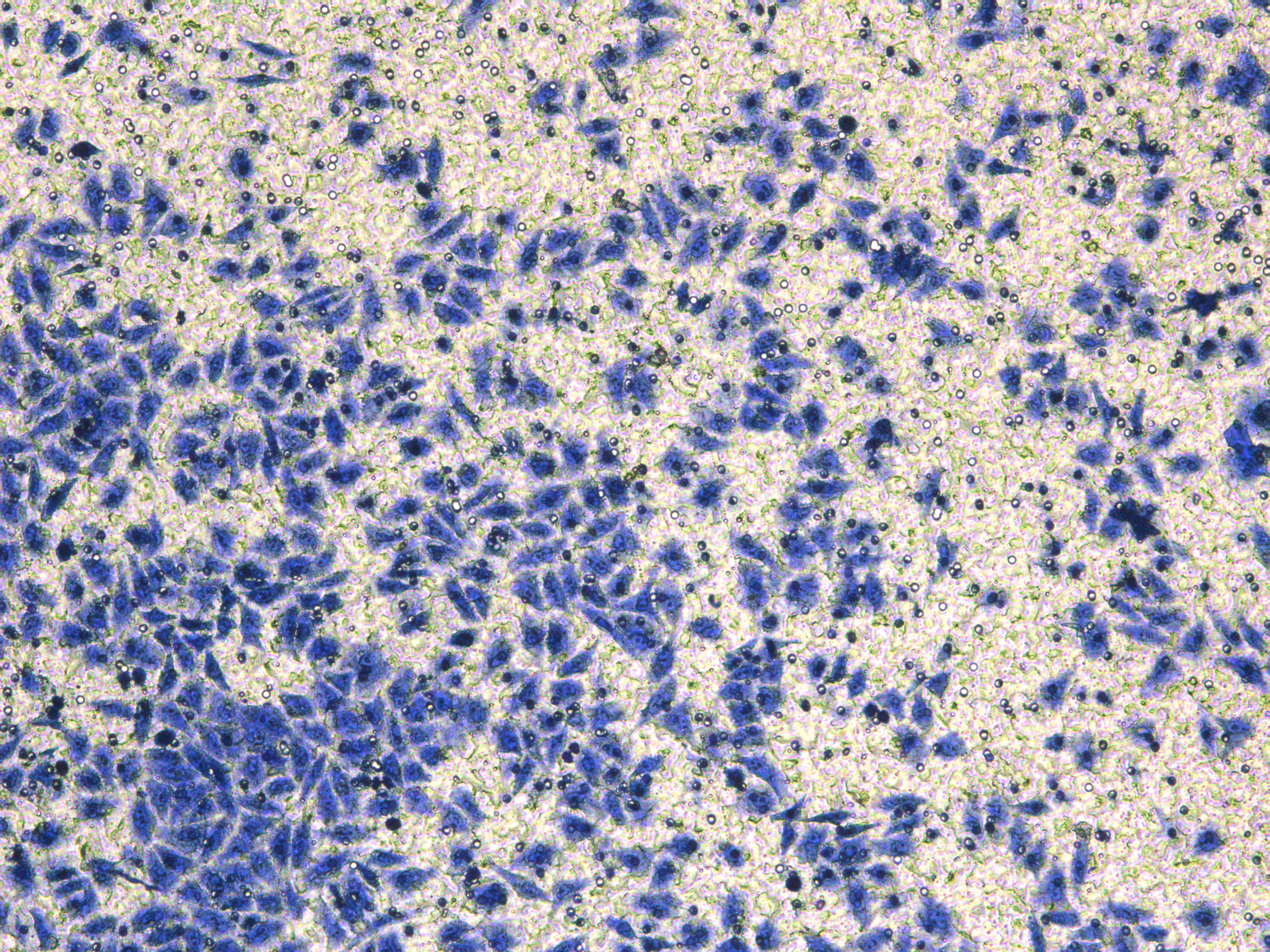

Supplement: S5 File — (ZIP) [file pone.0334639.s005.zip › S 10. File. Original FIgures. Fig.3/3f/Hepg2 2ngml.jpg]

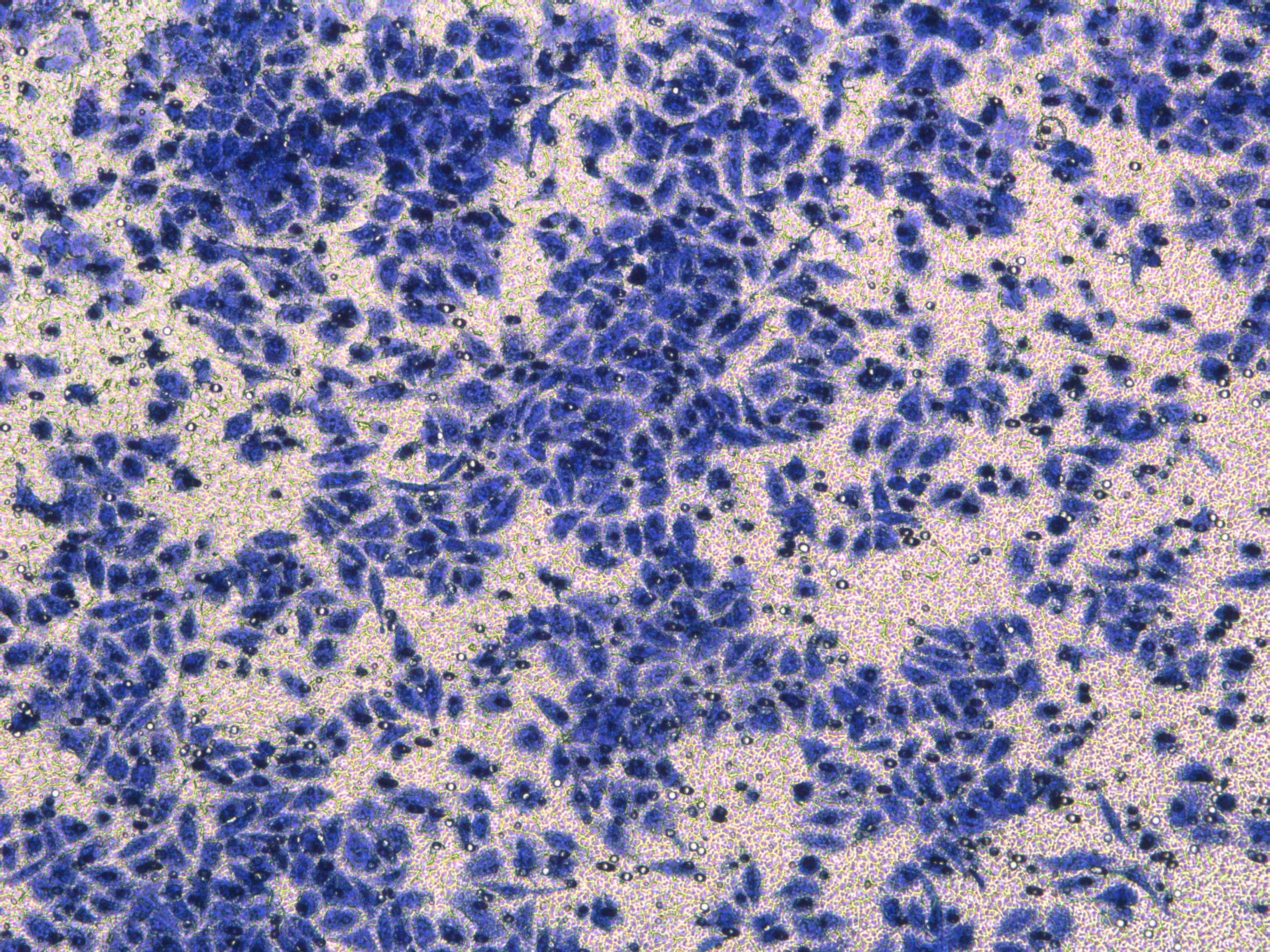

Supplement: S5 File — (ZIP) [file pone.0334639.s005.zip › S 10. File. Original FIgures. Fig.3/3f/Hepg2 30ngml.jpg]

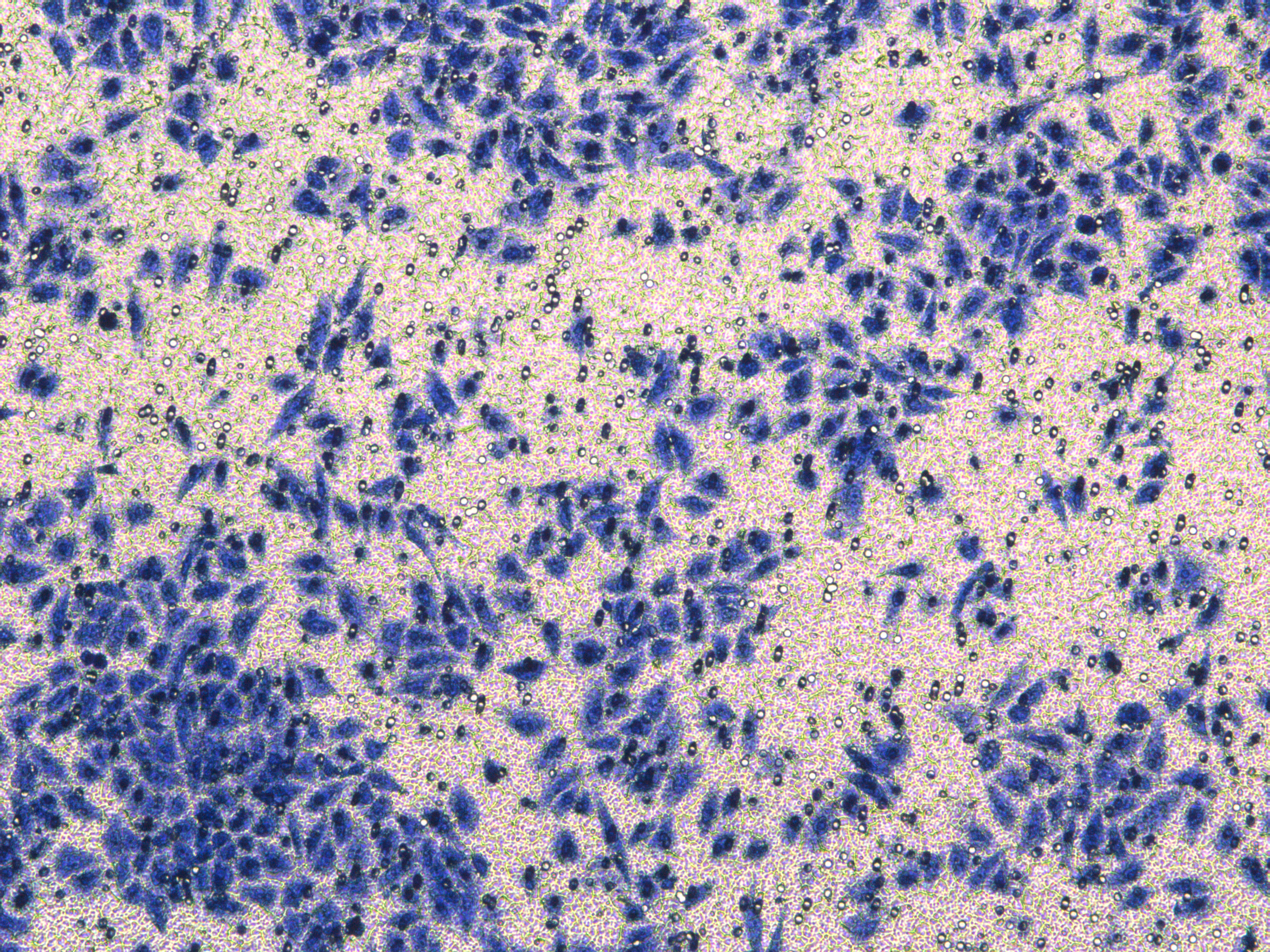

Supplement: S5 File — (ZIP) [file pone.0334639.s005.zip › S 10. File. Original FIgures. Fig.3/3f/Hepg2 5ngml.jpg]

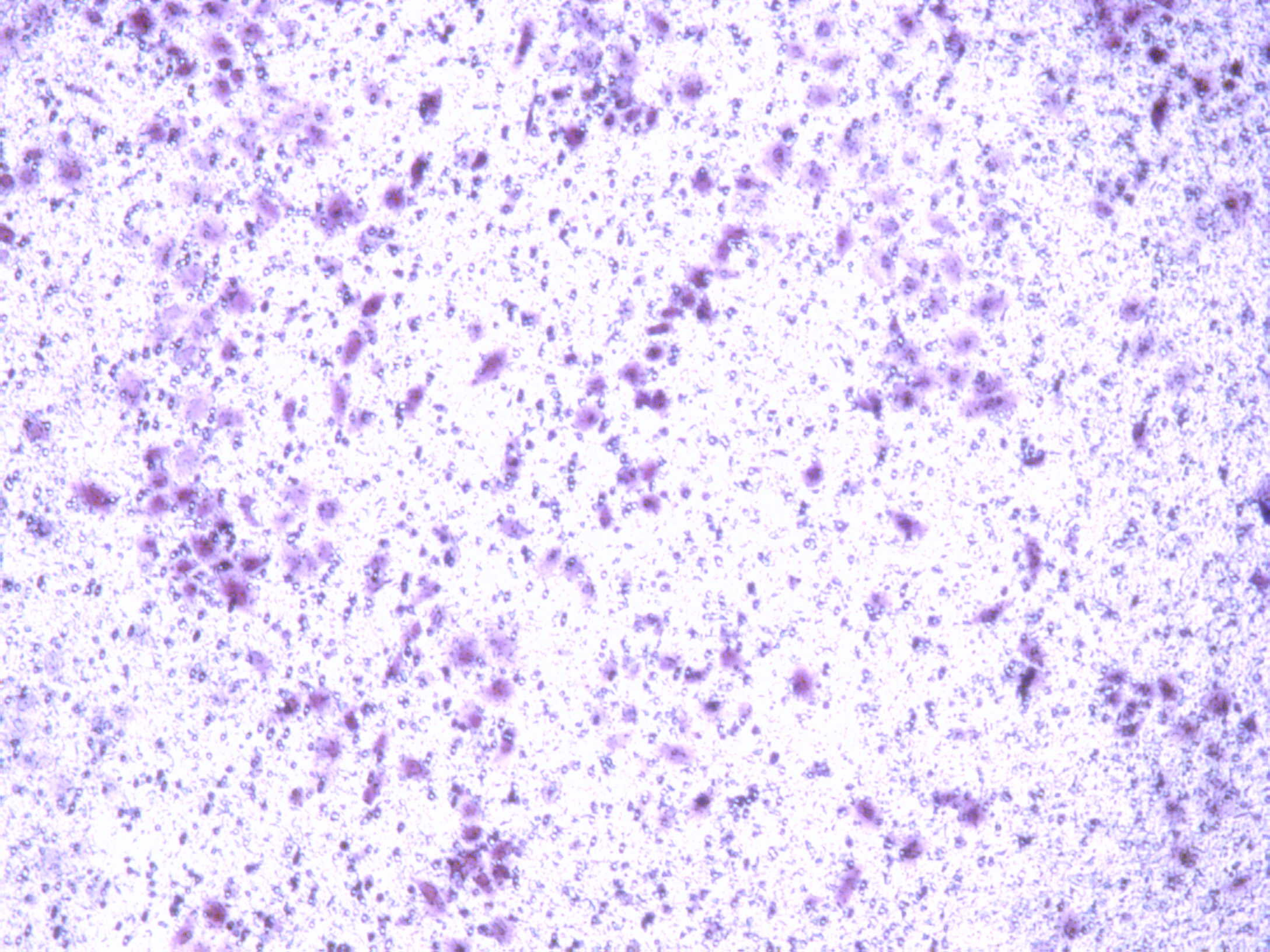

Supplement: S5 File — (ZIP) [file pone.0334639.s005.zip › S 10. File. Original FIgures. Fig.3/3g/SMMC-0ngml.jpg]

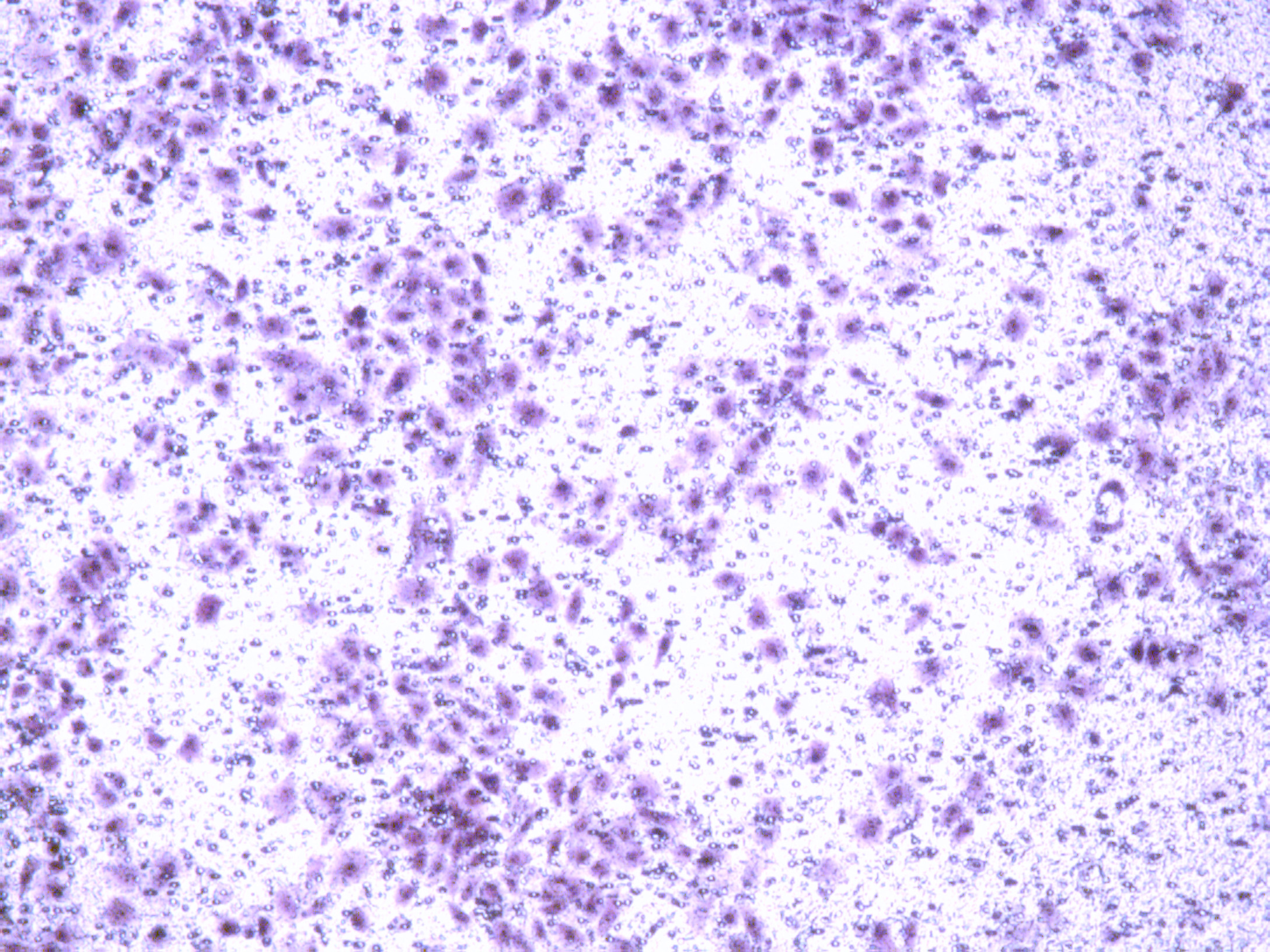

Supplement: S5 File — (ZIP) [file pone.0334639.s005.zip › S 10. File. Original FIgures. Fig.3/3g/SMMC-10ngml.jpg]

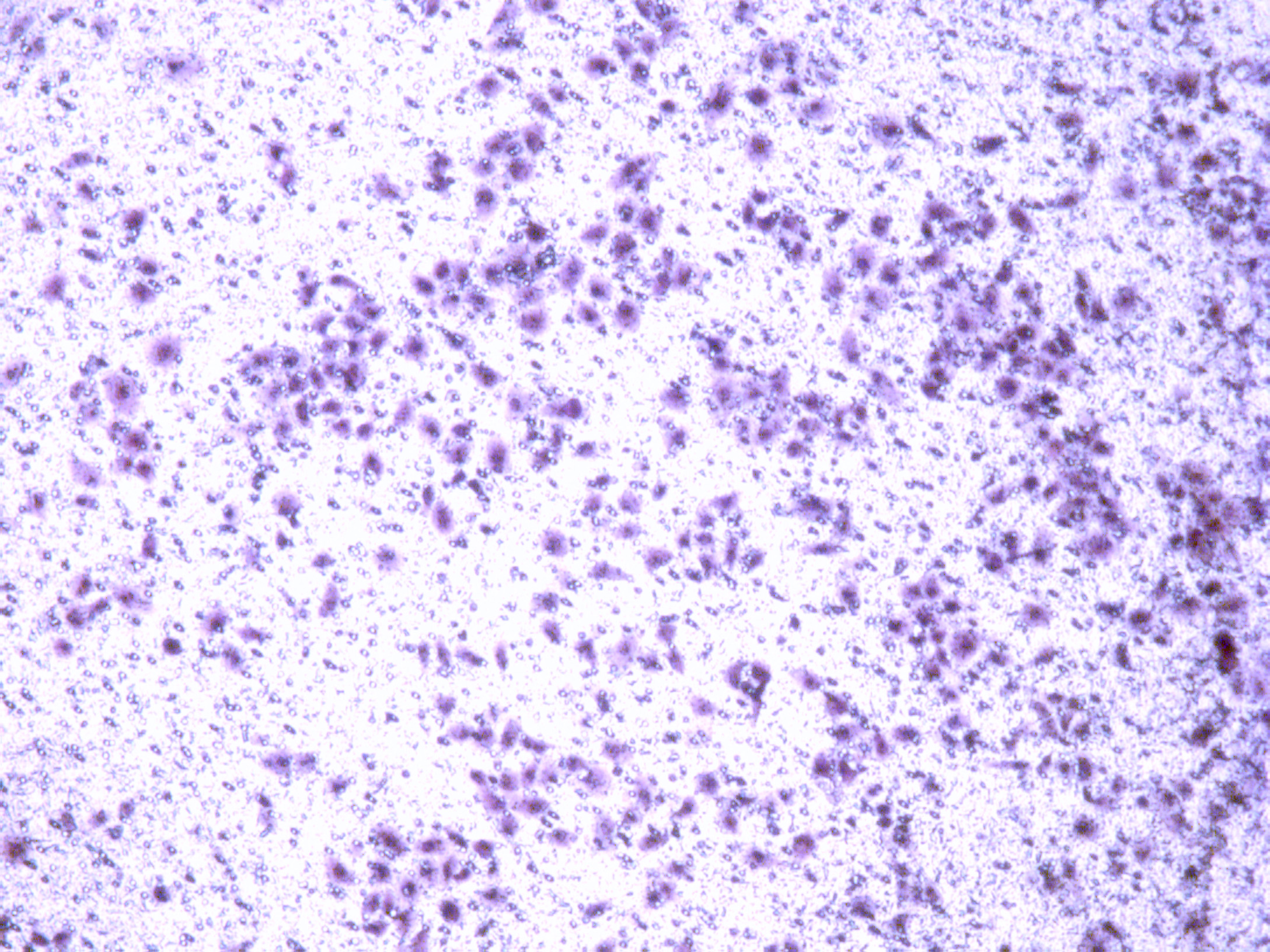

Supplement: S5 File — (ZIP) [file pone.0334639.s005.zip › S 10. File. Original FIgures. Fig.3/3g/SMMC-20ngml.jpg]

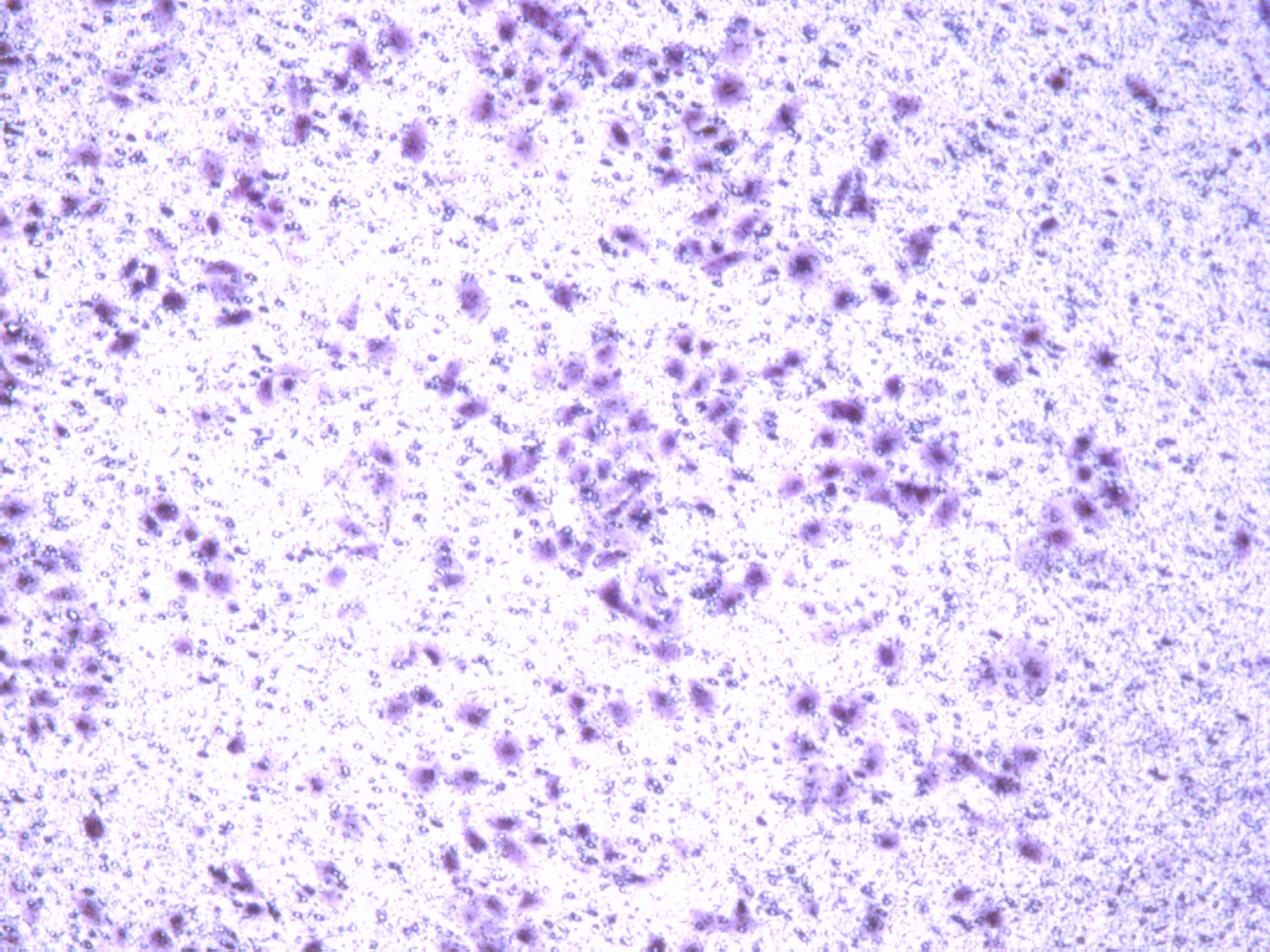

Supplement: S5 File — (ZIP) [file pone.0334639.s005.zip › S 10. File. Original FIgures. Fig.3/3g/SMMC-2ngml.jpg]

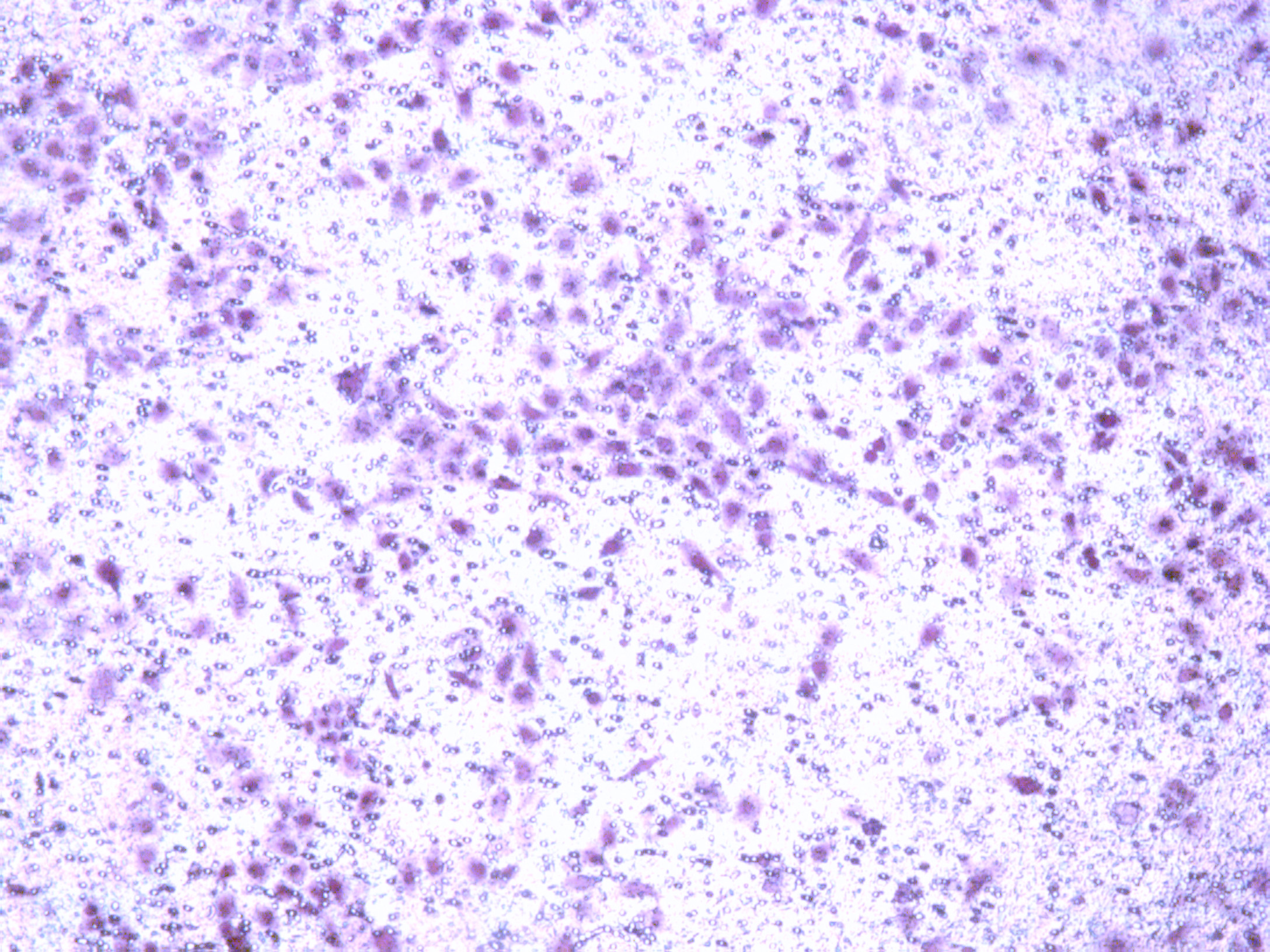

Supplement: S5 File — (ZIP) [file pone.0334639.s005.zip › S 10. File. Original FIgures. Fig.3/3g/SMMC-30ngml.jpg]

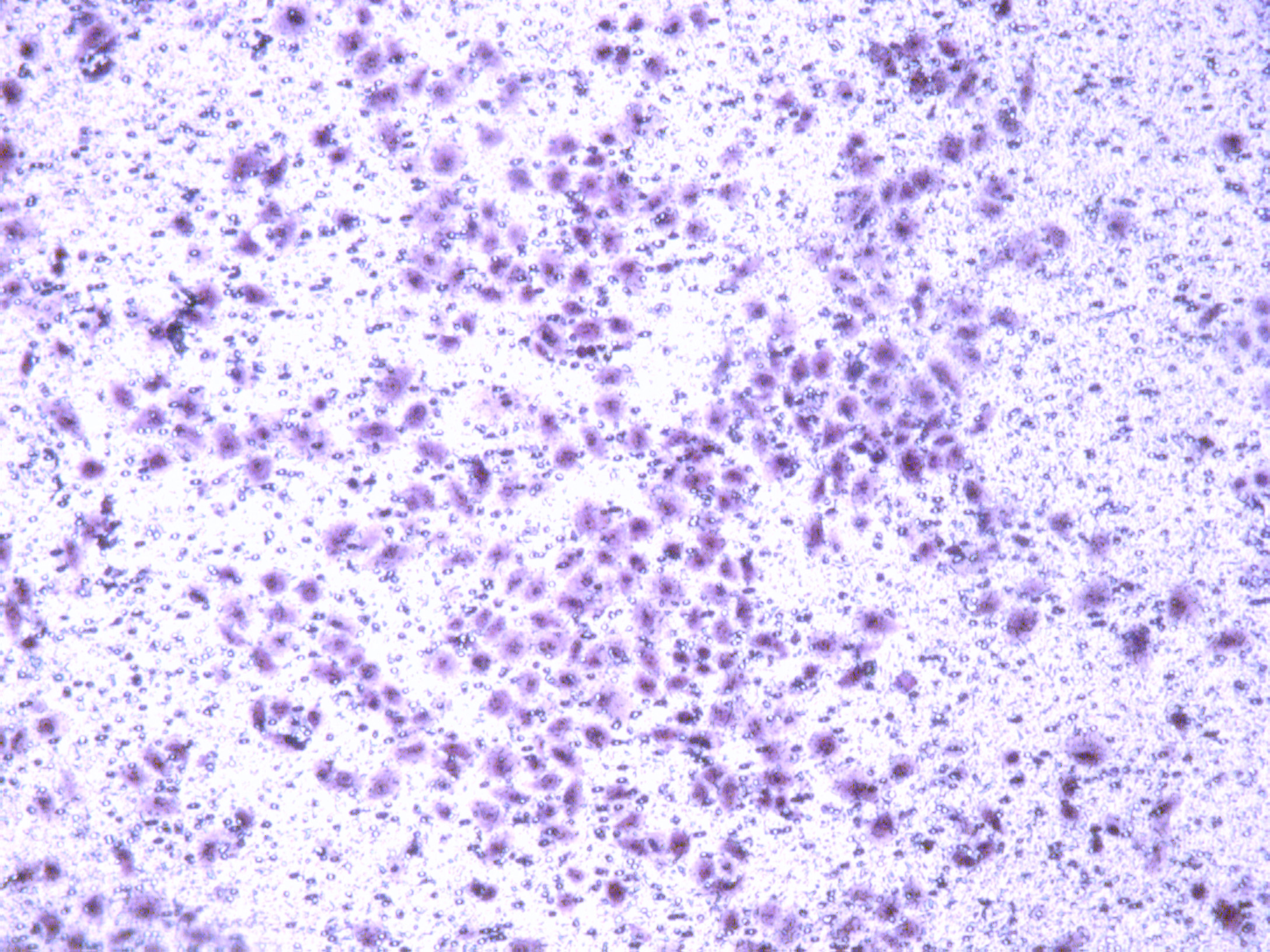

Supplement: S5 File — (ZIP) [file pone.0334639.s005.zip › S 10. File. Original FIgures. Fig.3/3g/SMMC-5ngml.jpg]

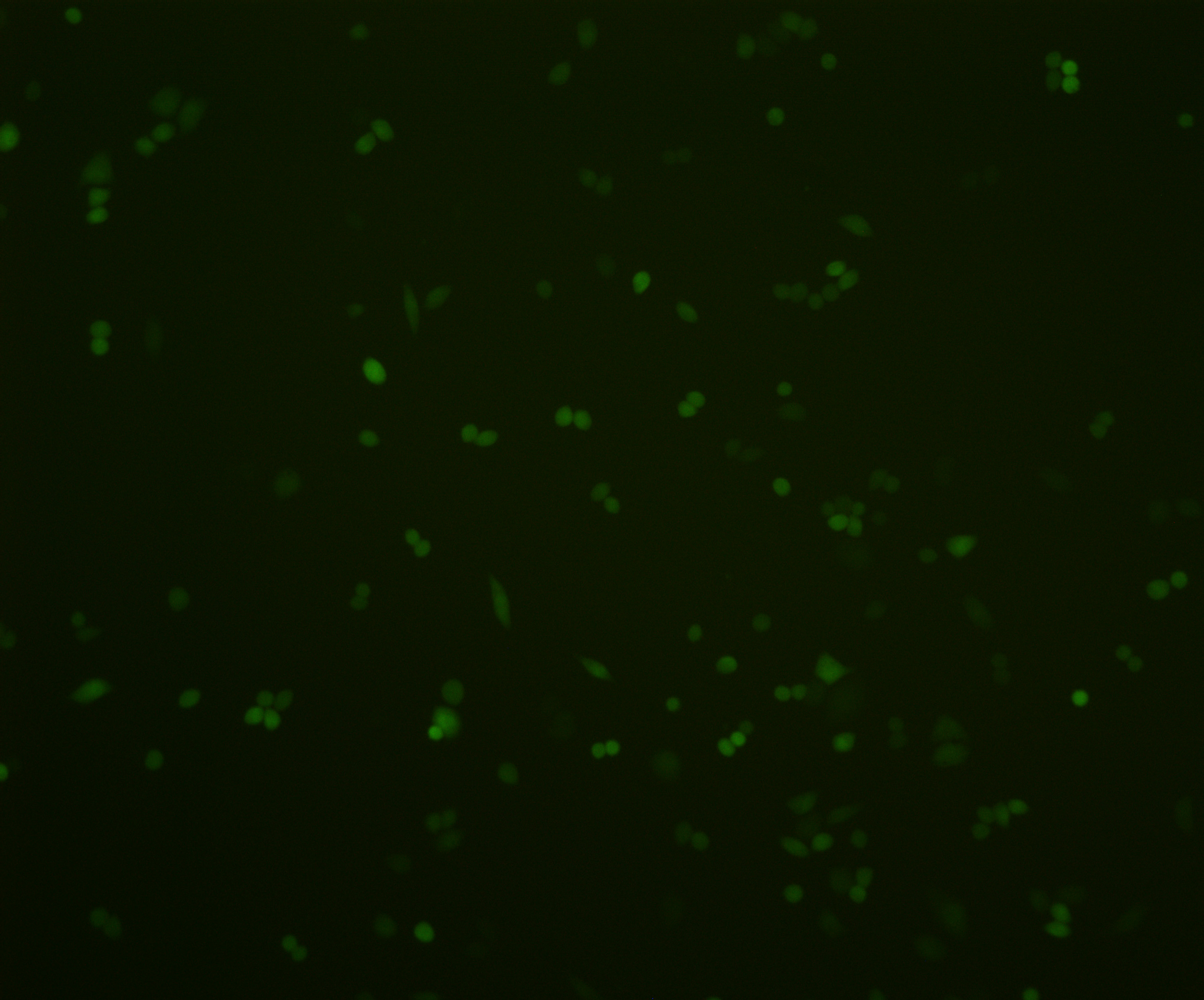

Supplement: S6 File — (ZIP) [file pone.0334639.s006.zip › S 11. File. Original Images. Fig4/S 11. File. Original FIgures. Fig.4/4a/Bel-7402/BEI-7402 mock-yg.jpg]

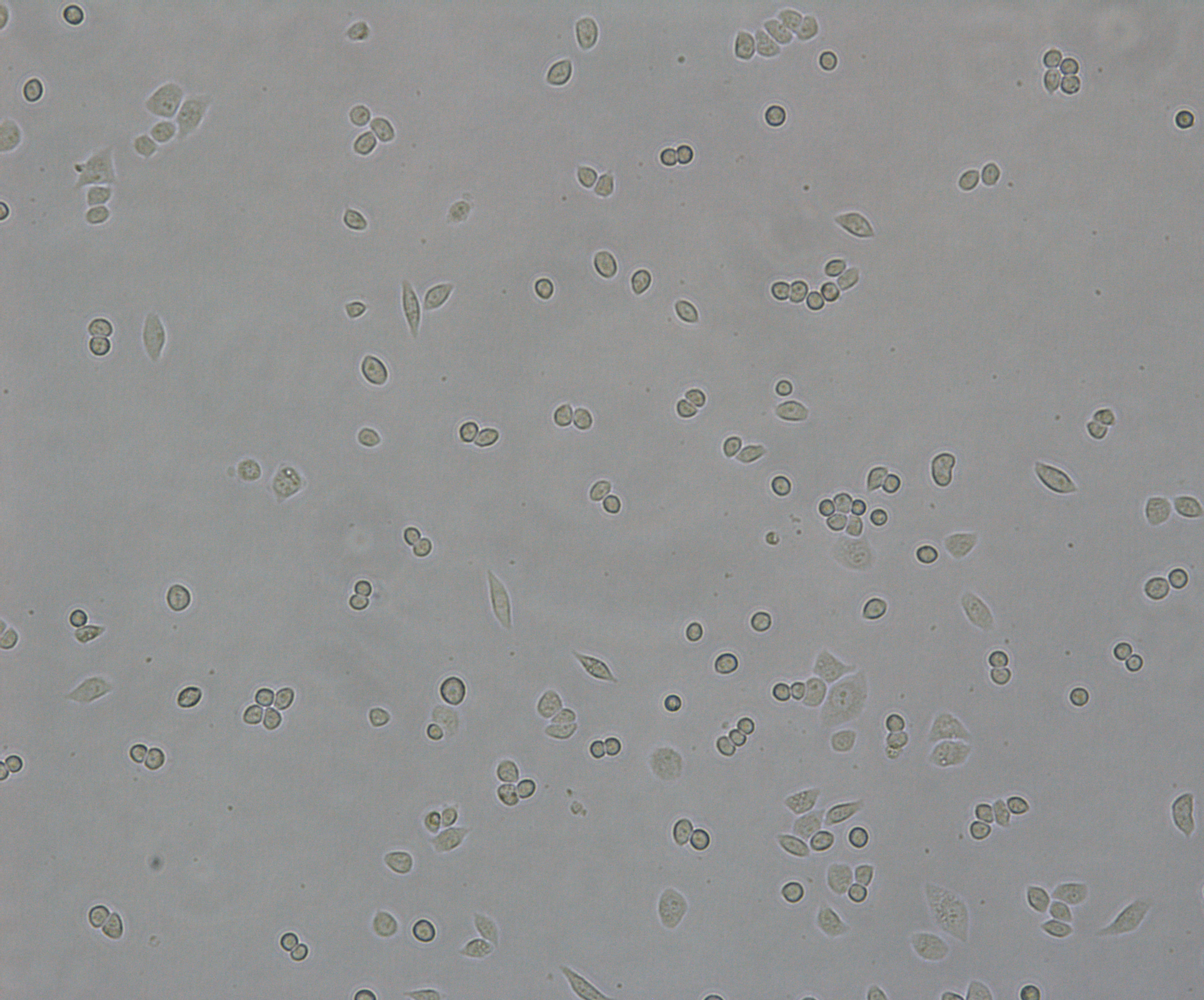

Supplement: S6 File — (ZIP) [file pone.0334639.s006.zip › S 11. File. Original Images. Fig4/S 11. File. Original FIgures. Fig.4/4a/Bel-7402/BEI-7402 Mock-z.jpg]

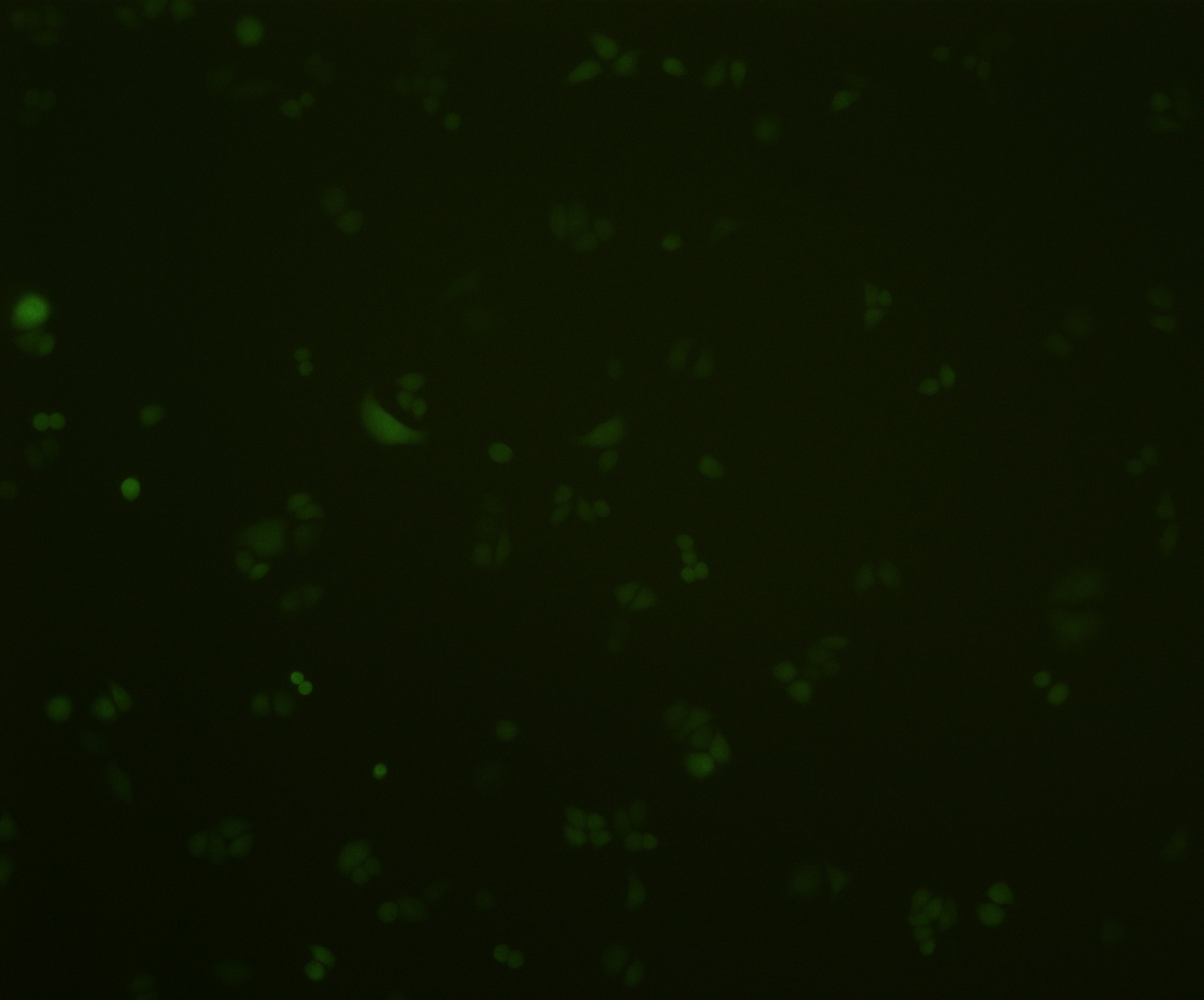

Supplement: S6 File — (ZIP) [file pone.0334639.s006.zip › S 11. File. Original Images. Fig4/S 11. File. Original FIgures. Fig.4/4a/Bel-7402/BEI-7402 Overexpression -y.jpg]

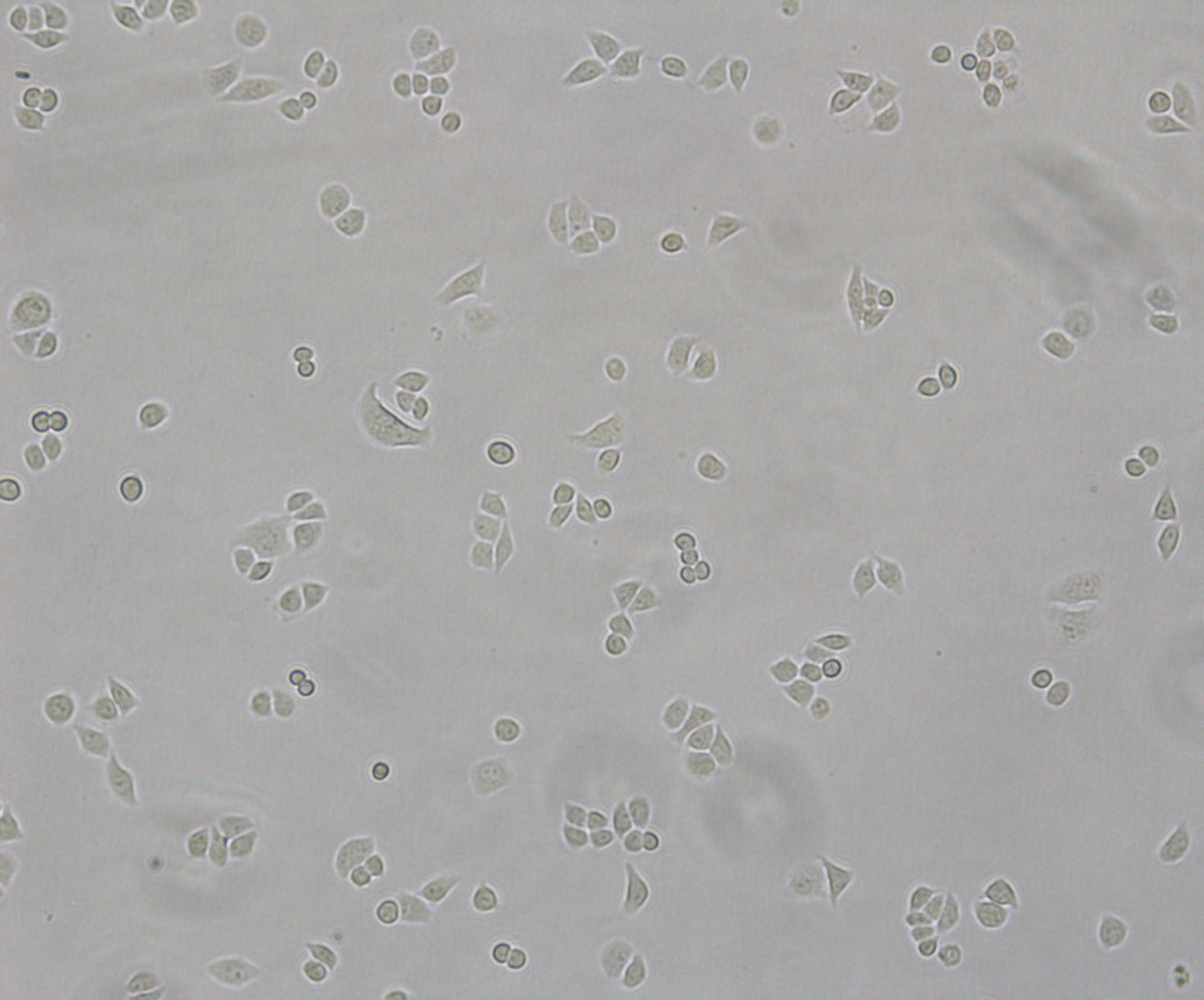

Supplement: S6 File — (ZIP) [file pone.0334639.s006.zip › S 11. File. Original Images. Fig4/S 11. File. Original FIgures. Fig.4/4a/Bel-7402/BEI-7402 Overexpression--z.jpg]

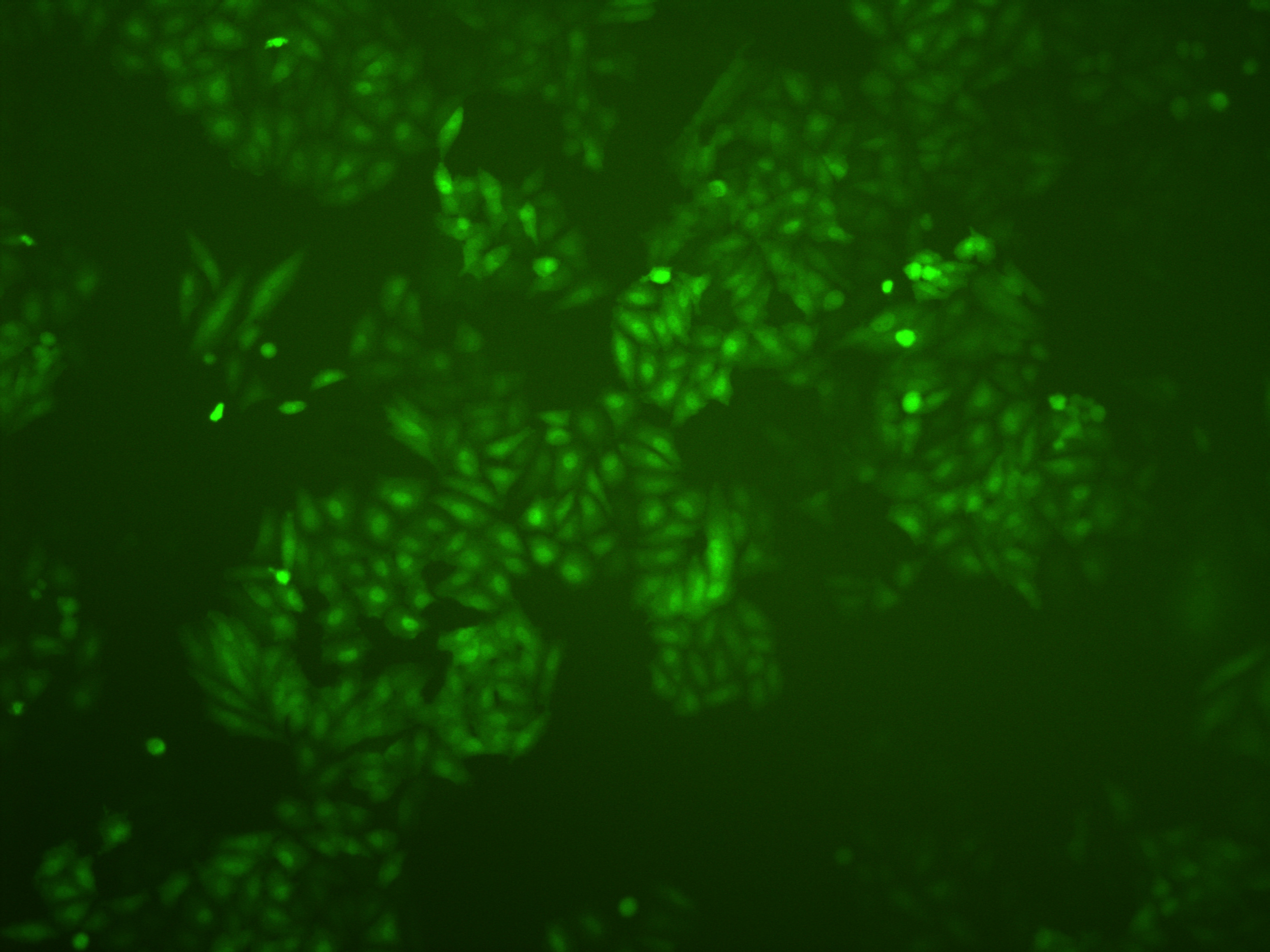

Supplement: S6 File — (ZIP) [file pone.0334639.s006.zip › S 11. File. Original Images. Fig4/S 11. File. Original FIgures. Fig.4/4a/hepg2/hepg2 Overexpression-y.jpg]

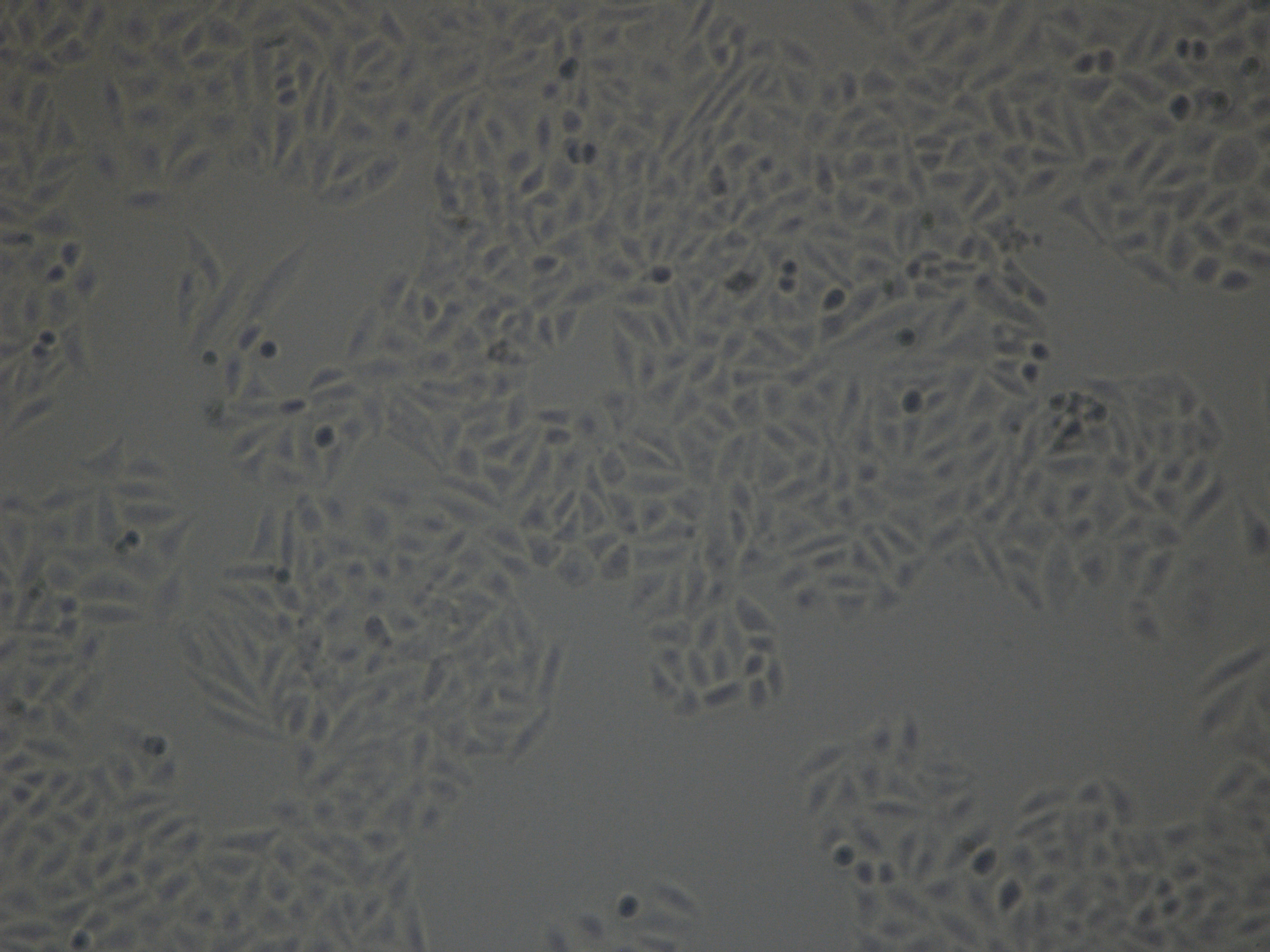

Supplement: S6 File — (ZIP) [file pone.0334639.s006.zip › S 11. File. Original Images. Fig4/S 11. File. Original FIgures. Fig.4/4a/hepg2/hepg2 Overexpression-z.jpg]

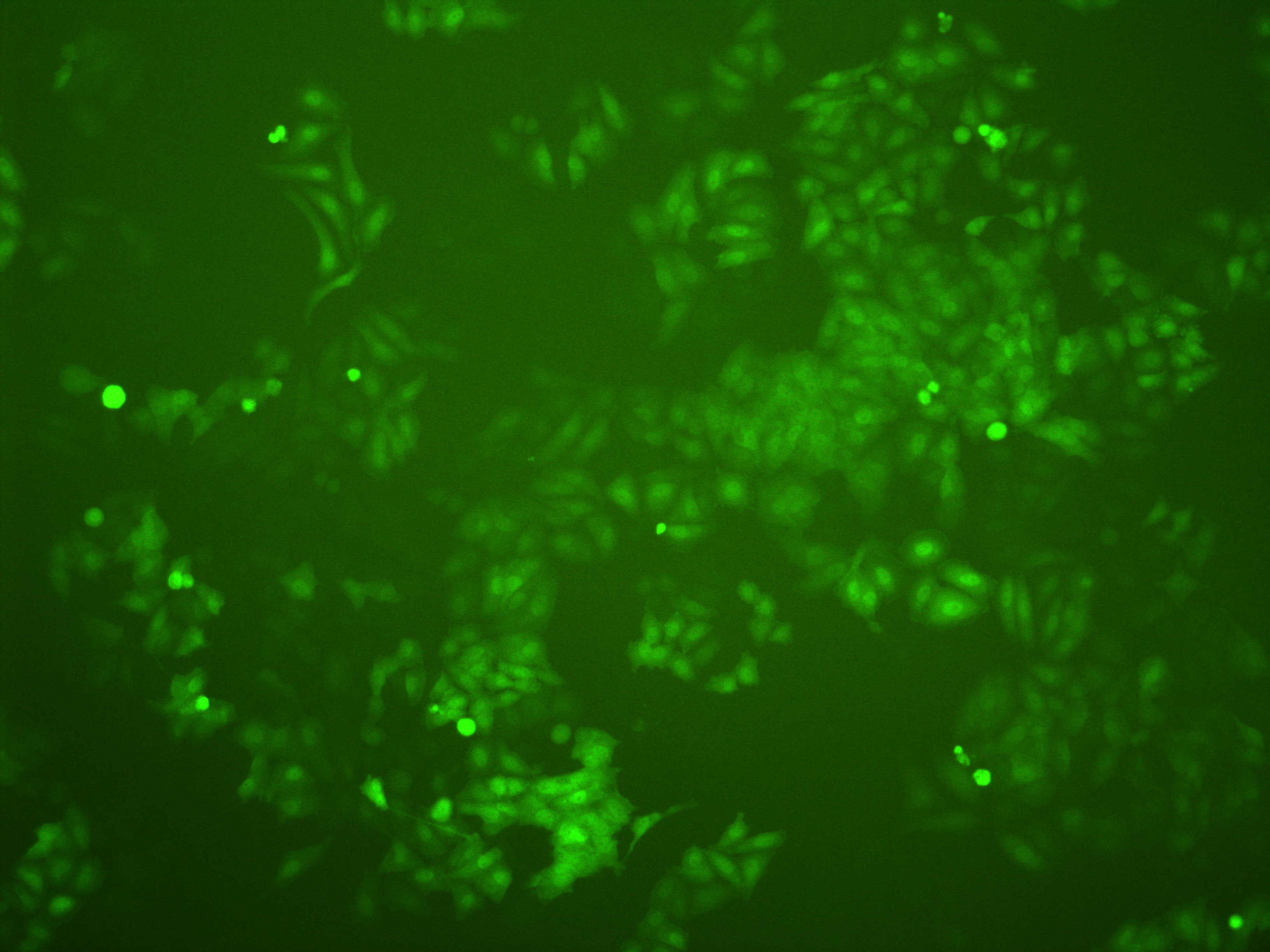

Supplement: S6 File — (ZIP) [file pone.0334639.s006.zip › S 11. File. Original Images. Fig4/S 11. File. Original FIgures. Fig.4/4a/hepg2/hepg2 mock--y.jpg]

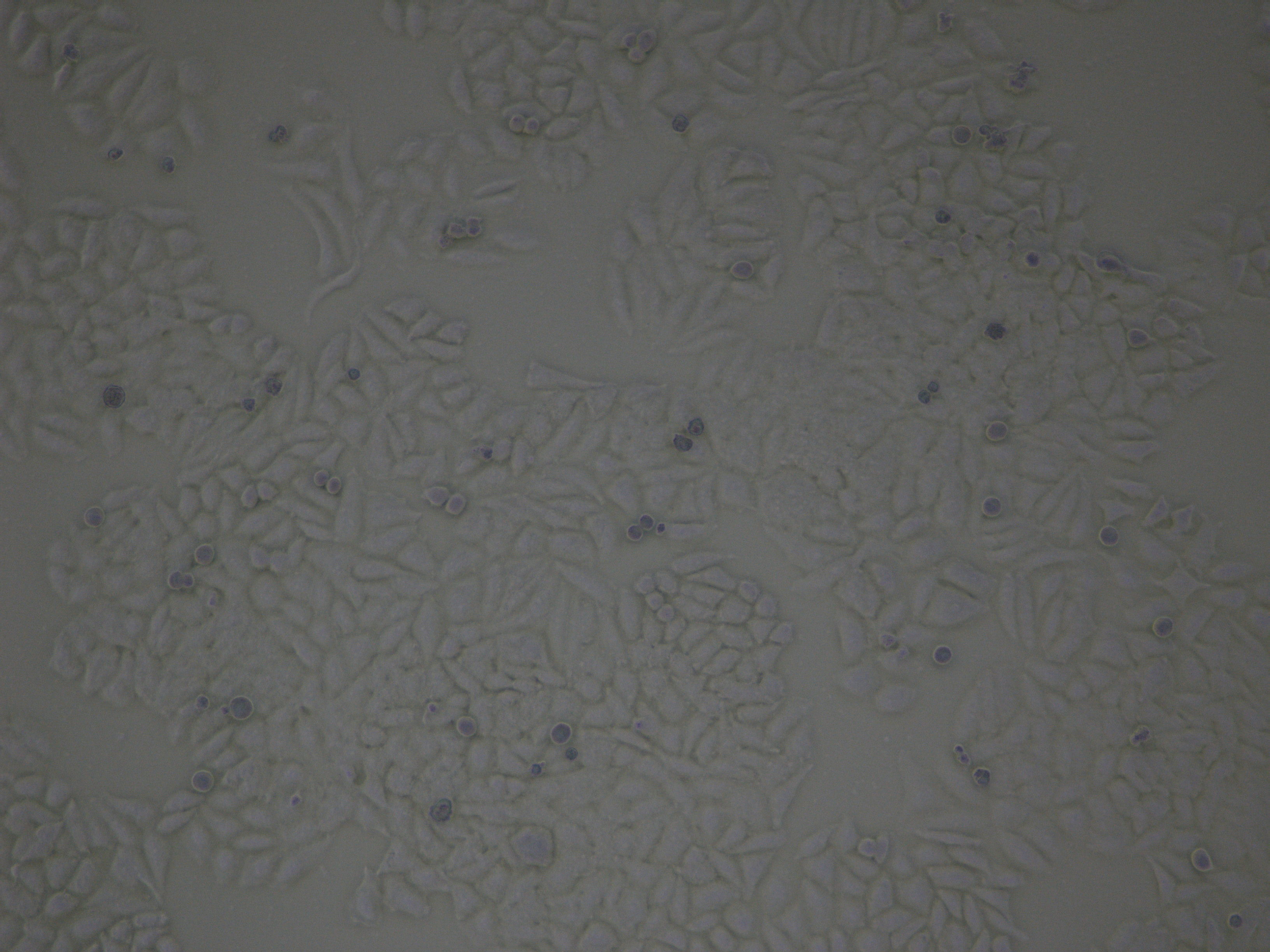

Supplement: S6 File — (ZIP) [file pone.0334639.s006.zip › S 11. File. Original Images. Fig4/S 11. File. Original FIgures. Fig.4/4a/hepg2/hepg2 mock-z.jpg]

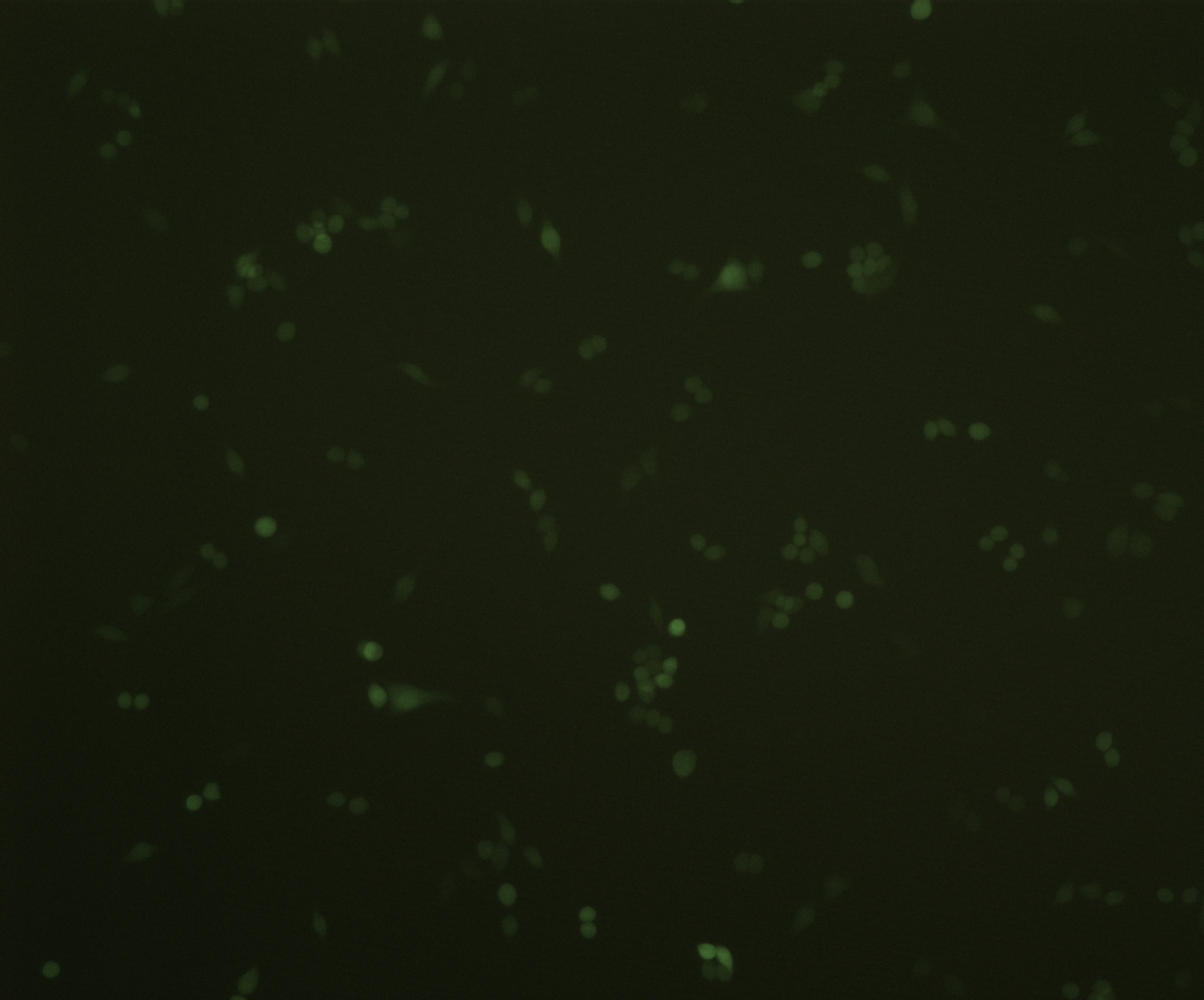

Supplement: S6 File — (ZIP) [file pone.0334639.s006.zip › S 11. File. Original Images. Fig4/S 11. File. Original FIgures. Fig.4/4a/smmc-7721/Smmc7721 MOCK-y.jpg]

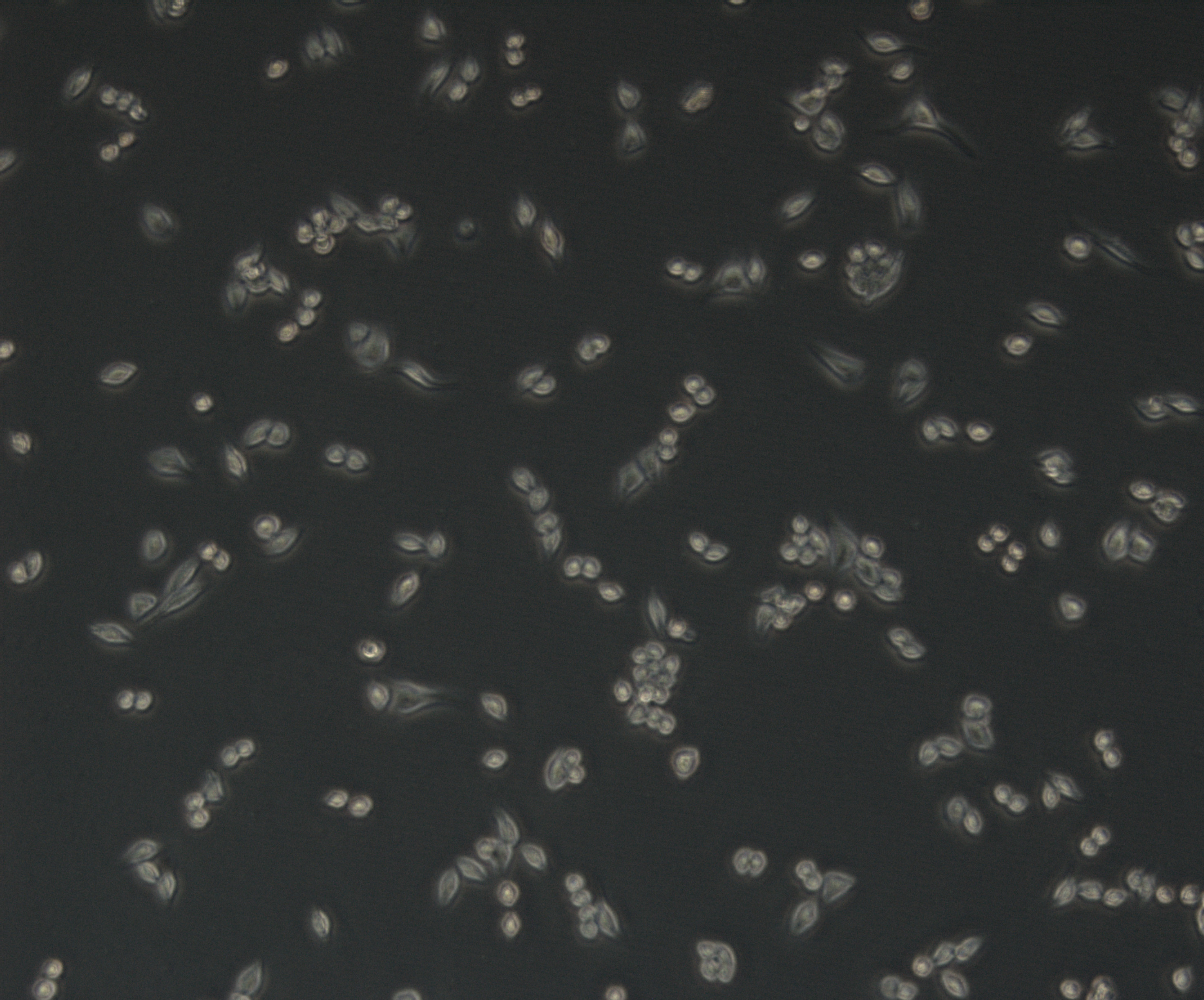

Supplement: S6 File — (ZIP) [file pone.0334639.s006.zip › S 11. File. Original Images. Fig4/S 11. File. Original FIgures. Fig.4/4a/smmc-7721/Smmc7721 MOCK-z.jpg]

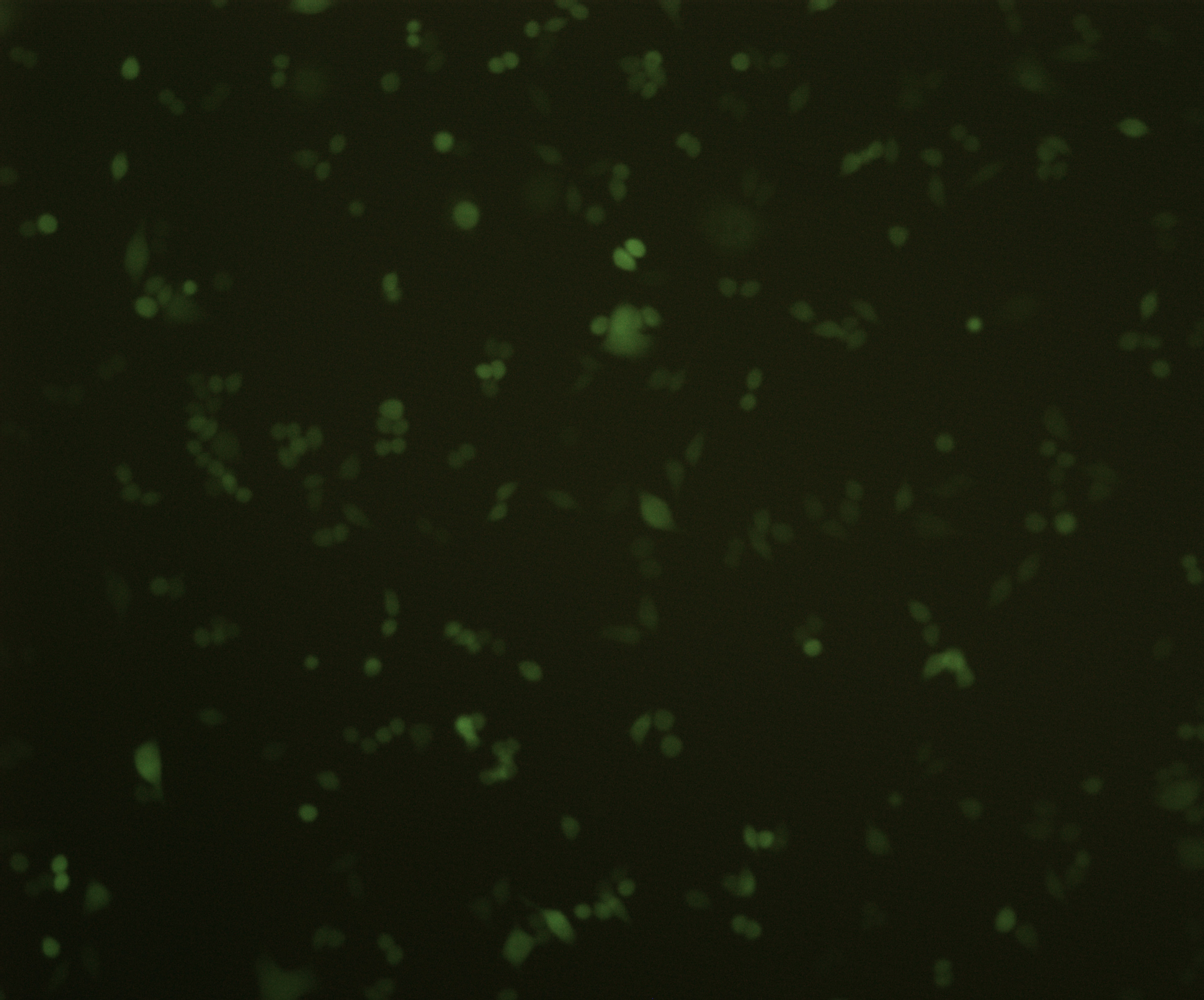

Supplement: S6 File — (ZIP) [file pone.0334639.s006.zip › S 11. File. Original Images. Fig4/S 11. File. Original FIgures. Fig.4/4a/smmc-7721/Smmc7721 Overexpression-y.jpg]

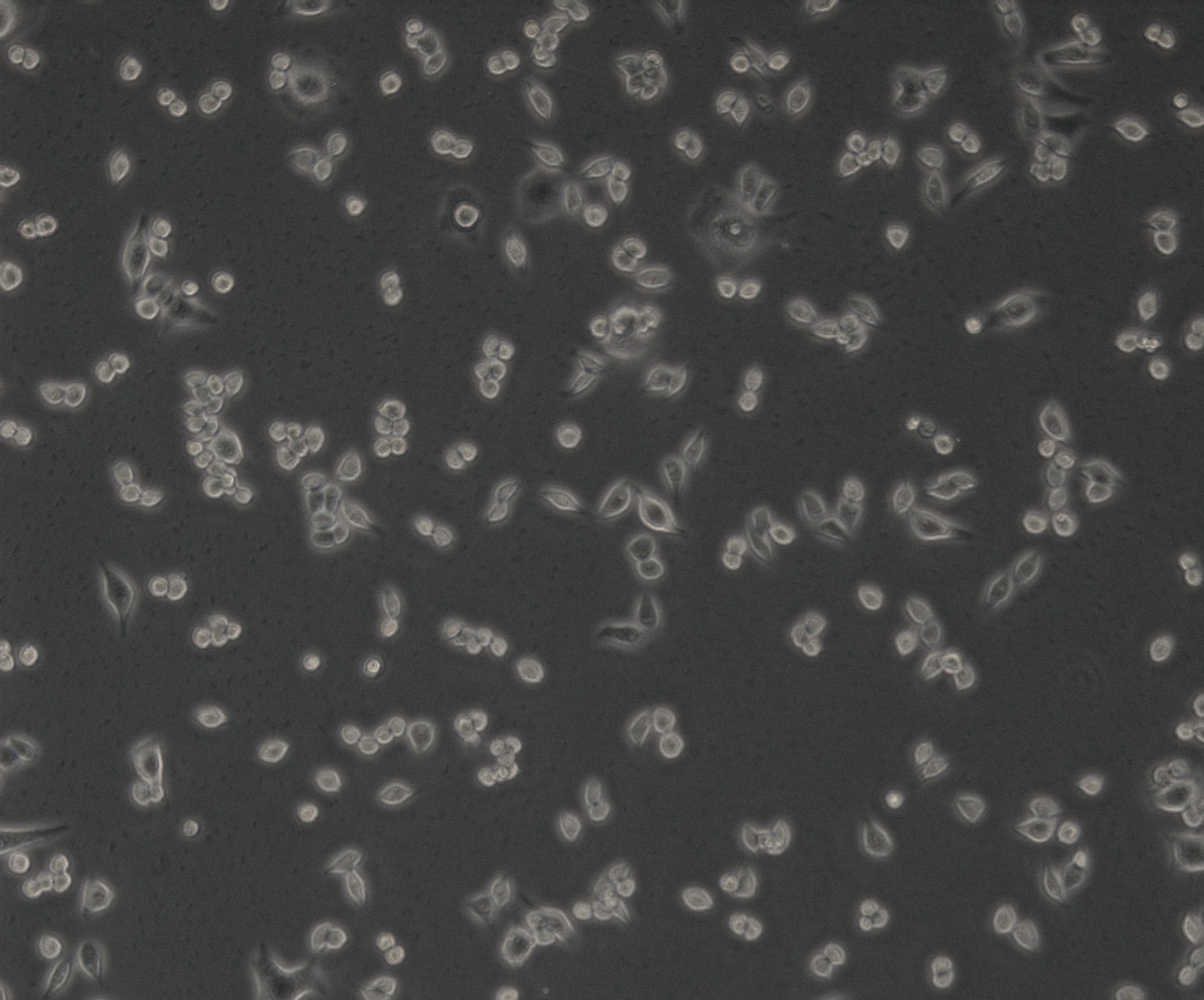

Supplement: S6 File — (ZIP) [file pone.0334639.s006.zip › S 11. File. Original Images. Fig4/S 11. File. Original FIgures. Fig.4/4a/smmc-7721/Smmc7721 Overexpression-z.jpg]

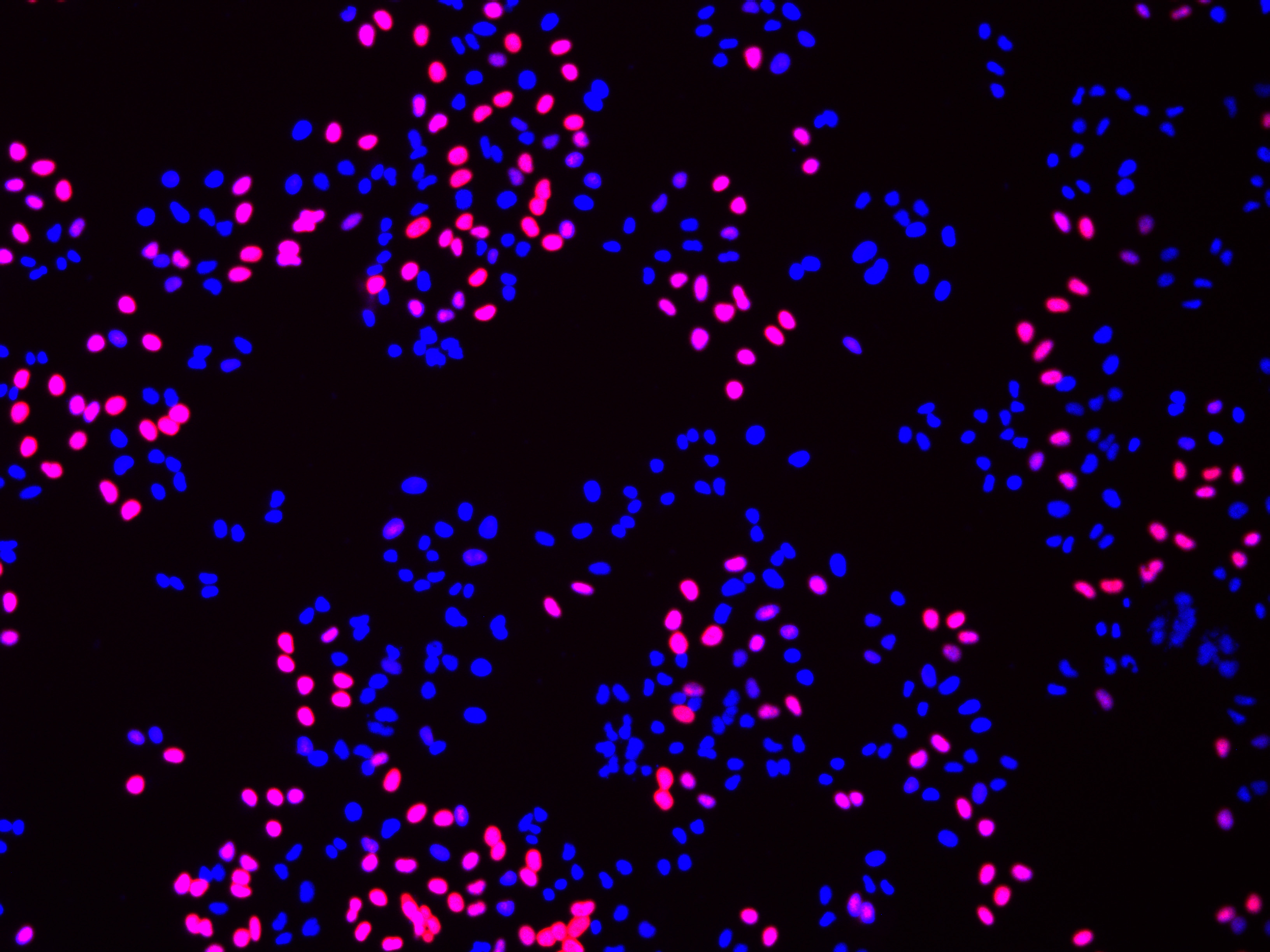

Supplement: S6 File — (ZIP) [file pone.0334639.s006.zip › S 11. File. Original Images. Fig4/S 11. File. Original FIgures. Fig.4/4e/BEL-7402 MOCK merge.jpg]

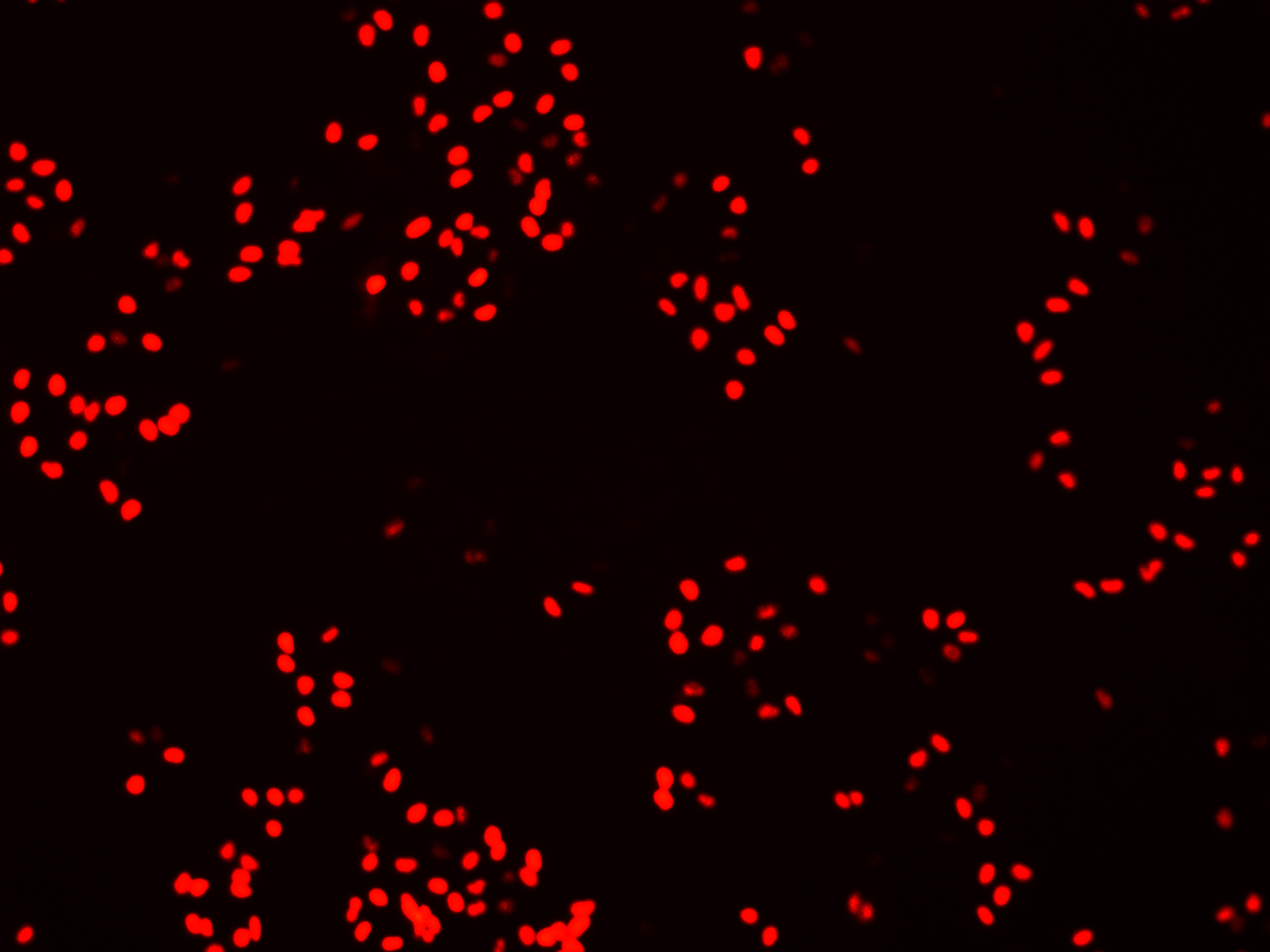

Supplement: S6 File — (ZIP) [file pone.0334639.s006.zip › S 11. File. Original Images. Fig4/S 11. File. Original FIgures. Fig.4/4e/BEL-7402 MOCK --edu.jpg]

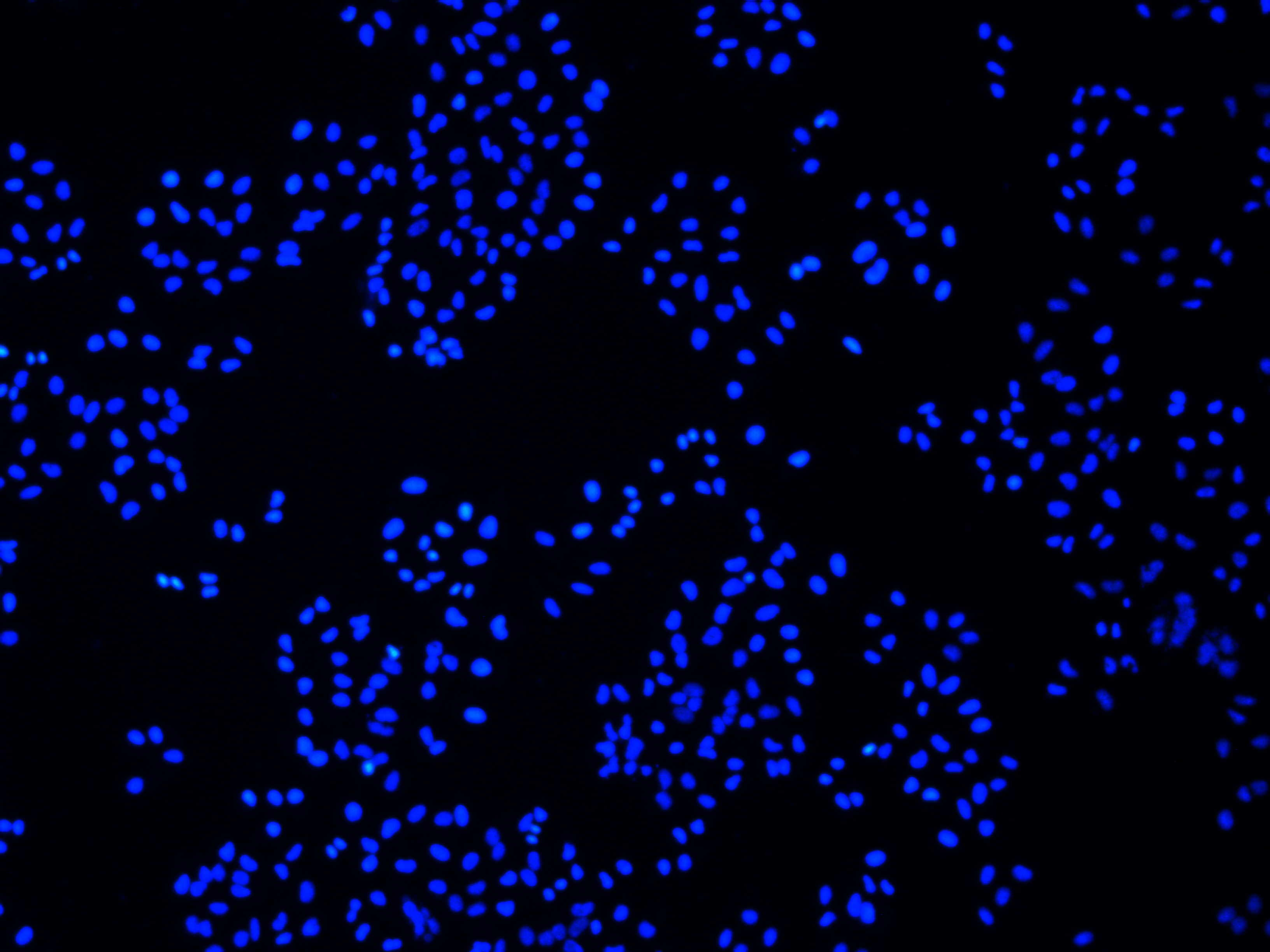

Supplement: S6 File — (ZIP) [file pone.0334639.s006.zip › S 11. File. Original Images. Fig4/S 11. File. Original FIgures. Fig.4/4e/BEL-7402 MOCK Hoechst33342.jpg]

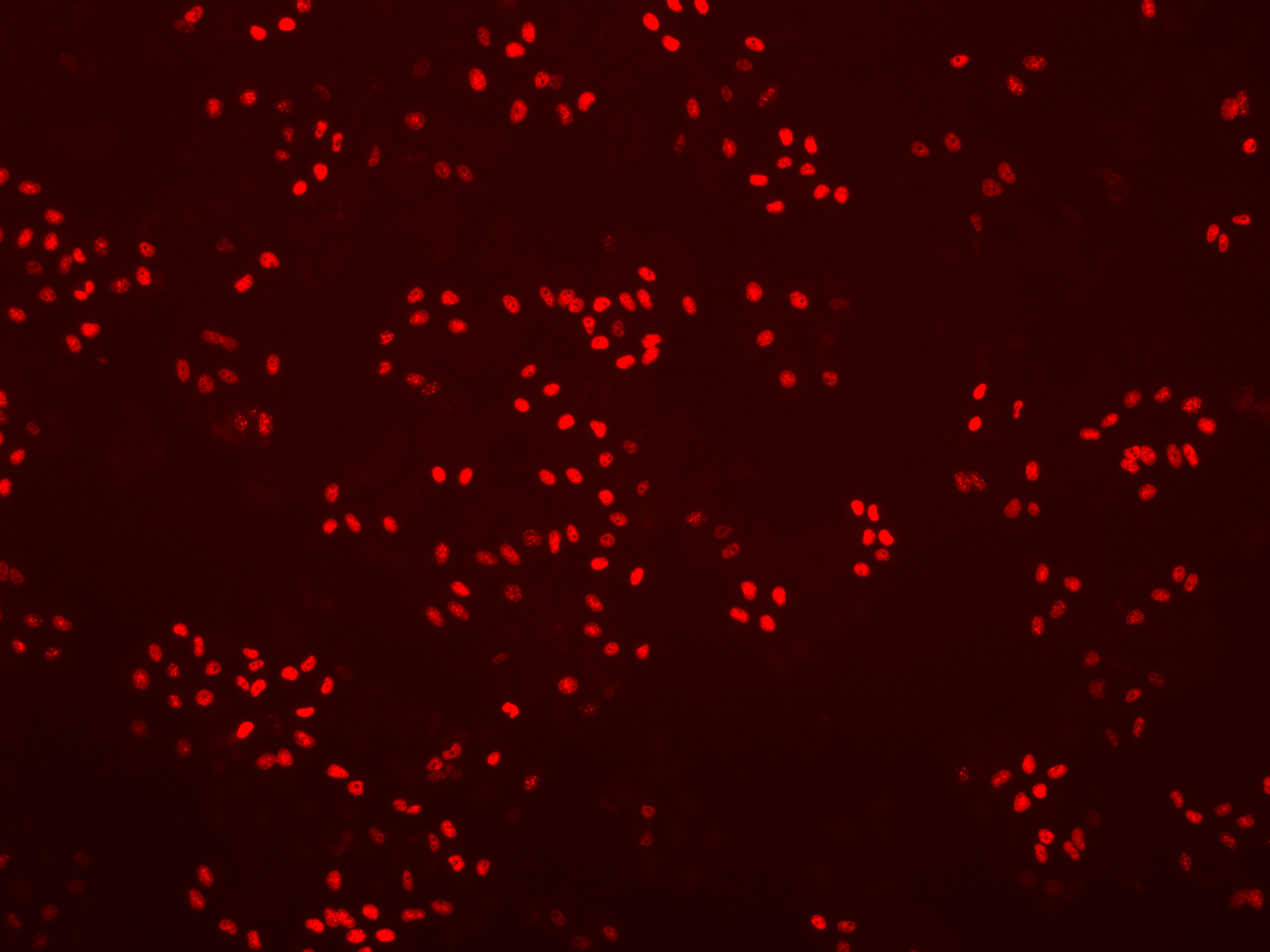

Supplement: S6 File — (ZIP) [file pone.0334639.s006.zip › S 11. File. Original Images. Fig4/S 11. File. Original FIgures. Fig.4/4e/BEL-7402 Overexpression-edu.jpg]

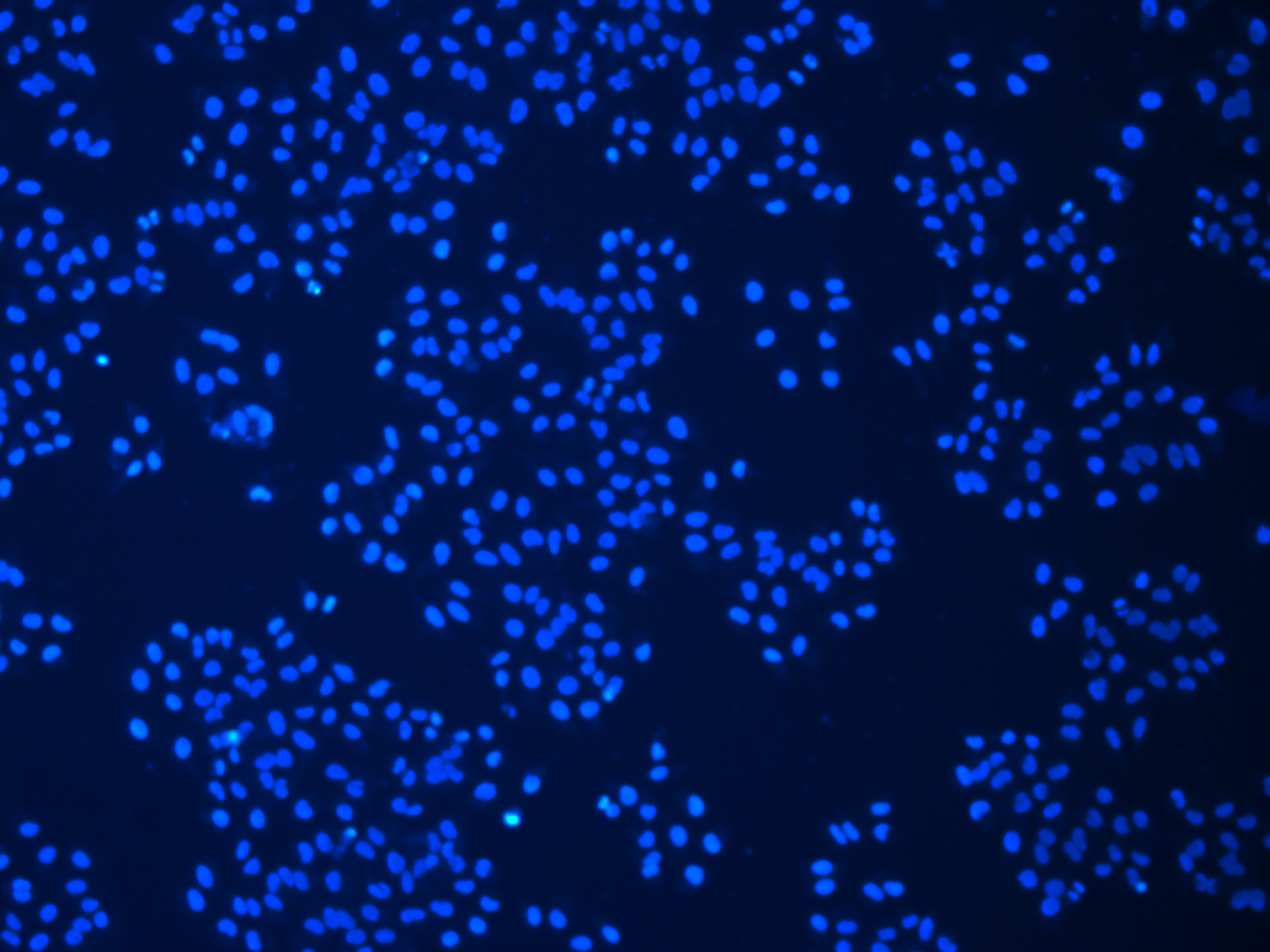

Supplement: S6 File — (ZIP) [file pone.0334639.s006.zip › S 11. File. Original Images. Fig4/S 11. File. Original FIgures. Fig.4/4e/BEL-7402 Overexpression-Hoechst33342.jpg]

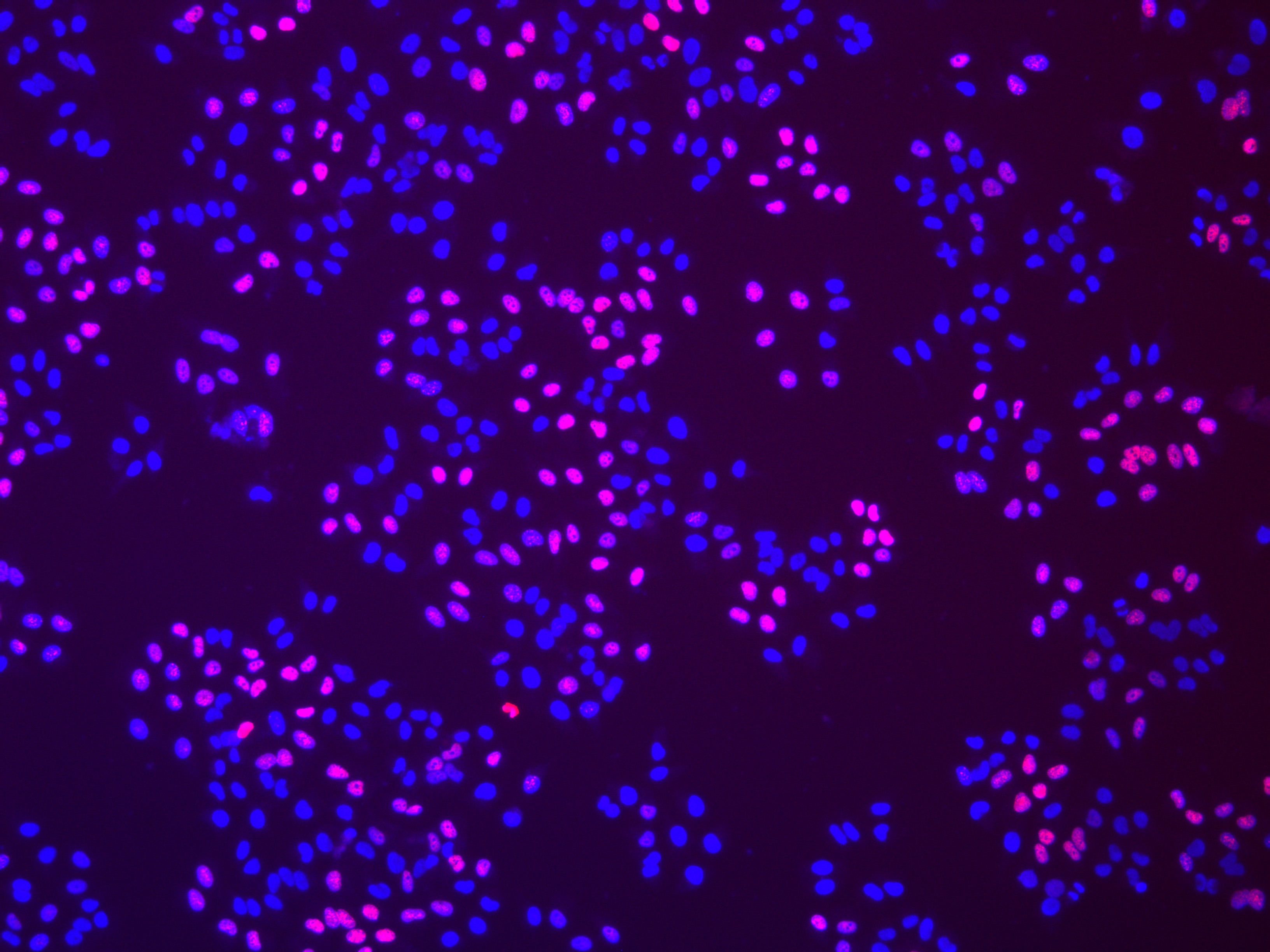

Supplement: S6 File — (ZIP) [file pone.0334639.s006.zip › S 11. File. Original Images. Fig4/S 11. File. Original FIgures. Fig.4/4e/BEL-7402 Overexpression-merge.jpg]

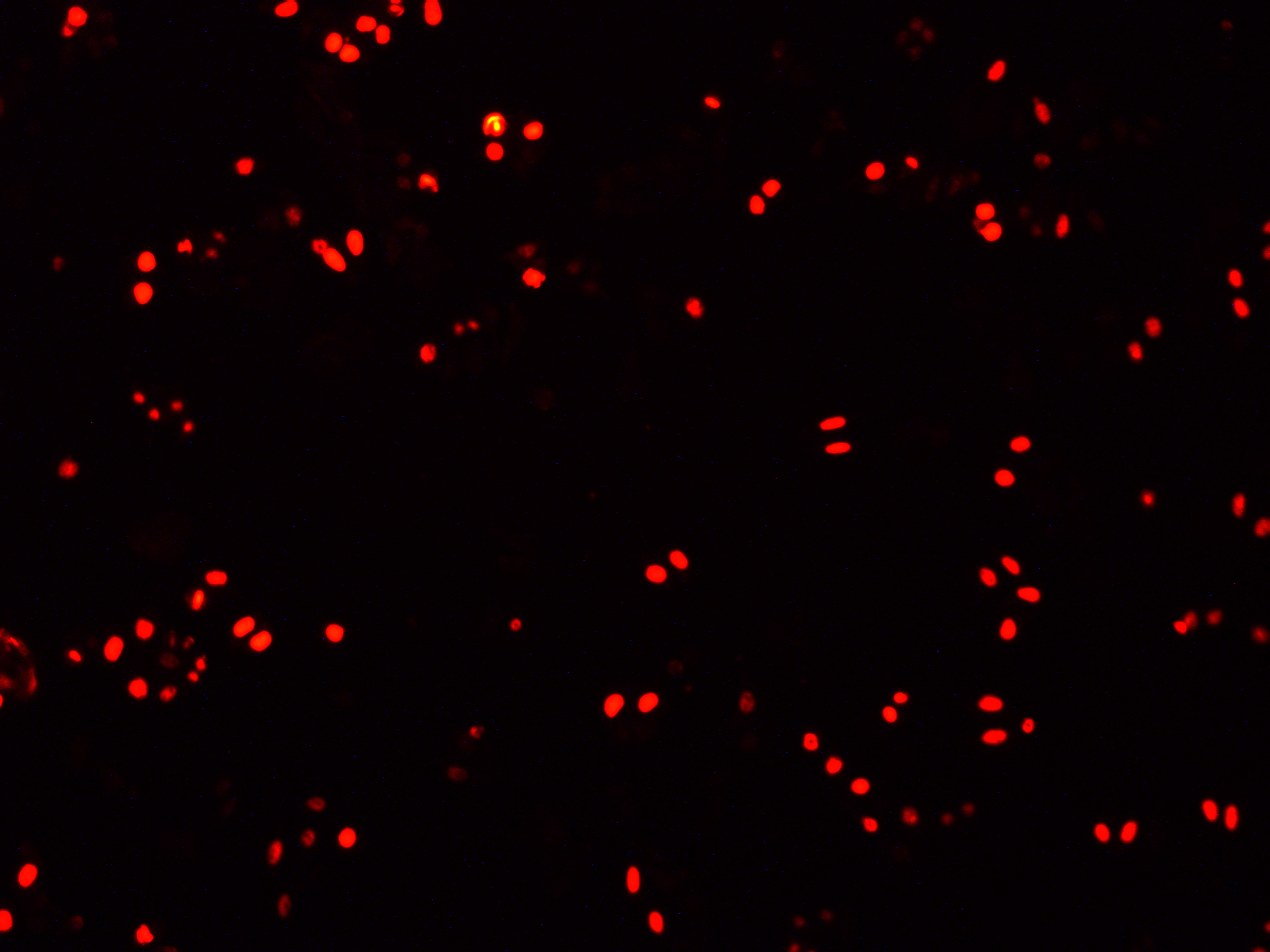

Supplement: S6 File — (ZIP) [file pone.0334639.s006.zip › S 11. File. Original Images. Fig4/S 11. File. Original FIgures. Fig.4/4f/hepG2 Mock-edu.jpg]

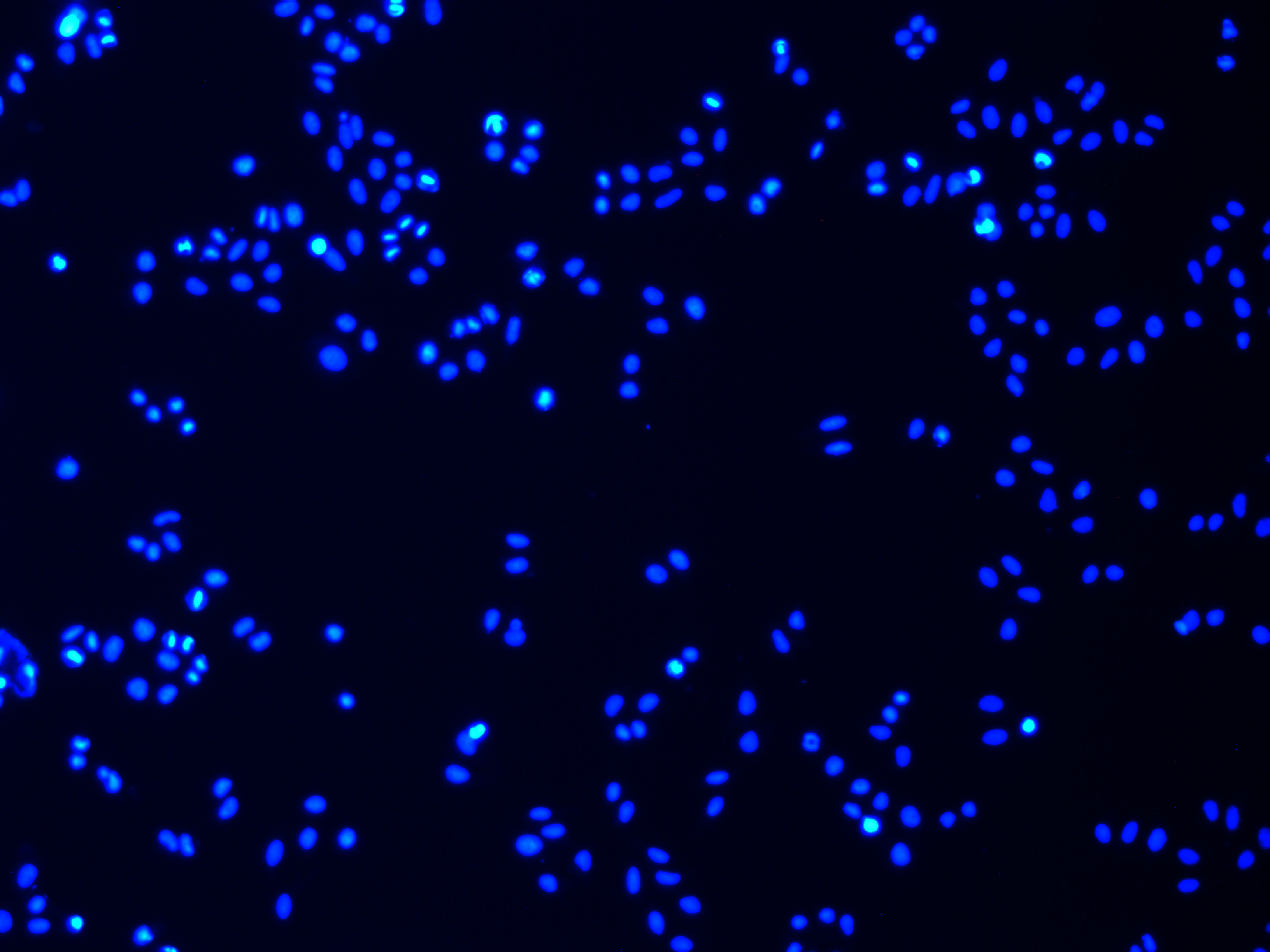

Supplement: S6 File — (ZIP) [file pone.0334639.s006.zip › S 11. File. Original Images. Fig4/S 11. File. Original FIgures. Fig.4/4f/hepG2 Mock-Hoechst33342.jpg]

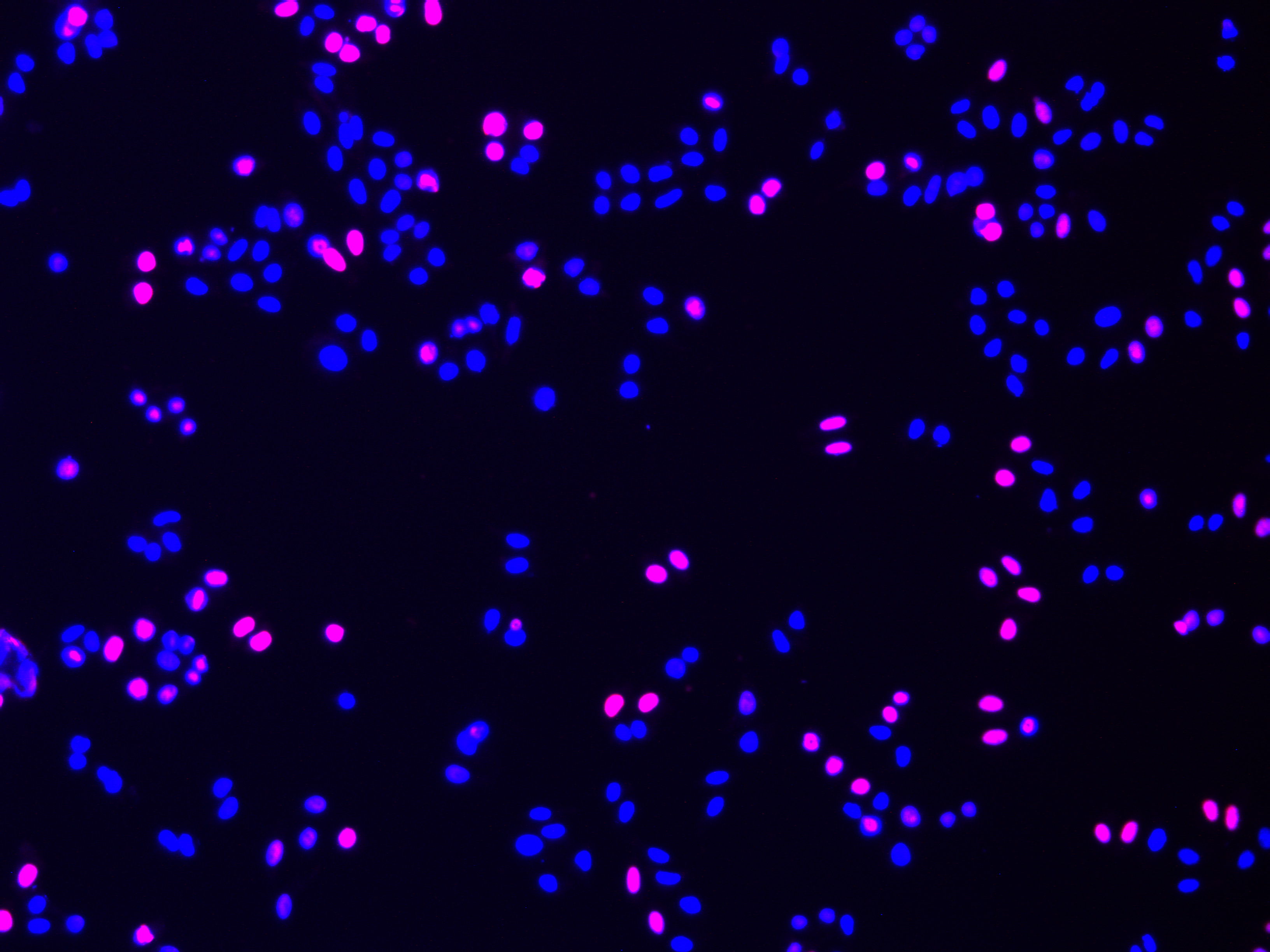

Supplement: S6 File — (ZIP) [file pone.0334639.s006.zip › S 11. File. Original Images. Fig4/S 11. File. Original FIgures. Fig.4/4f/hepG2 Mock-merge.jpg]

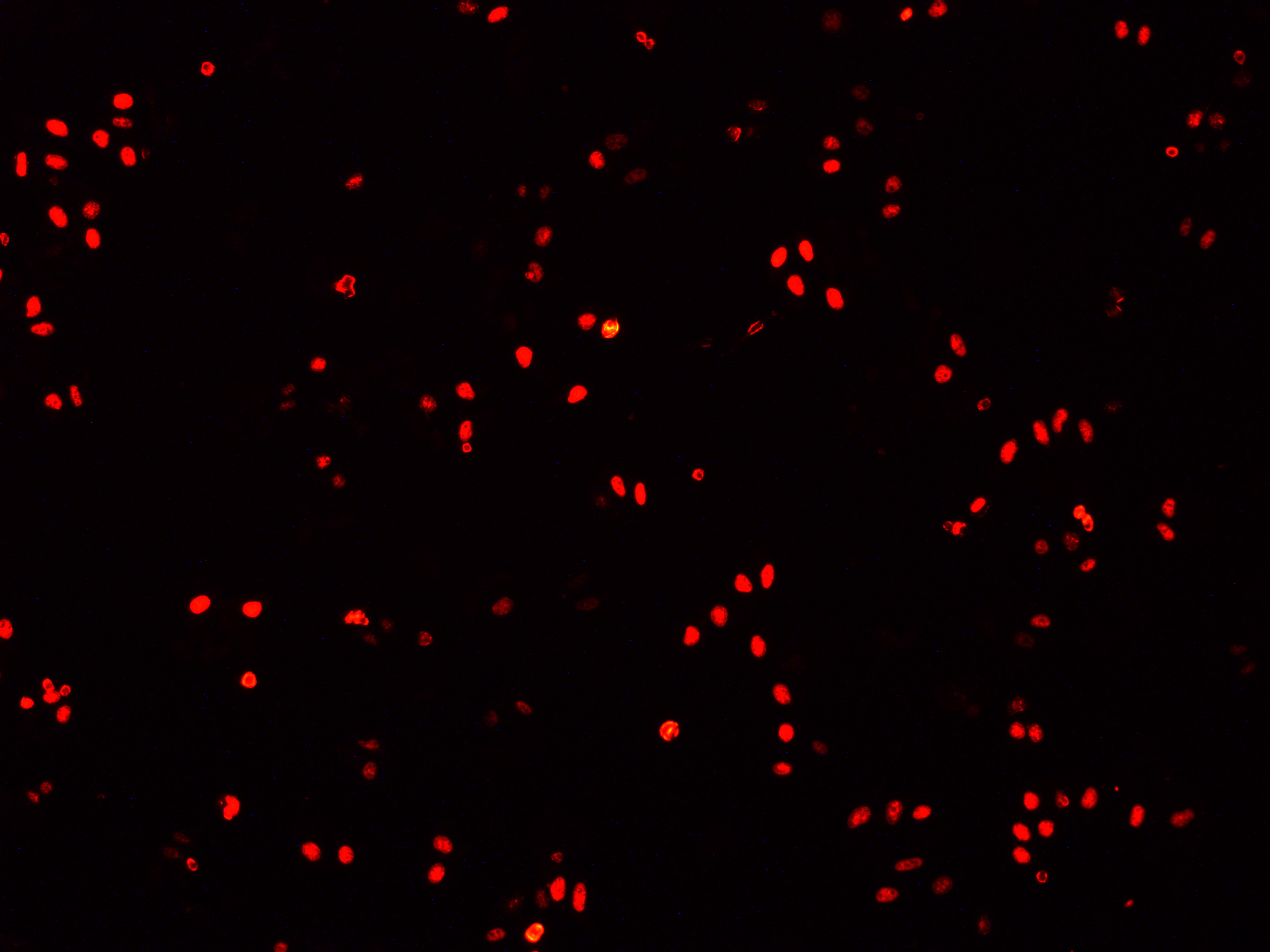

Supplement: S6 File — (ZIP) [file pone.0334639.s006.zip › S 11. File. Original Images. Fig4/S 11. File. Original FIgures. Fig.4/4f/hepG2 Overexpression-edu.jpg]

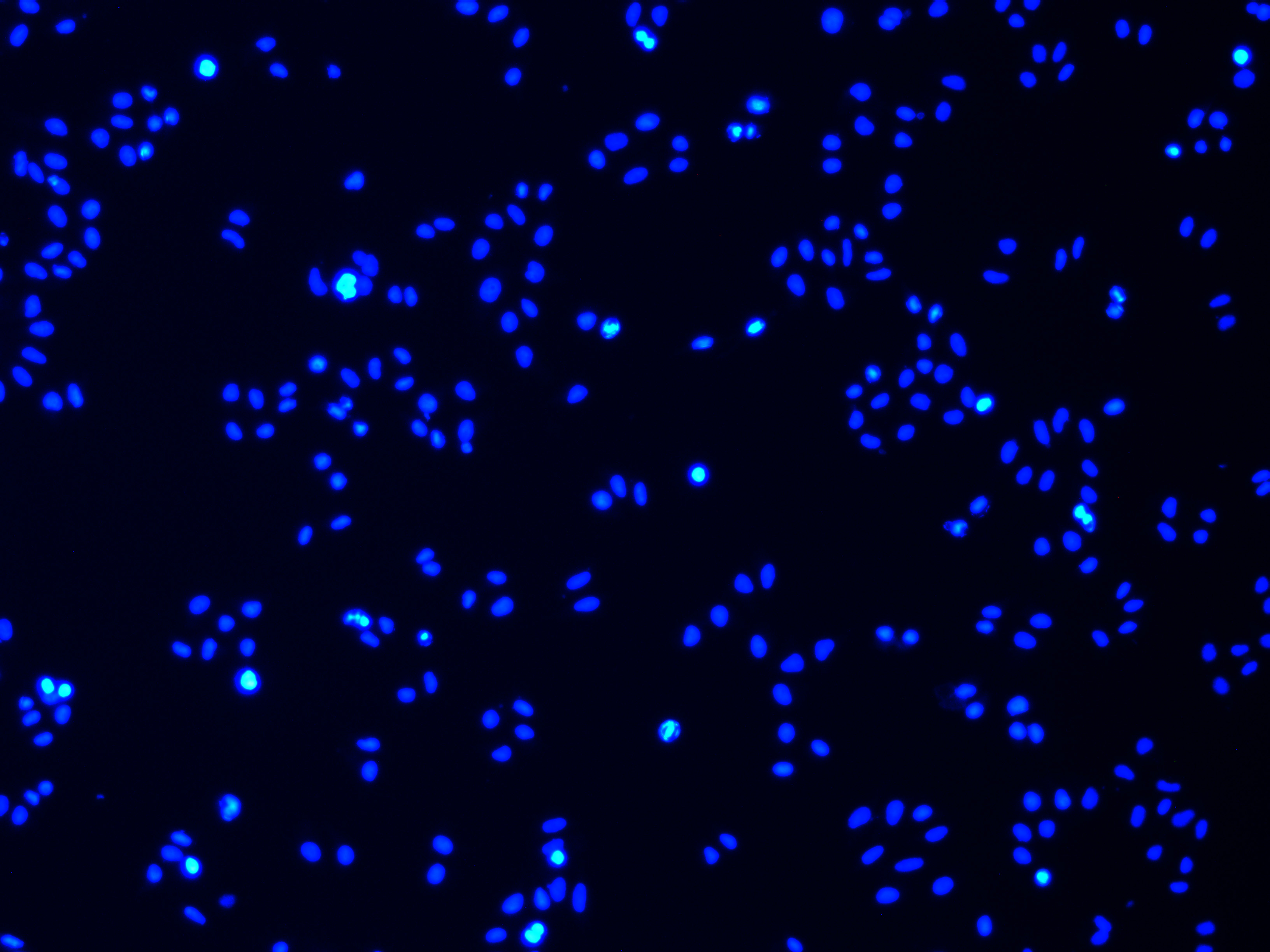

Supplement: S6 File — (ZIP) [file pone.0334639.s006.zip › S 11. File. Original Images. Fig4/S 11. File. Original FIgures. Fig.4/4f/hepG2 Overexpression-Hoechst33342.jpg]

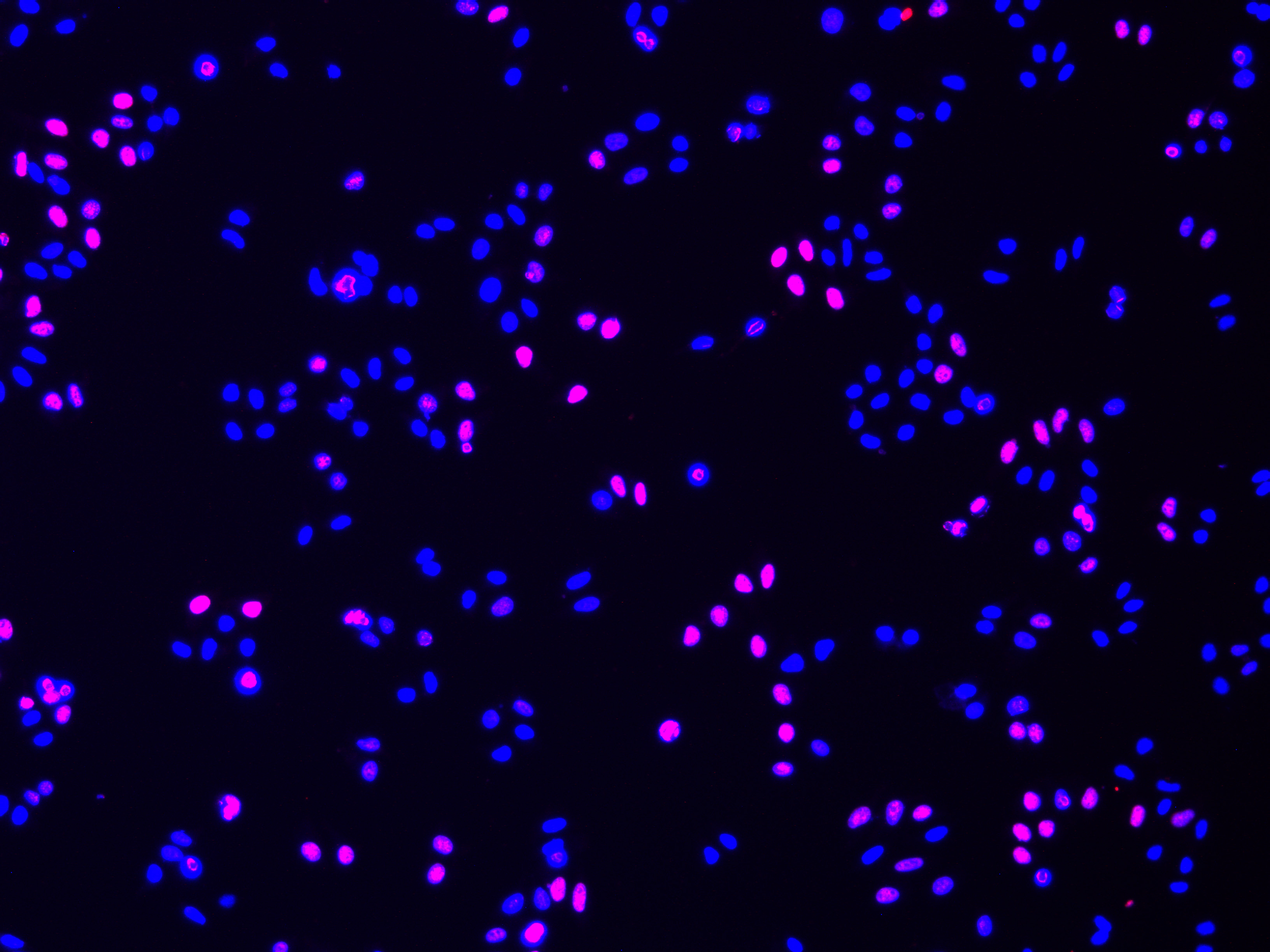

Supplement: S6 File — (ZIP) [file pone.0334639.s006.zip › S 11. File. Original Images. Fig4/S 11. File. Original FIgures. Fig.4/4f/hepG2 Overexpression-merge.jpg]

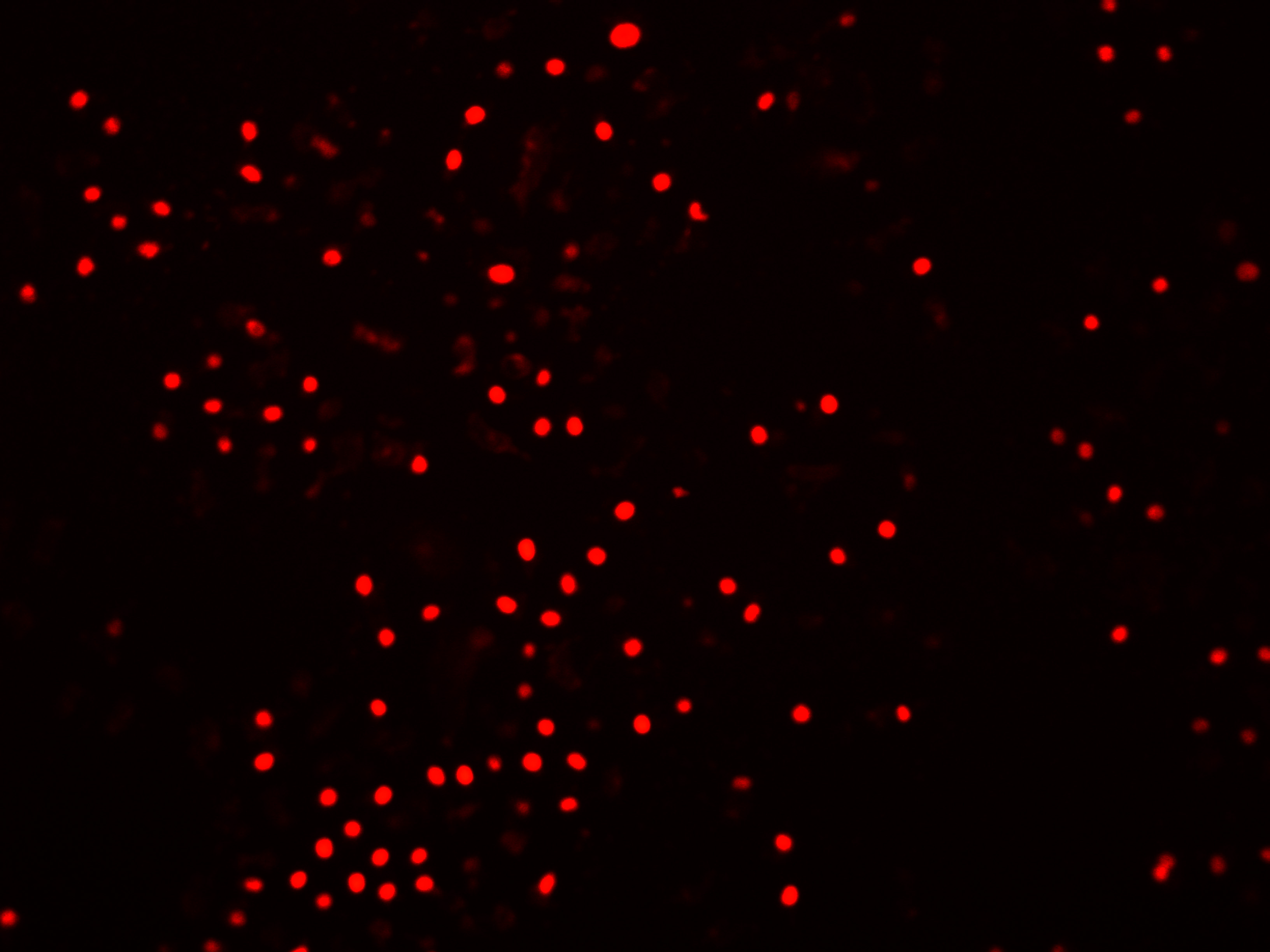

Supplement: S6 File — (ZIP) [file pone.0334639.s006.zip › S 11. File. Original Images. Fig4/S 11. File. Original FIgures. Fig.4/4g/SMMC-7721 Mock-EDU.jpg]

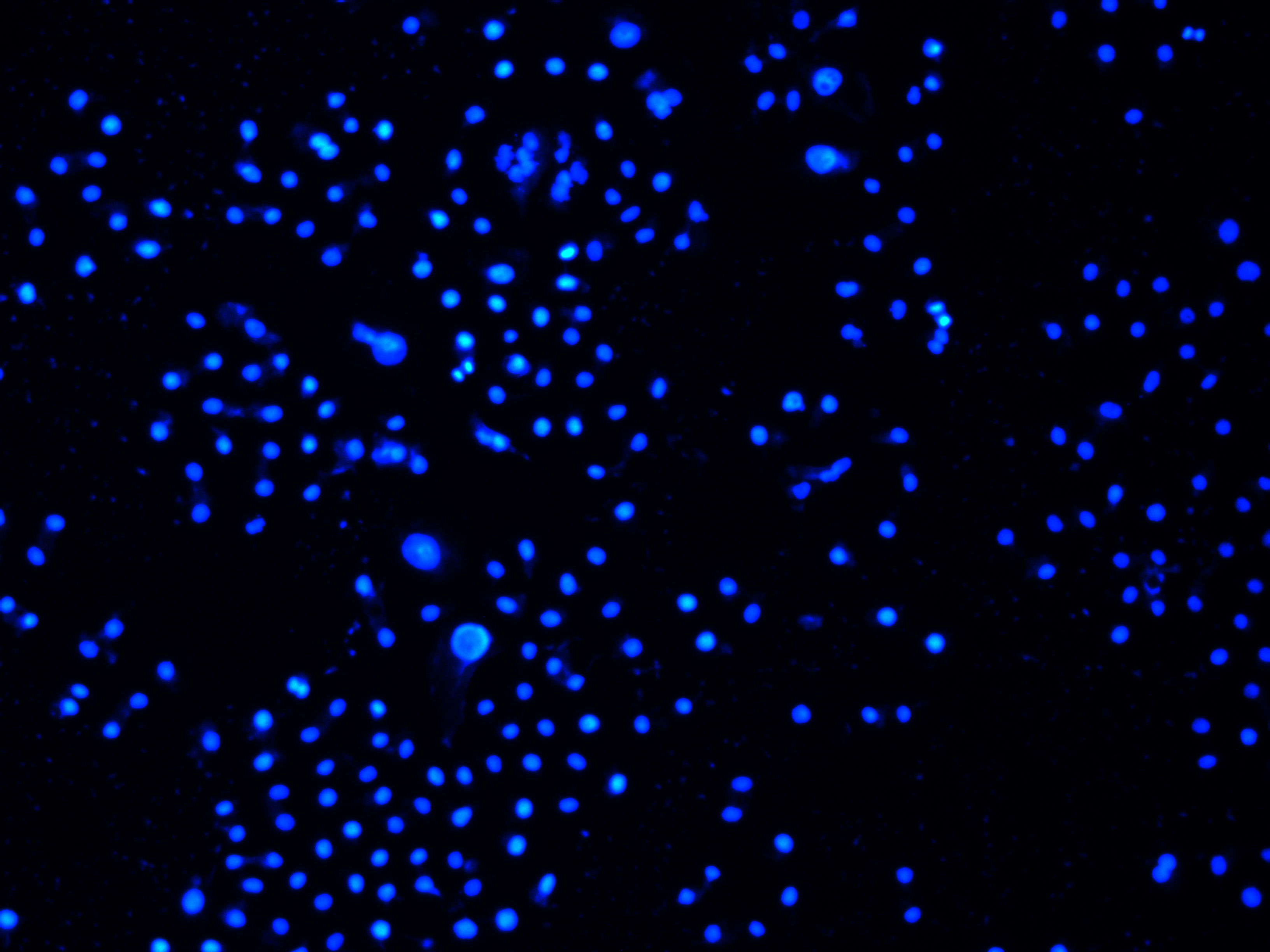

Supplement: S6 File — (ZIP) [file pone.0334639.s006.zip › S 11. File. Original Images. Fig4/S 11. File. Original FIgures. Fig.4/4g/SMMC-7721 Mock-Hoechst33342.jpg]

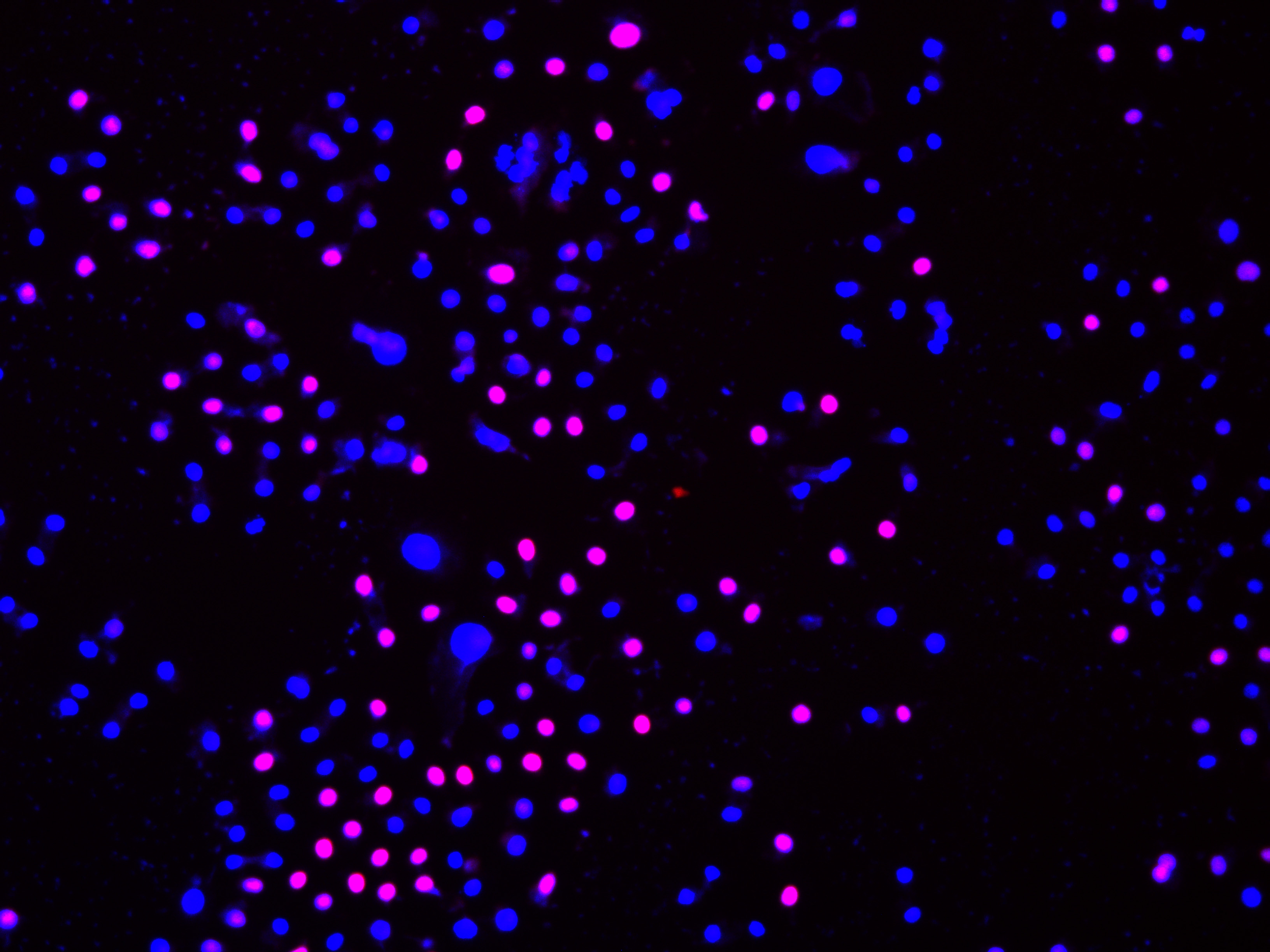

Supplement: S6 File — (ZIP) [file pone.0334639.s006.zip › S 11. File. Original Images. Fig4/S 11. File. Original FIgures. Fig.4/4g/SMMC-7721 Mock-MERGE.jpg]

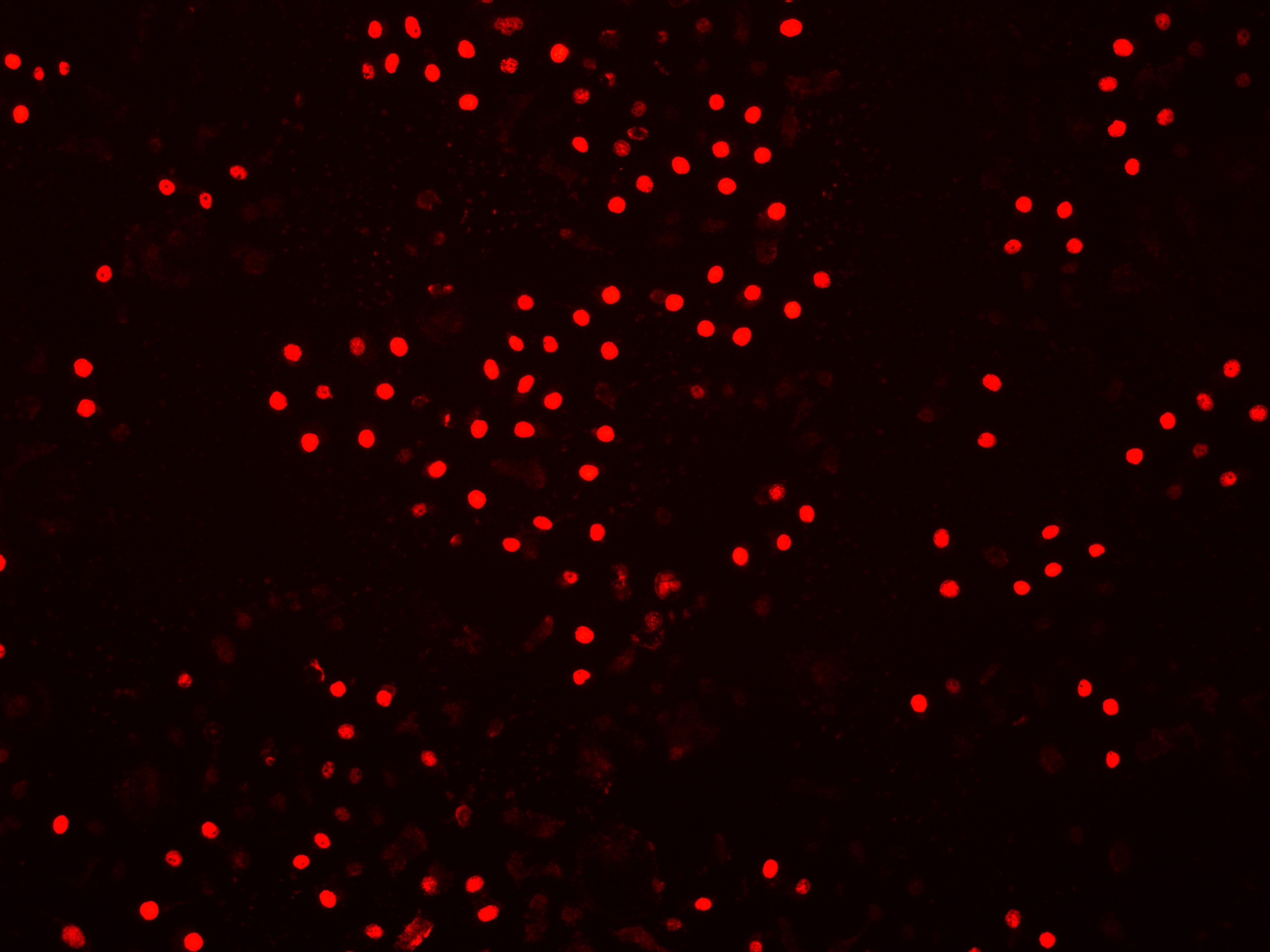

Supplement: S6 File — (ZIP) [file pone.0334639.s006.zip › S 11. File. Original Images. Fig4/S 11. File. Original FIgures. Fig.4/4g/SMMC-7721 Overexpression-edu.jpg]

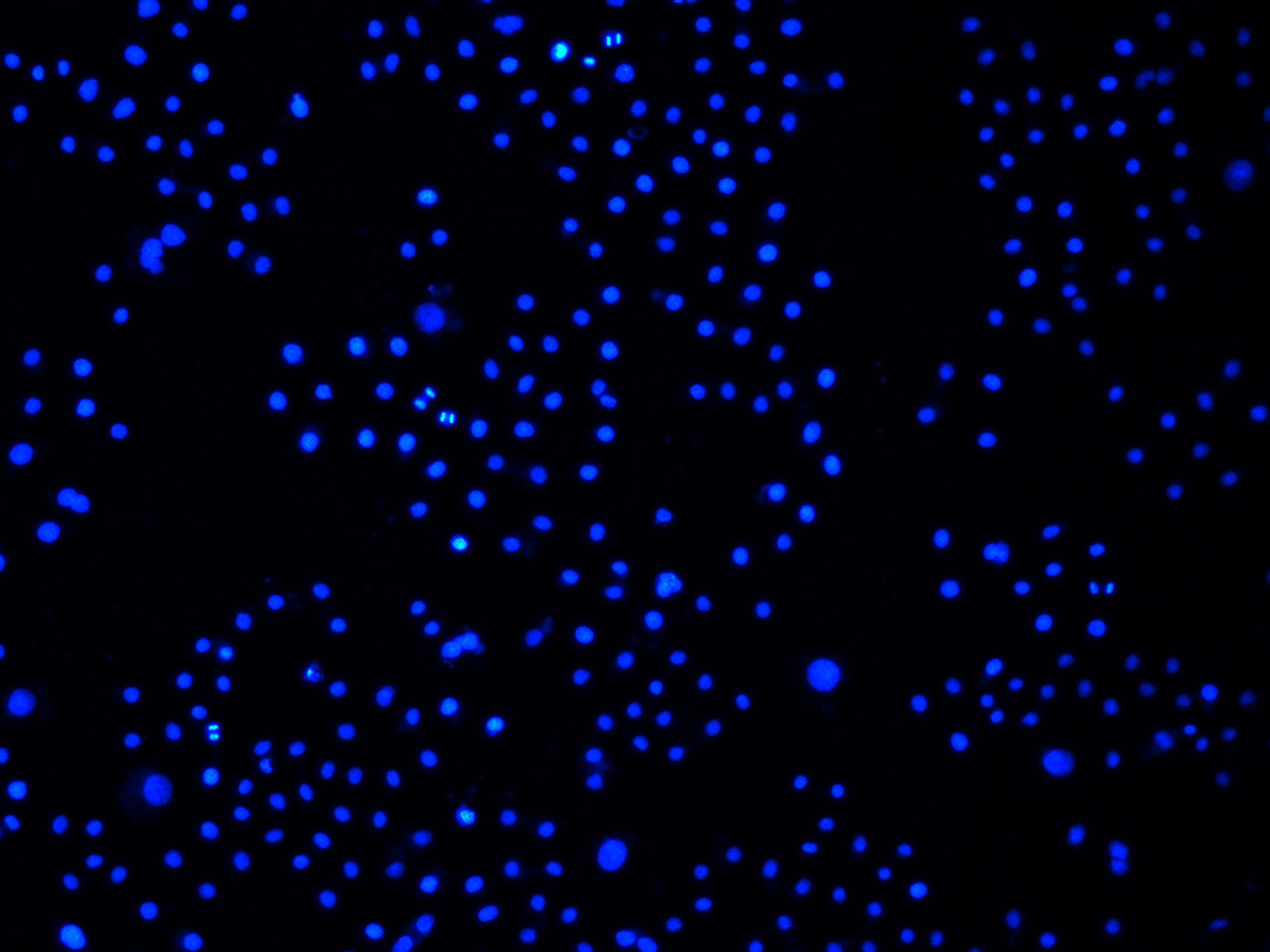

Supplement: S6 File — (ZIP) [file pone.0334639.s006.zip › S 11. File. Original Images. Fig4/S 11. File. Original FIgures. Fig.4/4g/SMMC-7721 Overexpression-Hoechst33342.jpg]

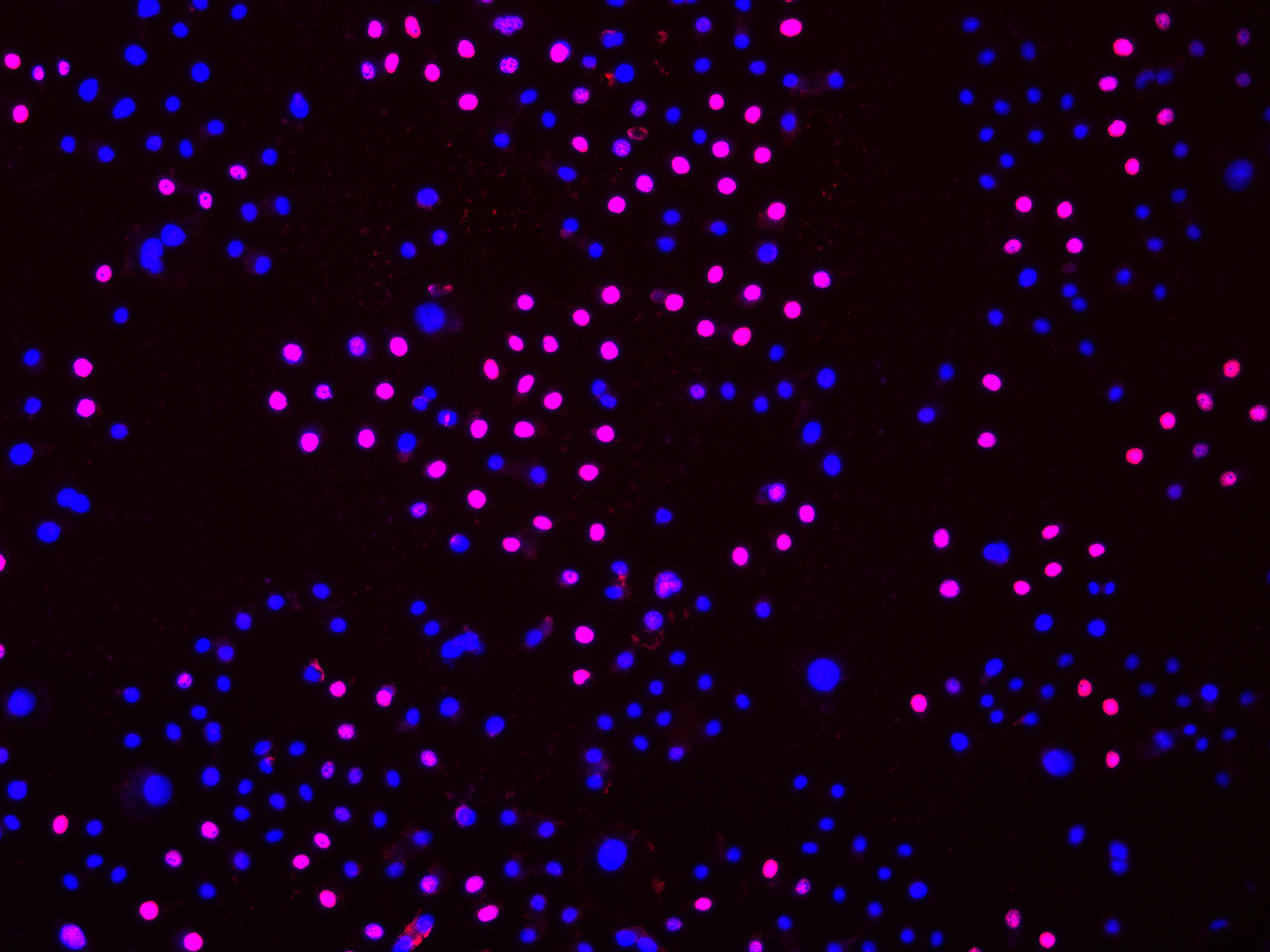

Supplement: S6 File — (ZIP) [file pone.0334639.s006.zip › S 11. File. Original Images. Fig4/S 11. File. Original FIgures. Fig.4/4g/SMMC-7721 Overexpression-merge.jpg]

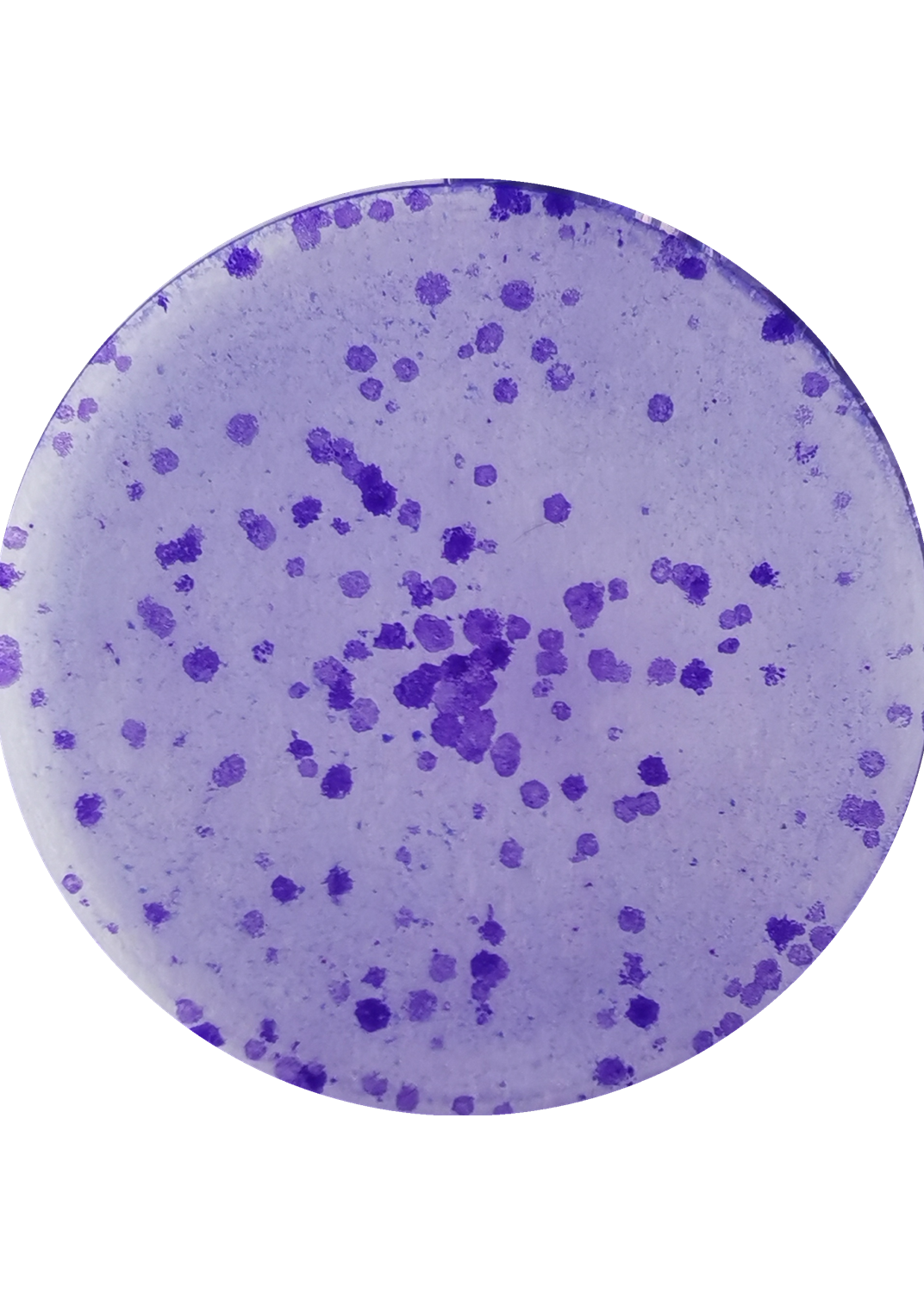

Supplement: S6 File — (ZIP) [file pone.0334639.s006.zip › S 11. File. Original Images. Fig4/S 11. File. Original FIgures. Fig.4/4h/BEL-7402 MOCK.png]

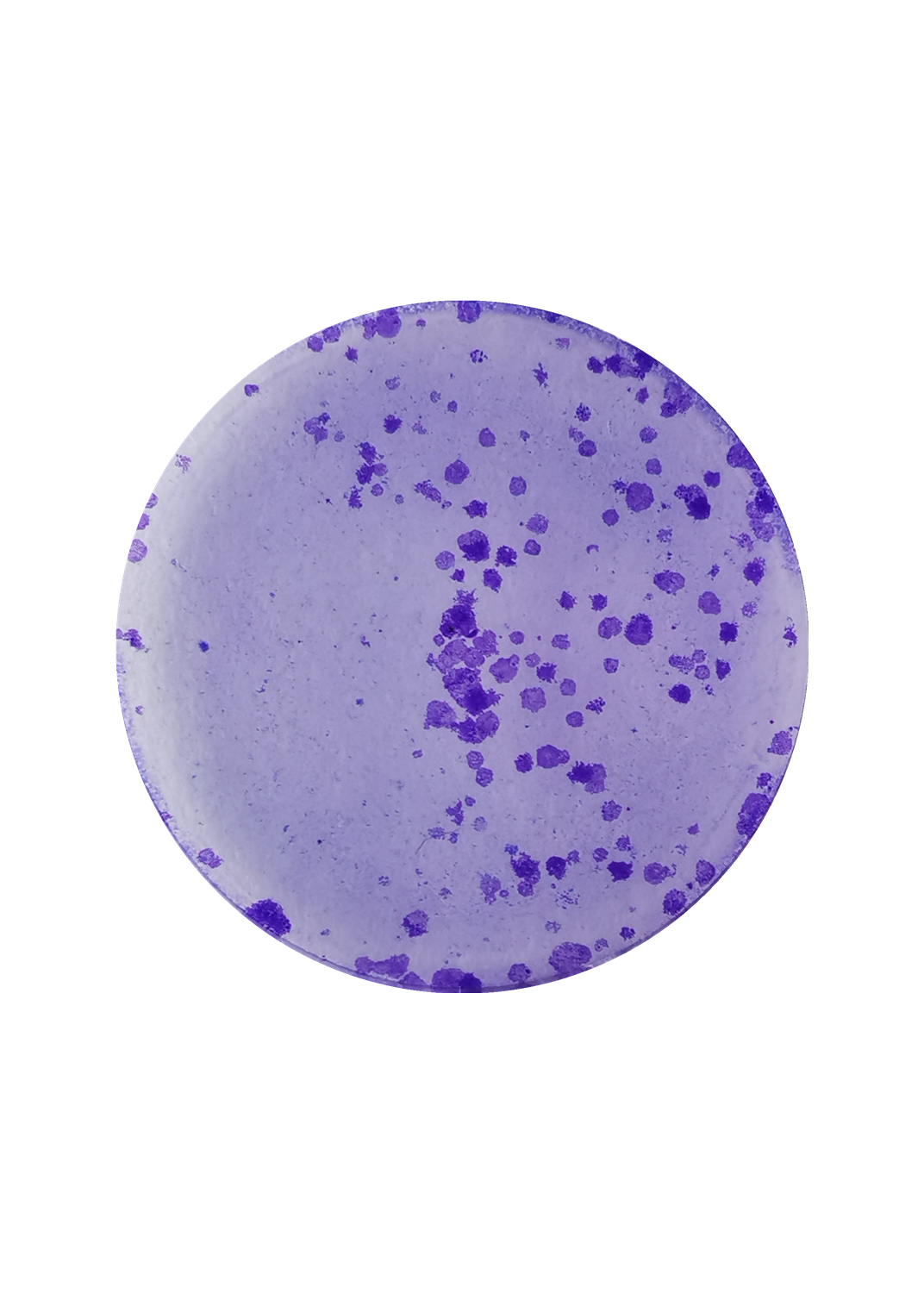

Supplement: S6 File — (ZIP) [file pone.0334639.s006.zip › S 11. File. Original Images. Fig4/S 11. File. Original FIgures. Fig.4/4h/BEL-7402 Overexpression.png]

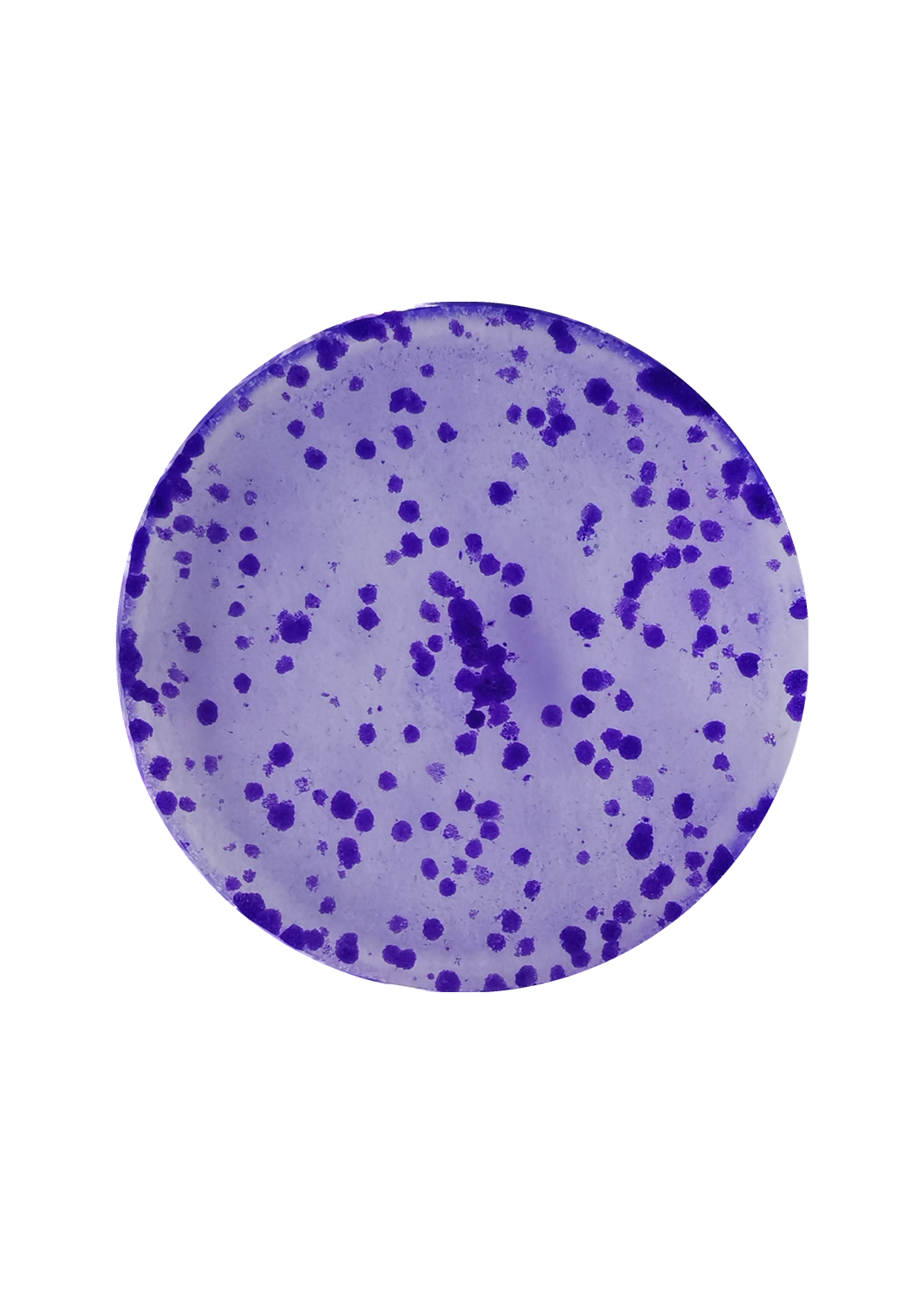

Supplement: S6 File — (ZIP) [file pone.0334639.s006.zip › S 11. File. Original Images. Fig4/S 11. File. Original FIgures. Fig.4/4h/Hepg2 MOCK.png]

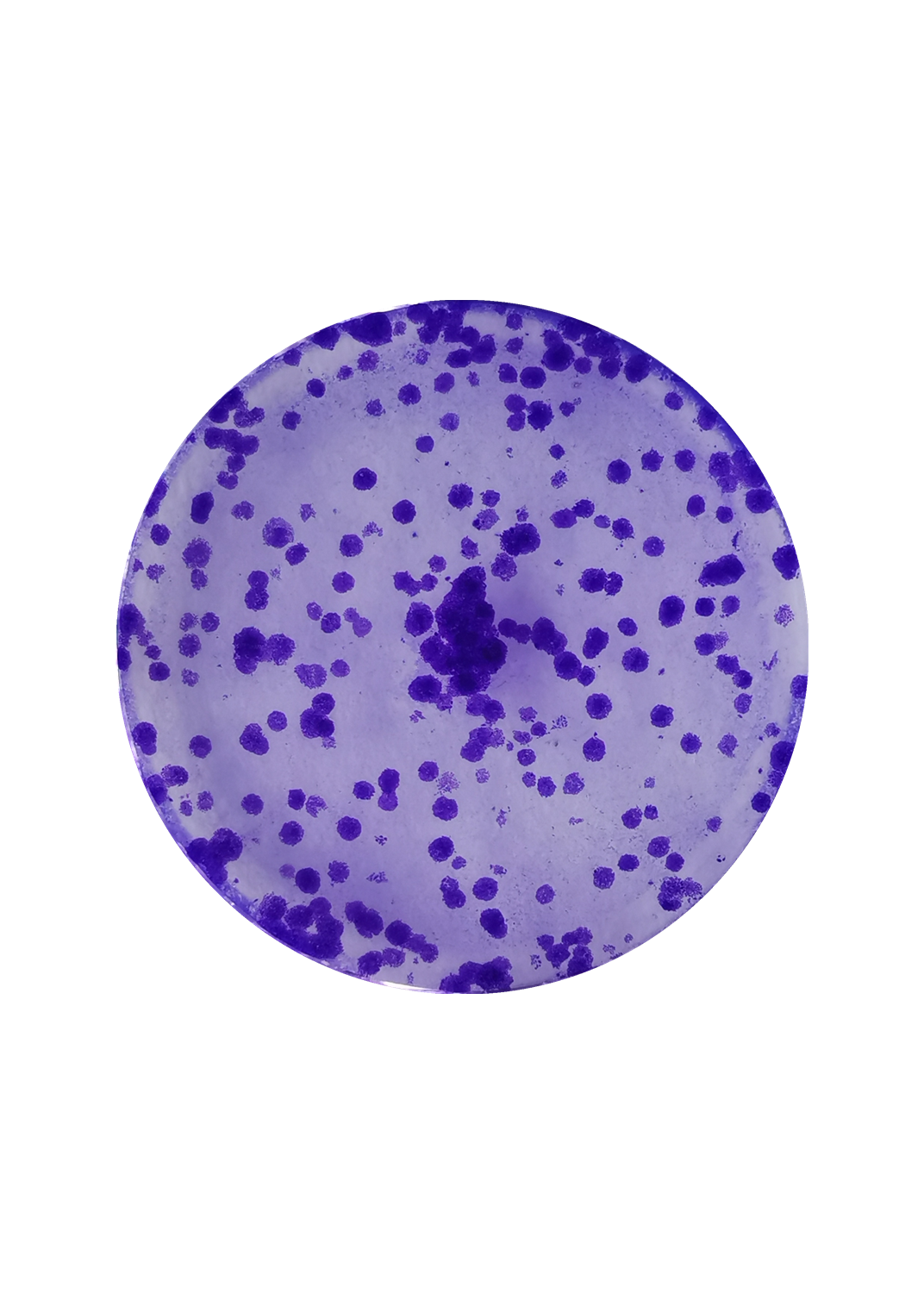

Supplement: S6 File — (ZIP) [file pone.0334639.s006.zip › S 11. File. Original Images. Fig4/S 11. File. Original FIgures. Fig.4/4h/HepG2 Overexpression.png]

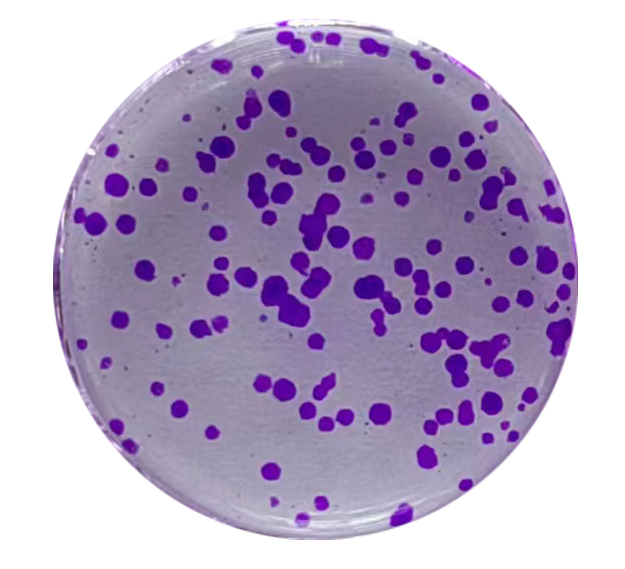

Supplement: S6 File — (ZIP) [file pone.0334639.s006.zip › S 11. File. Original Images. Fig4/S 11. File. Original FIgures. Fig.4/4h/SMMC-7721 MOCK.jpg]

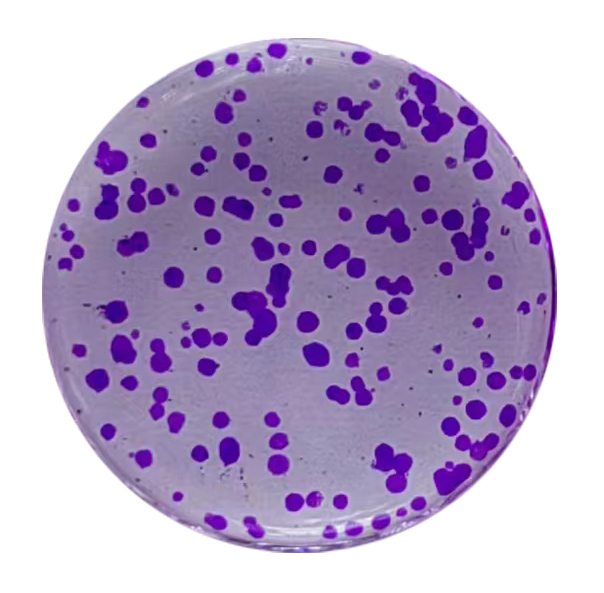

Supplement: S6 File — (ZIP) [file pone.0334639.s006.zip › S 11. File. Original Images. Fig4/S 11. File. Original FIgures. Fig.4/4h/SMMC-7721 Overexpression.jpg]

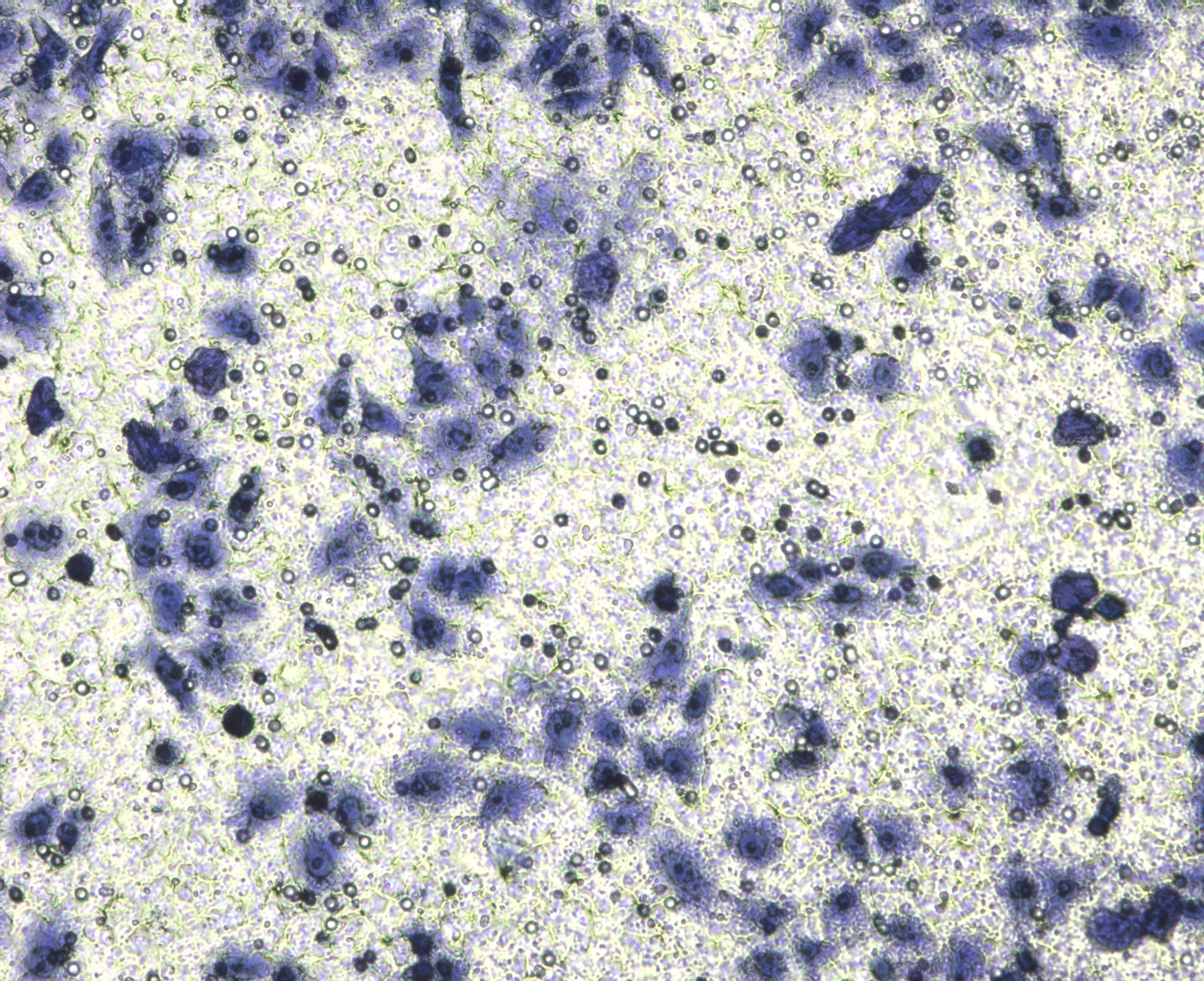

Supplement: S6 File — (ZIP) [file pone.0334639.s006.zip › S 11. File. Original Images. Fig4/S 11. File. Original FIgures. Fig.4/4j/Bel-7402 MOCK.jpg]

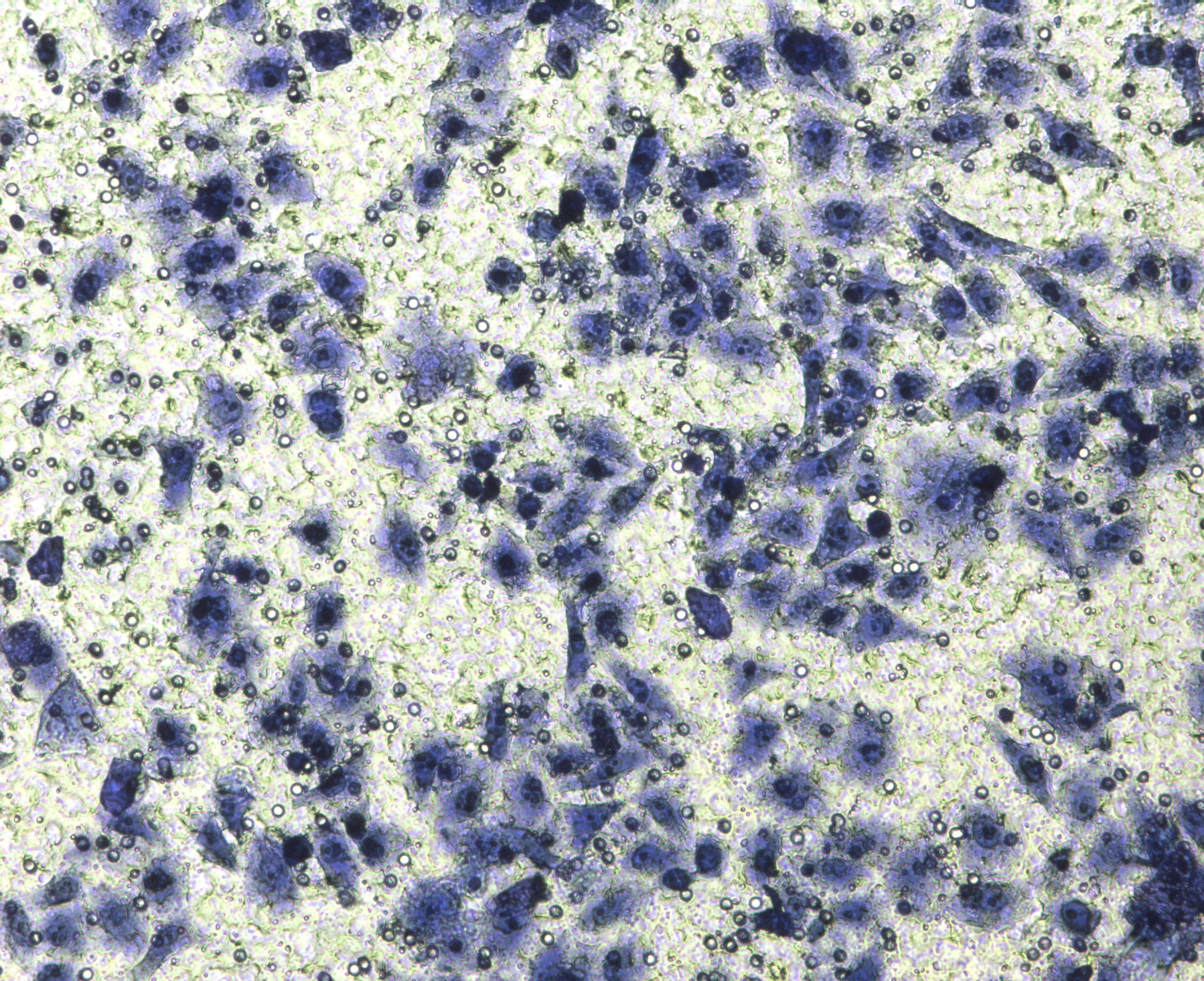

Supplement: S6 File — (ZIP) [file pone.0334639.s006.zip › S 11. File. Original Images. Fig4/S 11. File. Original FIgures. Fig.4/4j/Bel-7402 Overexpression.jpg]

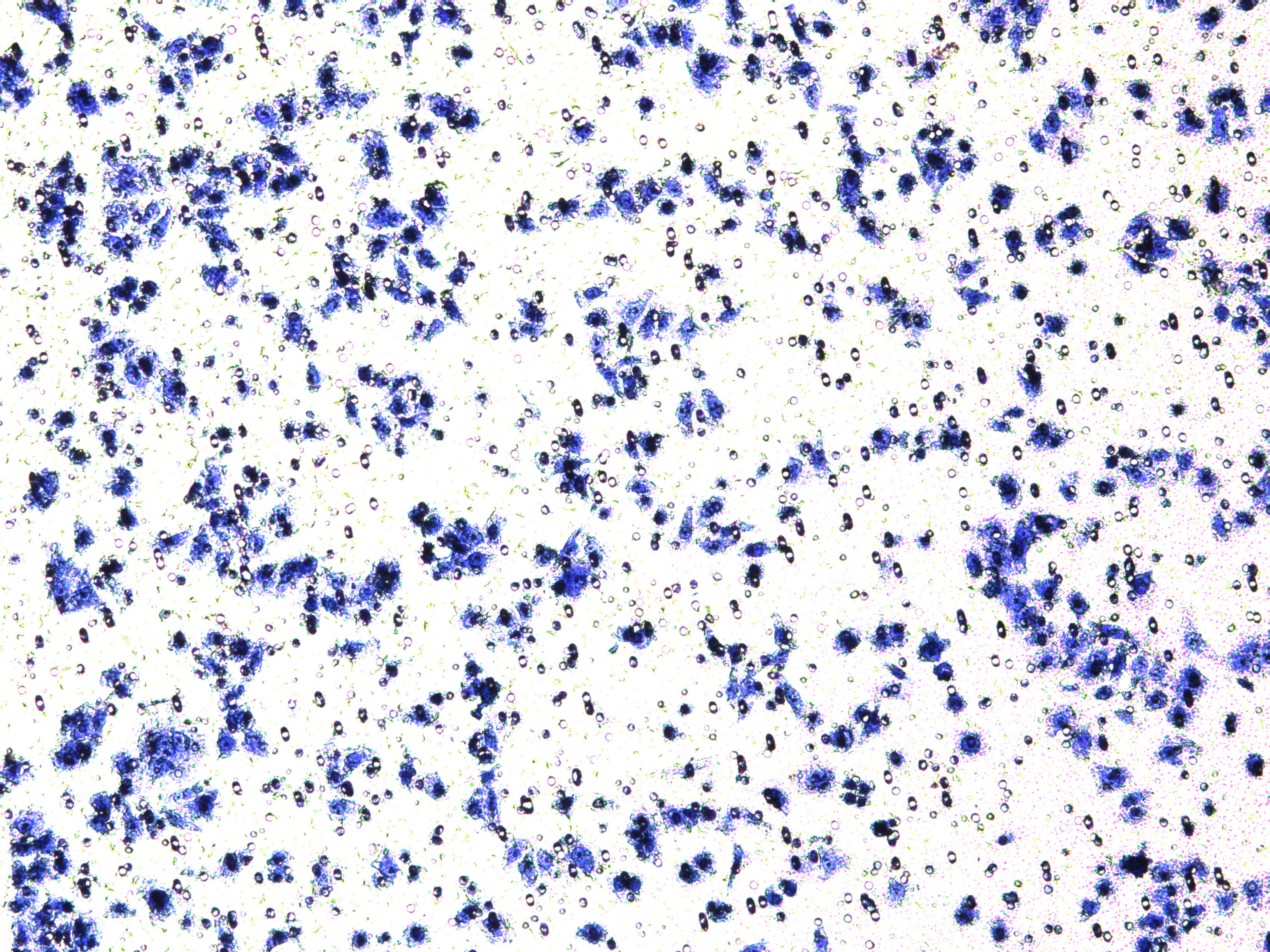

Supplement: S6 File — (ZIP) [file pone.0334639.s006.zip › S 11. File. Original Images. Fig4/S 11. File. Original FIgures. Fig.4/4j/Hepg2 MOCK.jpg]

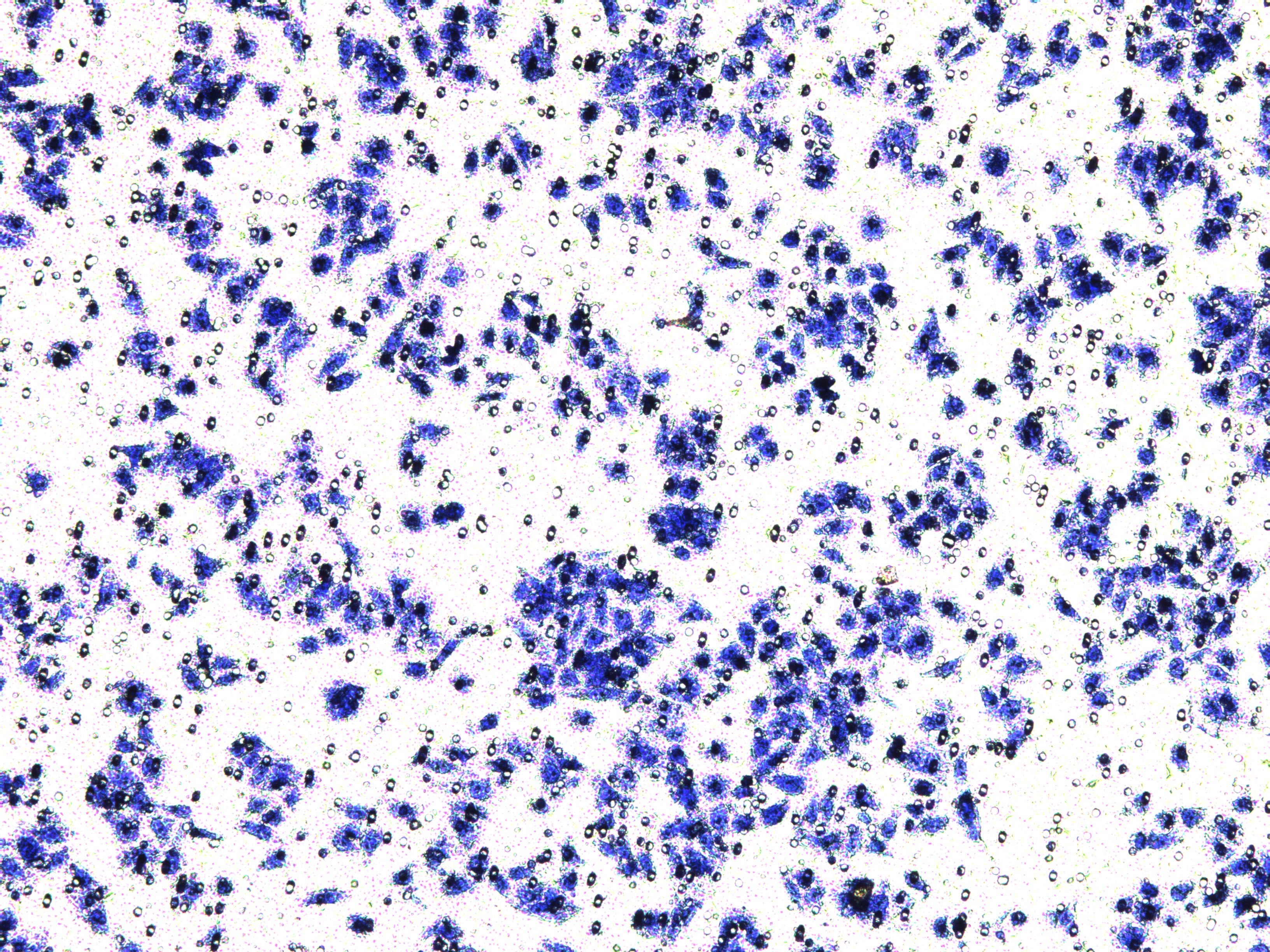

Supplement: S6 File — (ZIP) [file pone.0334639.s006.zip › S 11. File. Original Images. Fig4/S 11. File. Original FIgures. Fig.4/4j/Hepg2Overexpression.jpg]

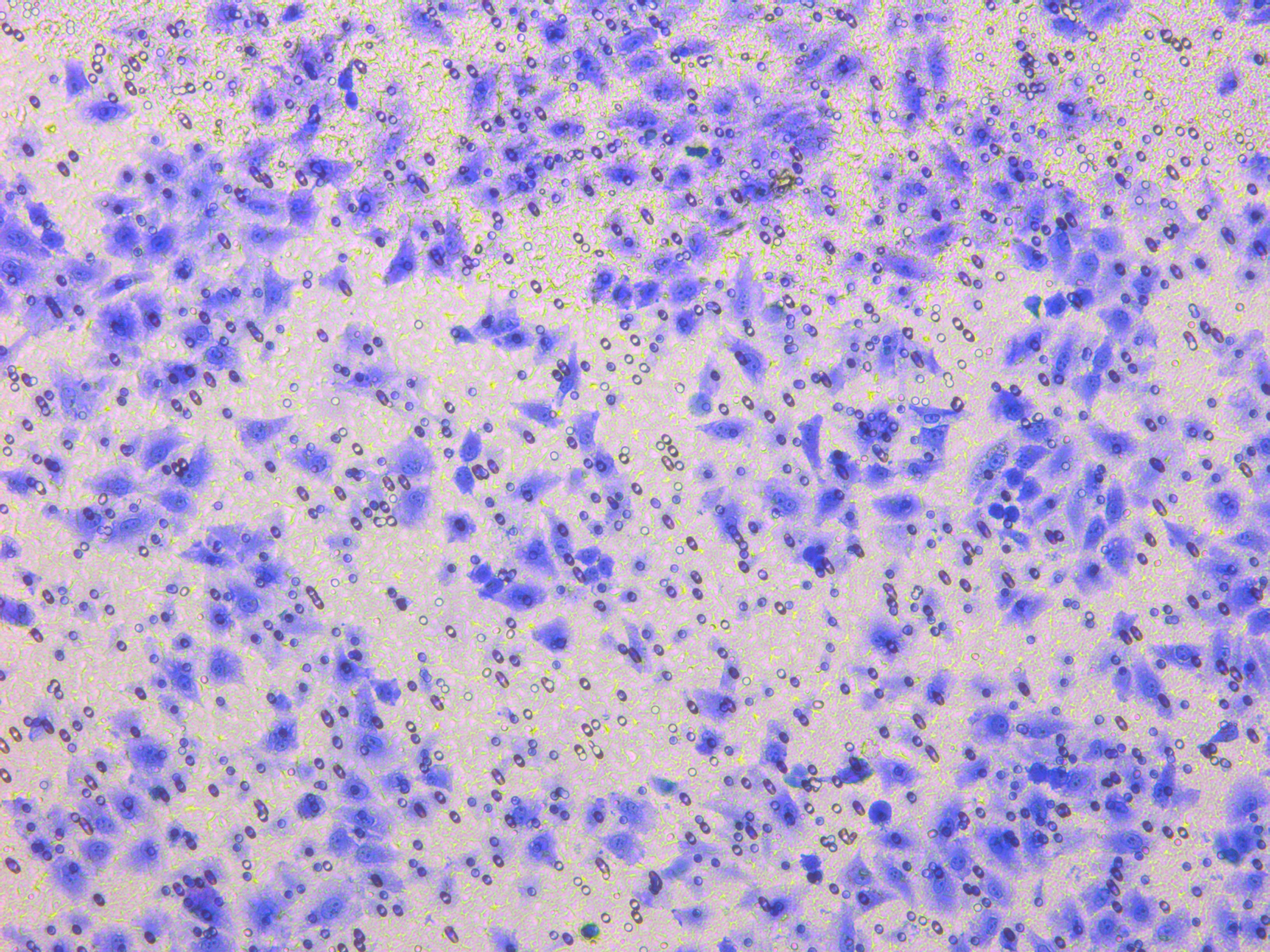

Supplement: S6 File — (ZIP) [file pone.0334639.s006.zip › S 11. File. Original Images. Fig4/S 11. File. Original FIgures. Fig.4/4j/SMMC-7721 cxcl3 Overexpression.jpg]

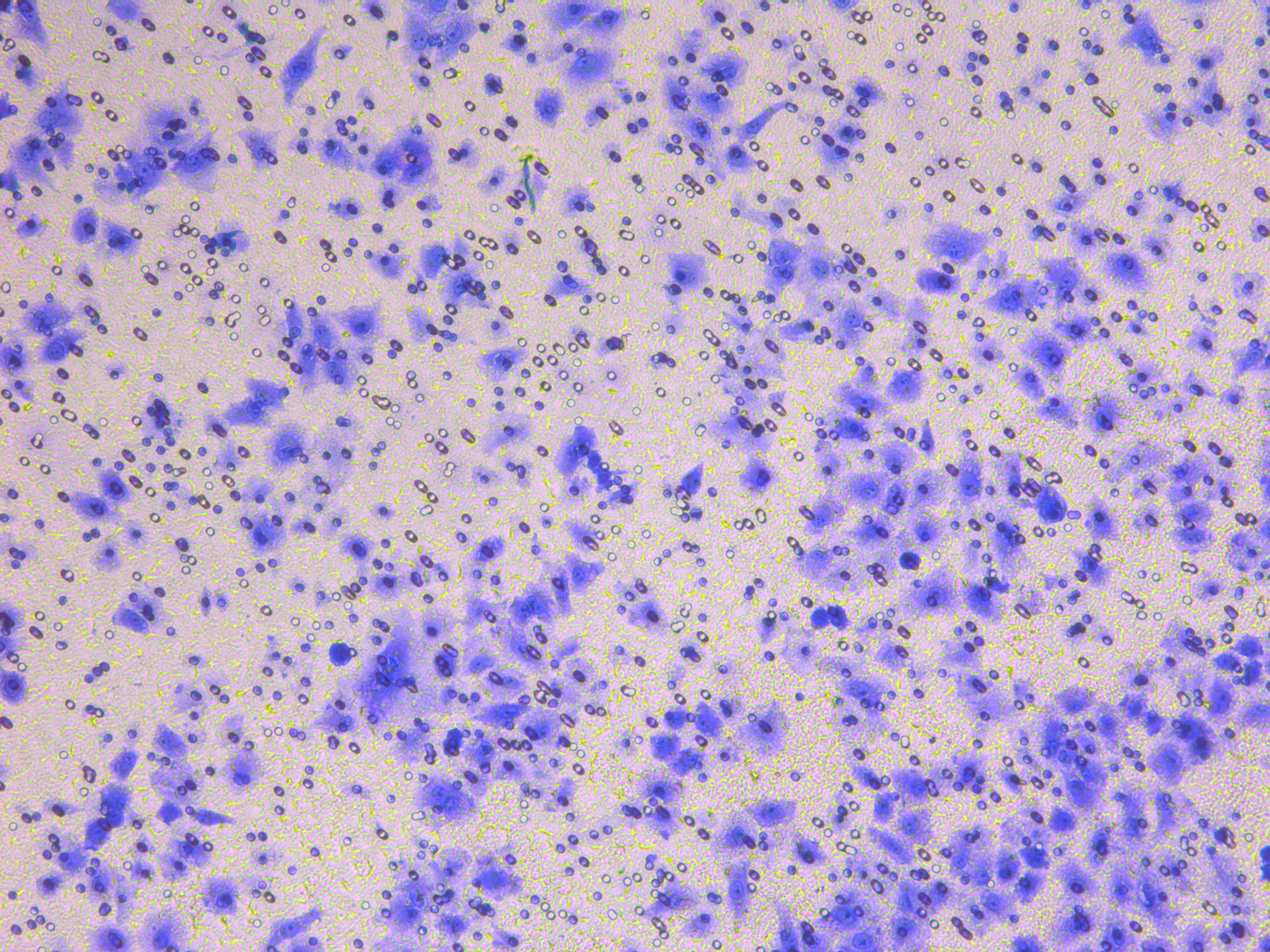

Supplement: S6 File — (ZIP) [file pone.0334639.s006.zip › S 11. File. Original Images. Fig4/S 11. File. Original FIgures. Fig.4/4j/SMMC-7721 cxcl3 MOCK.jpg]

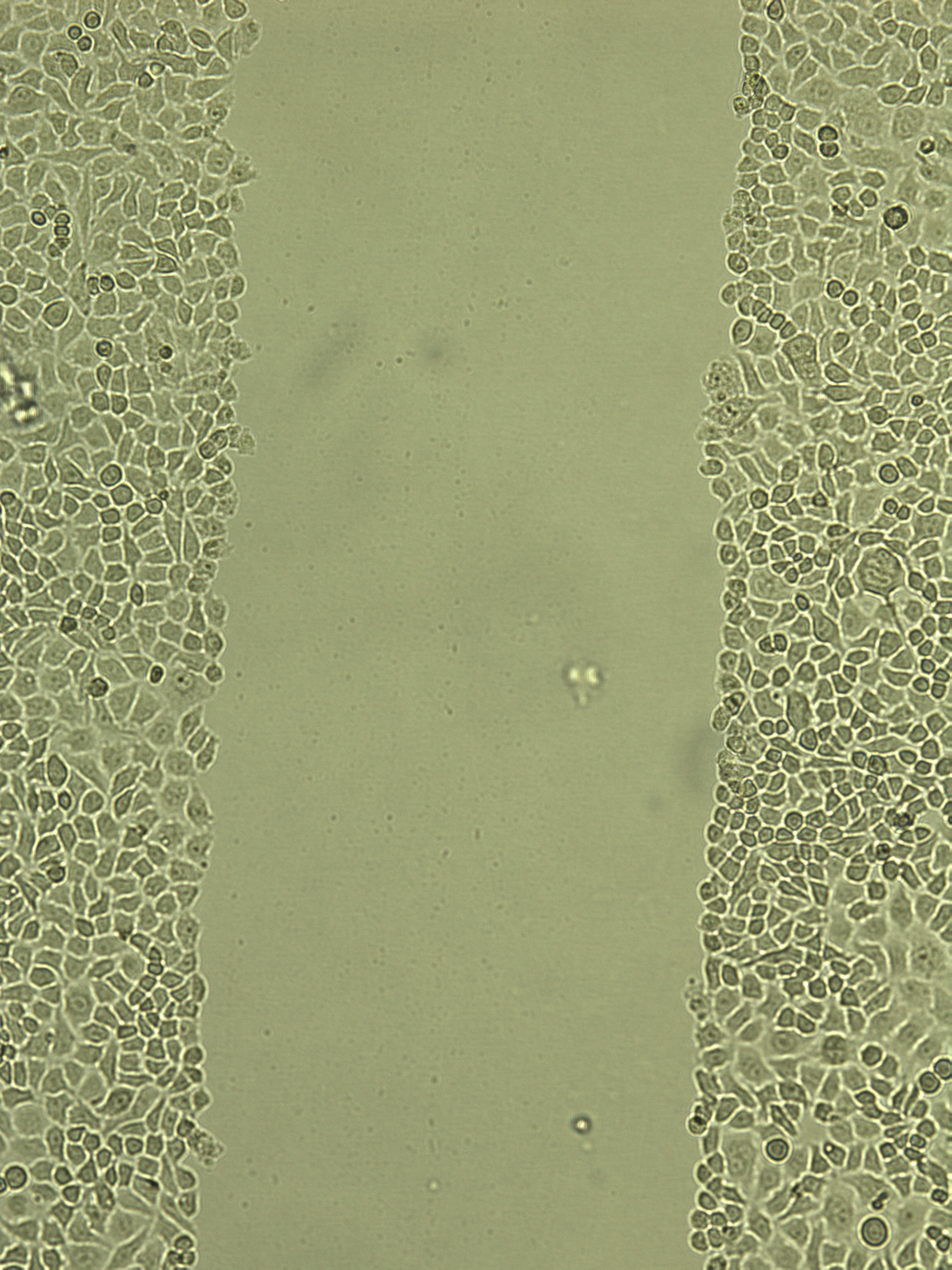

Supplement: S6 File — (ZIP) [file pone.0334639.s006.zip › S 11. File. Original Images. Fig4/S 11. File. Original FIgures. Fig.4/4k/bel-7402 CXCL3 MOCK--0H.jpg]

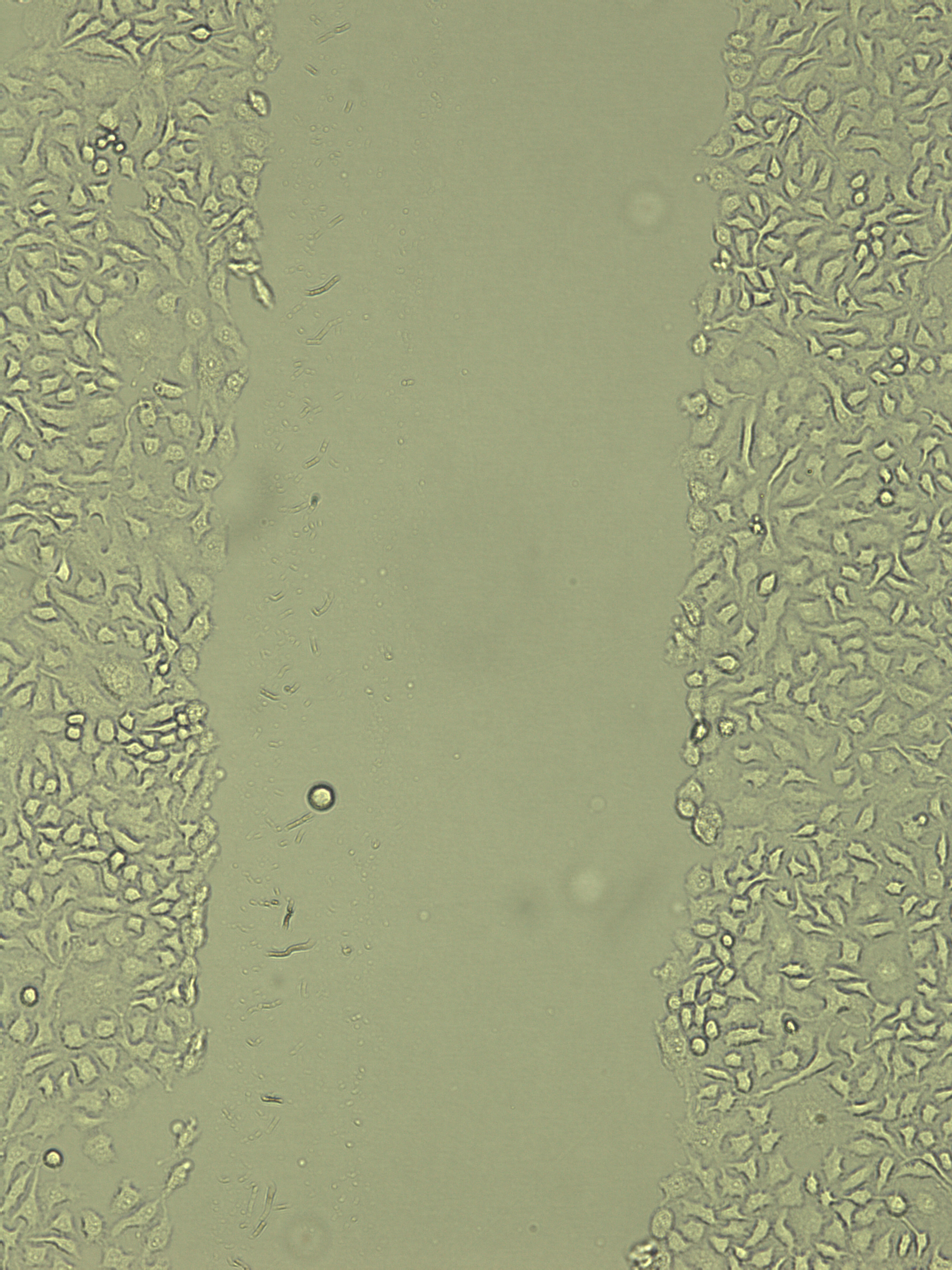

Supplement: S6 File — (ZIP) [file pone.0334639.s006.zip › S 11. File. Original Images. Fig4/S 11. File. Original FIgures. Fig.4/4k/bel-7402 CXCL3 MOCK--24H).jpg]

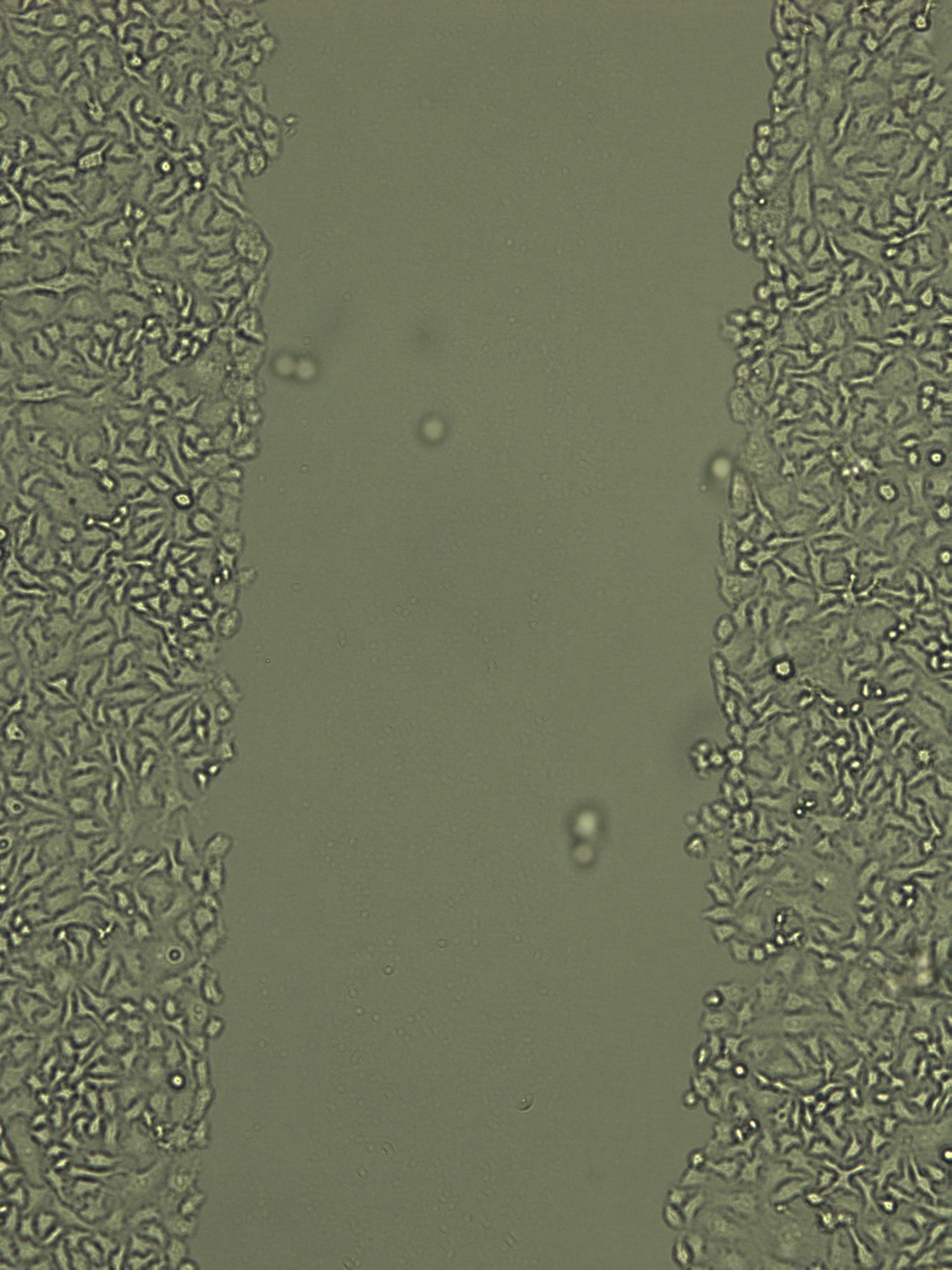

Supplement: S6 File — (ZIP) [file pone.0334639.s006.zip › S 11. File. Original Images. Fig4/S 11. File. Original FIgures. Fig.4/4k/bel-7402 CXCL3 Overexpression--24H.jpg]

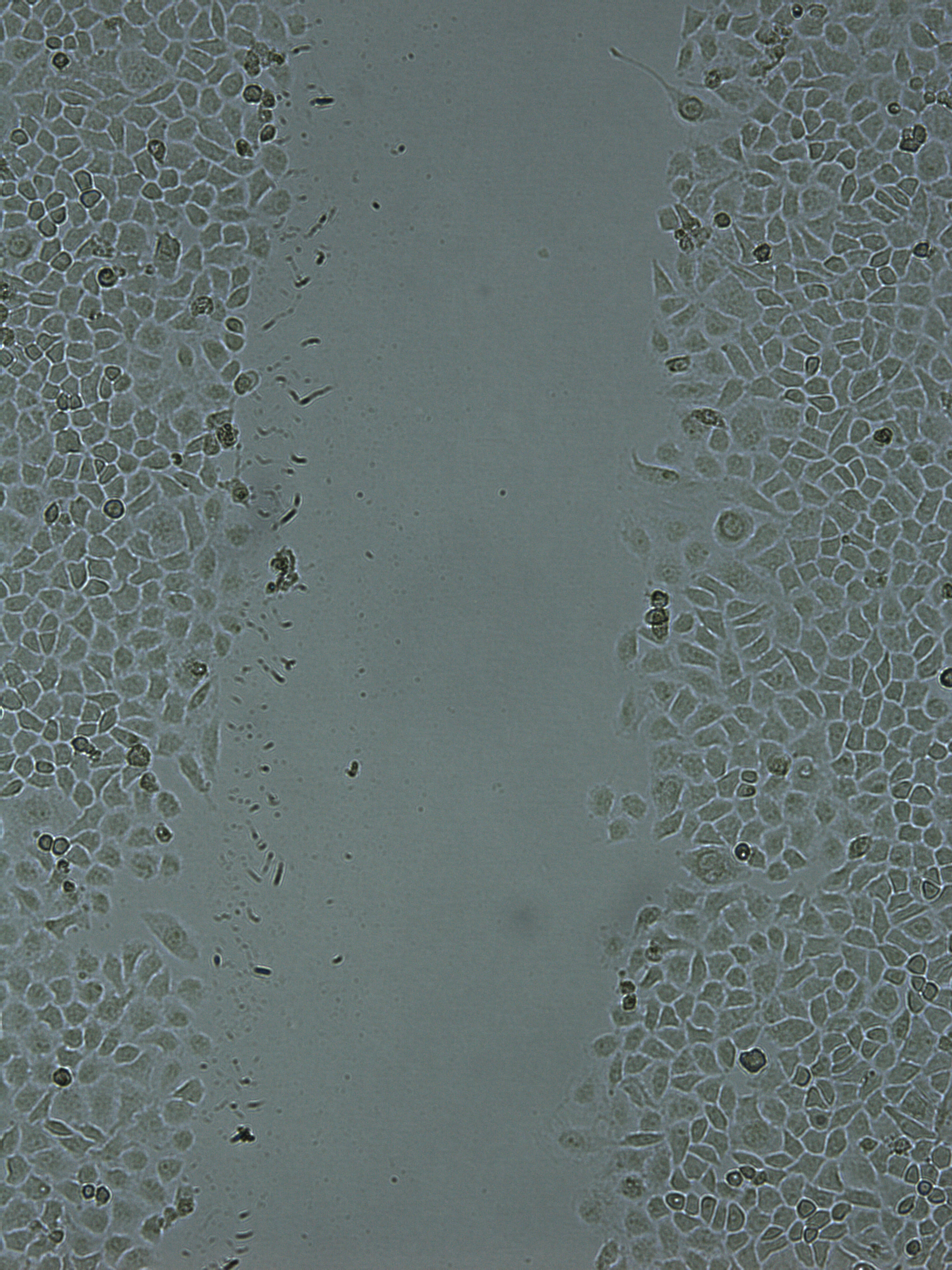

Supplement: S6 File — (ZIP) [file pone.0334639.s006.zip › S 11. File. Original Images. Fig4/S 11. File. Original FIgures. Fig.4/4k/bel-7402 CXCL3 Overexpression--48H(684).jpg]

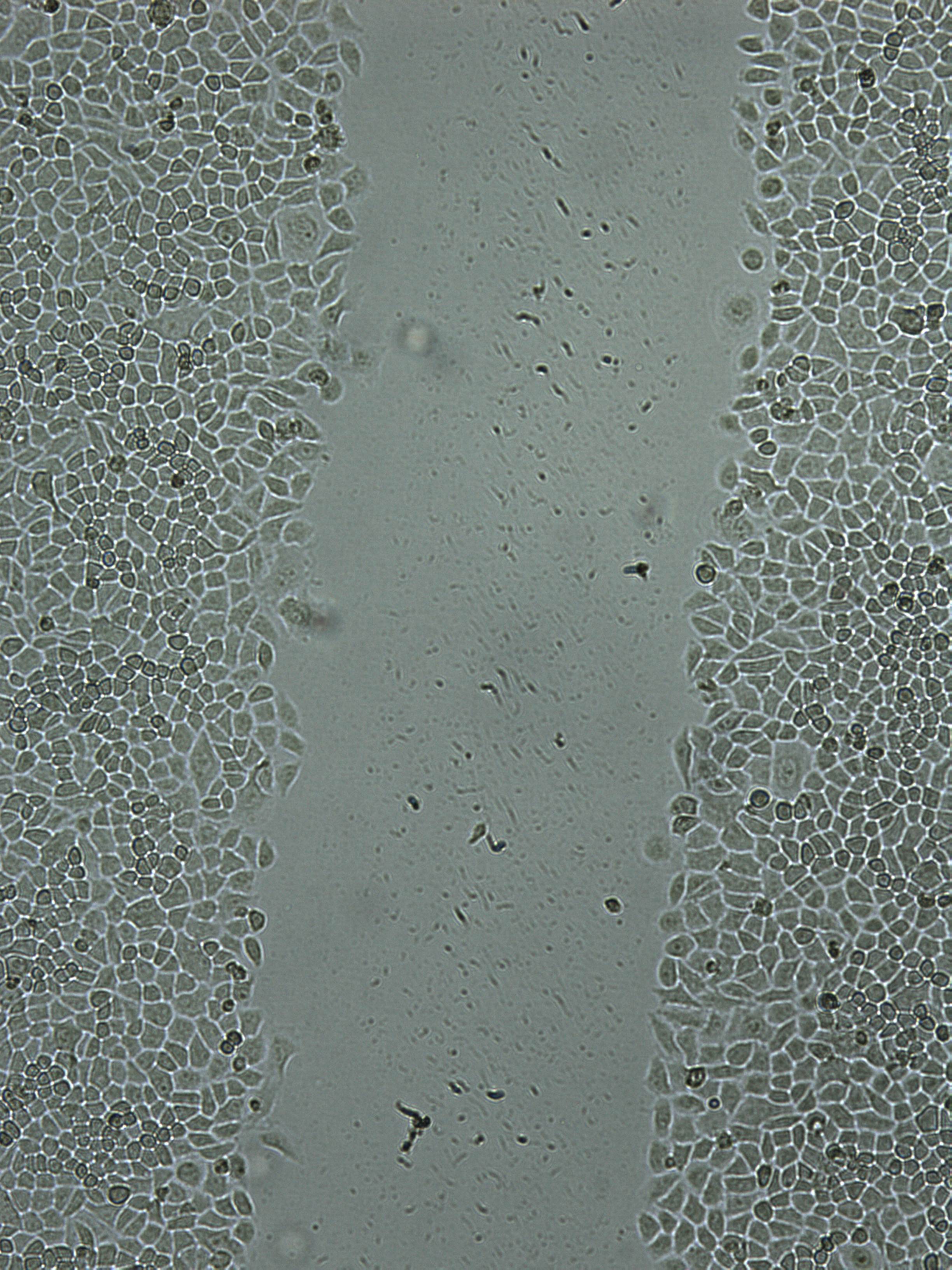

Supplement: S6 File — (ZIP) [file pone.0334639.s006.zip › S 11. File. Original Images. Fig4/S 11. File. Original FIgures. Fig.4/4k/bel-7402 cxcl3 MOCK 48H.jpg]

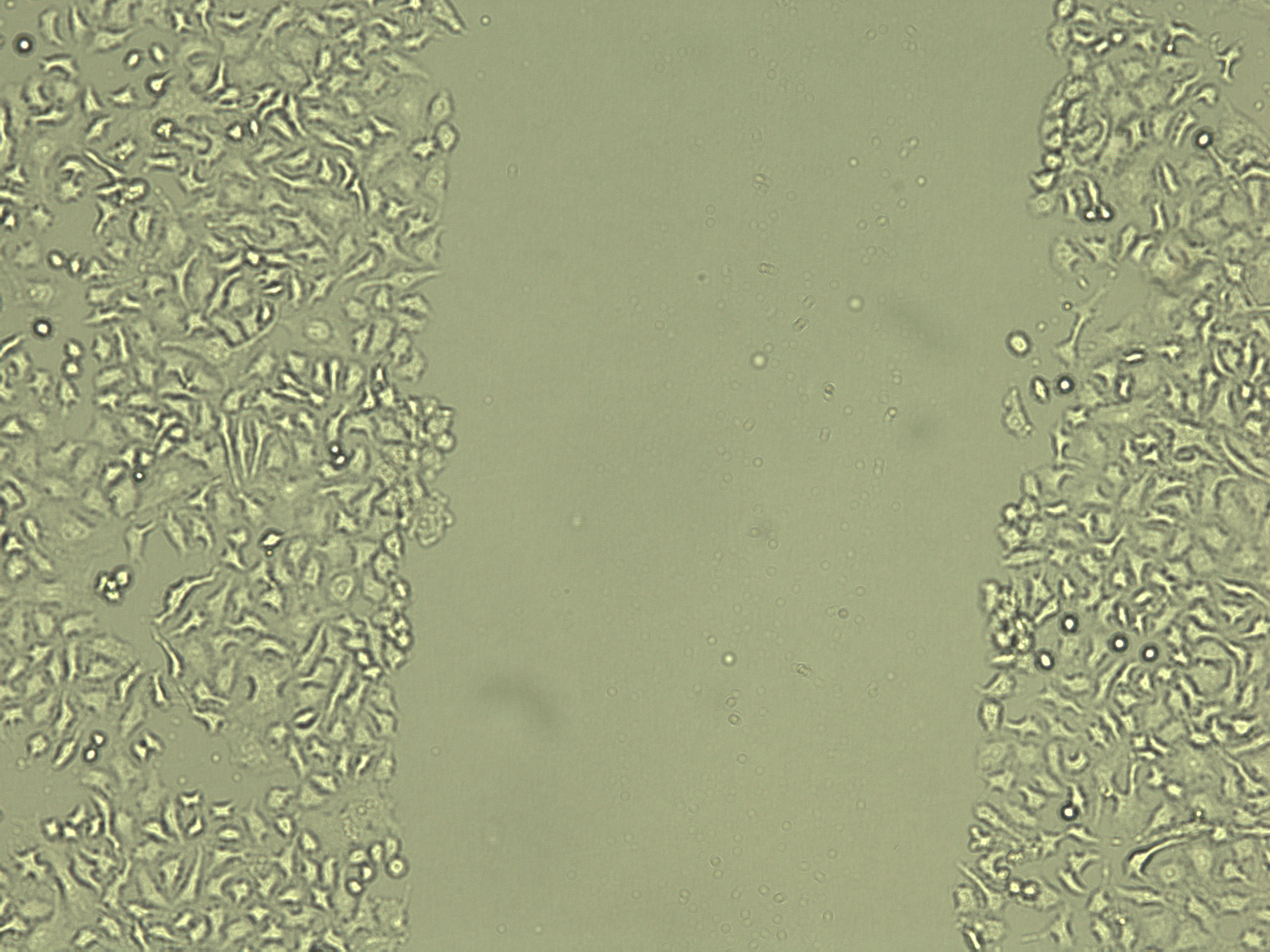

Supplement: S6 File — (ZIP) [file pone.0334639.s006.zip › S 11. File. Original Images. Fig4/S 11. File. Original FIgures. Fig.4/4k/bel-7402 CXCL3 Overexpression-- 0H .jpg]

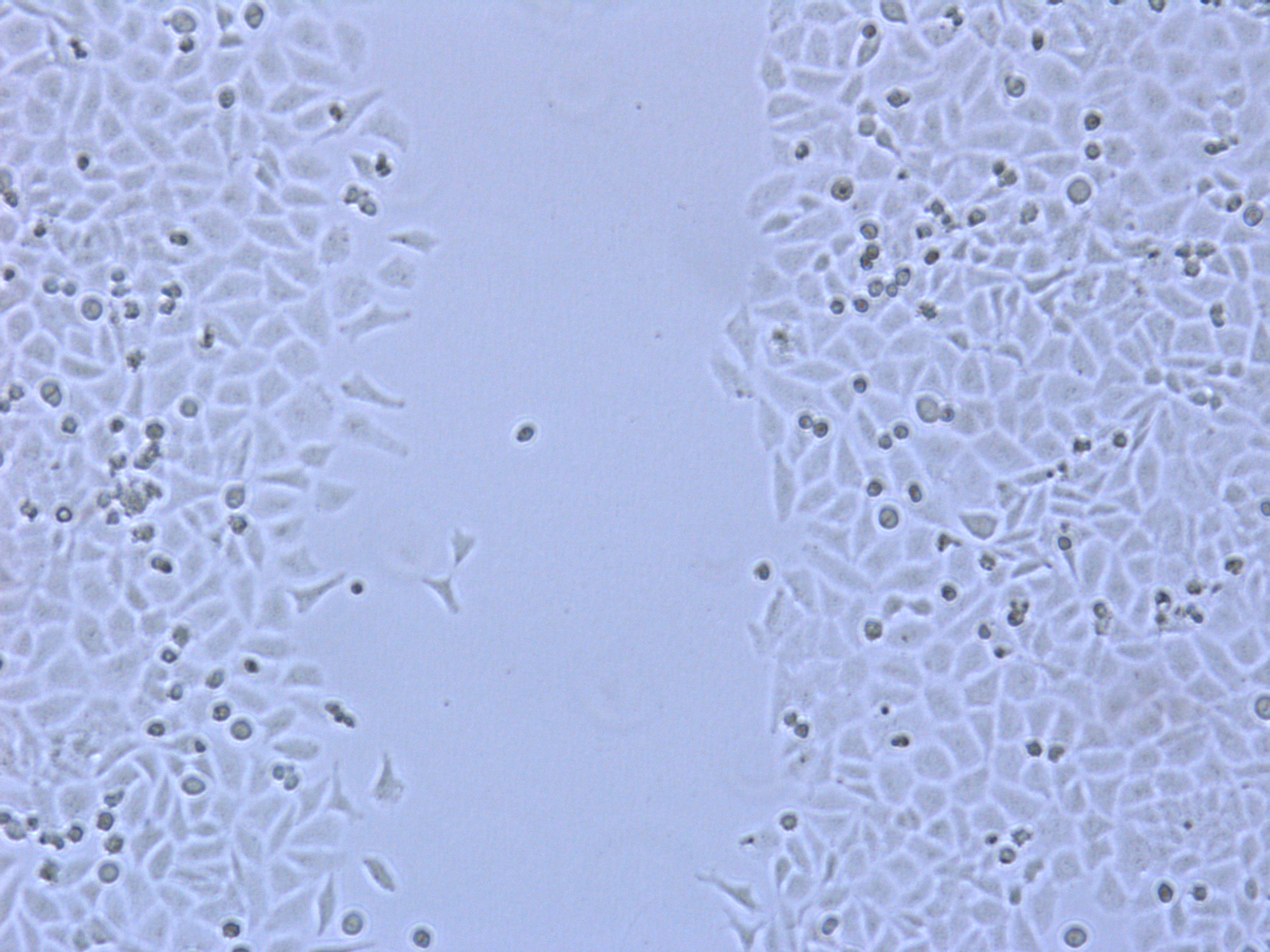

Supplement: S6 File — (ZIP) [file pone.0334639.s006.zip › S 11. File. Original Images. Fig4/S 11. File. Original FIgures. Fig.4/4l/HepG 2 CXCL3 MOCK --0h.jpg]

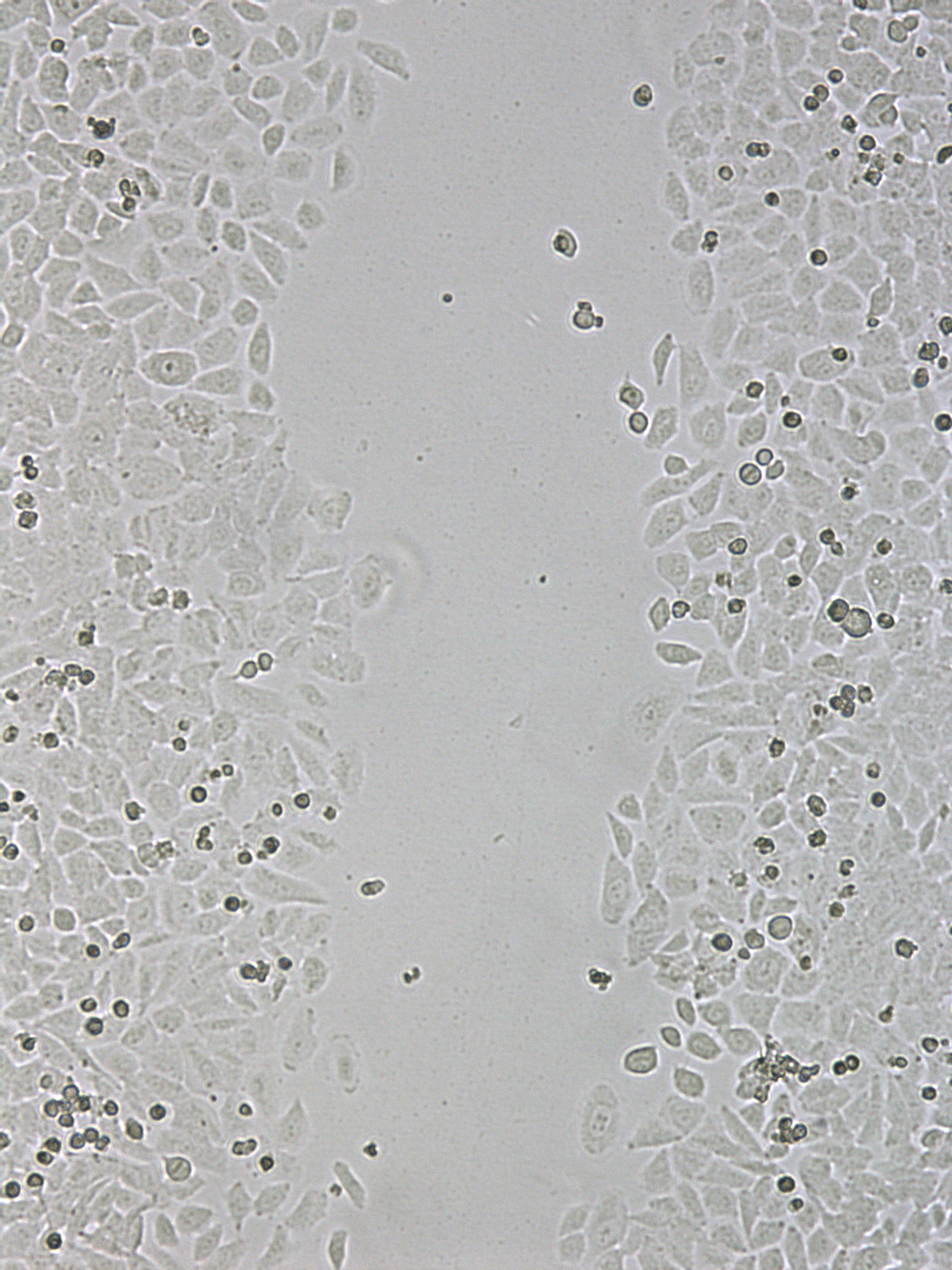

Supplement: S6 File — (ZIP) [file pone.0334639.s006.zip › S 11. File. Original Images. Fig4/S 11. File. Original FIgures. Fig.4/4l/HepG 2 CXCL3 MOCK--24h.jpg]

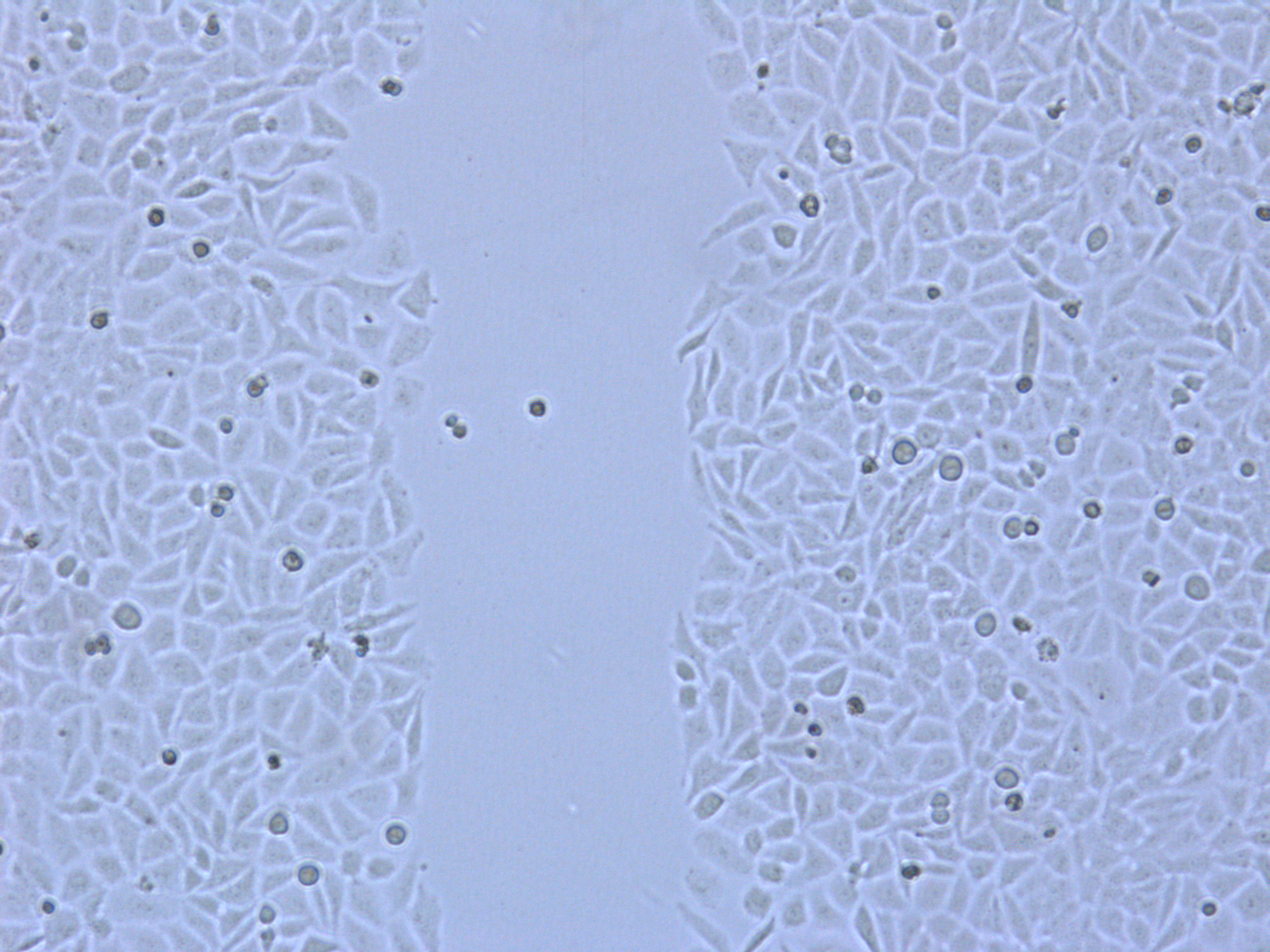

Supplement: S6 File — (ZIP) [file pone.0334639.s006.zip › S 11. File. Original Images. Fig4/S 11. File. Original FIgures. Fig.4/4l/HepG 2 CXCL3 Overexpression --0h.jpg]

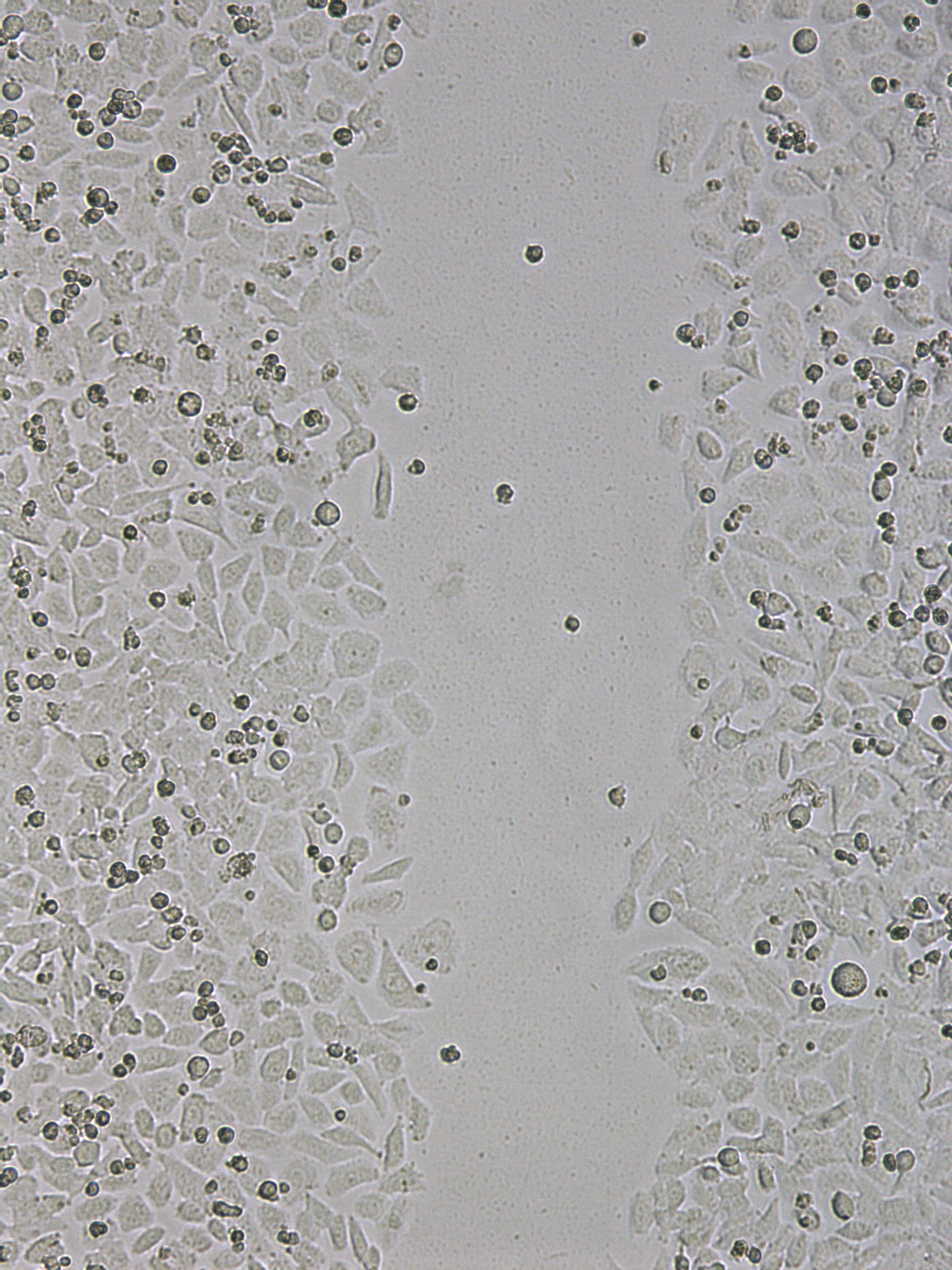

Supplement: S6 File — (ZIP) [file pone.0334639.s006.zip › S 11. File. Original Images. Fig4/S 11. File. Original FIgures. Fig.4/4l/HepG 2 CXCL3 Overexpression --24h.jpg]

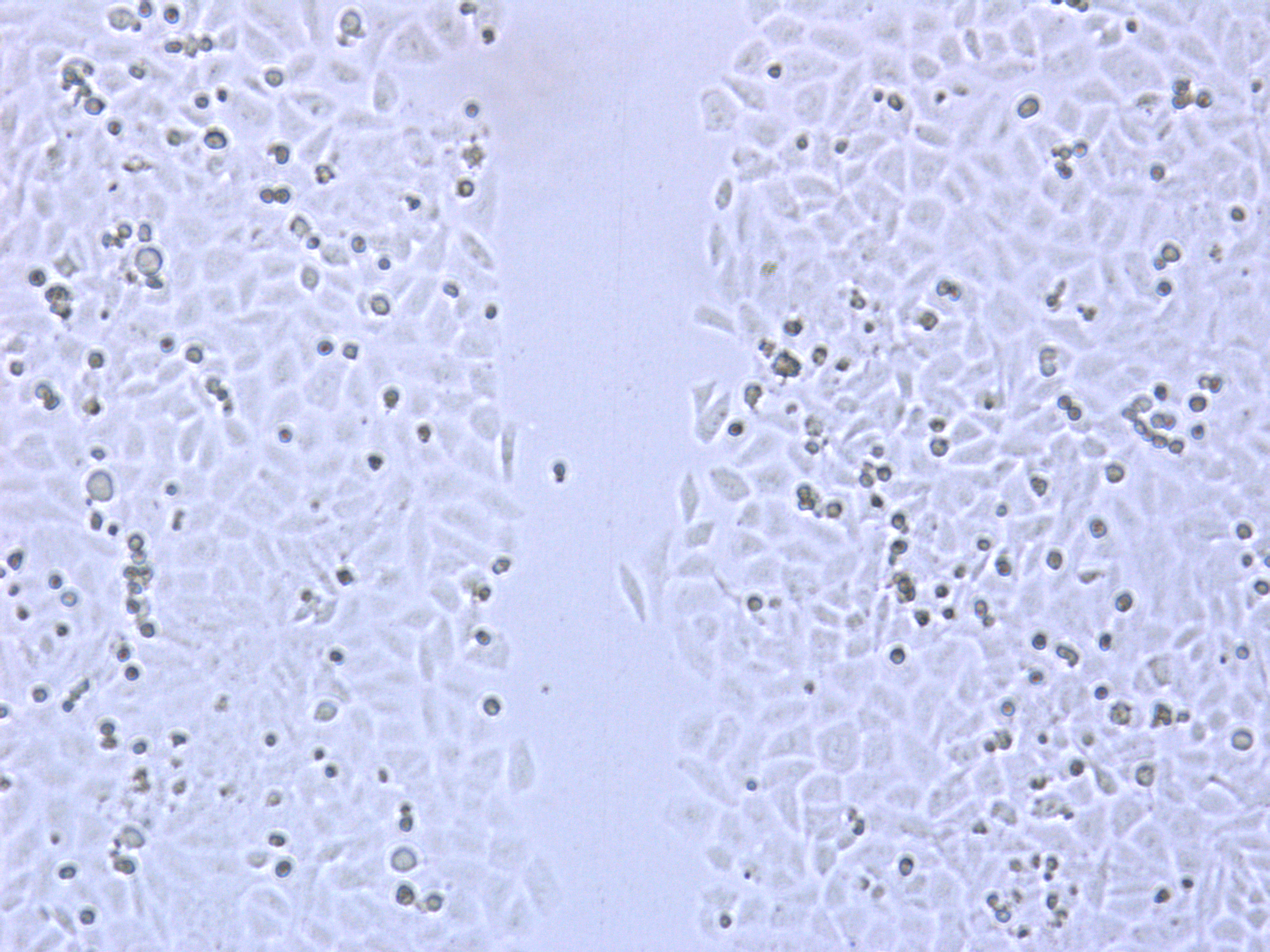

Supplement: S6 File — (ZIP) [file pone.0334639.s006.zip › S 11. File. Original Images. Fig4/S 11. File. Original FIgures. Fig.4/4l/HepG 2 CXCL3 Overexpression --48h.jpg]

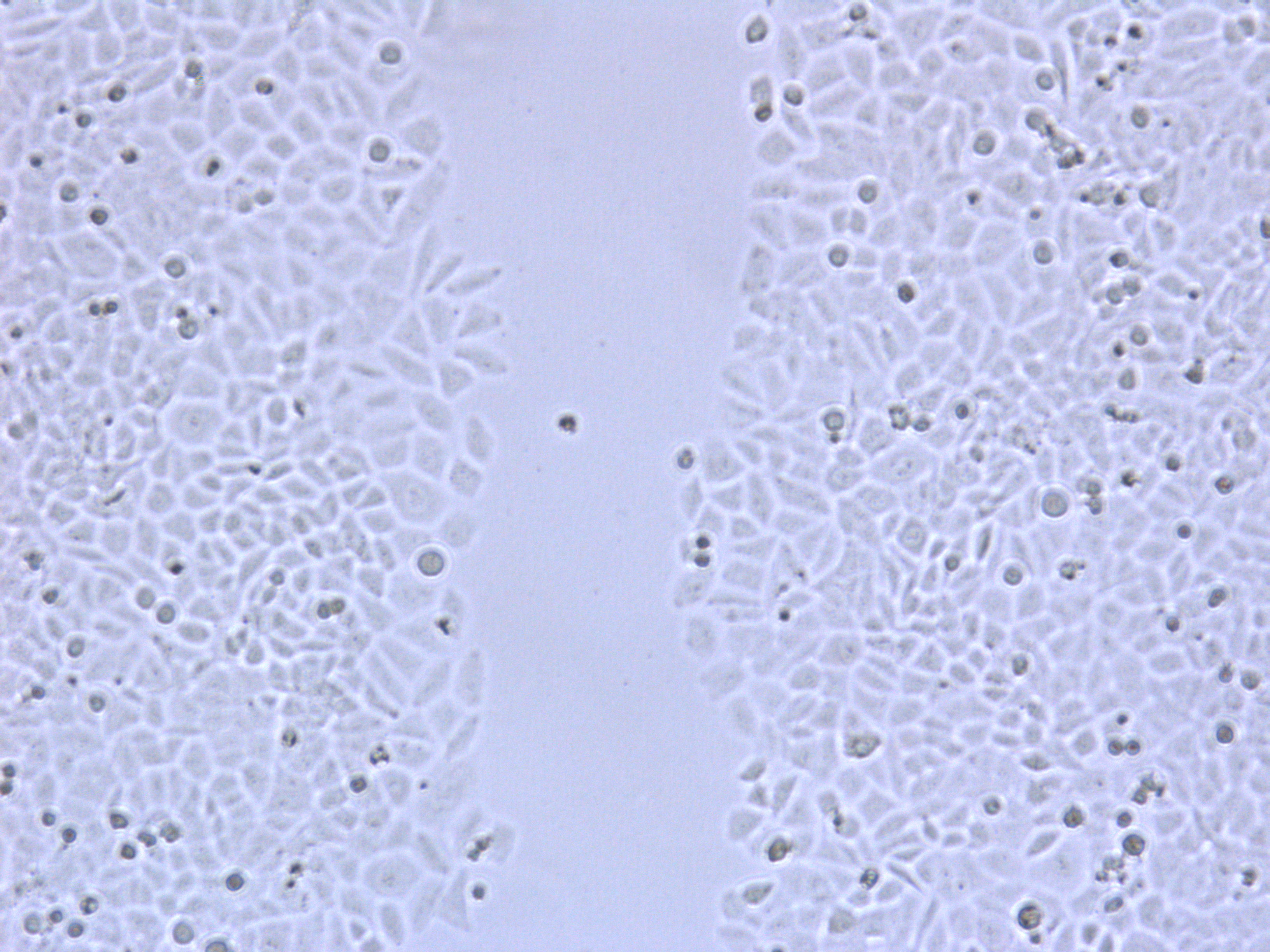

Supplement: S6 File — (ZIP) [file pone.0334639.s006.zip › S 11. File. Original Images. Fig4/S 11. File. Original FIgures. Fig.4/4l/HepG2 cxcl3 mock--48h.jpg]

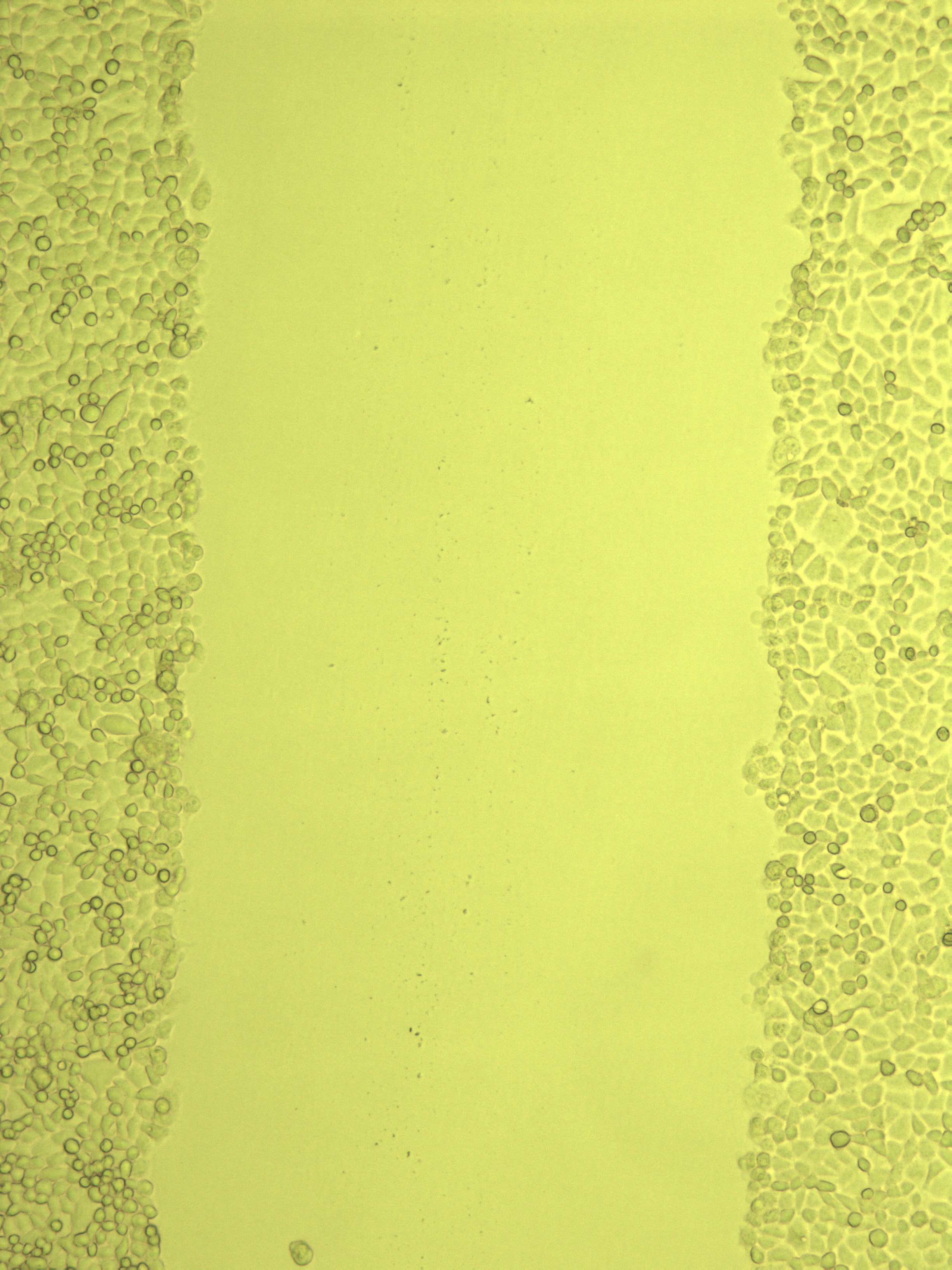

Supplement: S6 File — (ZIP) [file pone.0334639.s006.zip › S 11. File. Original Images. Fig4/S 11. File. Original FIgures. Fig.4/4m/SMMC-7721 MOCK--0H.jpg]
