# Supplementary figures and images for: CXCL3 promotes liver cancer progression by modulating the tumor microenvironment via the PI3K/AKT/mTOR pathway (part 2 of 3)
Source: PLoS One. 2025 Nov 19;20(11):e0334639. doi: 10.1371/journal.pone.0334639 (PMC12629499; doi:10.1371/journal.pone.0334639)

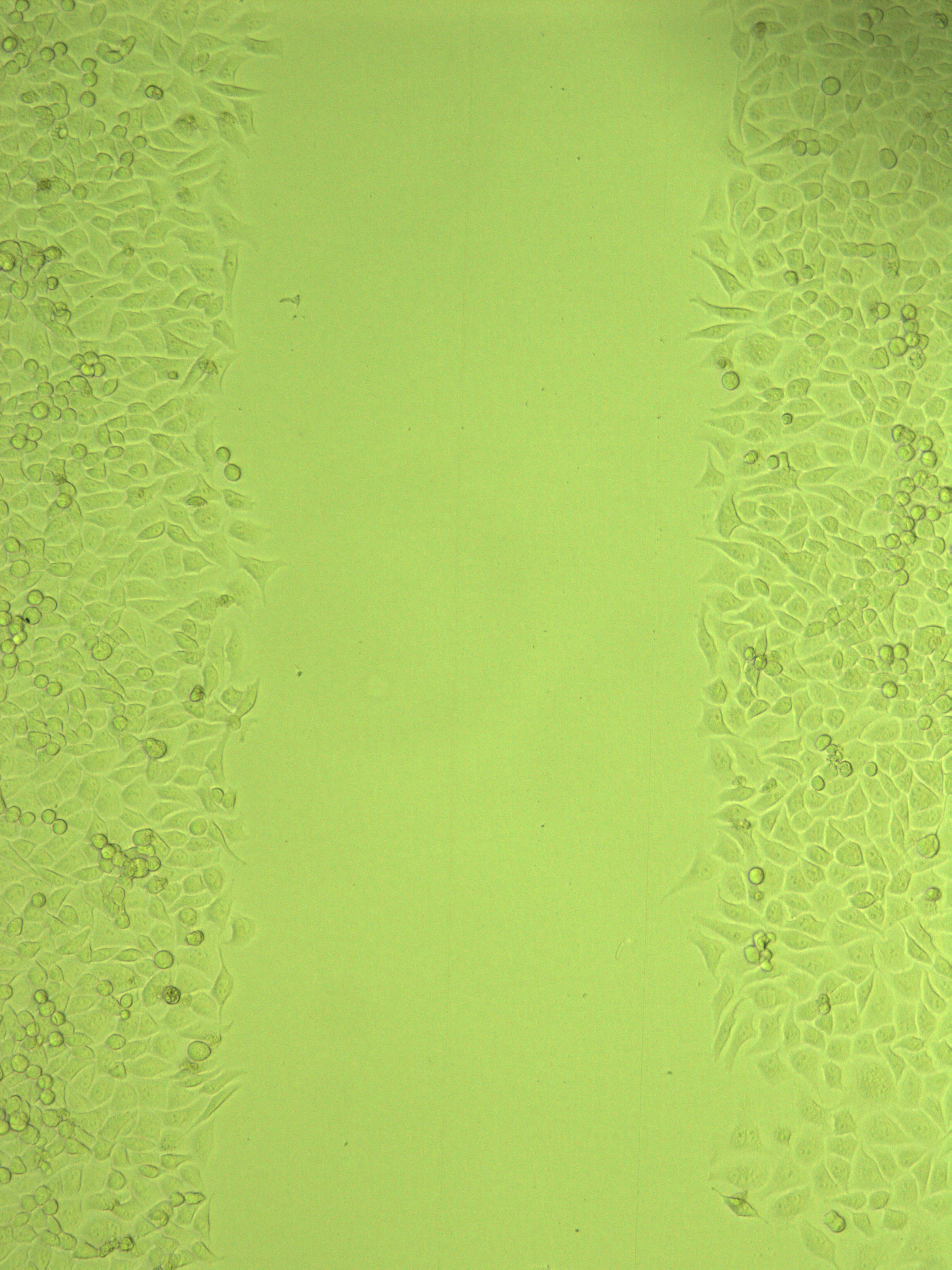

Supplement: S6 File — (ZIP) [file pone.0334639.s006.zip › S 11. File. Original Images. Fig4/S 11. File. Original FIgures. Fig.4/4m/SMMC-7721 MOCK--24H.jpg]

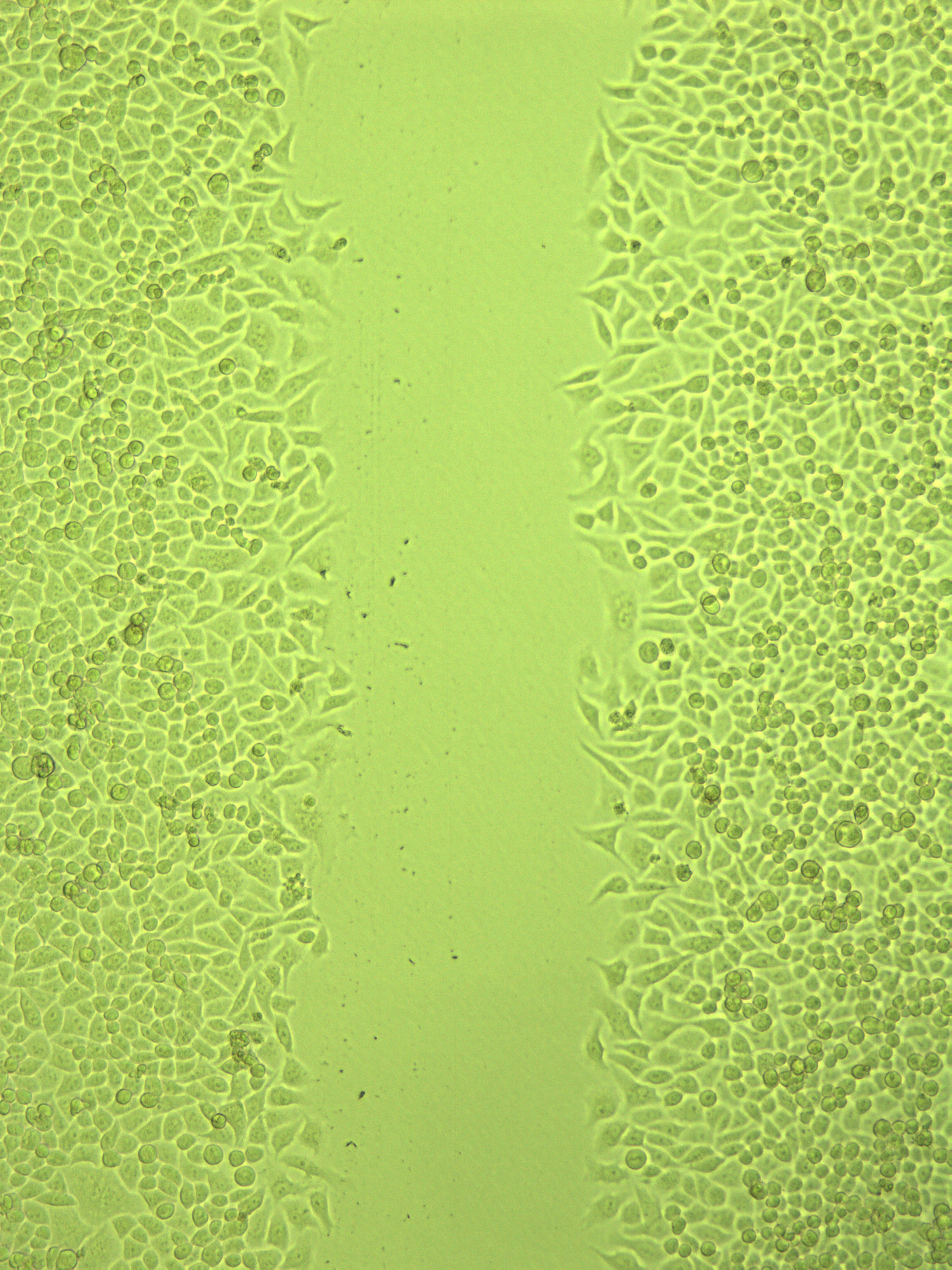

Supplement: S6 File — (ZIP) [file pone.0334639.s006.zip › S 11. File. Original Images. Fig4/S 11. File. Original FIgures. Fig.4/4m/SMMC-7721 Overexpression --48h.jpg]

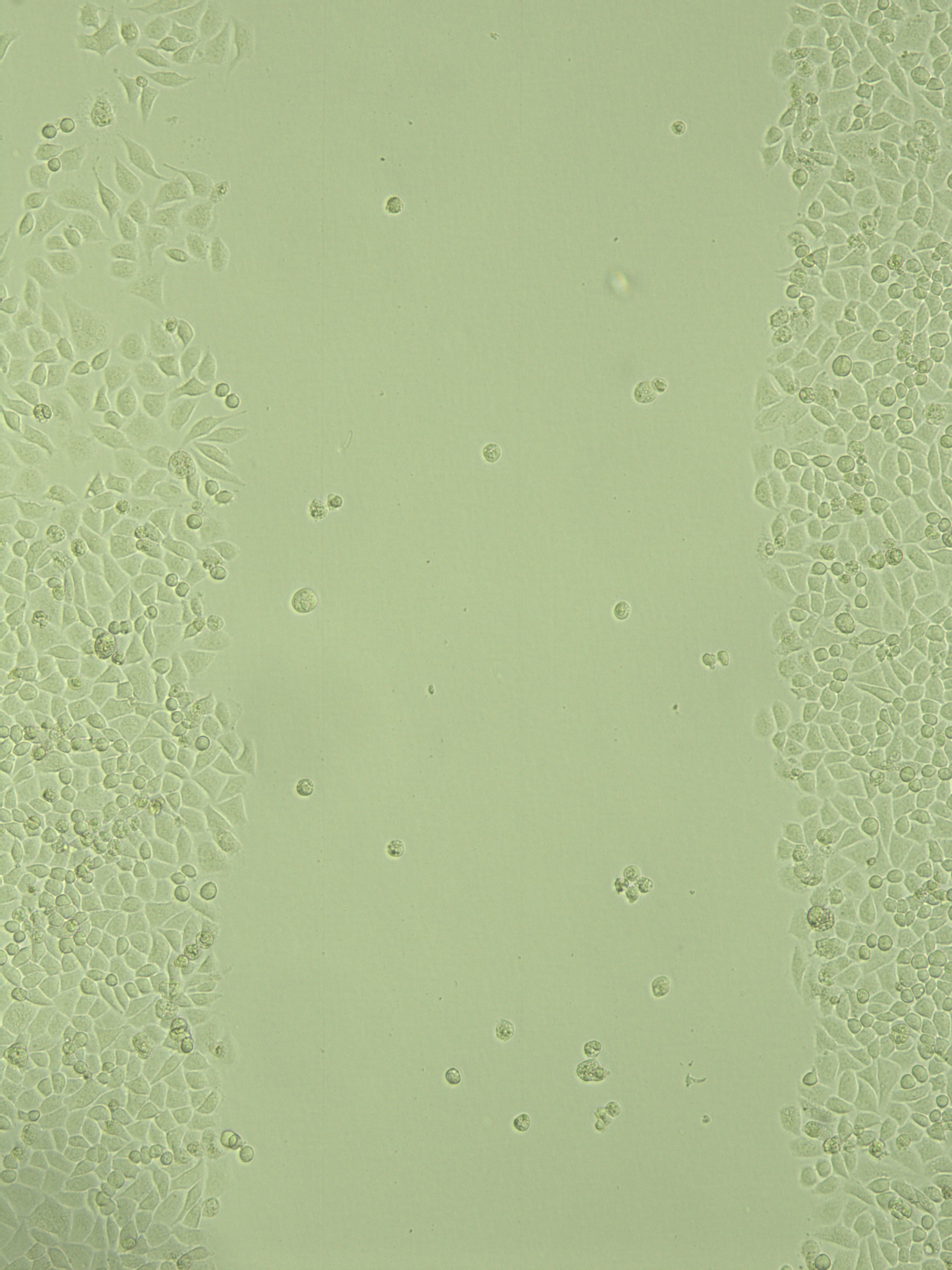

Supplement: S6 File — (ZIP) [file pone.0334639.s006.zip › S 11. File. Original Images. Fig4/S 11. File. Original FIgures. Fig.4/4m/SMMC-7721 Overexpression--0H.jpg]

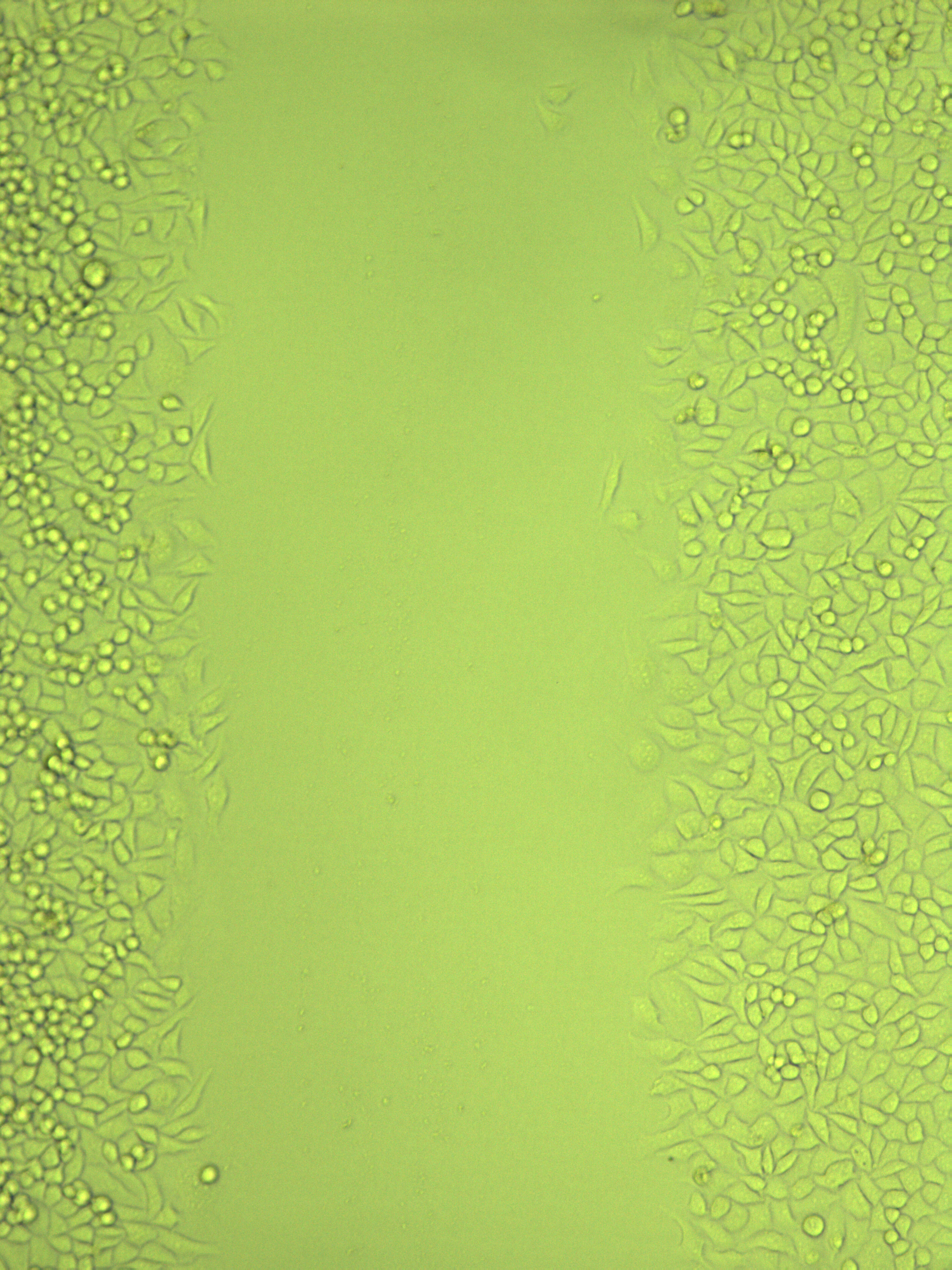

Supplement: S6 File — (ZIP) [file pone.0334639.s006.zip › S 11. File. Original Images. Fig4/S 11. File. Original FIgures. Fig.4/4m/SMMC-7721 Overexpression--24H).jpg]

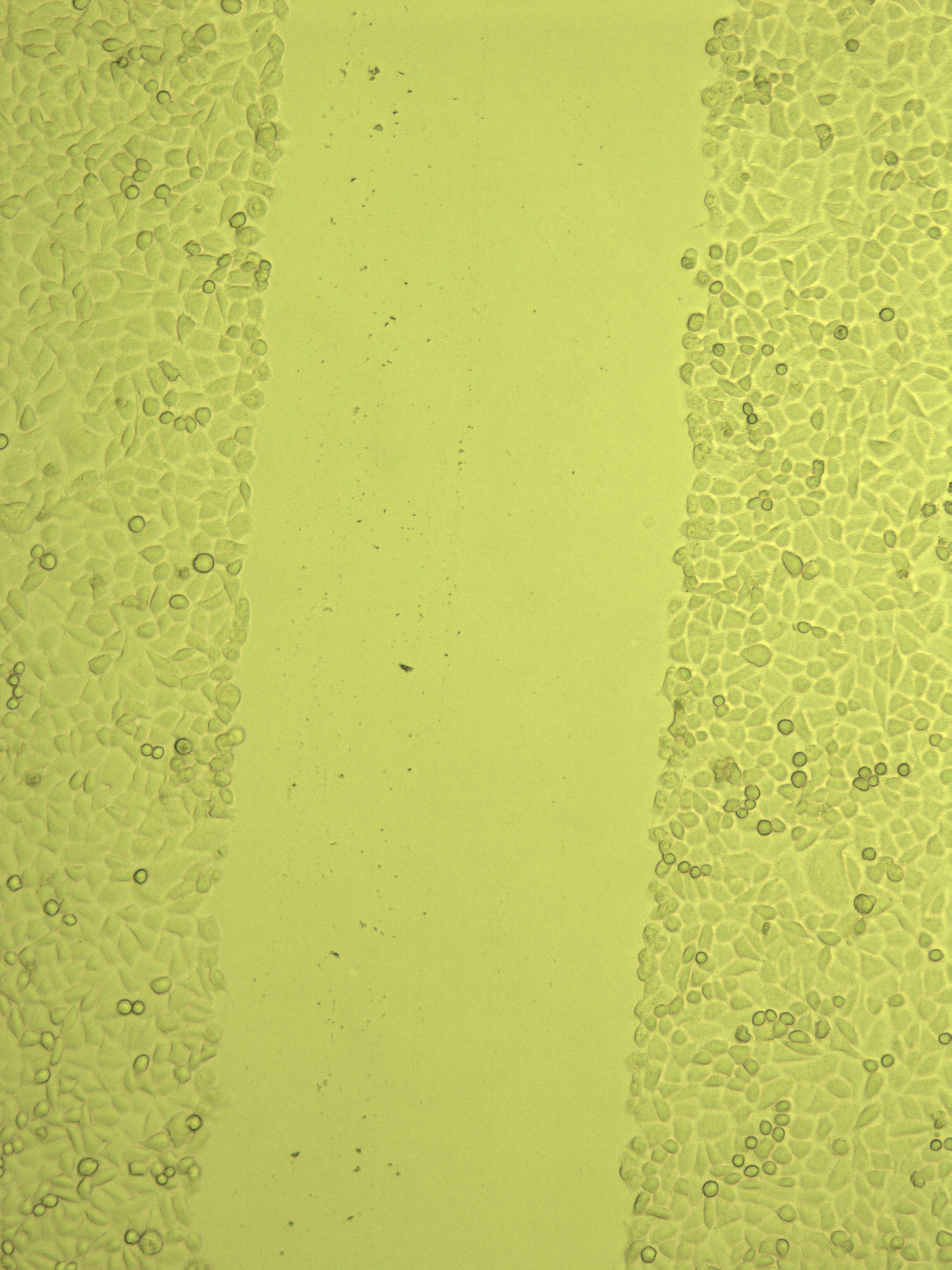

Supplement: S6 File — (ZIP) [file pone.0334639.s006.zip › S 11. File. Original Images. Fig4/S 11. File. Original FIgures. Fig.4/4m/SMMC-7721MOCK 48H.jpg]

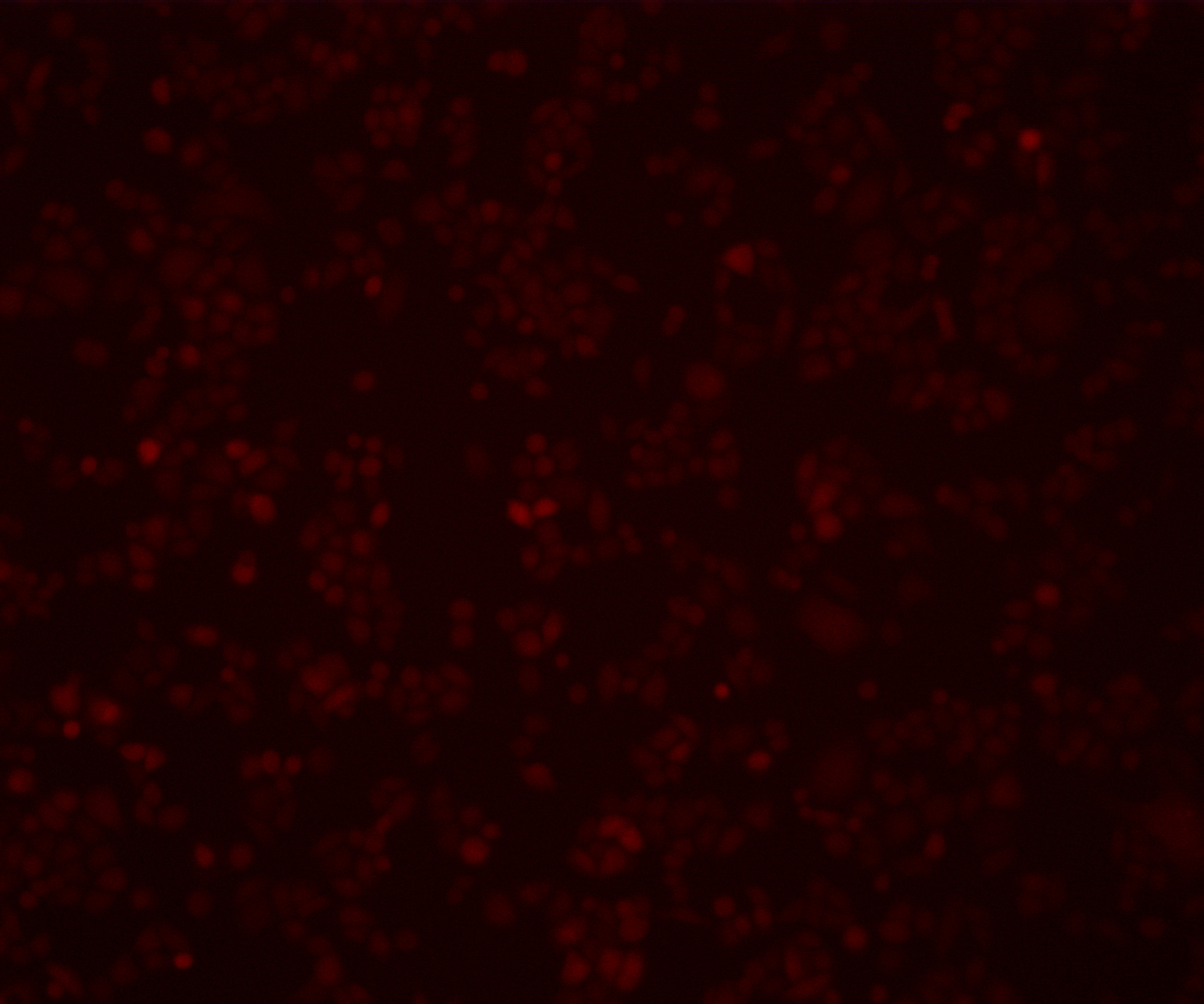

Supplement: S7 File — (ZIP) [file pone.0334639.s007.zip › S 12. File. Original FIgures. Fig.5/5b/BEL-7402 荧光对比图/BEL-7402 sh-CXCL3--y.jpg]

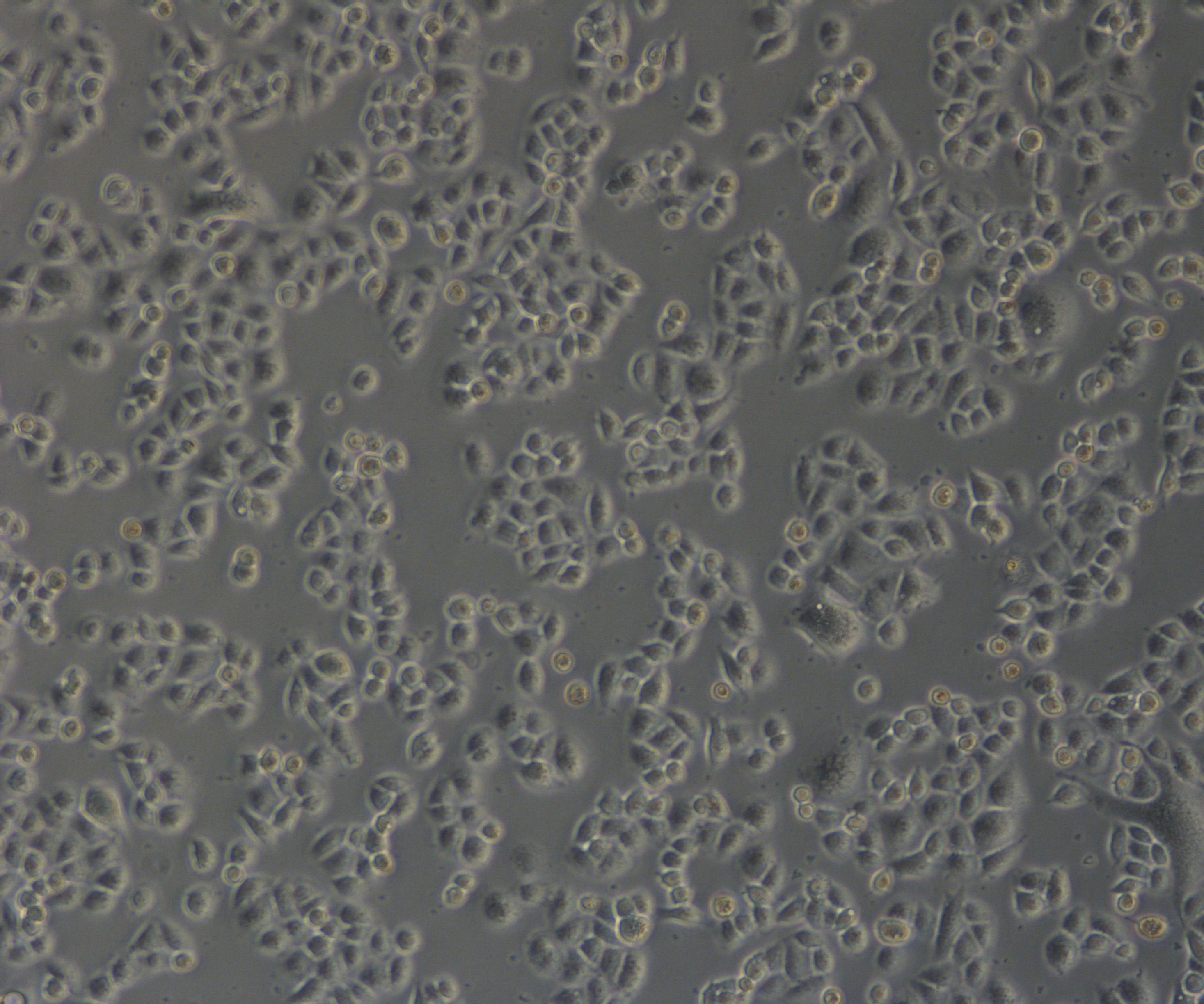

Supplement: S7 File — (ZIP) [file pone.0334639.s007.zip › S 12. File. Original FIgures. Fig.5/5b/BEL-7402 荧光对比图/BEL-7402 sh-CXCL3-Z.jpg]

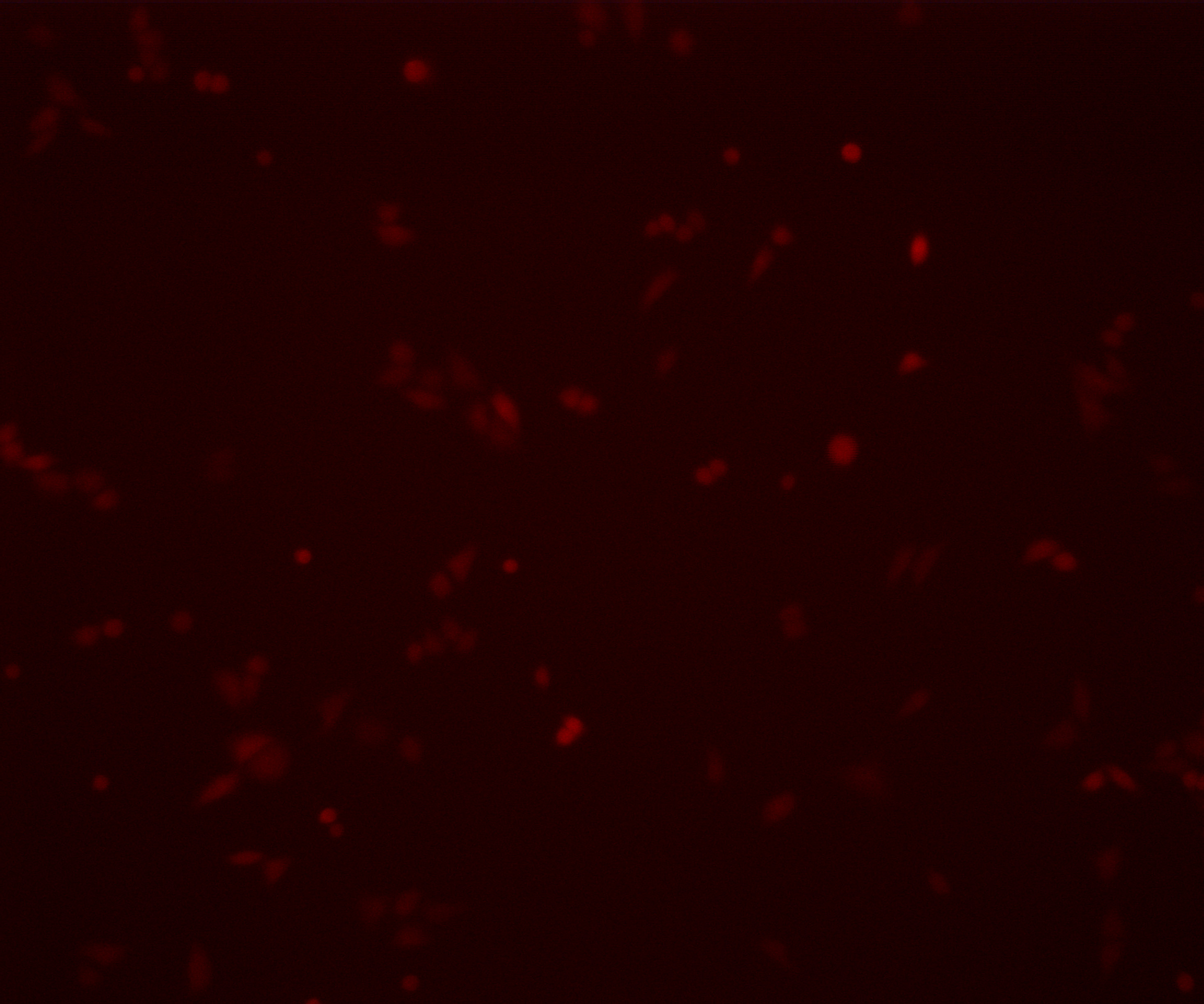

Supplement: S7 File — (ZIP) [file pone.0334639.s007.zip › S 12. File. Original FIgures. Fig.5/5b/BEL-7402 荧光对比图/BEL-7402 sh-NC-Y.jpg]

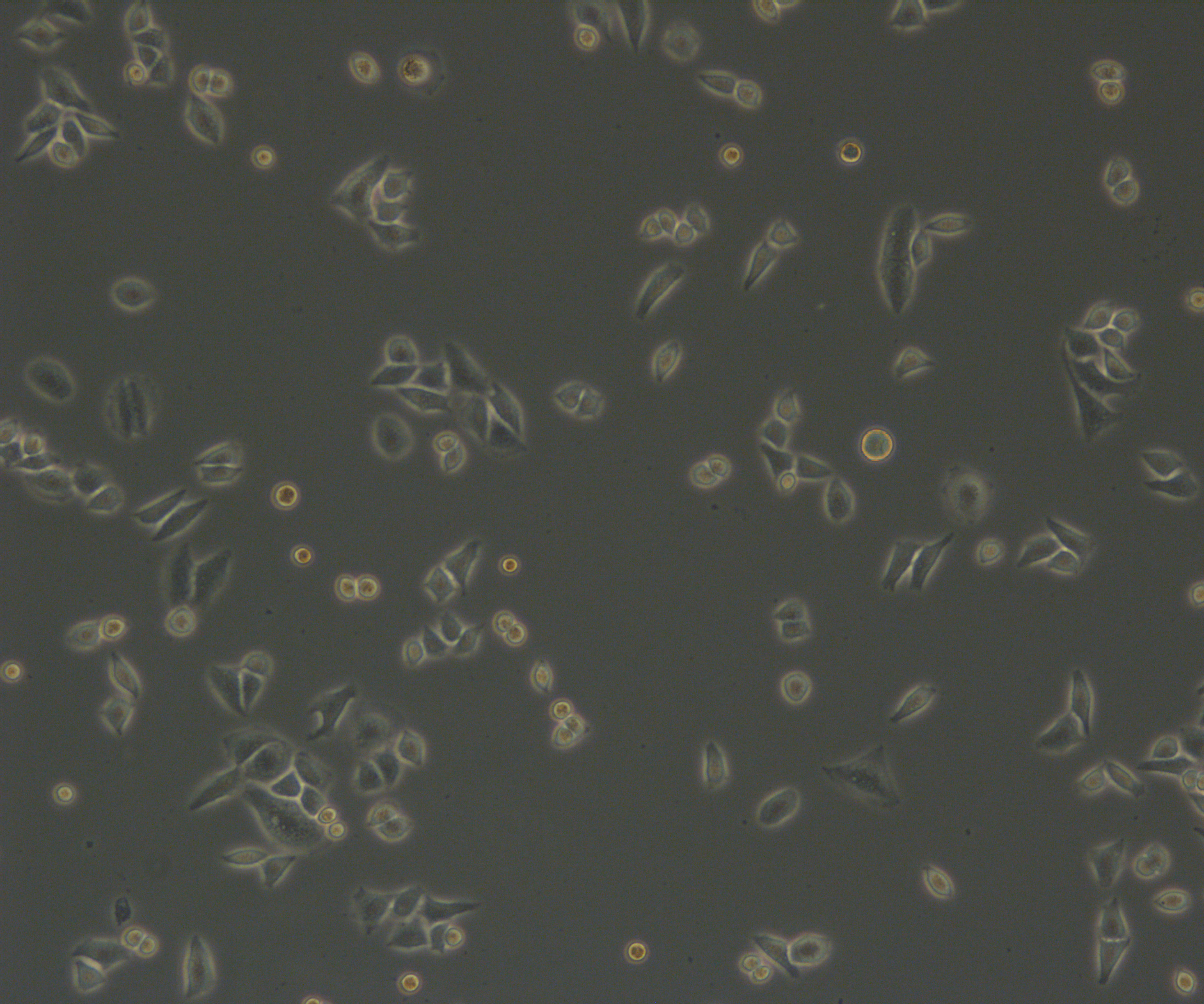

Supplement: S7 File — (ZIP) [file pone.0334639.s007.zip › S 12. File. Original FIgures. Fig.5/5b/BEL-7402 荧光对比图/BEL-7402 sh-NC-Z.jpg]

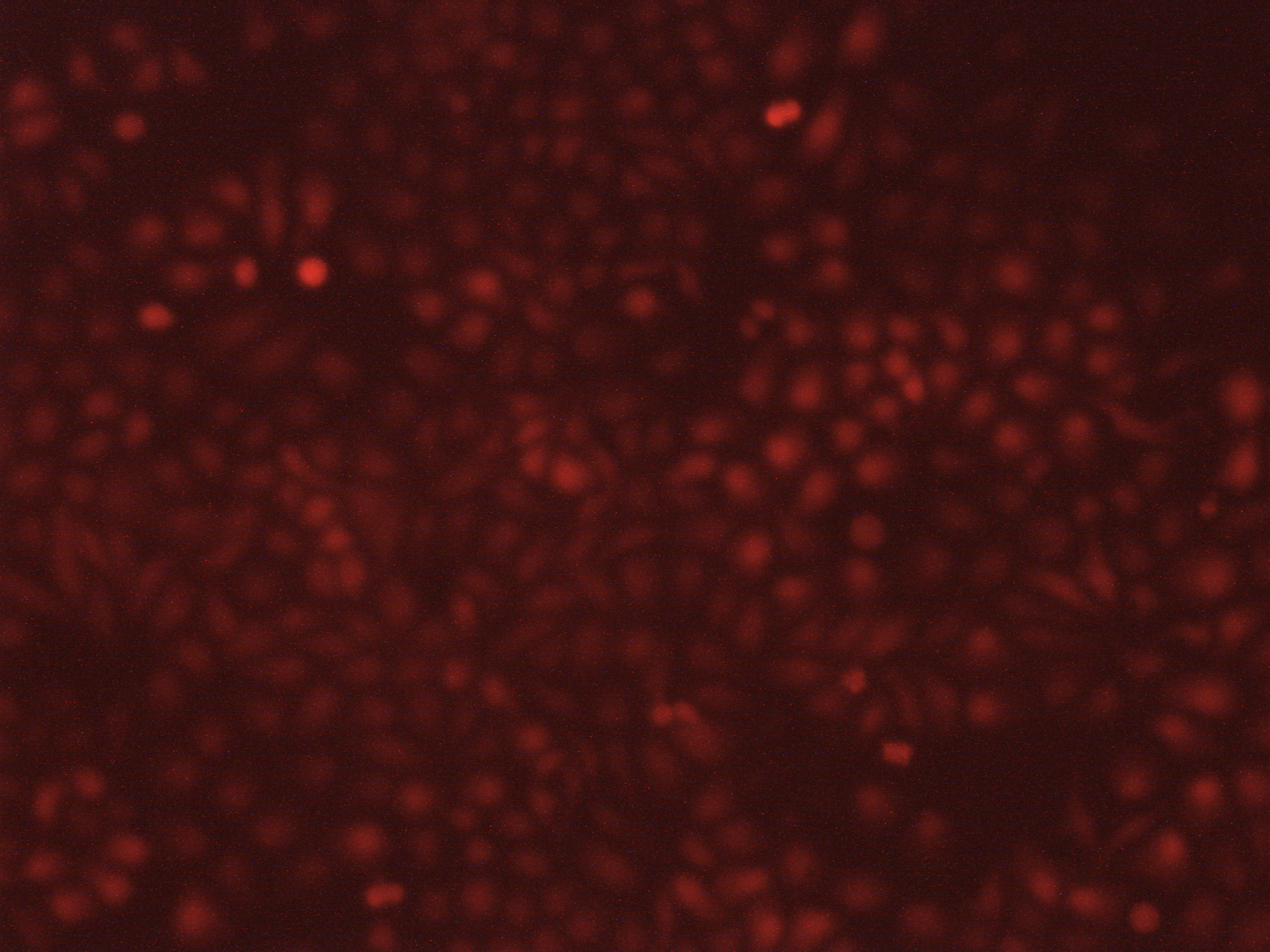

Supplement: S7 File — (ZIP) [file pone.0334639.s007.zip › S 12. File. Original FIgures. Fig.5/5b/Hepg2 荧光对比图/HEPG2 sh-CXCL3 --Y.jpg]

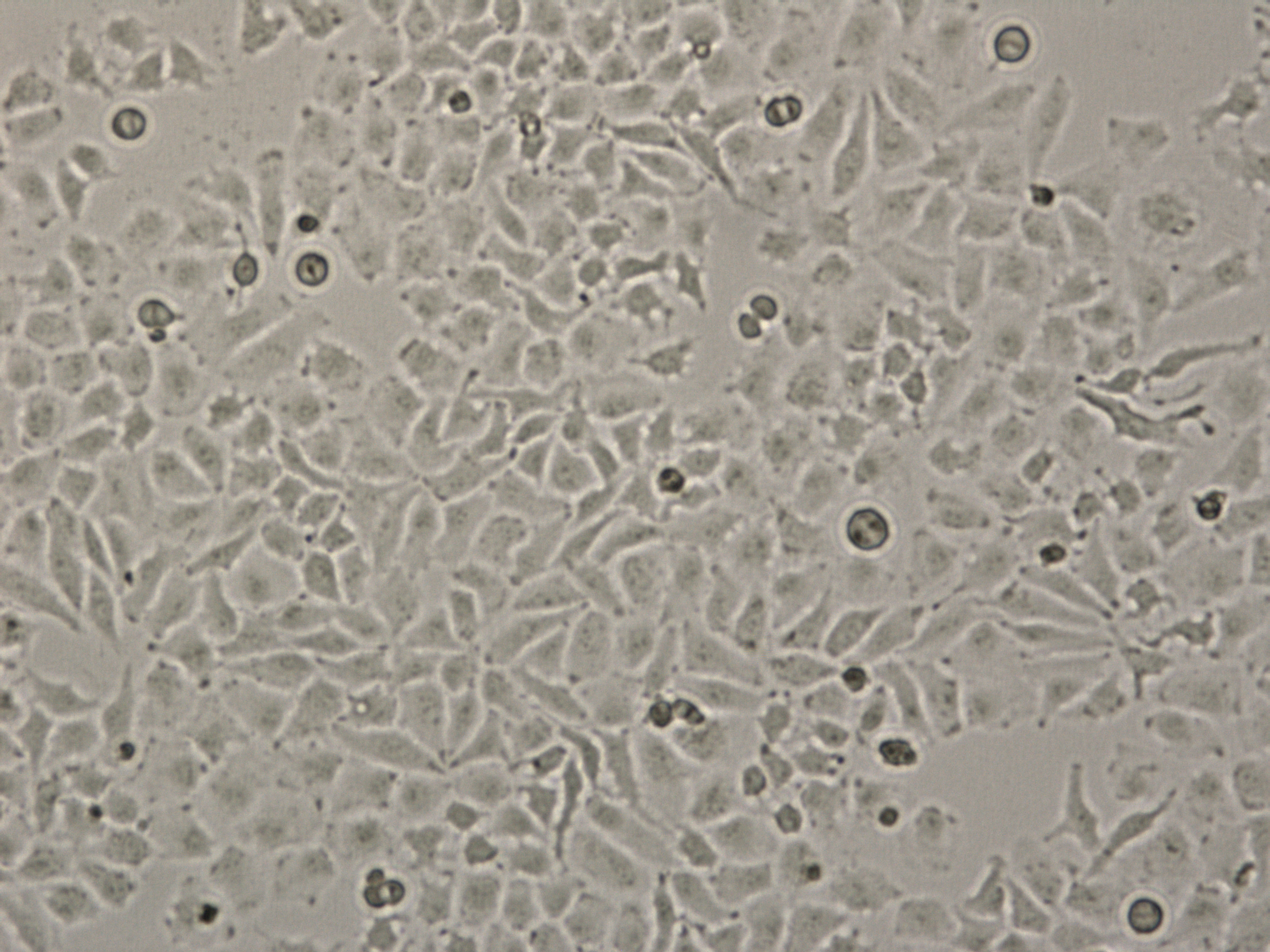

Supplement: S7 File — (ZIP) [file pone.0334639.s007.zip › S 12. File. Original FIgures. Fig.5/5b/Hepg2 荧光对比图/HEPG2 sh-CXCL3-Z.jpg]

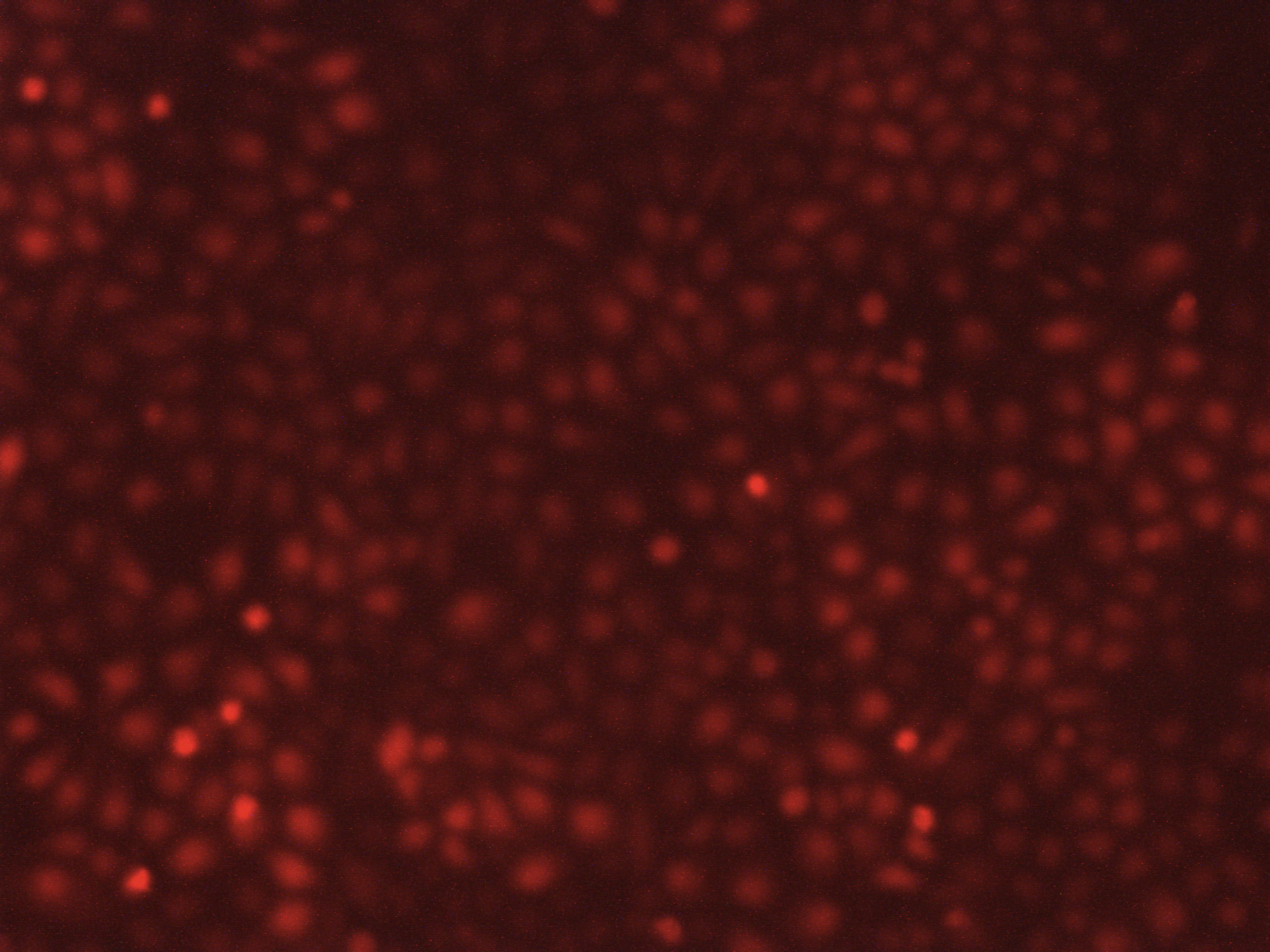

Supplement: S7 File — (ZIP) [file pone.0334639.s007.zip › S 12. File. Original FIgures. Fig.5/5b/Hepg2 荧光对比图/HEPG2 sh-NC-Y.jpg]

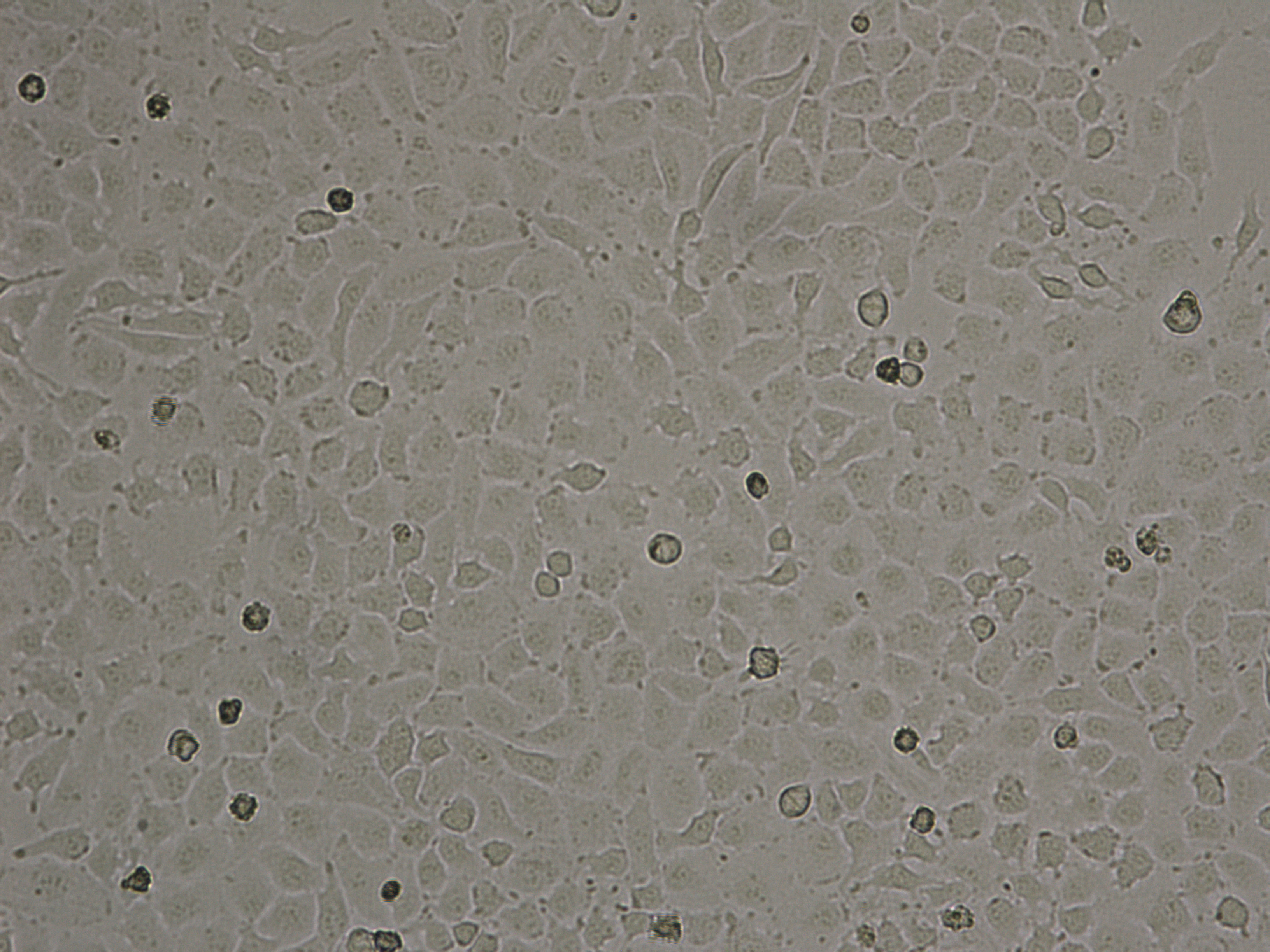

Supplement: S7 File — (ZIP) [file pone.0334639.s007.zip › S 12. File. Original FIgures. Fig.5/5b/Hepg2 荧光对比图/HEPG2 sh-NC-Z.jpg]

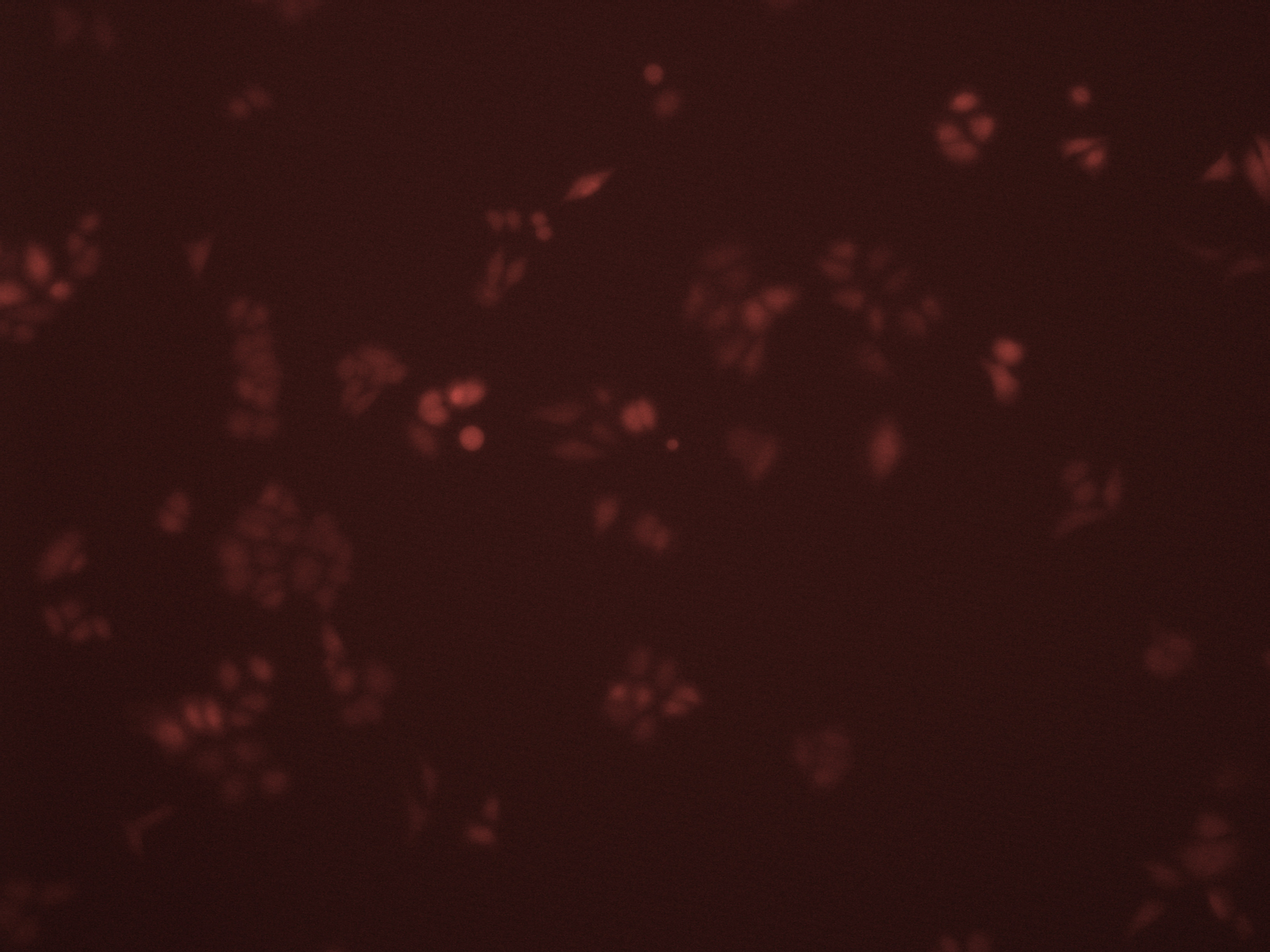

Supplement: S7 File — (ZIP) [file pone.0334639.s007.zip › S 12. File. Original FIgures. Fig.5/5b/SMMC7721荧光对比图/SMMC-7721 sh-CXCL3 -Y.jpg]

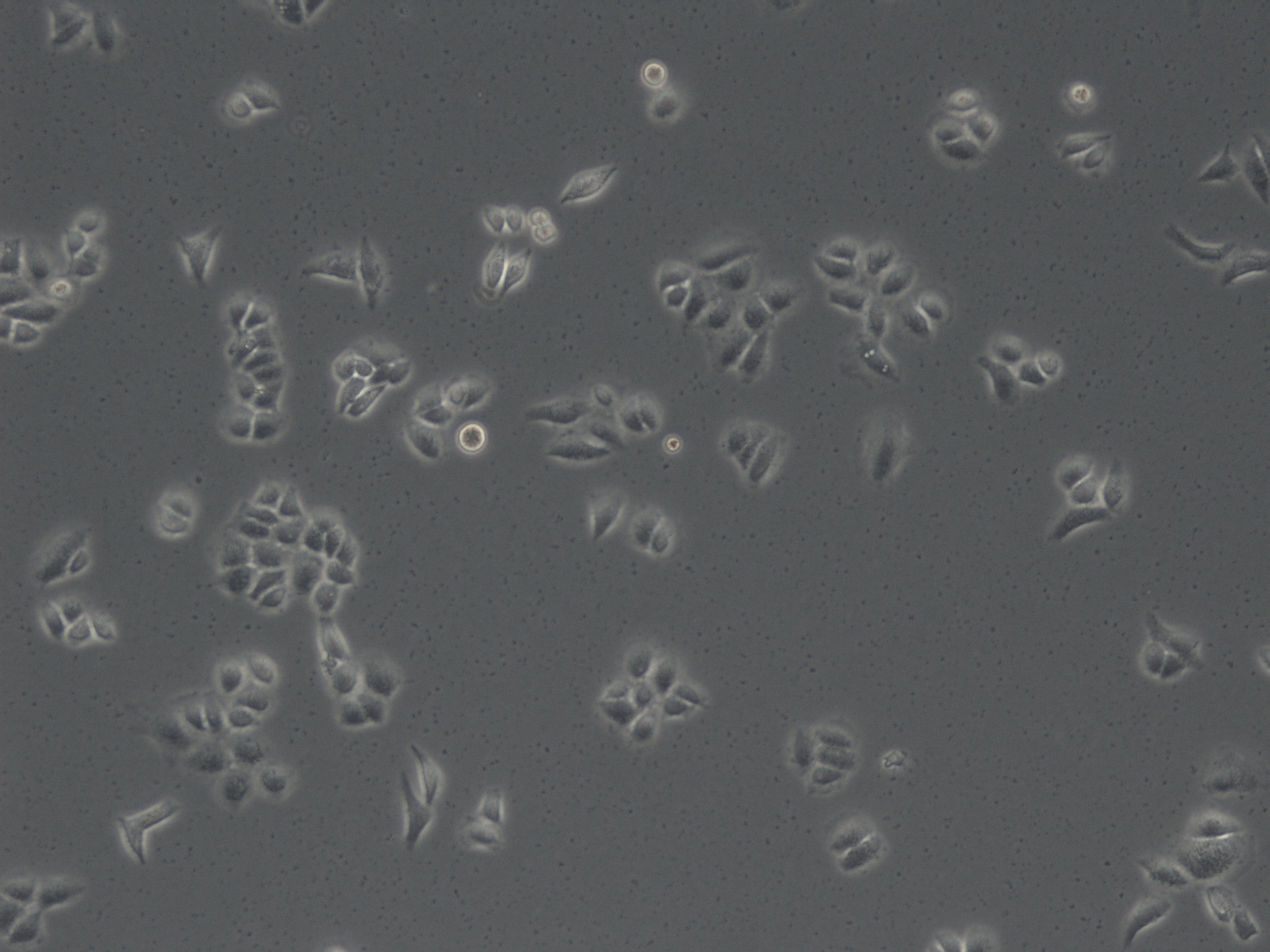

Supplement: S7 File — (ZIP) [file pone.0334639.s007.zip › S 12. File. Original FIgures. Fig.5/5b/SMMC7721荧光对比图/SMMC-7721 sh-CXCL3--Z.jpg]

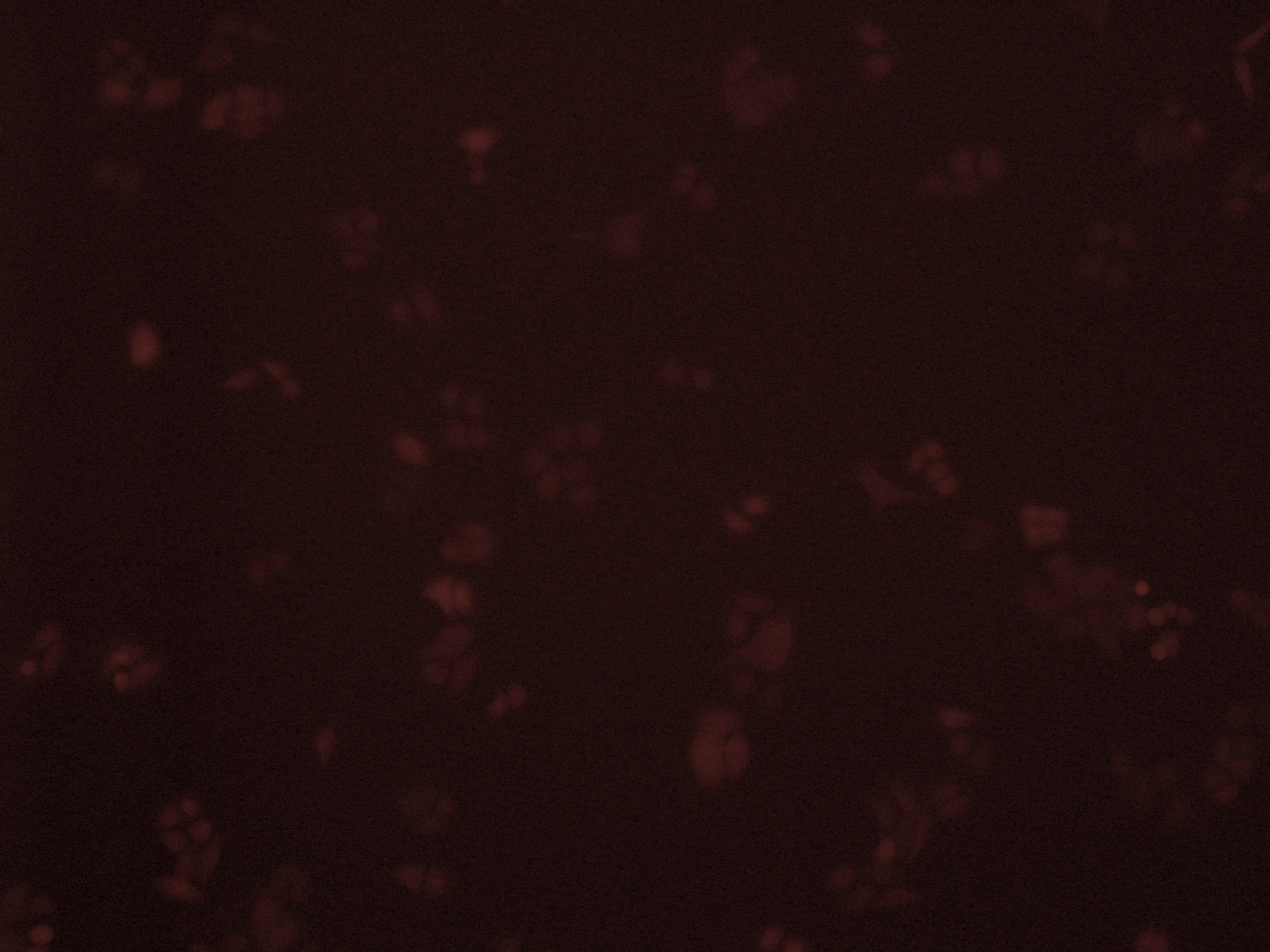

Supplement: S7 File — (ZIP) [file pone.0334639.s007.zip › S 12. File. Original FIgures. Fig.5/5b/SMMC7721荧光对比图/SMMC-7721 sh-NC--Y.jpg]

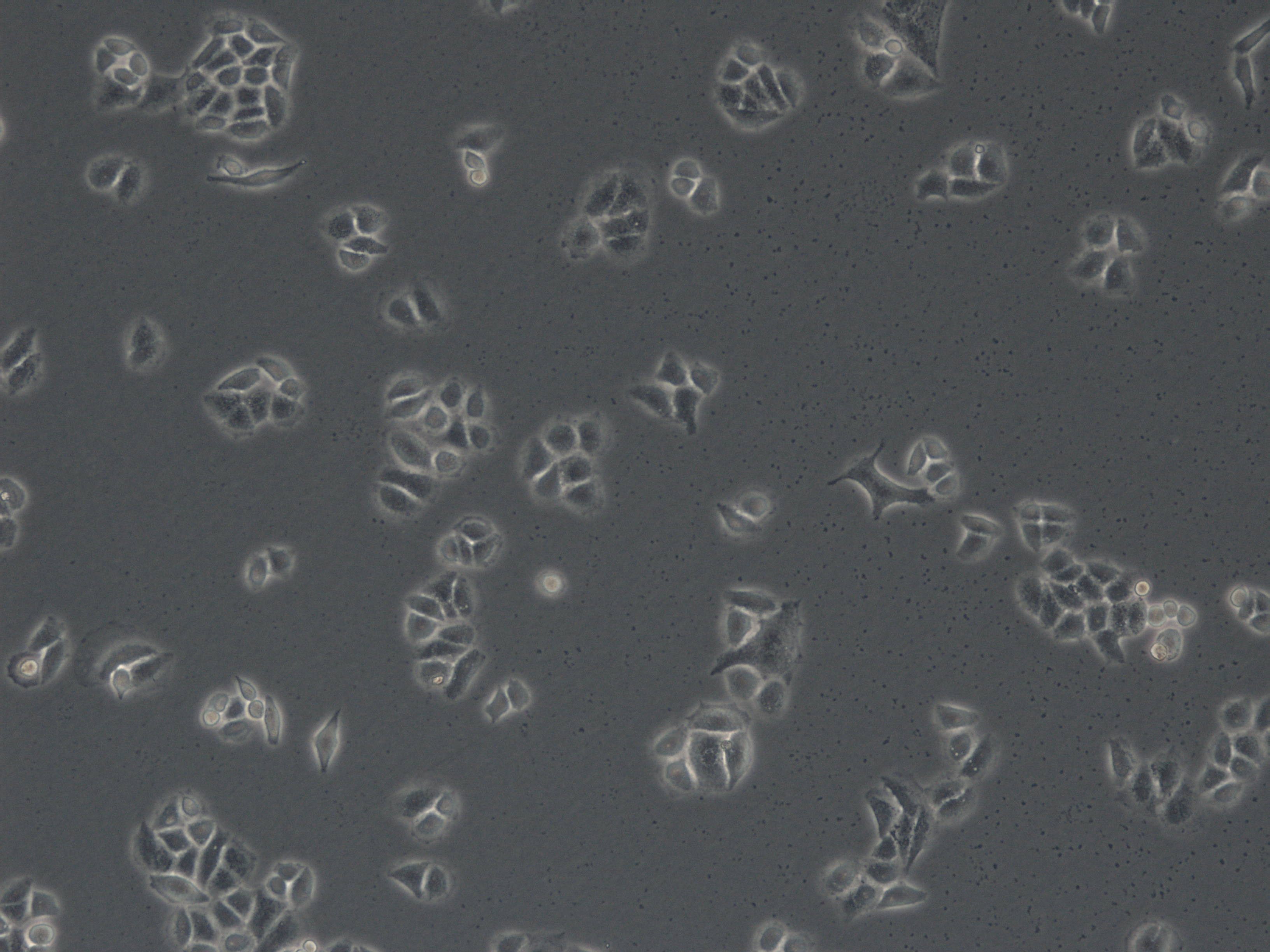

Supplement: S7 File — (ZIP) [file pone.0334639.s007.zip › S 12. File. Original FIgures. Fig.5/5b/SMMC7721荧光对比图/SMMC-7721 sh-NC--Z.jpg]

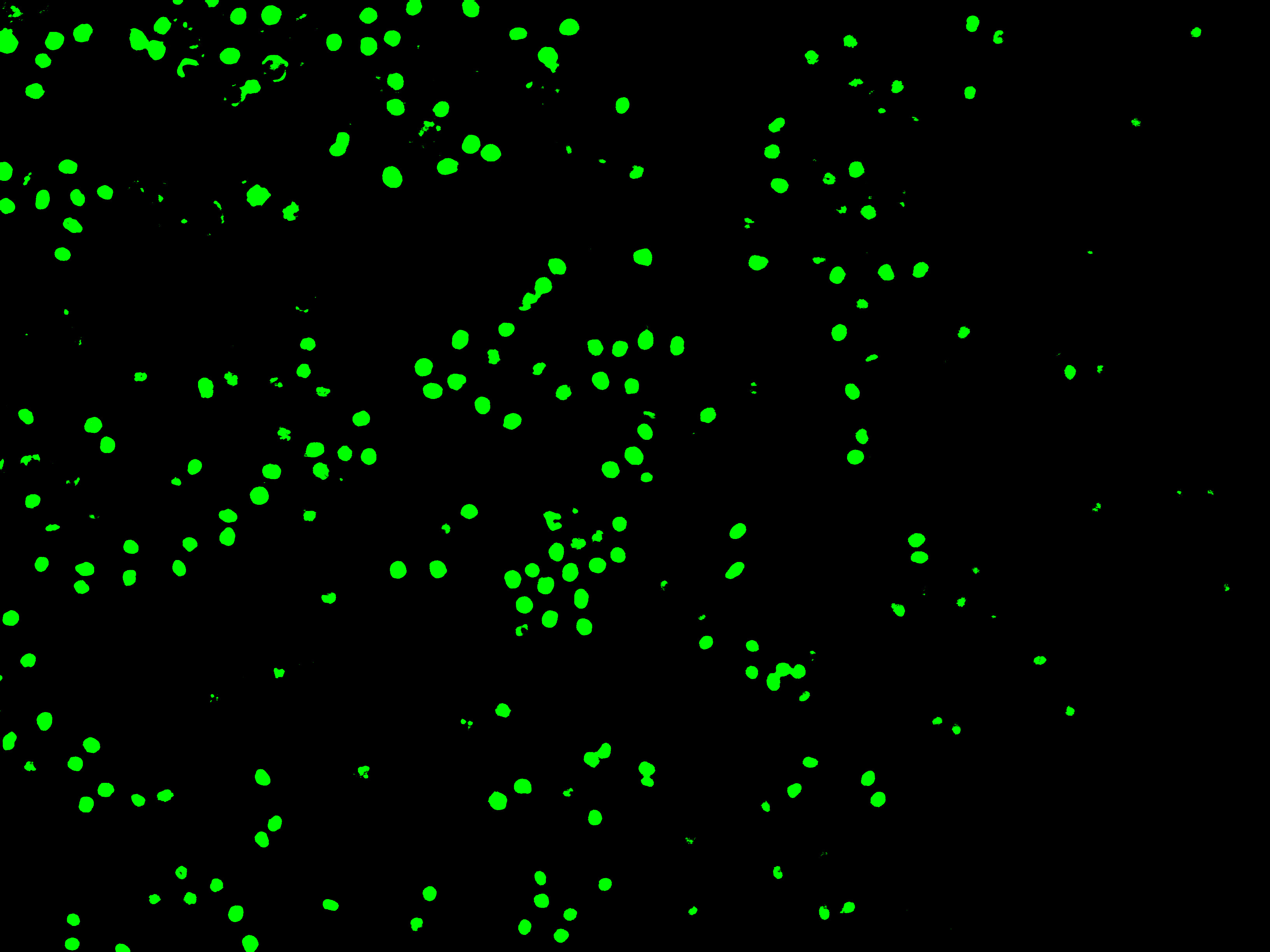

Supplement: S7 File — (ZIP) [file pone.0334639.s007.zip › S 12. File. Original FIgures. Fig.5/5e/BEL-7402 sh-CXCL3 EDU.jpg]

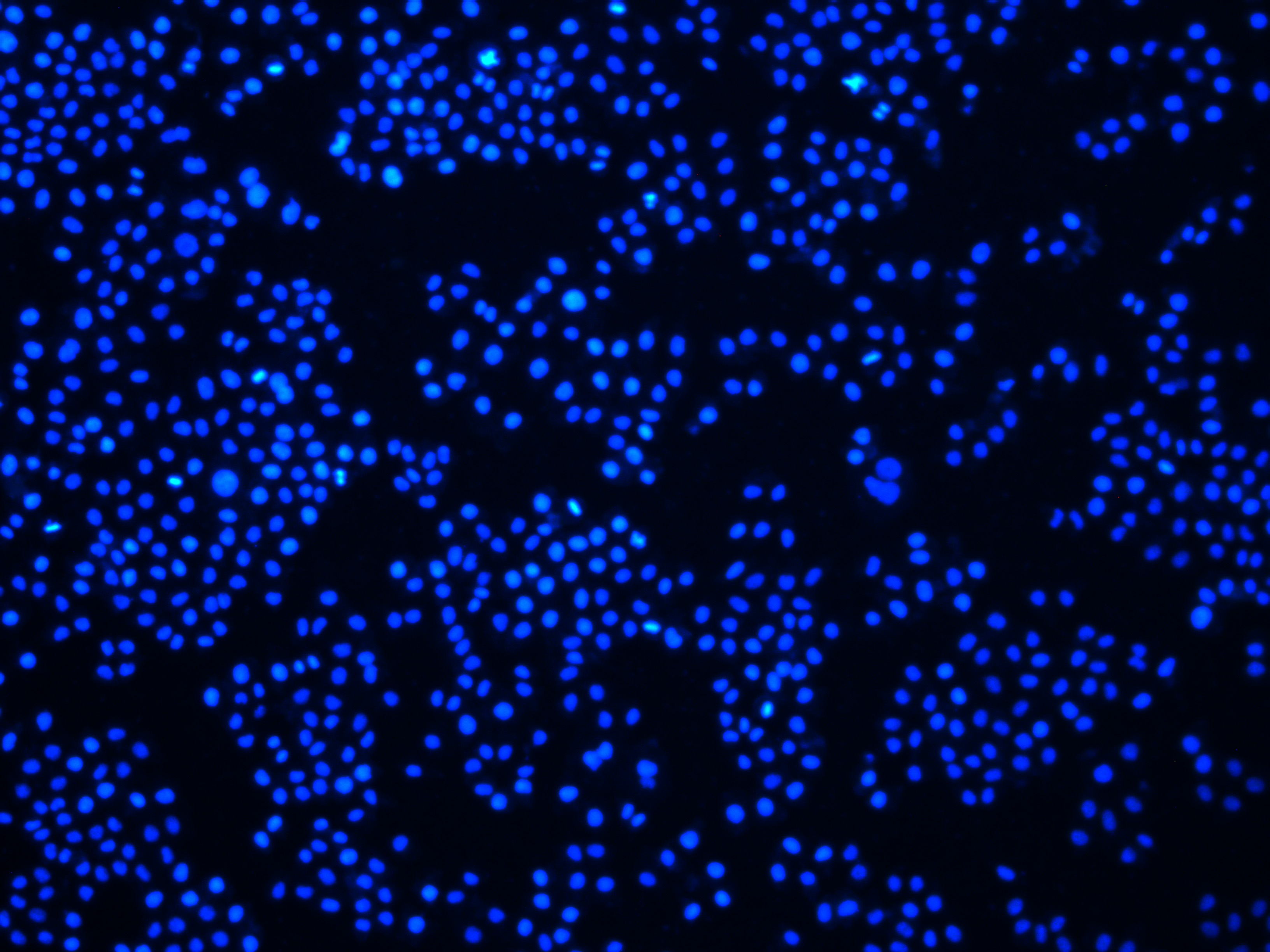

Supplement: S7 File — (ZIP) [file pone.0334639.s007.zip › S 12. File. Original FIgures. Fig.5/5e/BEL-7402 sh-CXCL3 Hoechst33342 .jpg]

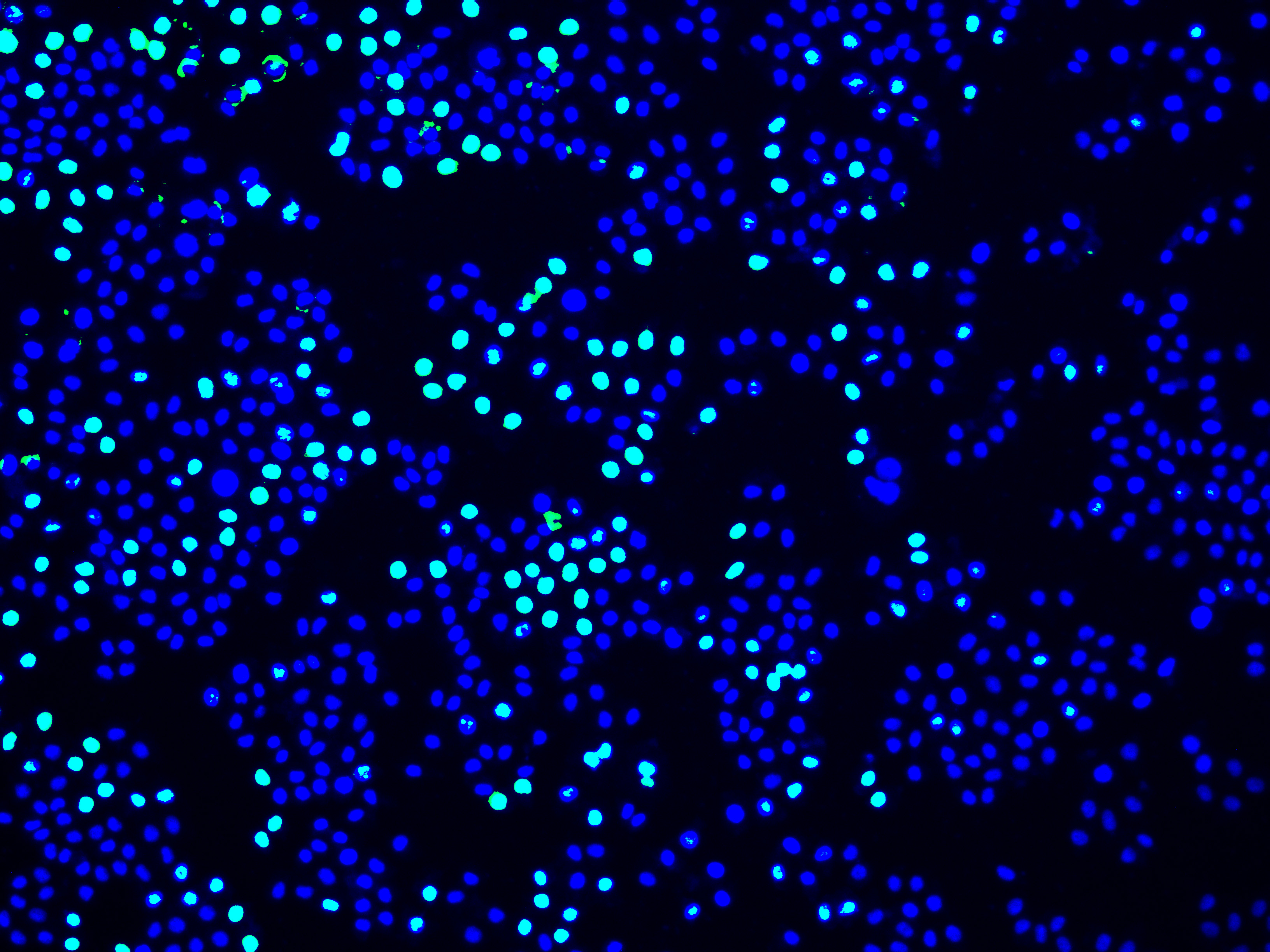

Supplement: S7 File — (ZIP) [file pone.0334639.s007.zip › S 12. File. Original FIgures. Fig.5/5e/BEL-7402 sh-CXCL3 Merge.jpg]

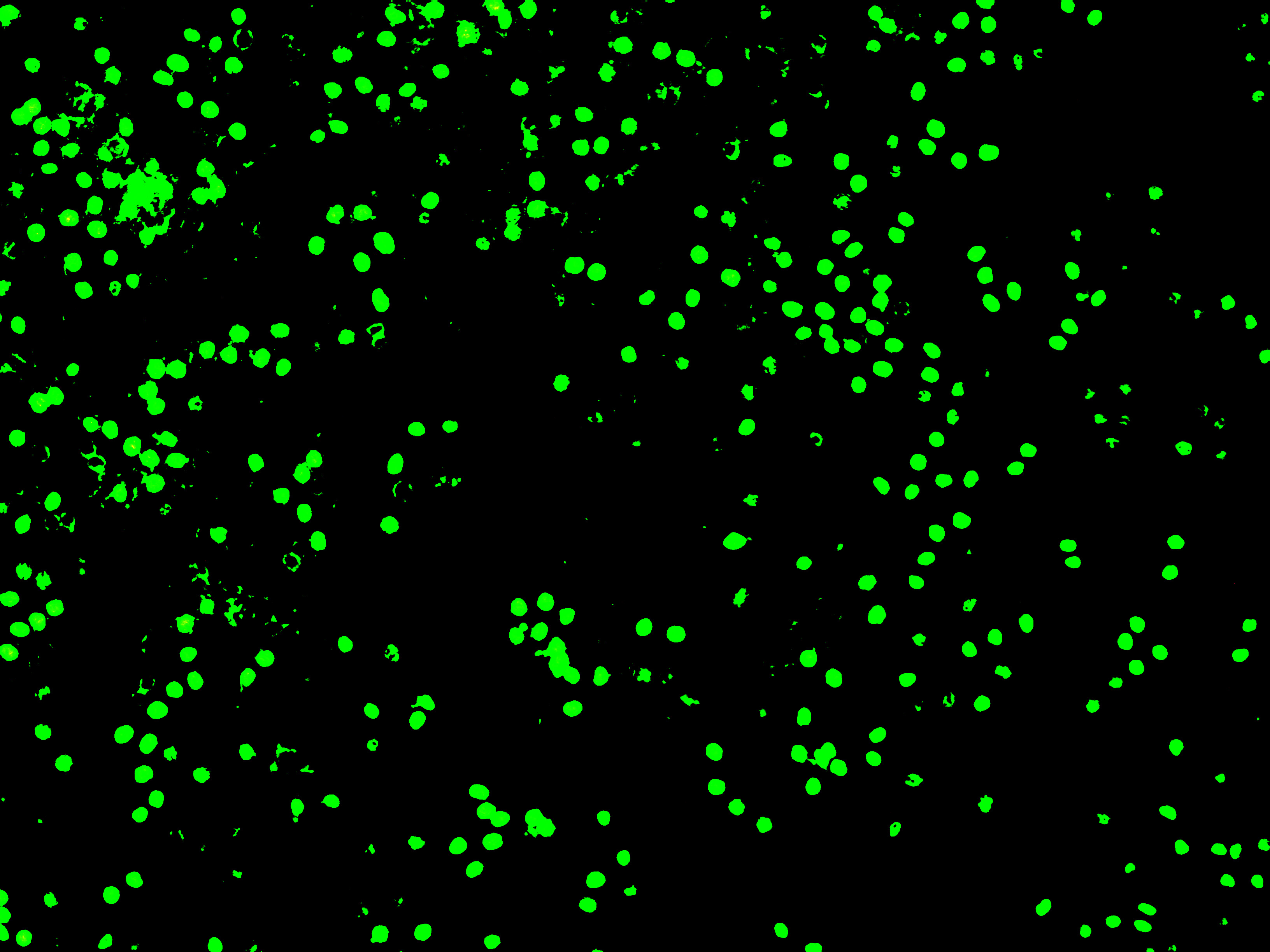

Supplement: S7 File — (ZIP) [file pone.0334639.s007.zip › S 12. File. Original FIgures. Fig.5/5e/BEL-7402 sh-NC EDU.jpg]

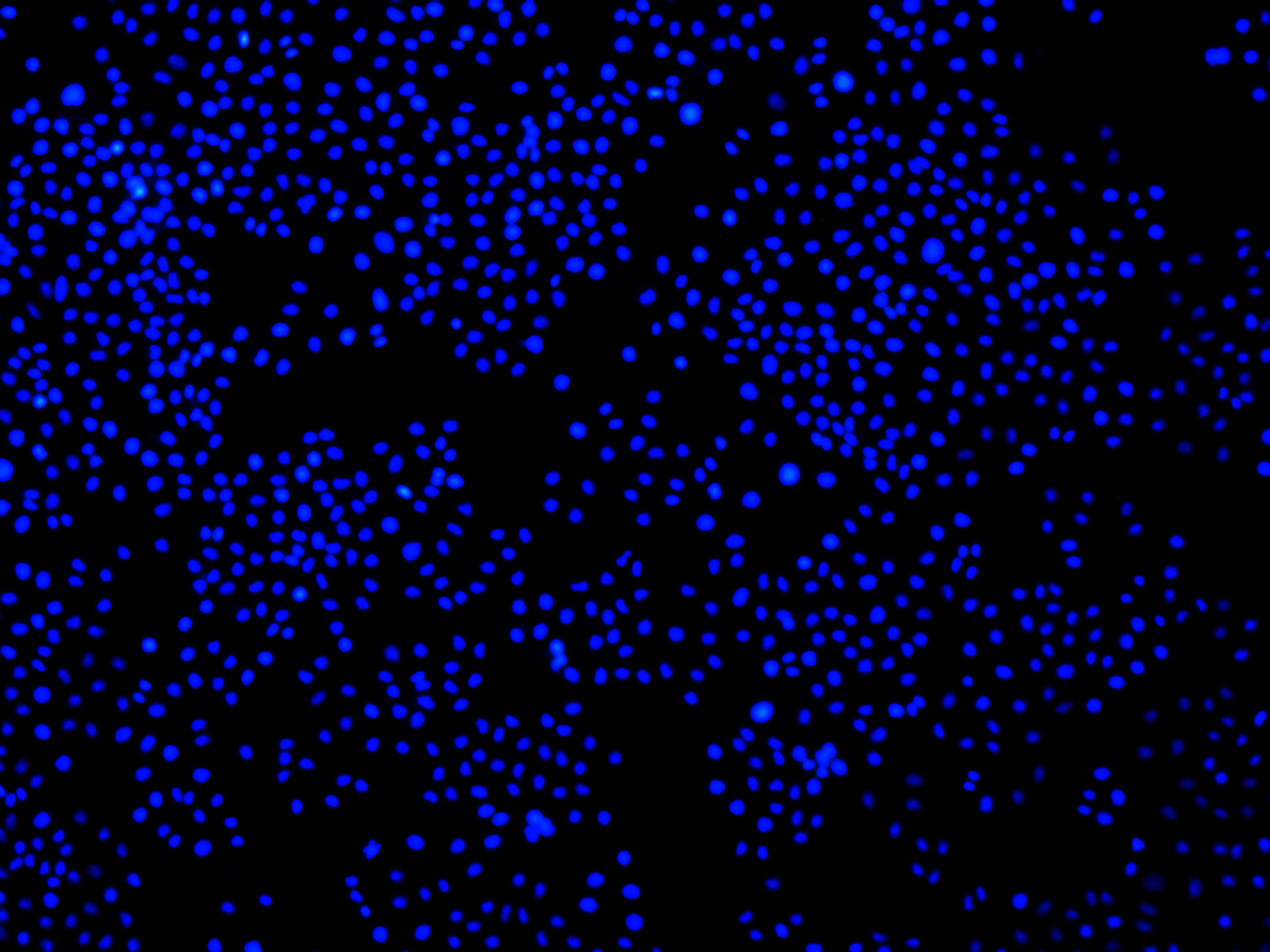

Supplement: S7 File — (ZIP) [file pone.0334639.s007.zip › S 12. File. Original FIgures. Fig.5/5e/BEL-7402 sh-NC Hoechst33342 .jpg]

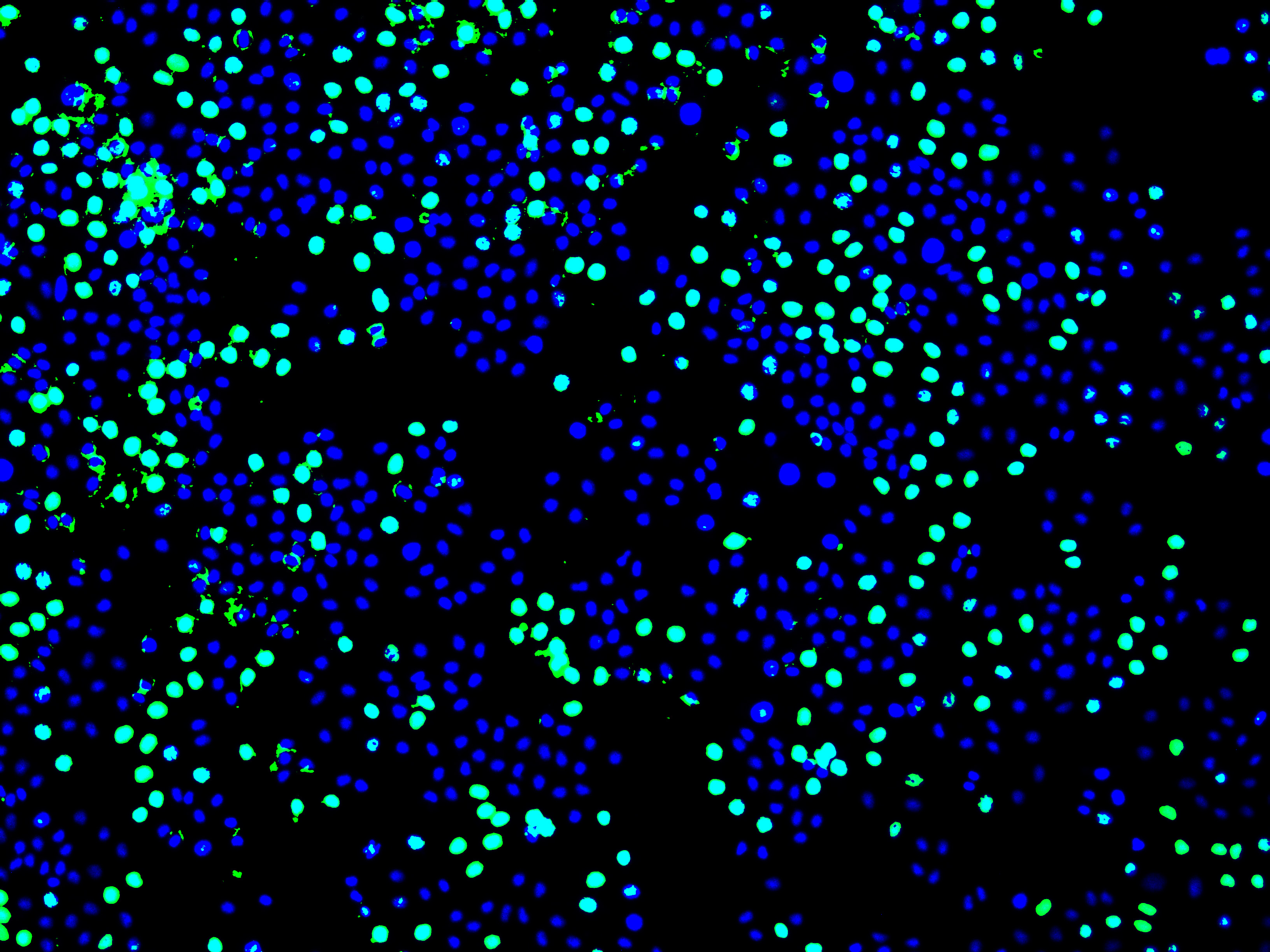

Supplement: S7 File — (ZIP) [file pone.0334639.s007.zip › S 12. File. Original FIgures. Fig.5/5e/BEL-7402 sh-NC--MERGE.jpg]

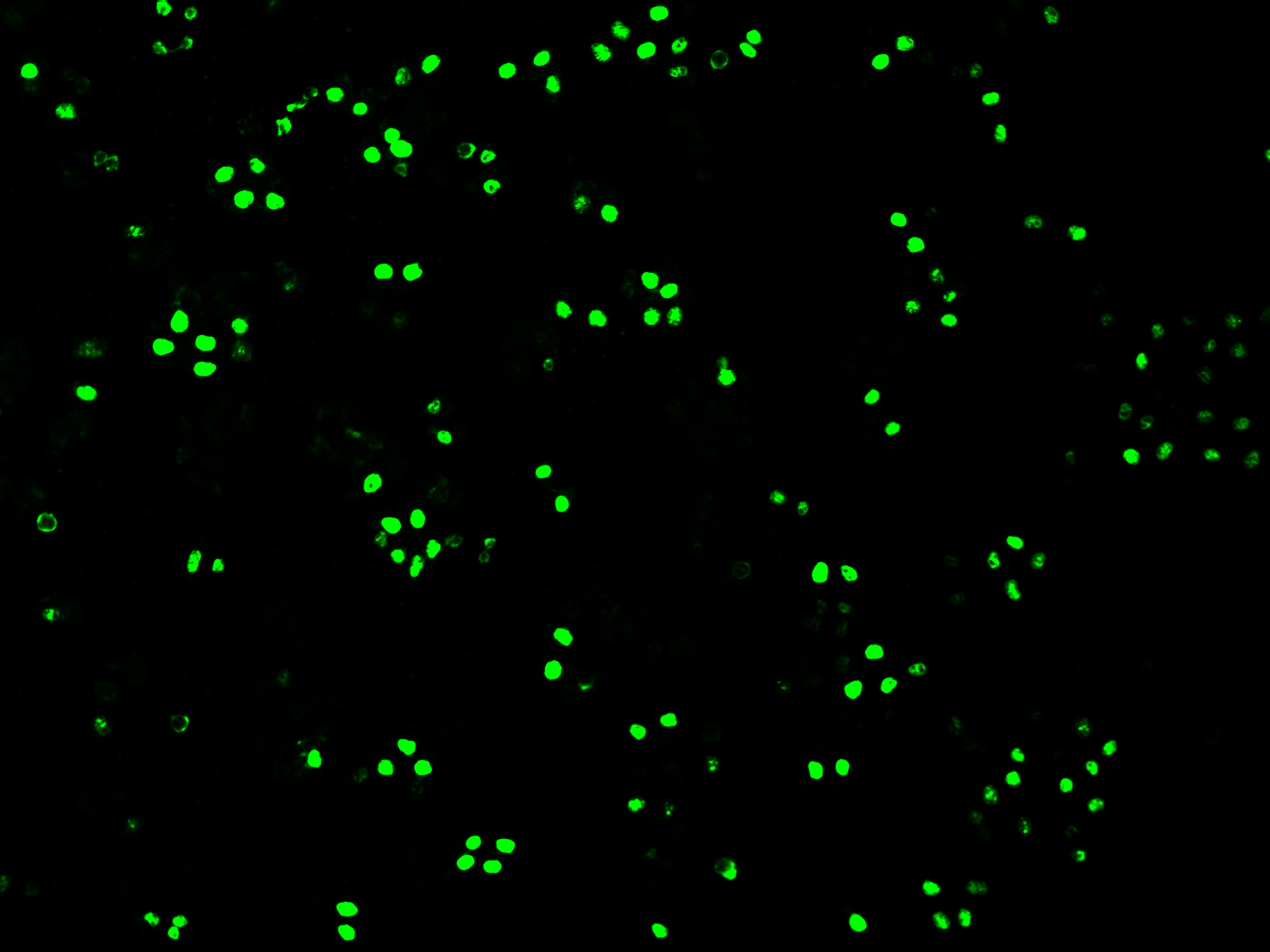

Supplement: S7 File — (ZIP) [file pone.0334639.s007.zip › S 12. File. Original FIgures. Fig.5/5f/HEPG2 sh-CXCL3 EDU.jpg]

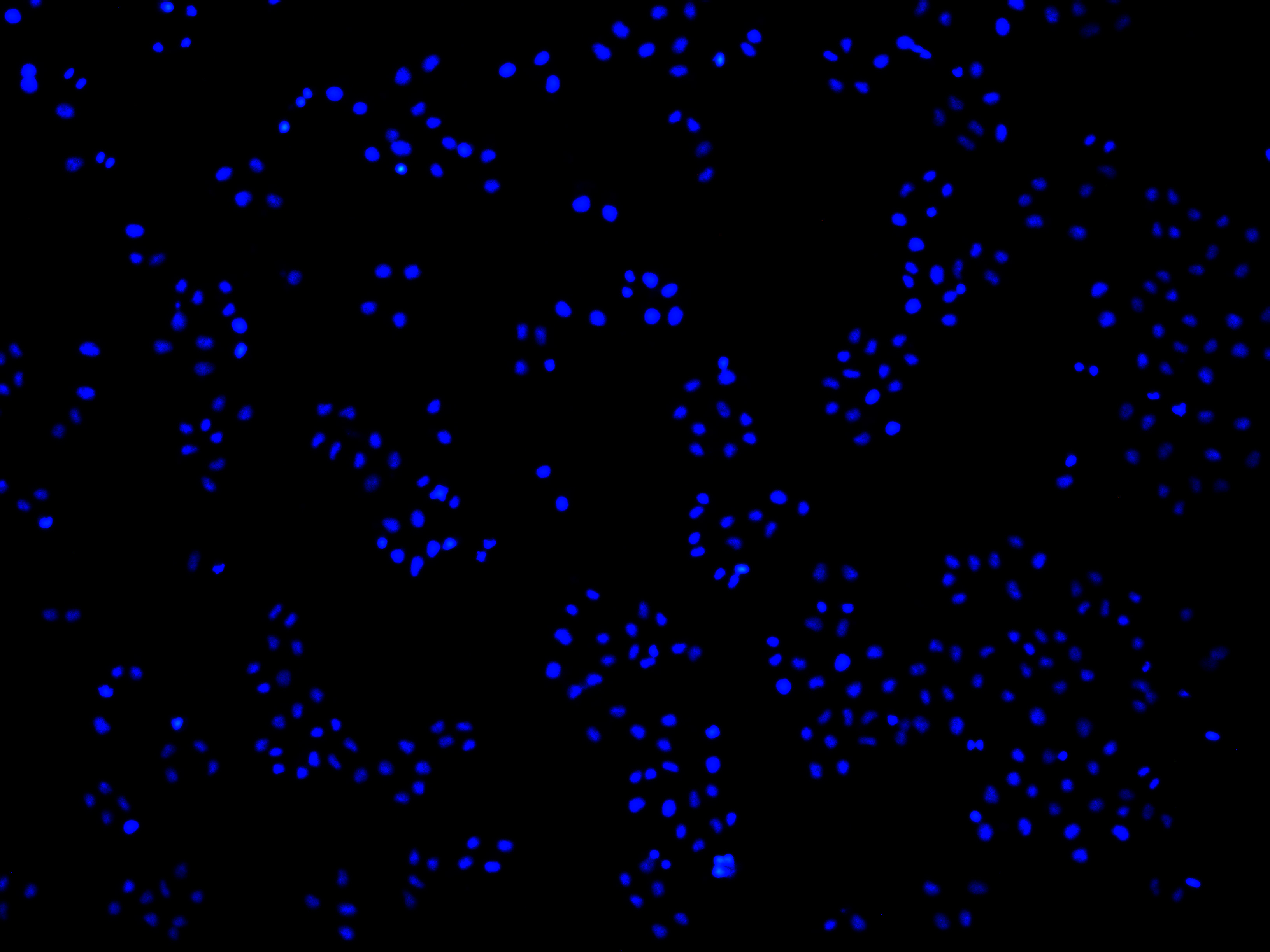

Supplement: S7 File — (ZIP) [file pone.0334639.s007.zip › S 12. File. Original FIgures. Fig.5/5f/HEPG2 sh-CXCL3 Hoechst33342 .jpg]

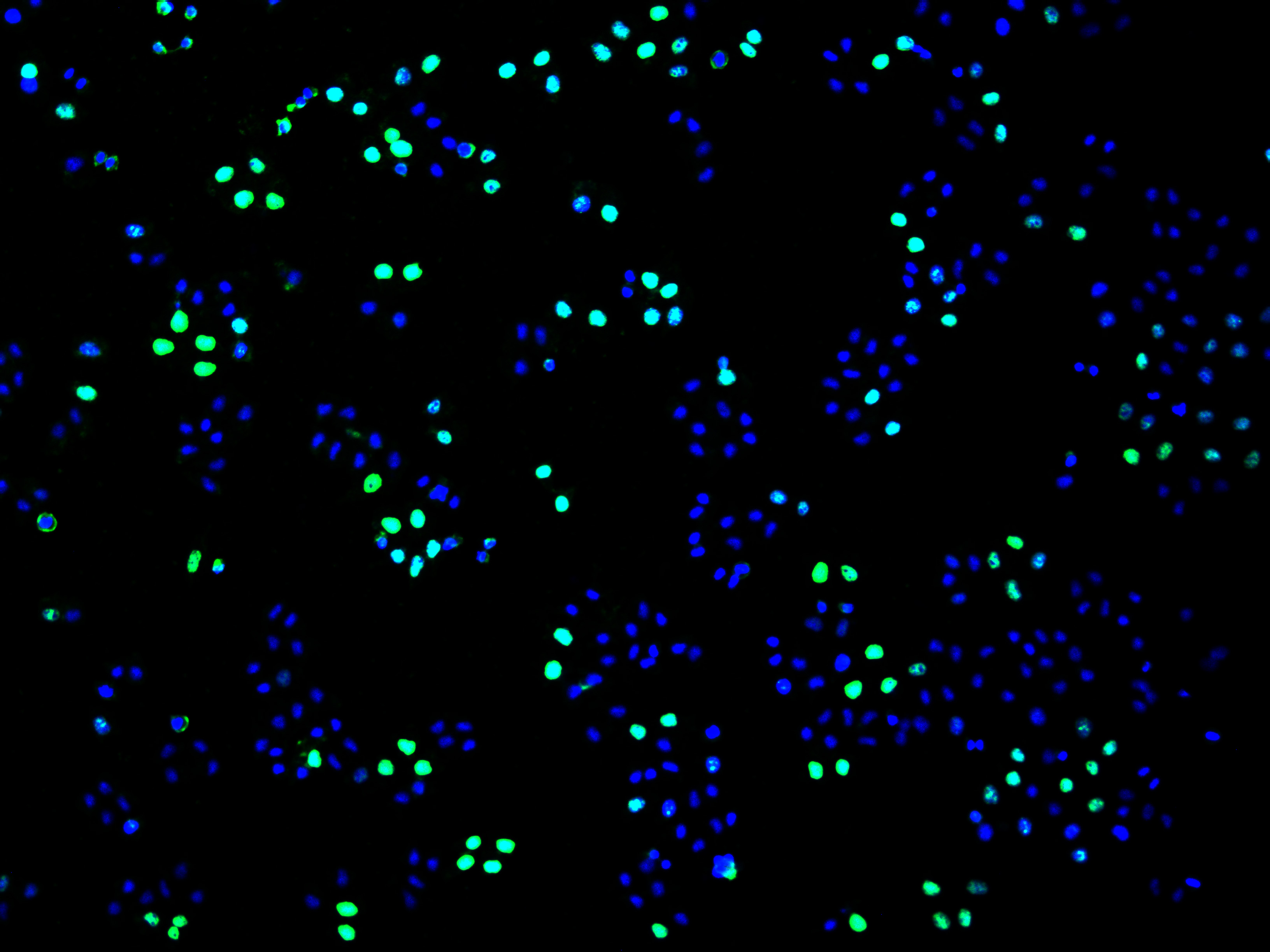

Supplement: S7 File — (ZIP) [file pone.0334639.s007.zip › S 12. File. Original FIgures. Fig.5/5f/HEPG2 sh-CXCL3 MERGE.jpg]

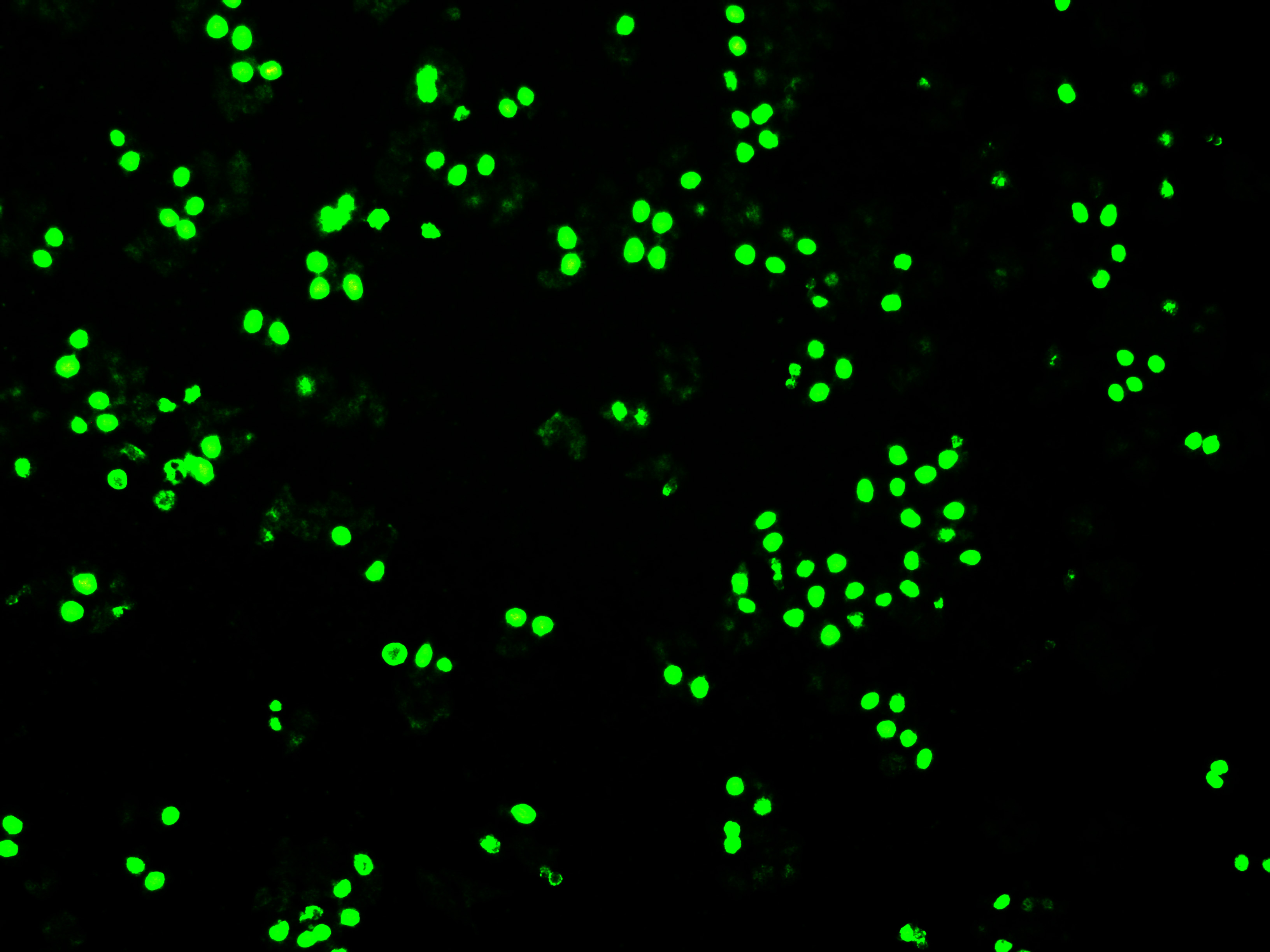

Supplement: S7 File — (ZIP) [file pone.0334639.s007.zip › S 12. File. Original FIgures. Fig.5/5f/HEPG2 sh-NC EDU.jpg]

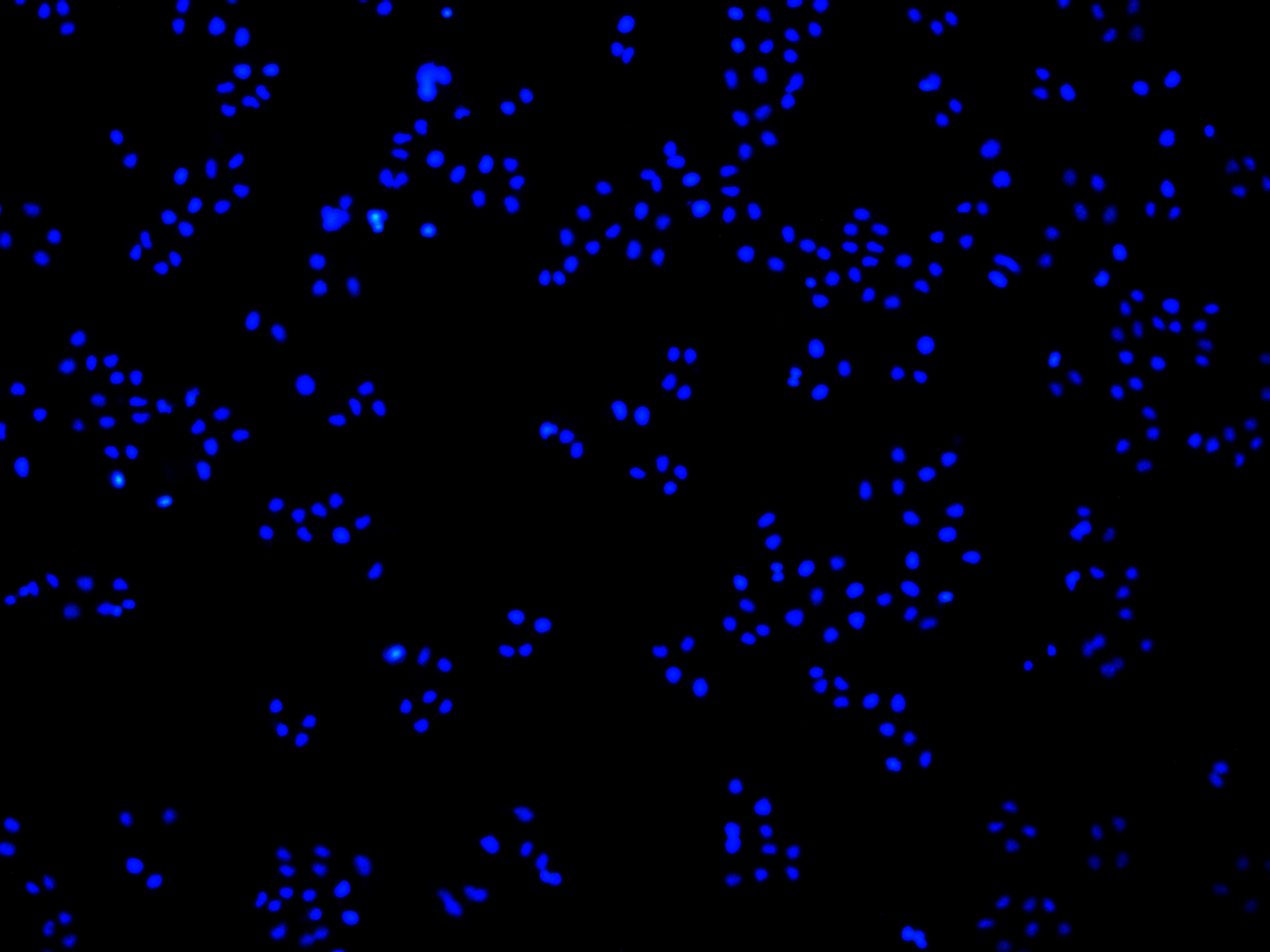

Supplement: S7 File — (ZIP) [file pone.0334639.s007.zip › S 12. File. Original FIgures. Fig.5/5f/HEPG2 sh-NC Hoechst33342 .jpg]

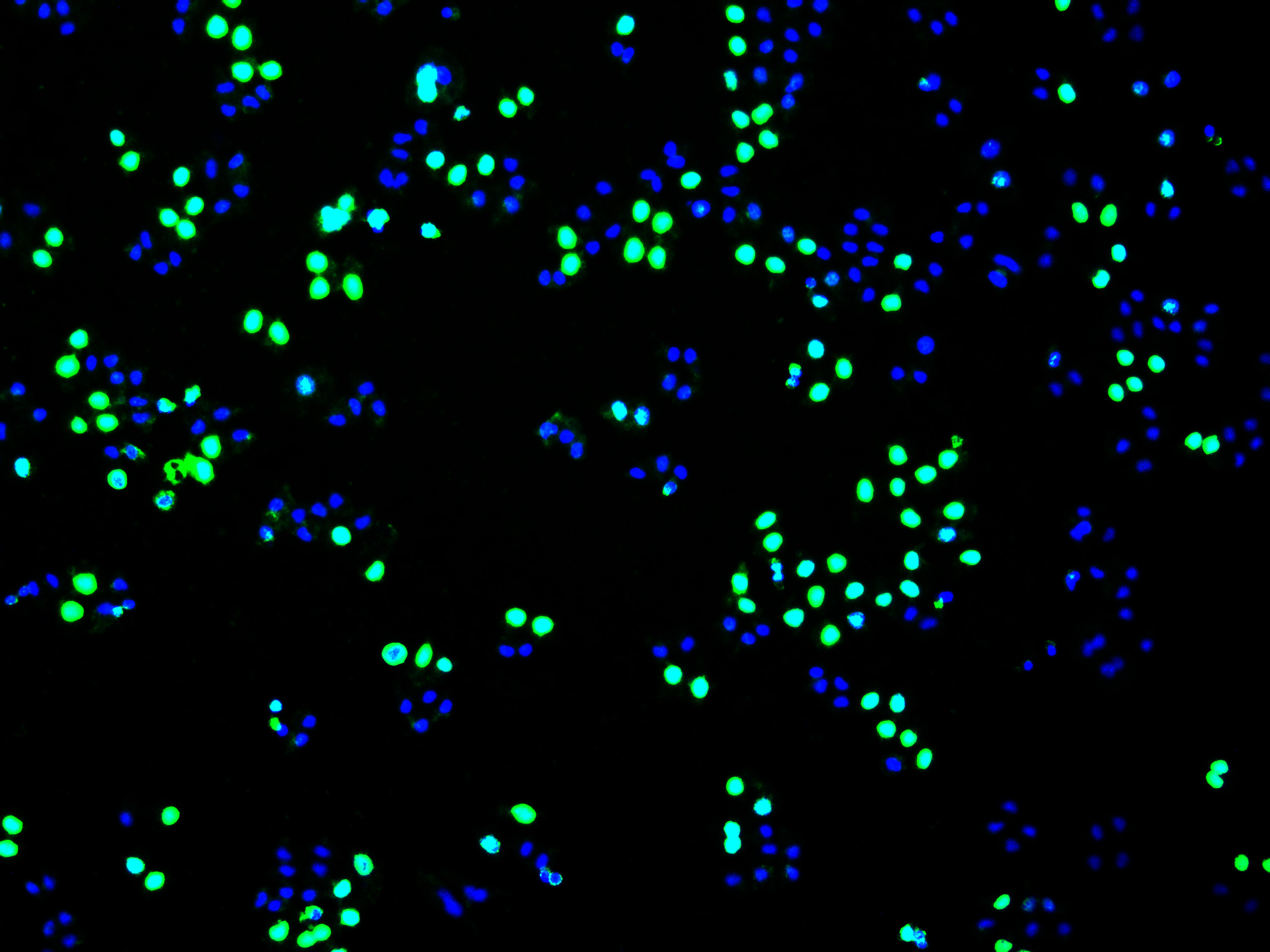

Supplement: S7 File — (ZIP) [file pone.0334639.s007.zip › S 12. File. Original FIgures. Fig.5/5f/HEPG2 sh-NC MERGE.jpg]

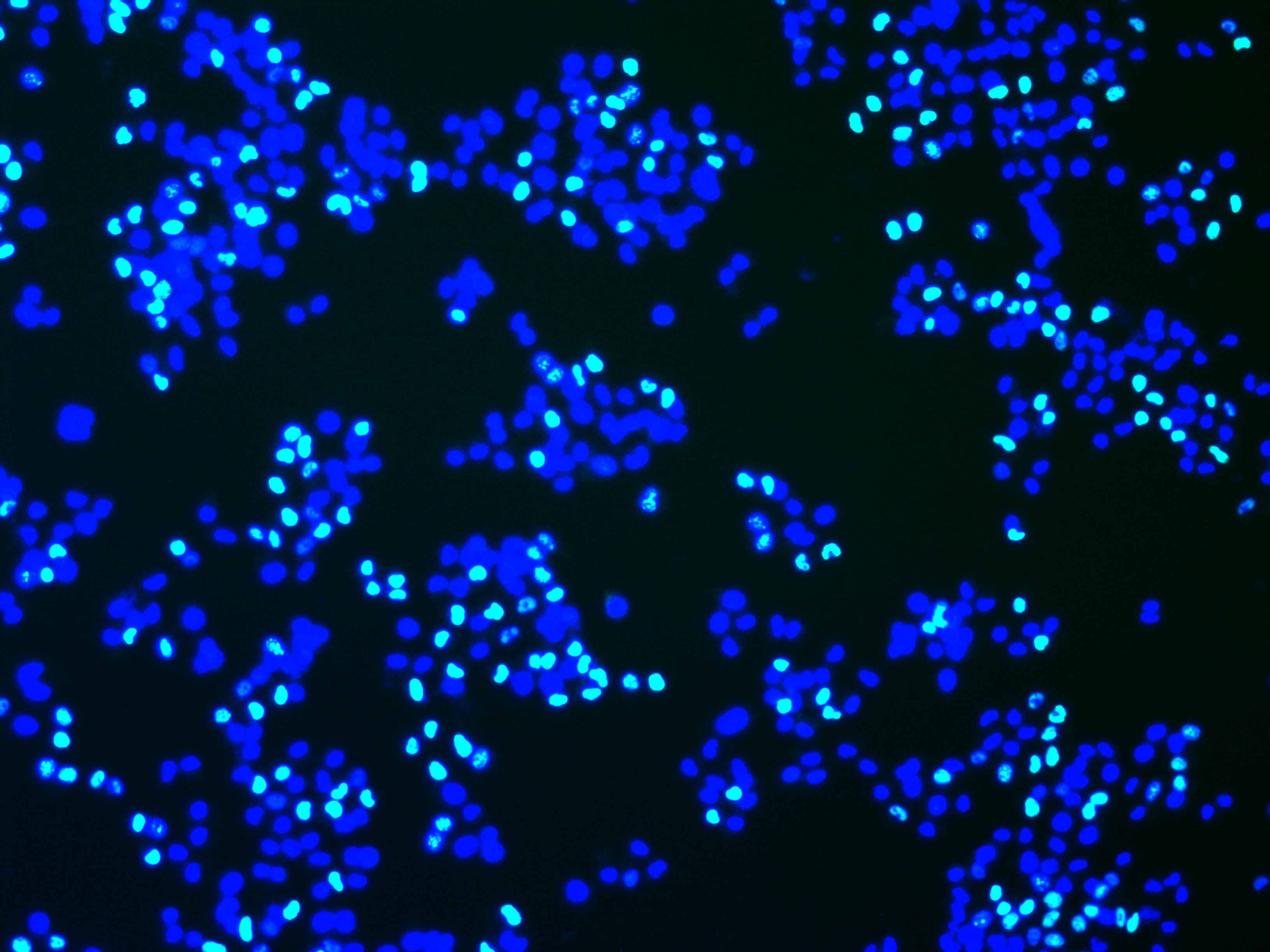

Supplement: S7 File — (ZIP) [file pone.0334639.s007.zip › S 12. File. Original FIgures. Fig.5/5g/SMMC-721 sh-CXCL3 MERGE.jpg]

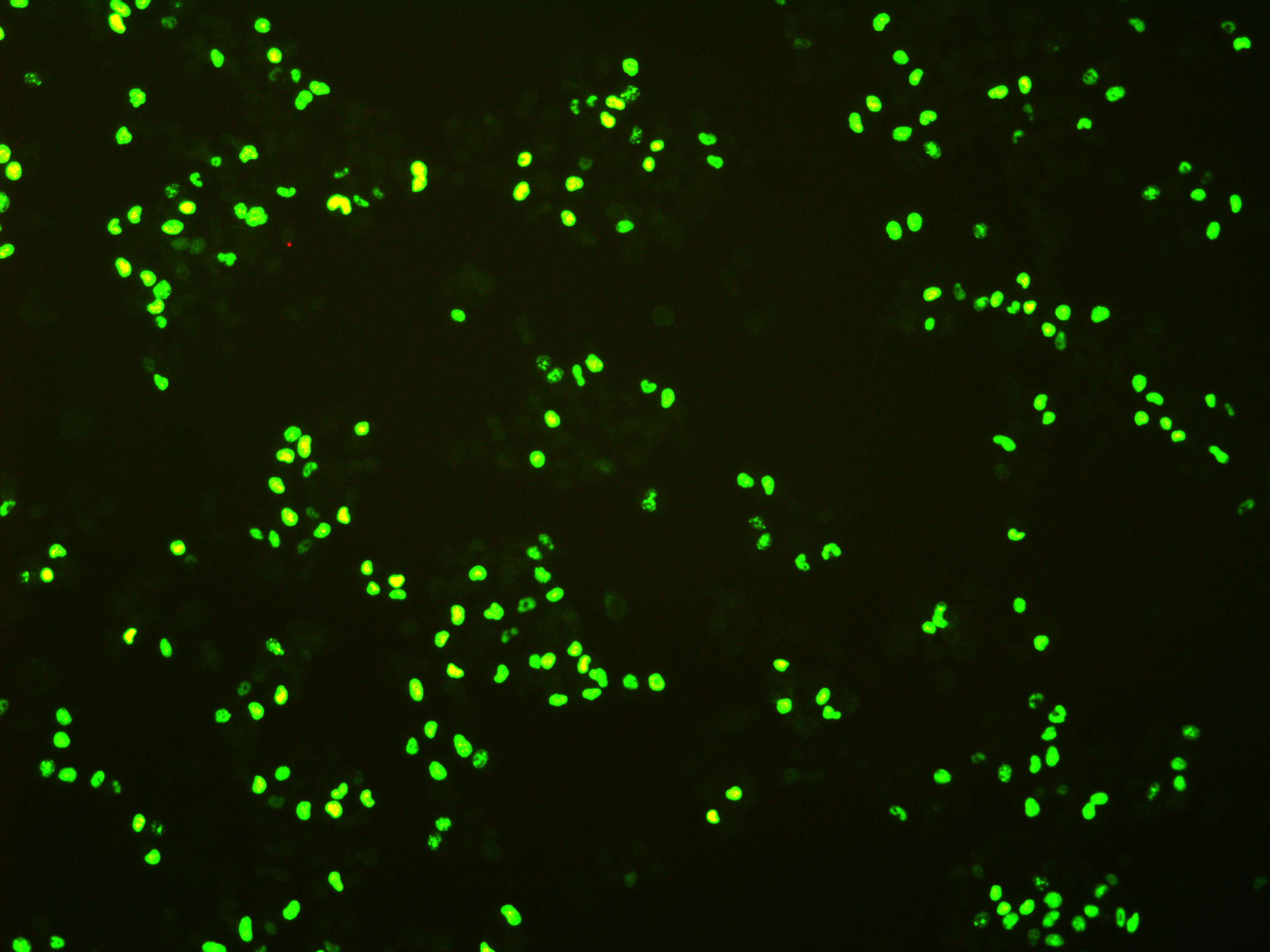

Supplement: S7 File — (ZIP) [file pone.0334639.s007.zip › S 12. File. Original FIgures. Fig.5/5g/SMMC-721 sh-CXCL3 EDU.jpg]

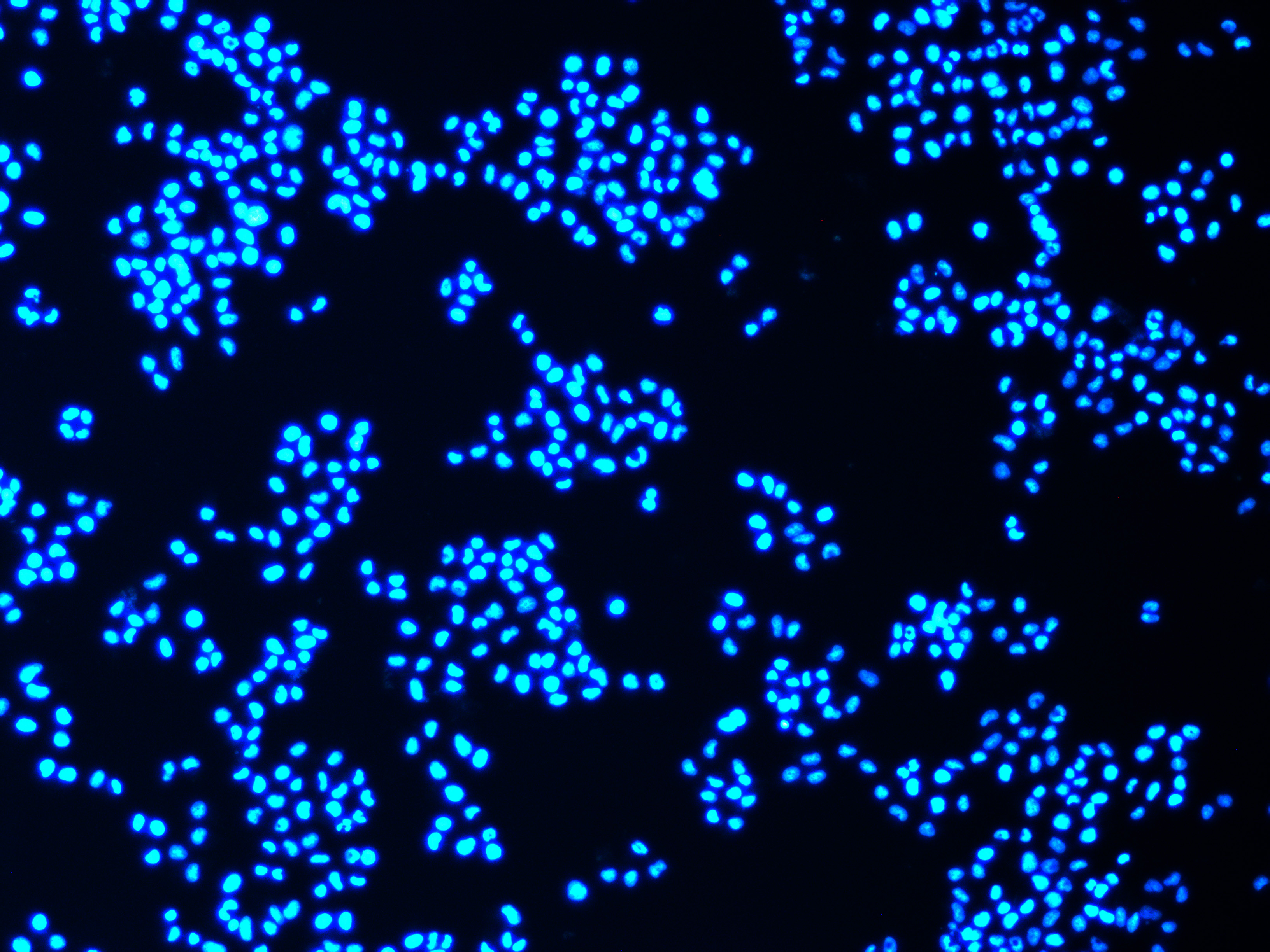

Supplement: S7 File — (ZIP) [file pone.0334639.s007.zip › S 12. File. Original FIgures. Fig.5/5g/SMMC-721 sh-CXCL3 Hoechst33342 .jpg]

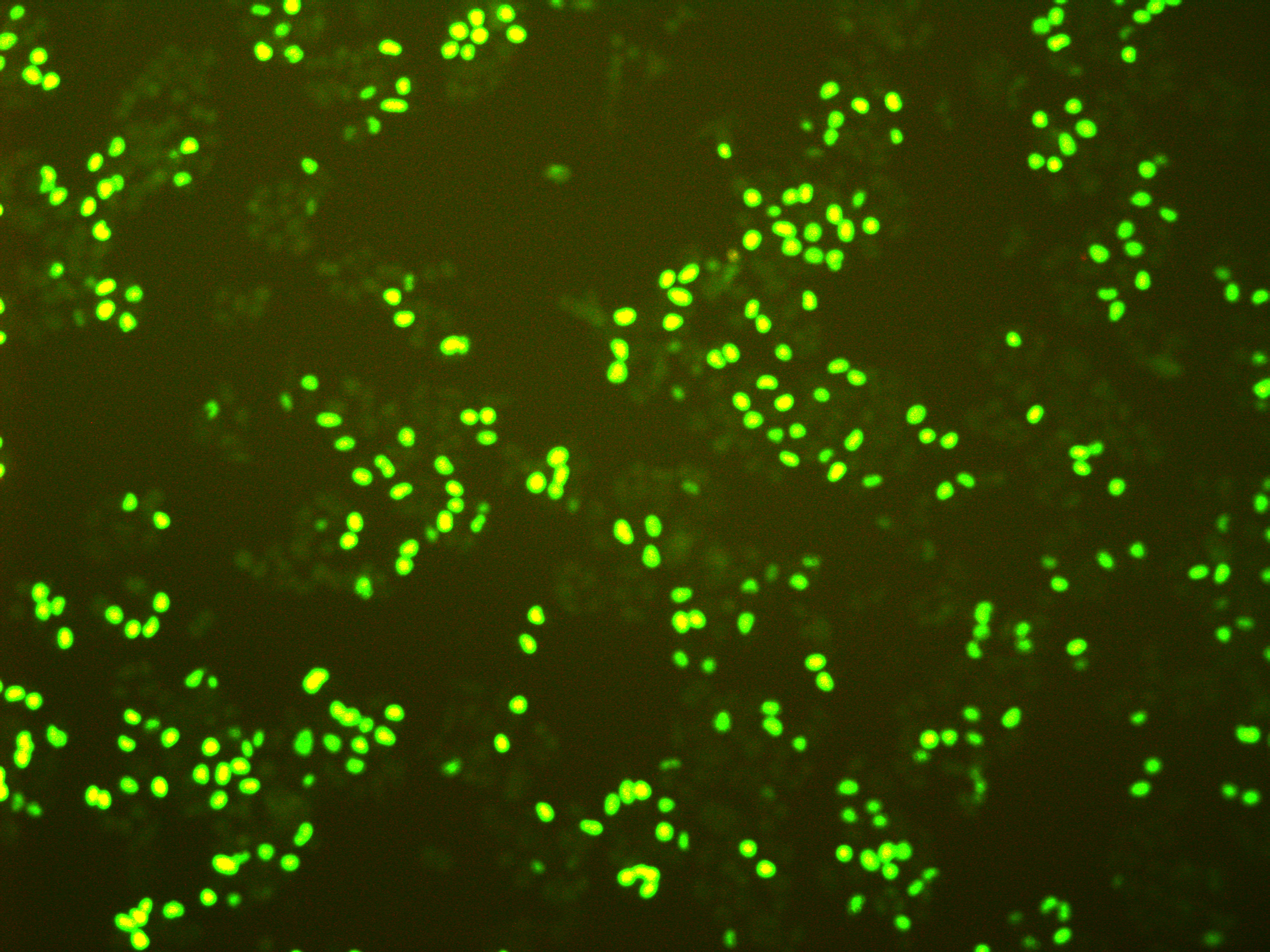

Supplement: S7 File — (ZIP) [file pone.0334639.s007.zip › S 12. File. Original FIgures. Fig.5/5g/SMMC-7721 sh-NC EDU.jpg]

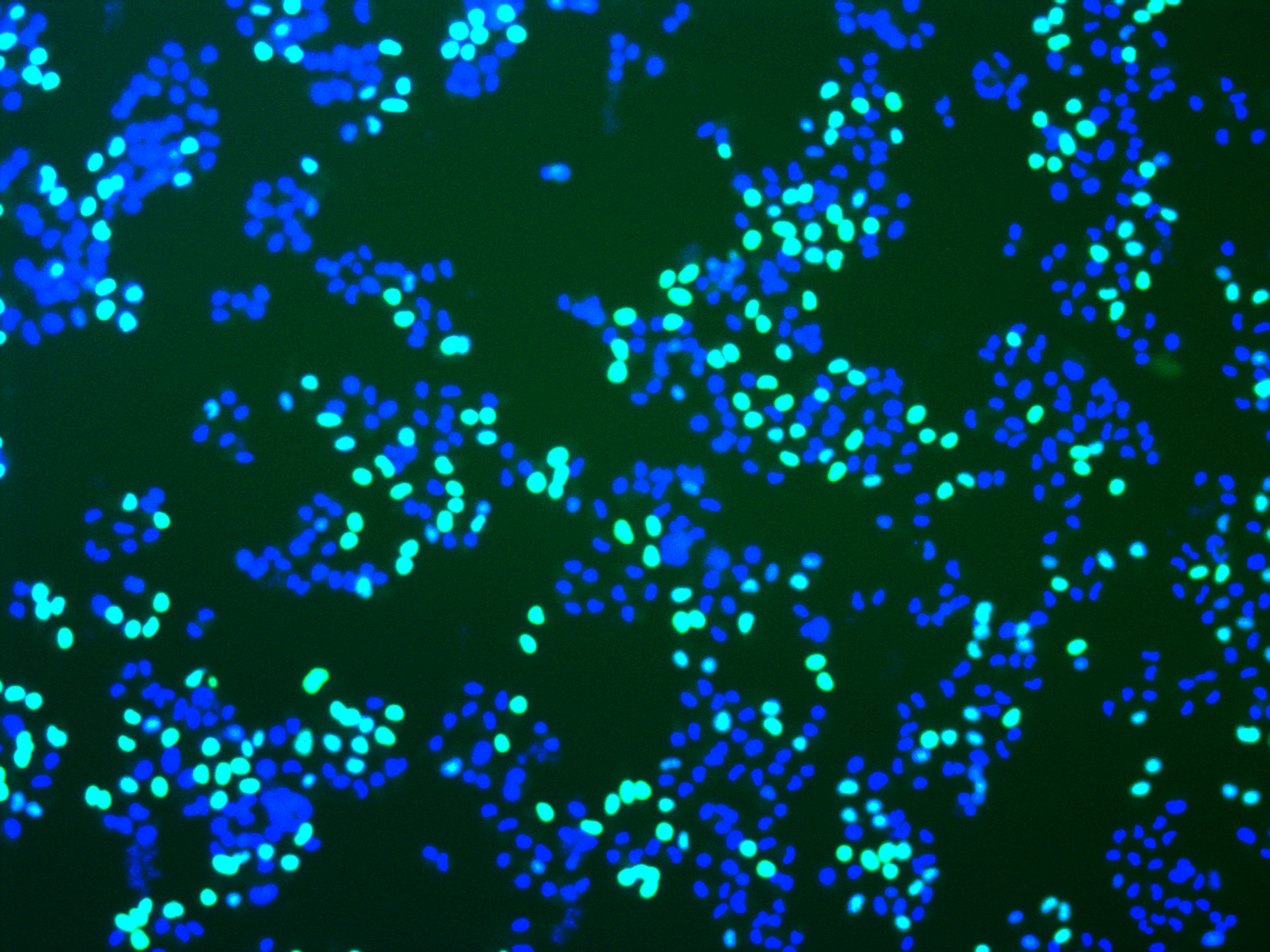

Supplement: S7 File — (ZIP) [file pone.0334639.s007.zip › S 12. File. Original FIgures. Fig.5/5g/SMMC-7721 sh-NC MERGE.jpg]

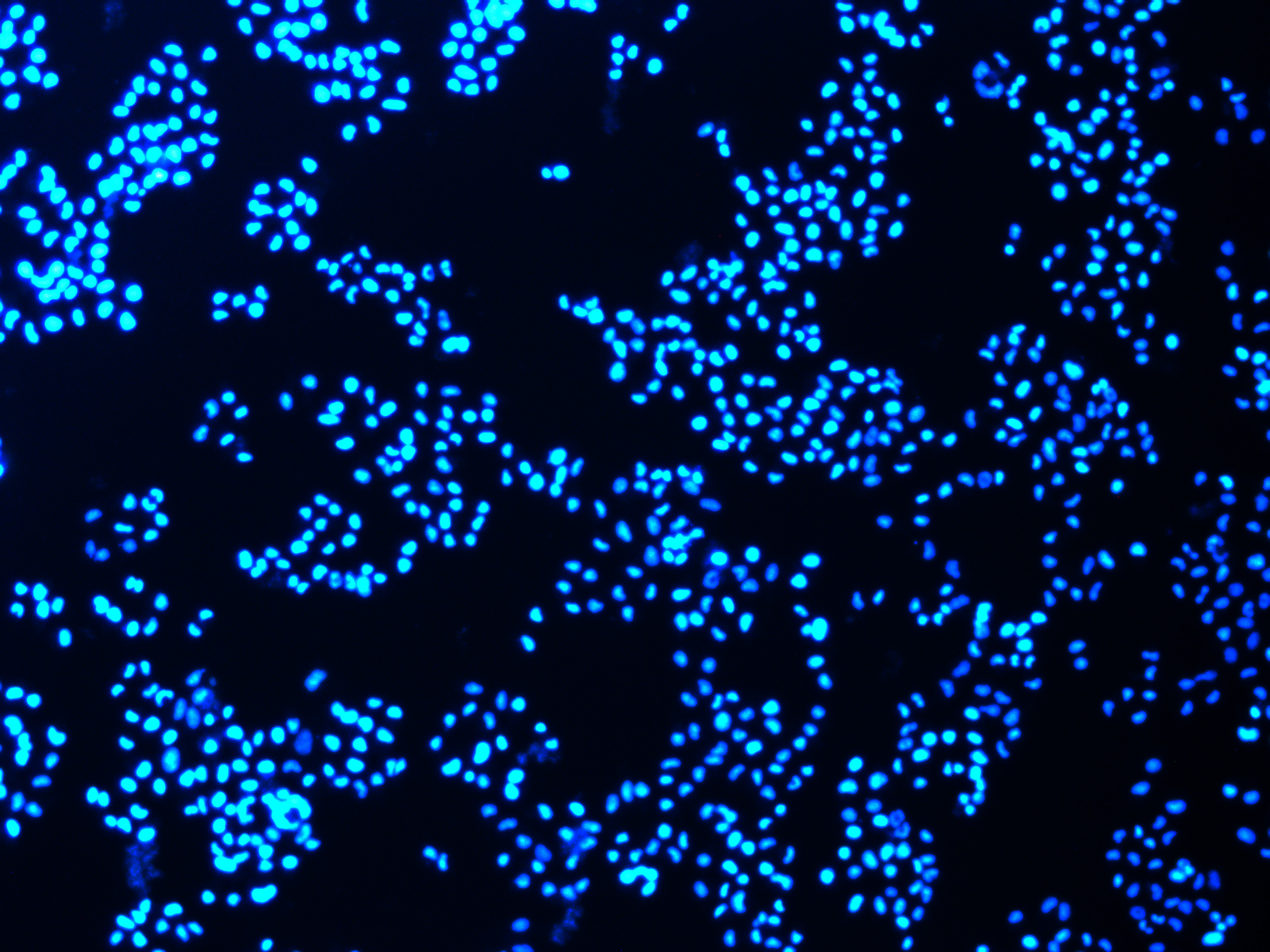

Supplement: S7 File — (ZIP) [file pone.0334639.s007.zip › S 12. File. Original FIgures. Fig.5/5g/SMMC-7721 sh-NC-Hoechst33342 .jpg]

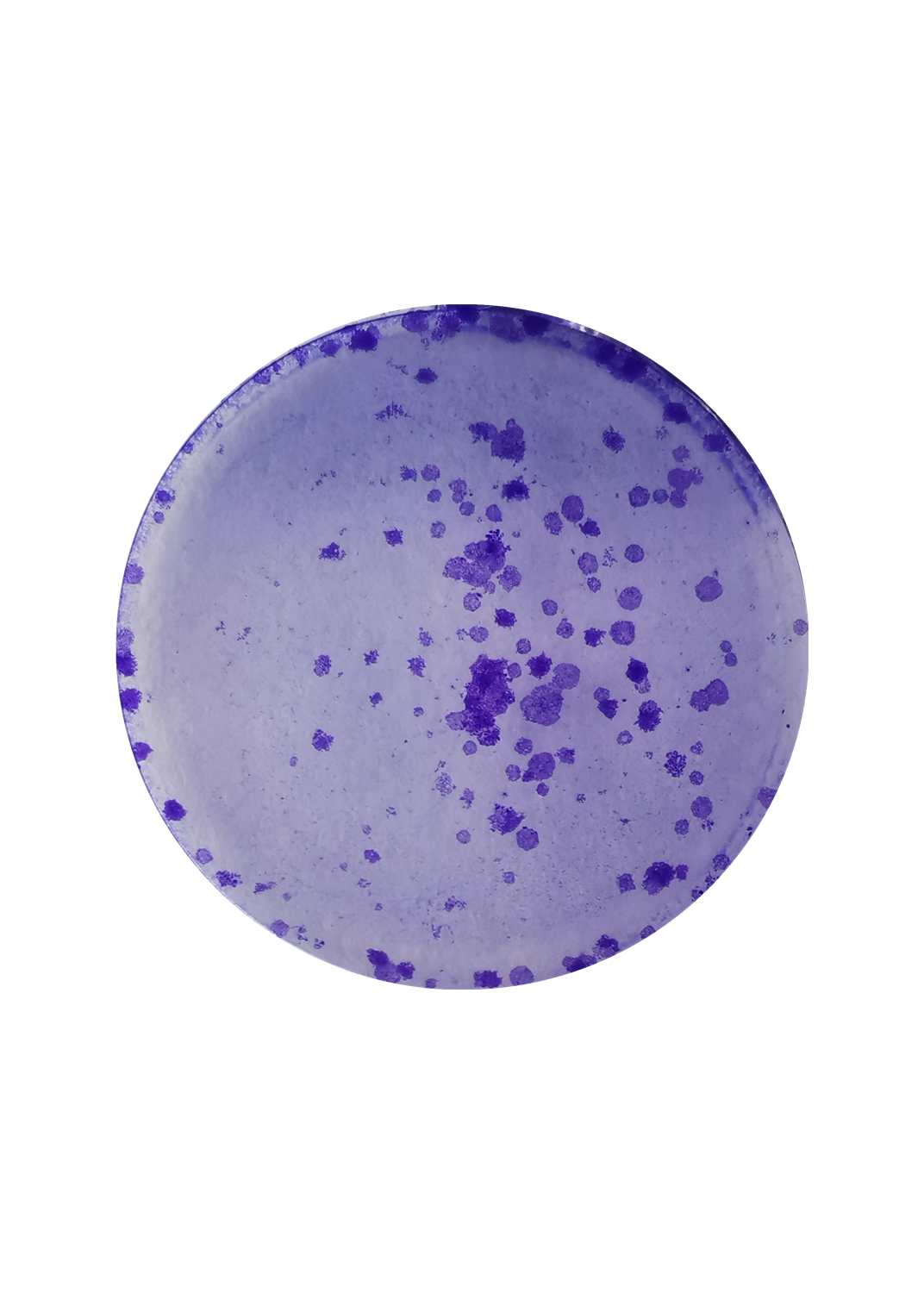

Supplement: S7 File — (ZIP) [file pone.0334639.s007.zip › S 12. File. Original FIgures. Fig.5/5h/BEL-7402-shCXCL3.png]

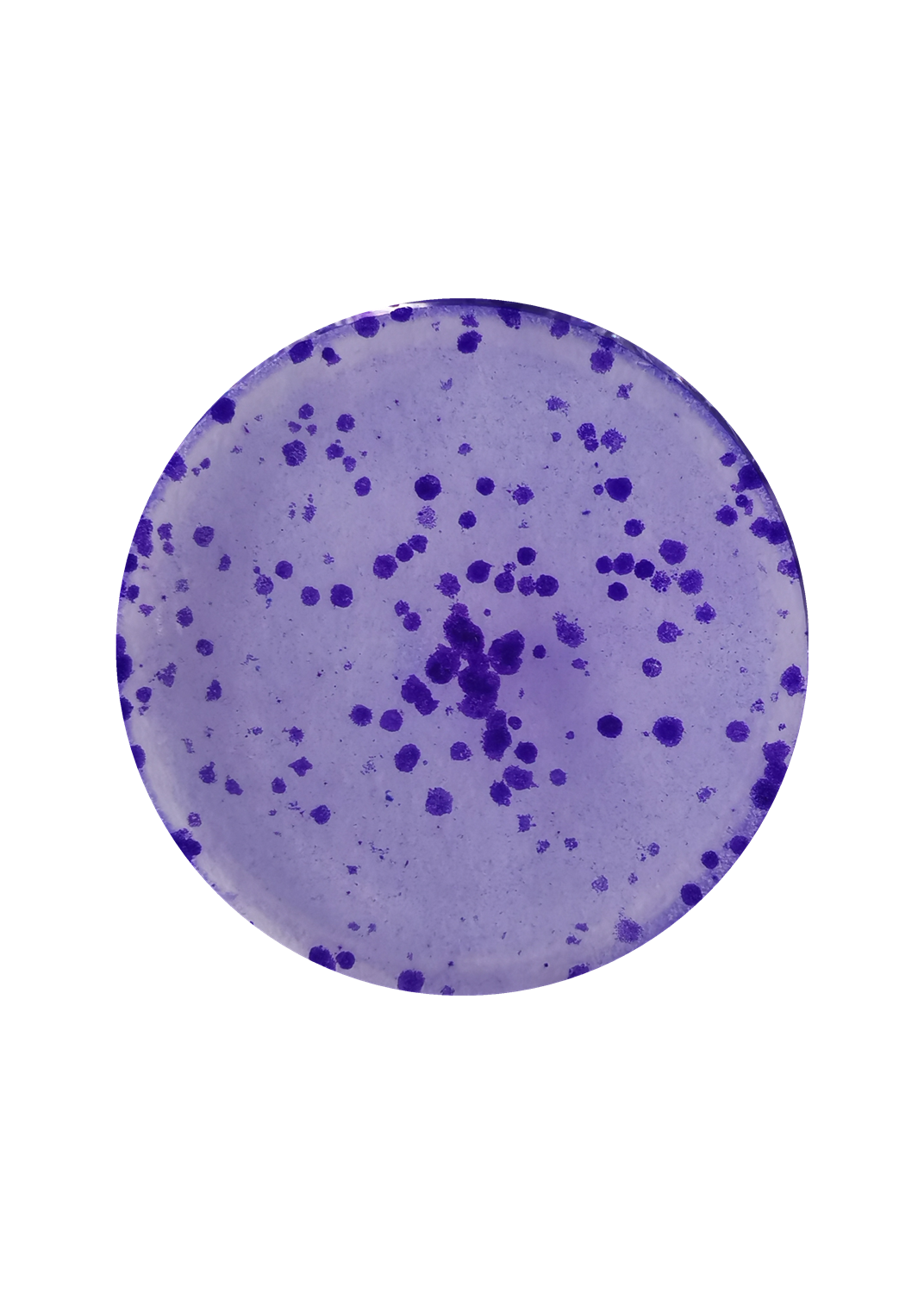

Supplement: S7 File — (ZIP) [file pone.0334639.s007.zip › S 12. File. Original FIgures. Fig.5/5h/BEL-7402-shNC.png]

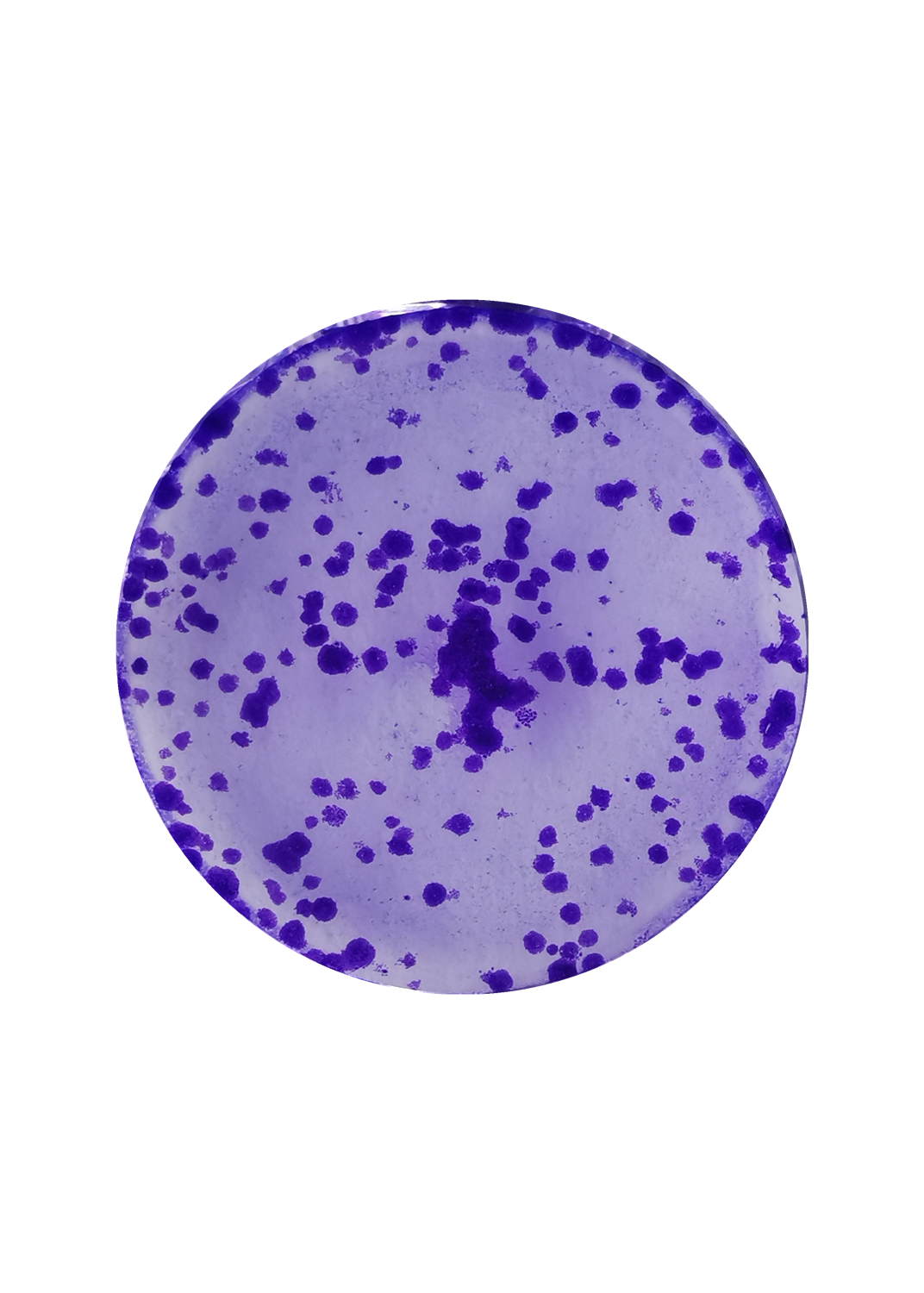

Supplement: S7 File — (ZIP) [file pone.0334639.s007.zip › S 12. File. Original FIgures. Fig.5/5h/hepg2-sh-NC.png]

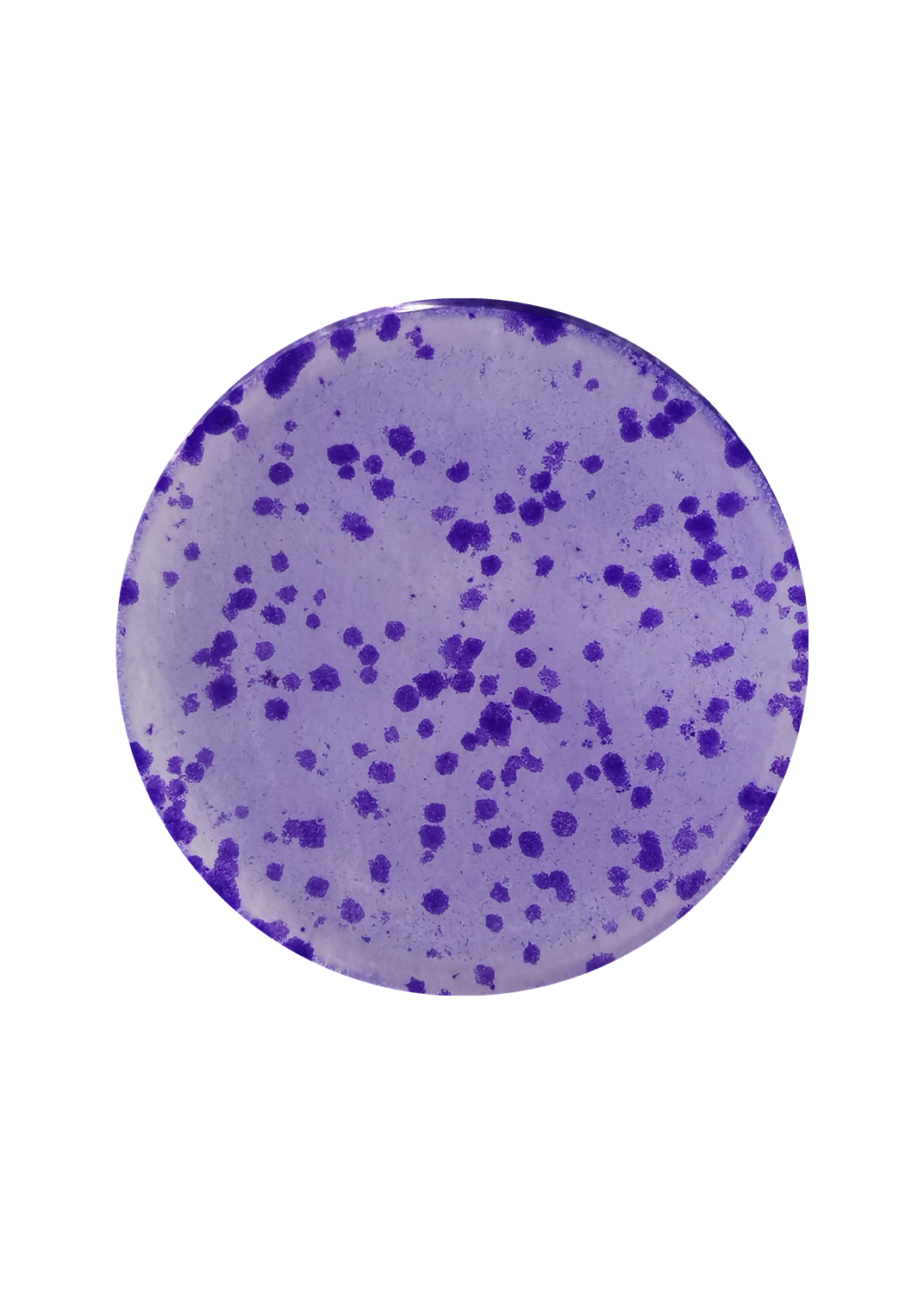

Supplement: S7 File — (ZIP) [file pone.0334639.s007.zip › S 12. File. Original FIgures. Fig.5/5h/hepg2-shCXCL3.png]

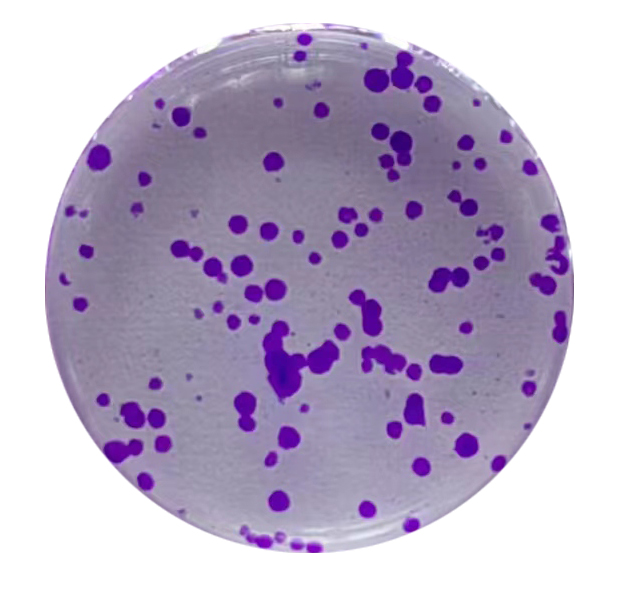

Supplement: S7 File — (ZIP) [file pone.0334639.s007.zip › S 12. File. Original FIgures. Fig.5/5h/SMMC-7721-sh-CXCL3.jpg]

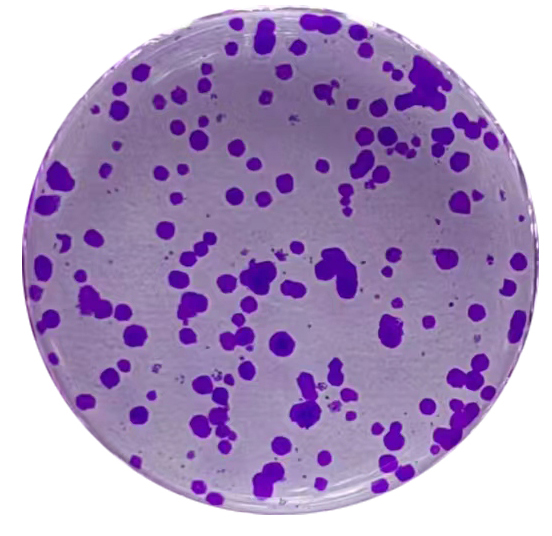

Supplement: S7 File — (ZIP) [file pone.0334639.s007.zip › S 12. File. Original FIgures. Fig.5/5h/SMMC-7721-sh-NC.jpg]

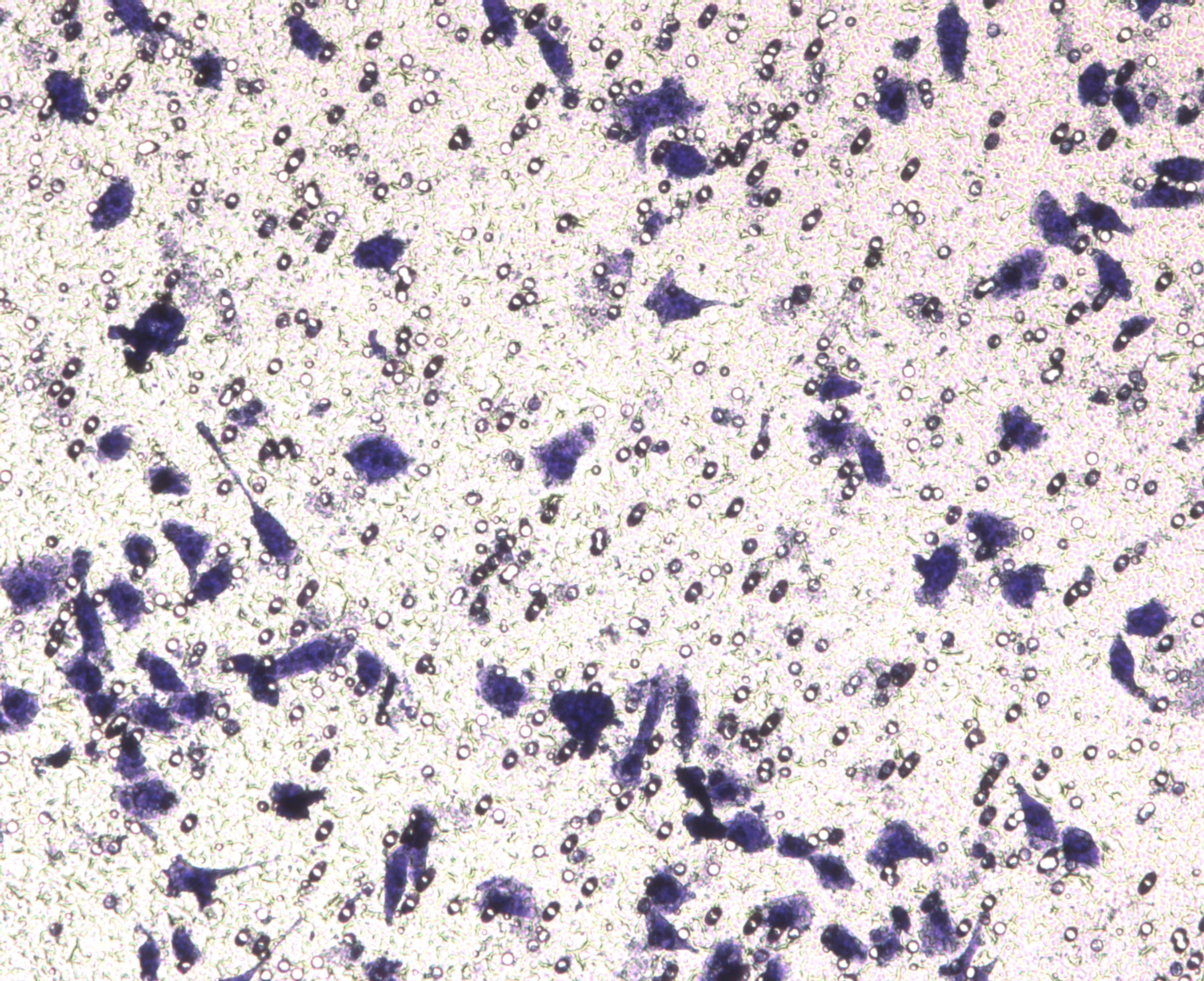

Supplement: S7 File — (ZIP) [file pone.0334639.s007.zip › S 12. File. Original FIgures. Fig.5/5i/bel-7402 sh-CXCL3 .jpg]

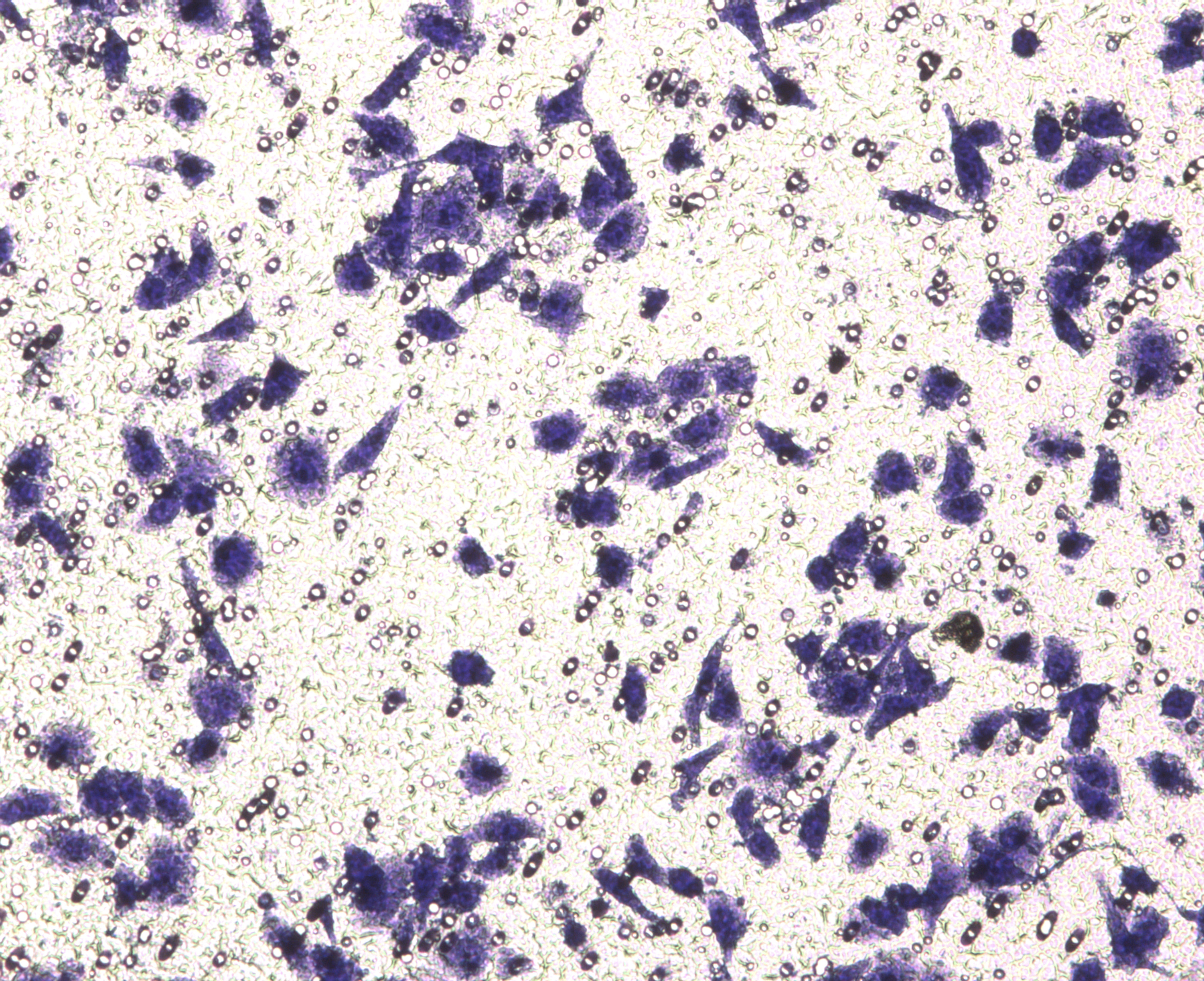

Supplement: S7 File — (ZIP) [file pone.0334639.s007.zip › S 12. File. Original FIgures. Fig.5/5i/Bel-7402 sh-NC.jpg]

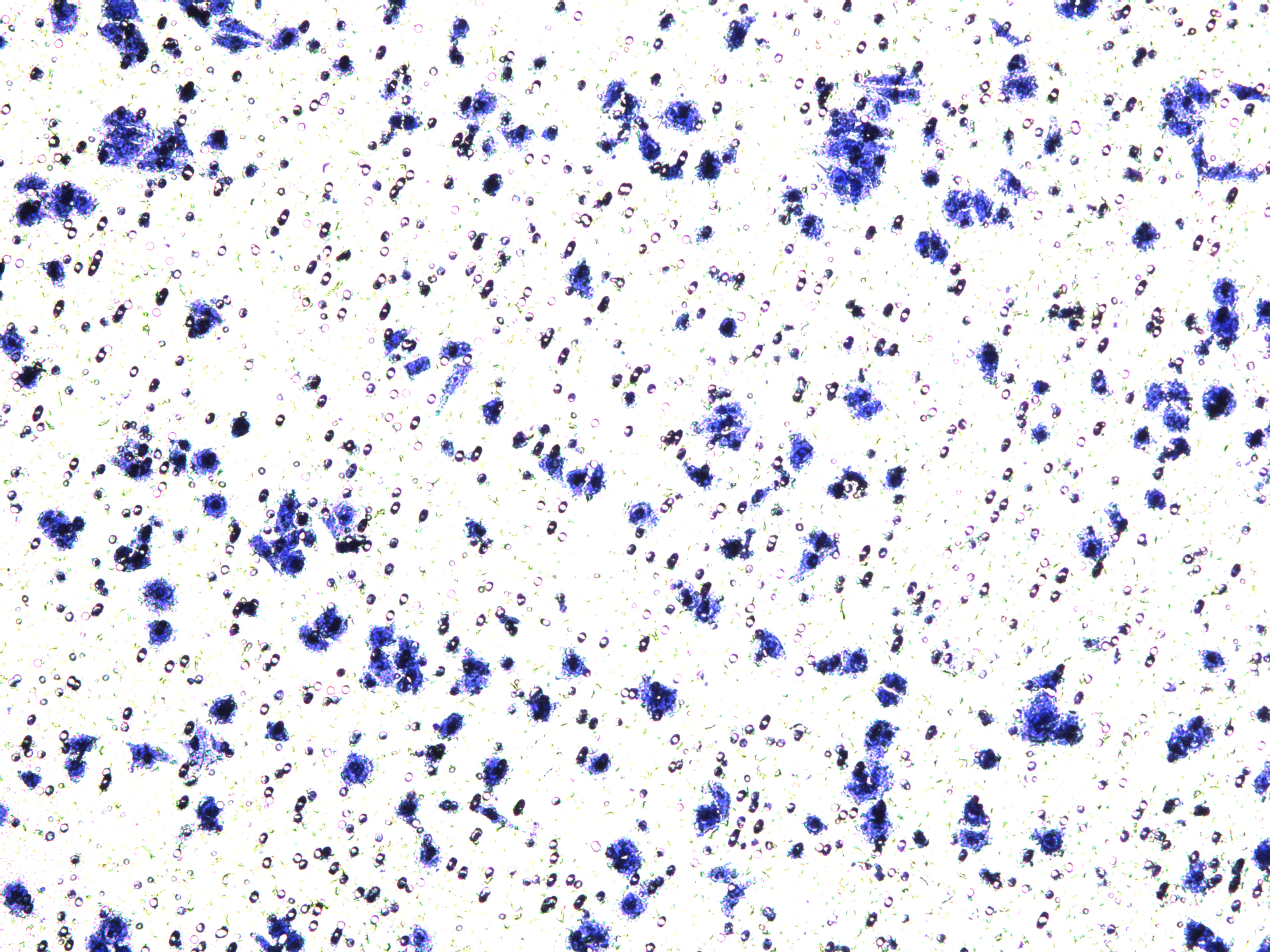

Supplement: S7 File — (ZIP) [file pone.0334639.s007.zip › S 12. File. Original FIgures. Fig.5/5i/Hepg2 sh-CXCL3.jpg]

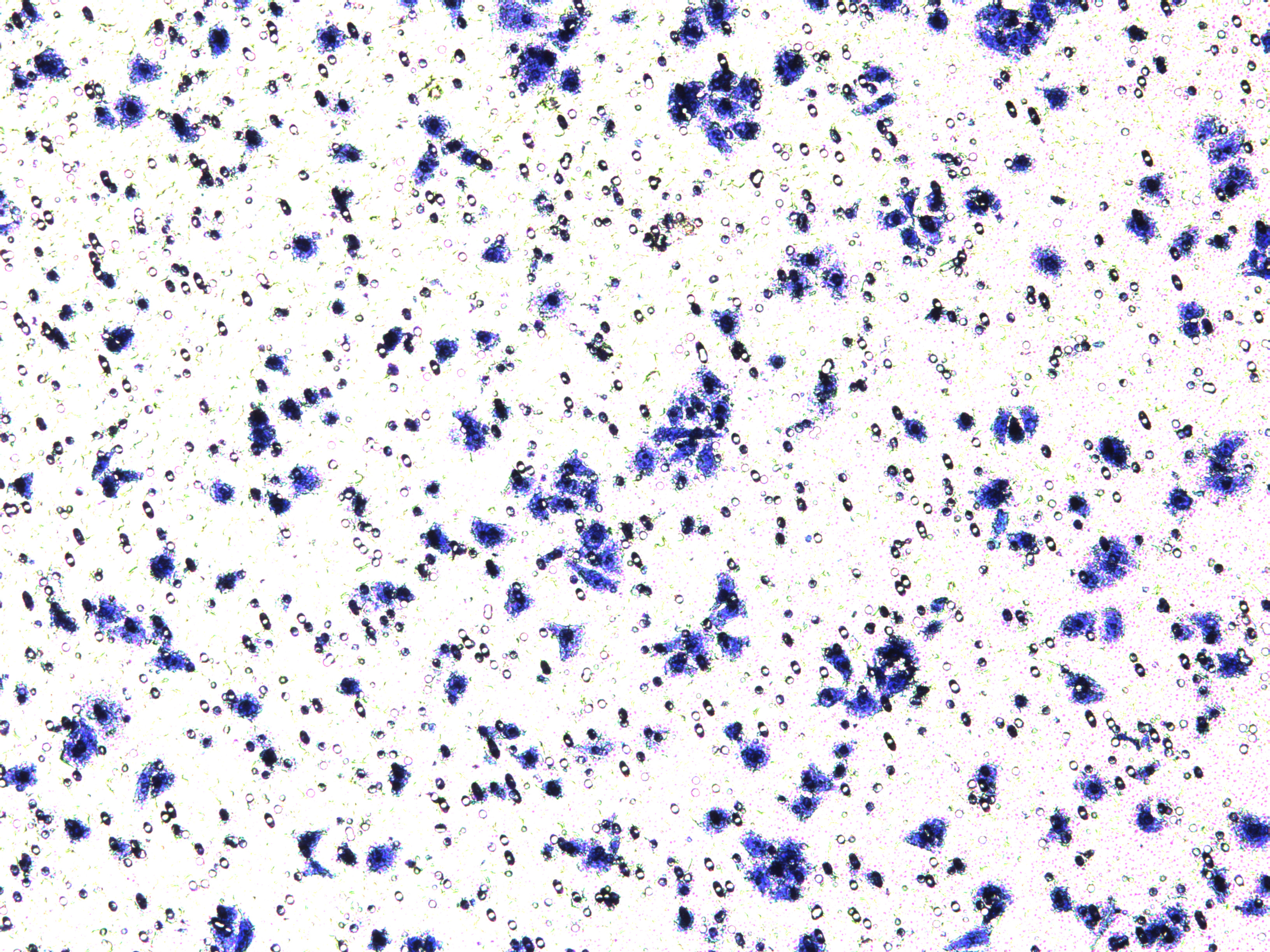

Supplement: S7 File — (ZIP) [file pone.0334639.s007.zip › S 12. File. Original FIgures. Fig.5/5i/Hepg2 sh-NC.jpg]

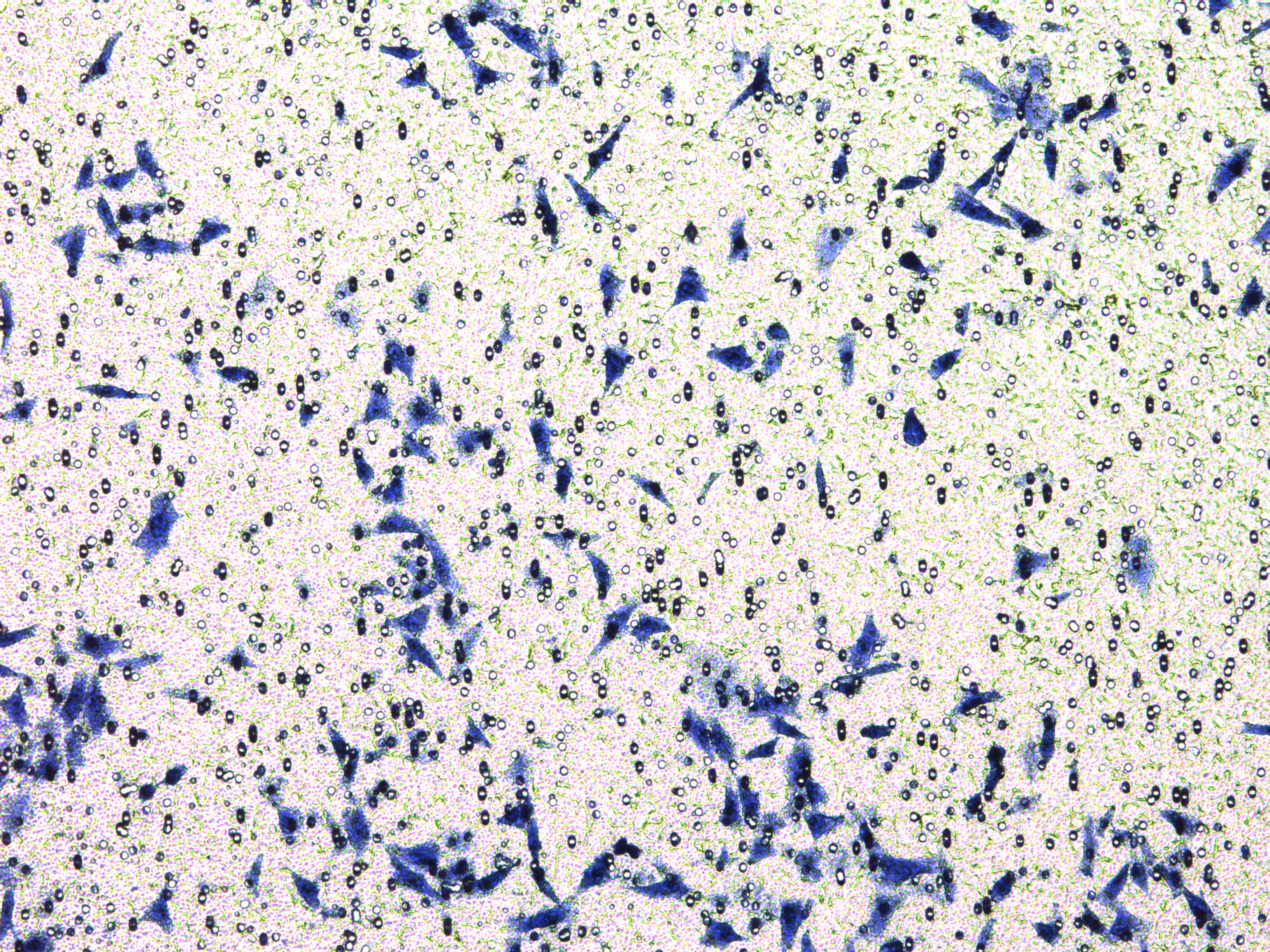

Supplement: S7 File — (ZIP) [file pone.0334639.s007.zip › S 12. File. Original FIgures. Fig.5/5i/SMMC-7721 sh-CXCL3.jpg]

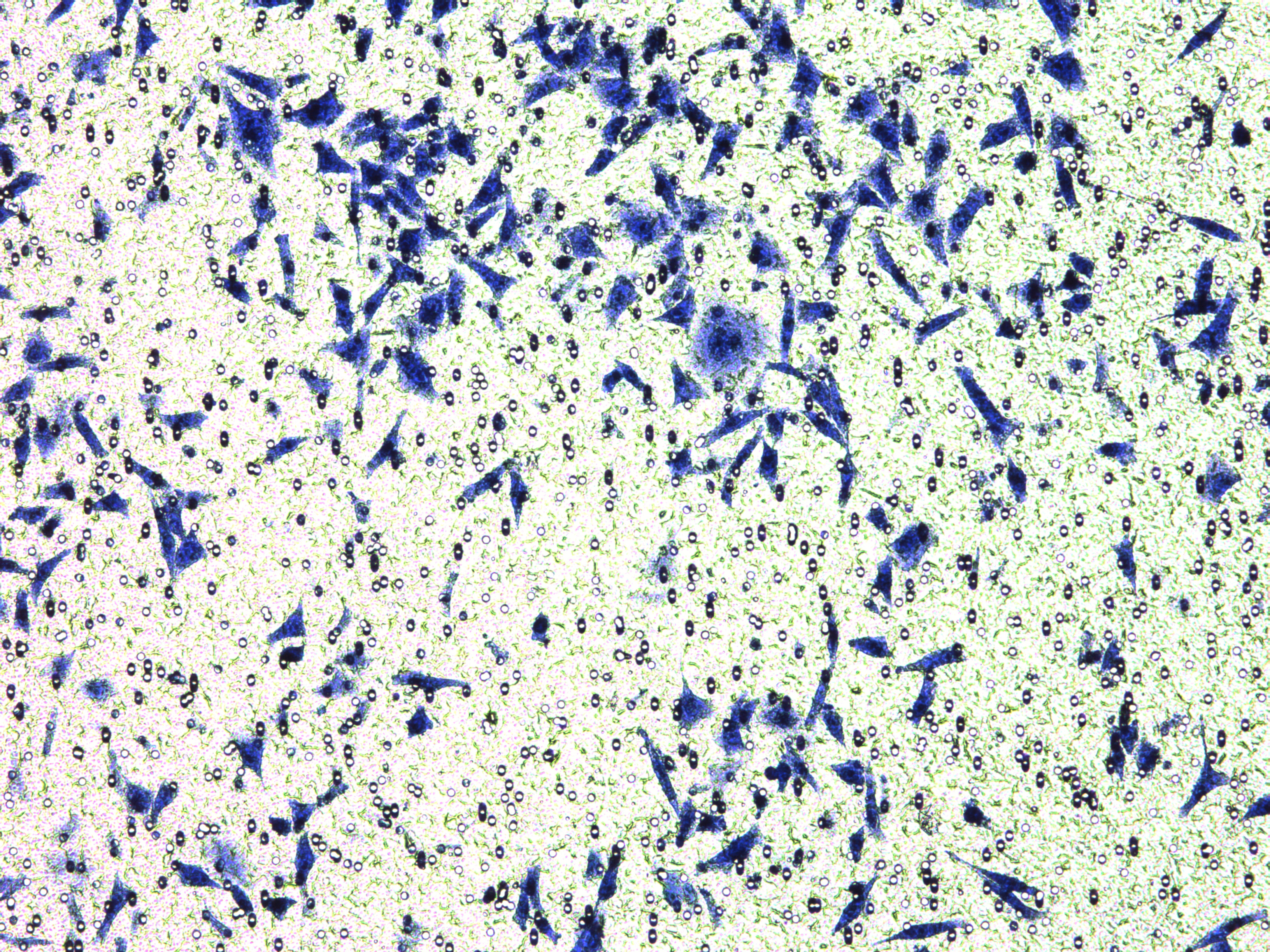

Supplement: S7 File — (ZIP) [file pone.0334639.s007.zip › S 12. File. Original FIgures. Fig.5/5i/SMMC-7721 sh-NC.jpg]

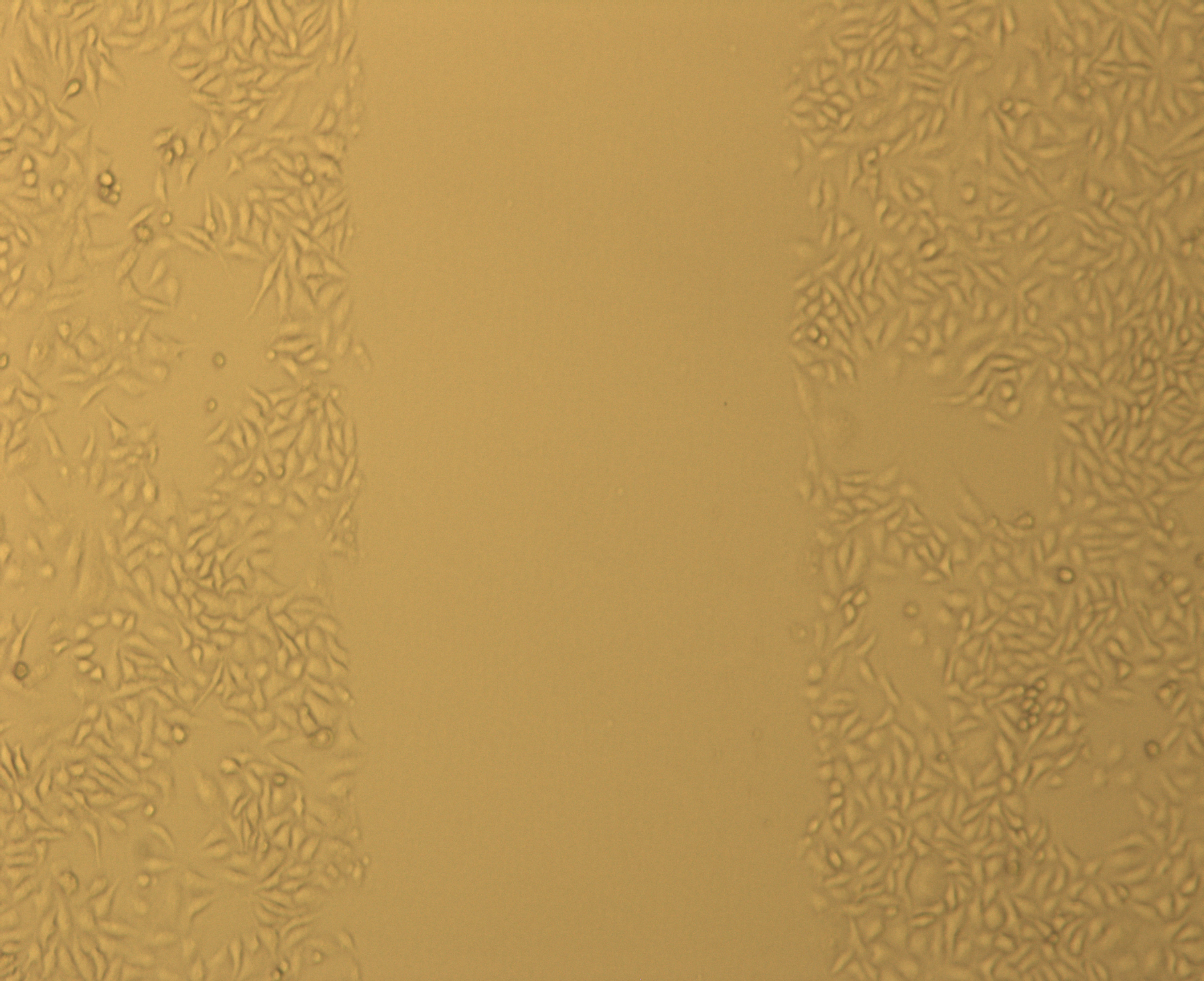

Supplement: S7 File — (ZIP) [file pone.0334639.s007.zip › S 12. File. Original FIgures. Fig.5/5j/BEL-7402 sh-CXCL3 0H.jpg]

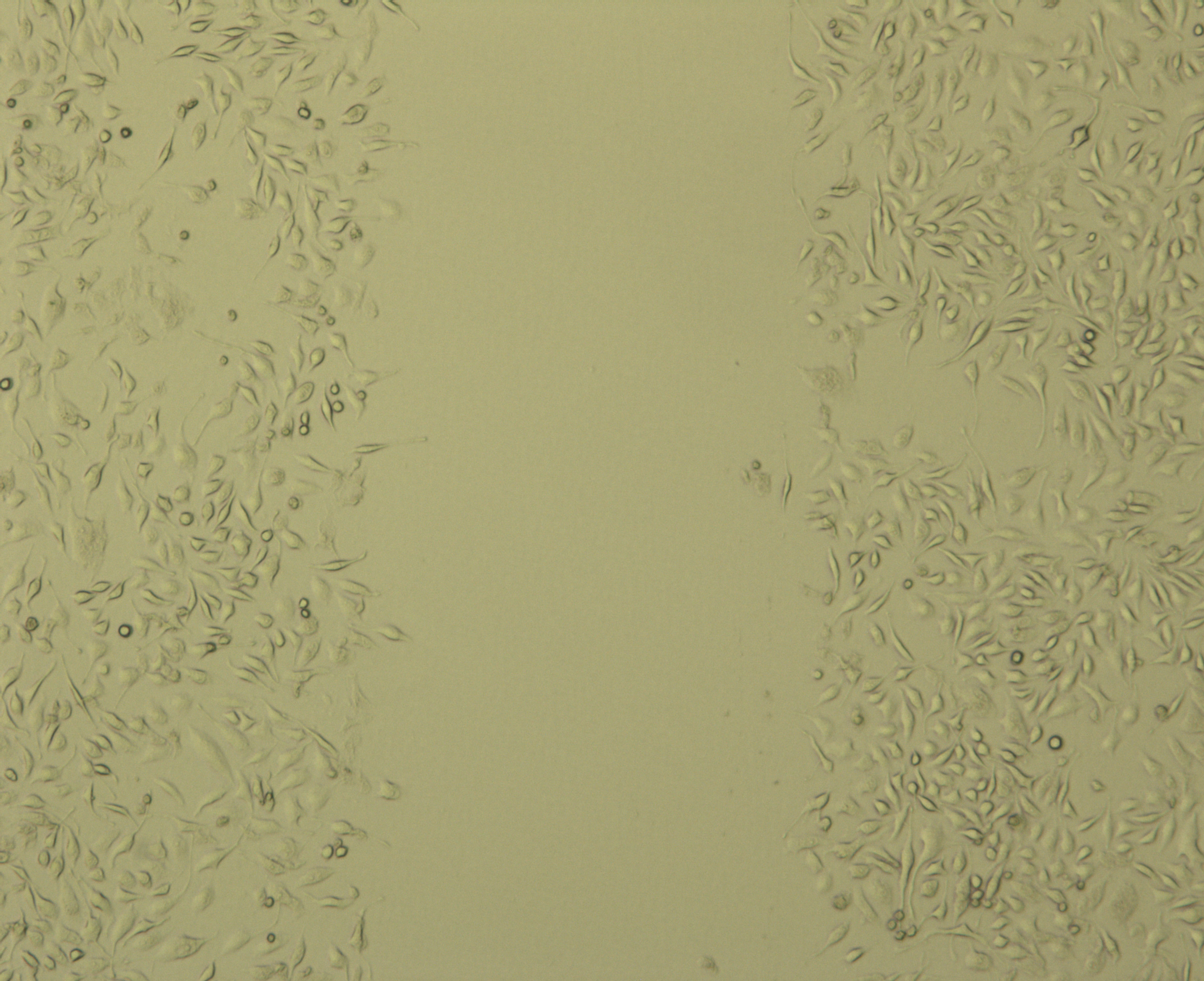

Supplement: S7 File — (ZIP) [file pone.0334639.s007.zip › S 12. File. Original FIgures. Fig.5/5j/BEL-7402 sh-CXCL3 24H.jpg]

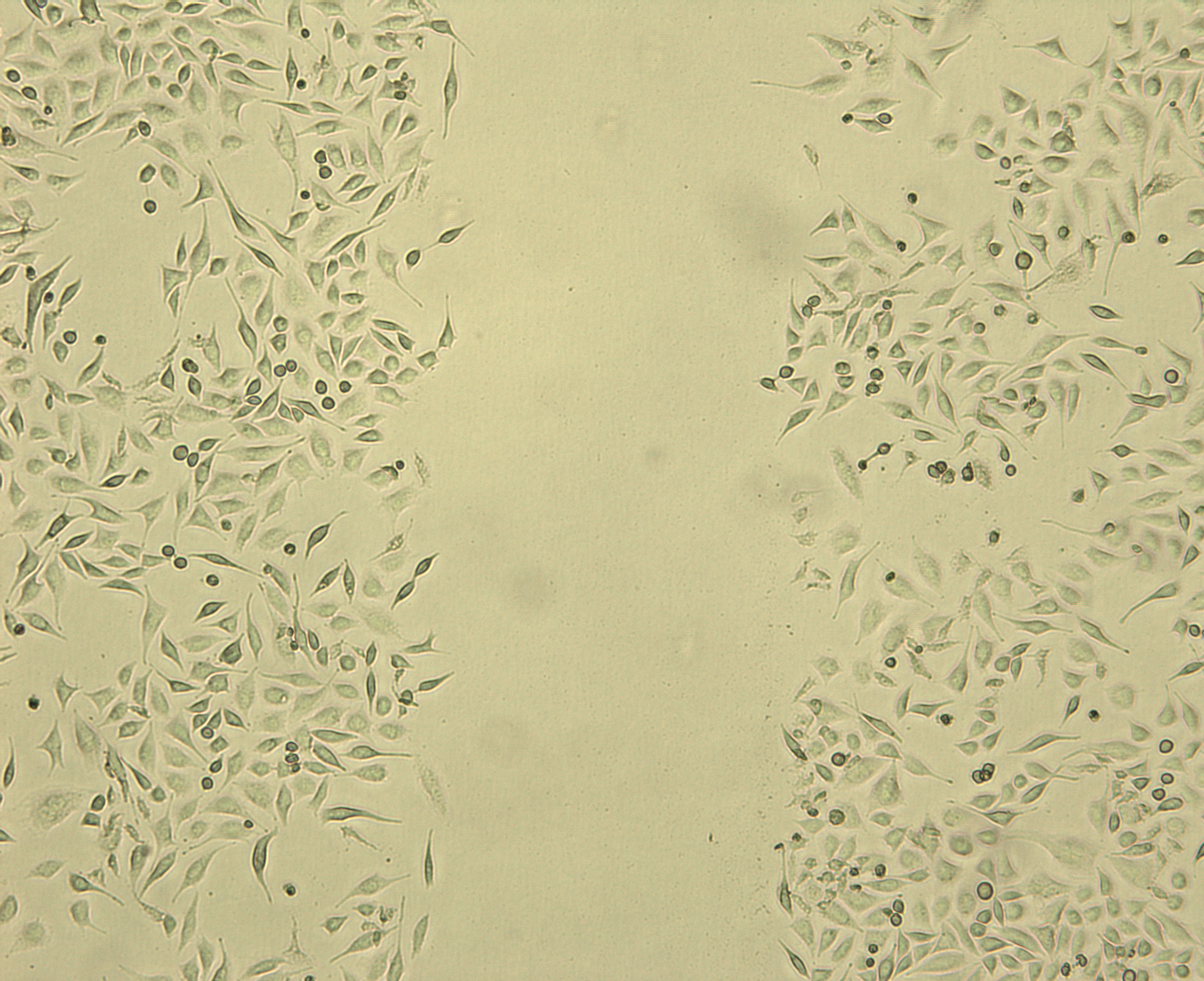

Supplement: S7 File — (ZIP) [file pone.0334639.s007.zip › S 12. File. Original FIgures. Fig.5/5j/BEL-7402 sh-CXCL3 48H.jpg]

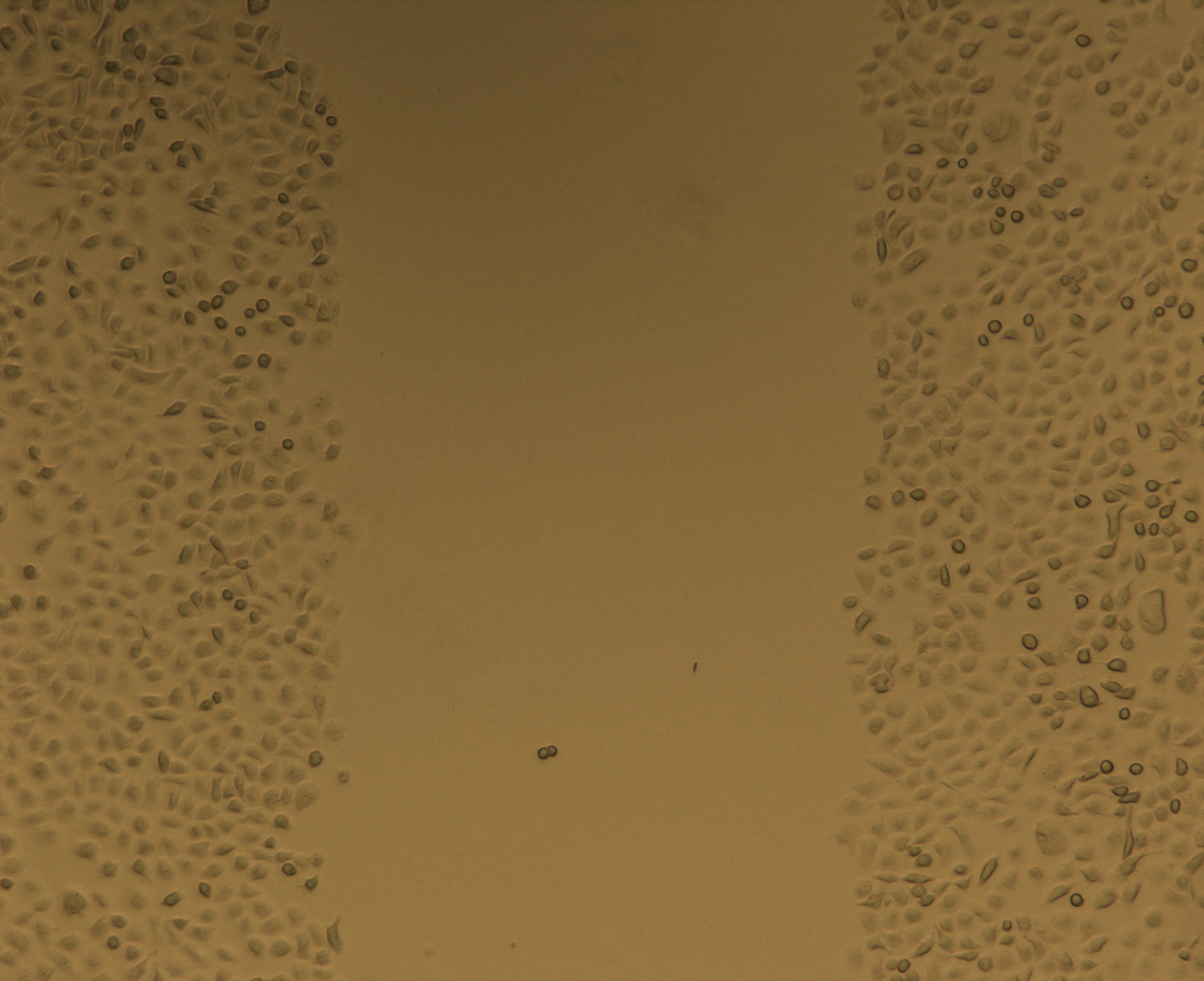

Supplement: S7 File — (ZIP) [file pone.0334639.s007.zip › S 12. File. Original FIgures. Fig.5/5j/BEL-7402 sh-NC 0H.jpg]

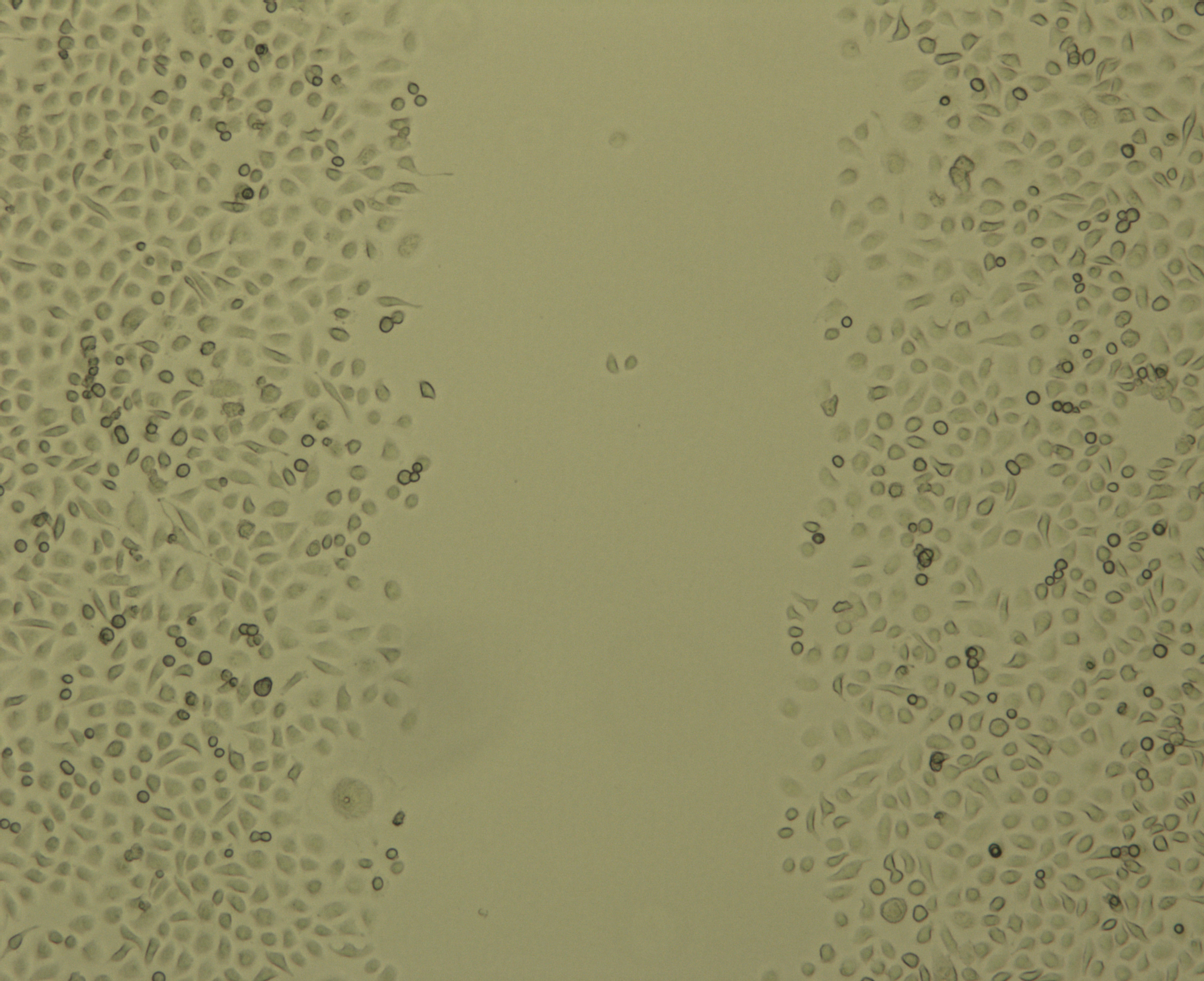

Supplement: S7 File — (ZIP) [file pone.0334639.s007.zip › S 12. File. Original FIgures. Fig.5/5j/BEL-7402 sh-NC 24H.jpg]

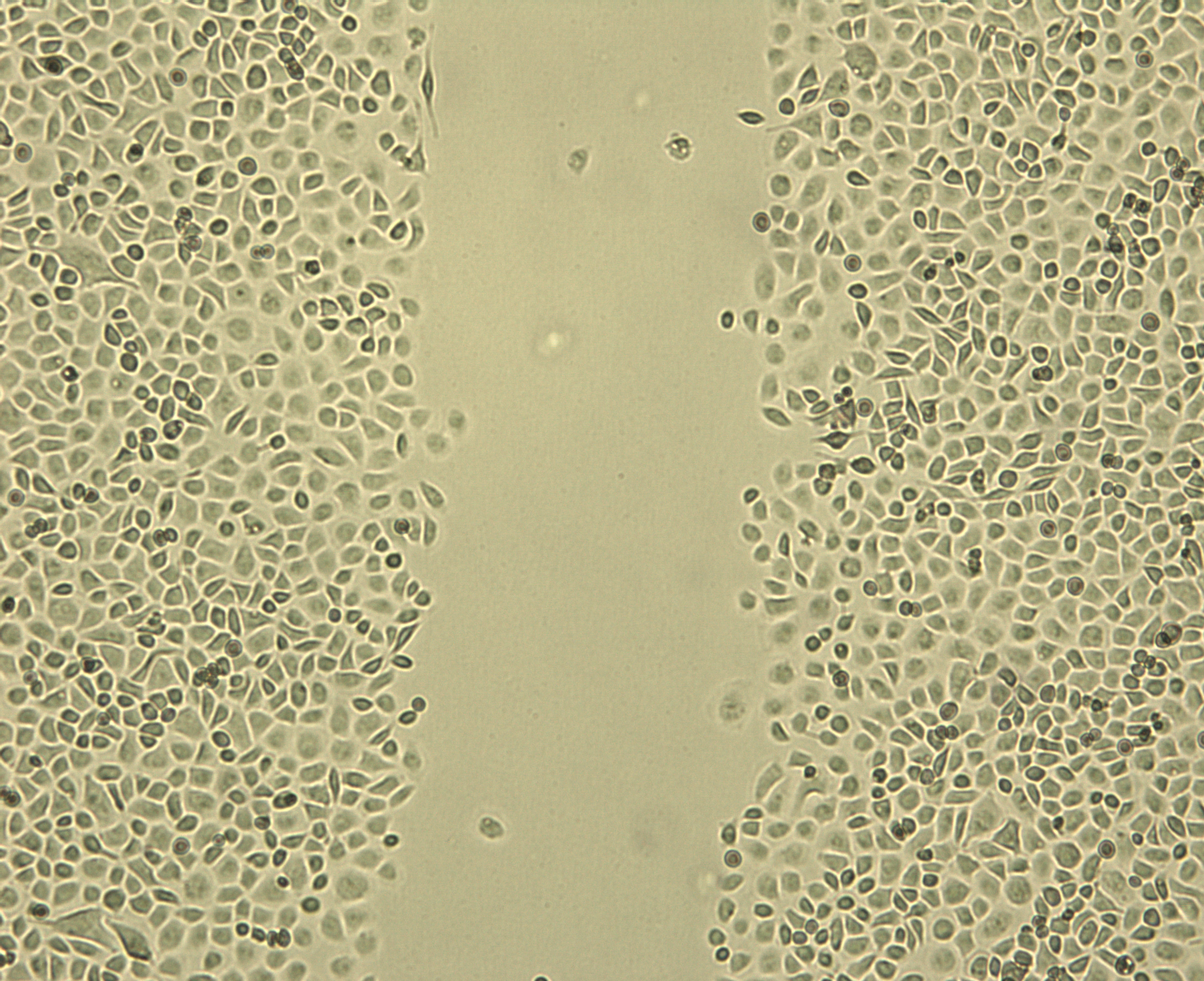

Supplement: S7 File — (ZIP) [file pone.0334639.s007.zip › S 12. File. Original FIgures. Fig.5/5j/BEL-7402 sh-NC 48H.jpg]

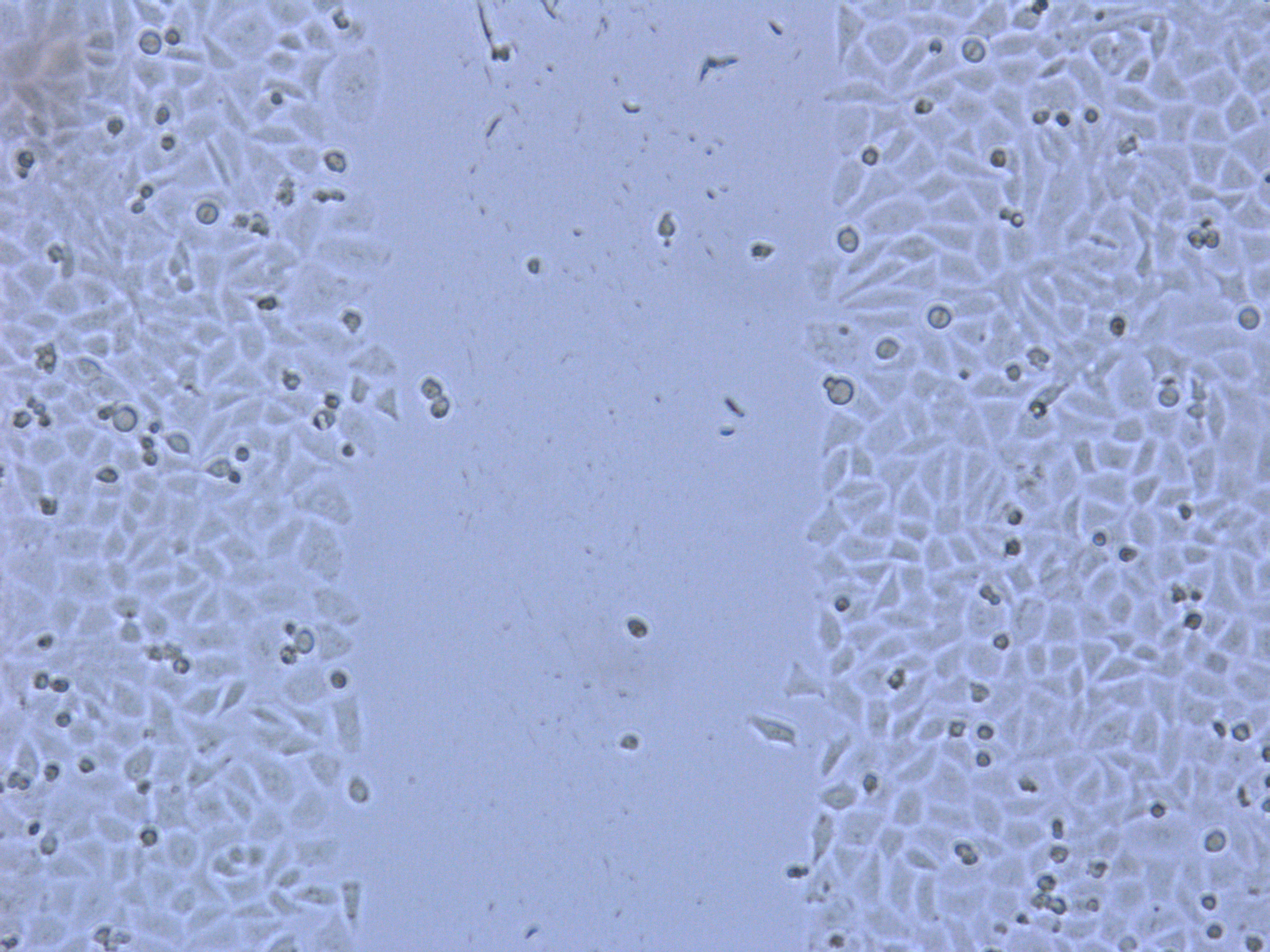

Supplement: S7 File — (ZIP) [file pone.0334639.s007.zip › S 12. File. Original FIgures. Fig.5/5k/HepG2 sh-CXCL3 0H.jpg]

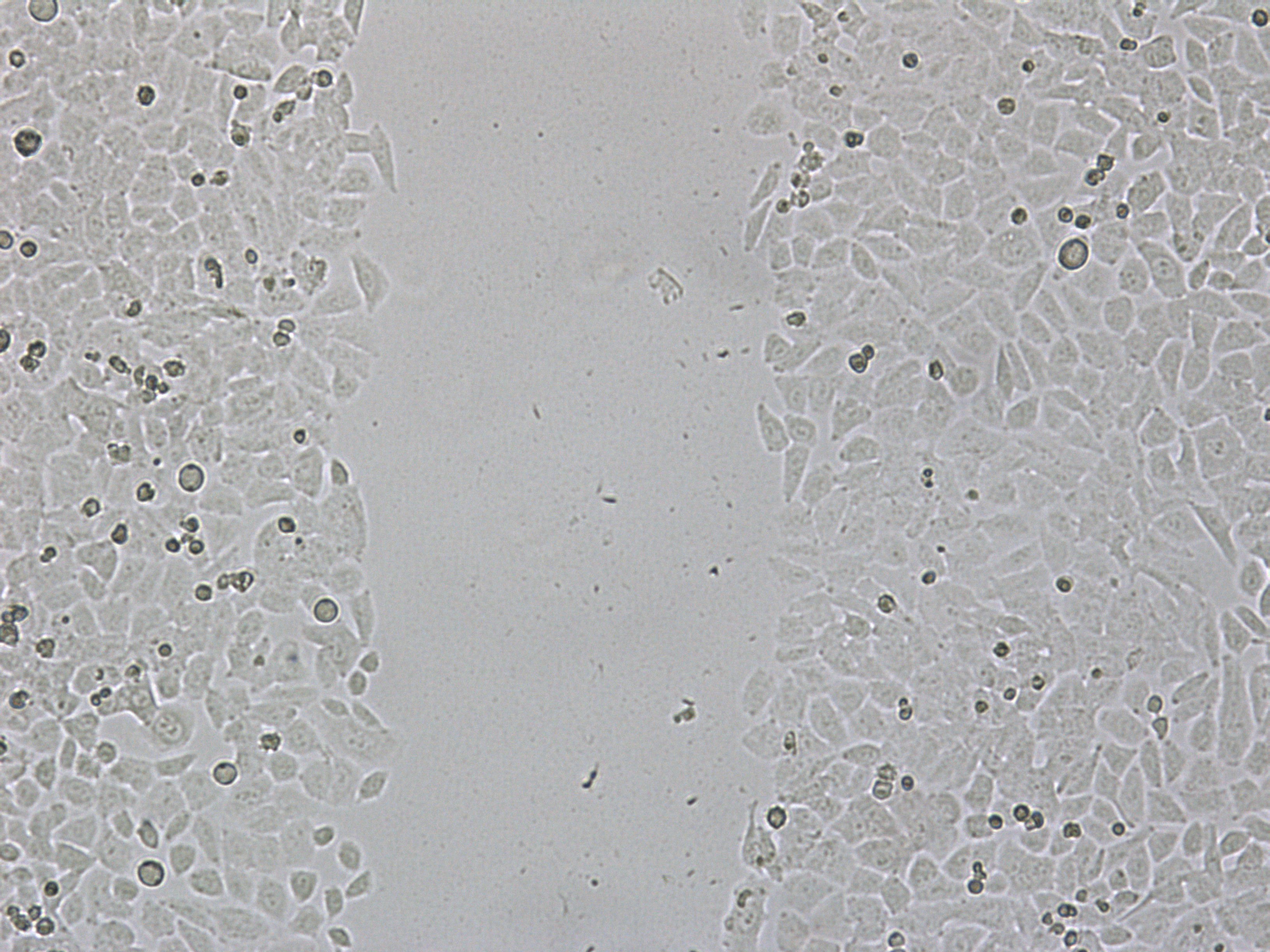

Supplement: S7 File — (ZIP) [file pone.0334639.s007.zip › S 12. File. Original FIgures. Fig.5/5k/HepG2 sh-CXCL3 24H.jpg]

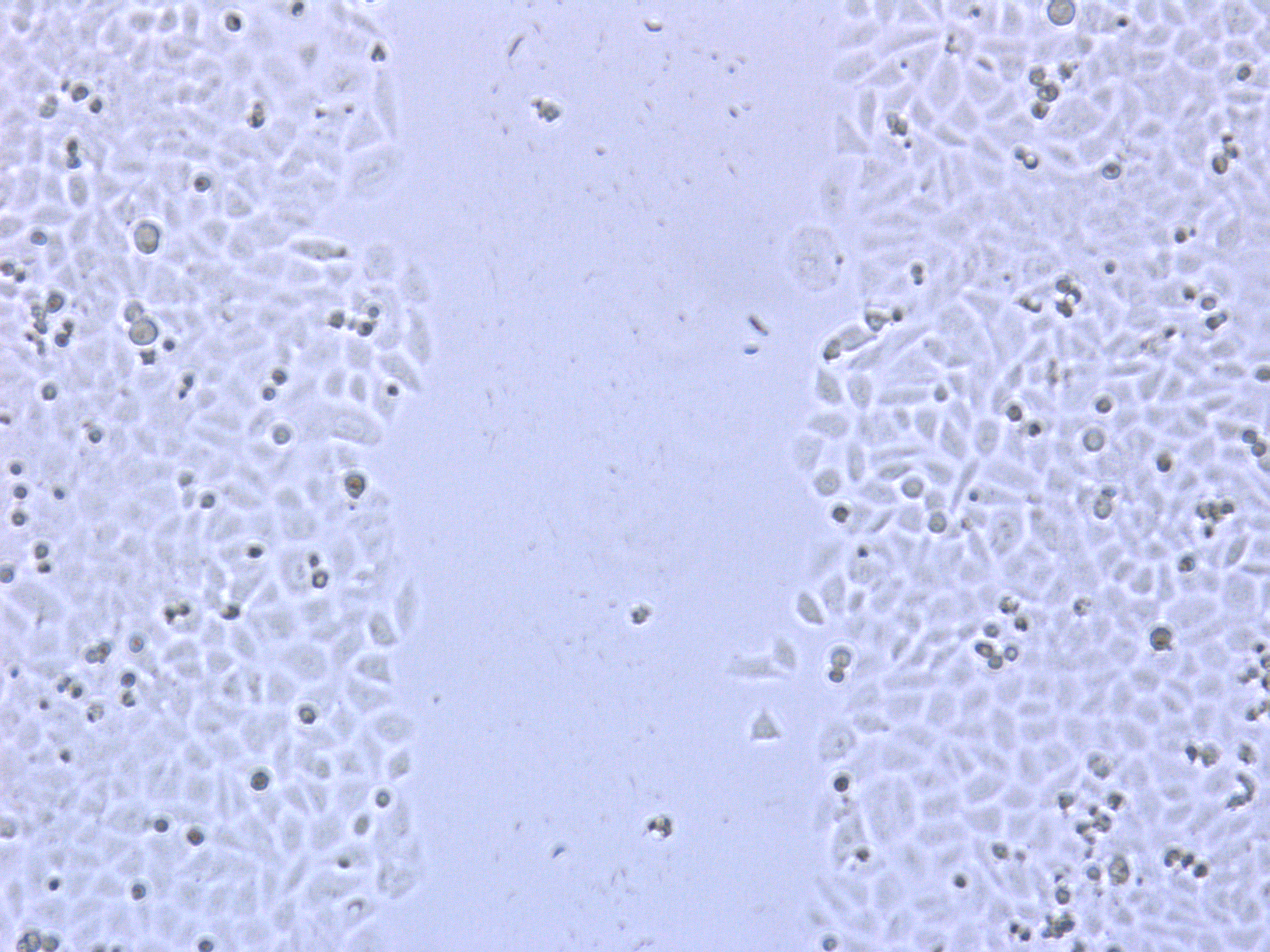

Supplement: S7 File — (ZIP) [file pone.0334639.s007.zip › S 12. File. Original FIgures. Fig.5/5k/HepG2 sh-CXCL3 48H.jpg]

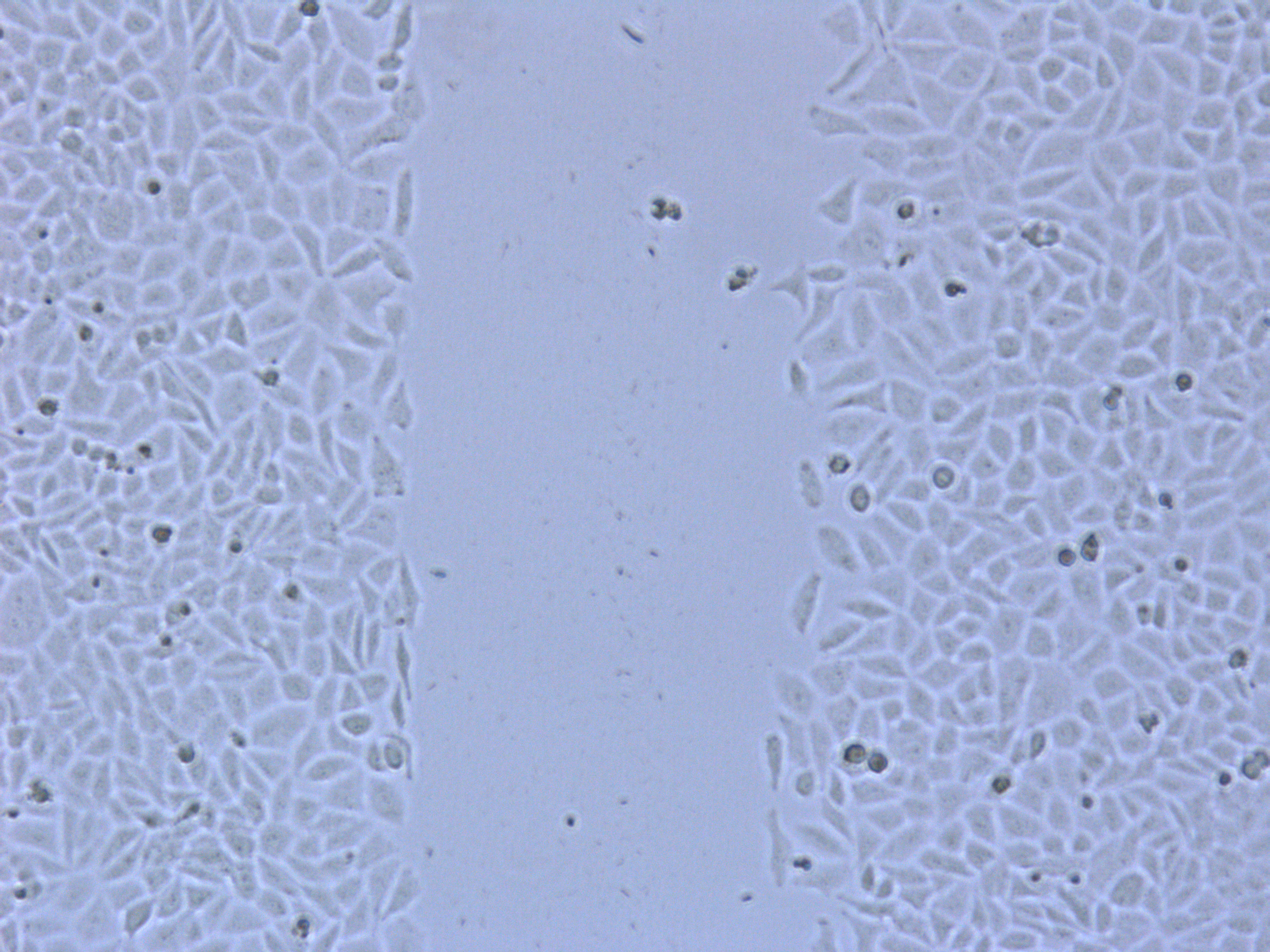

Supplement: S7 File — (ZIP) [file pone.0334639.s007.zip › S 12. File. Original FIgures. Fig.5/5k/HepG2 sh-NC 0H.jpg]

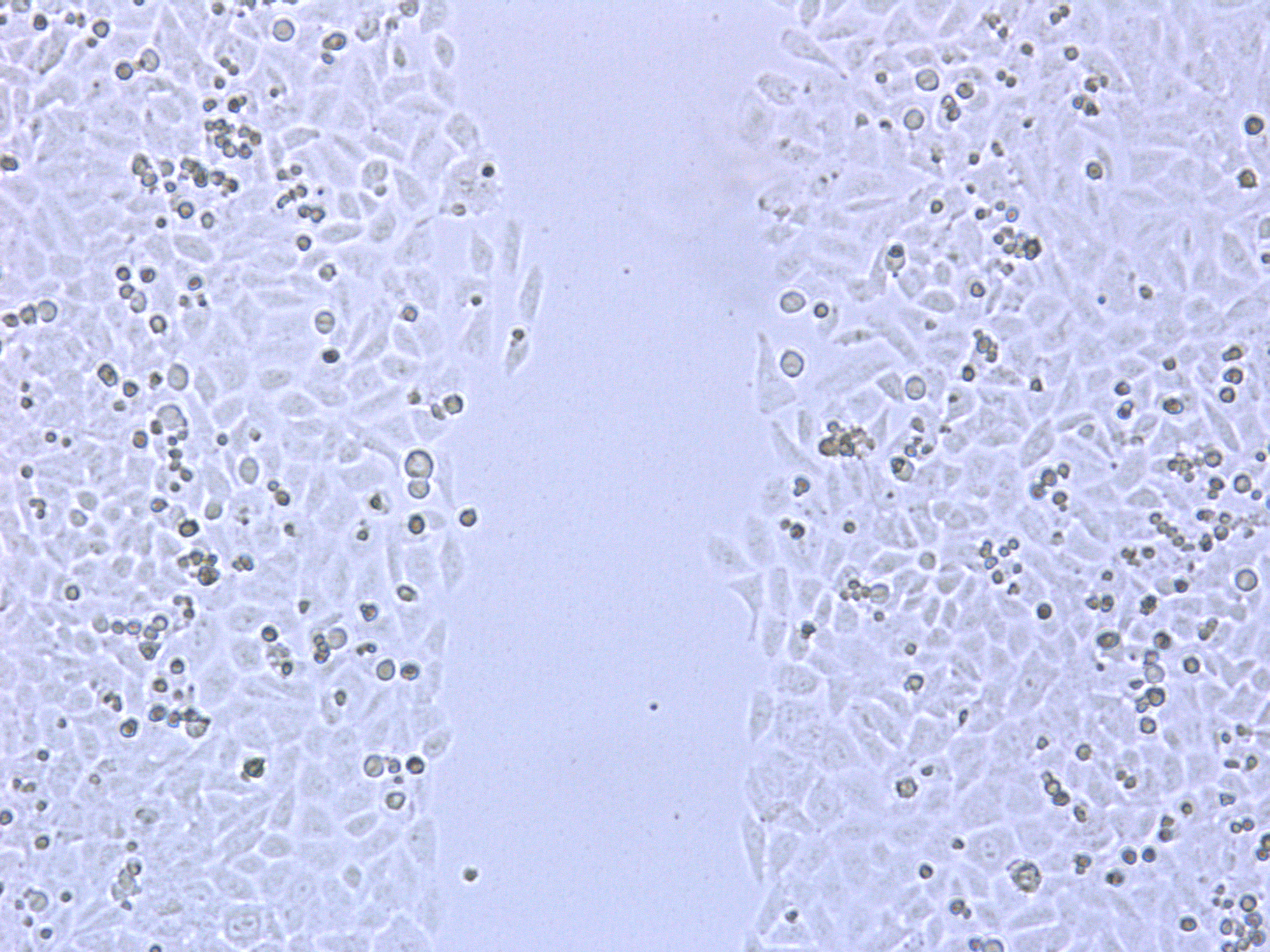

Supplement: S7 File — (ZIP) [file pone.0334639.s007.zip › S 12. File. Original FIgures. Fig.5/5k/HepG2 sh-NC 24H.jpg]

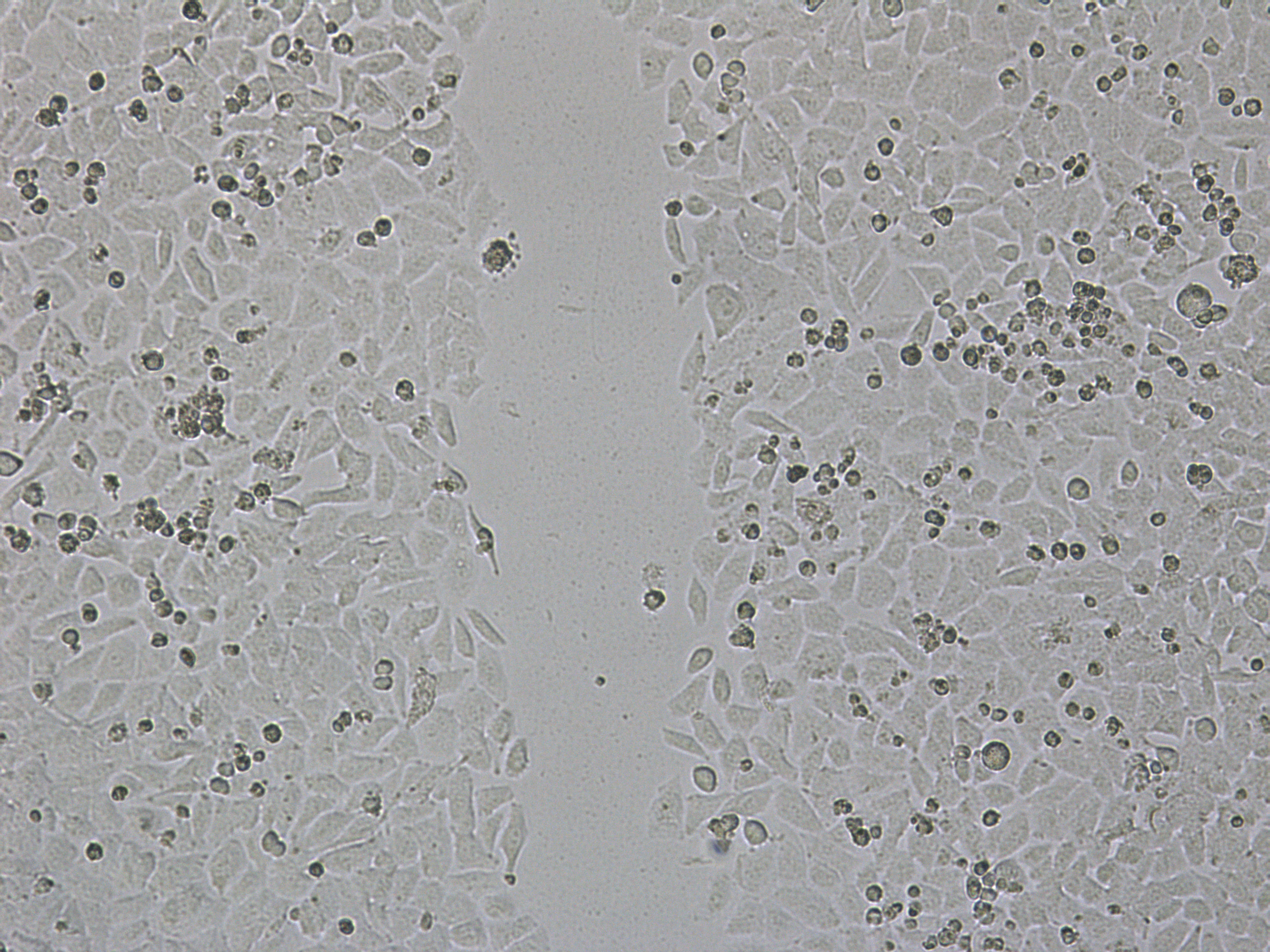

Supplement: S7 File — (ZIP) [file pone.0334639.s007.zip › S 12. File. Original FIgures. Fig.5/5k/HepG2 sh-NC 48H.jpg]

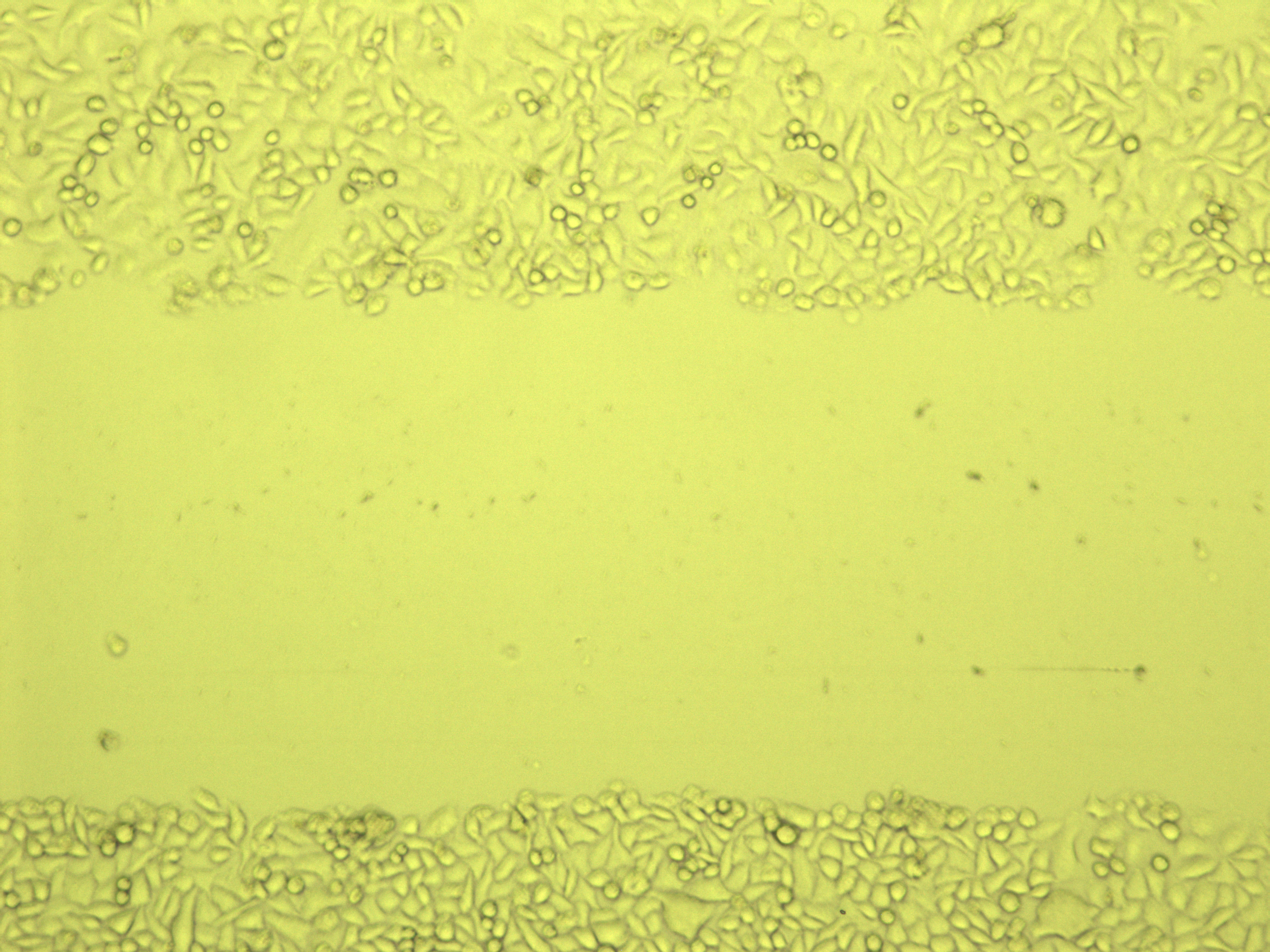

Supplement: S7 File — (ZIP) [file pone.0334639.s007.zip › S 12. File. Original FIgures. Fig.5/5l/SMMC-7721 sh-CXCL3 0h.jpg]

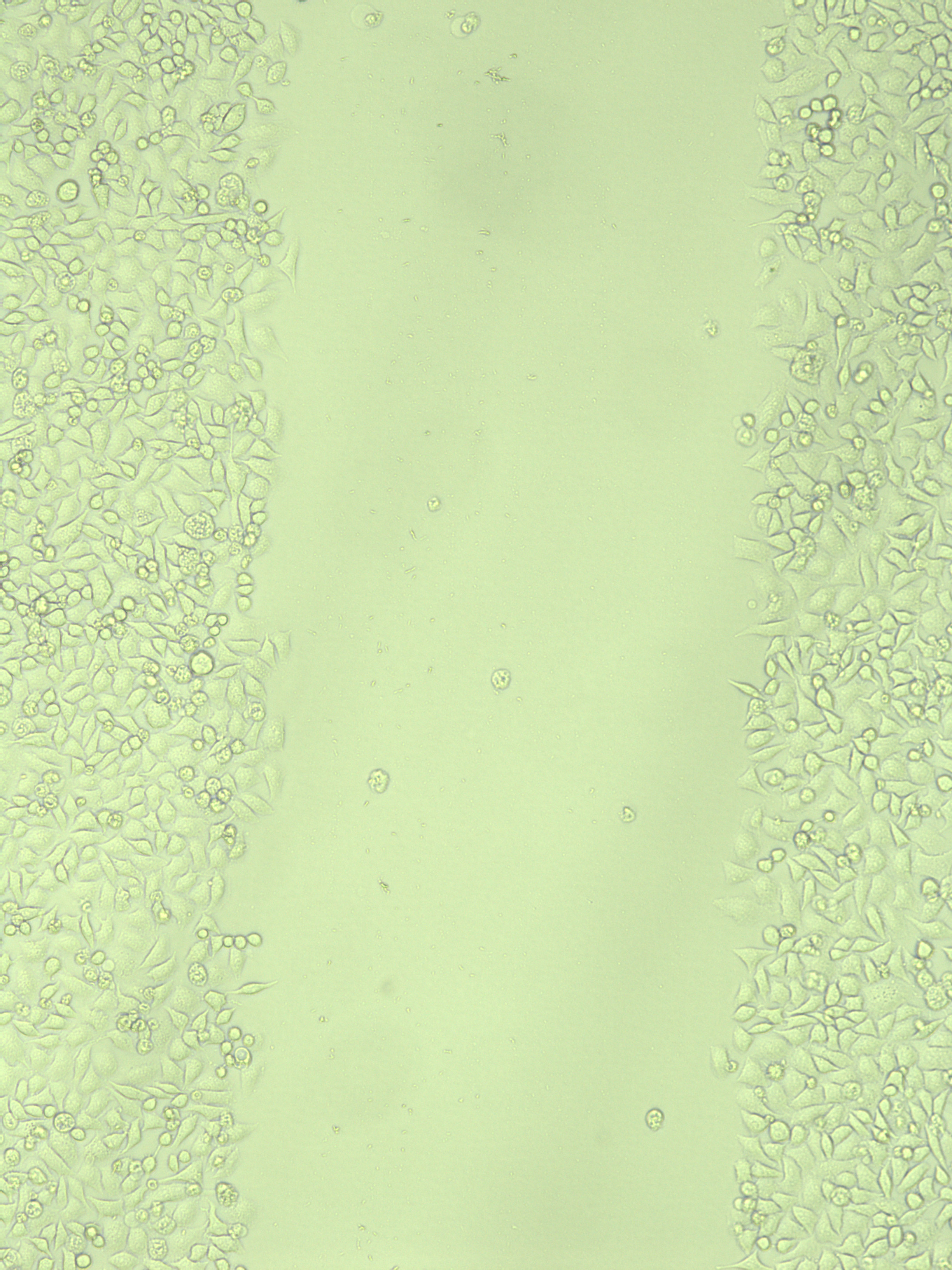

Supplement: S7 File — (ZIP) [file pone.0334639.s007.zip › S 12. File. Original FIgures. Fig.5/5l/SMMC-7721 sh-CXCL3 24h.jpg]

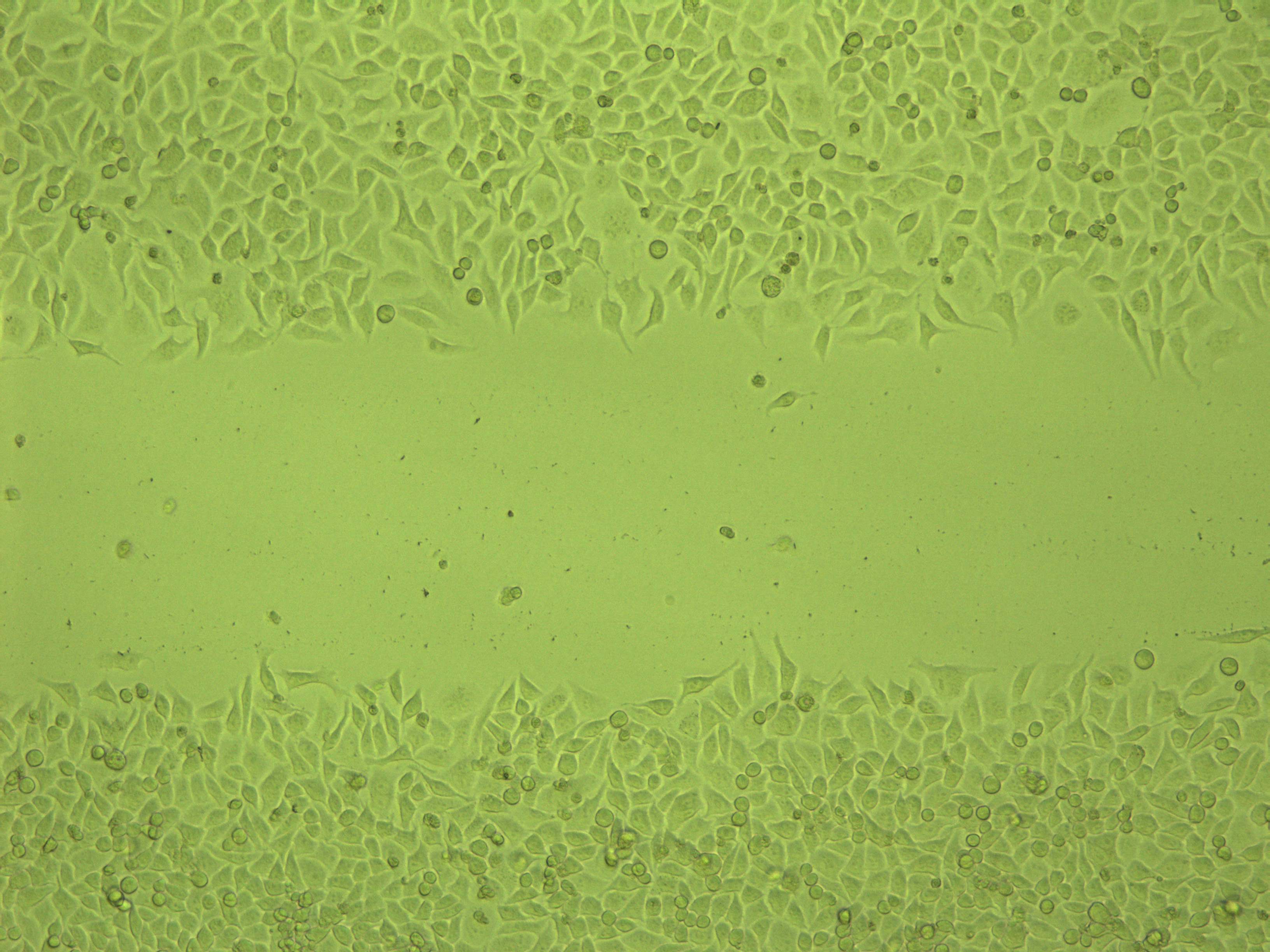

Supplement: S7 File — (ZIP) [file pone.0334639.s007.zip › S 12. File. Original FIgures. Fig.5/5l/SMMC-7721 sh-CXCL3 48h.jpg]

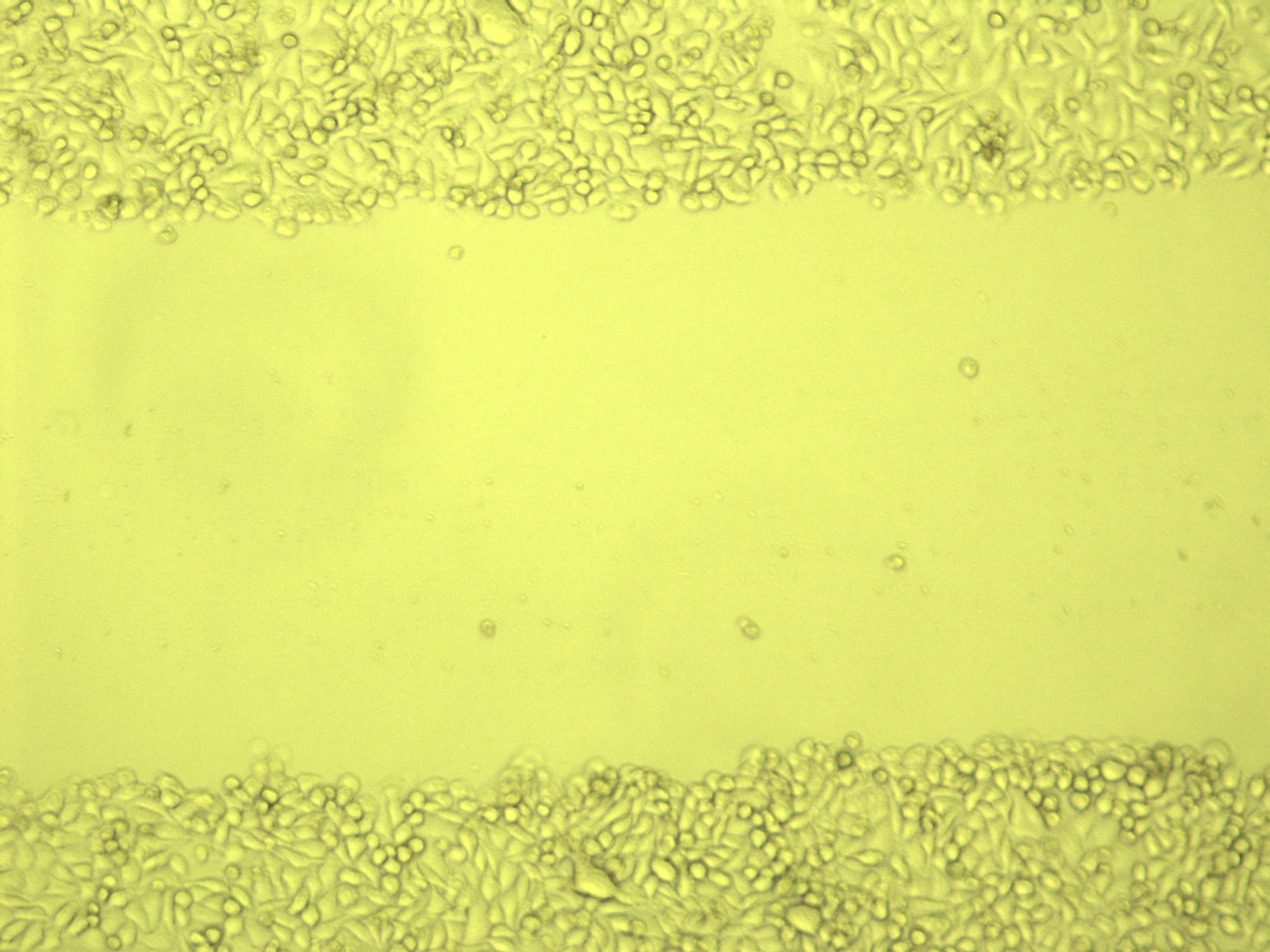

Supplement: S7 File — (ZIP) [file pone.0334639.s007.zip › S 12. File. Original FIgures. Fig.5/5l/SMMC-7721 sh-NC 0H.jpg]

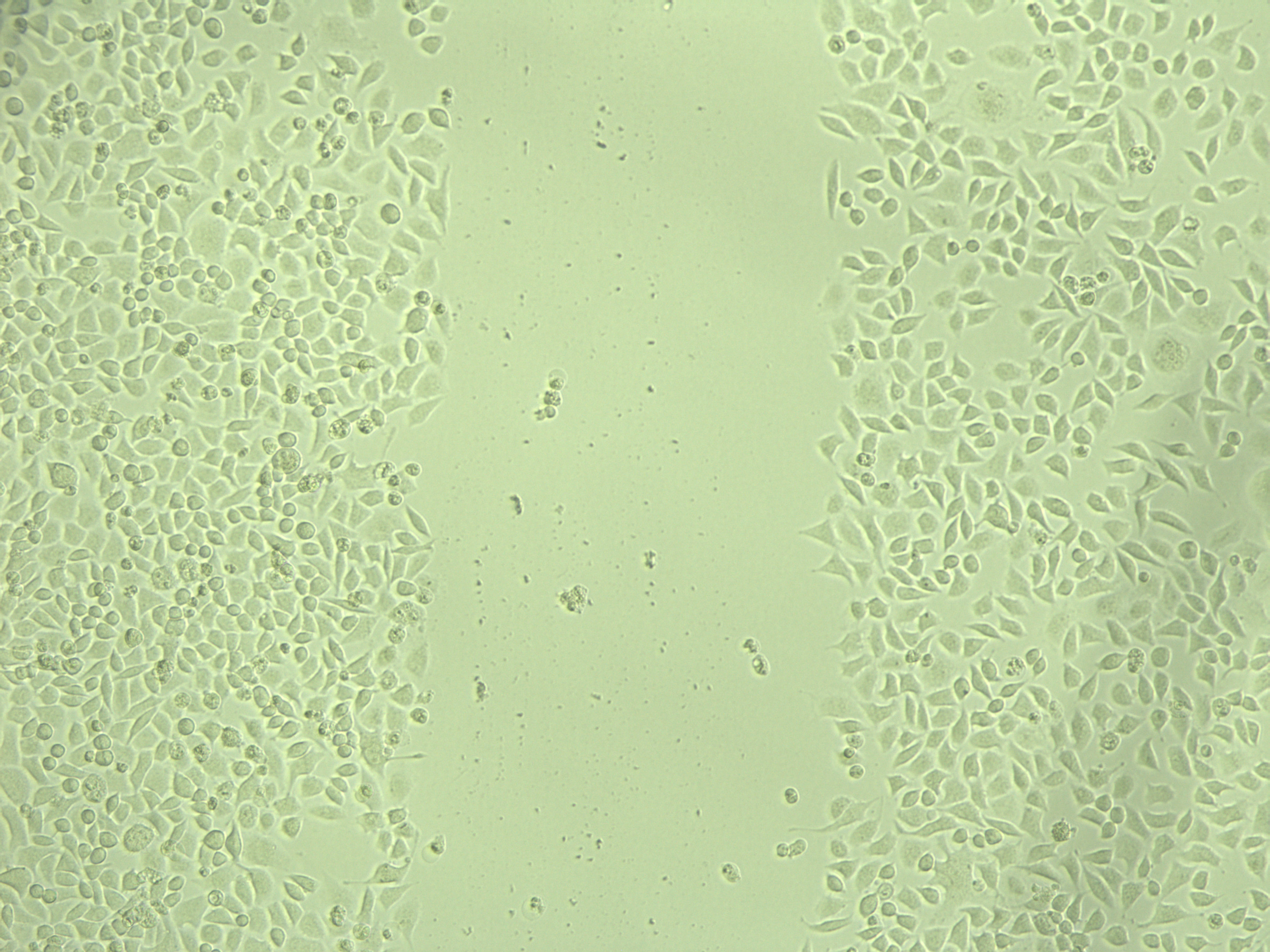

Supplement: S7 File — (ZIP) [file pone.0334639.s007.zip › S 12. File. Original FIgures. Fig.5/5l/SMMC-7721 sh-NC 24H.jpg]

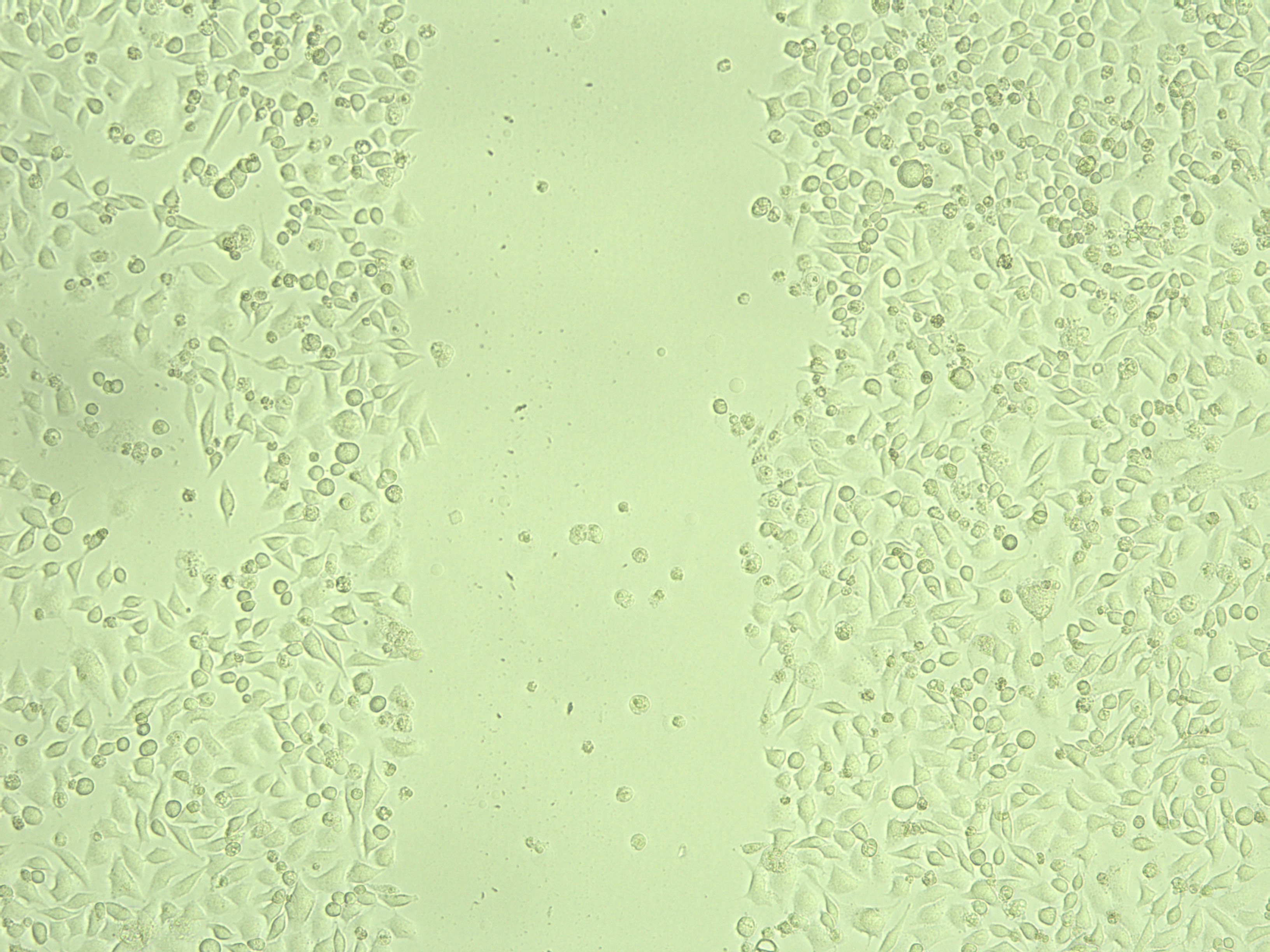

Supplement: S7 File — (ZIP) [file pone.0334639.s007.zip › S 12. File. Original FIgures. Fig.5/5l/SMMC-7721 sh-NC 48H.jpg]

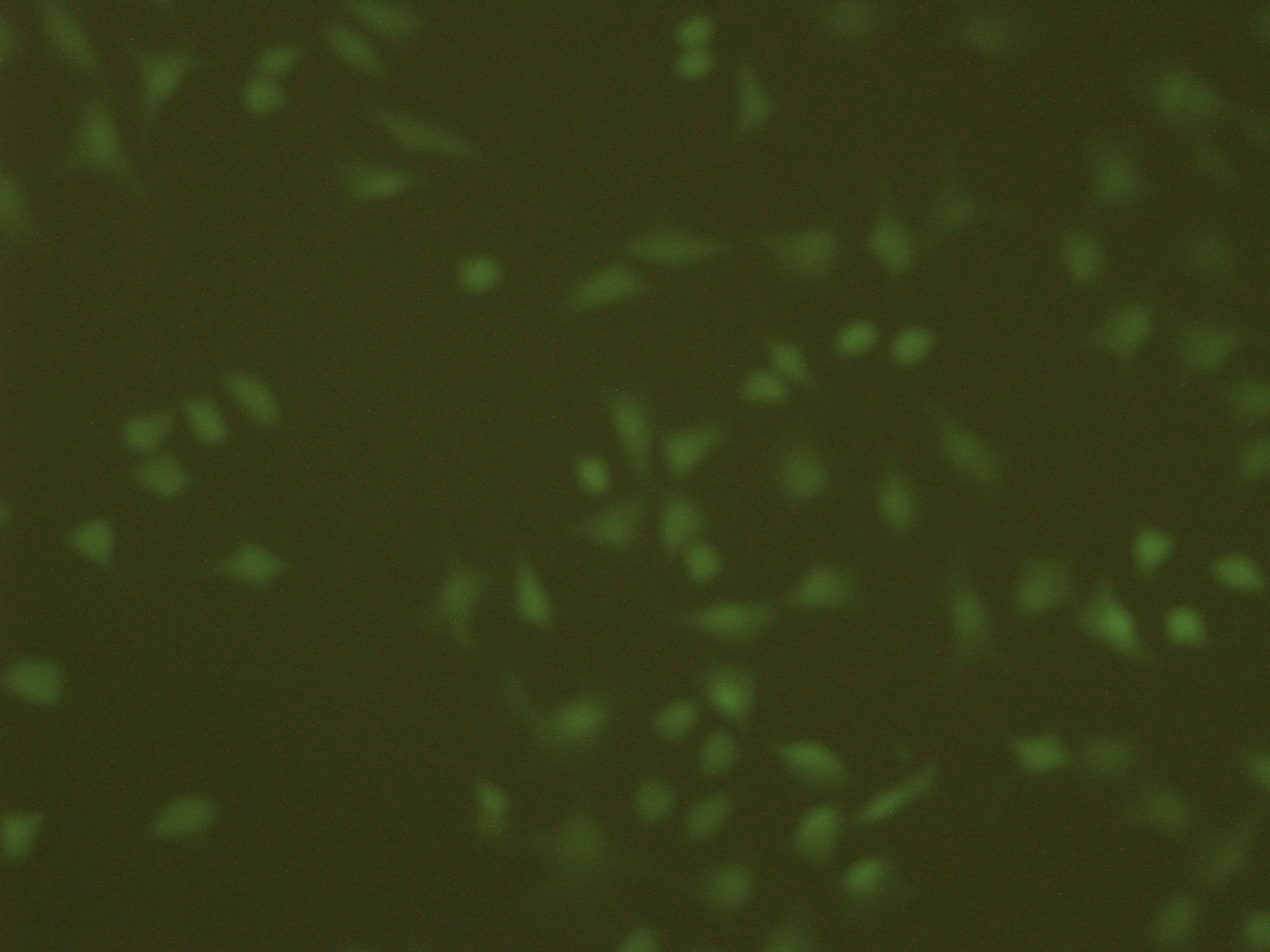

Supplement: S8 File — (ZIP) [file pone.0334639.s008.zip › S 13. File. Original Images. Fig6/S 13. File. Original FIgures. Fig.6/6a/MOCK/LX-2 CXCL3-MOCK-2.jpg]

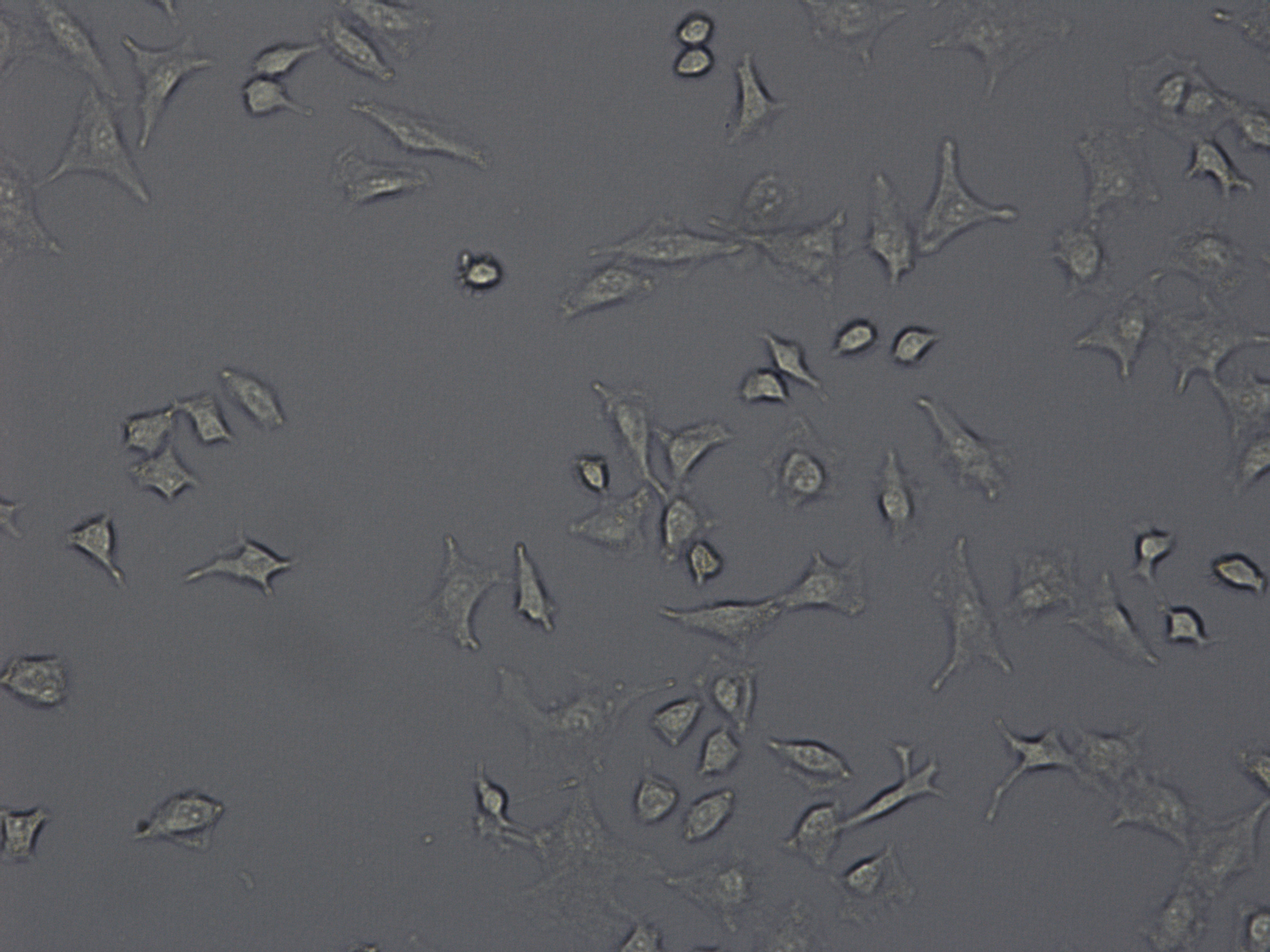

Supplement: S8 File — (ZIP) [file pone.0334639.s008.zip › S 13. File. Original Images. Fig6/S 13. File. Original FIgures. Fig.6/6a/MOCK/LX-2 CXCL3-MOCK.jpg]

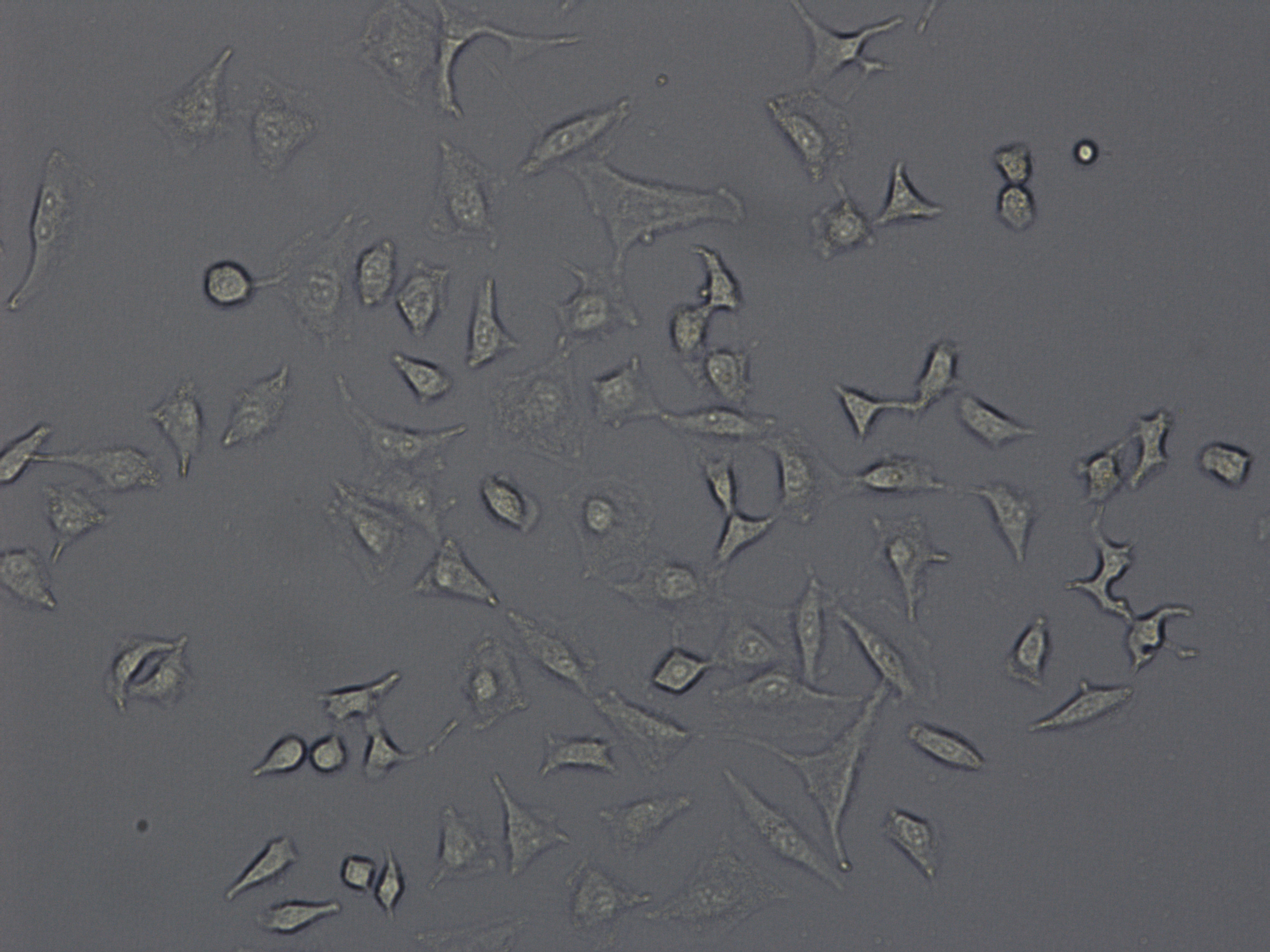

Supplement: S8 File — (ZIP) [file pone.0334639.s008.zip › S 13. File. Original Images. Fig6/S 13. File. Original FIgures. Fig.6/6a/OVEREXPRESSION/LX-2 CXCL3-A1113- 2-1.jpg]

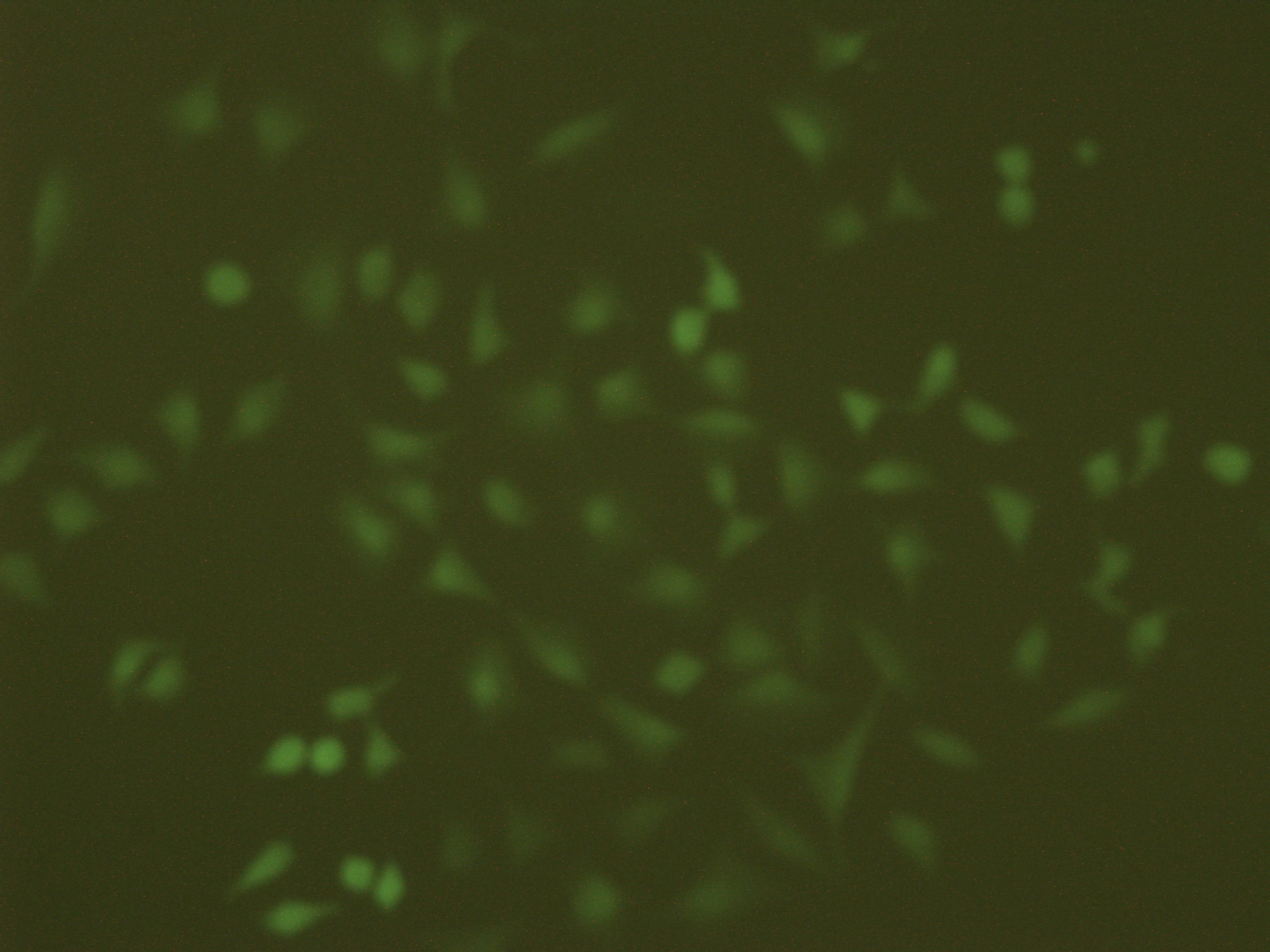

Supplement: S8 File — (ZIP) [file pone.0334639.s008.zip › S 13. File. Original Images. Fig6/S 13. File. Original FIgures. Fig.6/6a/OVEREXPRESSION/LX-2 CXCL3-A1113- 2-2.jpg]

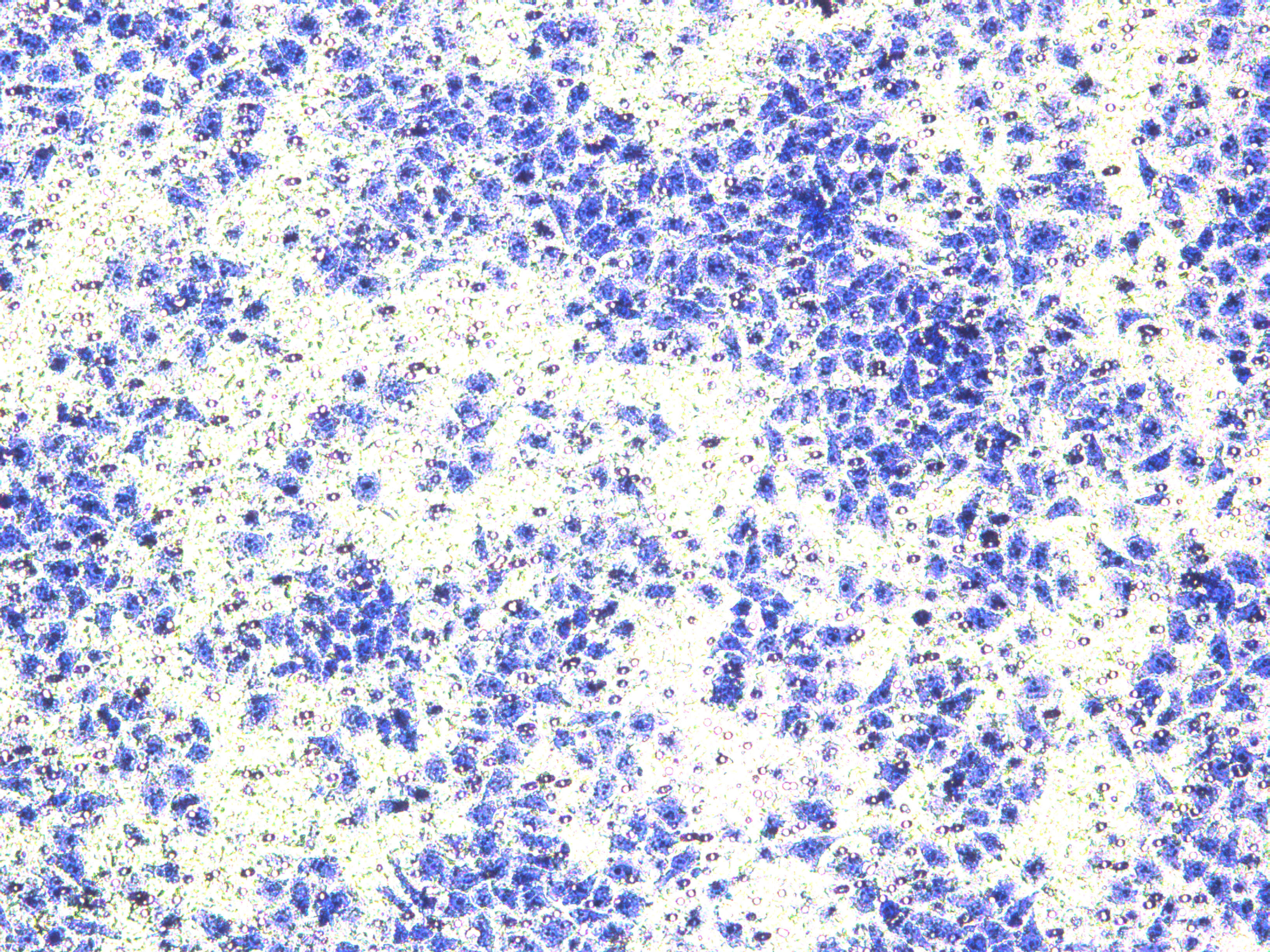

Supplement: S8 File — (ZIP) [file pone.0334639.s008.zip › S 13. File. Original Images. Fig6/S 13. File. Original FIgures. Fig.6/6e/bel-7402/A/BEL-7402 LX-2 0%.jpg]

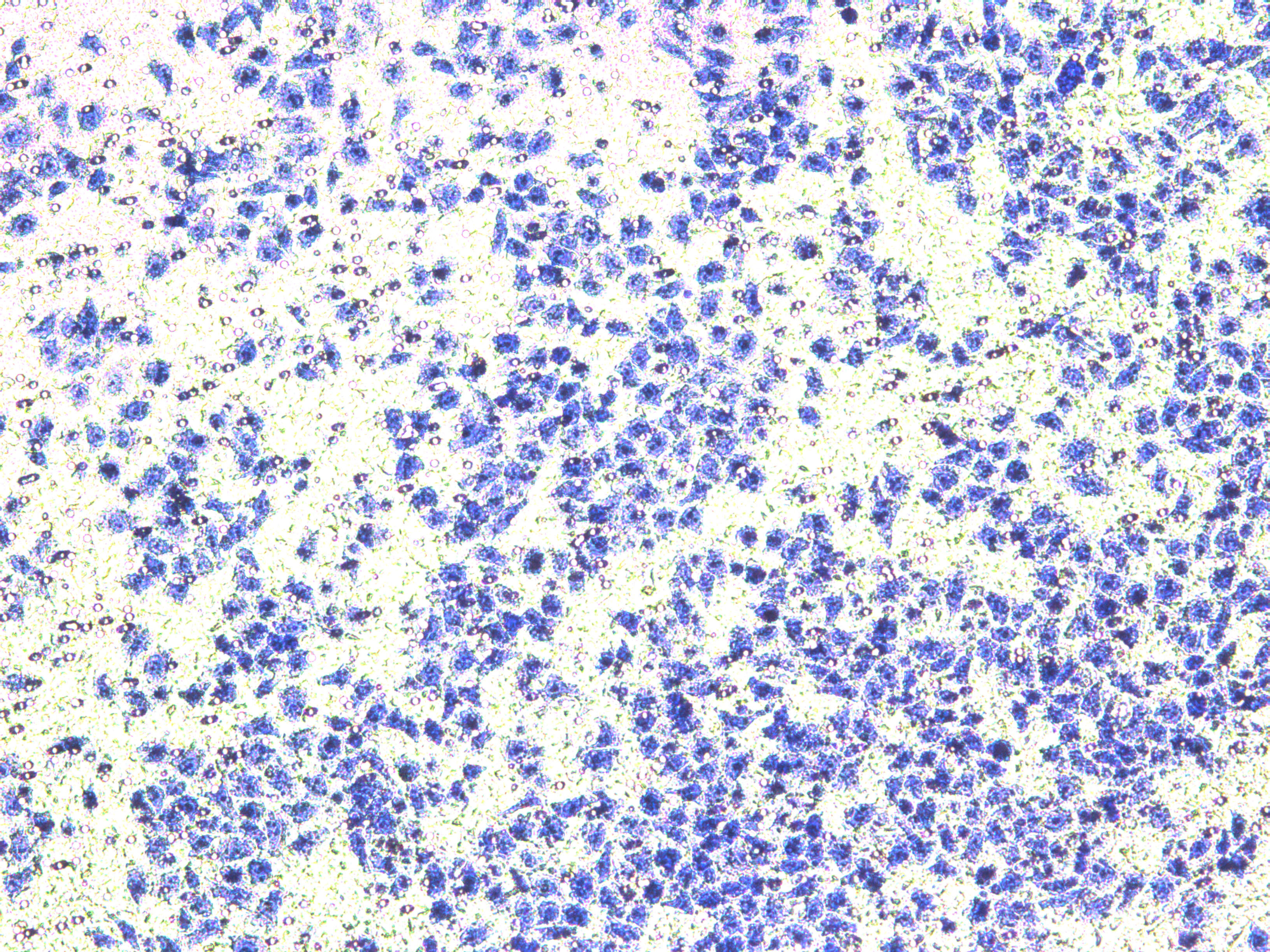

Supplement: S8 File — (ZIP) [file pone.0334639.s008.zip › S 13. File. Original Images. Fig6/S 13. File. Original FIgures. Fig.6/6e/bel-7402/A/BEL-7402 LX-2 20%.jpg]

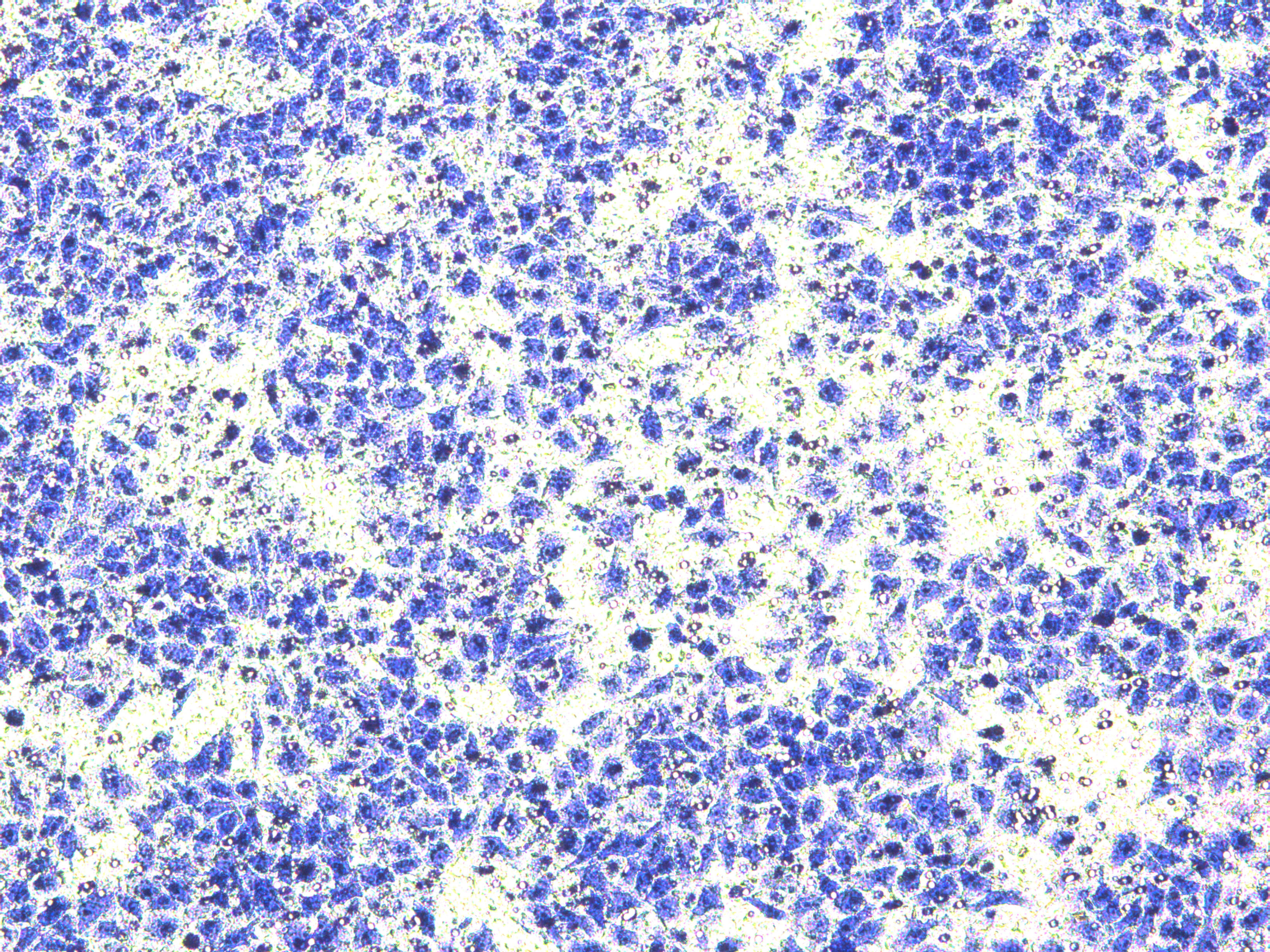

Supplement: S8 File — (ZIP) [file pone.0334639.s008.zip › S 13. File. Original Images. Fig6/S 13. File. Original FIgures. Fig.6/6e/bel-7402/A/BEL-7402 LX-2 40%.jpg]

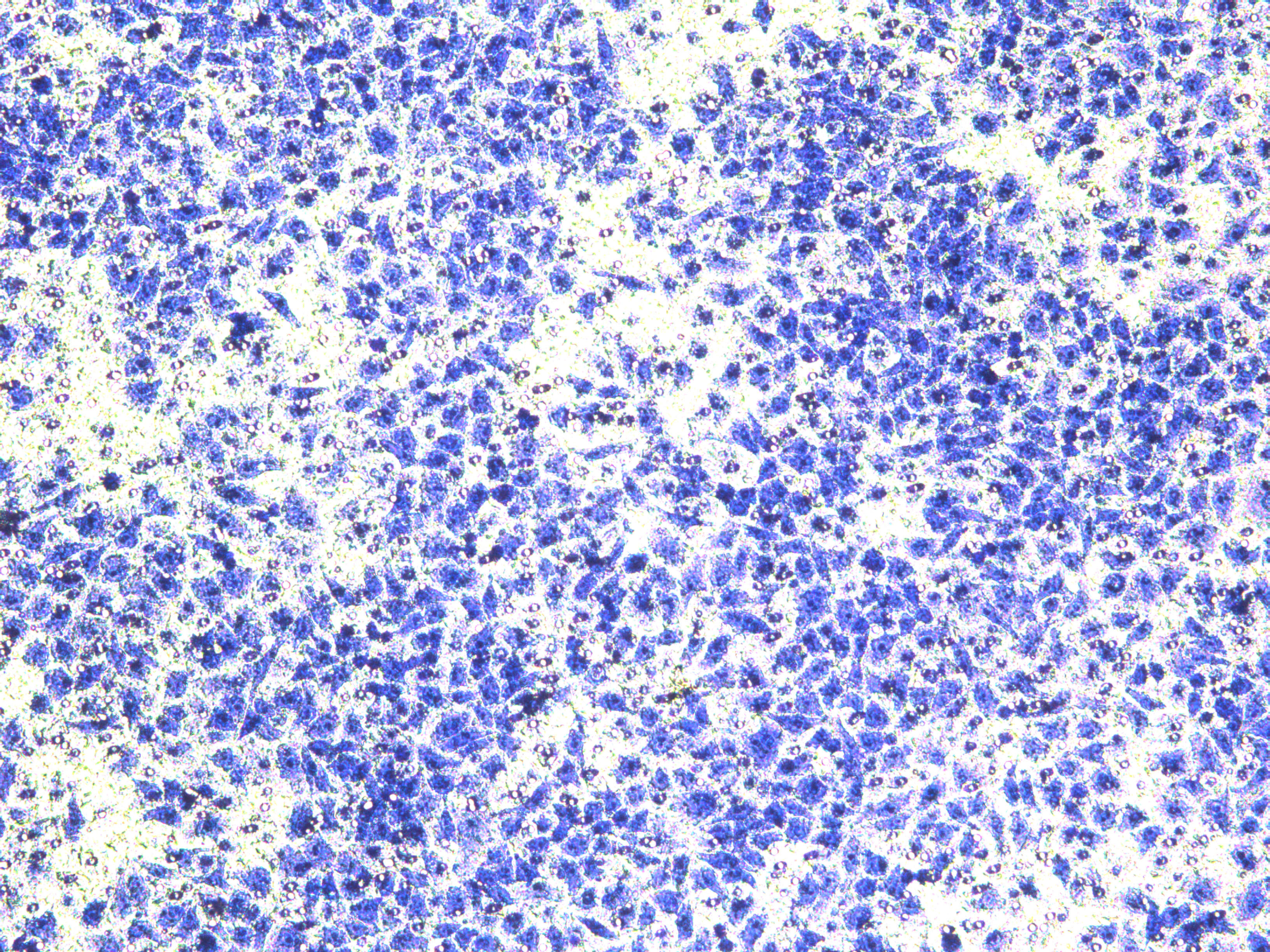

Supplement: S8 File — (ZIP) [file pone.0334639.s008.zip › S 13. File. Original Images. Fig6/S 13. File. Original FIgures. Fig.6/6e/bel-7402/A/BEL-7402 LX-2 60%.jpg]

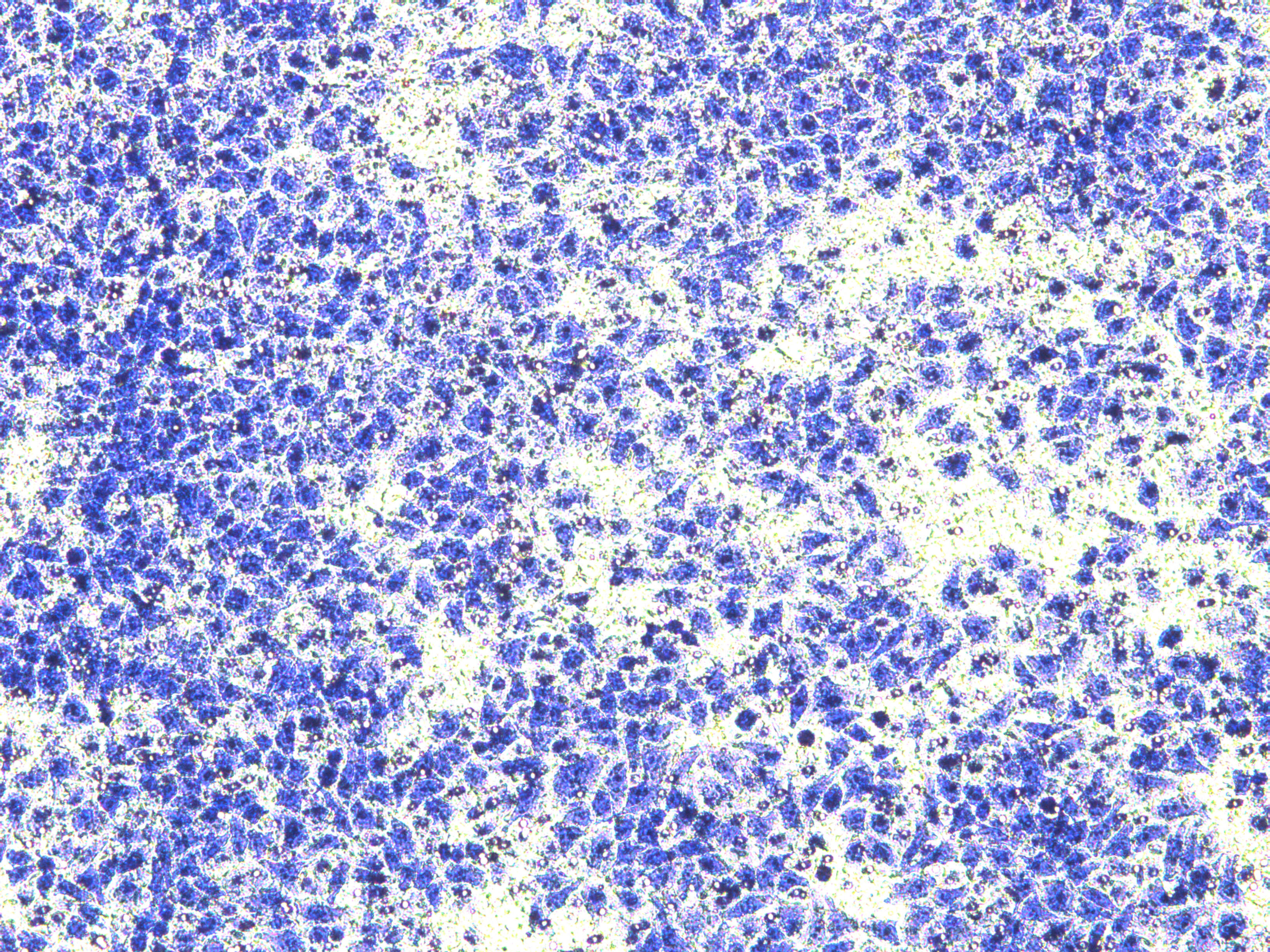

Supplement: S8 File — (ZIP) [file pone.0334639.s008.zip › S 13. File. Original Images. Fig6/S 13. File. Original FIgures. Fig.6/6e/bel-7402/A/BEL-7402 LX-2 80%.jpg]

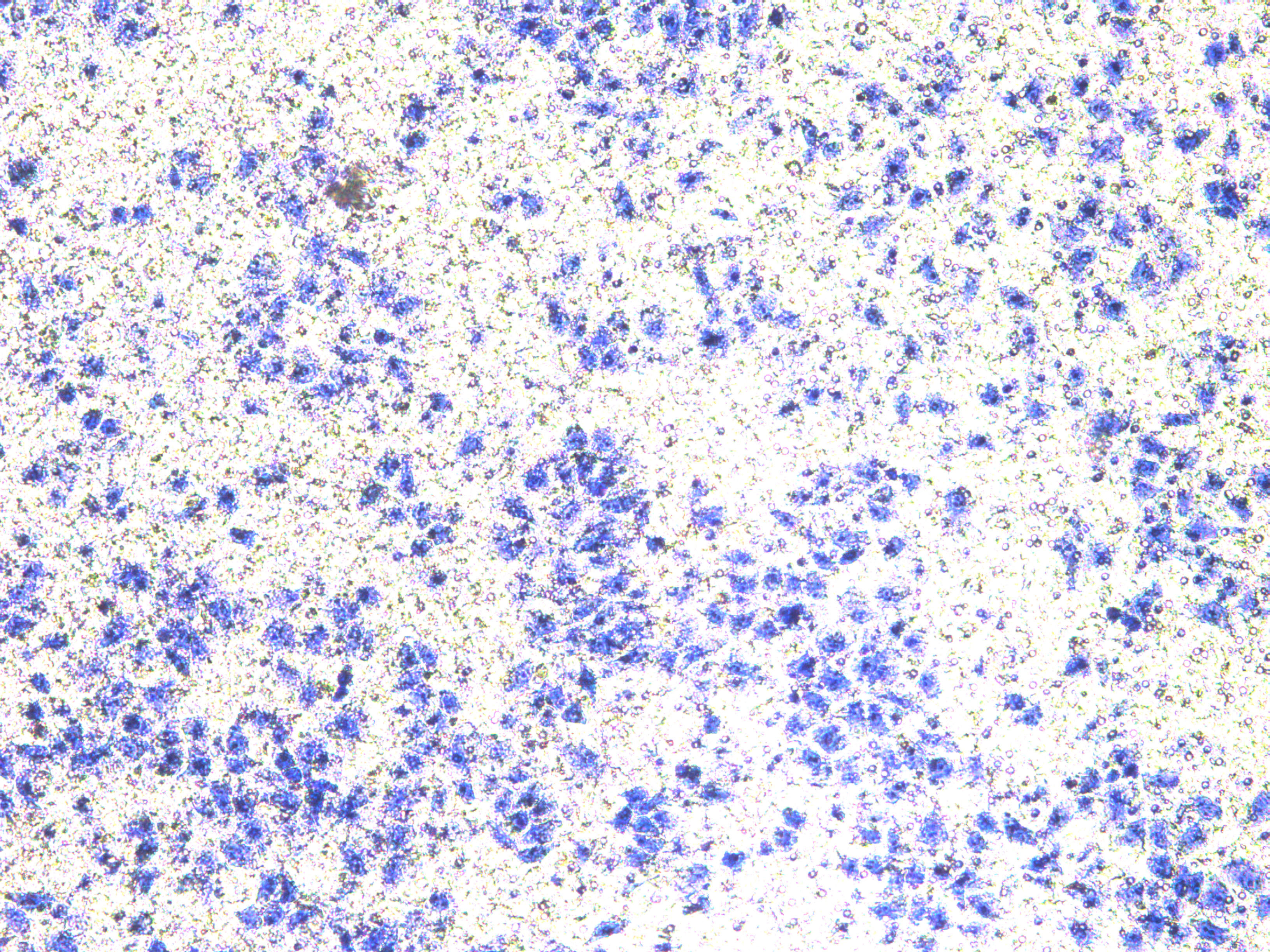

Supplement: S8 File — (ZIP) [file pone.0334639.s008.zip › S 13. File. Original Images. Fig6/S 13. File. Original FIgures. Fig.6/6e/bel-7402/N/BEL-7402 LX-2 0%.jpg]

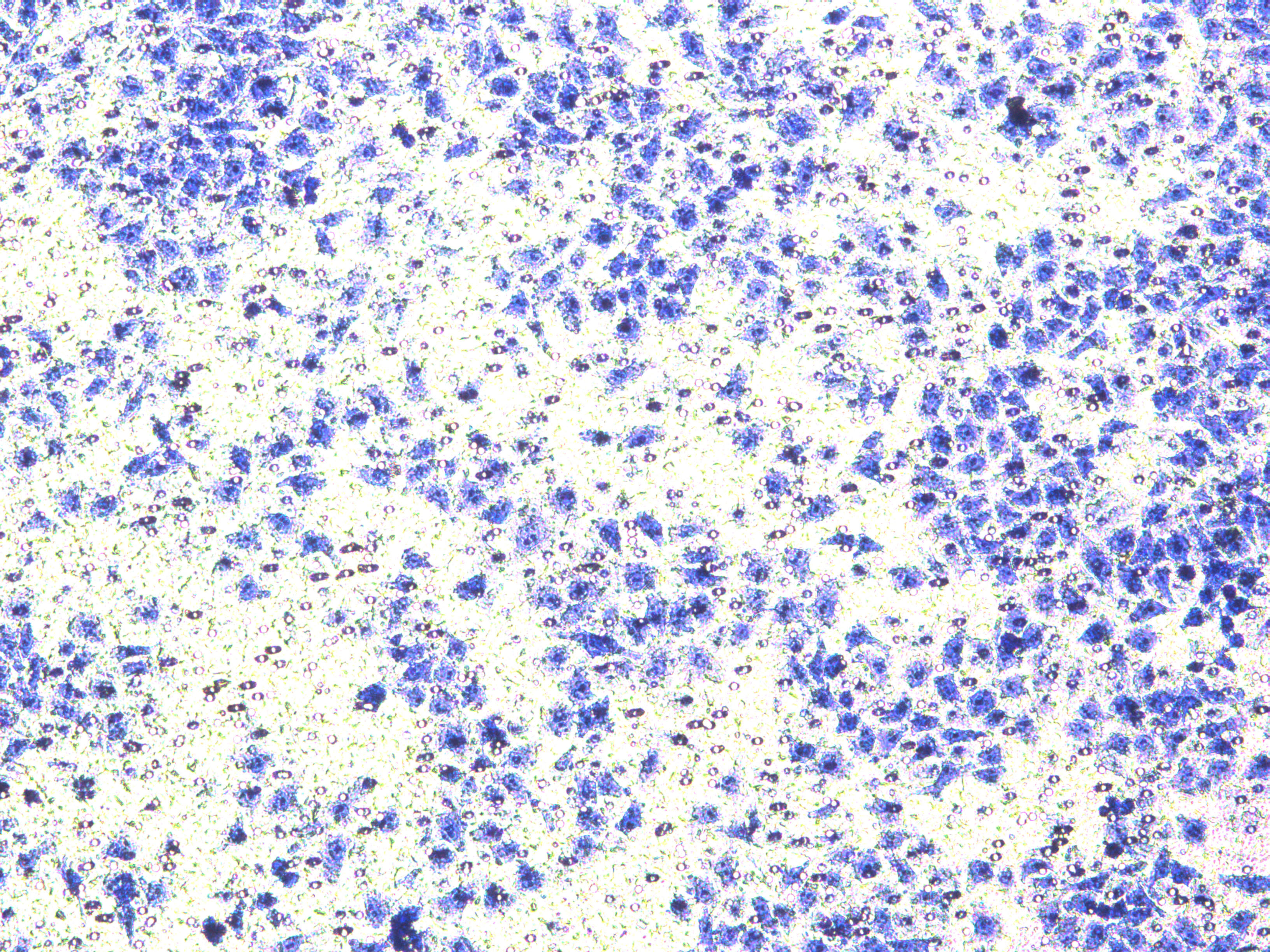

Supplement: S8 File — (ZIP) [file pone.0334639.s008.zip › S 13. File. Original Images. Fig6/S 13. File. Original FIgures. Fig.6/6e/bel-7402/N/BEL-7402 LX-2 20%.jpg]

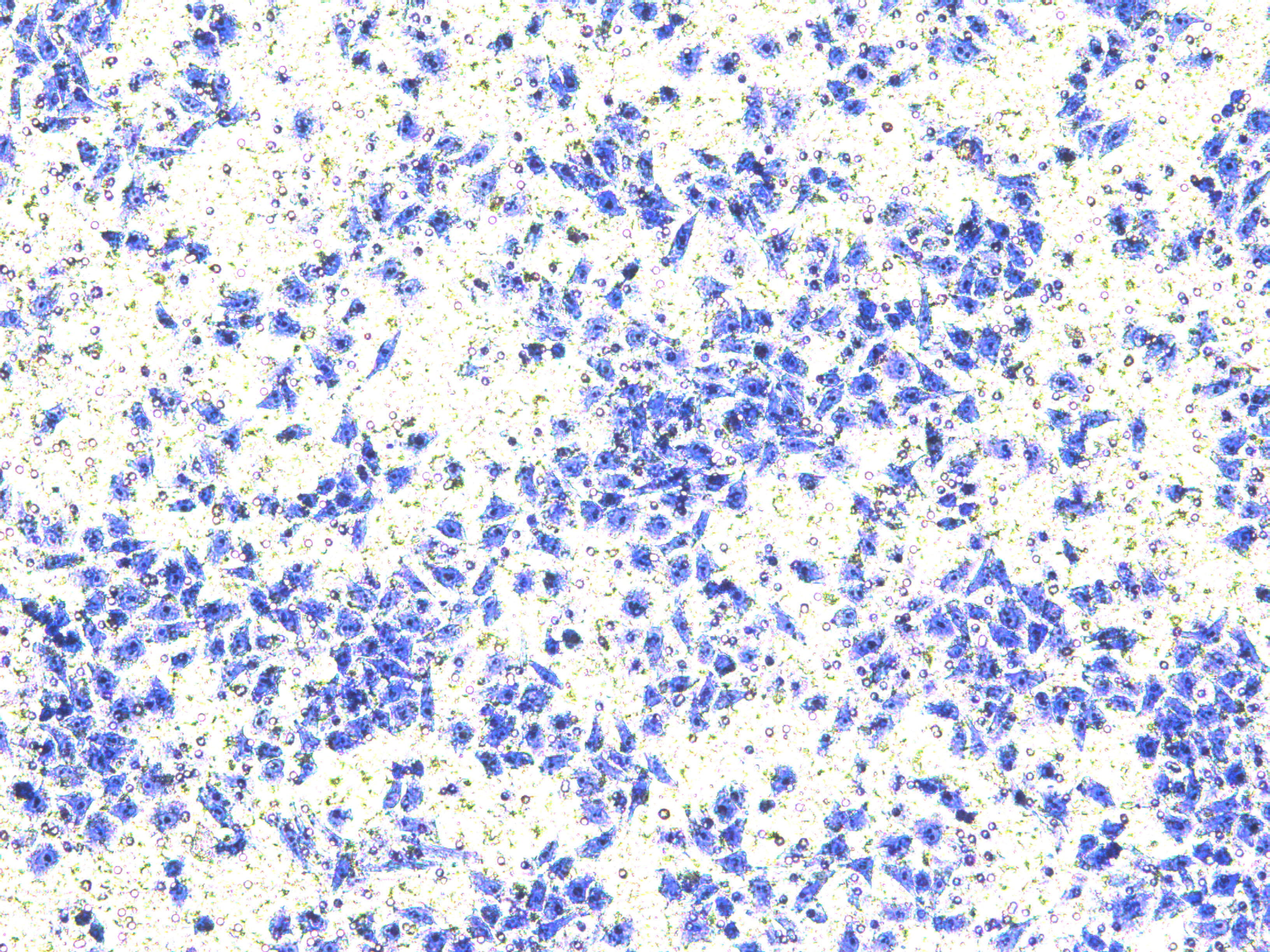

Supplement: S8 File — (ZIP) [file pone.0334639.s008.zip › S 13. File. Original Images. Fig6/S 13. File. Original FIgures. Fig.6/6e/bel-7402/N/BEL-7402 LX-2 40%.jpg]

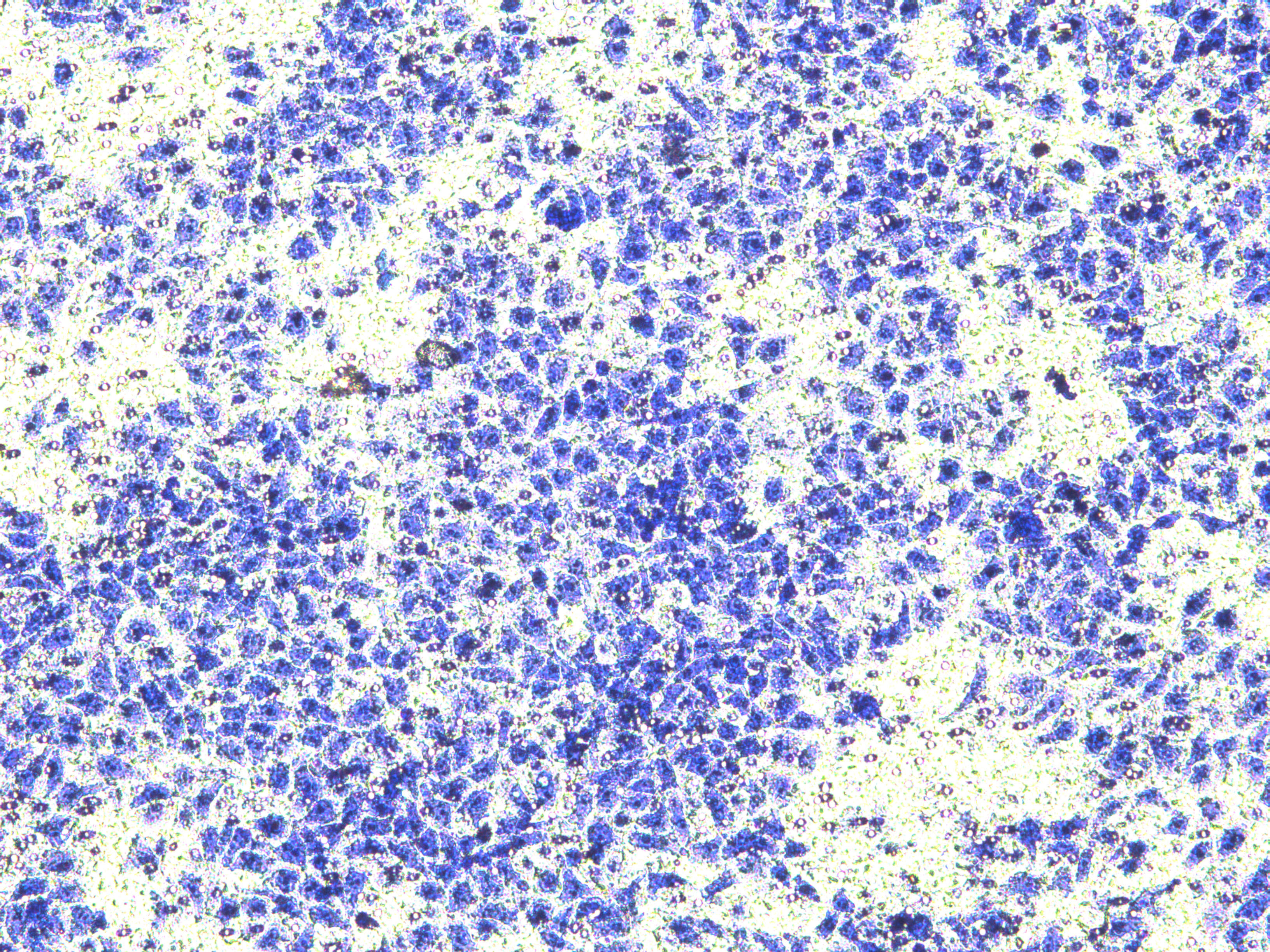

Supplement: S8 File — (ZIP) [file pone.0334639.s008.zip › S 13. File. Original Images. Fig6/S 13. File. Original FIgures. Fig.6/6e/bel-7402/N/BEL-7402 LX-2 60%.jpg]

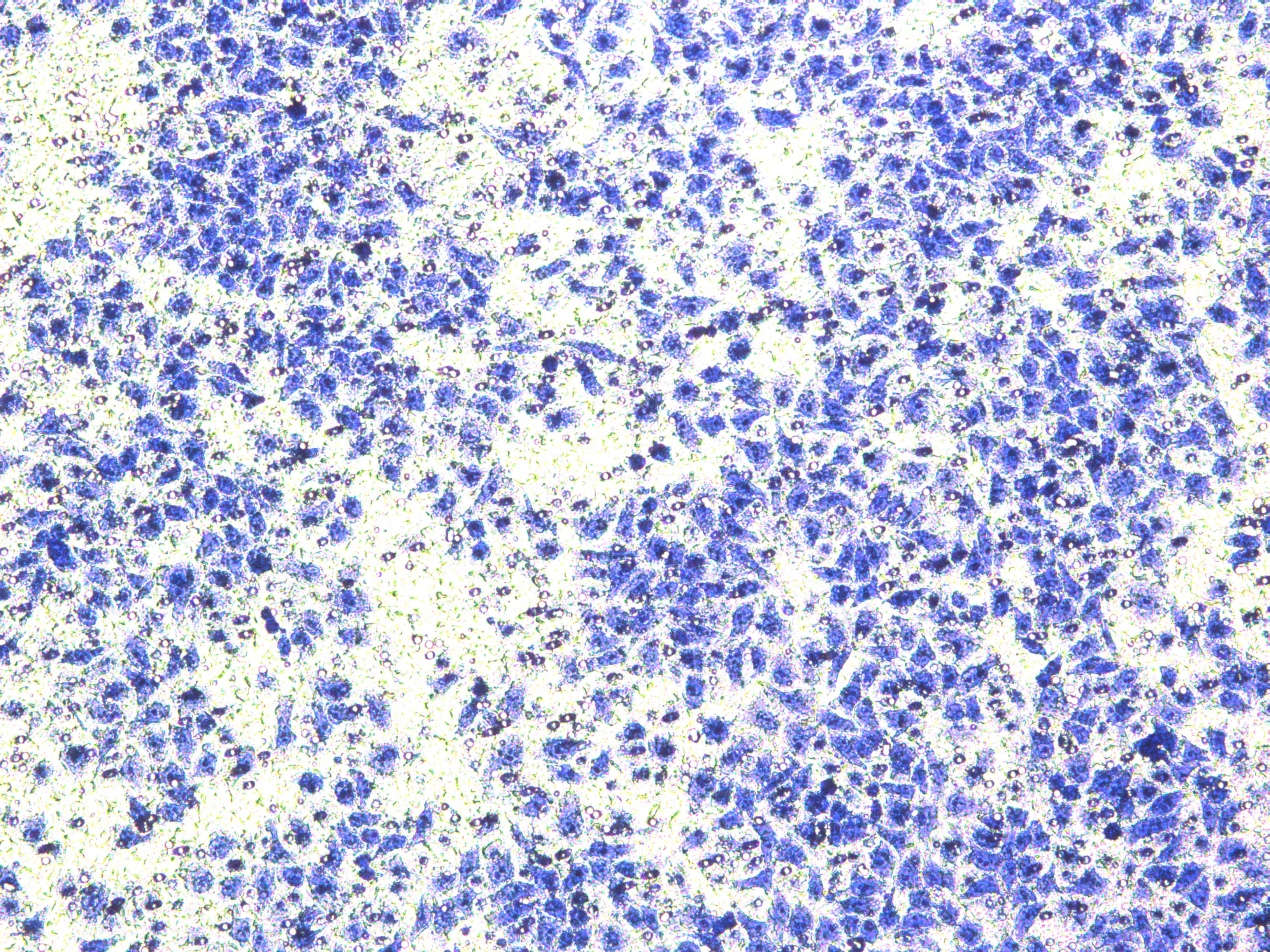

Supplement: S8 File — (ZIP) [file pone.0334639.s008.zip › S 13. File. Original Images. Fig6/S 13. File. Original FIgures. Fig.6/6e/bel-7402/N/BEL-7402 LX-2 80%.jpg]

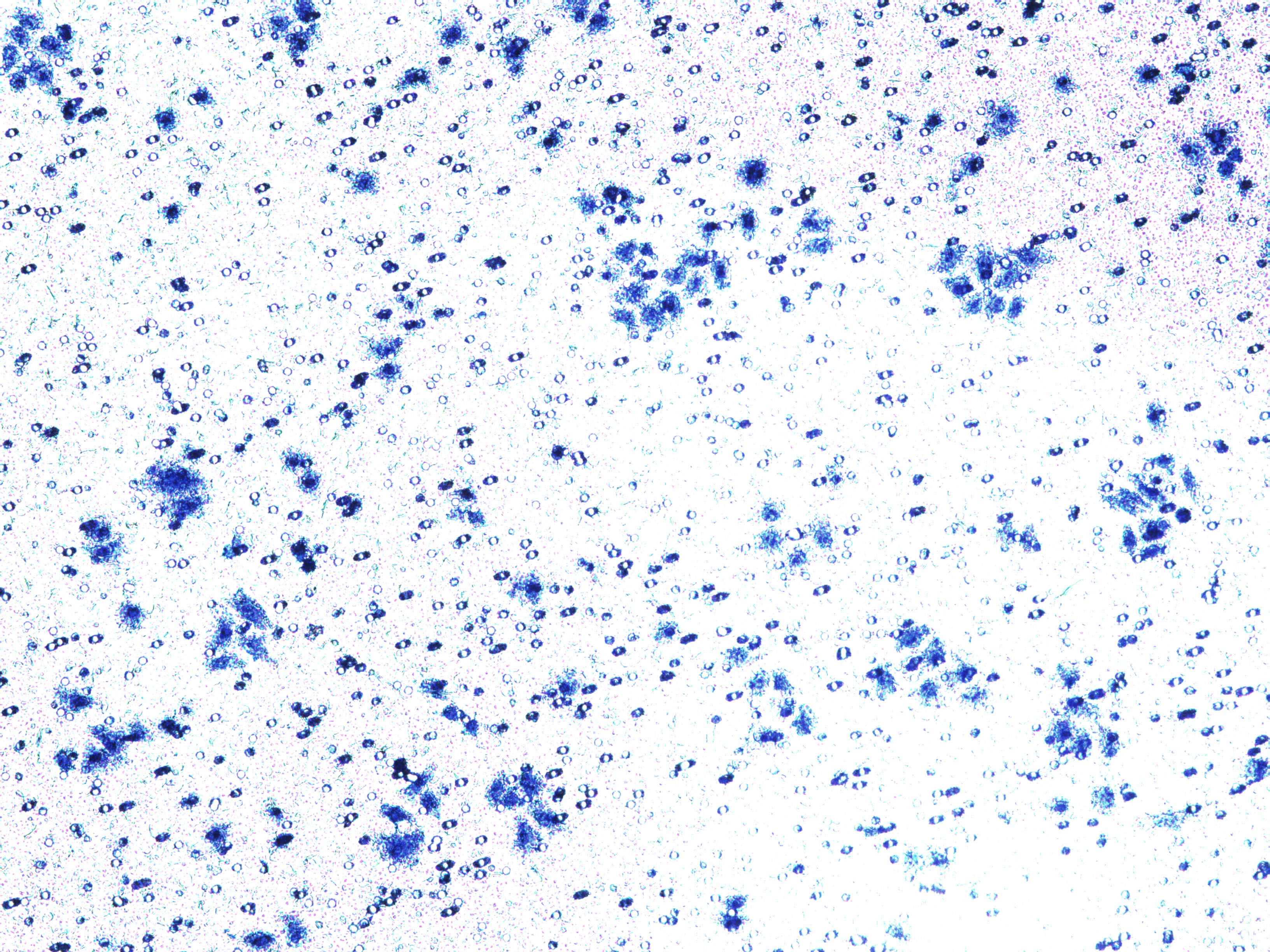

Supplement: S8 File — (ZIP) [file pone.0334639.s008.zip › S 13. File. Original Images. Fig6/S 13. File. Original FIgures. Fig.6/6f/HEPG2/A/hepG2 LX2 A-20%.jpg]

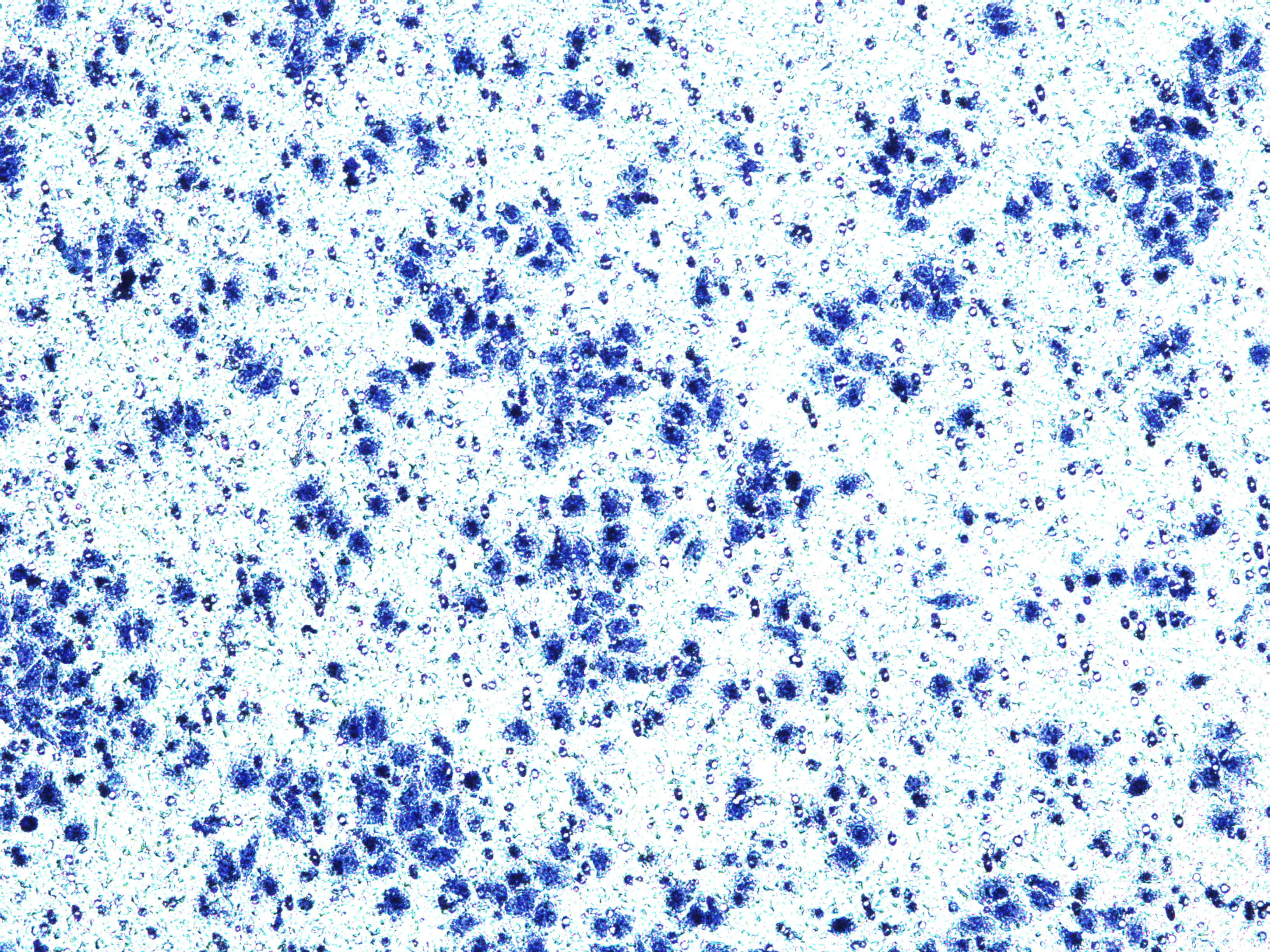

Supplement: S8 File — (ZIP) [file pone.0334639.s008.zip › S 13. File. Original Images. Fig6/S 13. File. Original FIgures. Fig.6/6f/HEPG2/A/hepG2 LX2 A-80%.jpg]

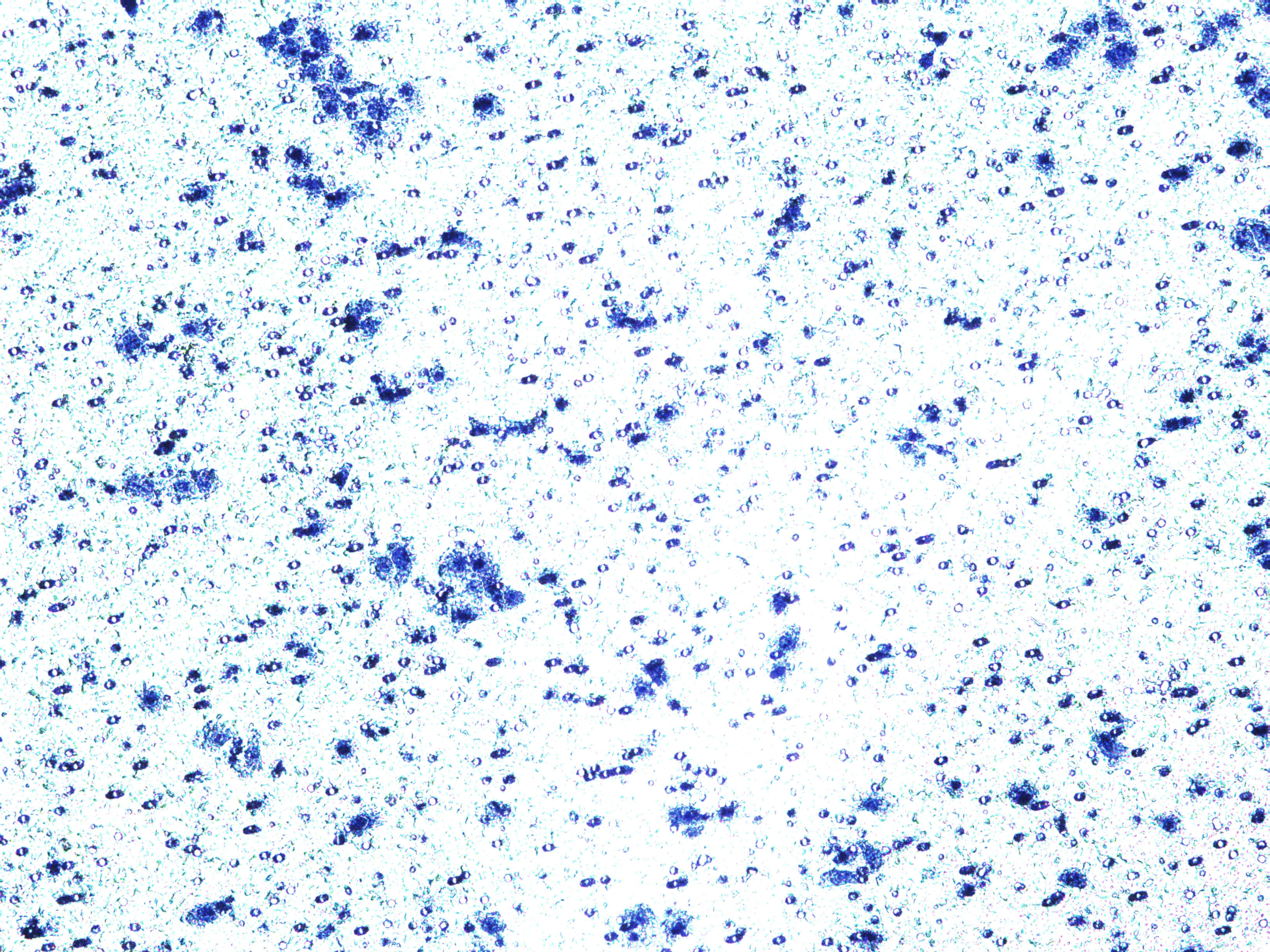

Supplement: S8 File — (ZIP) [file pone.0334639.s008.zip › S 13. File. Original Images. Fig6/S 13. File. Original FIgures. Fig.6/6f/HEPG2/A/hepG2 LX2 A0%.jpg]

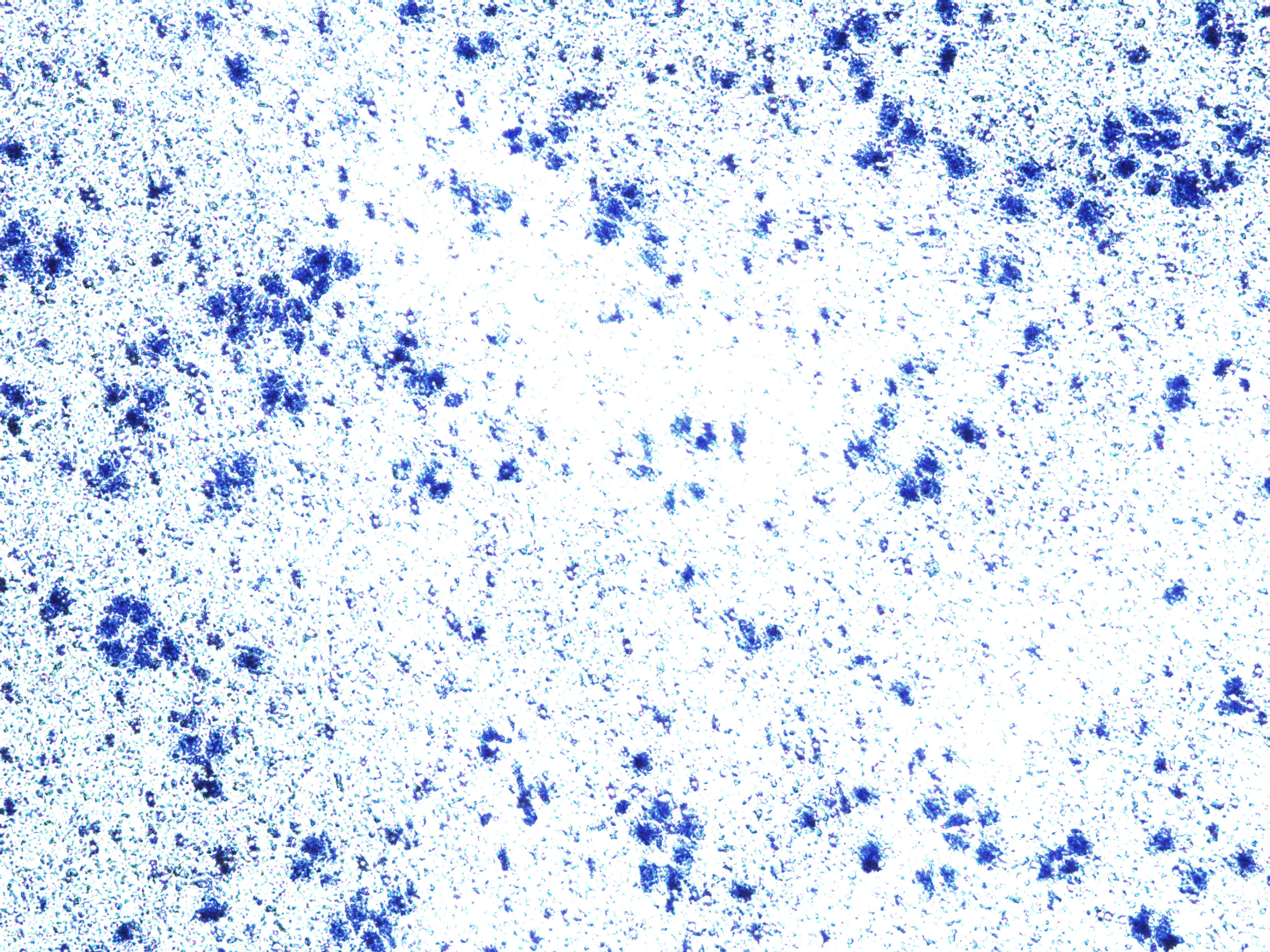

Supplement: S8 File — (ZIP) [file pone.0334639.s008.zip › S 13. File. Original Images. Fig6/S 13. File. Original FIgures. Fig.6/6f/HEPG2/A/hepG2 LX2 A40%).jpg]

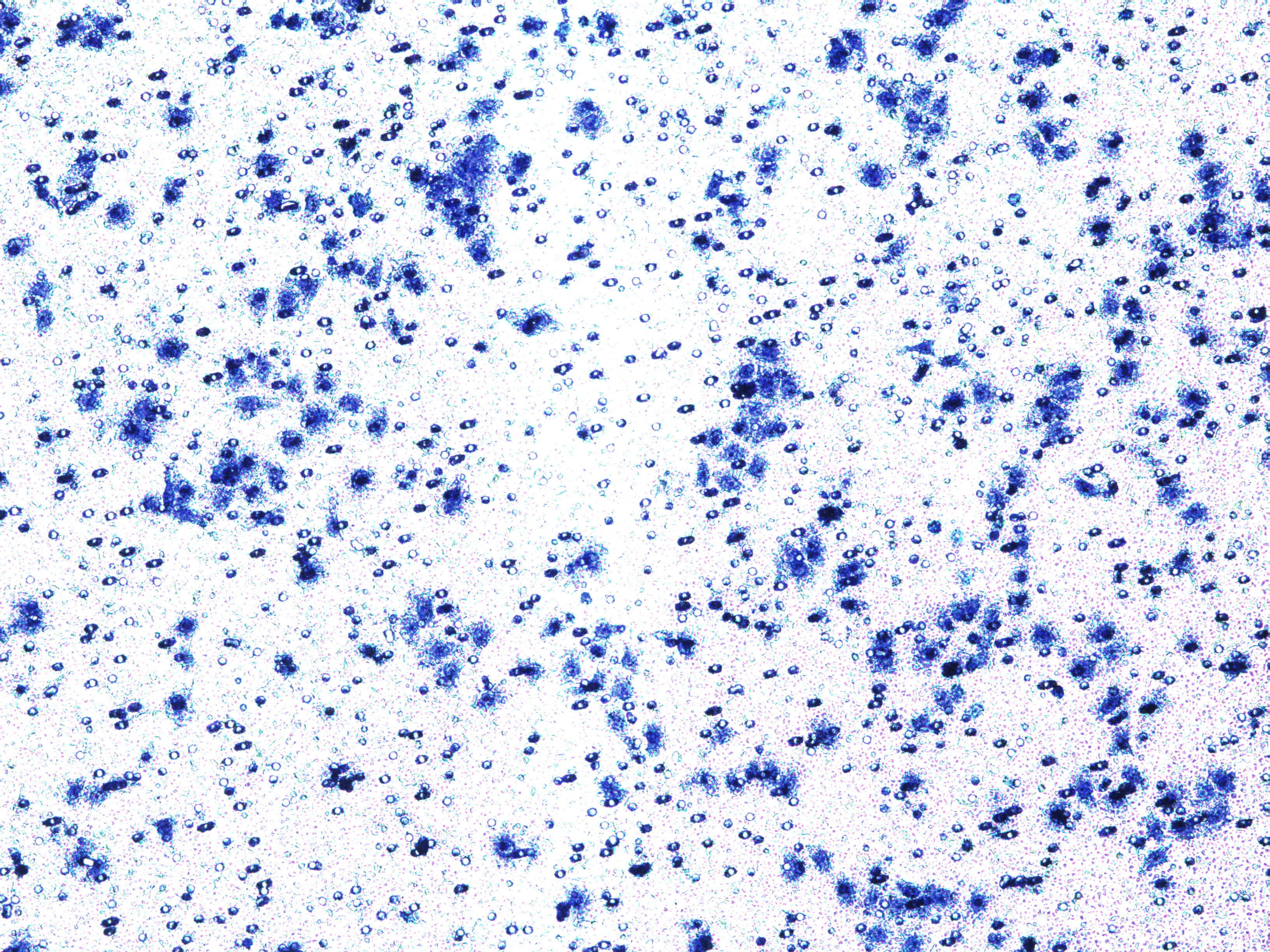

Supplement: S8 File — (ZIP) [file pone.0334639.s008.zip › S 13. File. Original Images. Fig6/S 13. File. Original FIgures. Fig.6/6f/HEPG2/A/hepG2 LX2 A60%.jpg]

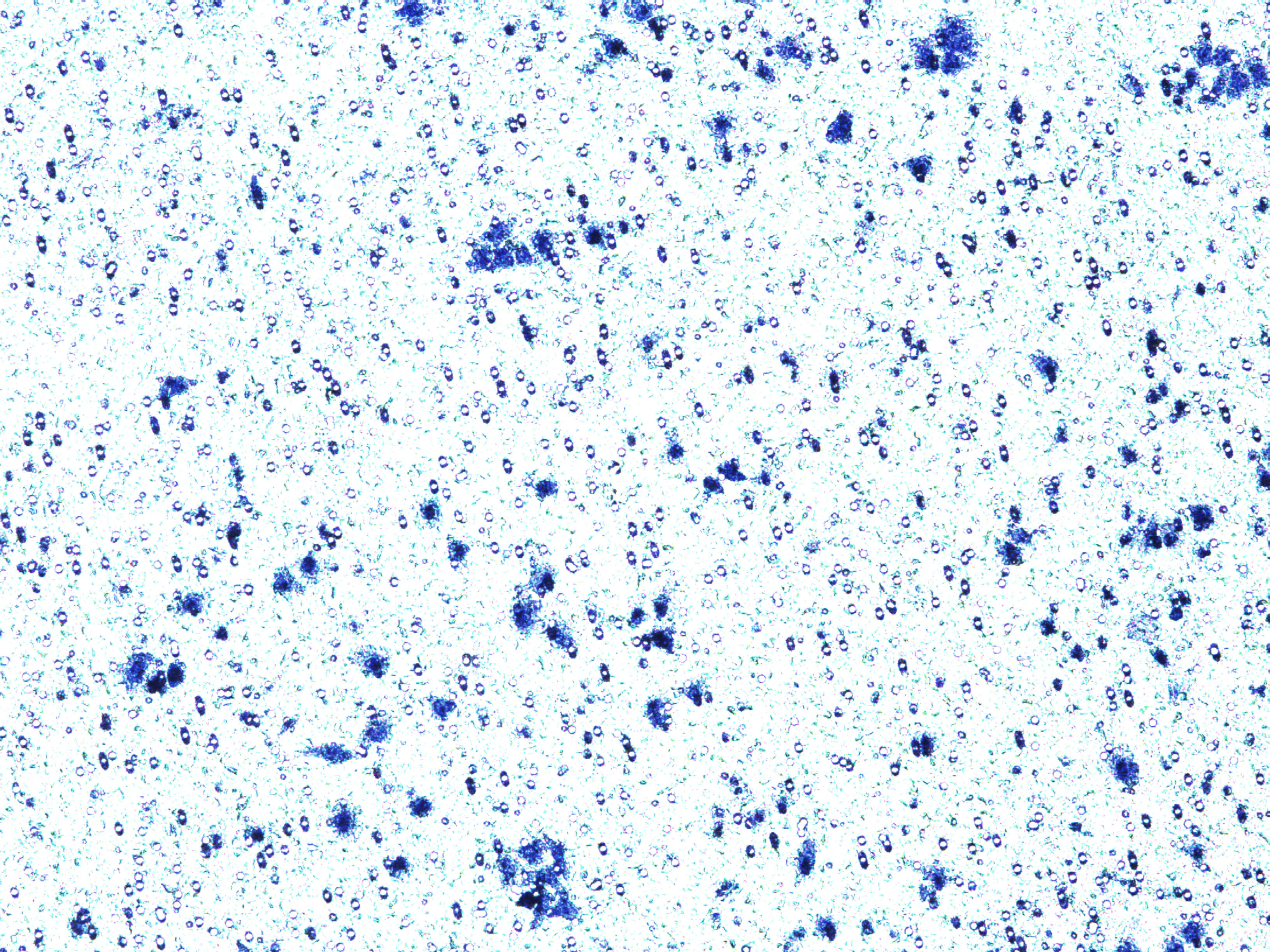

Supplement: S8 File — (ZIP) [file pone.0334639.s008.zip › S 13. File. Original Images. Fig6/S 13. File. Original FIgures. Fig.6/6f/HEPG2/N/hepG2 LX2 0%.jpg]

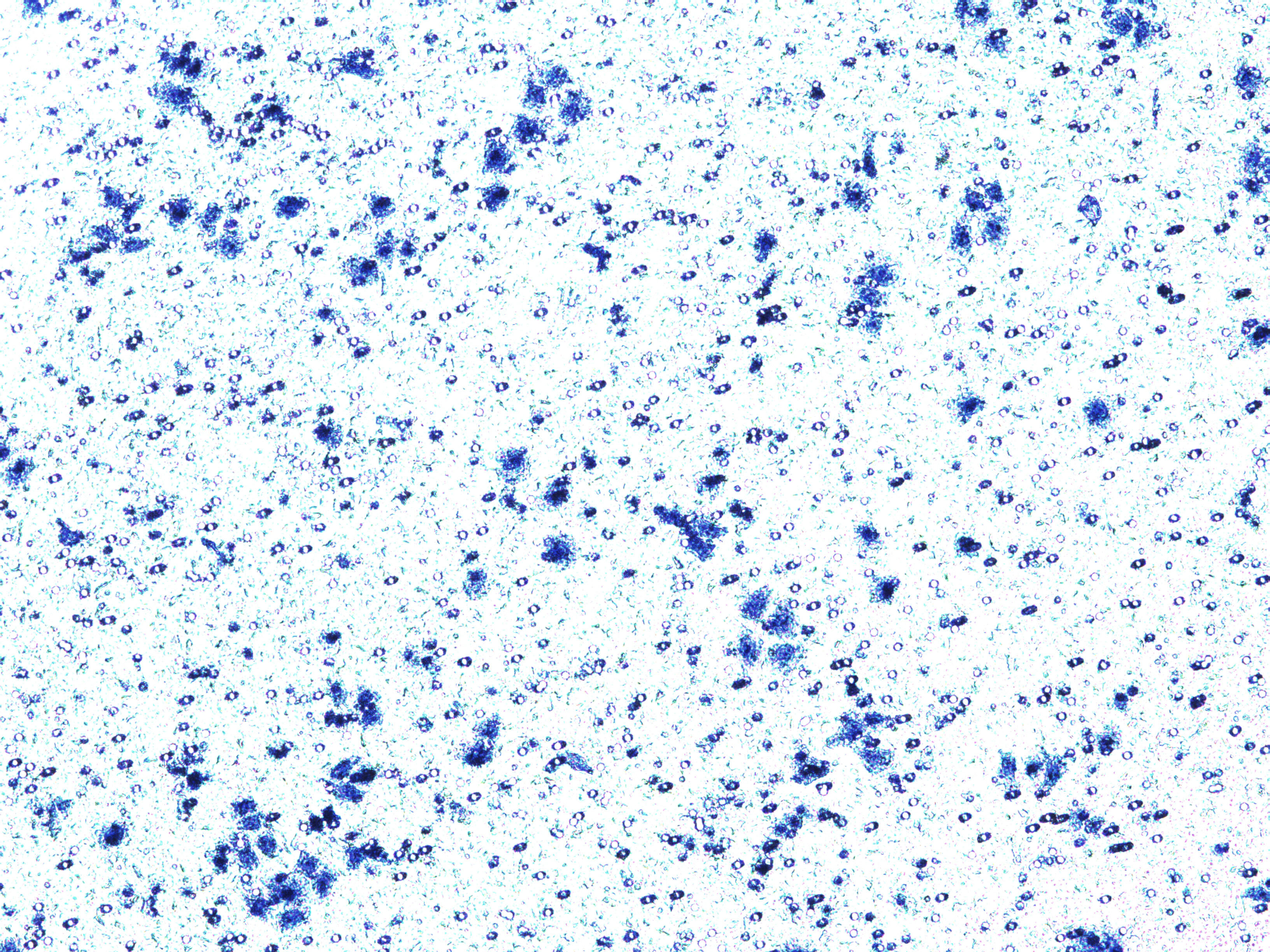

Supplement: S8 File — (ZIP) [file pone.0334639.s008.zip › S 13. File. Original Images. Fig6/S 13. File. Original FIgures. Fig.6/6f/HEPG2/N/hepG2 LX2 20%.jpg]

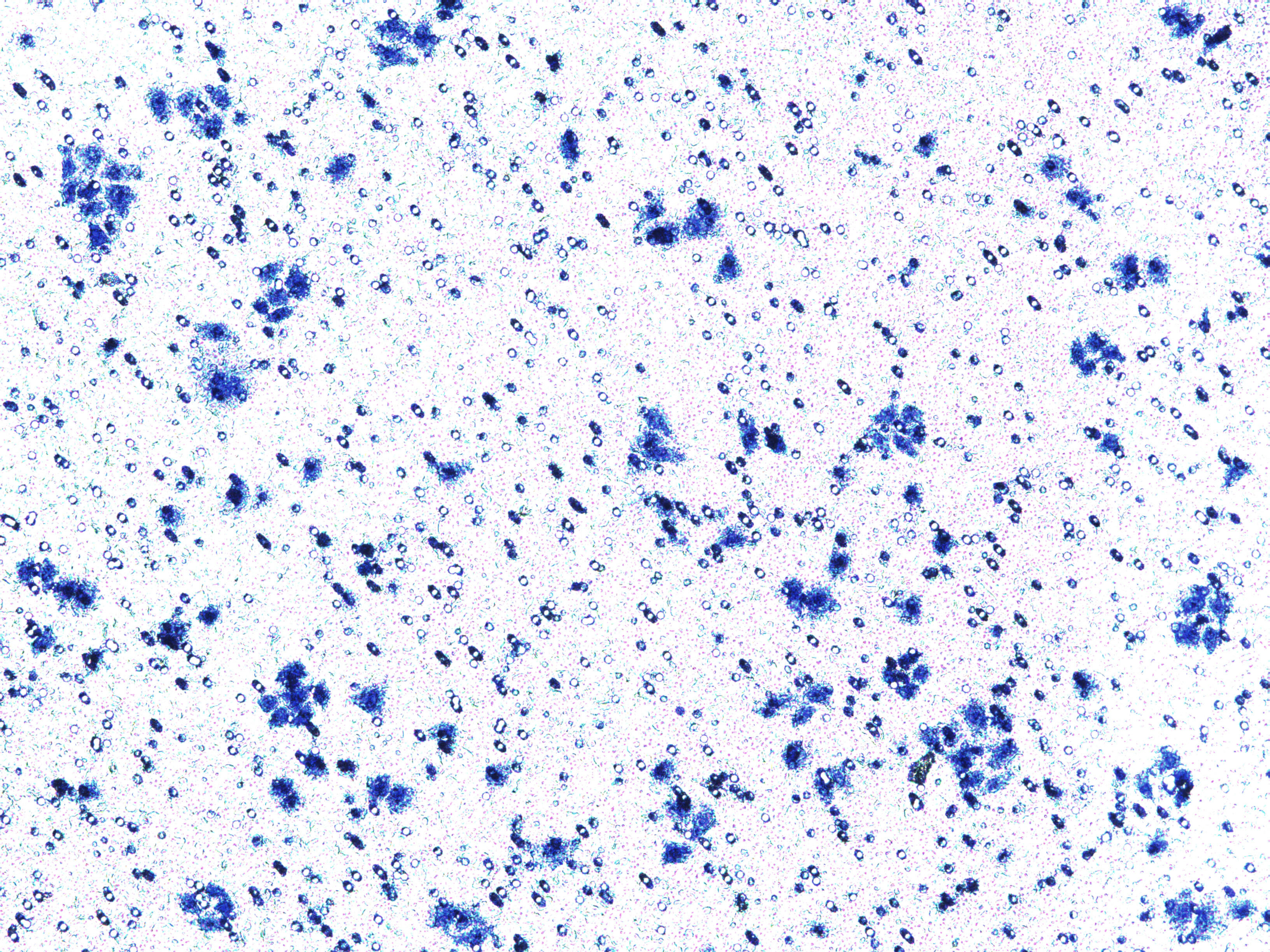

Supplement: S8 File — (ZIP) [file pone.0334639.s008.zip › S 13. File. Original Images. Fig6/S 13. File. Original FIgures. Fig.6/6f/HEPG2/N/hepG2 LX2 40%.jpg]

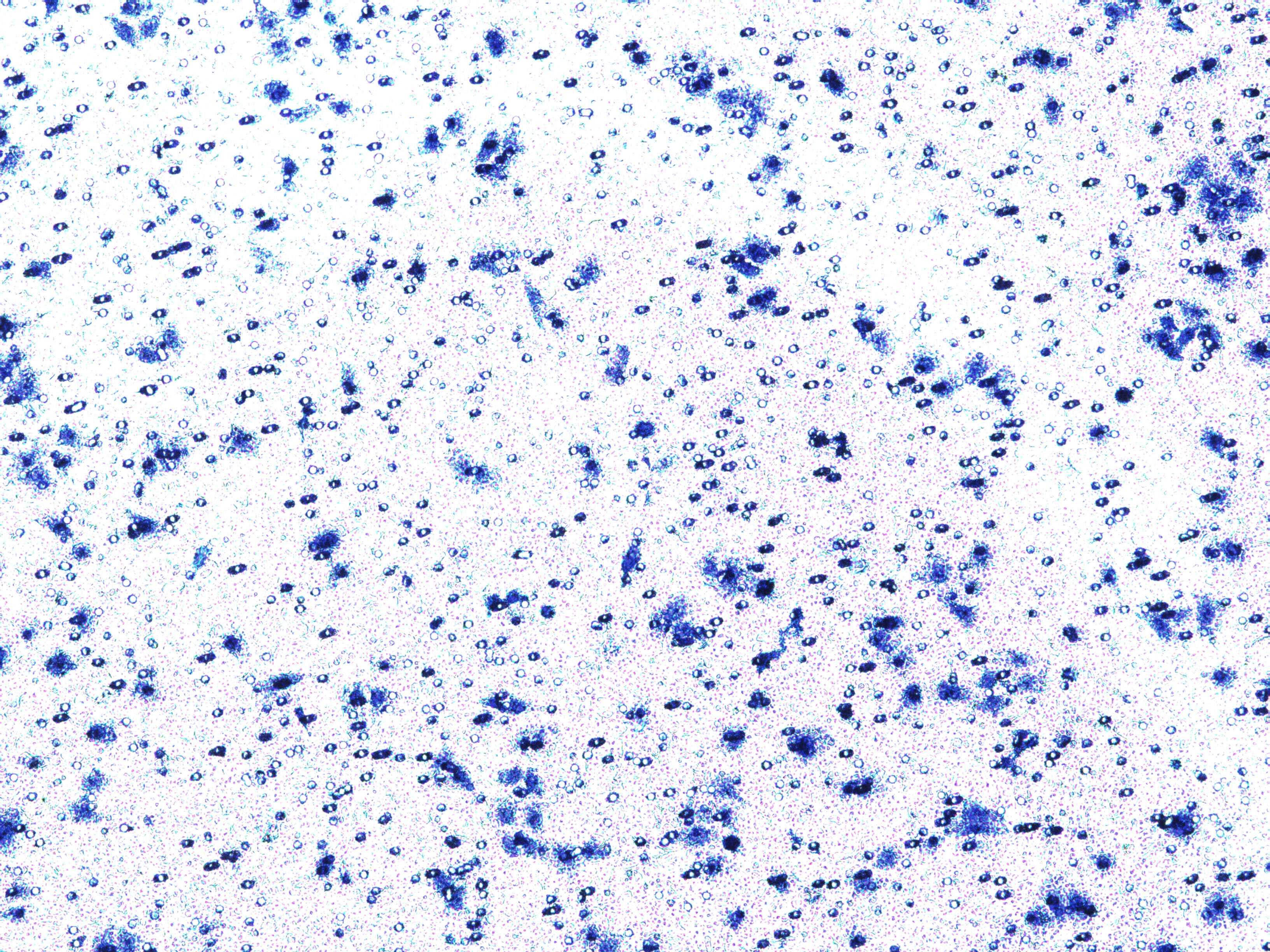

Supplement: S8 File — (ZIP) [file pone.0334639.s008.zip › S 13. File. Original Images. Fig6/S 13. File. Original FIgures. Fig.6/6f/HEPG2/N/hepG2 LX2 60%.jpg]

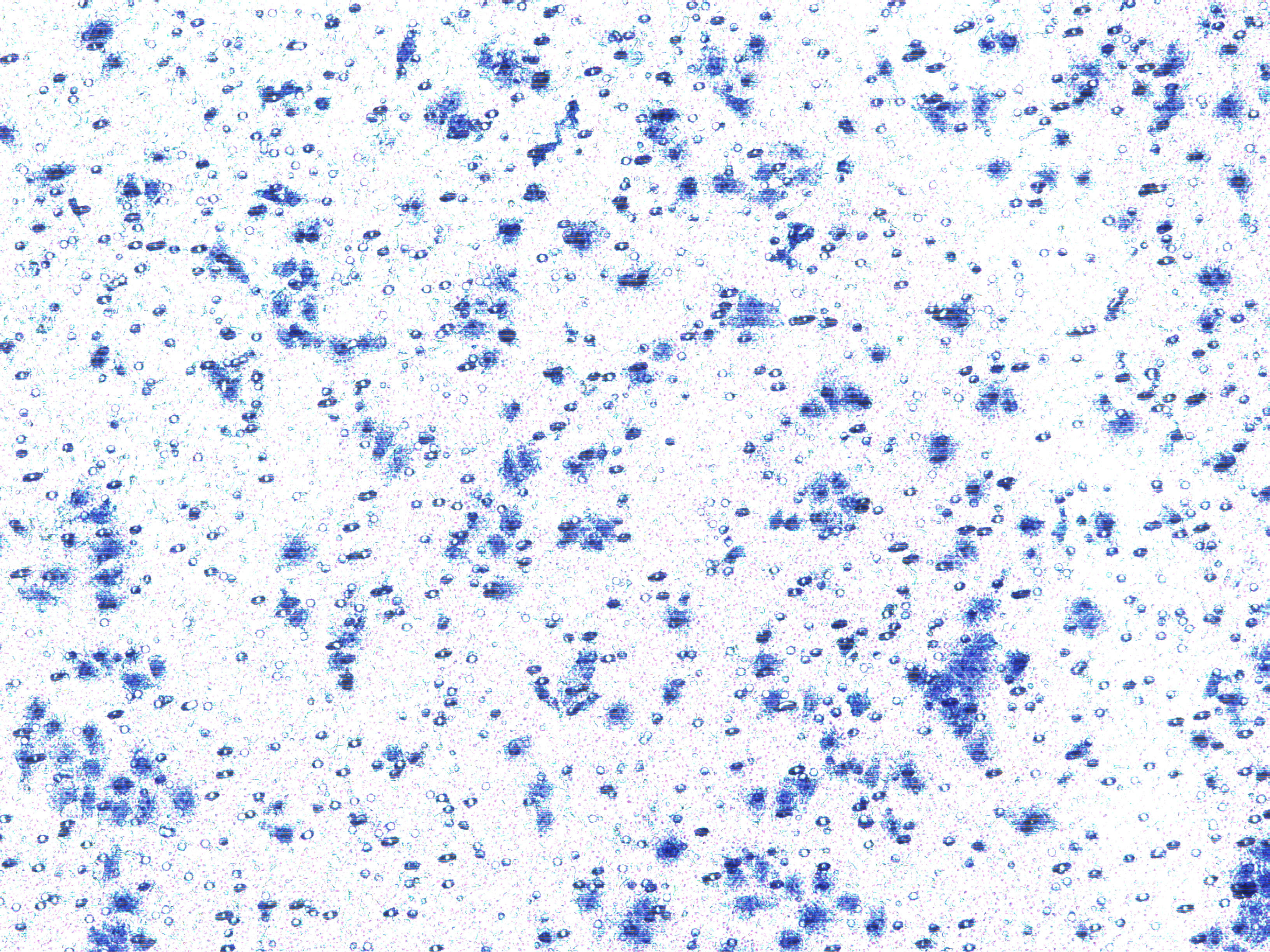

Supplement: S8 File — (ZIP) [file pone.0334639.s008.zip › S 13. File. Original Images. Fig6/S 13. File. Original FIgures. Fig.6/6f/HEPG2/N/hepG2 LX2 80%.jpg]

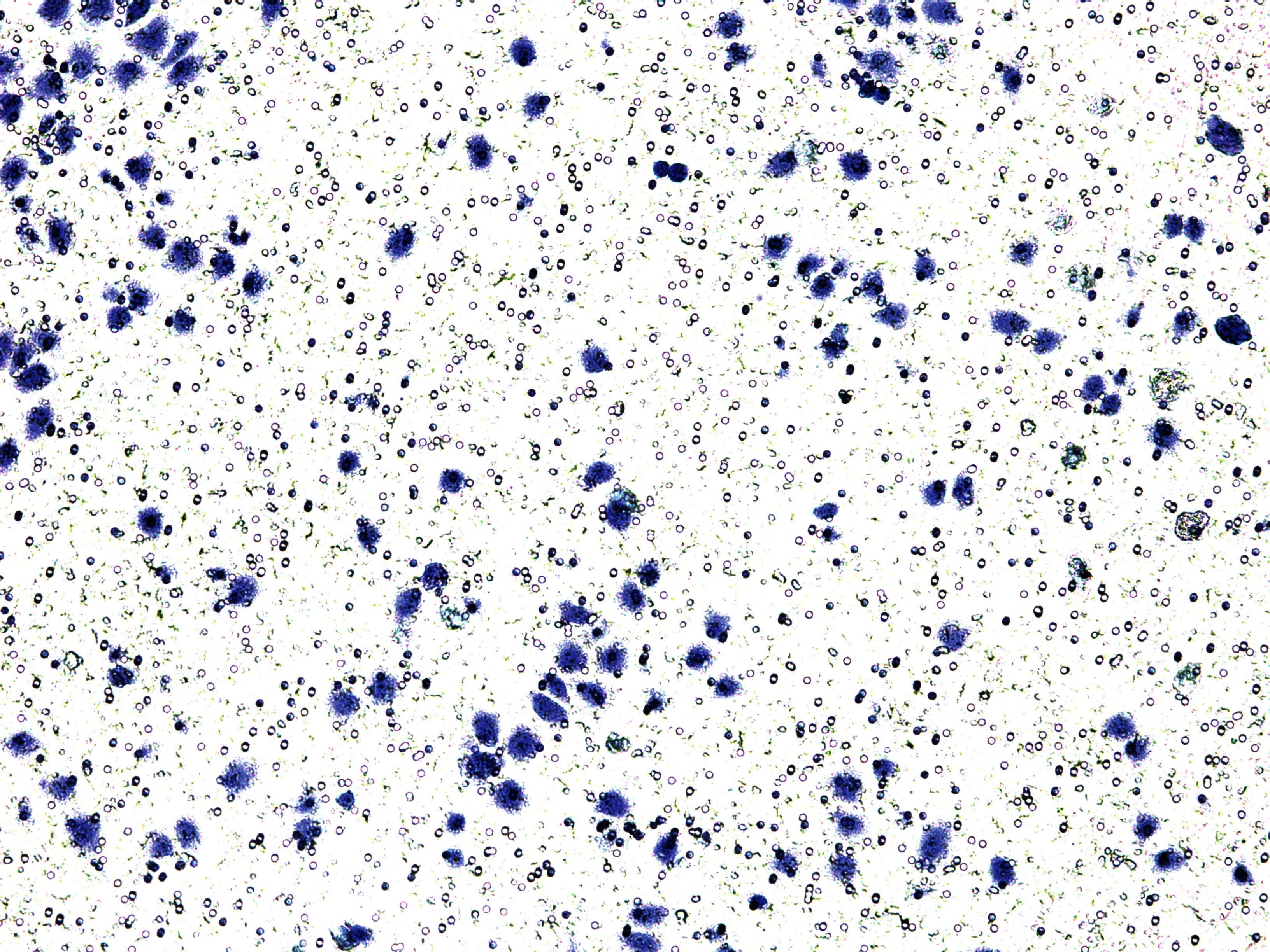

Supplement: S8 File — (ZIP) [file pone.0334639.s008.zip › S 13. File. Original Images. Fig6/S 13. File. Original FIgures. Fig.6/6g/SMMC-7721/A/smmc-7721 lx2 0%.jpg]

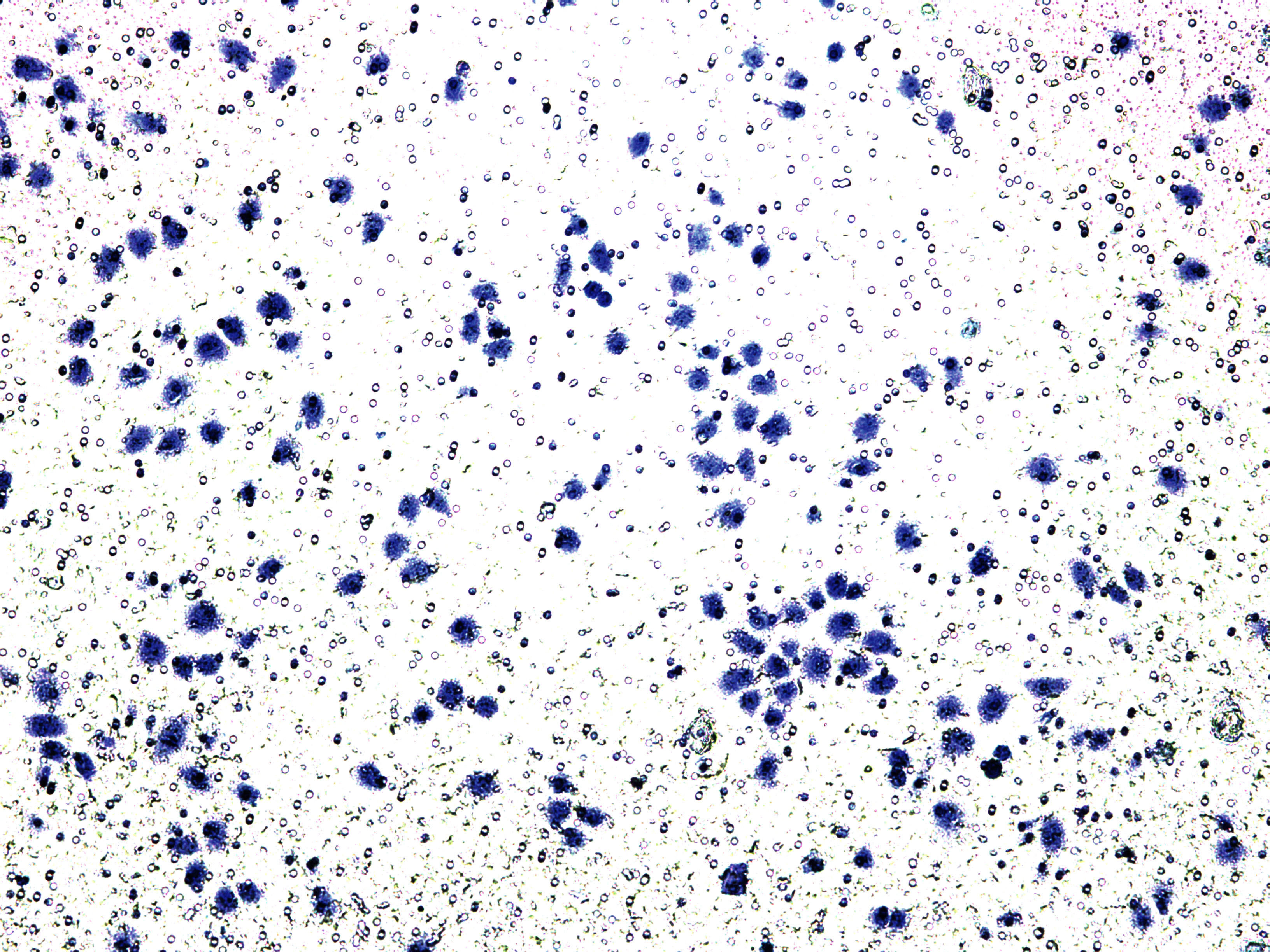

Supplement: S8 File — (ZIP) [file pone.0334639.s008.zip › S 13. File. Original Images. Fig6/S 13. File. Original FIgures. Fig.6/6g/SMMC-7721/A/smmc-7721 lx2 20%.jpg]

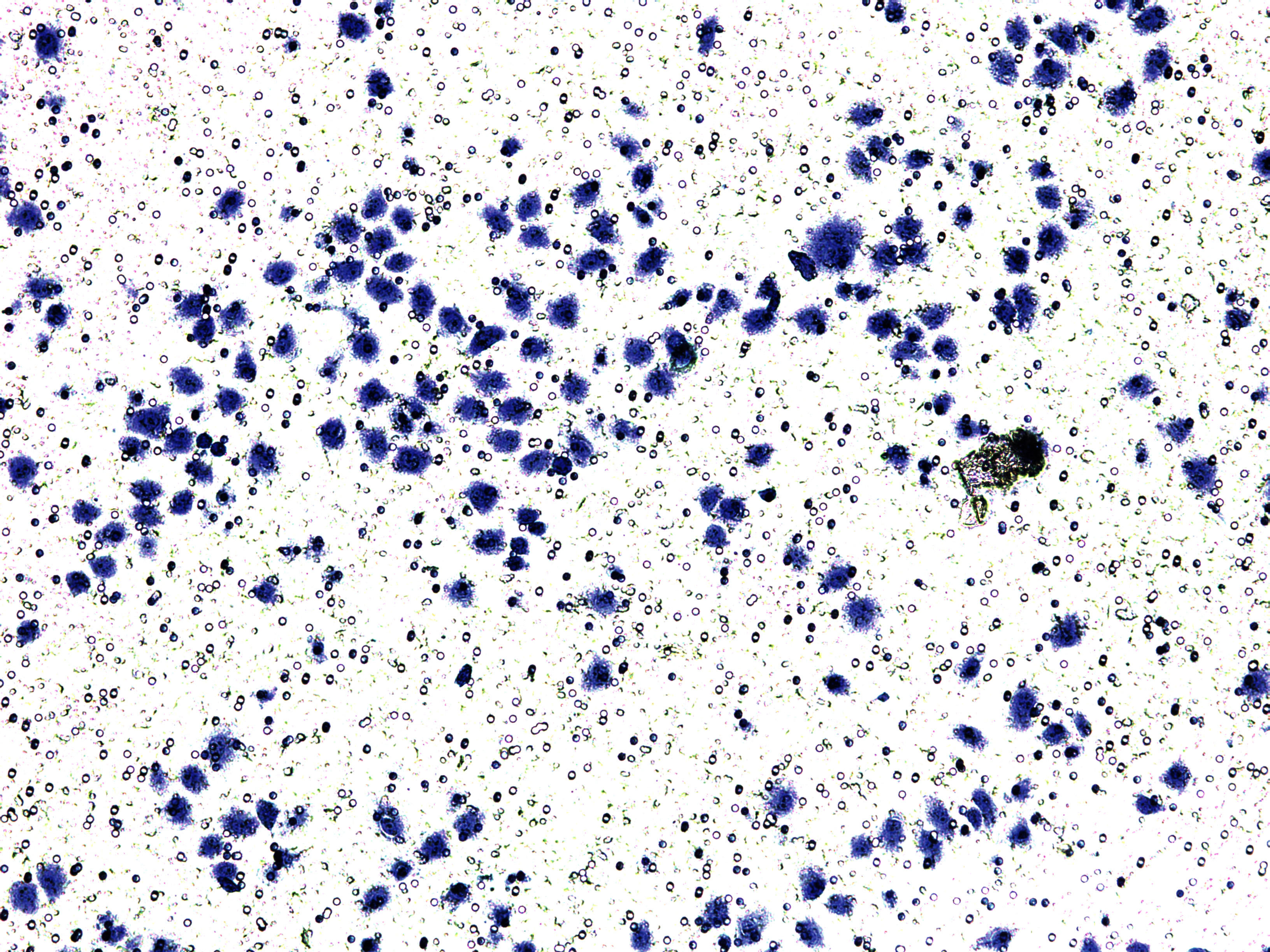

Supplement: S8 File — (ZIP) [file pone.0334639.s008.zip › S 13. File. Original Images. Fig6/S 13. File. Original FIgures. Fig.6/6g/SMMC-7721/A/smmc-7721 lx2 40%.jpg]

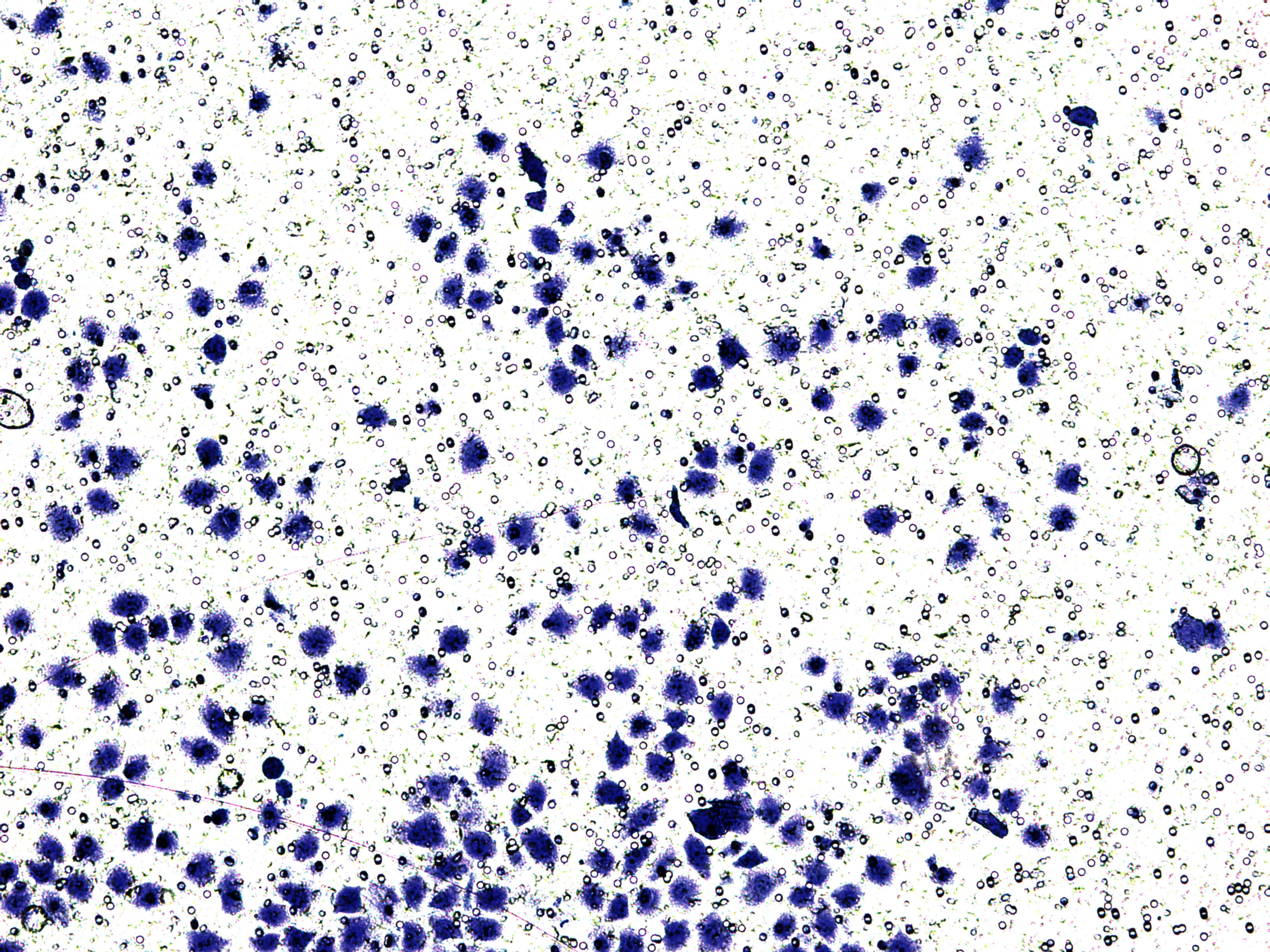

Supplement: S8 File — (ZIP) [file pone.0334639.s008.zip › S 13. File. Original Images. Fig6/S 13. File. Original FIgures. Fig.6/6g/SMMC-7721/A/smmc-7721 lx2 A60%.jpg]

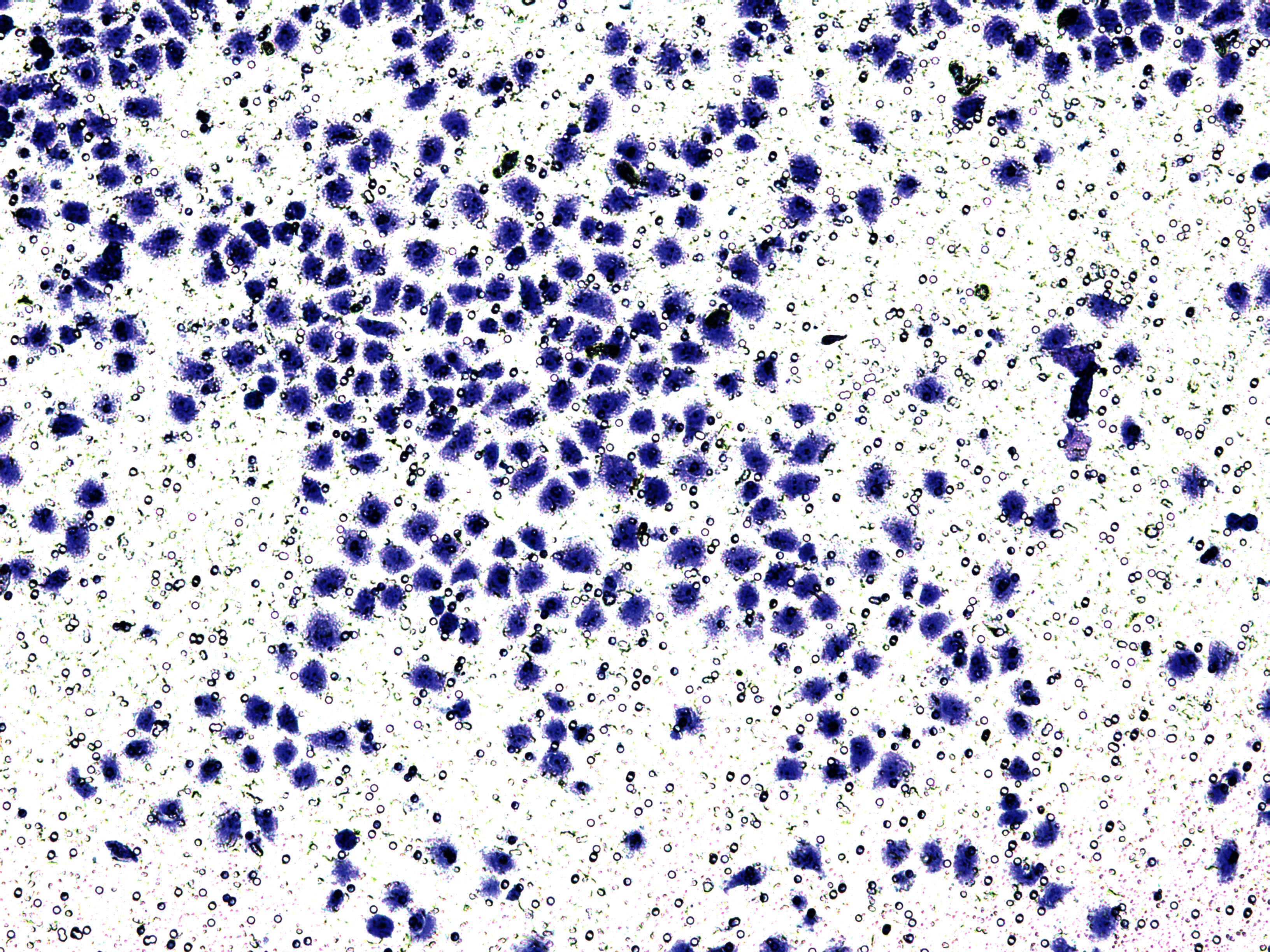

Supplement: S8 File — (ZIP) [file pone.0334639.s008.zip › S 13. File. Original Images. Fig6/S 13. File. Original FIgures. Fig.6/6g/SMMC-7721/A/smmc-7721 lx2 A80%.jpg]

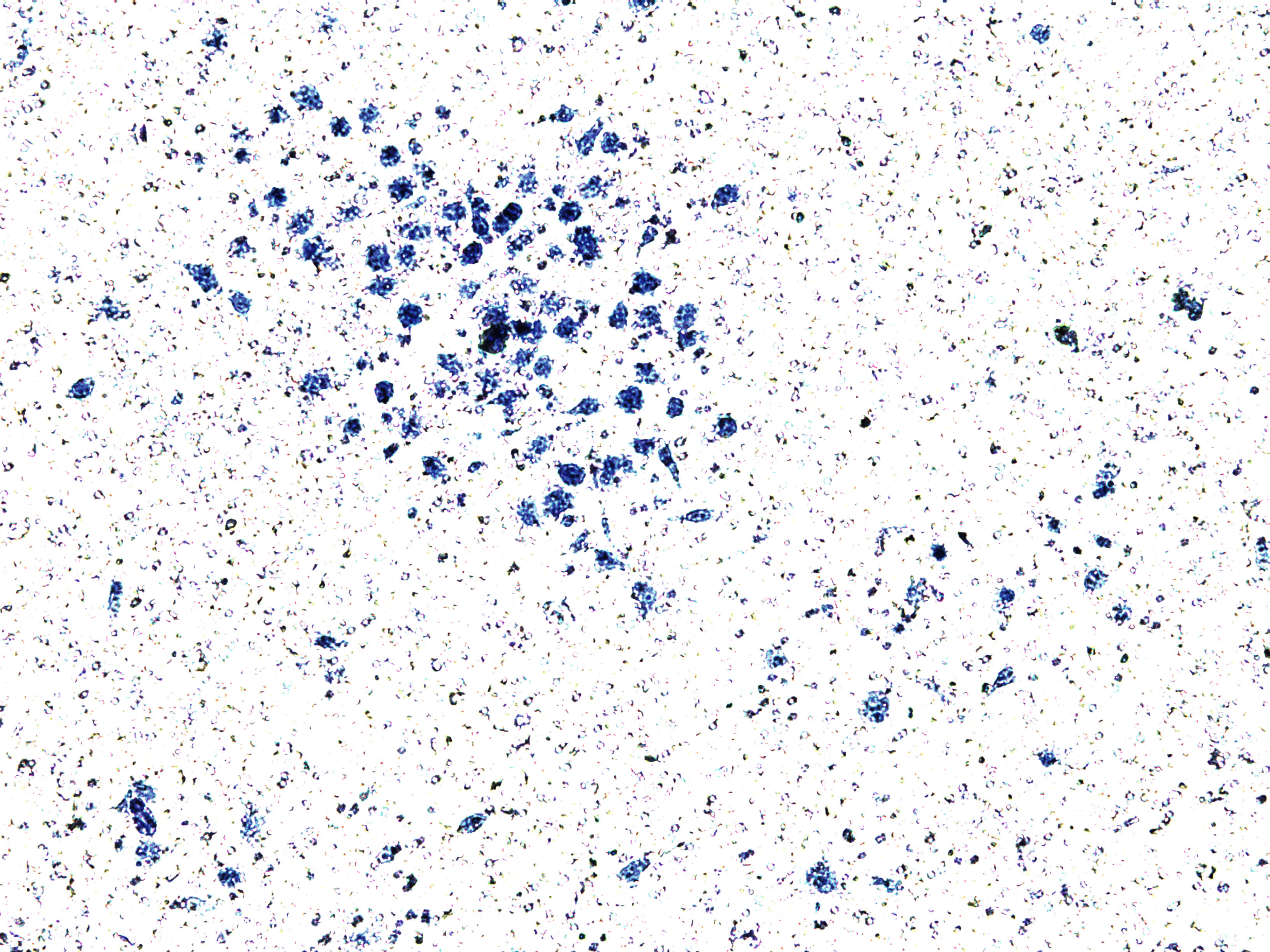

Supplement: S8 File — (ZIP) [file pone.0334639.s008.zip › S 13. File. Original Images. Fig6/S 13. File. Original FIgures. Fig.6/6g/SMMC-7721/N/smmc-7721 lx2 0%.jpg]

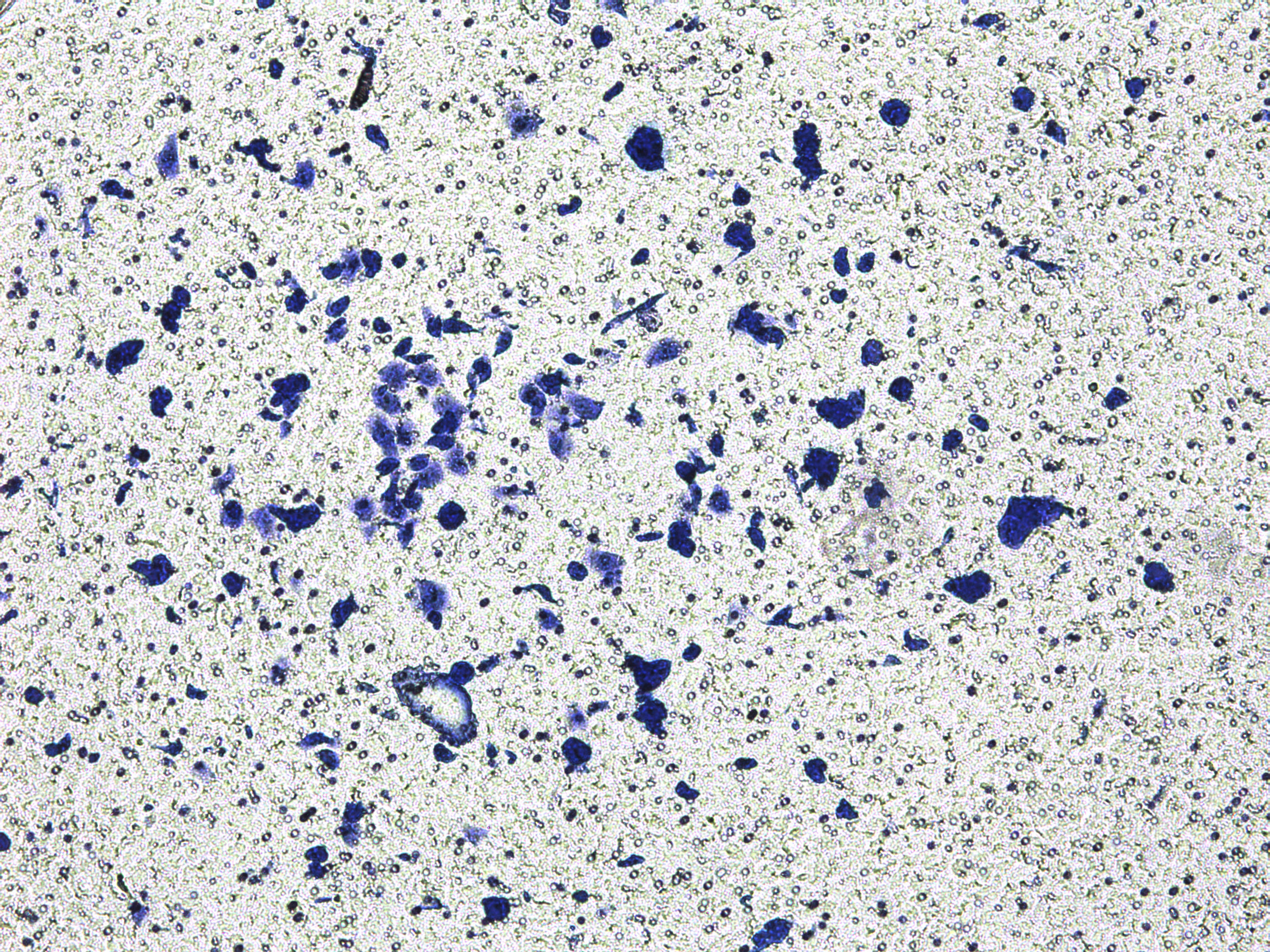

Supplement: S8 File — (ZIP) [file pone.0334639.s008.zip › S 13. File. Original Images. Fig6/S 13. File. Original FIgures. Fig.6/6g/SMMC-7721/N/smmc-7721 lx2 20%.jpg]

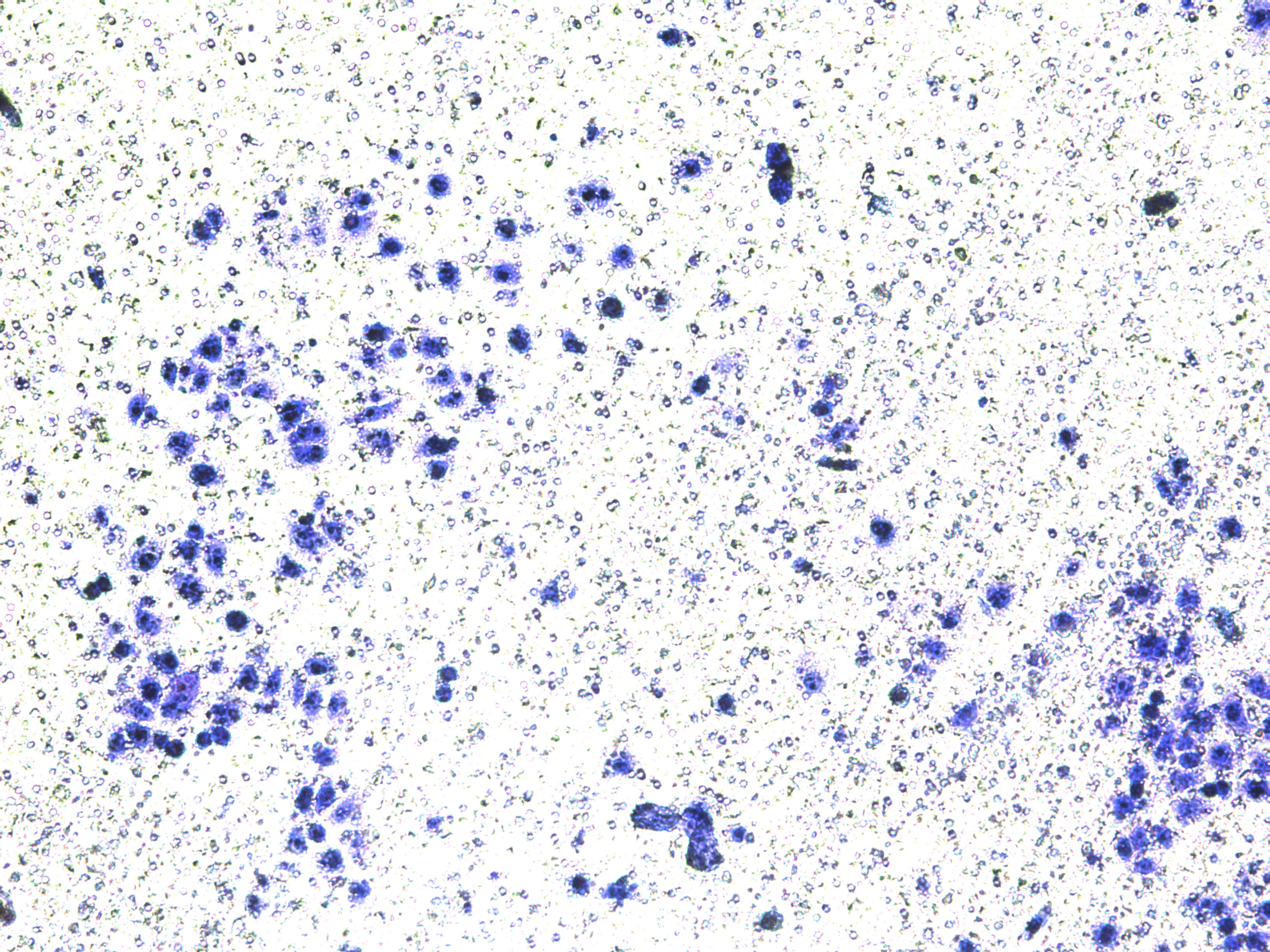

Supplement: S8 File — (ZIP) [file pone.0334639.s008.zip › S 13. File. Original Images. Fig6/S 13. File. Original FIgures. Fig.6/6g/SMMC-7721/N/smmc-7721 lx2 40%.jpg]

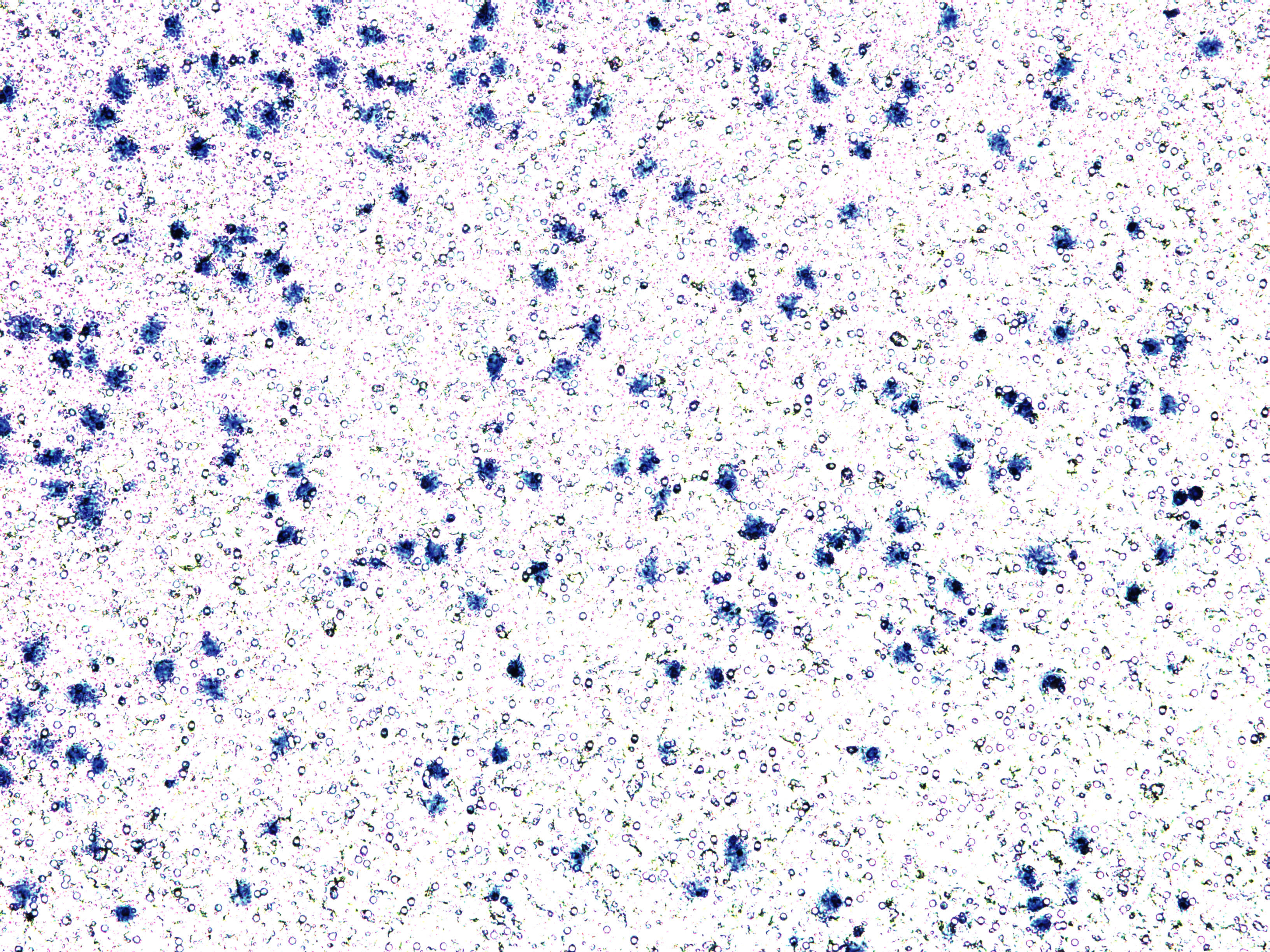

Supplement: S8 File — (ZIP) [file pone.0334639.s008.zip › S 13. File. Original Images. Fig6/S 13. File. Original FIgures. Fig.6/6g/SMMC-7721/N/smmc-7721 lx2 60%.jpg]

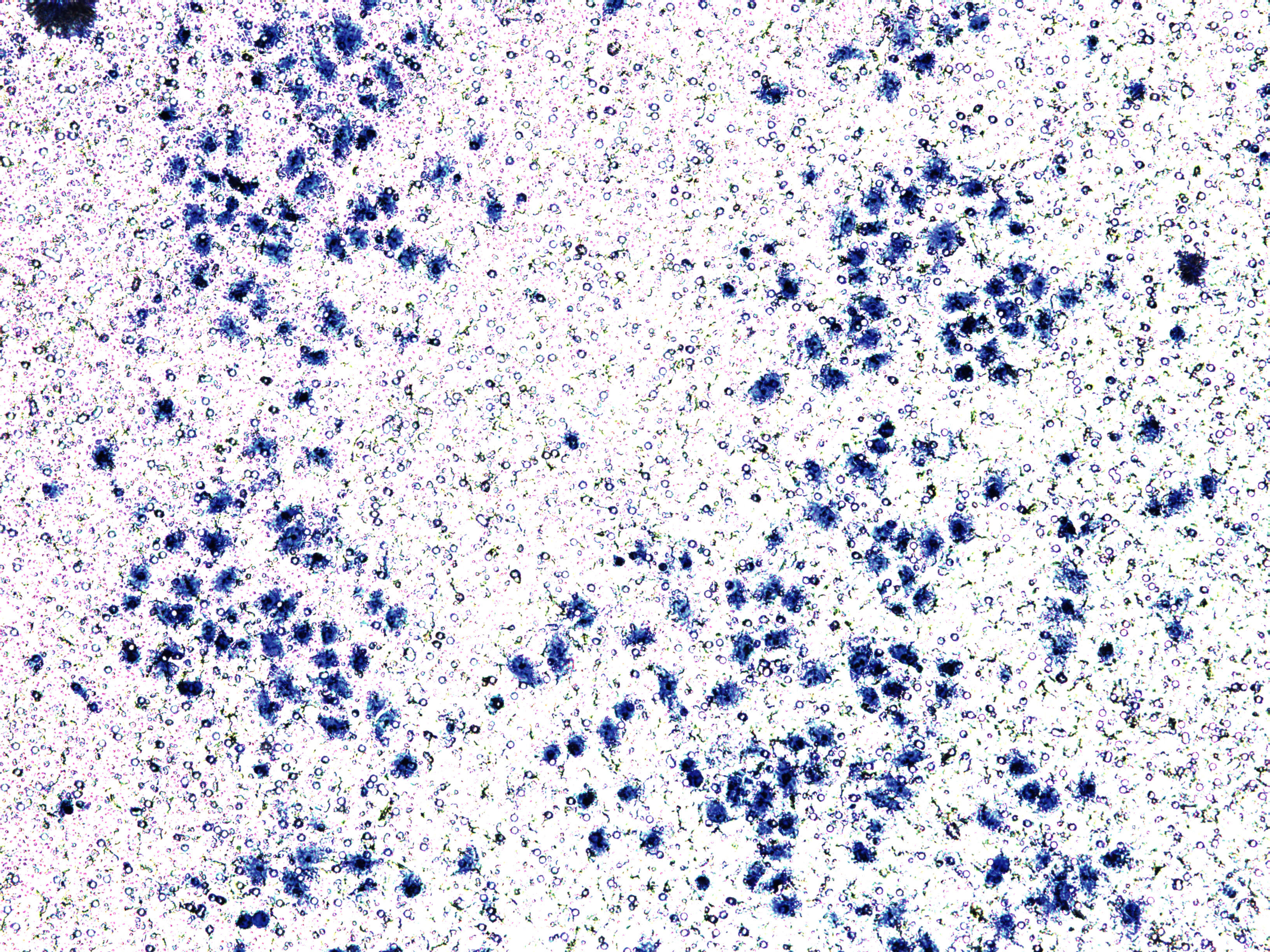

Supplement: S8 File — (ZIP) [file pone.0334639.s008.zip › S 13. File. Original Images. Fig6/S 13. File. Original FIgures. Fig.6/6g/SMMC-7721/N/smmc-7721 lx2 80%.jpg]

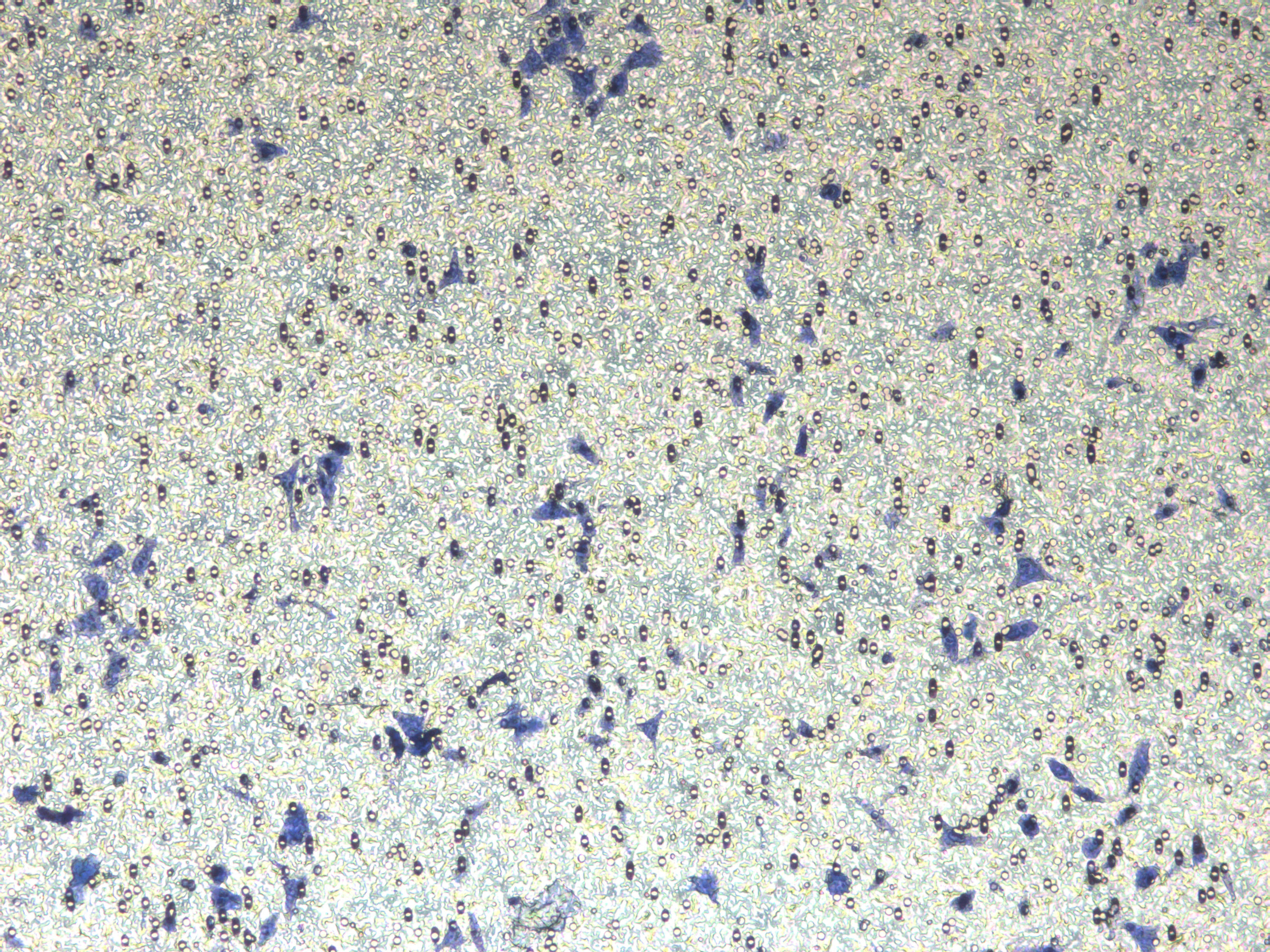

Supplement: S9 File — (ZIP) [file pone.0334639.s009.zip › S 14. File. Original Images. Fig7/S 14. File. Original FIgures. Fig.7/7d/BEL-7402/DMSO/bel cxcl3 0ngml.jpg]
