# Supplementary figures and images for: CXCL3 promotes liver cancer progression by modulating the tumor microenvironment via the PI3K/AKT/mTOR pathway (part 3 of 3)
Source: PLoS One. 2025 Nov 19;20(11):e0334639. doi: 10.1371/journal.pone.0334639 (PMC12629499; doi:10.1371/journal.pone.0334639)

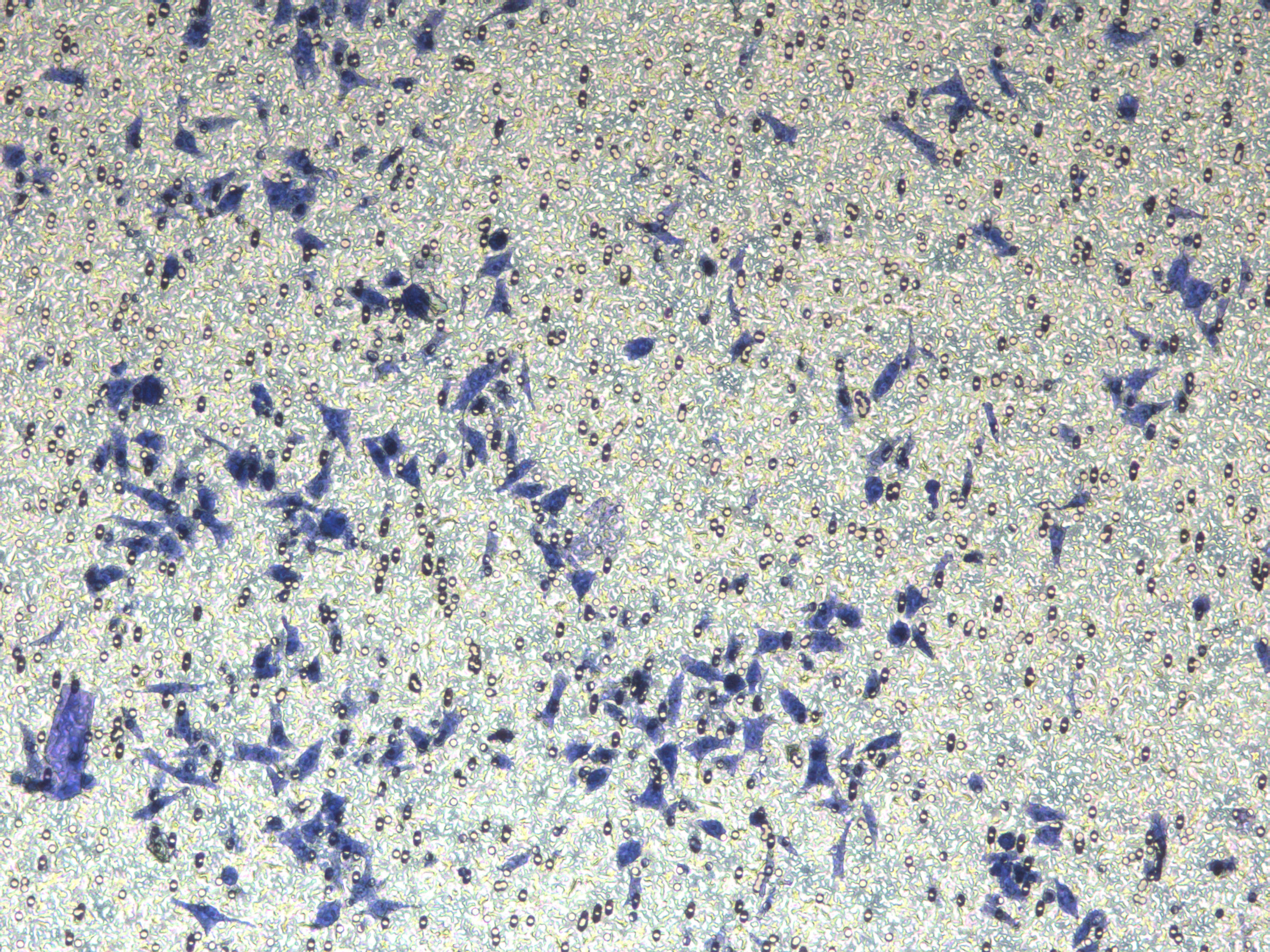

Supplement: S9 File — (ZIP) [file pone.0334639.s009.zip › S 14. File. Original Images. Fig7/S 14. File. Original FIgures. Fig.7/7d/BEL-7402/DMSO/bel cxcl3 10ngml.jpg]

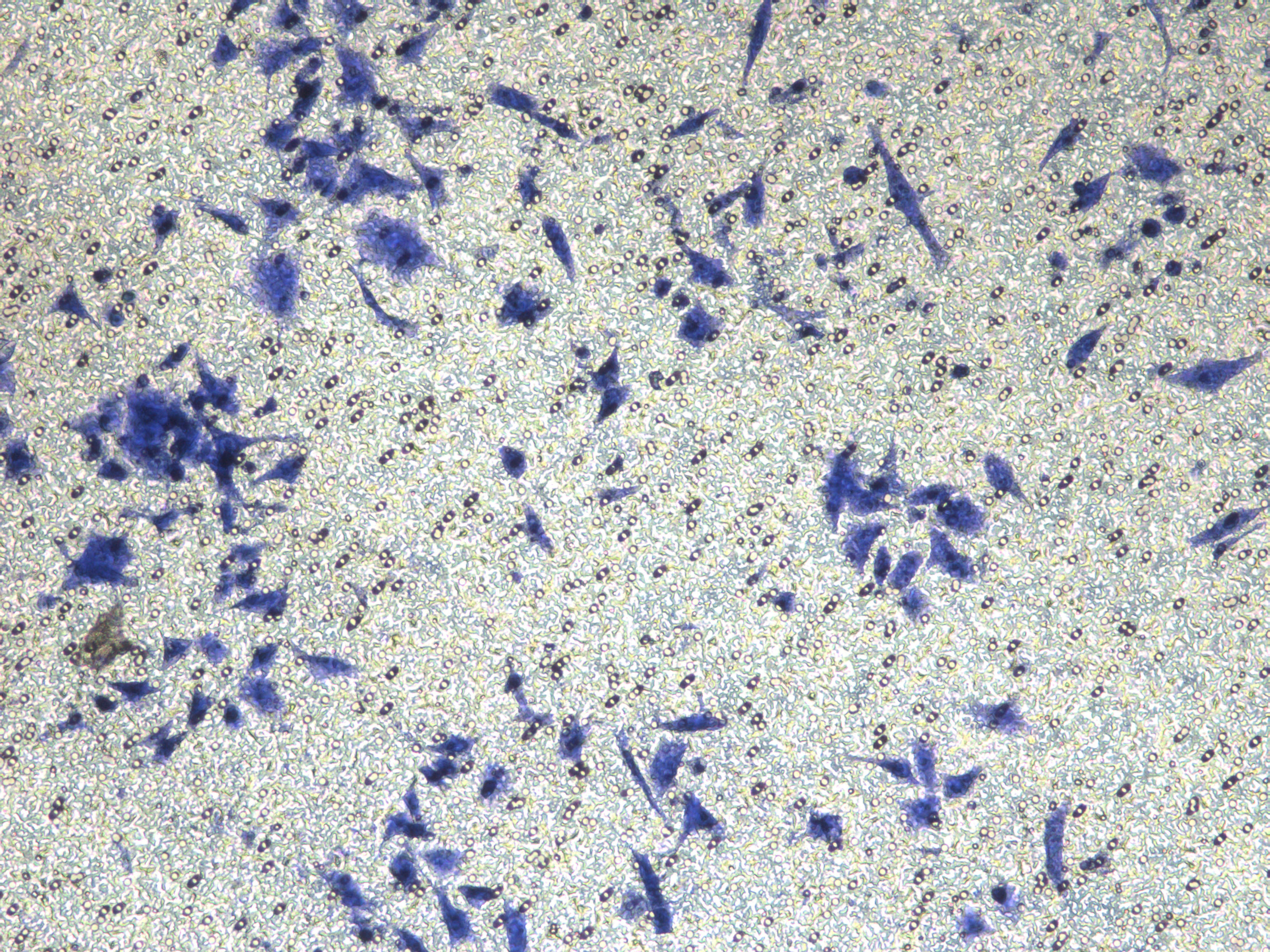

Supplement: S9 File — (ZIP) [file pone.0334639.s009.zip › S 14. File. Original Images. Fig7/S 14. File. Original FIgures. Fig.7/7d/BEL-7402/DMSO/bel cxcl3 2ngml.jpg]

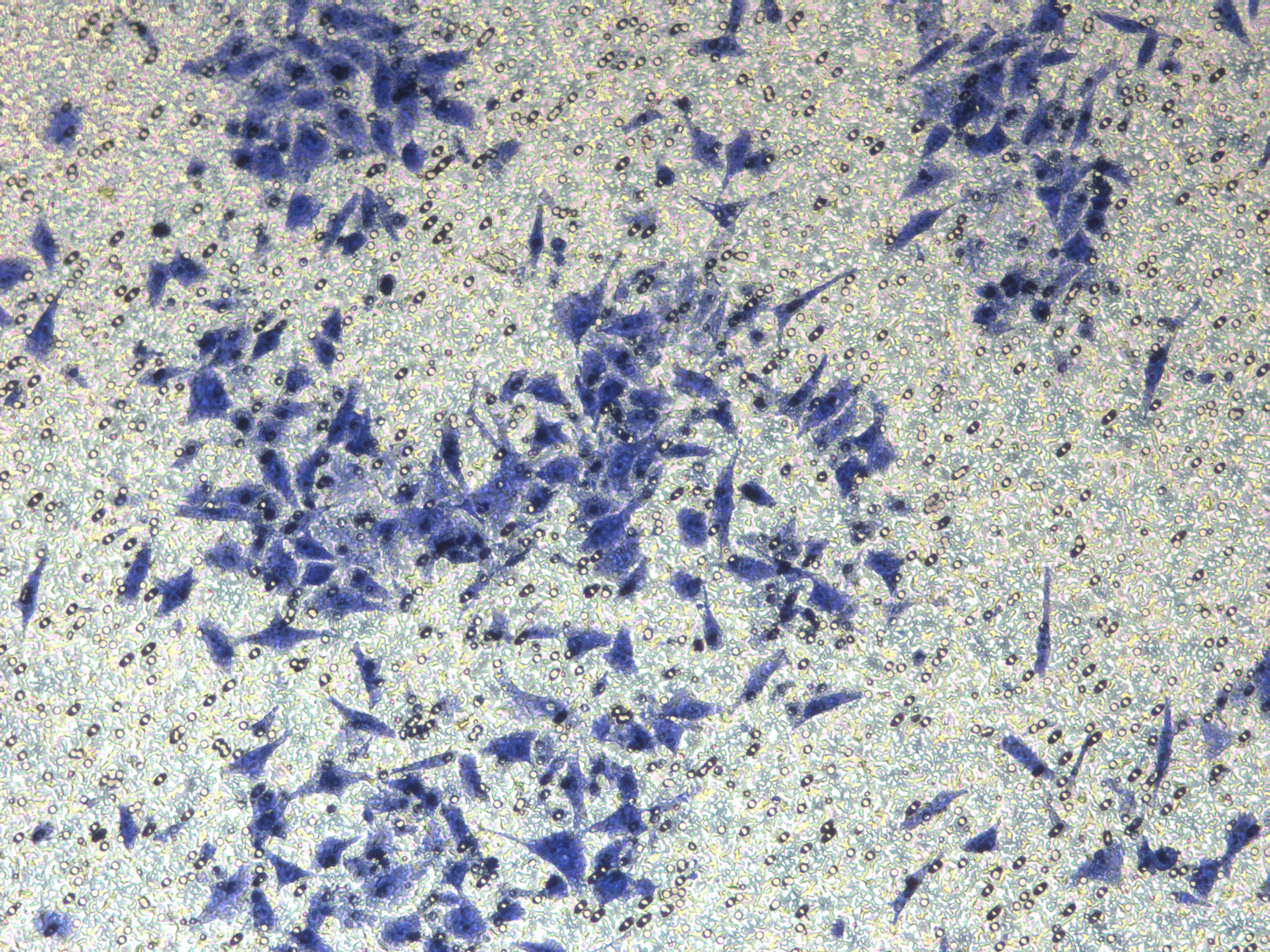

Supplement: S9 File — (ZIP) [file pone.0334639.s009.zip › S 14. File. Original Images. Fig7/S 14. File. Original FIgures. Fig.7/7d/BEL-7402/DMSO/bel cxcl3 5ngml.jpg]

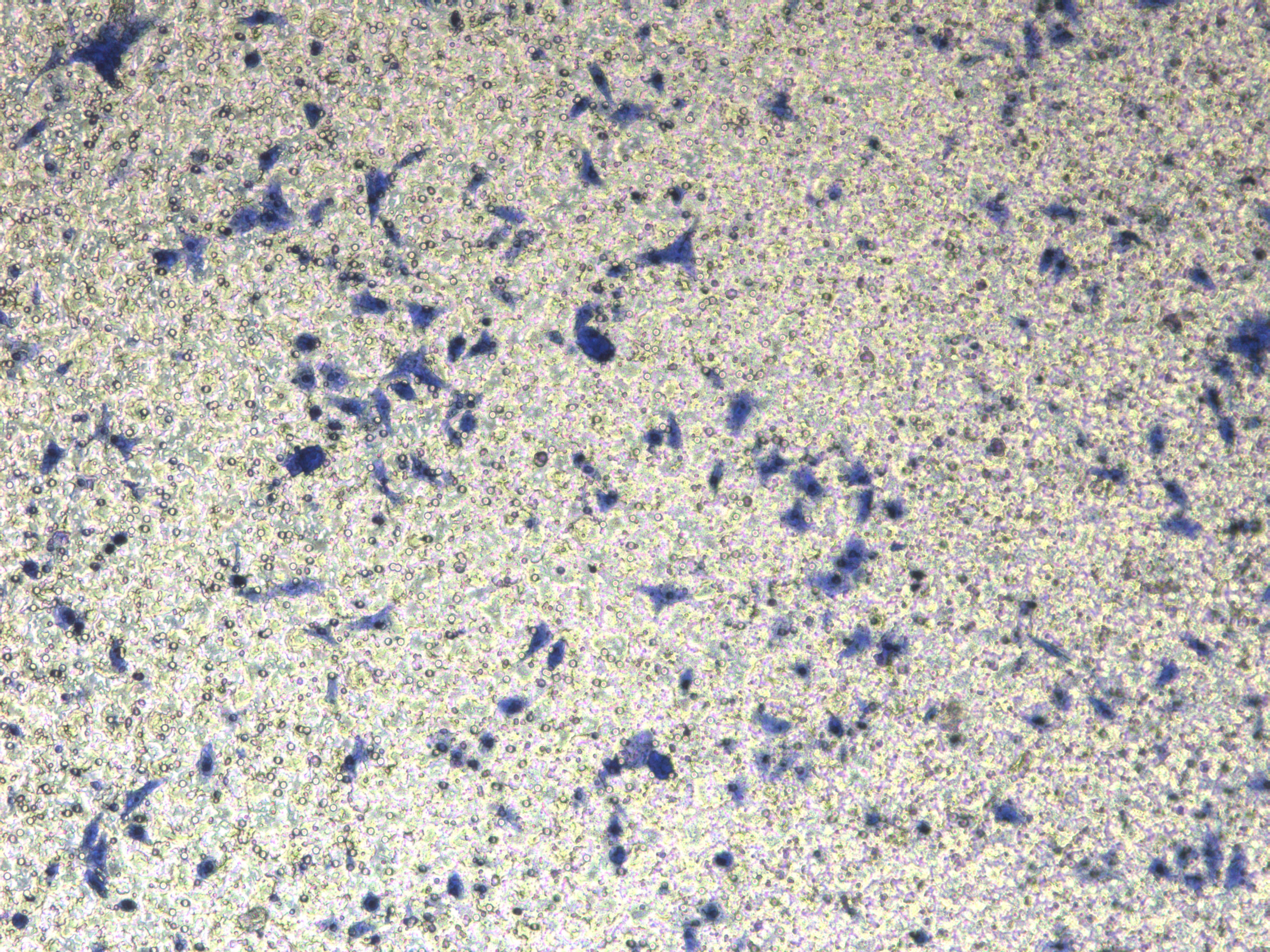

Supplement: S9 File — (ZIP) [file pone.0334639.s009.zip › S 14. File. Original Images. Fig7/S 14. File. Original FIgures. Fig.7/7d/BEL-7402/DMSO/bel cxcl3 20ngml.jpg]

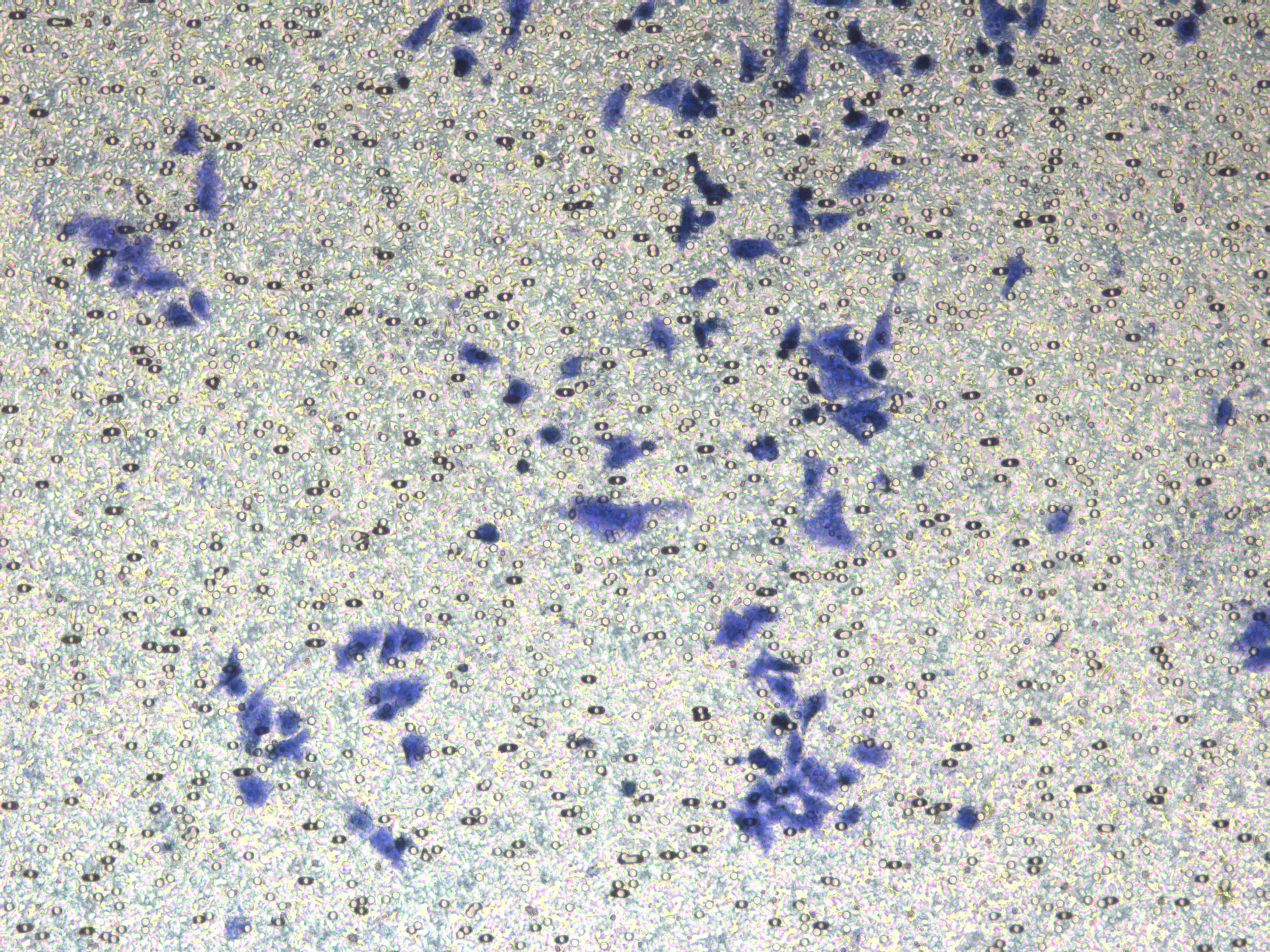

Supplement: S9 File — (ZIP) [file pone.0334639.s009.zip › S 14. File. Original Images. Fig7/S 14. File. Original FIgures. Fig.7/7d/BEL-7402/DMSO/bel cxcl3 30ngml.jpg]

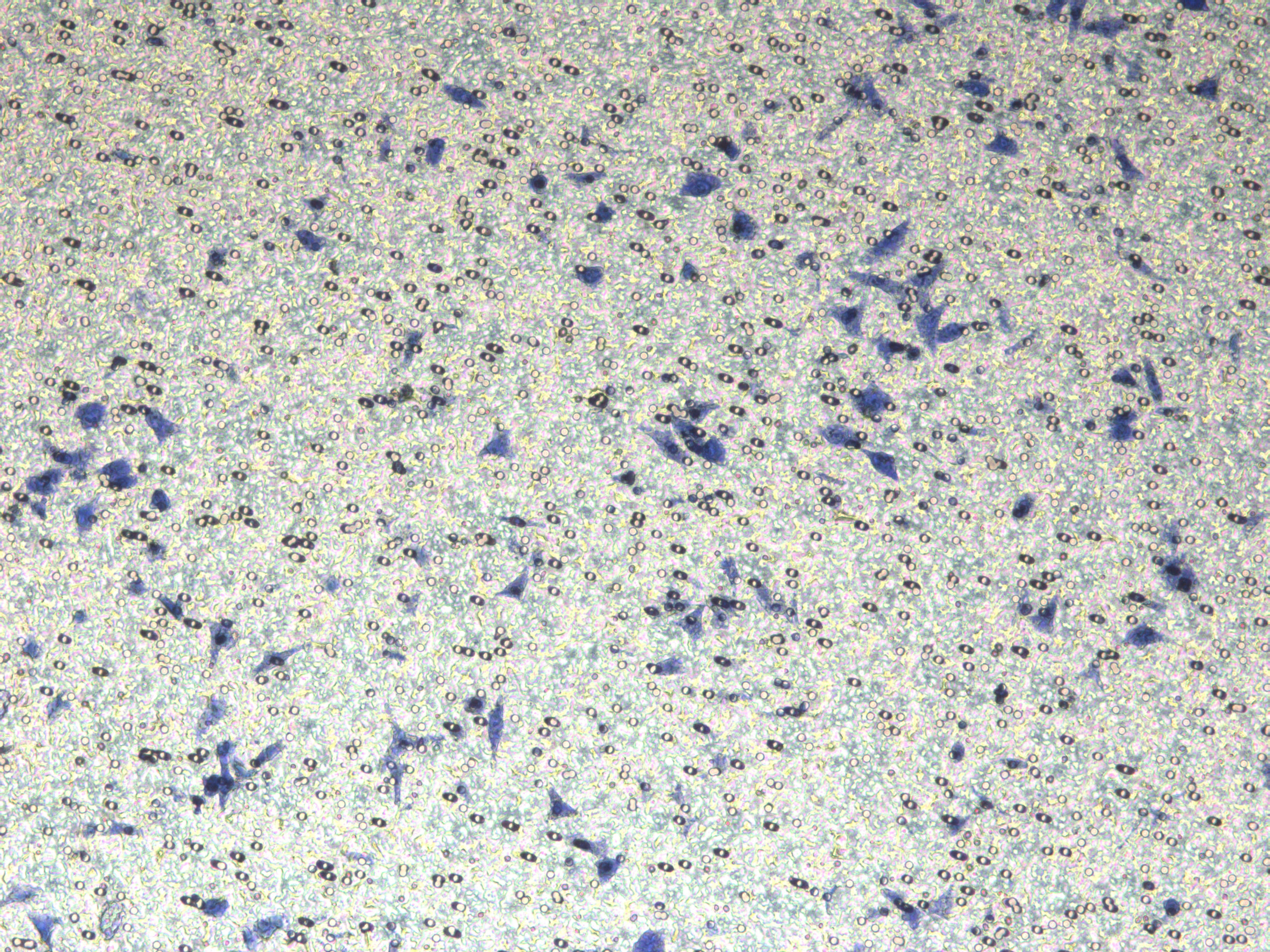

Supplement: S9 File — (ZIP) [file pone.0334639.s009.zip › S 14. File. Original Images. Fig7/S 14. File. Original FIgures. Fig.7/7d/BEL-7402/mtor/bel cxcl3 20ngml.jpg]

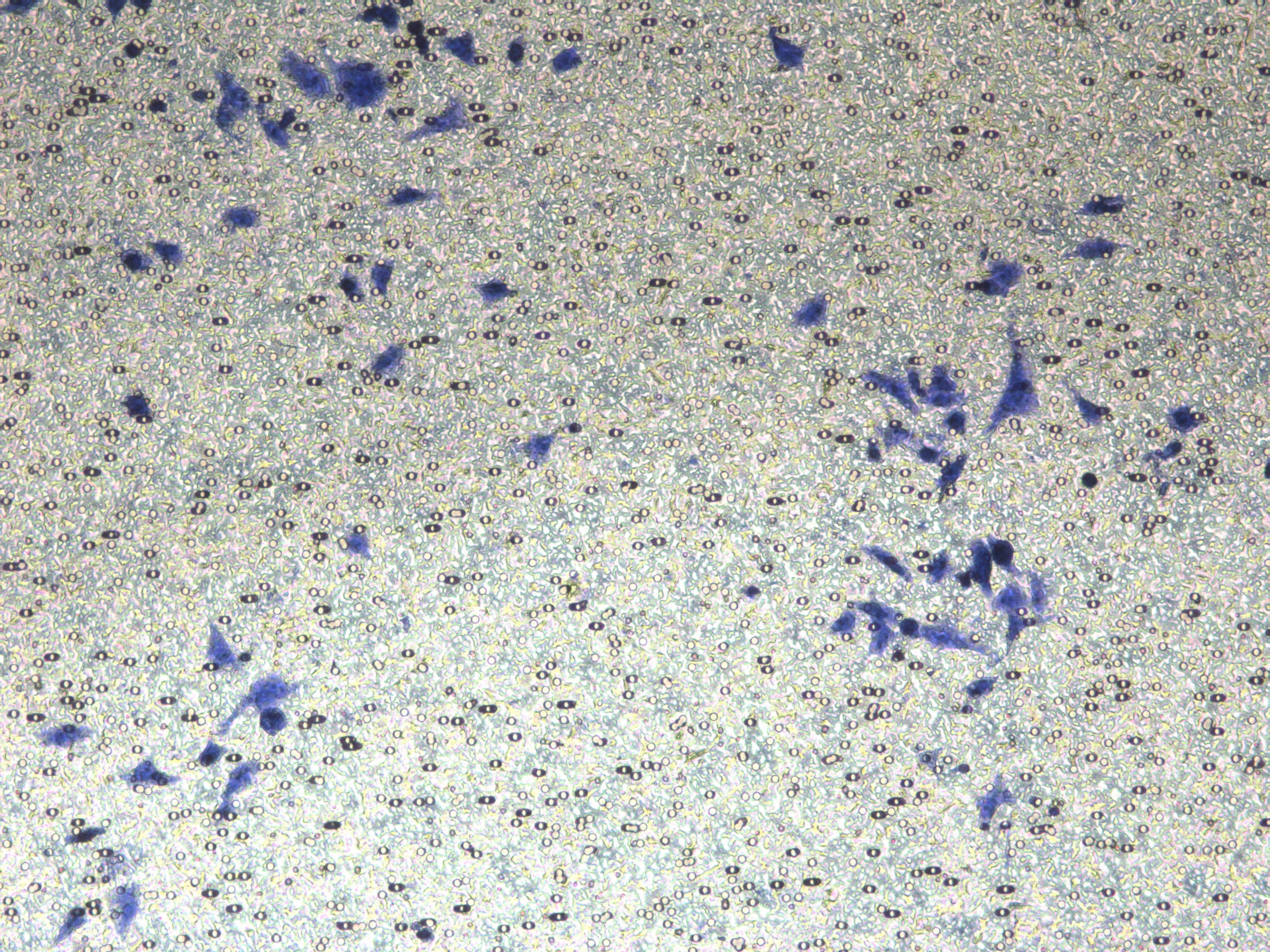

Supplement: S9 File — (ZIP) [file pone.0334639.s009.zip › S 14. File. Original Images. Fig7/S 14. File. Original FIgures. Fig.7/7d/BEL-7402/mtor/bel cxcl3 0ngml.jpg]

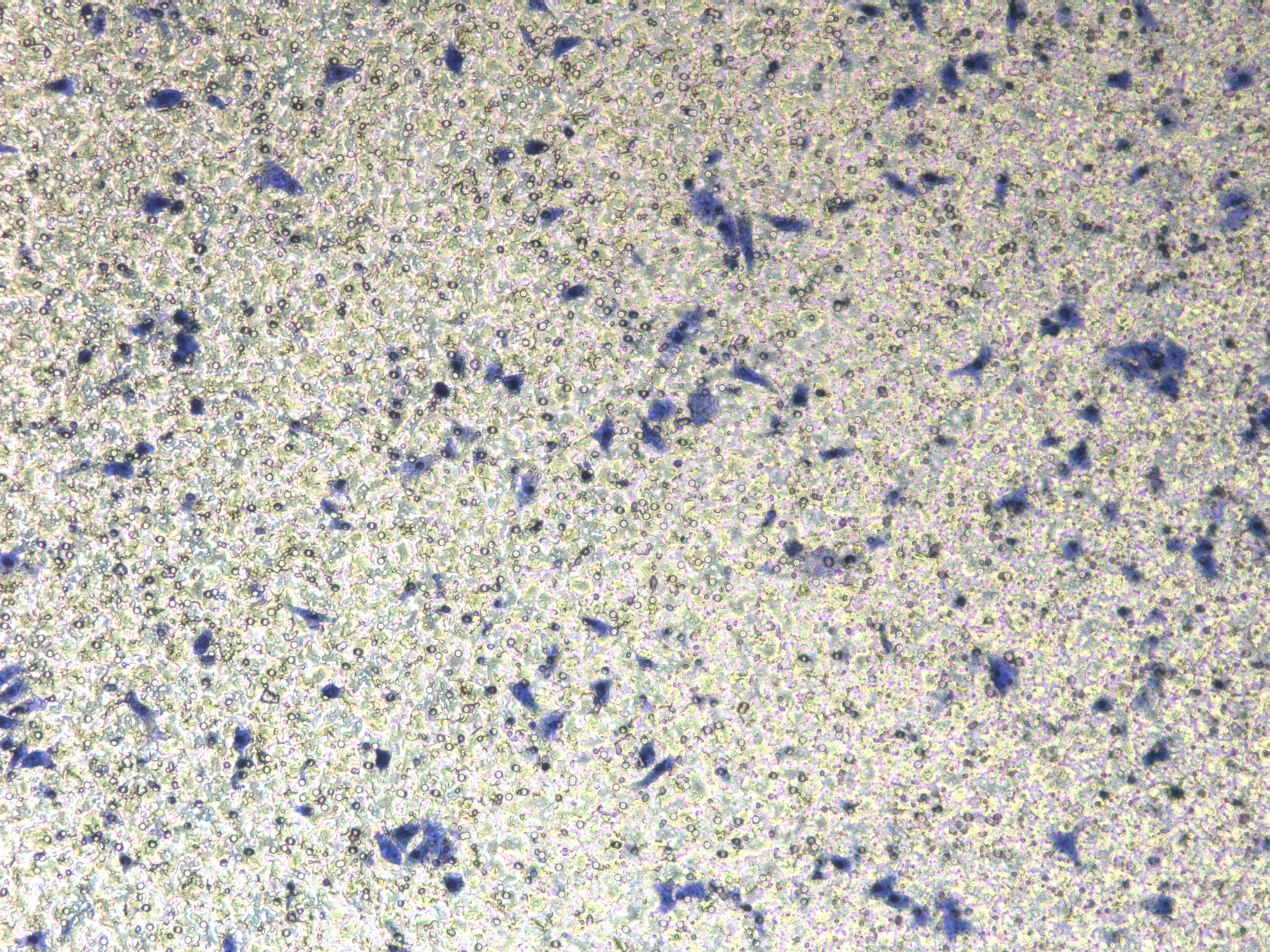

Supplement: S9 File — (ZIP) [file pone.0334639.s009.zip › S 14. File. Original Images. Fig7/S 14. File. Original FIgures. Fig.7/7d/BEL-7402/mtor/bel cxcl3 10ngml.jpg]

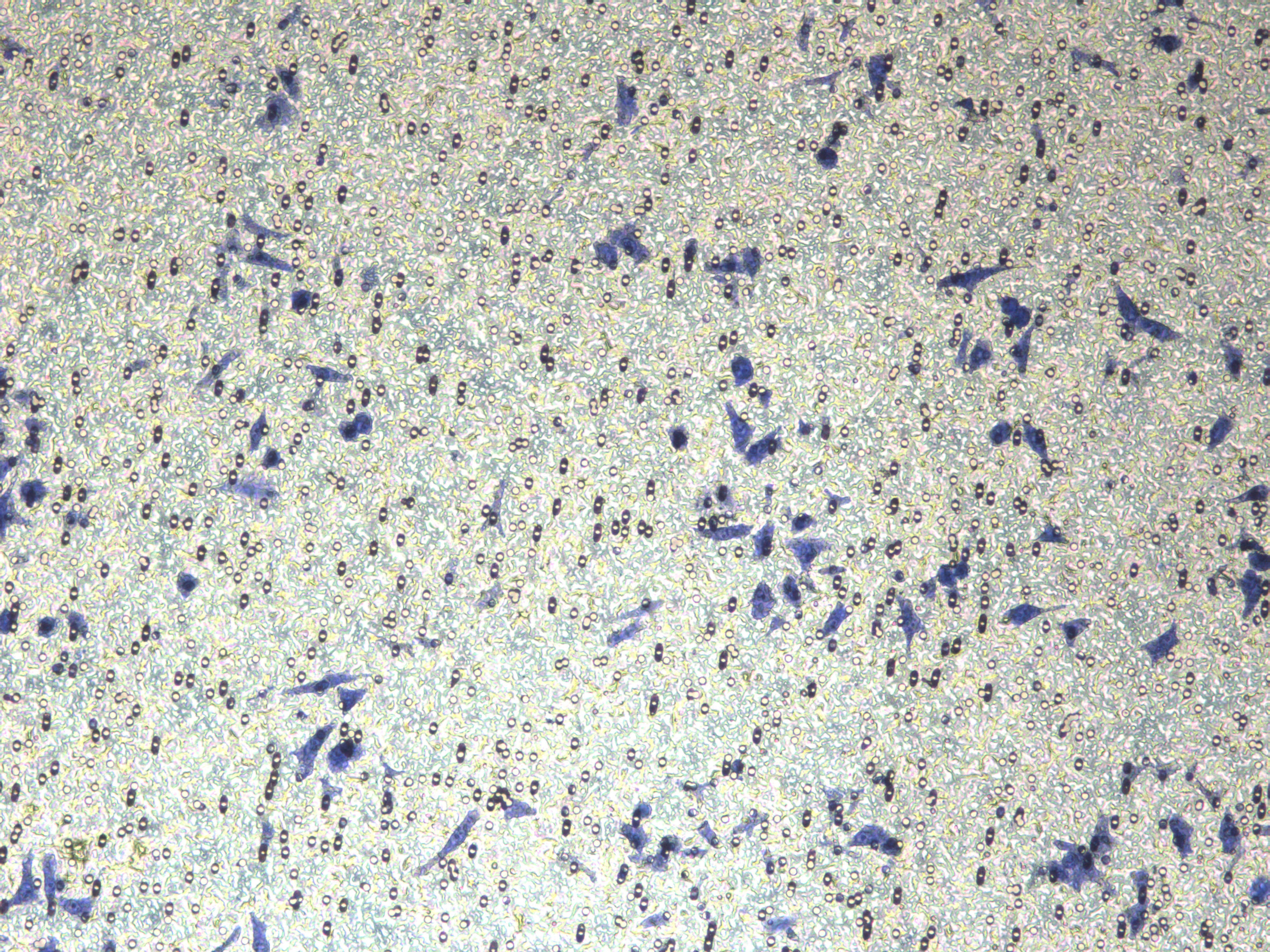

Supplement: S9 File — (ZIP) [file pone.0334639.s009.zip › S 14. File. Original Images. Fig7/S 14. File. Original FIgures. Fig.7/7d/BEL-7402/mtor/bel cxcl3 2ngml.jpg]

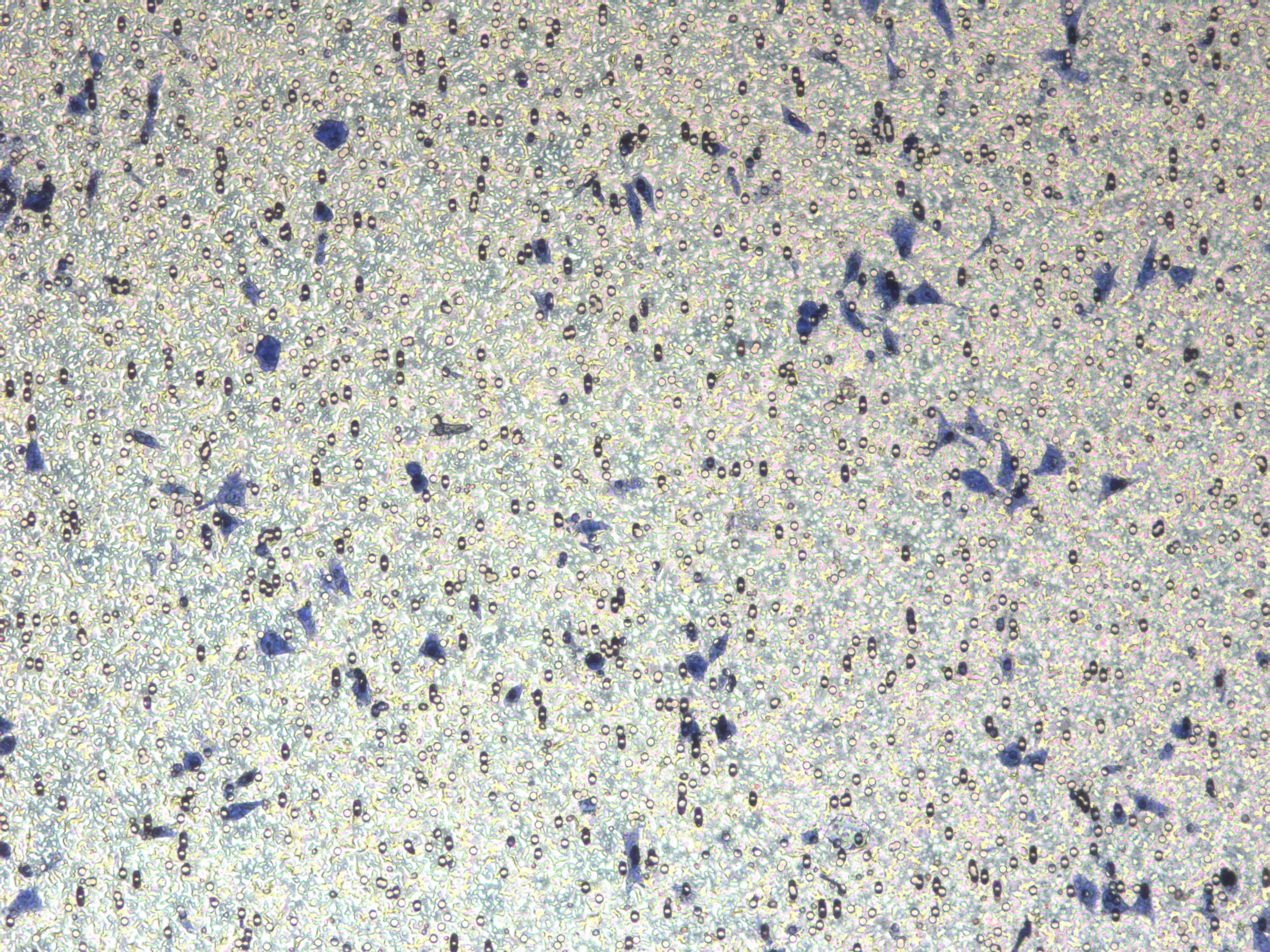

Supplement: S9 File — (ZIP) [file pone.0334639.s009.zip › S 14. File. Original Images. Fig7/S 14. File. Original FIgures. Fig.7/7d/BEL-7402/mtor/bel cxcl3 30ngml.jpg]

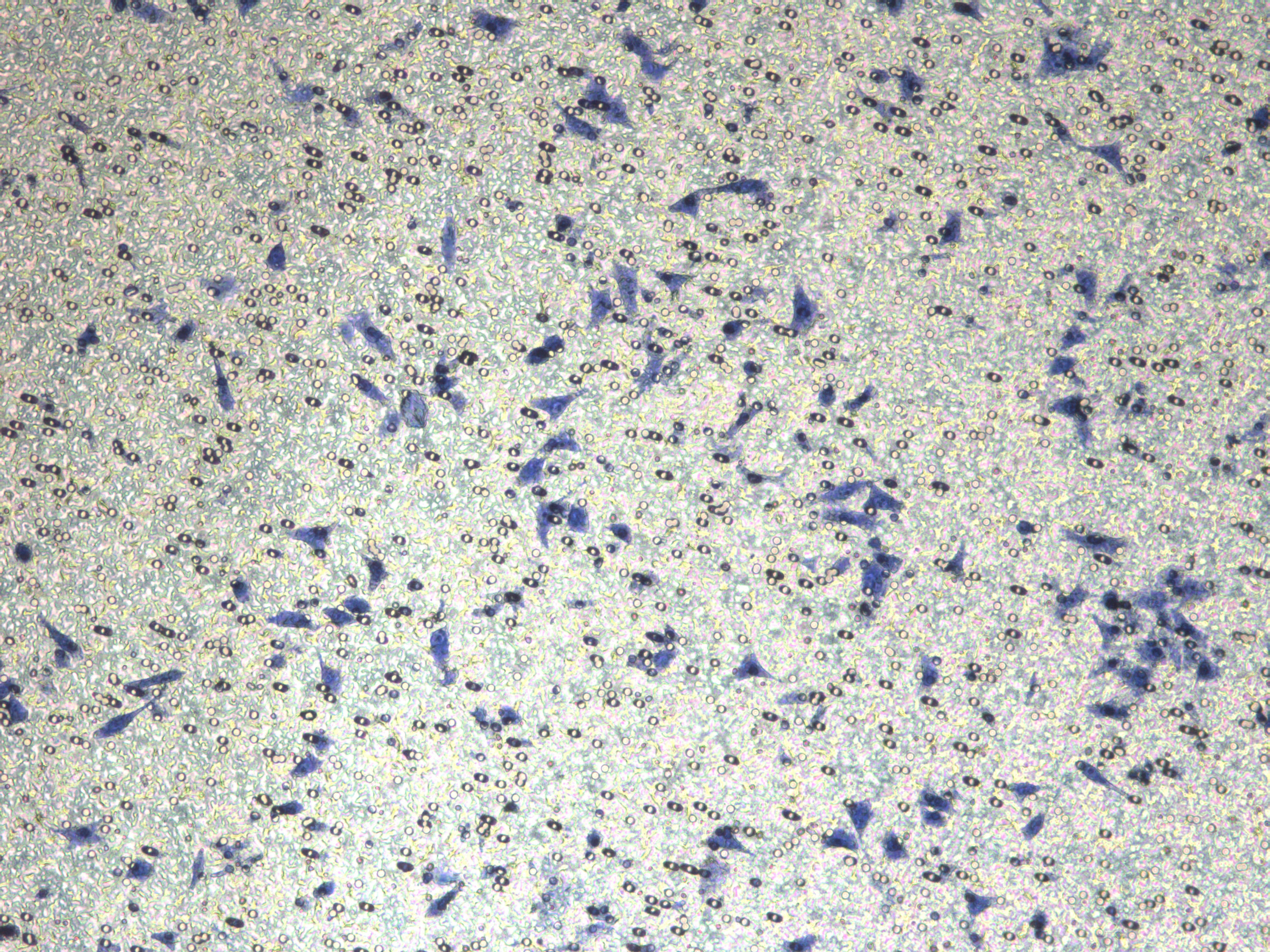

Supplement: S9 File — (ZIP) [file pone.0334639.s009.zip › S 14. File. Original Images. Fig7/S 14. File. Original FIgures. Fig.7/7d/BEL-7402/mtor/bel cxcl3 5ngml.jpg]

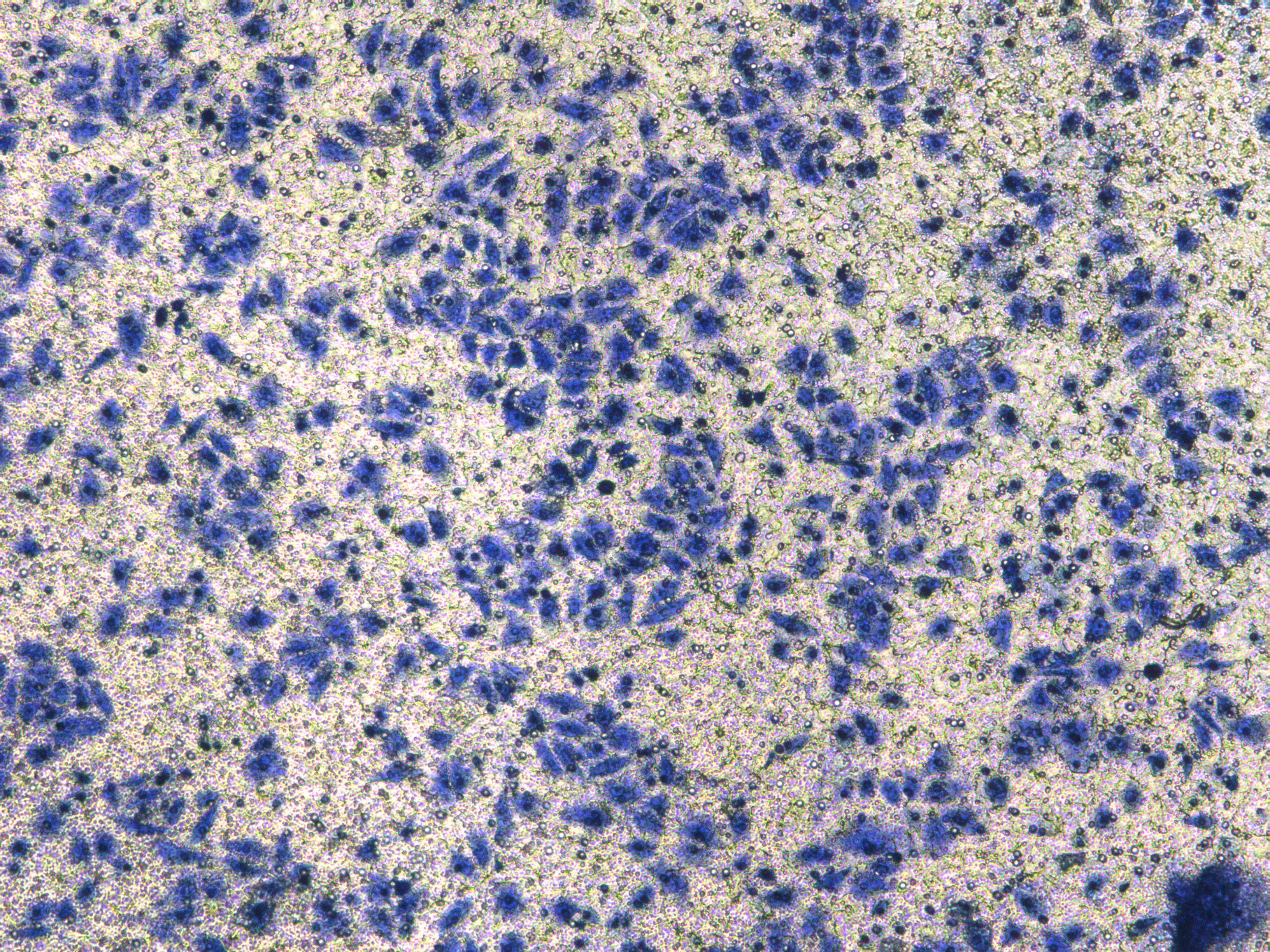

Supplement: S9 File — (ZIP) [file pone.0334639.s009.zip › S 14. File. Original Images. Fig7/S 14. File. Original FIgures. Fig.7/7d/HEPG2/DMSO/DMSO/Hepg2 cxcl3 10ngml.jpg]

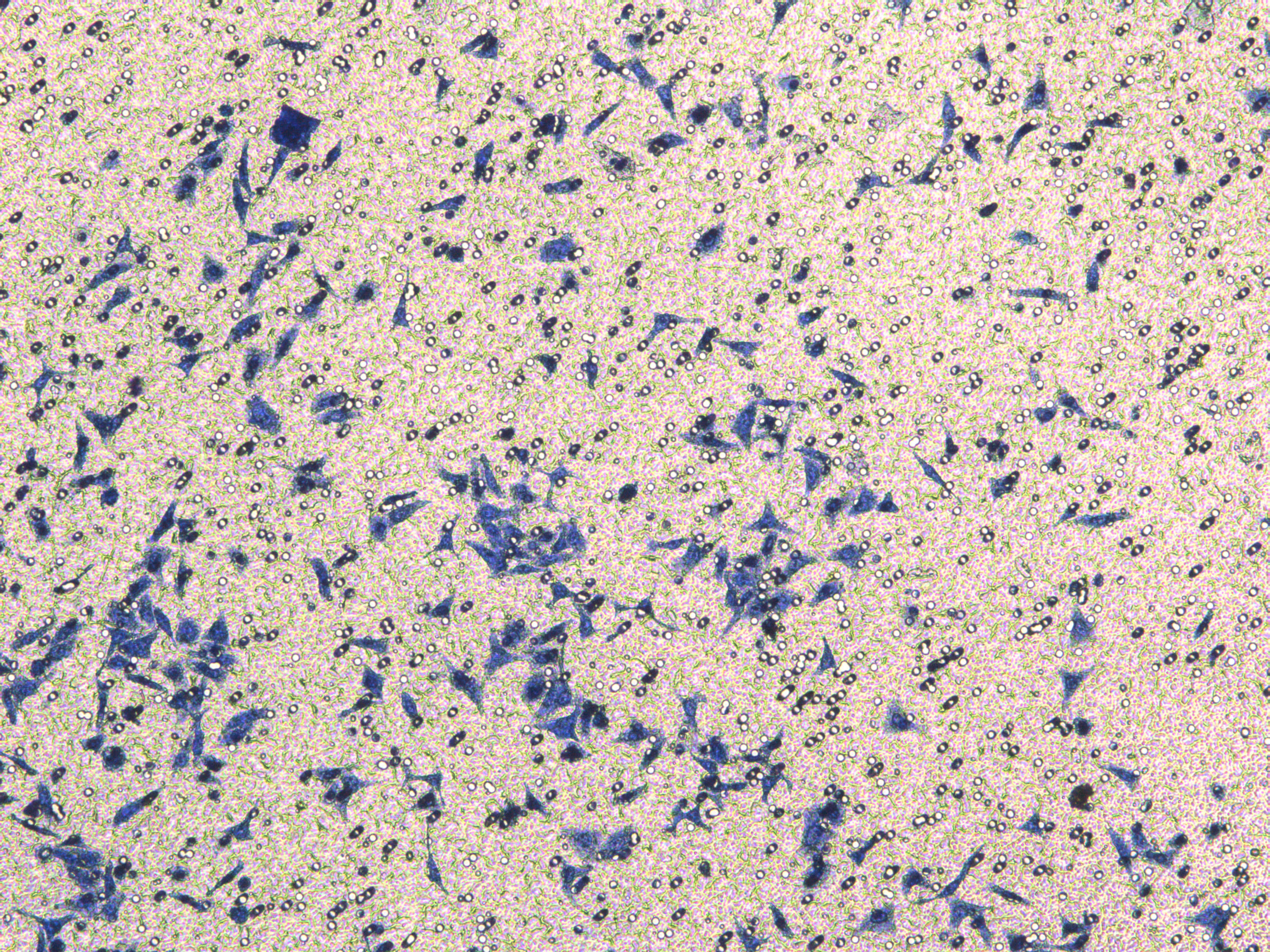

Supplement: S9 File — (ZIP) [file pone.0334639.s009.zip › S 14. File. Original Images. Fig7/S 14. File. Original FIgures. Fig.7/7d/HEPG2/DMSO/DMSO/Hepg2 cxcl3 2ngml.jpg]

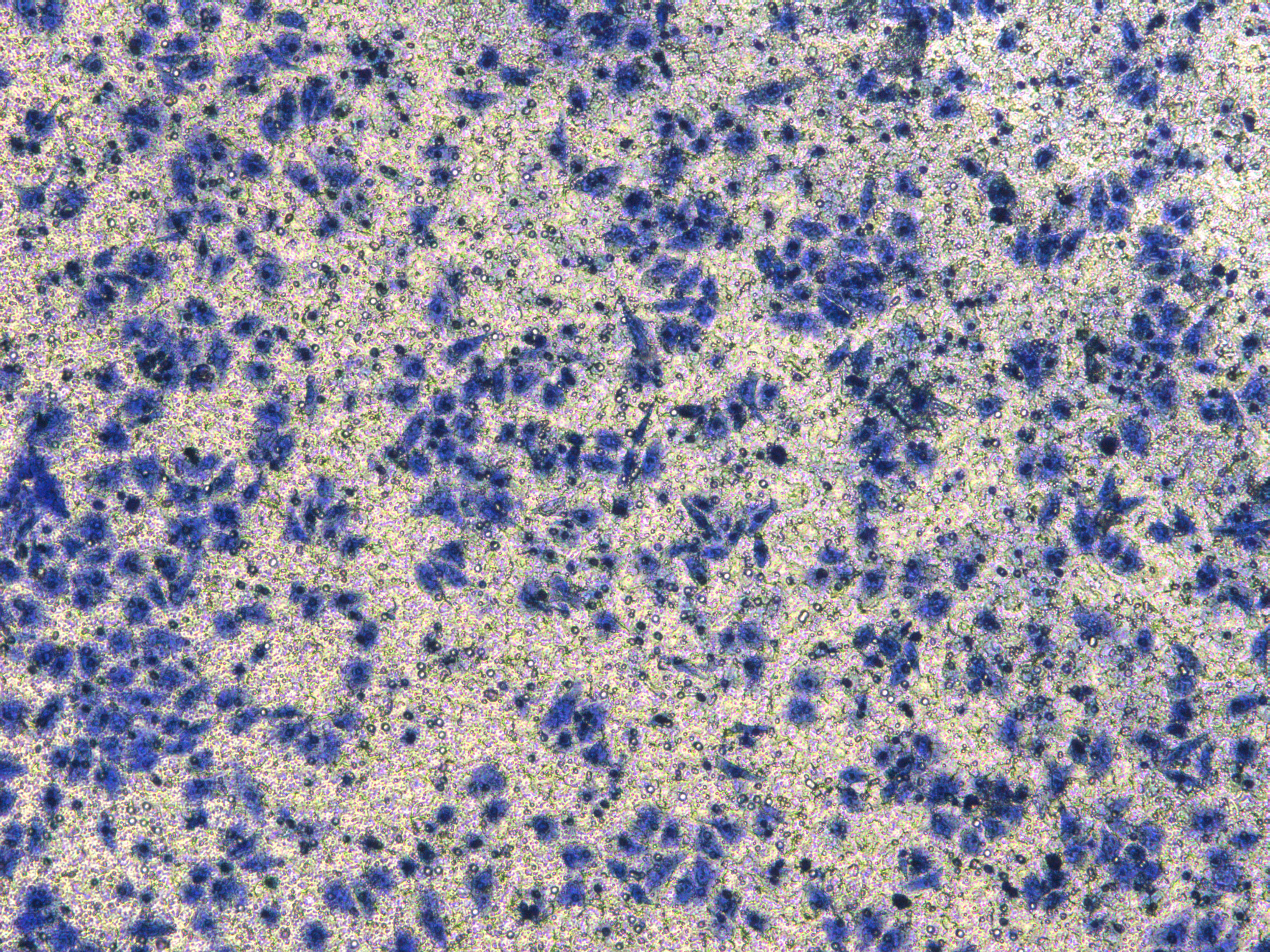

Supplement: S9 File — (ZIP) [file pone.0334639.s009.zip › S 14. File. Original Images. Fig7/S 14. File. Original FIgures. Fig.7/7d/HEPG2/DMSO/DMSO/Hepg2 cxcl3 30ngml.jpg]

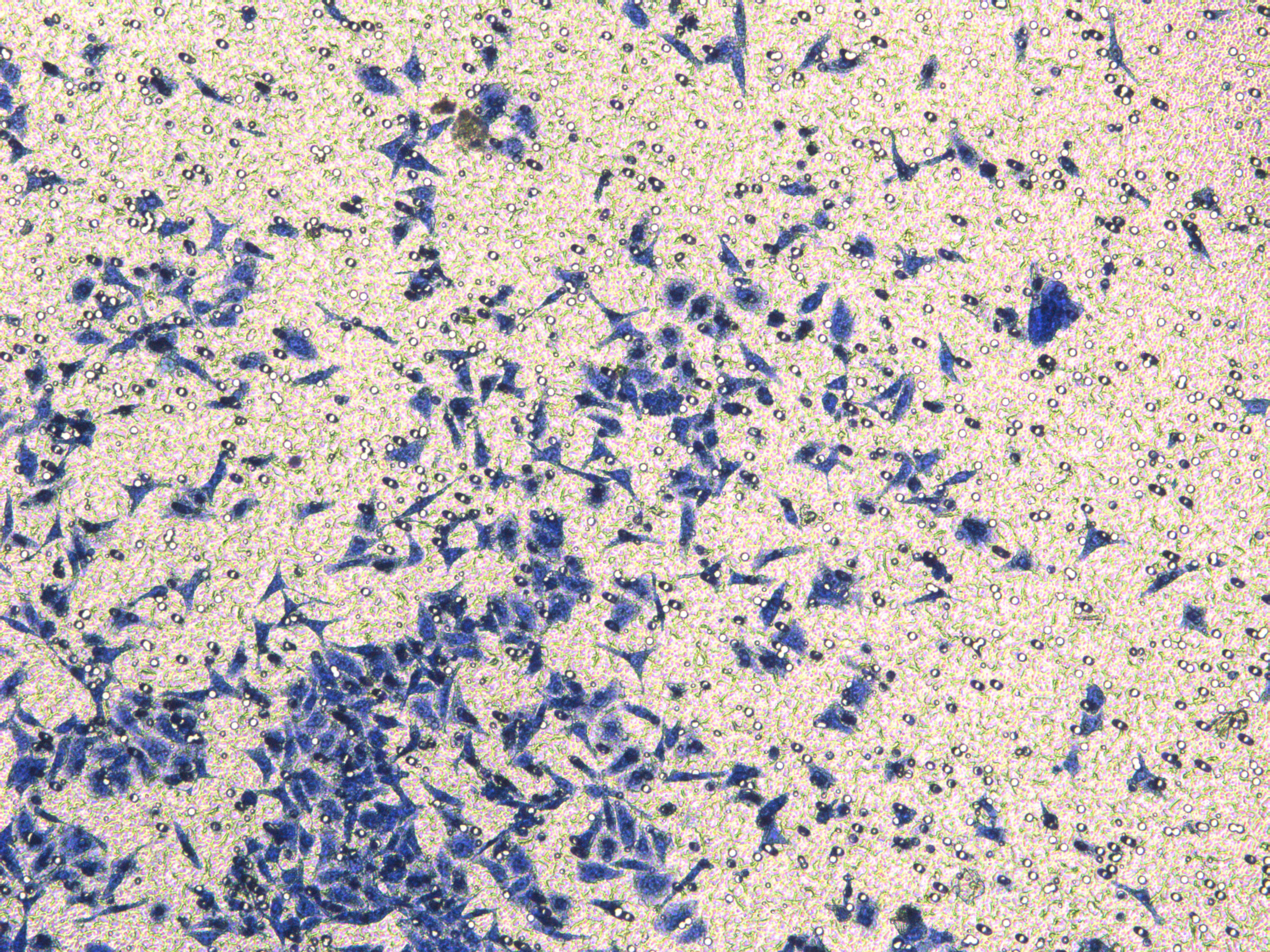

Supplement: S9 File — (ZIP) [file pone.0334639.s009.zip › S 14. File. Original Images. Fig7/S 14. File. Original FIgures. Fig.7/7d/HEPG2/DMSO/DMSO/Hepg2 cxcl3 5ngml.jpg]

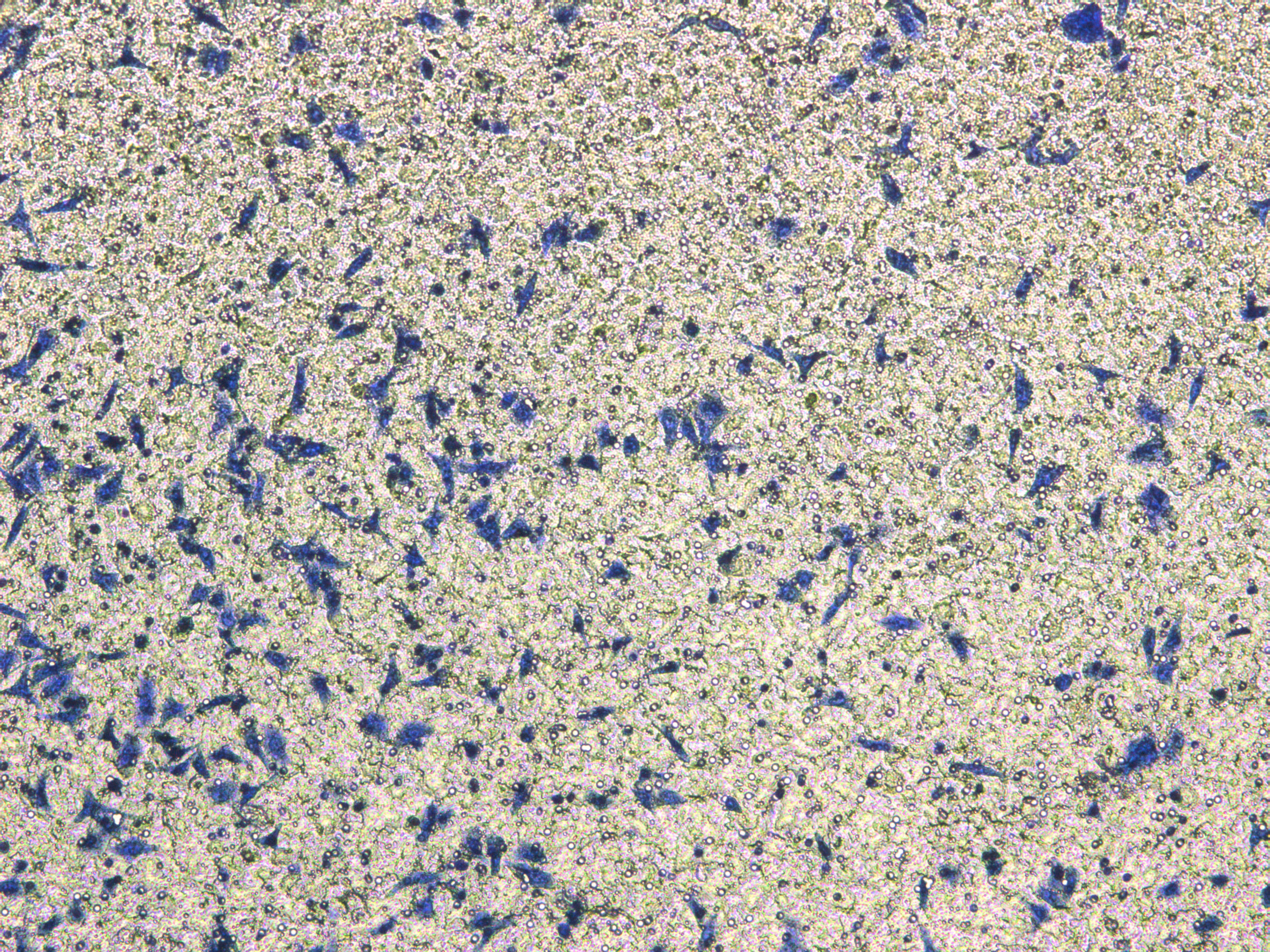

Supplement: S9 File — (ZIP) [file pone.0334639.s009.zip › S 14. File. Original Images. Fig7/S 14. File. Original FIgures. Fig.7/7d/HEPG2/DMSO/DMSO/Hepg2 cxcl3----0ngml.jpg]

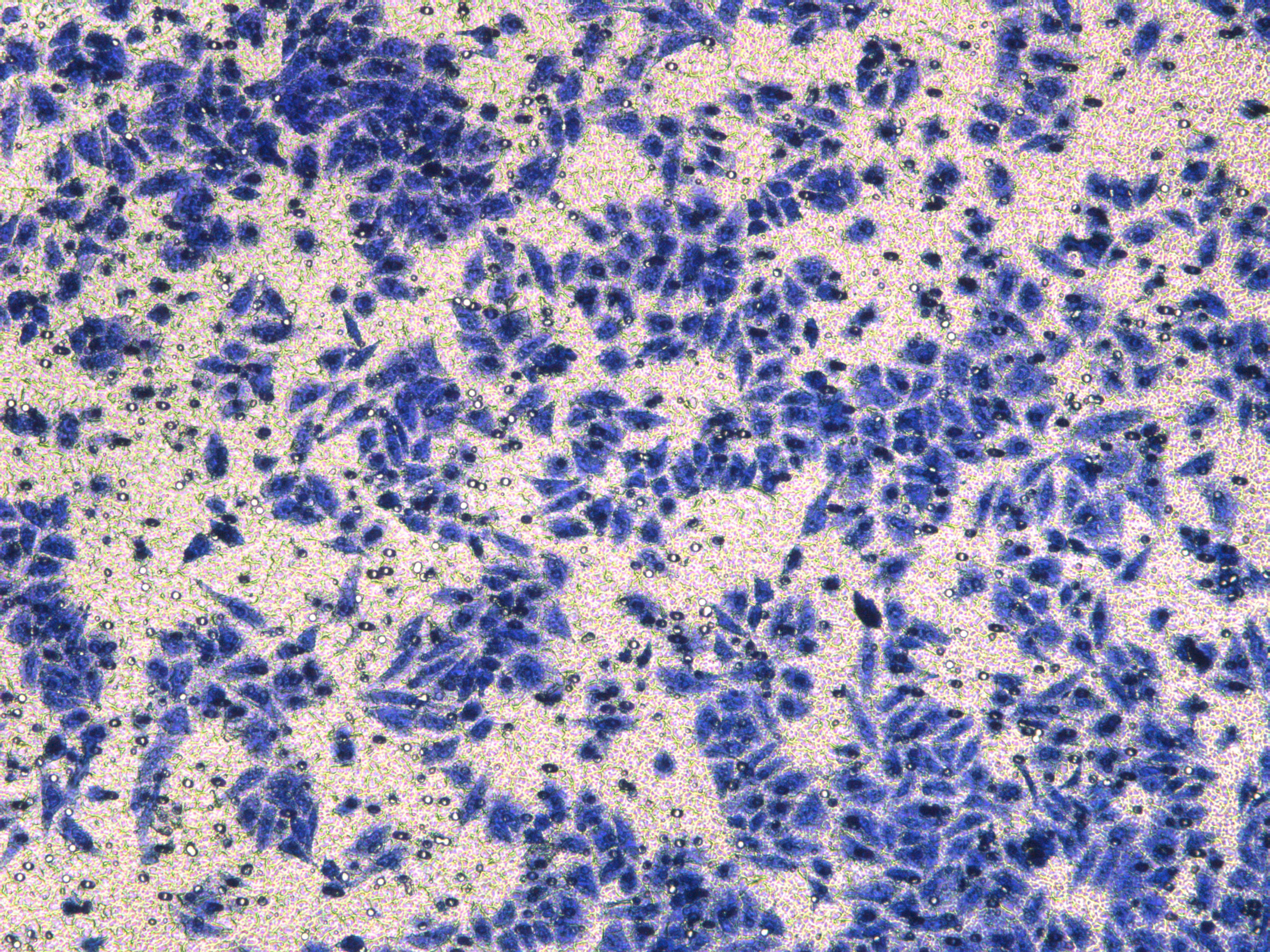

Supplement: S9 File — (ZIP) [file pone.0334639.s009.zip › S 14. File. Original Images. Fig7/S 14. File. Original FIgures. Fig.7/7d/HEPG2/DMSO/DMSO/Hepg2 cxcl3---20ngml.jpg]

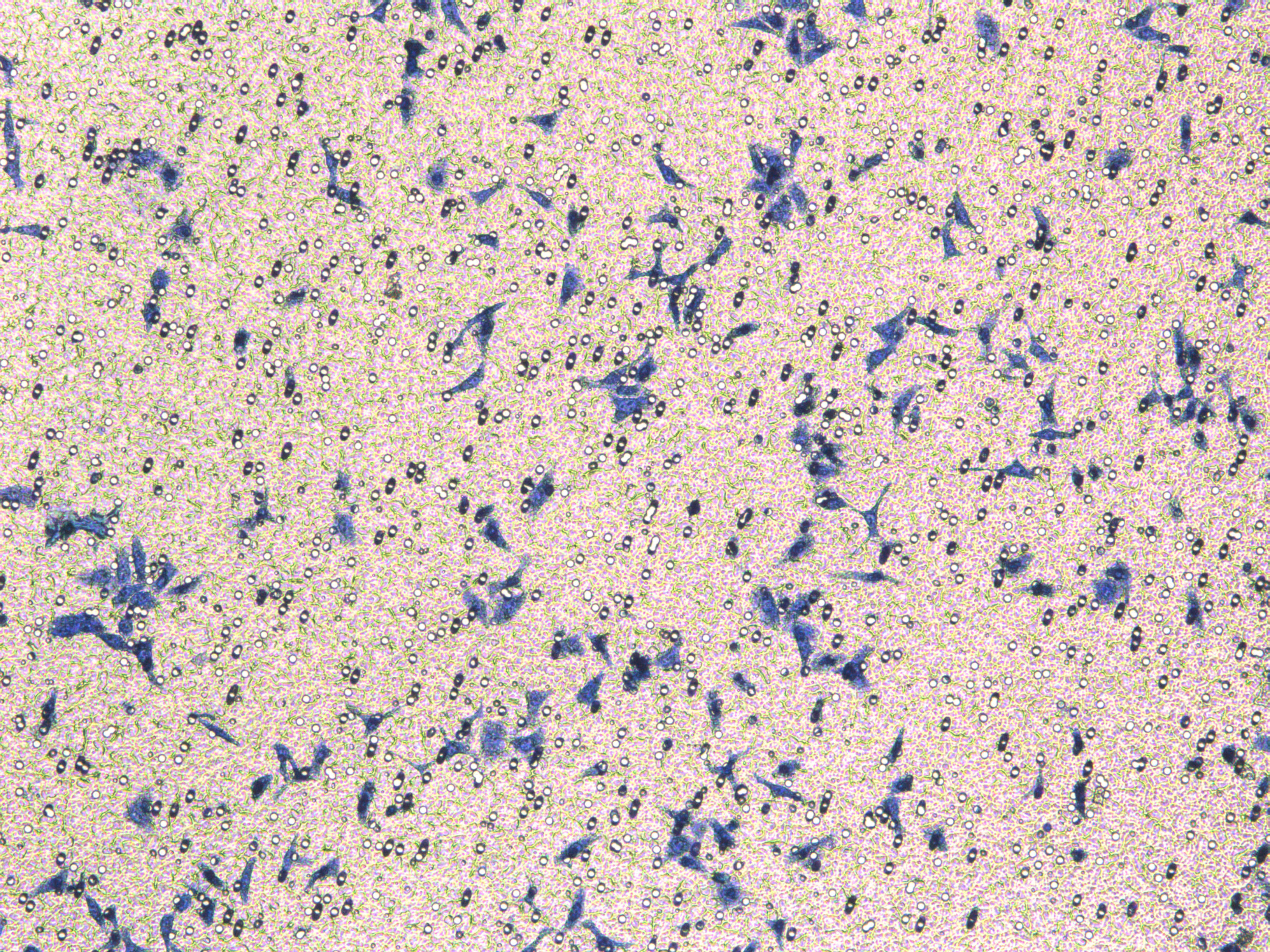

Supplement: S9 File — (ZIP) [file pone.0334639.s009.zip › S 14. File. Original Images. Fig7/S 14. File. Original FIgures. Fig.7/7d/HEPG2/mtor/MTOR/Hepg2 cxcl3 0ngml.jpg]

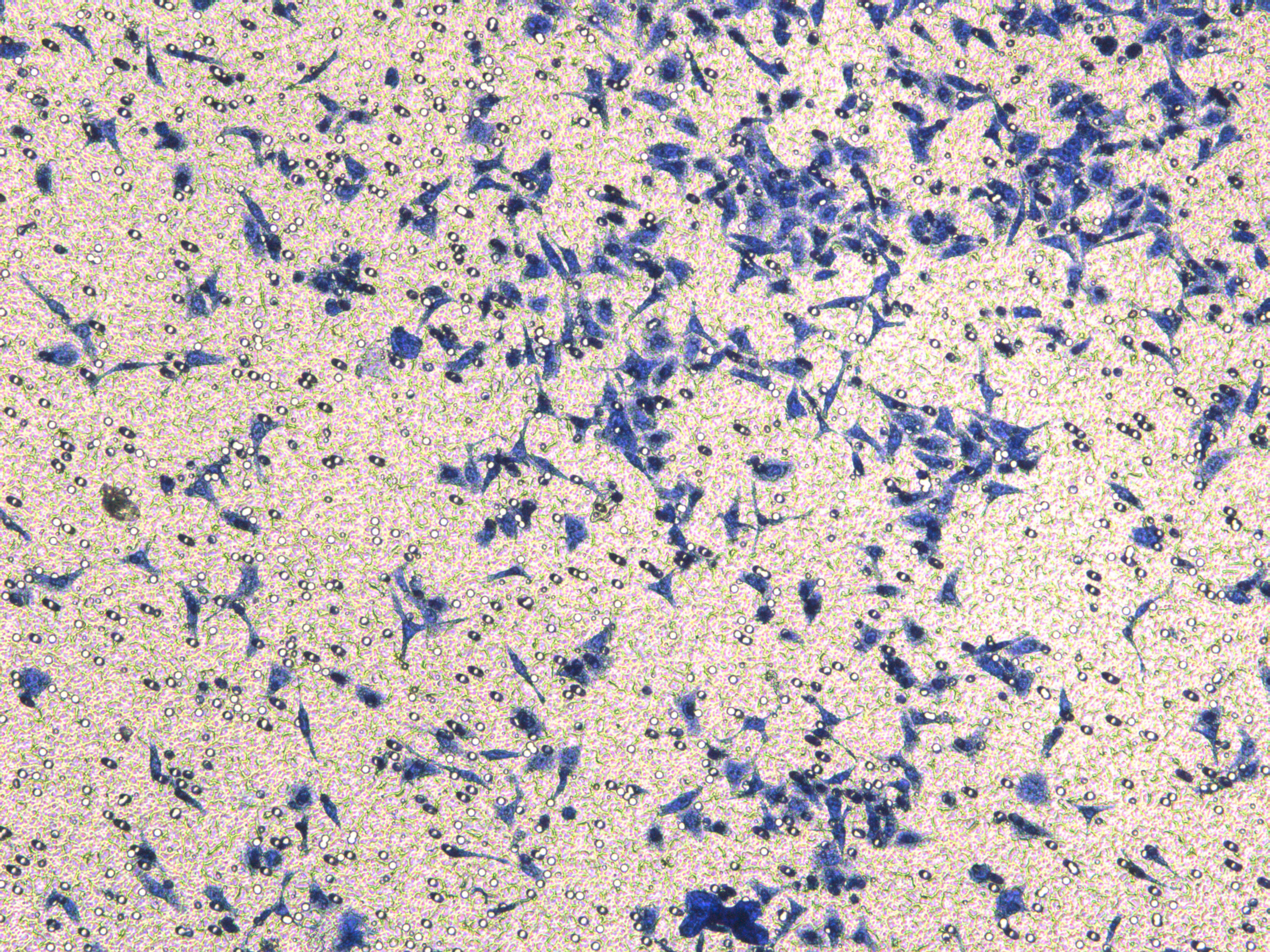

Supplement: S9 File — (ZIP) [file pone.0334639.s009.zip › S 14. File. Original Images. Fig7/S 14. File. Original FIgures. Fig.7/7d/HEPG2/mtor/MTOR/Hepg2 cxcl3 20ngml.jpg]

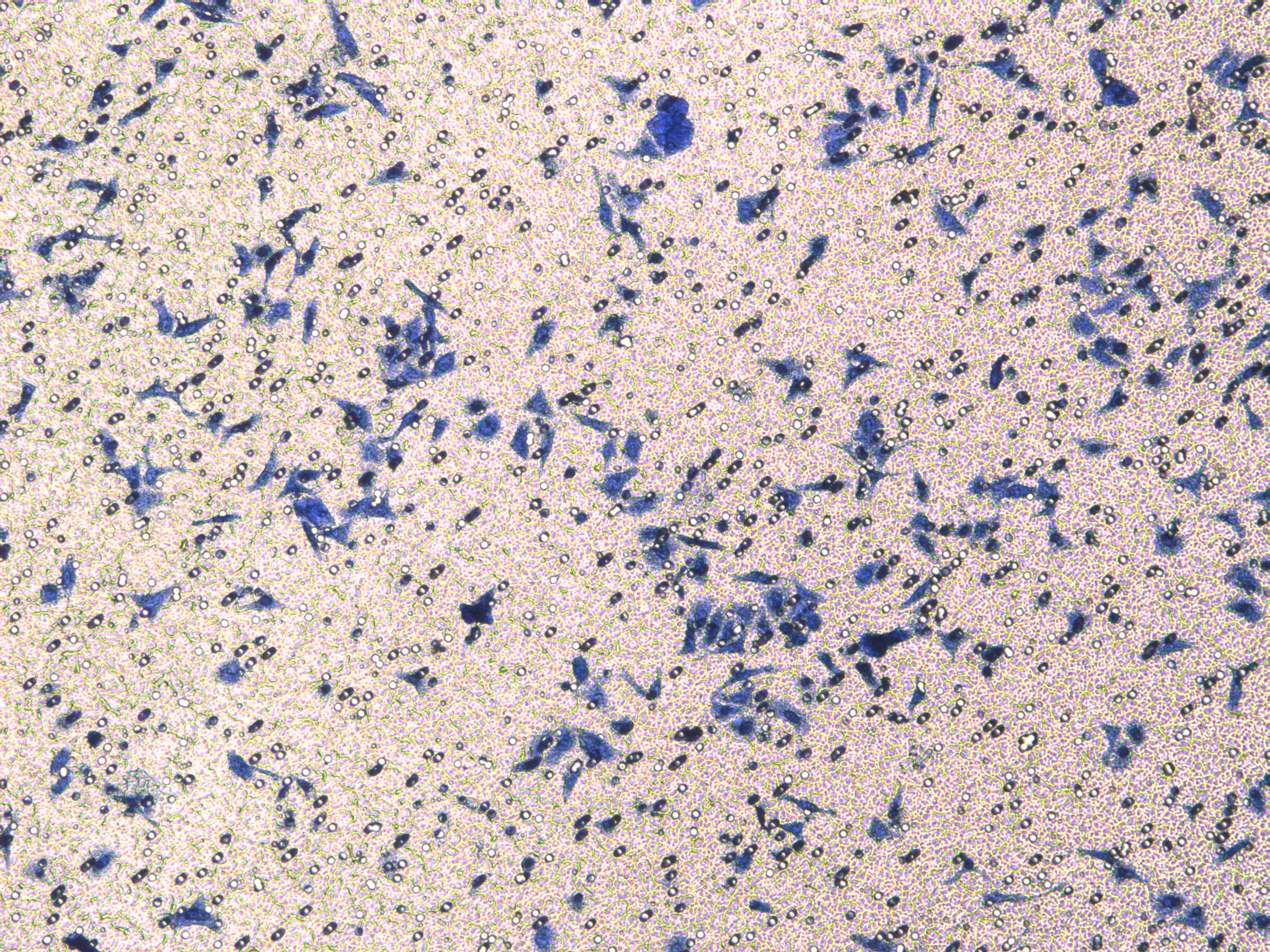

Supplement: S9 File — (ZIP) [file pone.0334639.s009.zip › S 14. File. Original Images. Fig7/S 14. File. Original FIgures. Fig.7/7d/HEPG2/mtor/MTOR/Hepg2 cxcl3 2ngml.jpg]

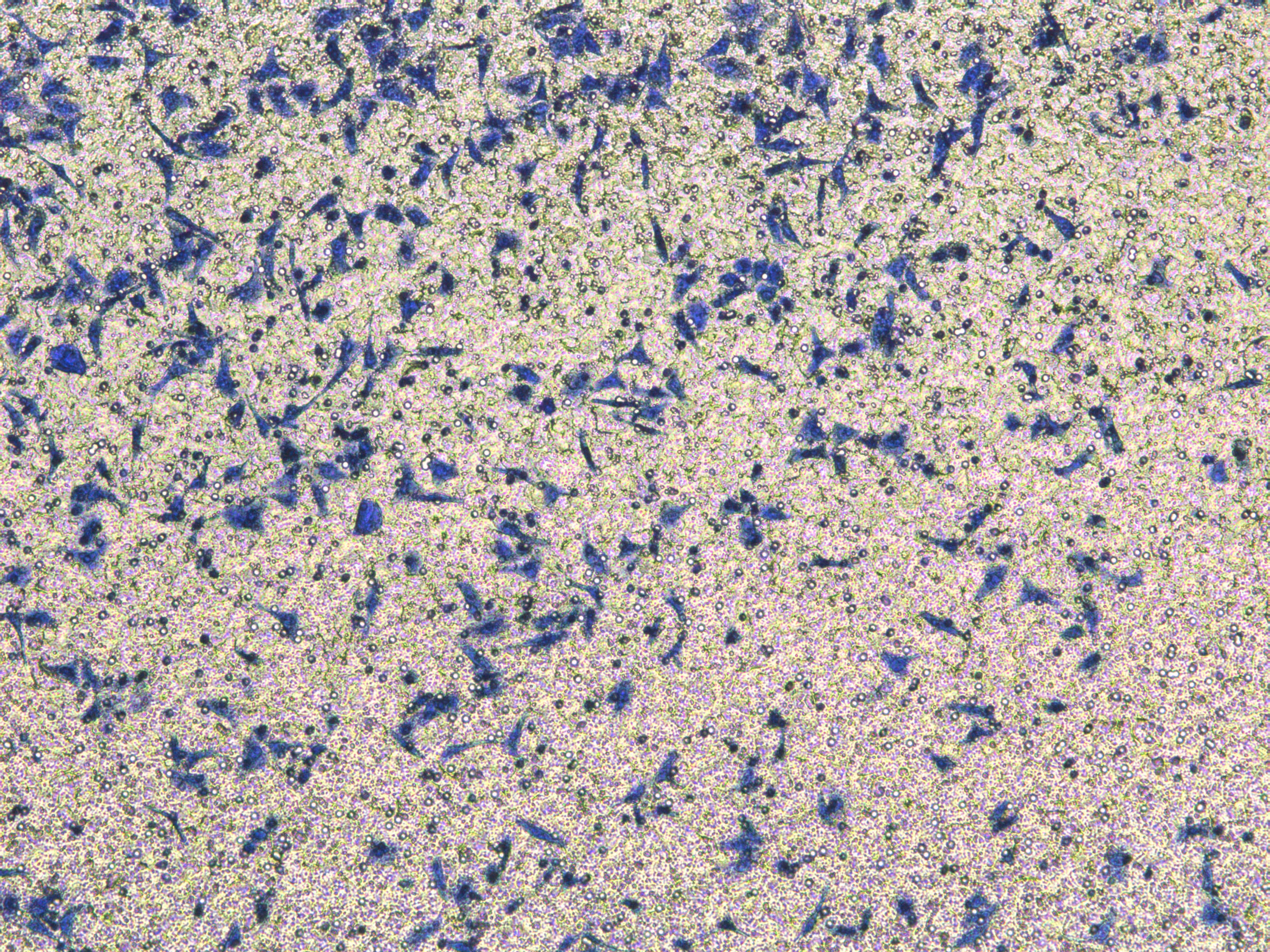

Supplement: S9 File — (ZIP) [file pone.0334639.s009.zip › S 14. File. Original Images. Fig7/S 14. File. Original FIgures. Fig.7/7d/HEPG2/mtor/MTOR/Hepg2 cxcl3---30ngml.jpg]

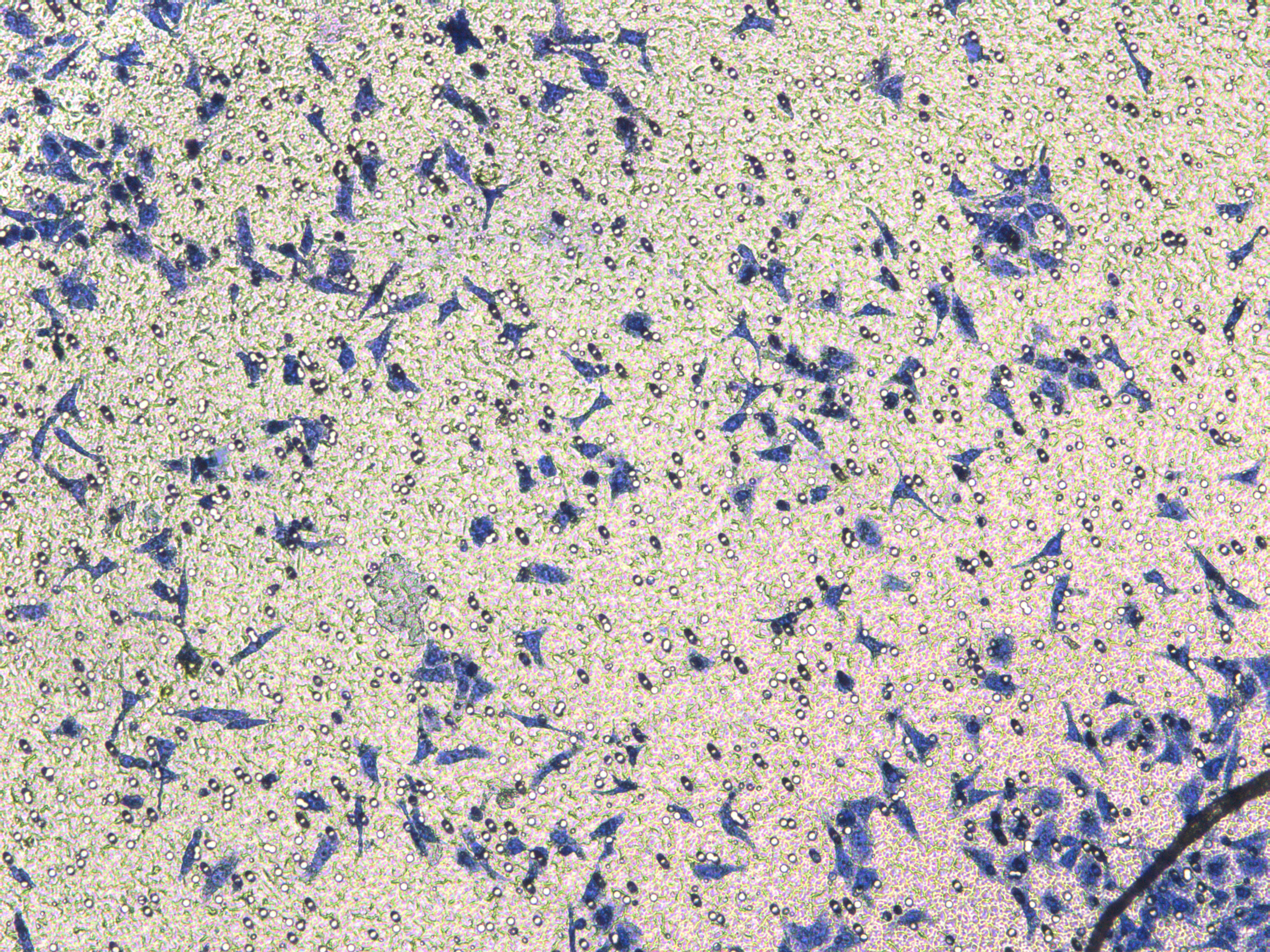

Supplement: S9 File — (ZIP) [file pone.0334639.s009.zip › S 14. File. Original Images. Fig7/S 14. File. Original FIgures. Fig.7/7d/HEPG2/mtor/MTOR/Hepg2 cxcl3---5ngml.jpg]

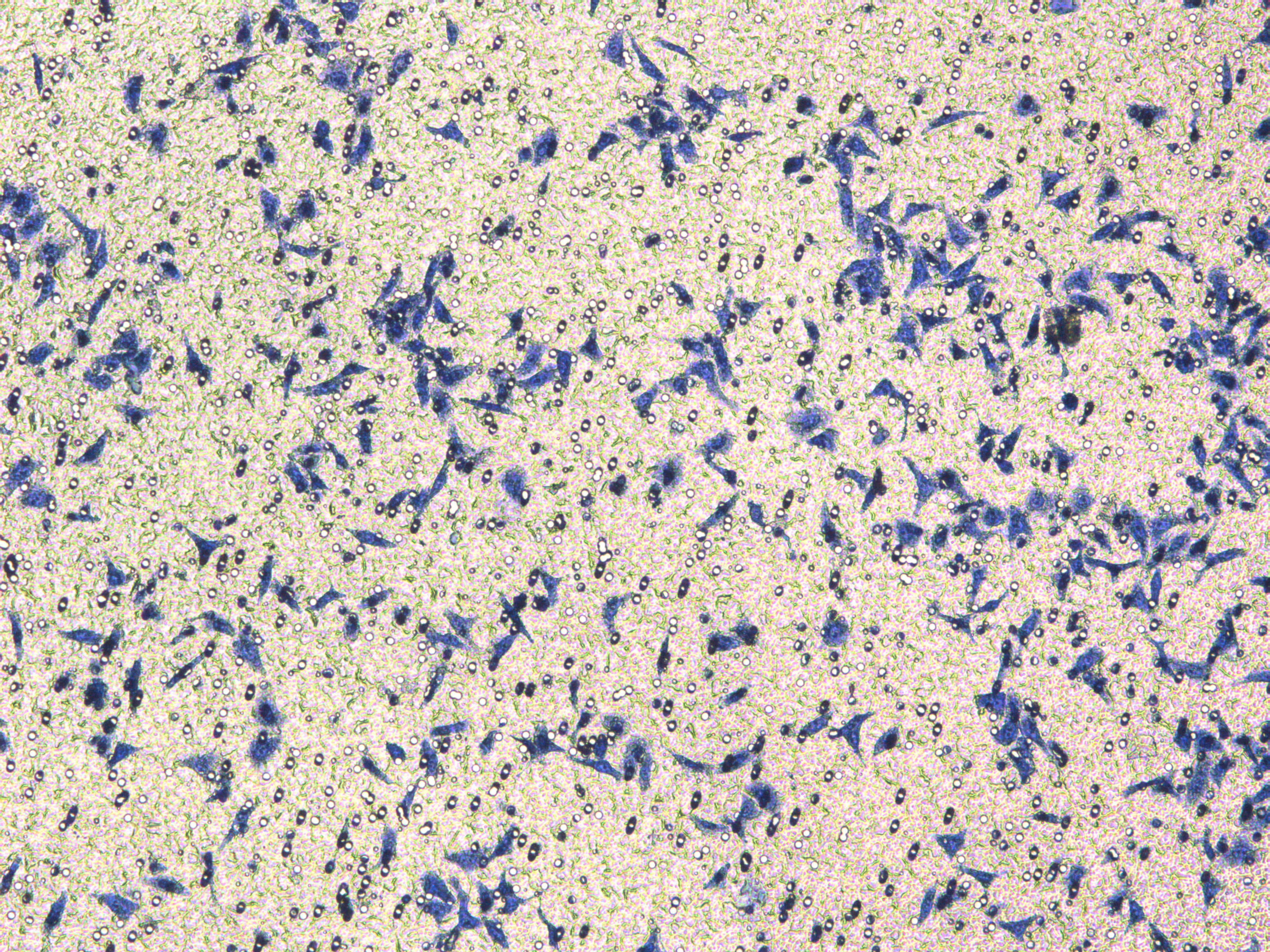

Supplement: S9 File — (ZIP) [file pone.0334639.s009.zip › S 14. File. Original Images. Fig7/S 14. File. Original FIgures. Fig.7/7d/HEPG2/mtor/MTOR/Hepg2 cxcl3--10ngml.jpg]

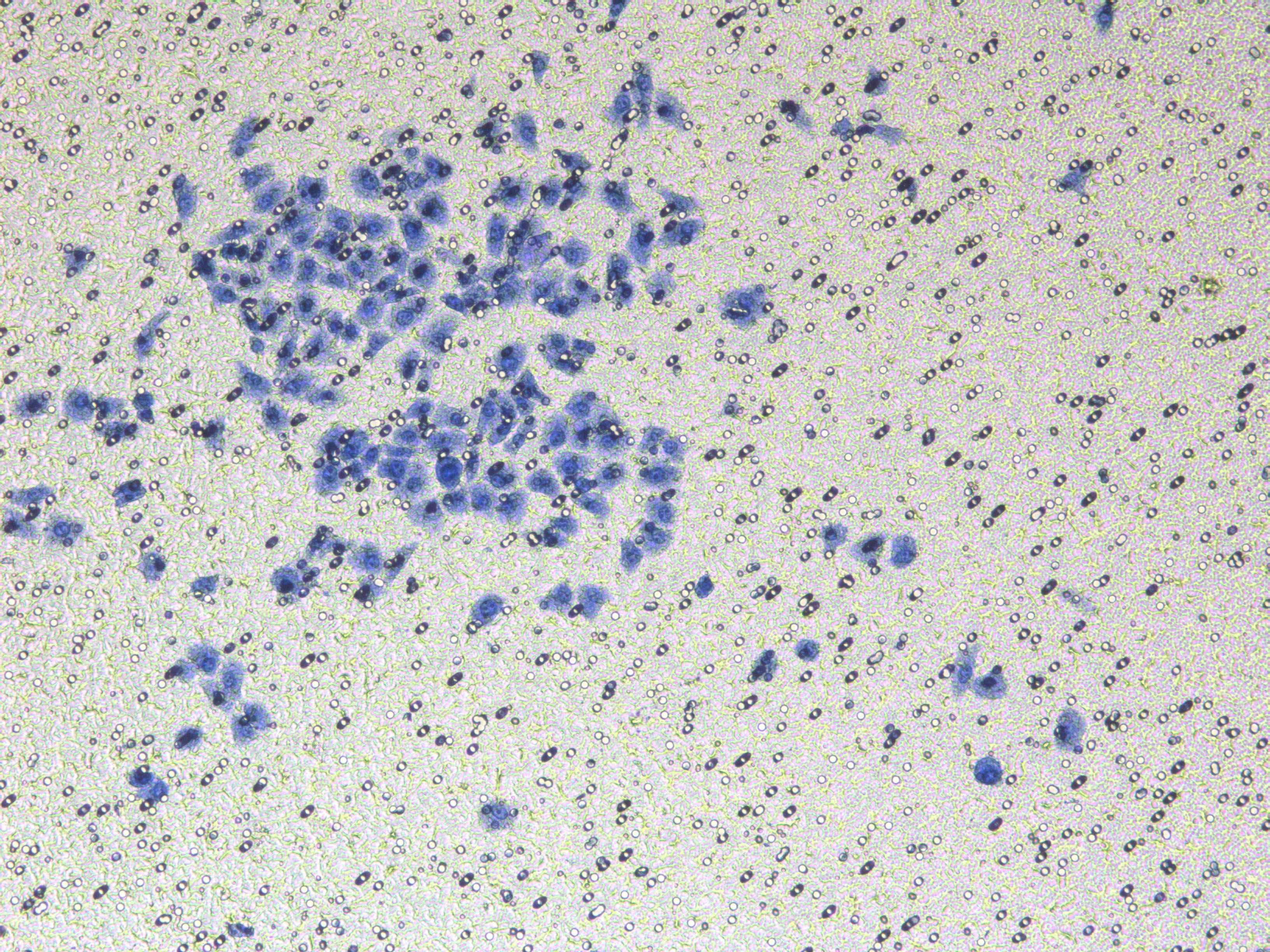

Supplement: S9 File — (ZIP) [file pone.0334639.s009.zip › S 14. File. Original Images. Fig7/S 14. File. Original FIgures. Fig.7/7d/SMMC-7721/DMSO/7721 cxcl3 0ngml-.jpg]

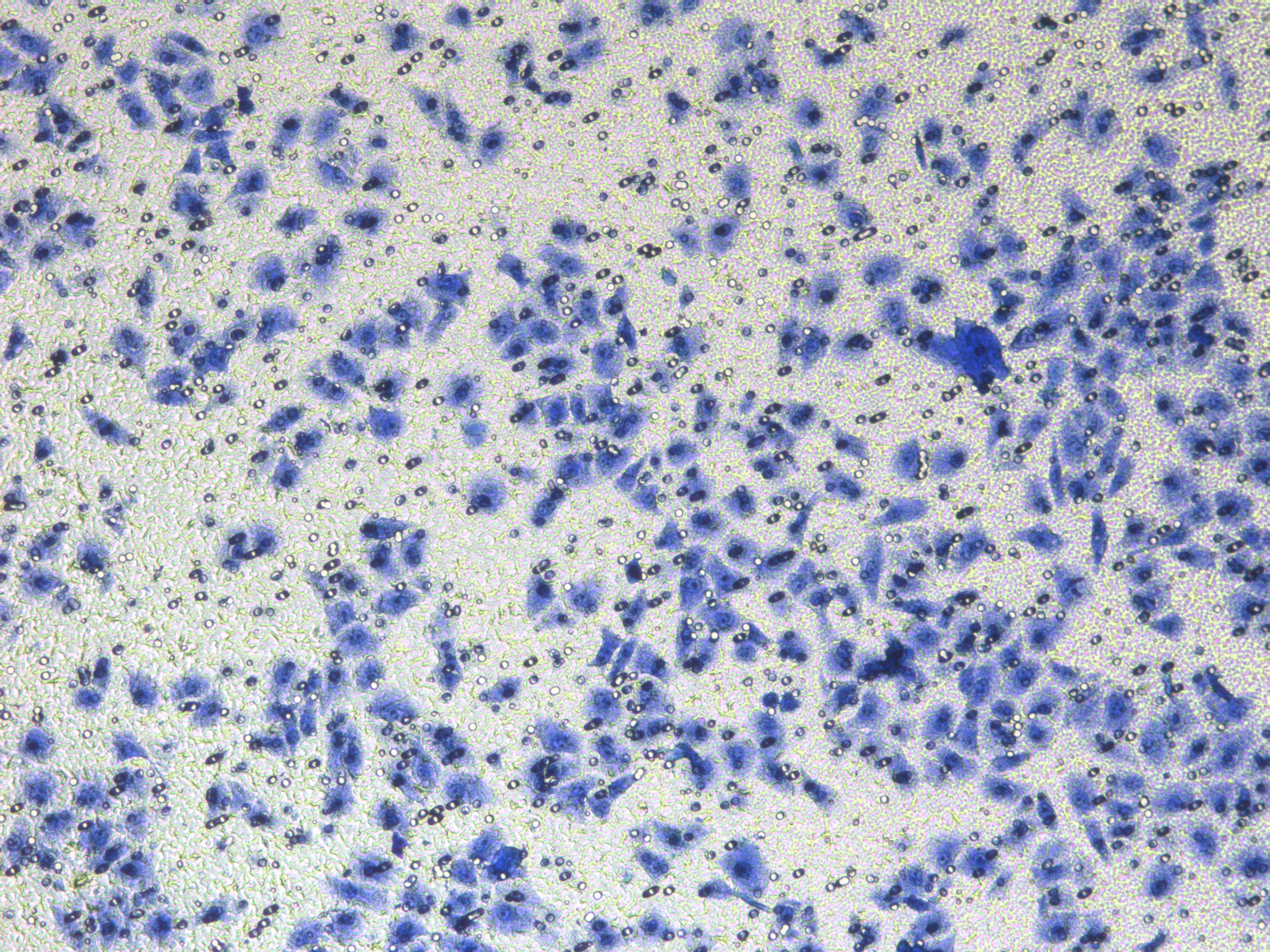

Supplement: S9 File — (ZIP) [file pone.0334639.s009.zip › S 14. File. Original Images. Fig7/S 14. File. Original FIgures. Fig.7/7d/SMMC-7721/DMSO/7721 cxcl3 10ngml-.jpg]

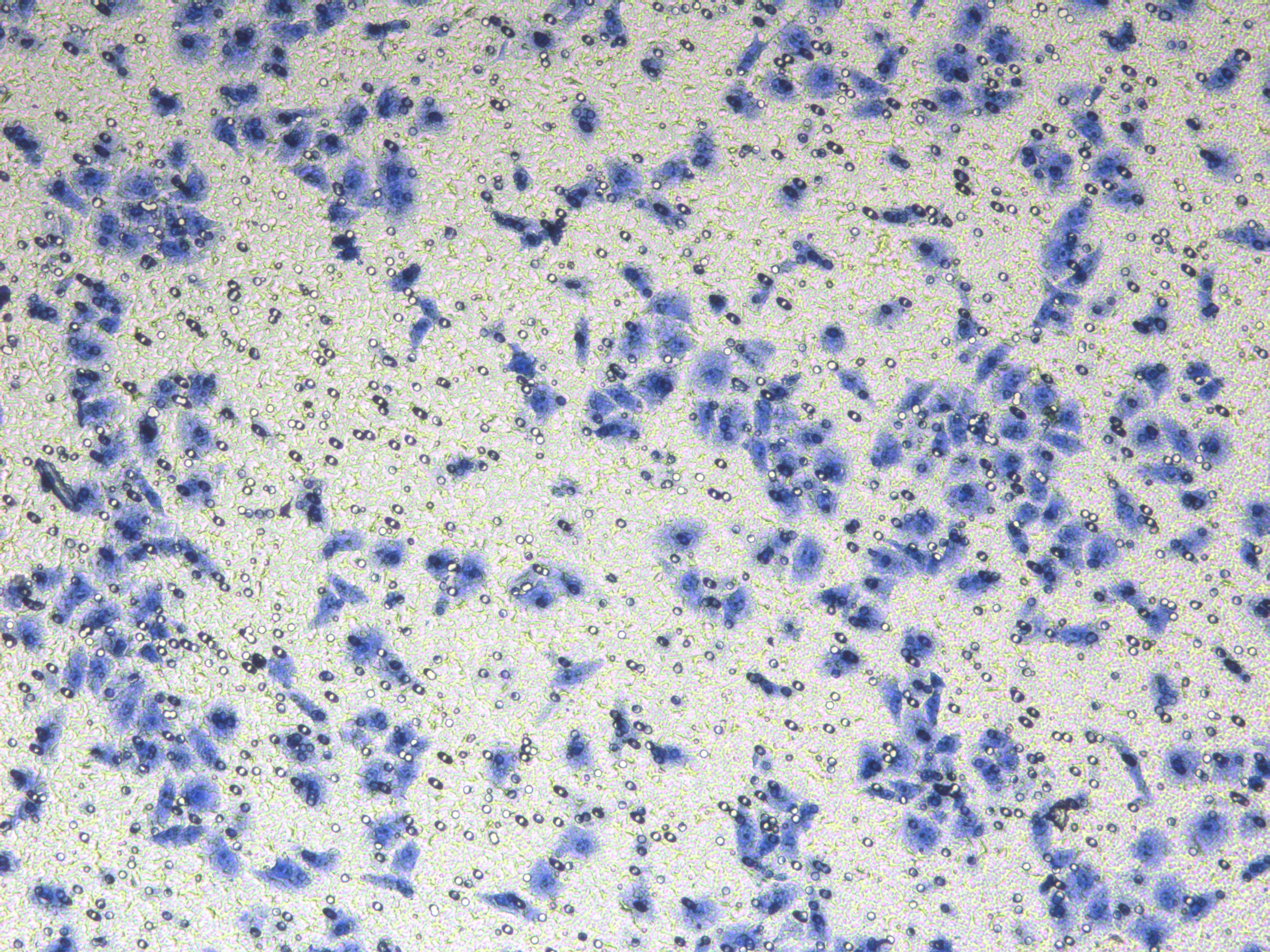

Supplement: S9 File — (ZIP) [file pone.0334639.s009.zip › S 14. File. Original Images. Fig7/S 14. File. Original FIgures. Fig.7/7d/SMMC-7721/DMSO/7721 cxcl3 20ngml-.jpg]

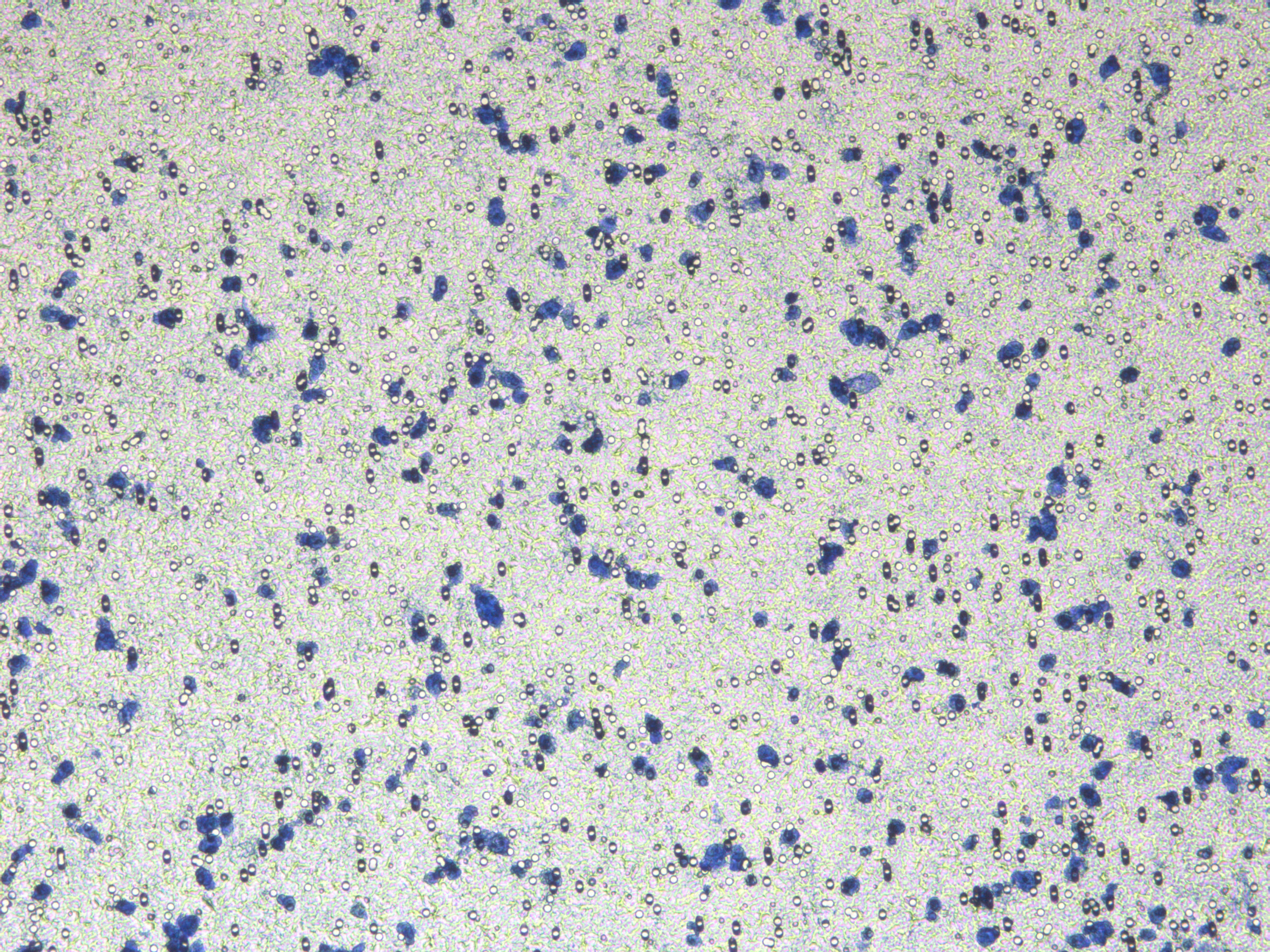

Supplement: S9 File — (ZIP) [file pone.0334639.s009.zip › S 14. File. Original Images. Fig7/S 14. File. Original FIgures. Fig.7/7d/SMMC-7721/DMSO/7721 cxcl3 2ngml-.jpg]

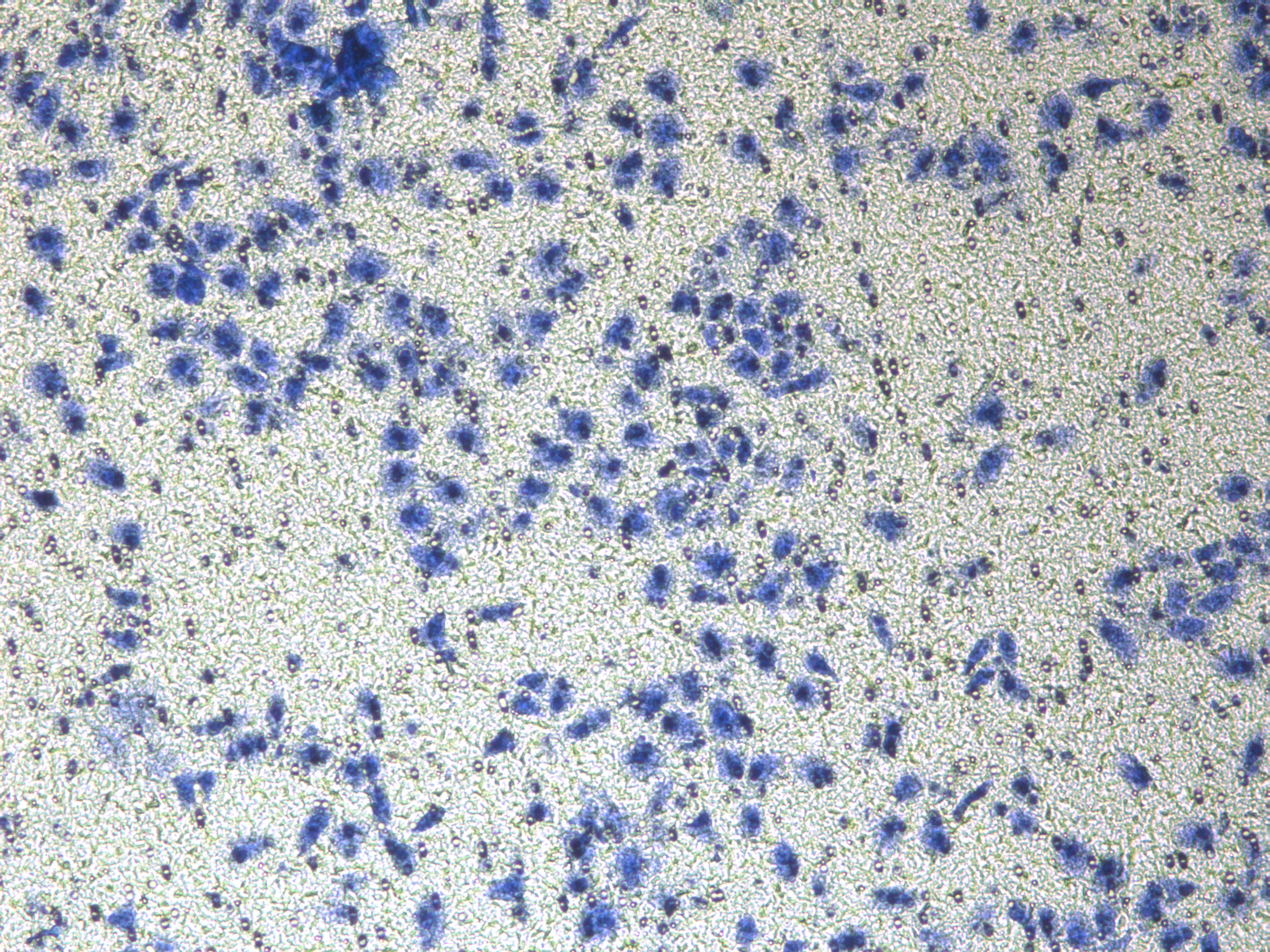

Supplement: S9 File — (ZIP) [file pone.0334639.s009.zip › S 14. File. Original Images. Fig7/S 14. File. Original FIgures. Fig.7/7d/SMMC-7721/DMSO/7721 cxcl3 30ngml-.jpg]

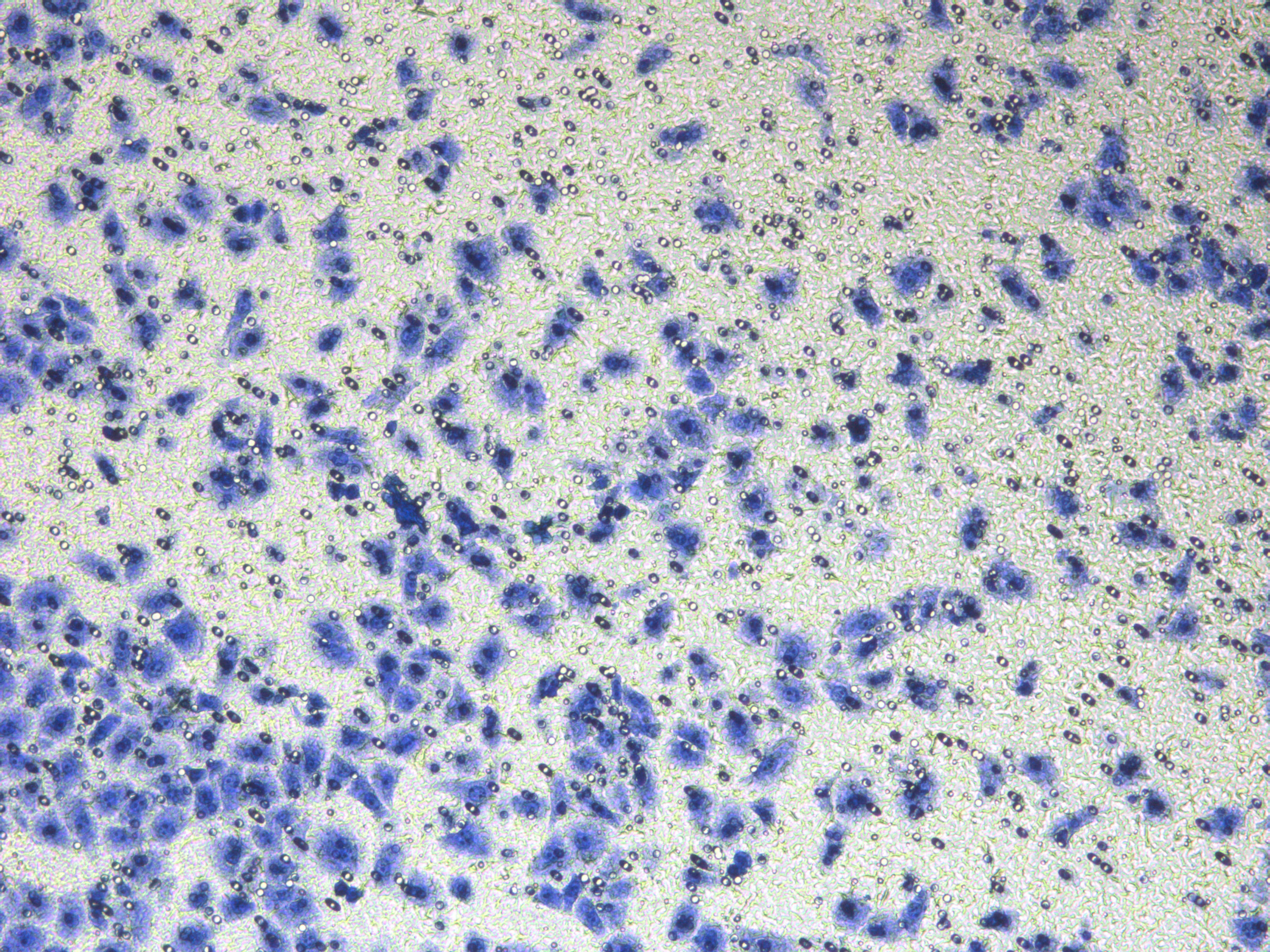

Supplement: S9 File — (ZIP) [file pone.0334639.s009.zip › S 14. File. Original Images. Fig7/S 14. File. Original FIgures. Fig.7/7d/SMMC-7721/DMSO/7721 cxcl3 5ngml-.jpg]

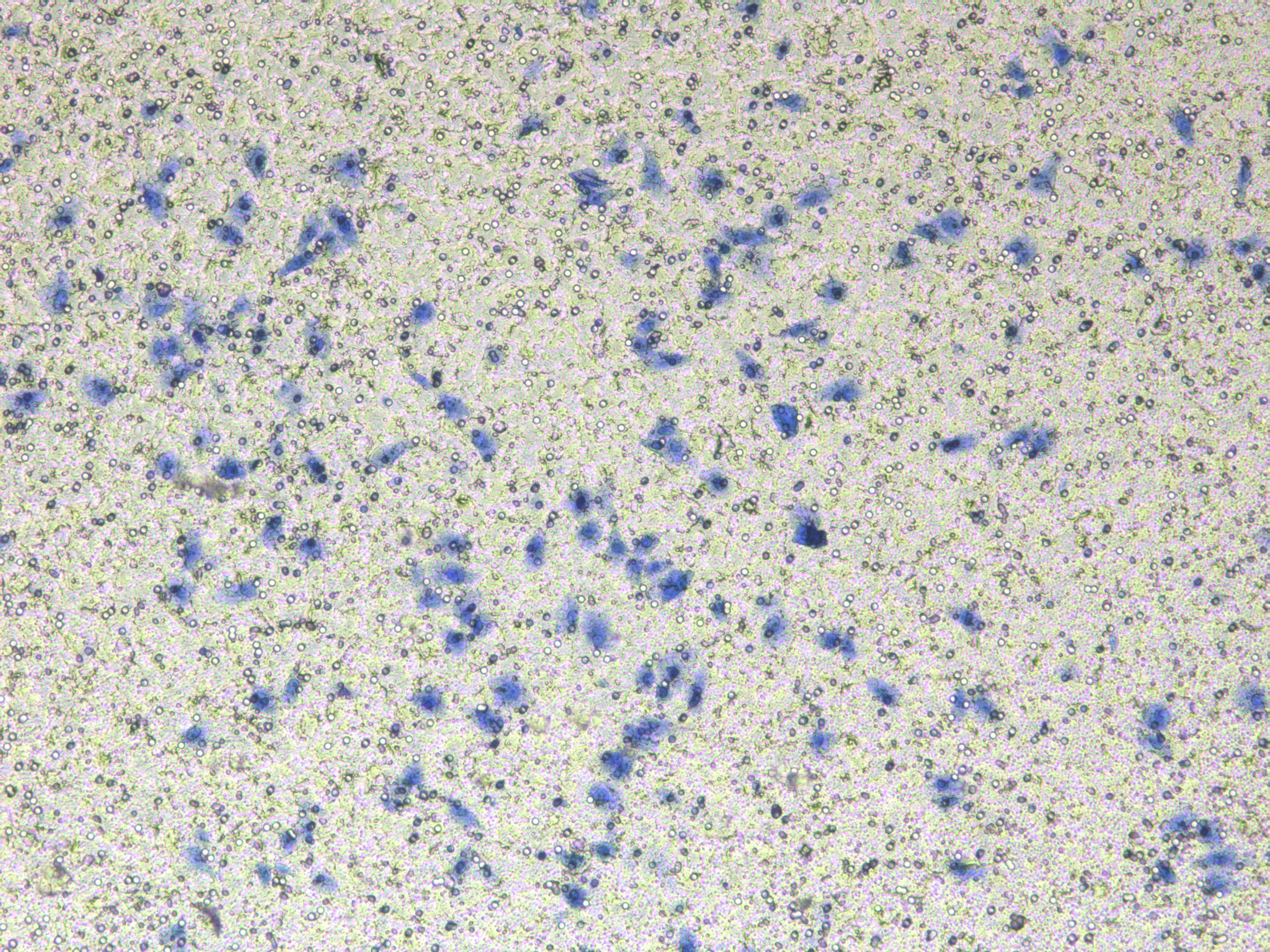

Supplement: S9 File — (ZIP) [file pone.0334639.s009.zip › S 14. File. Original Images. Fig7/S 14. File. Original FIgures. Fig.7/7d/SMMC-7721/mtor/7721 cxcl3 2ngml-.jpg]

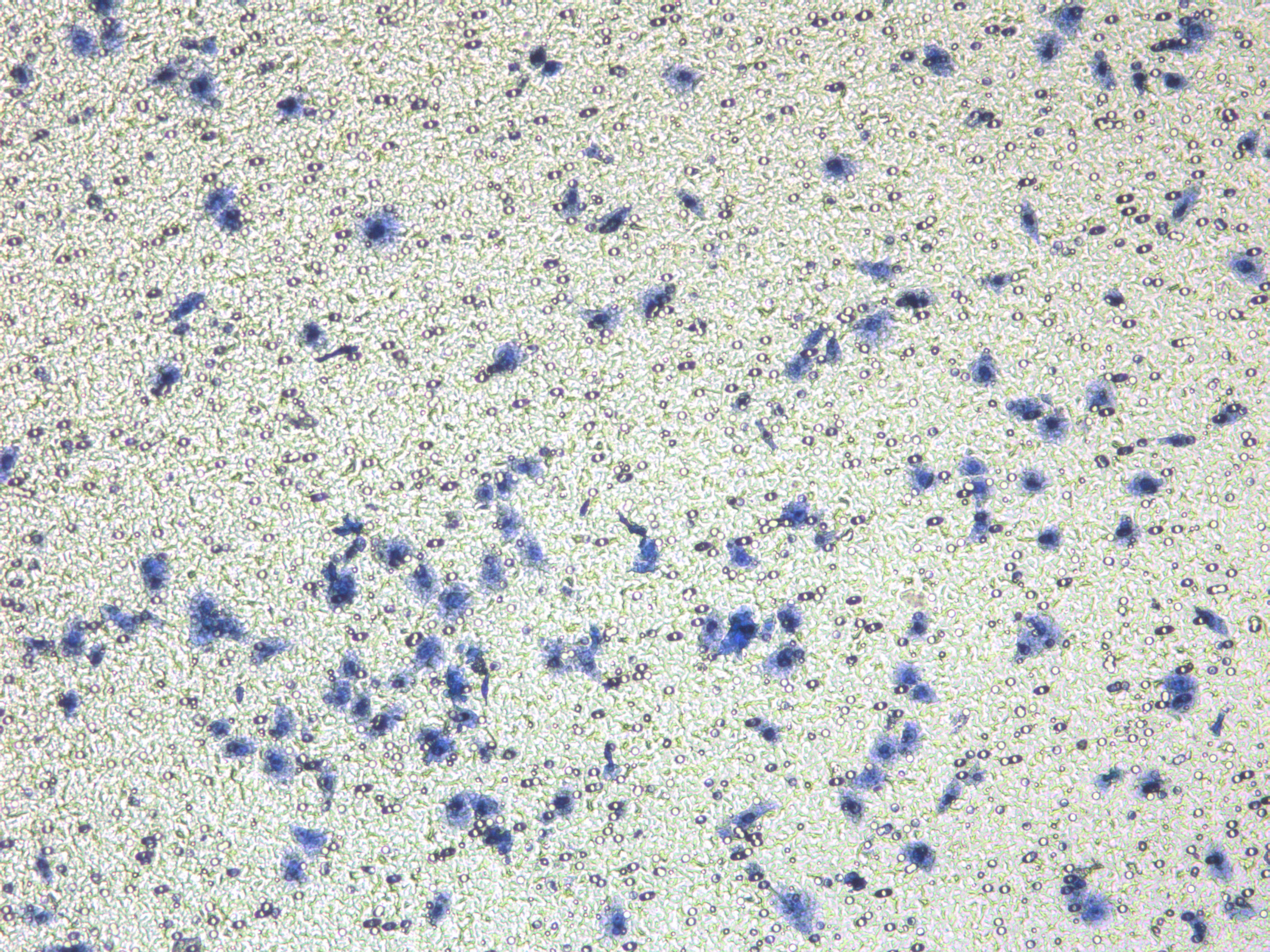

Supplement: S9 File — (ZIP) [file pone.0334639.s009.zip › S 14. File. Original Images. Fig7/S 14. File. Original FIgures. Fig.7/7d/SMMC-7721/mtor/7721 cxcl3 0ngml-.jpg]

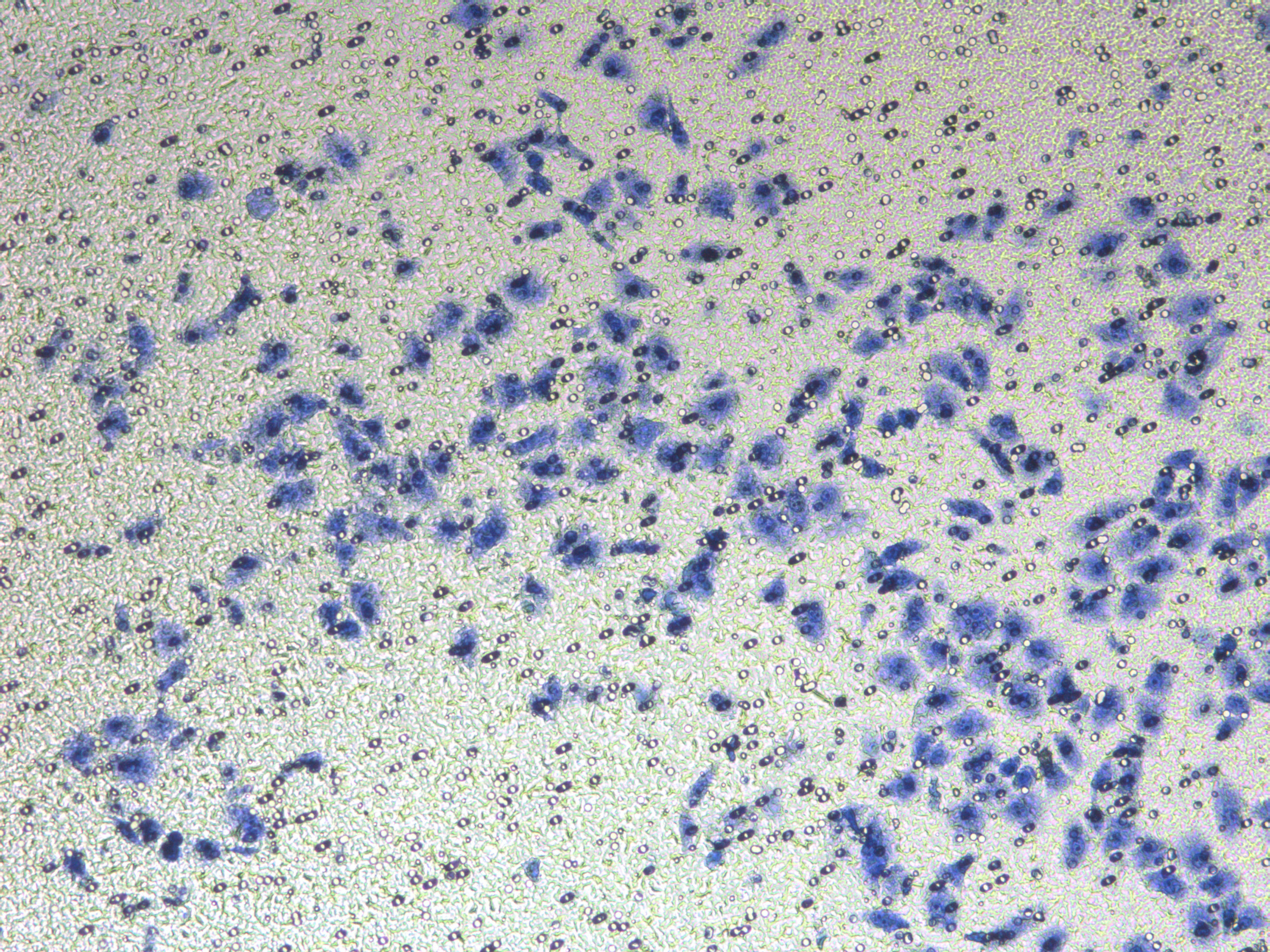

Supplement: S9 File — (ZIP) [file pone.0334639.s009.zip › S 14. File. Original Images. Fig7/S 14. File. Original FIgures. Fig.7/7d/SMMC-7721/mtor/7721 cxcl3 10ngml.jpg]

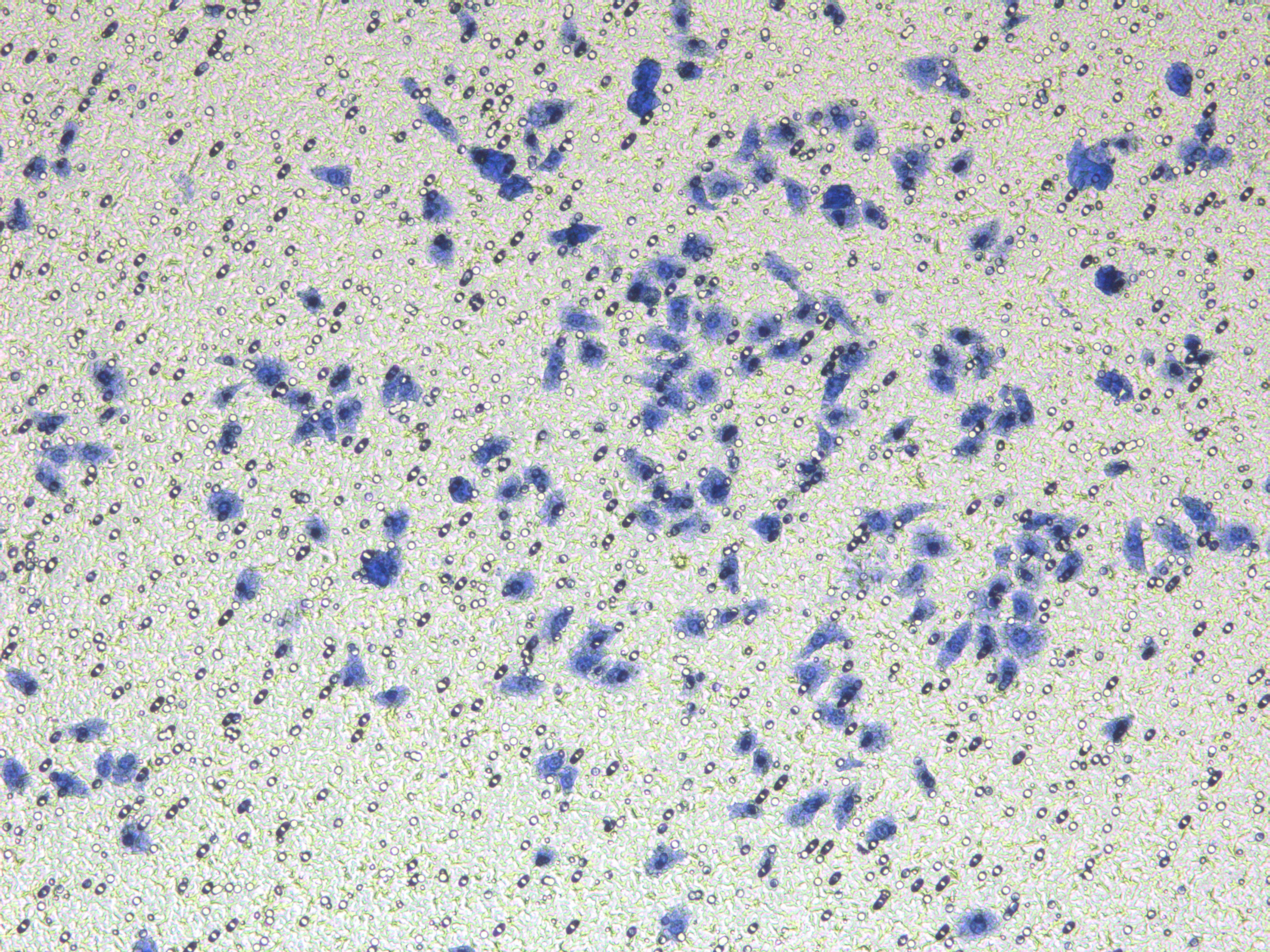

Supplement: S9 File — (ZIP) [file pone.0334639.s009.zip › S 14. File. Original Images. Fig7/S 14. File. Original FIgures. Fig.7/7d/SMMC-7721/mtor/7721 cxcl3 20ngml-.jpg]

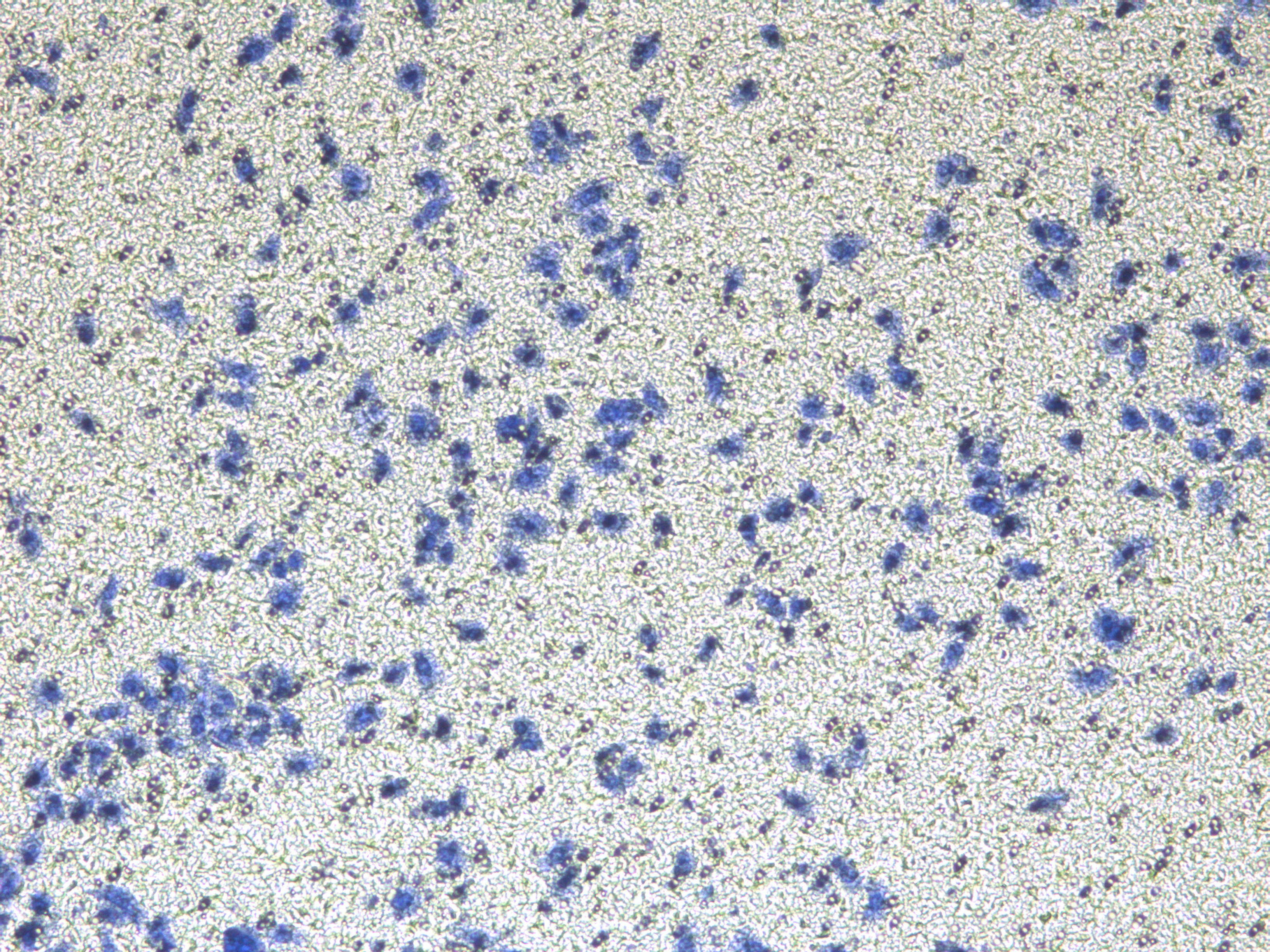

Supplement: S9 File — (ZIP) [file pone.0334639.s009.zip › S 14. File. Original Images. Fig7/S 14. File. Original FIgures. Fig.7/7d/SMMC-7721/mtor/7721 cxcl3 30ngml-.jpg]

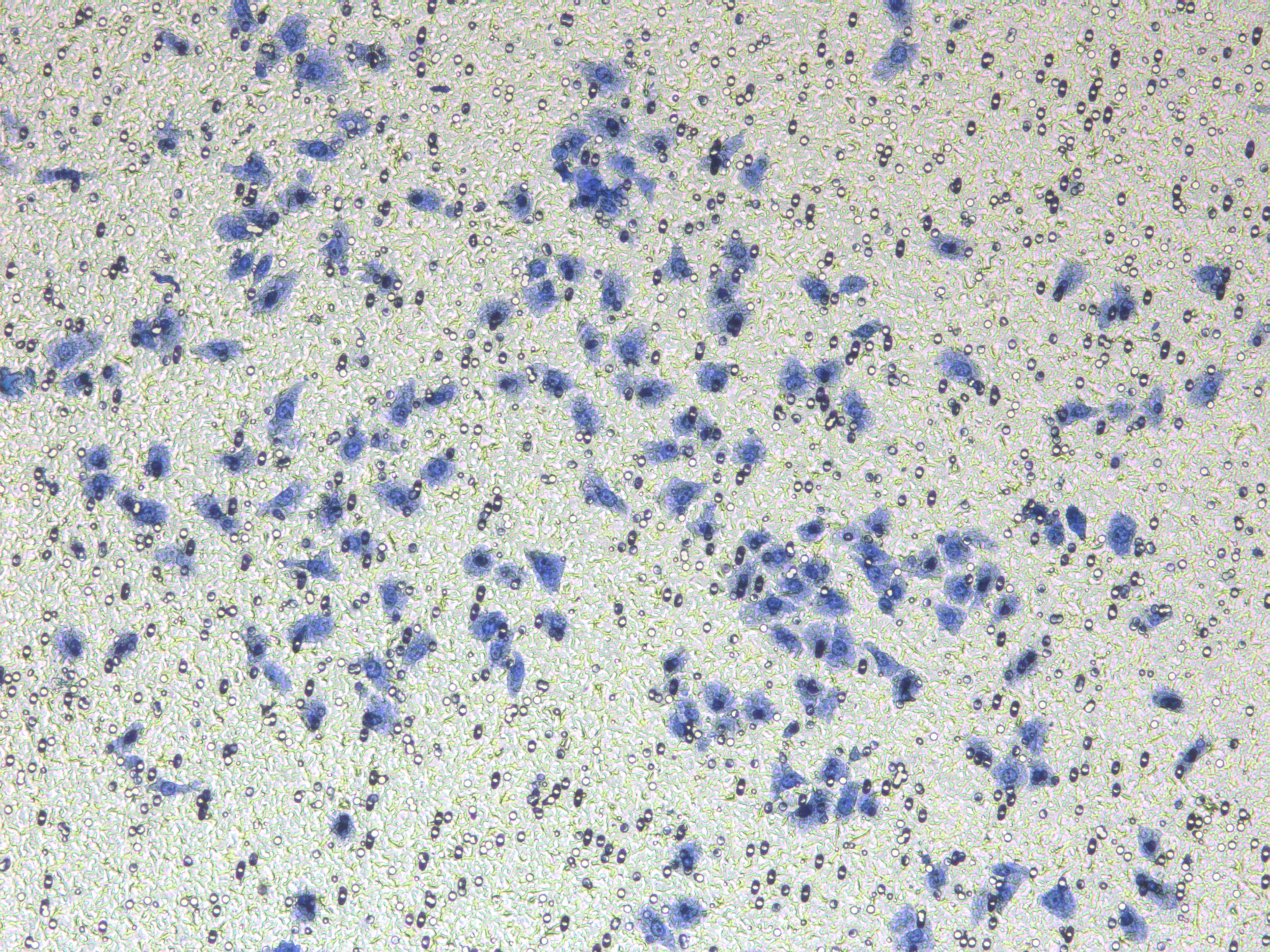

Supplement: S9 File — (ZIP) [file pone.0334639.s009.zip › S 14. File. Original Images. Fig7/S 14. File. Original FIgures. Fig.7/7d/SMMC-7721/mtor/7721 cxcl3 5ngml.jpg]

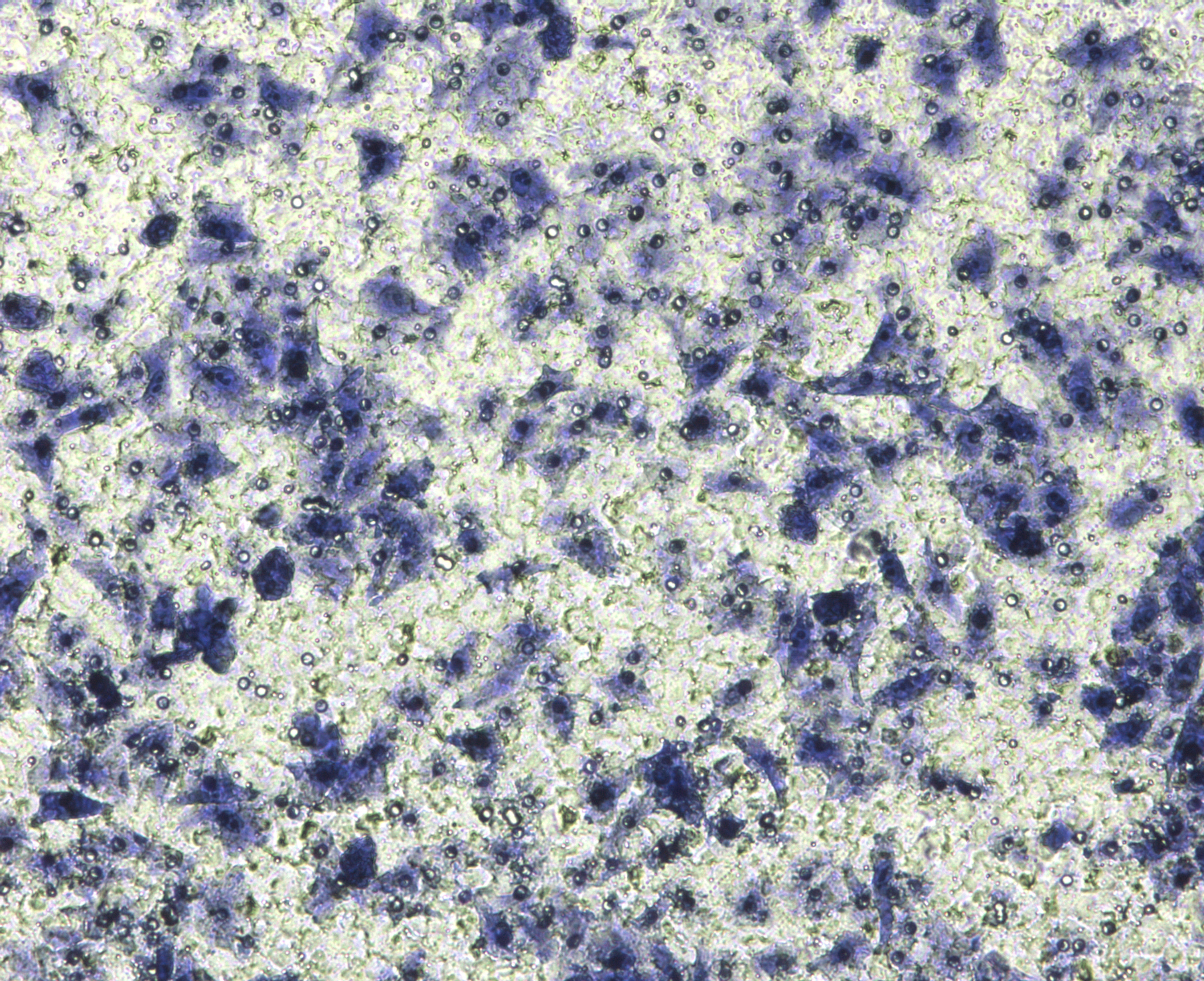

Supplement: S10 File — (ZIP) [file pone.0334639.s010.zip › S 15. File. Original FIgures. Fig.8/8d/Bel 7402-A+DMSO.jpg]

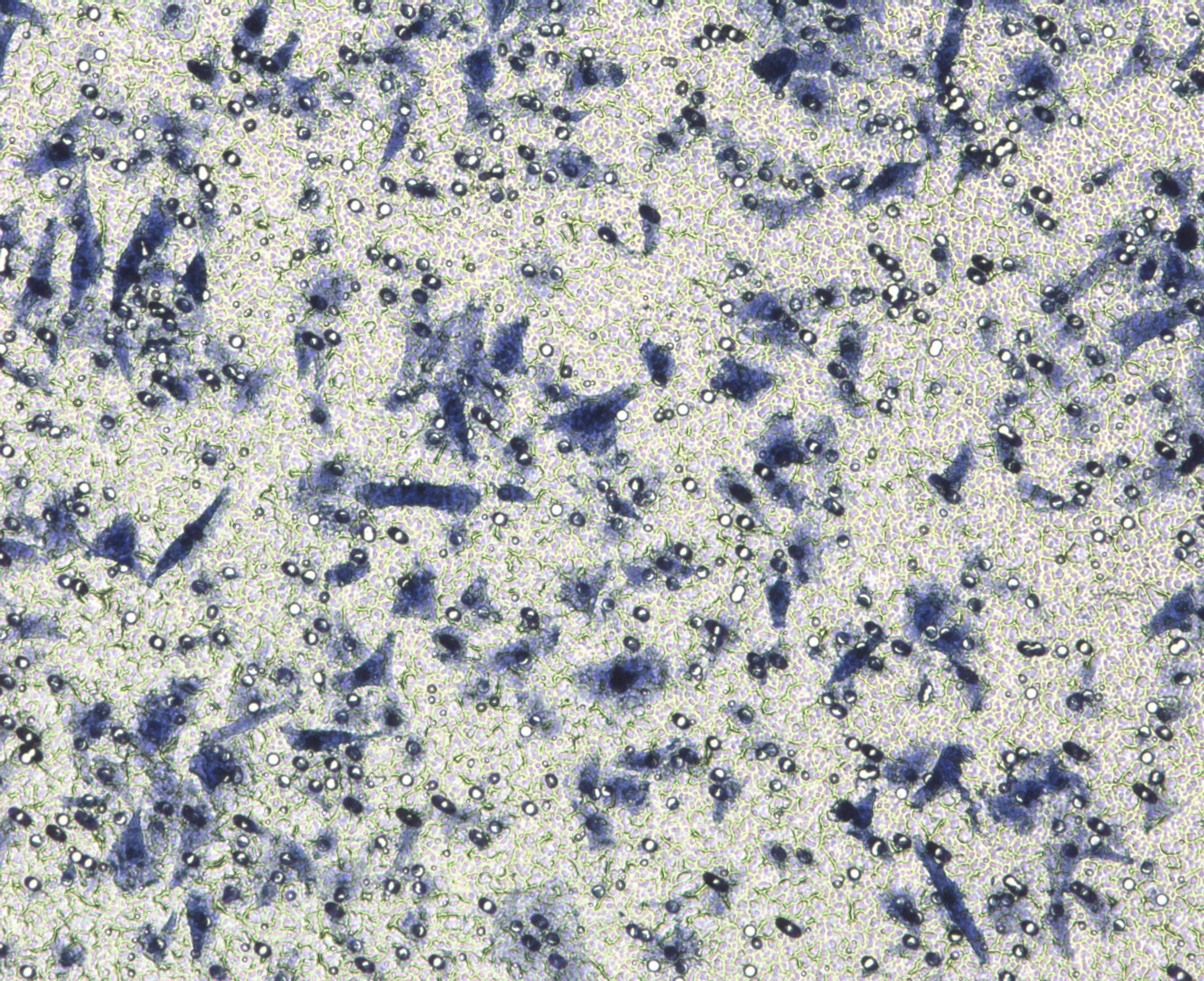

Supplement: S10 File — (ZIP) [file pone.0334639.s010.zip › S 15. File. Original FIgures. Fig.8/8d/Bel 7402-N+DMSO--135.jpg]

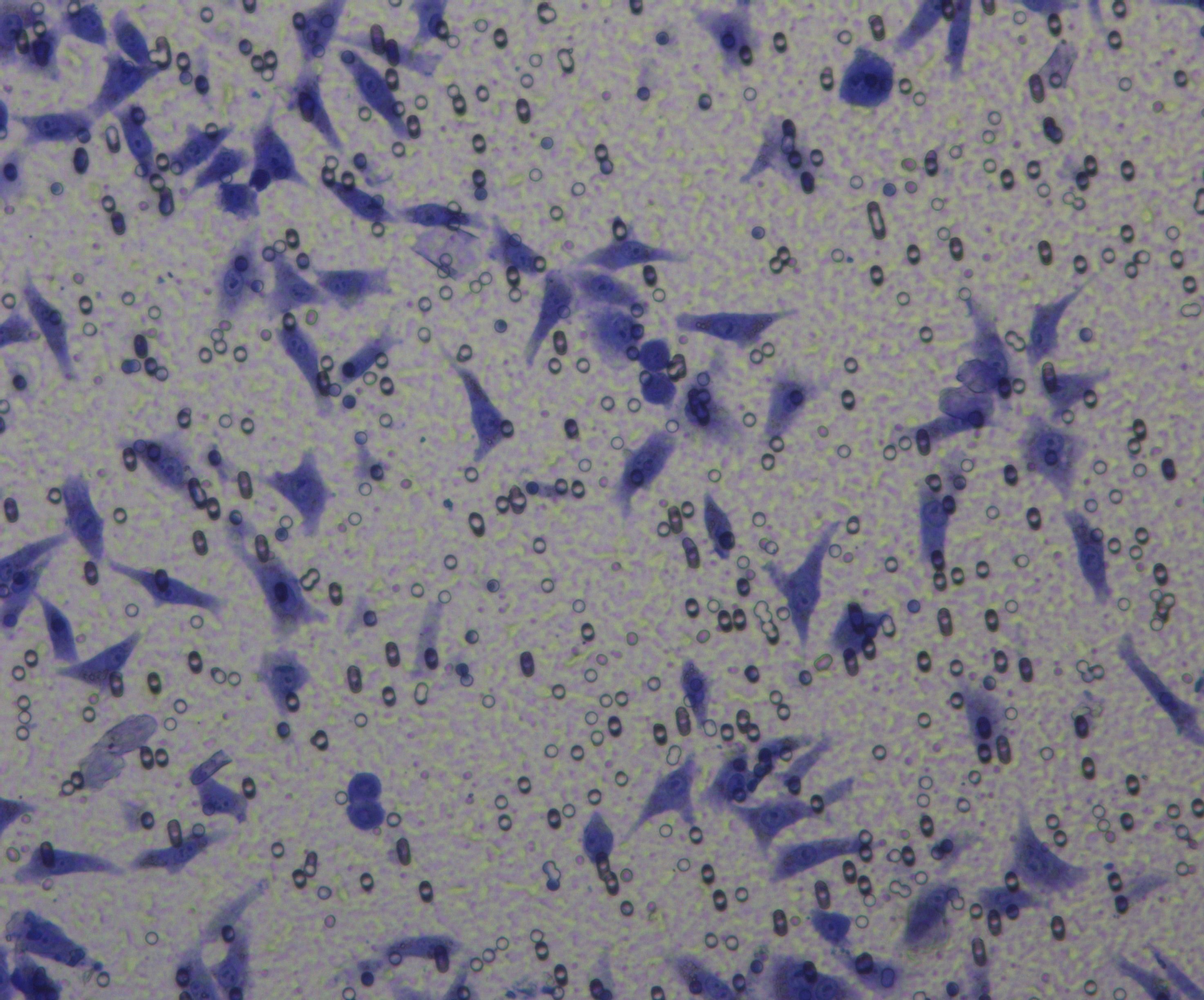

Supplement: S10 File — (ZIP) [file pone.0334639.s010.zip › S 15. File. Original FIgures. Fig.8/8d/Bel cxcl3 A+Mtor.jpg]

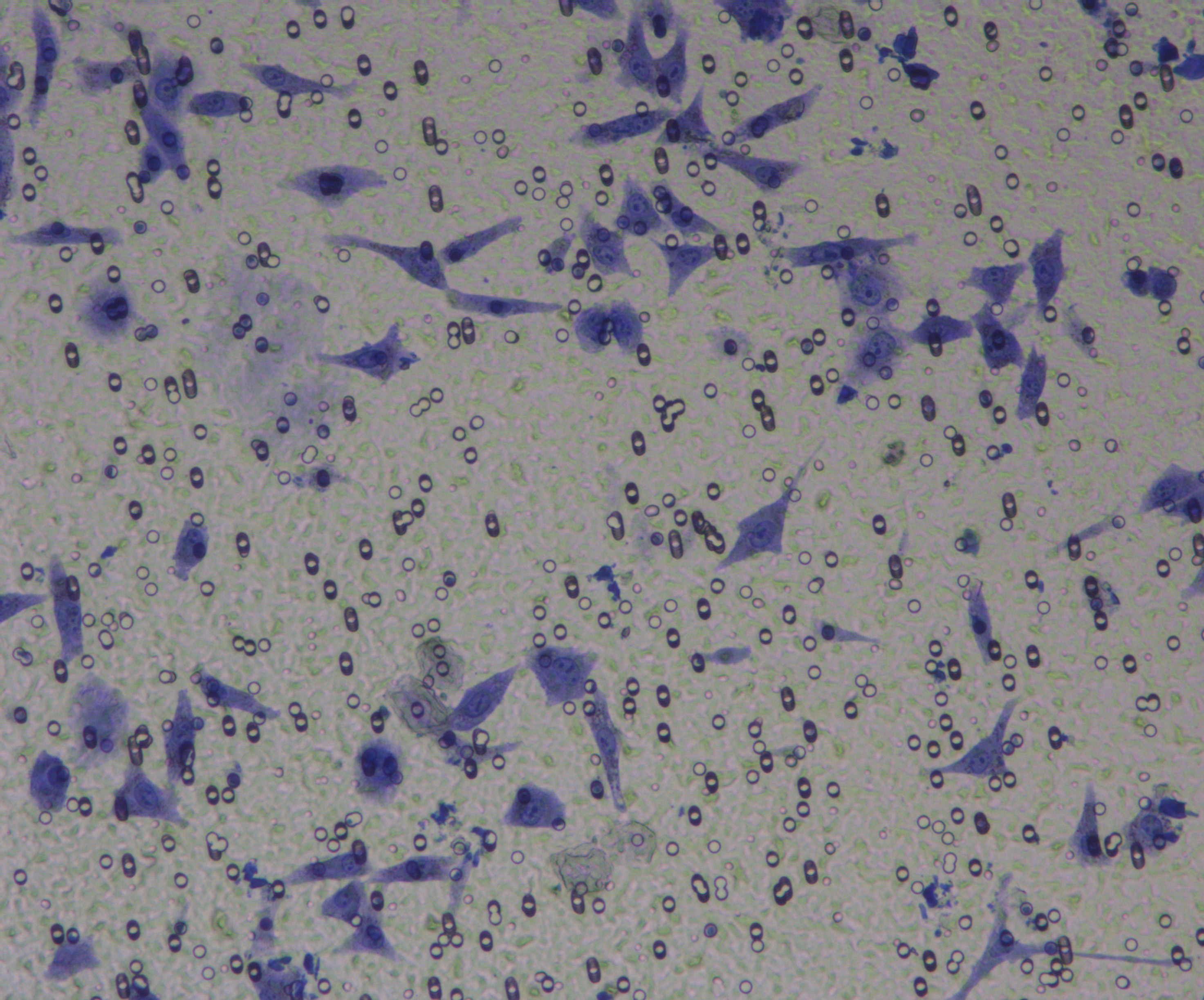

Supplement: S10 File — (ZIP) [file pone.0334639.s010.zip › S 15. File. Original FIgures. Fig.8/8d/Bel cxcl3 N+Mtor.jpg]

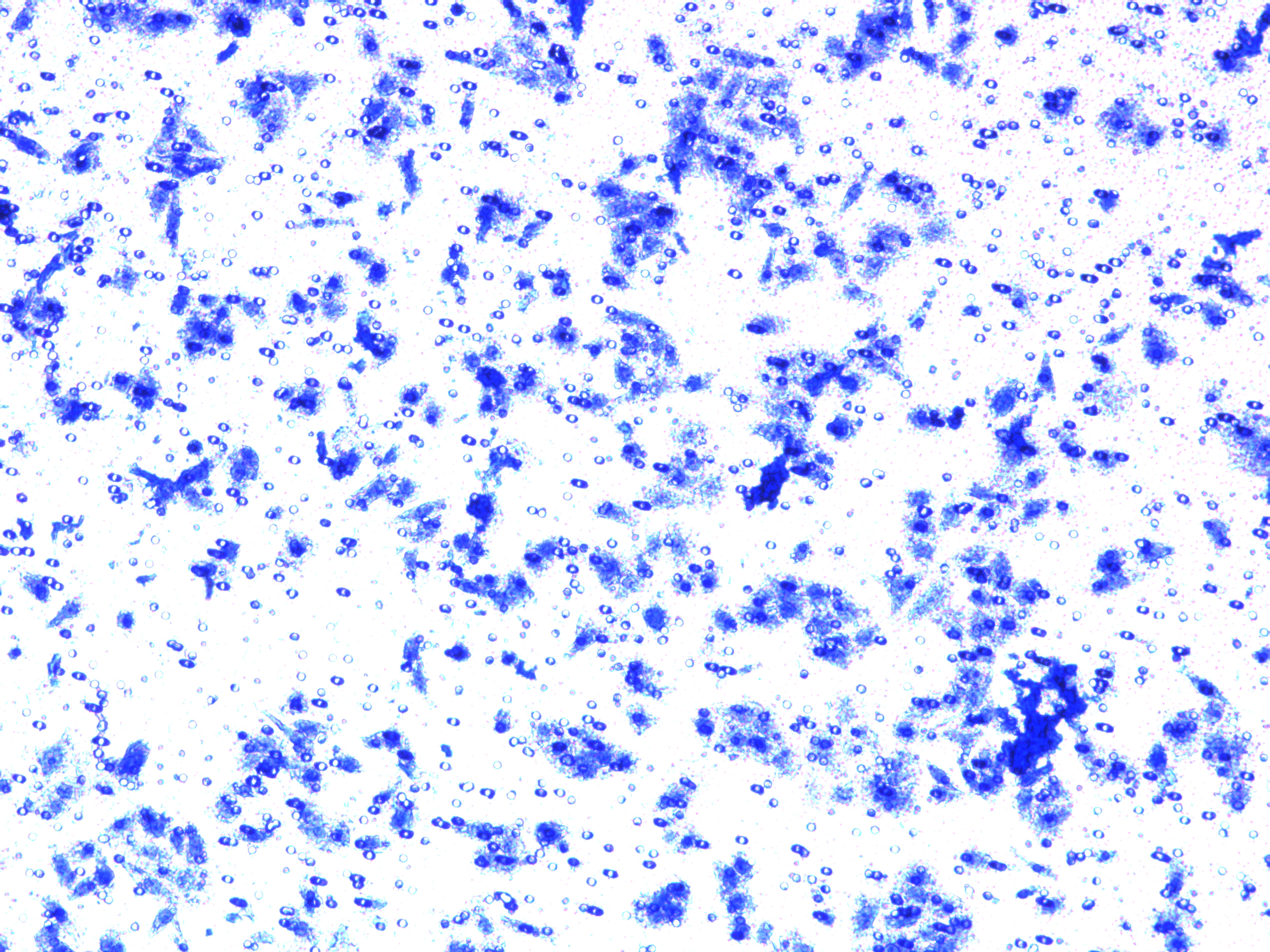

Supplement: S10 File — (ZIP) [file pone.0334639.s010.zip › S 15. File. Original FIgures. Fig.8/8d/hepG2 A+DMSO.jpg]

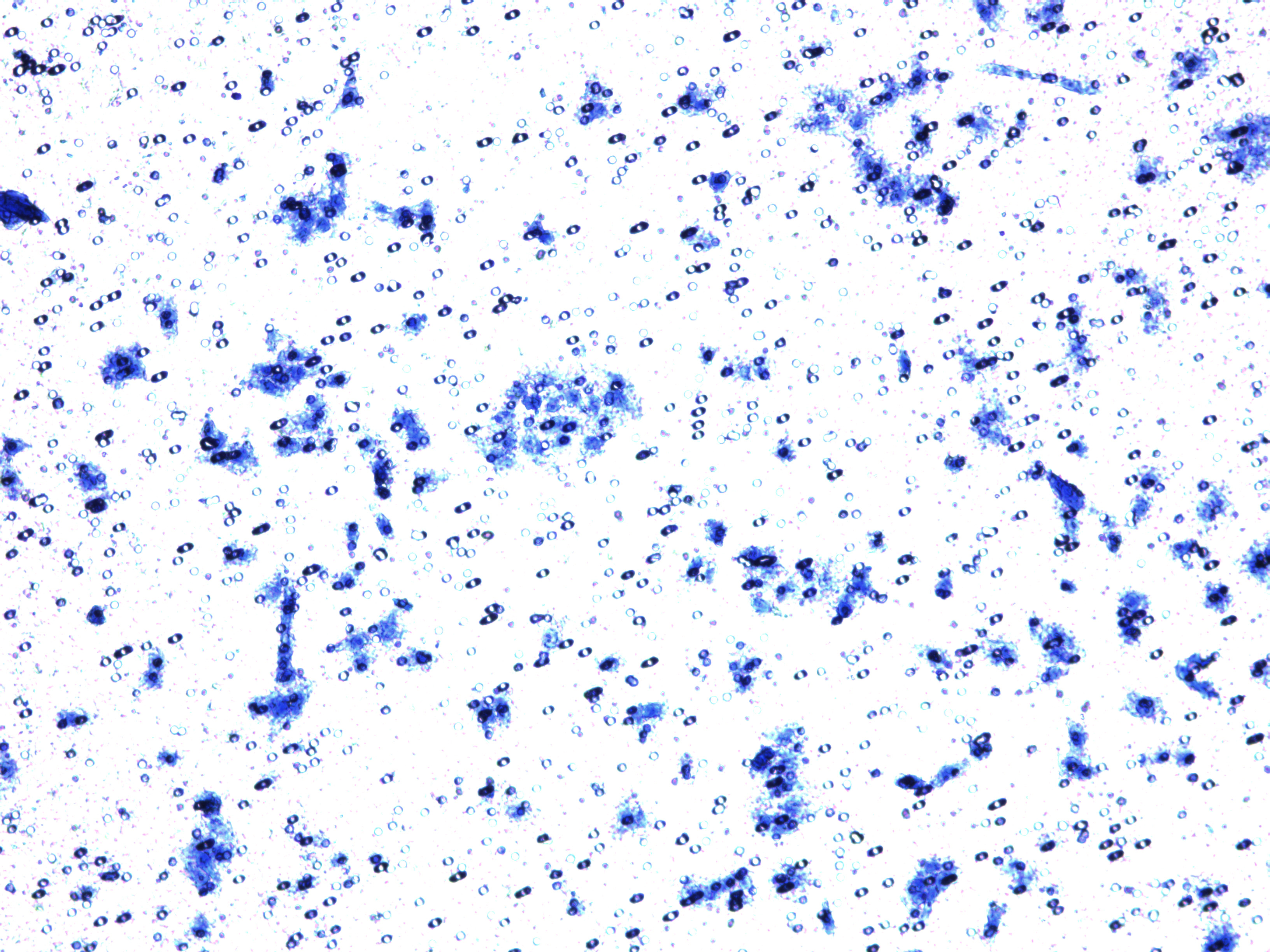

Supplement: S10 File — (ZIP) [file pone.0334639.s010.zip › S 15. File. Original FIgures. Fig.8/8d/hepG2 A+MTOR.jpg]

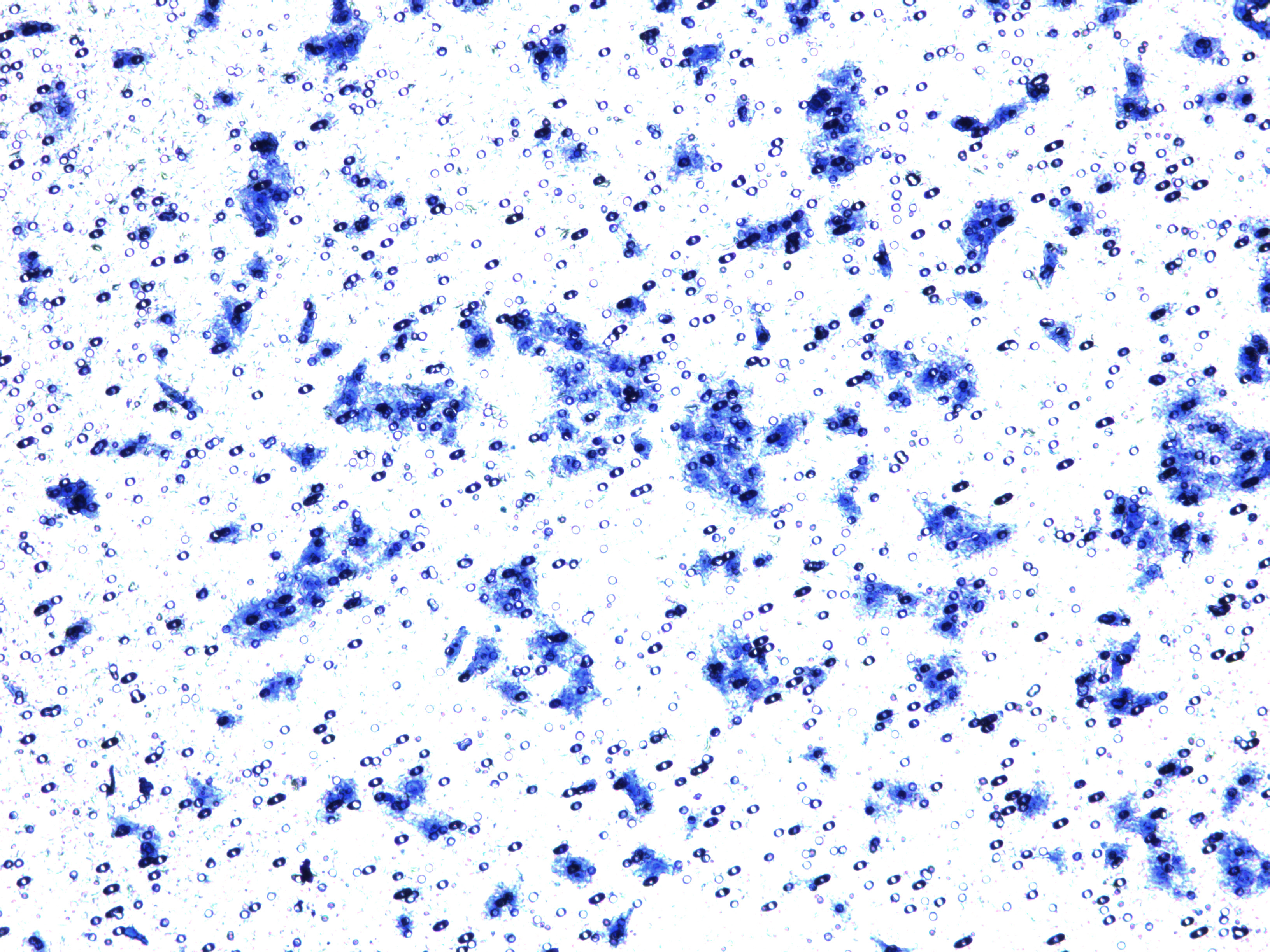

Supplement: S10 File — (ZIP) [file pone.0334639.s010.zip › S 15. File. Original FIgures. Fig.8/8d/hepG2 N+DMSO.jpg]

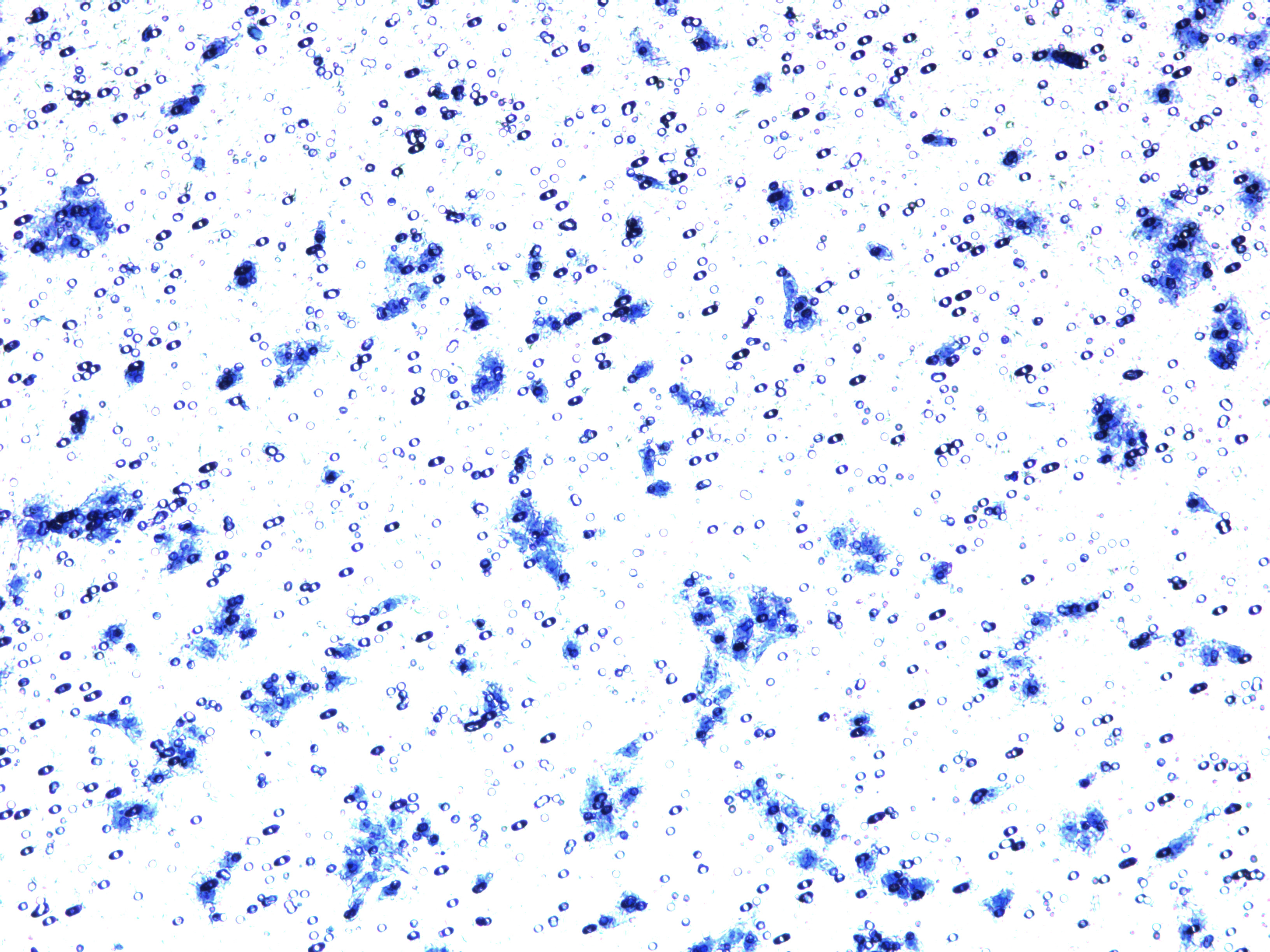

Supplement: S10 File — (ZIP) [file pone.0334639.s010.zip › S 15. File. Original FIgures. Fig.8/8d/hepG2N+MTOR.jpg]

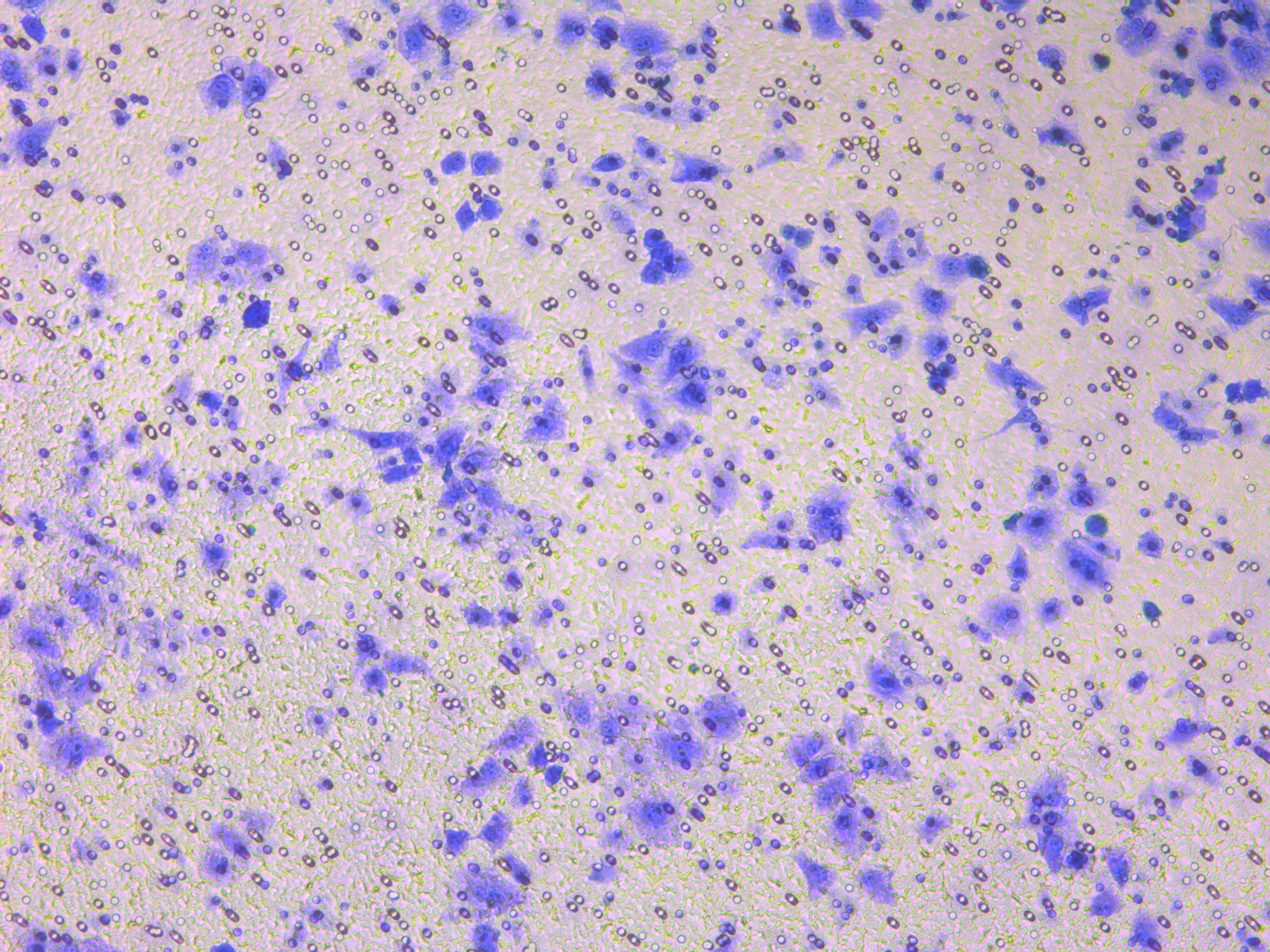

Supplement: S10 File — (ZIP) [file pone.0334639.s010.zip › S 15. File. Original FIgures. Fig.8/8d/SMMC-7721 cxcl3 N+DMSO.jpg]

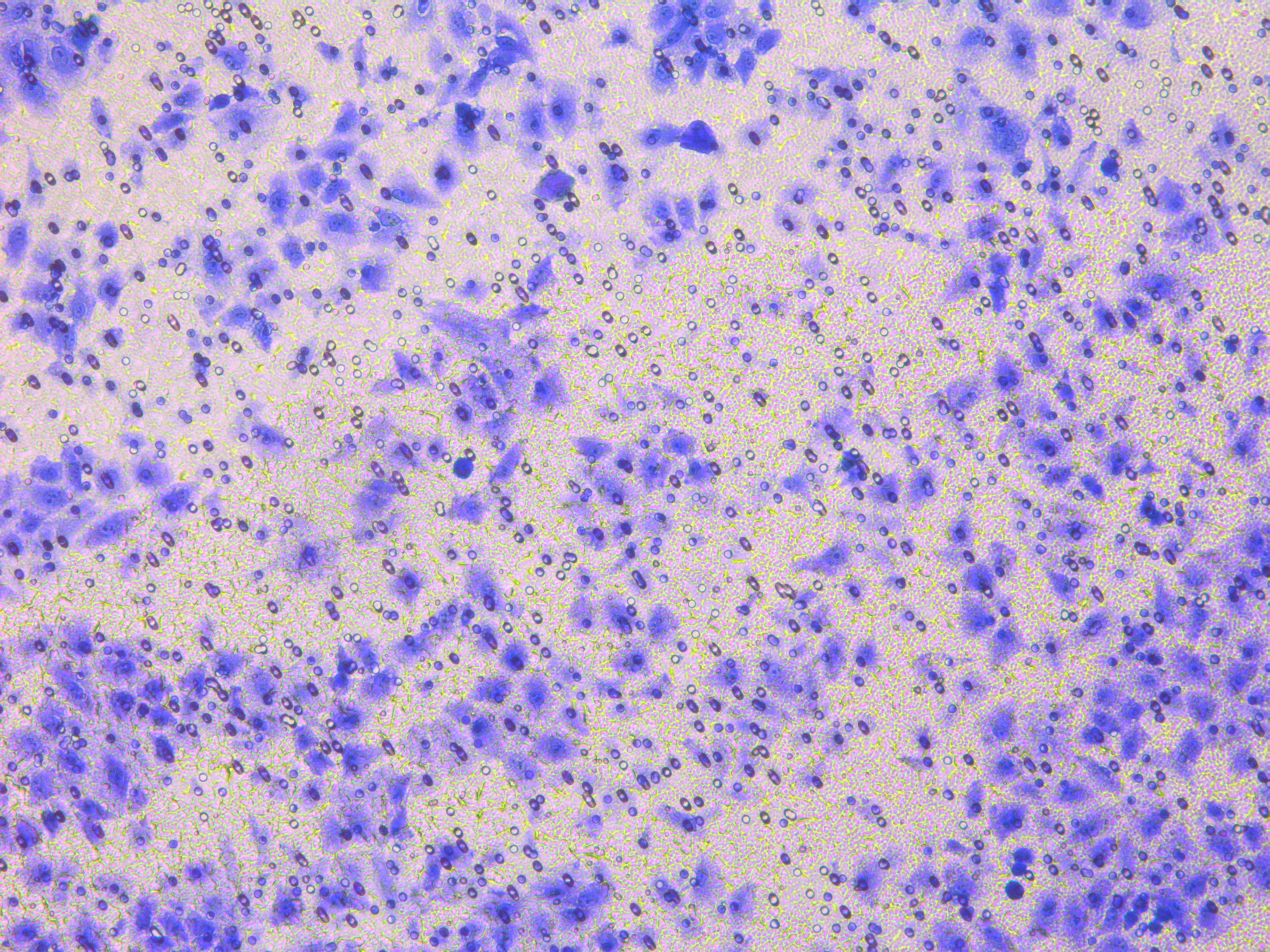

Supplement: S10 File — (ZIP) [file pone.0334639.s010.zip › S 15. File. Original FIgures. Fig.8/8d/SMMC-7721 cxcl3 A+DMSO .jpg]

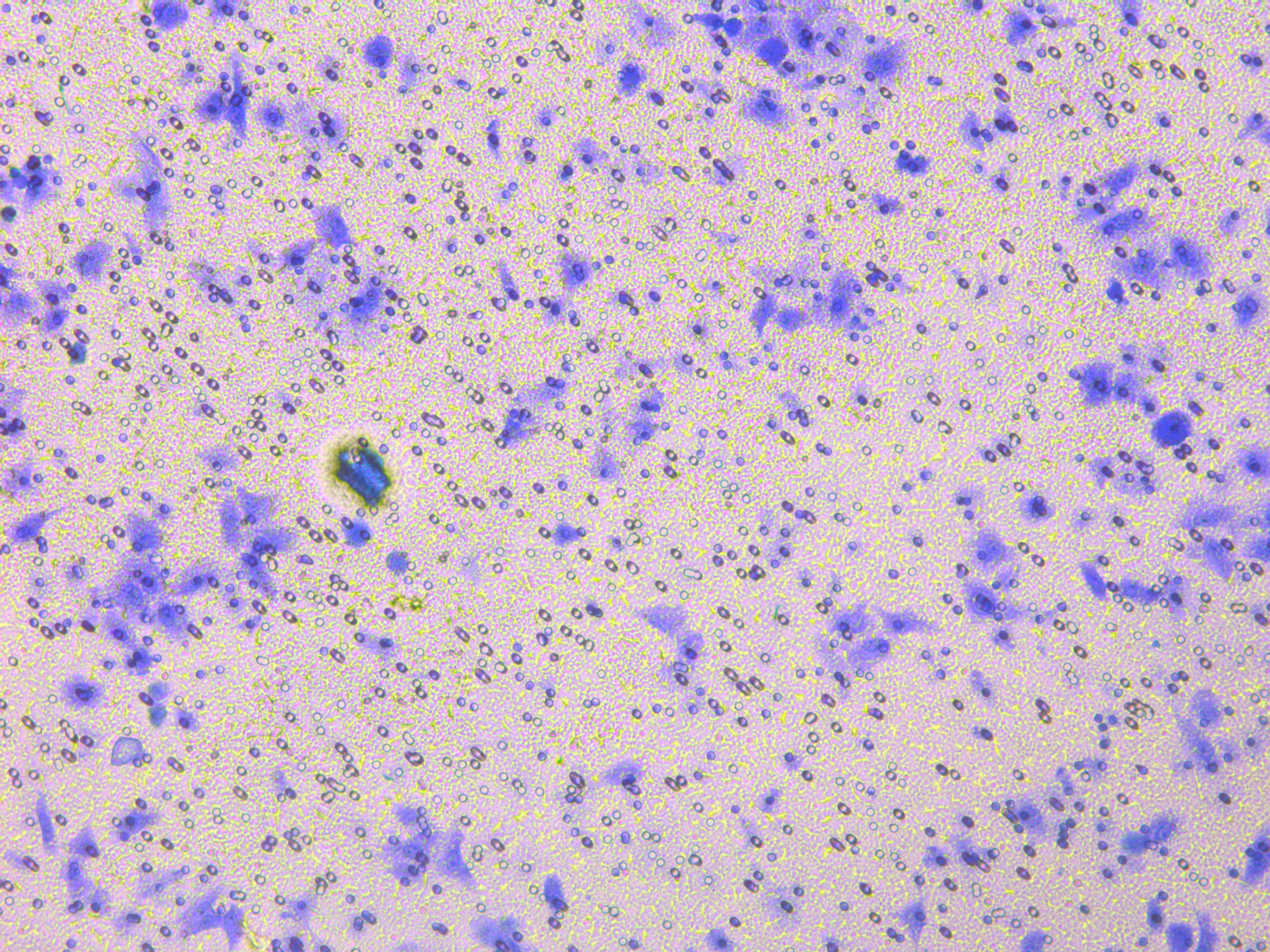

Supplement: S10 File — (ZIP) [file pone.0334639.s010.zip › S 15. File. Original FIgures. Fig.8/8d/SMMC-7721 cxcl3 N+MTOR.jpg]

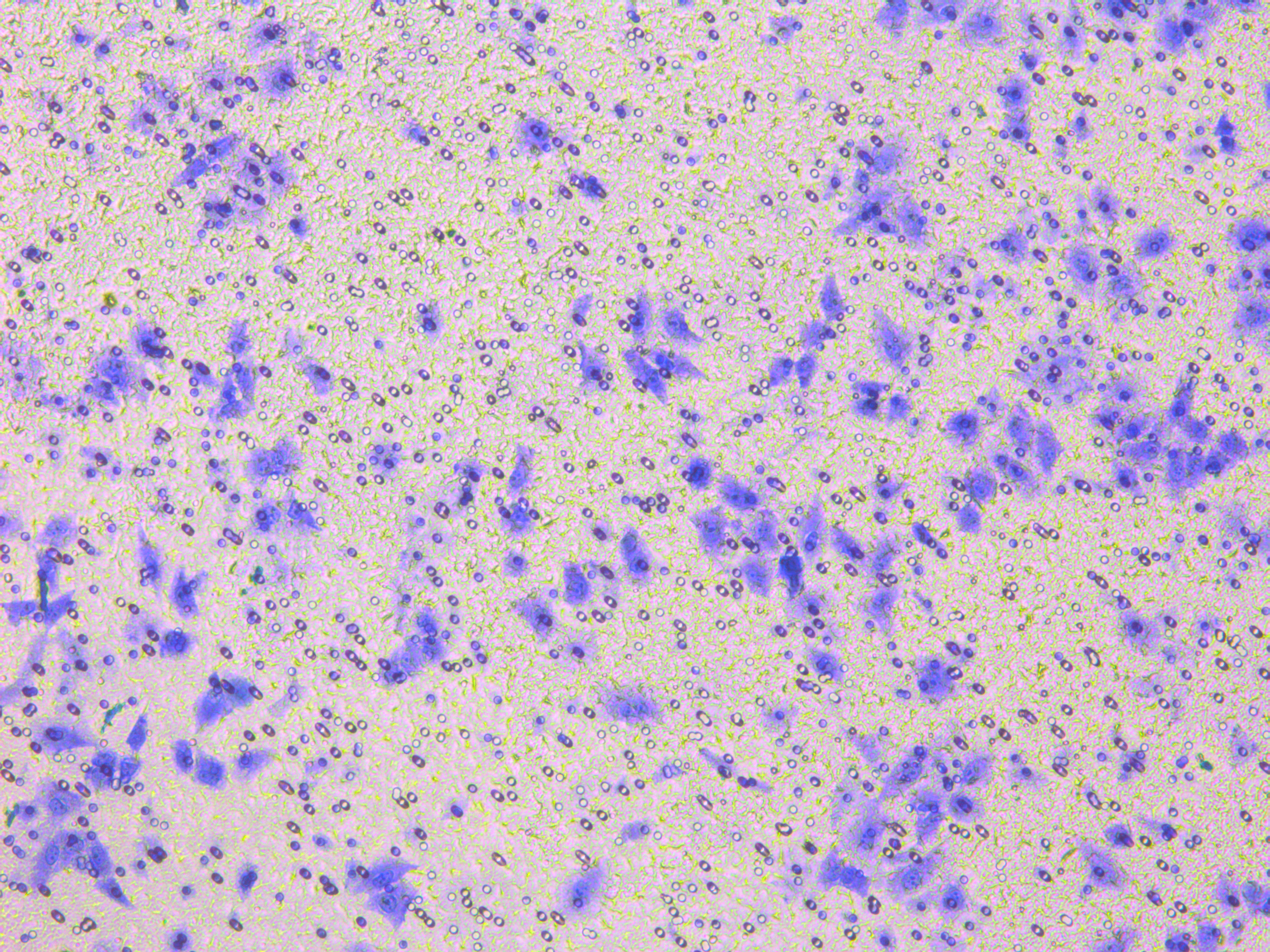

Supplement: S10 File — (ZIP) [file pone.0334639.s010.zip › S 15. File. Original FIgures. Fig.8/8d/SMMC-7721 cxcl3A+MTOR.jpg]

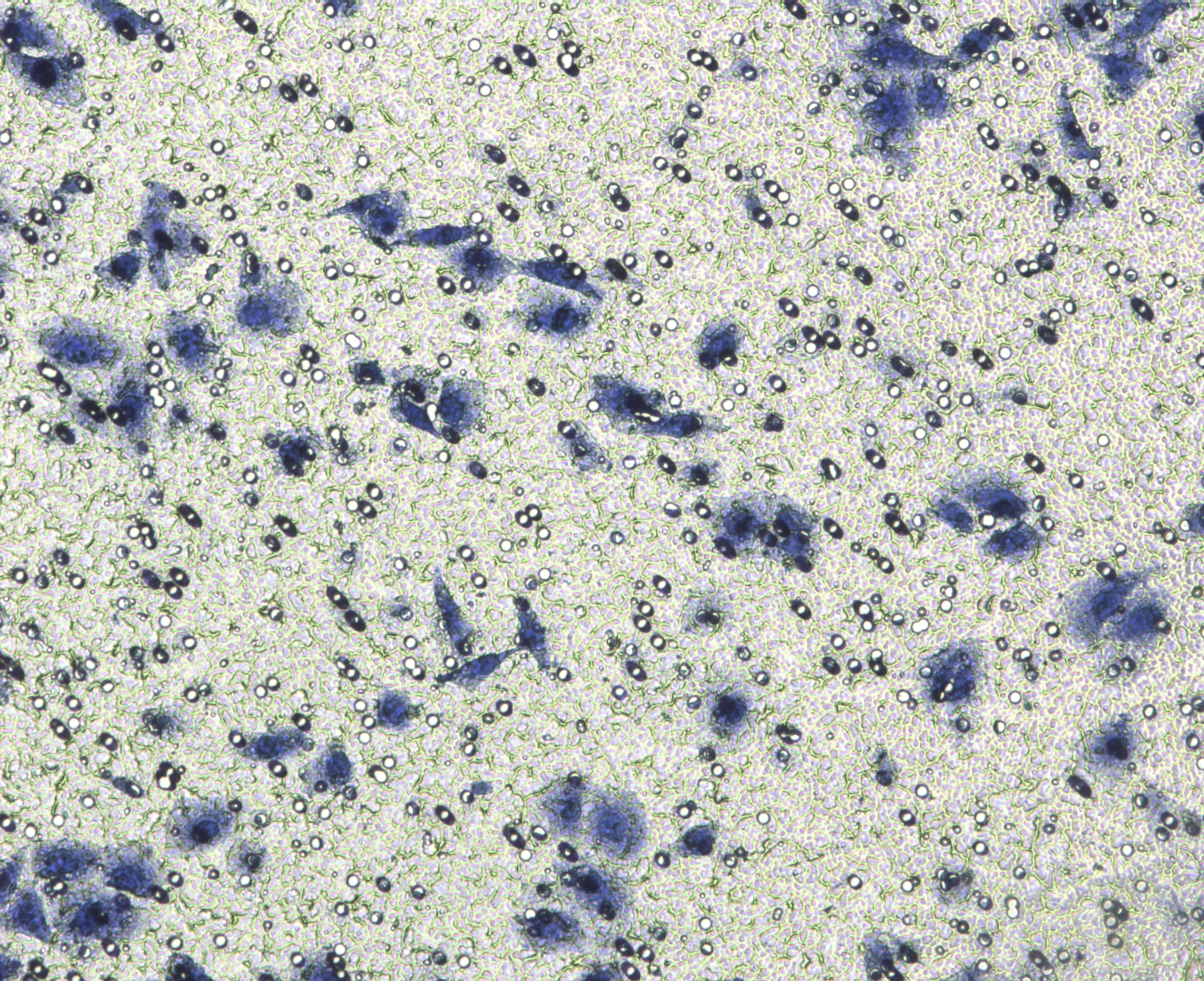

Supplement: S11 File — (ZIP) [file pone.0334639.s011.zip › S 16. File. Original FIgures. Fig.9/9d/BEL-7402 sh-CXCL3+MTOR.jpg]

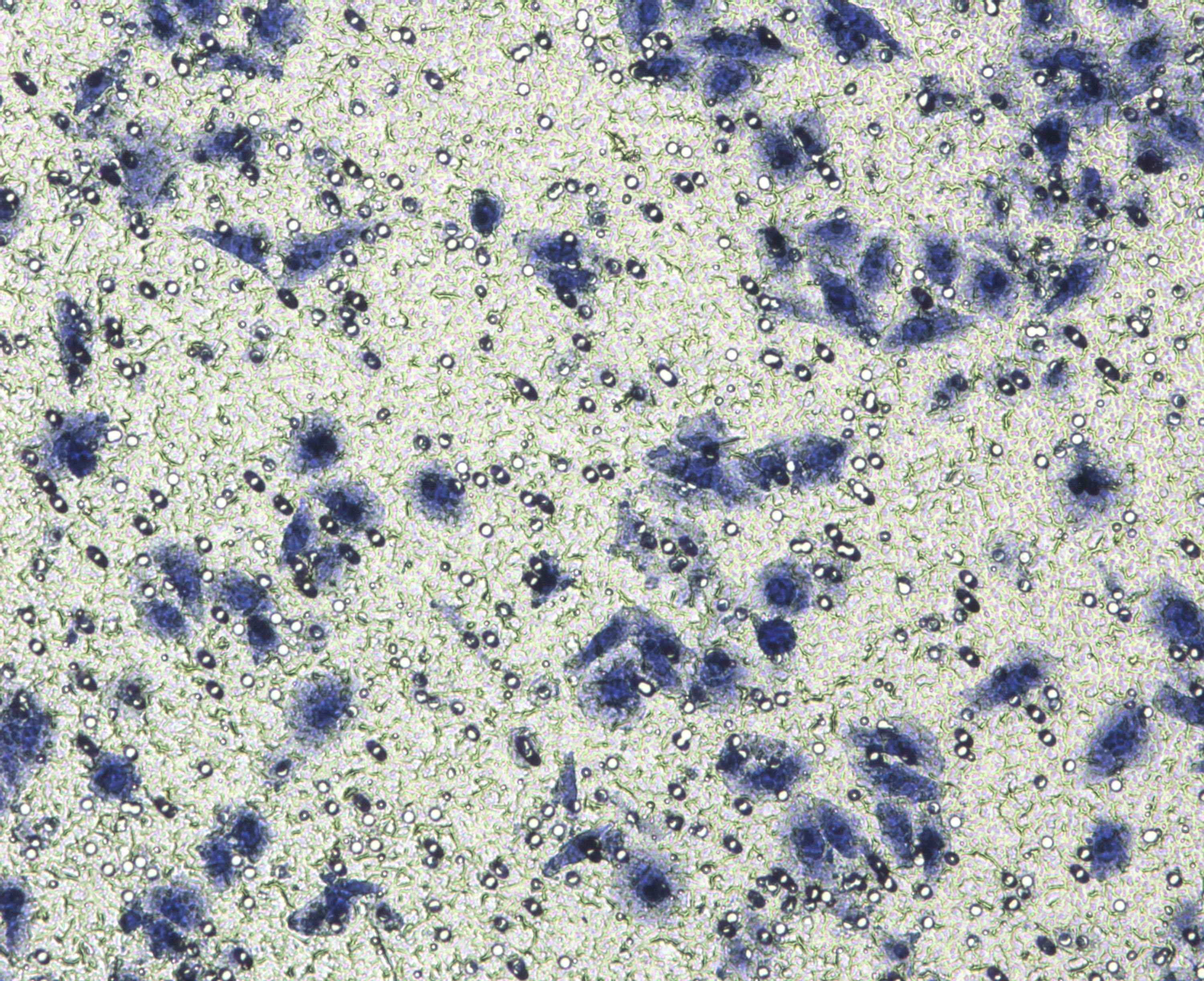

Supplement: S11 File — (ZIP) [file pone.0334639.s011.zip › S 16. File. Original FIgures. Fig.9/9d/BEL-7402 sh-NC+MTOR.jpg]

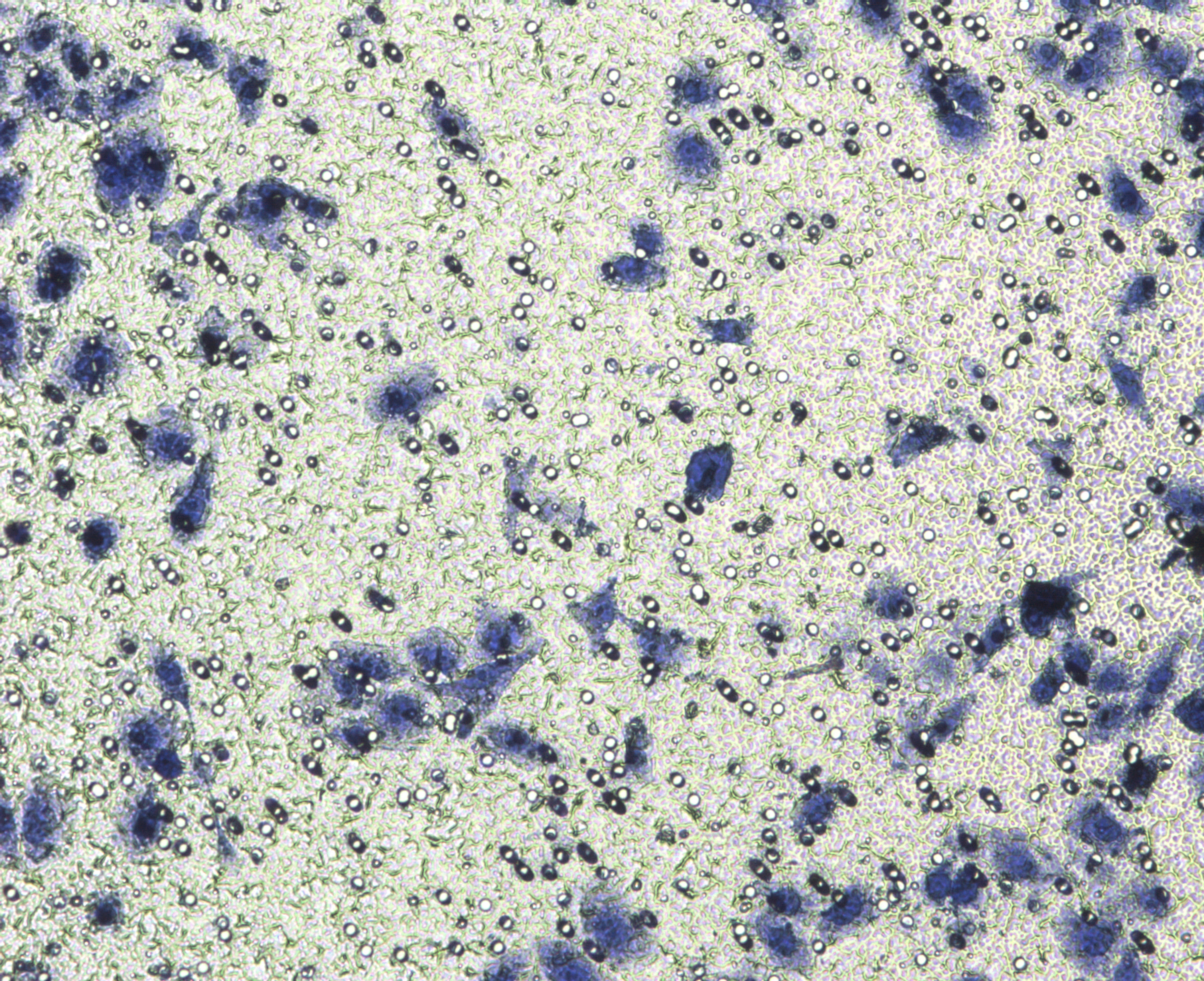

Supplement: S11 File — (ZIP) [file pone.0334639.s011.zip › S 16. File. Original FIgures. Fig.9/9d/Bel7402 shCXCL3 +DMSO.jpg]

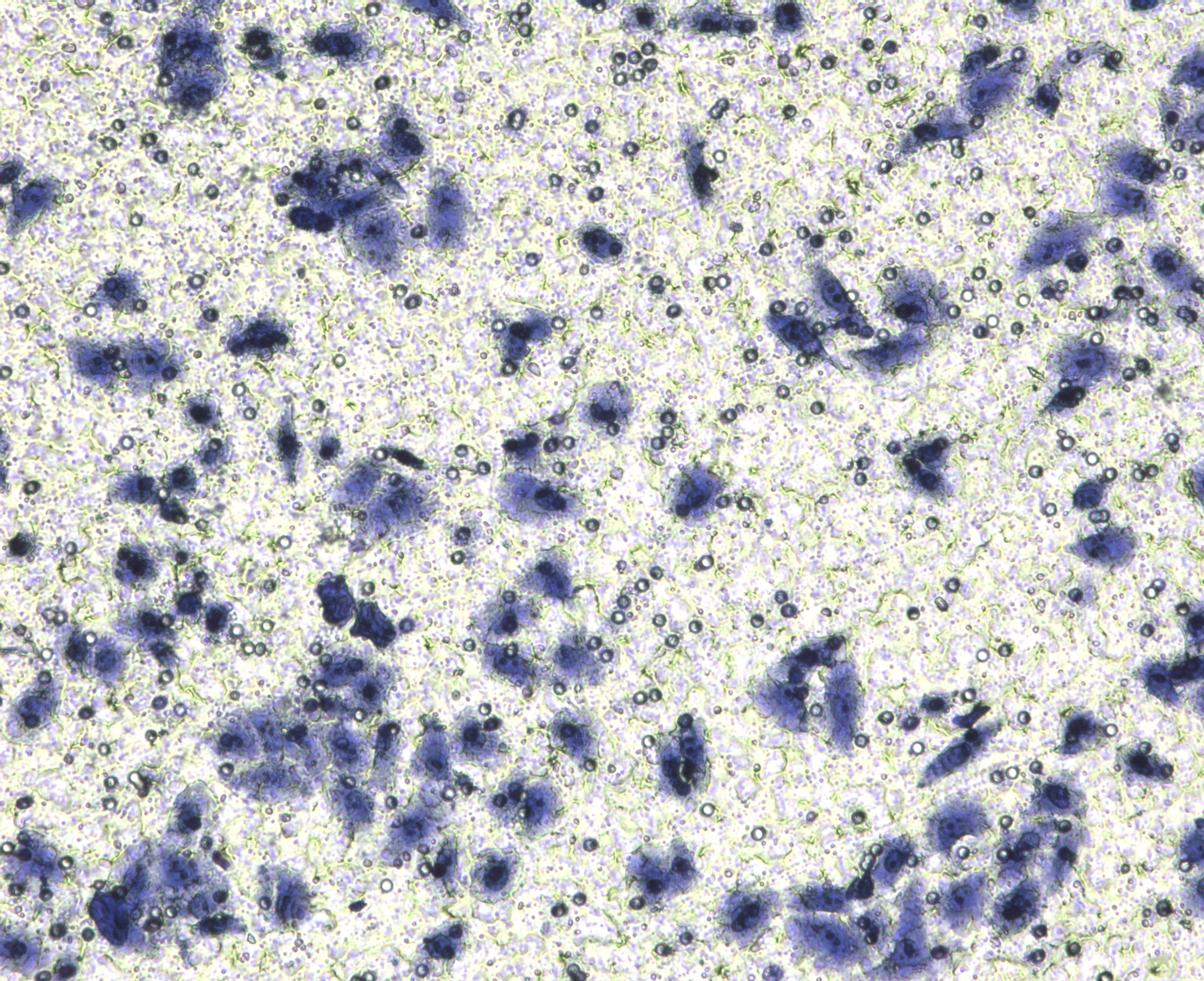

Supplement: S11 File — (ZIP) [file pone.0334639.s011.zip › S 16. File. Original FIgures. Fig.9/9d/BEL7402-sh-NC+DMSO.jpg]

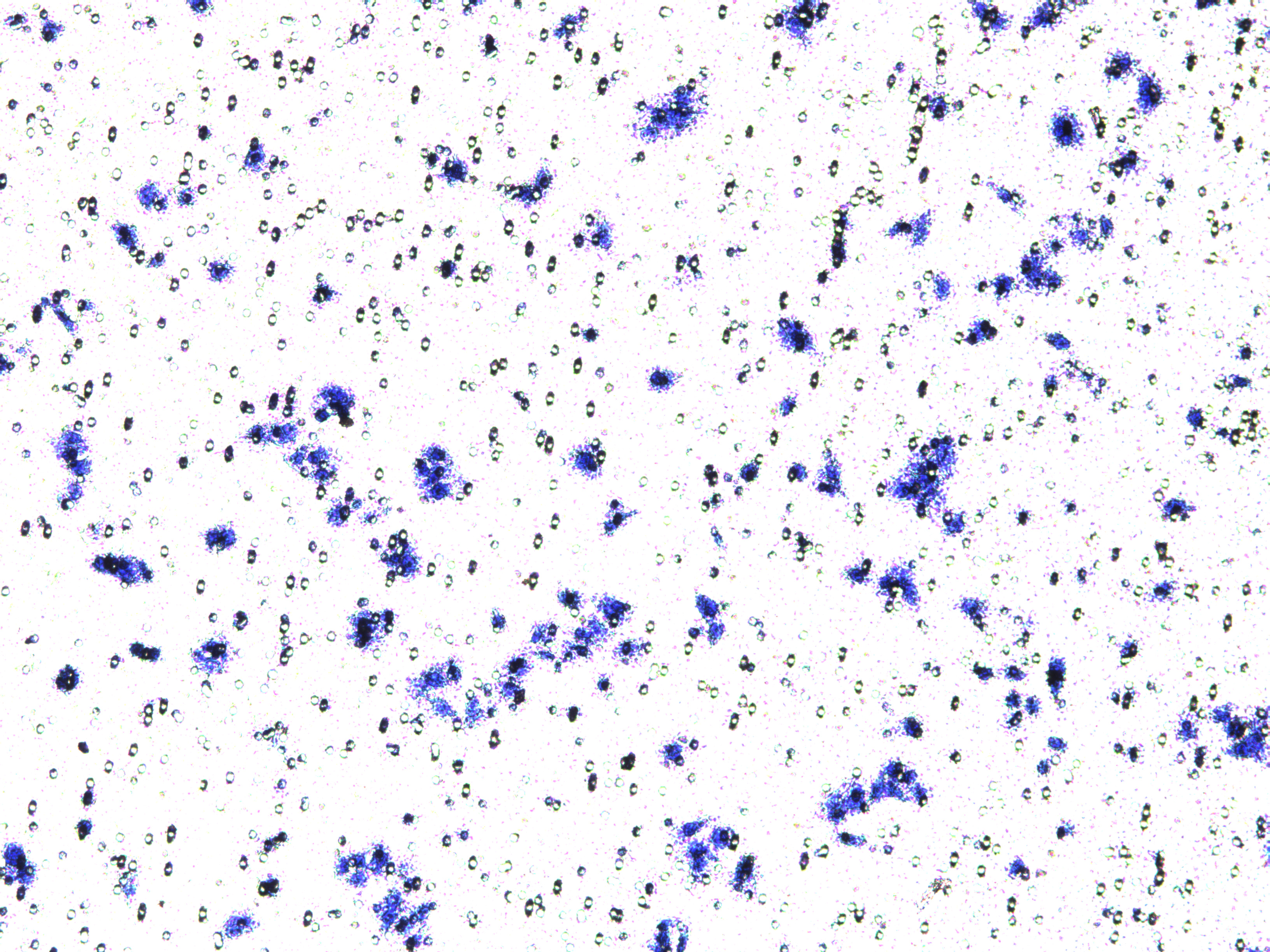

Supplement: S11 File — (ZIP) [file pone.0334639.s011.zip › S 16. File. Original FIgures. Fig.9/9d/hepg2sh-CXCL3+DMSO.jpg]

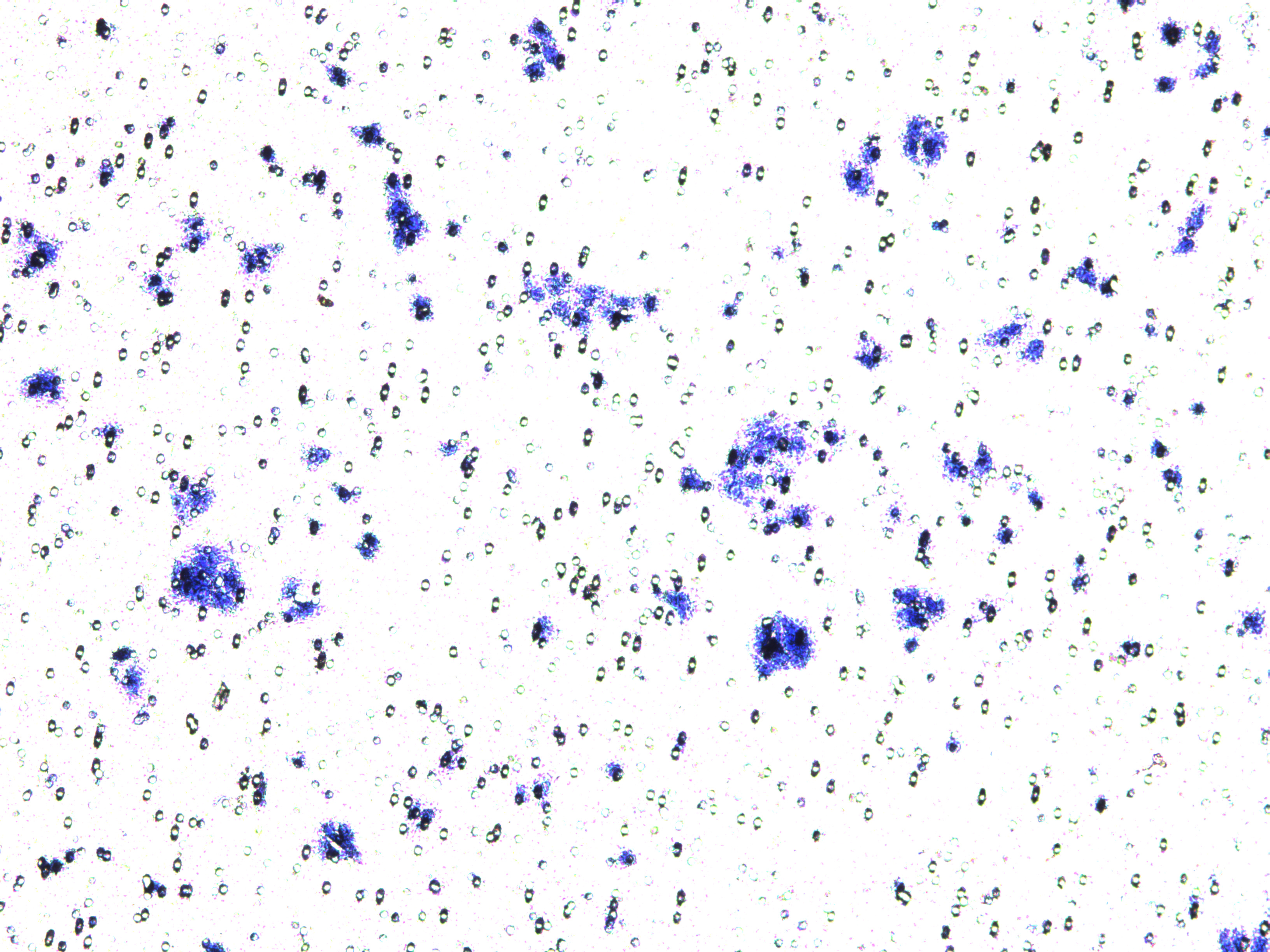

Supplement: S11 File — (ZIP) [file pone.0334639.s011.zip › S 16. File. Original FIgures. Fig.9/9d/hepg2sh-CXCL3+MTOR.jpg]

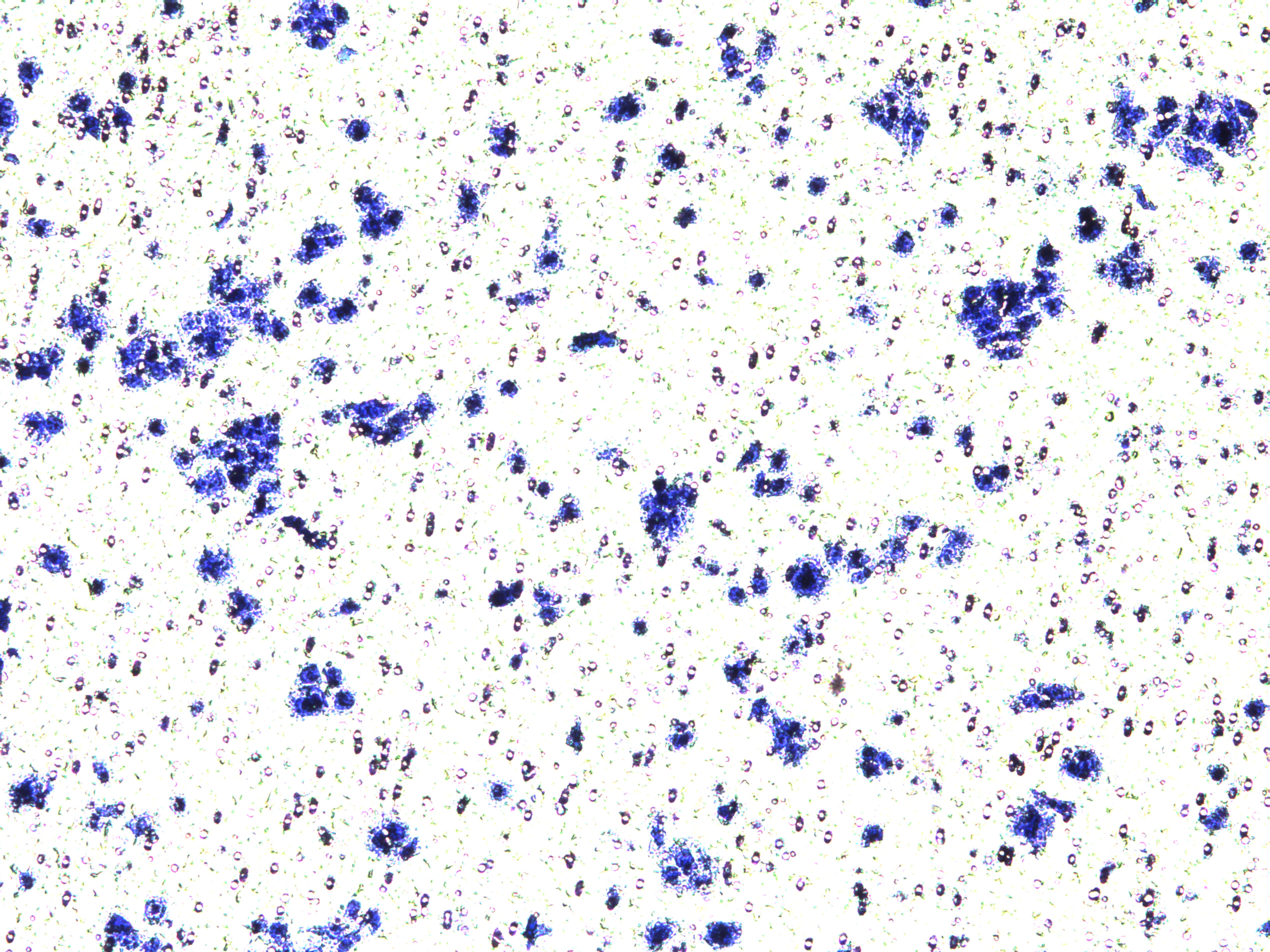

Supplement: S11 File — (ZIP) [file pone.0334639.s011.zip › S 16. File. Original FIgures. Fig.9/9d/hepg2sh-NC+DMSO .jpg]

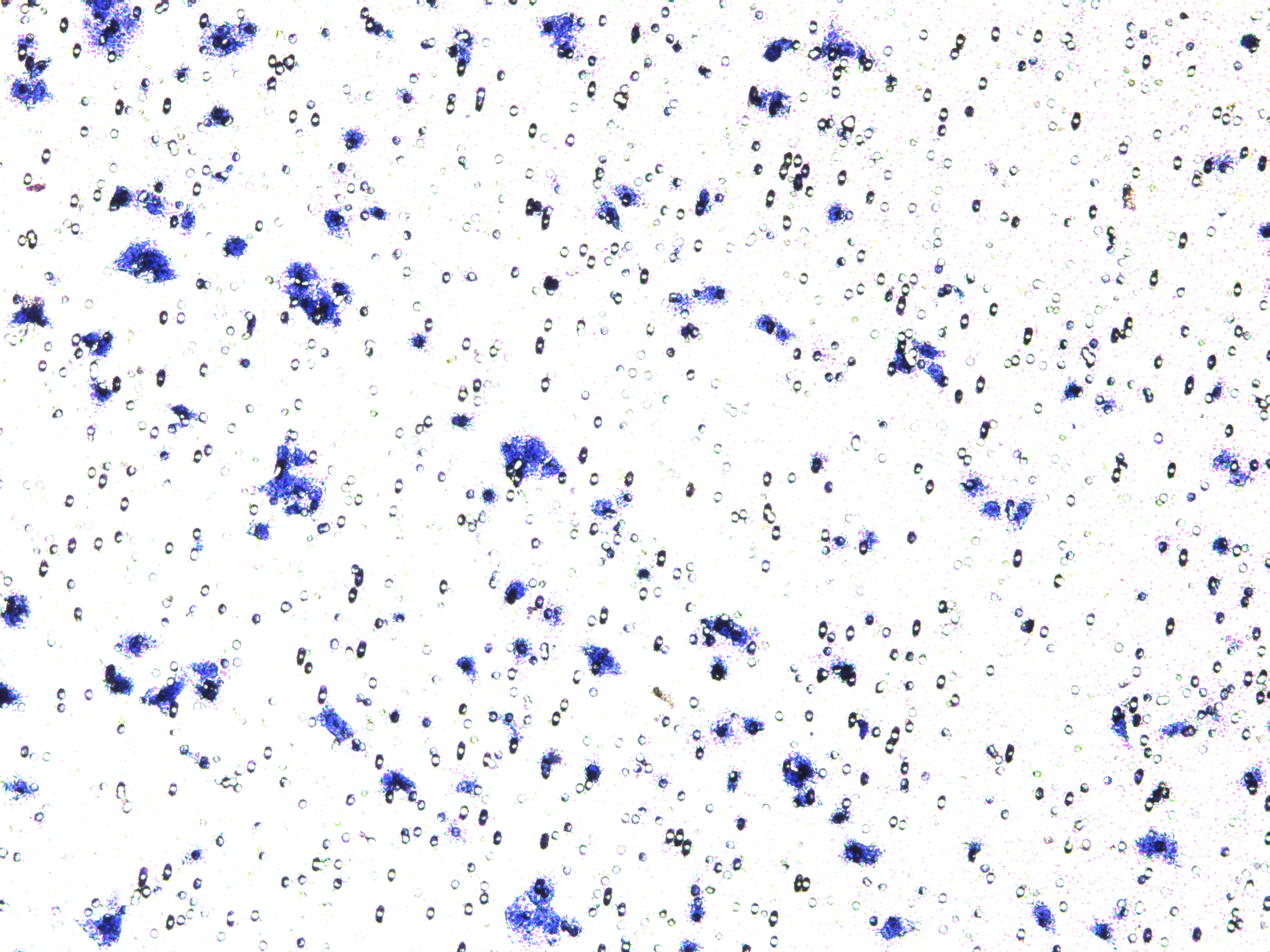

Supplement: S11 File — (ZIP) [file pone.0334639.s011.zip › S 16. File. Original FIgures. Fig.9/9d/hepg2sh-NC+MTOR.jpg]

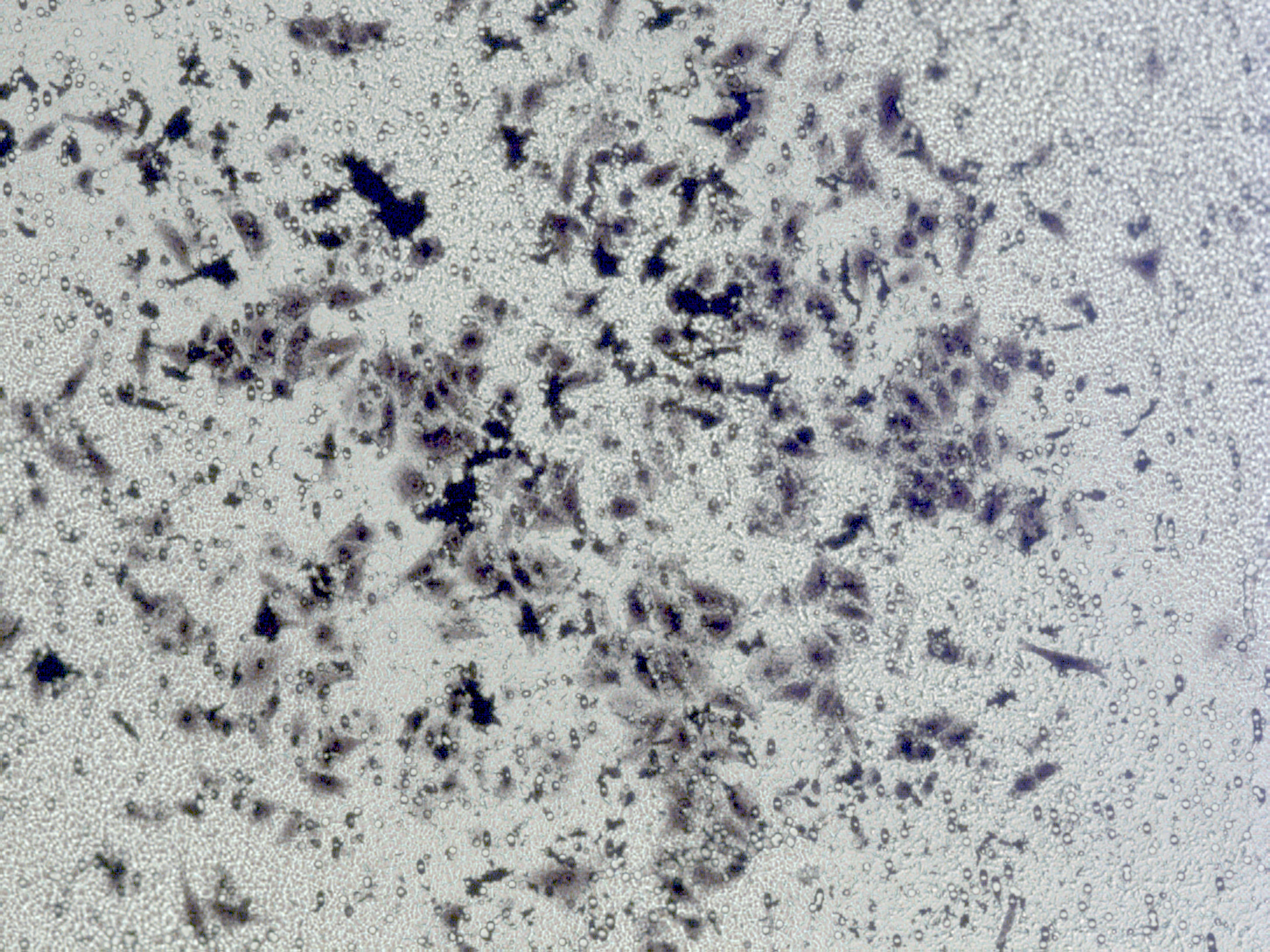

Supplement: S11 File — (ZIP) [file pone.0334639.s011.zip › S 16. File. Original FIgures. Fig.9/9d/SMMC-7721 sh-CXCL3+DMSO .jpg]

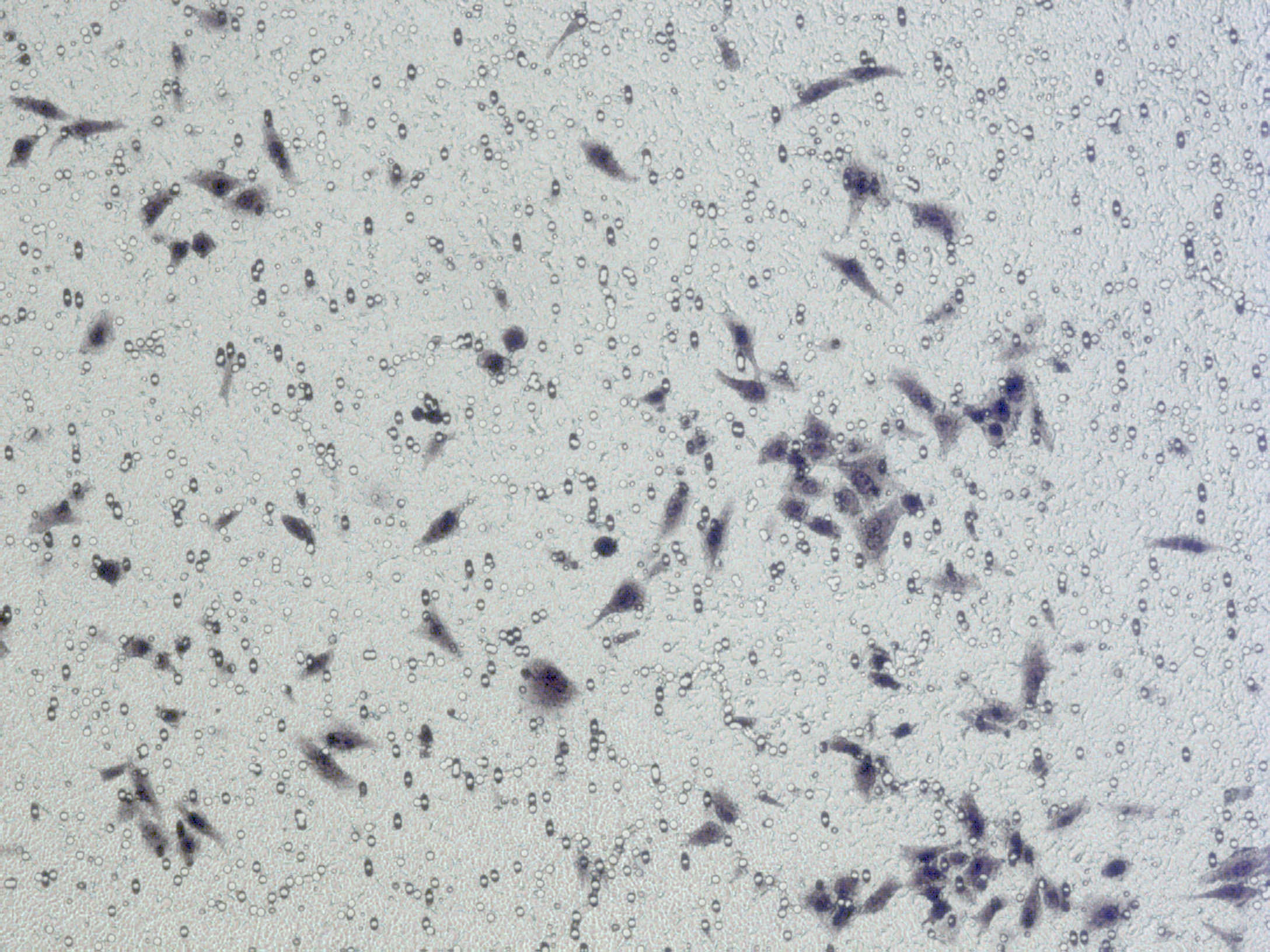

Supplement: S11 File — (ZIP) [file pone.0334639.s011.zip › S 16. File. Original FIgures. Fig.9/9d/SMMC-7721 sh-CXCL3+MTOR.jpg]

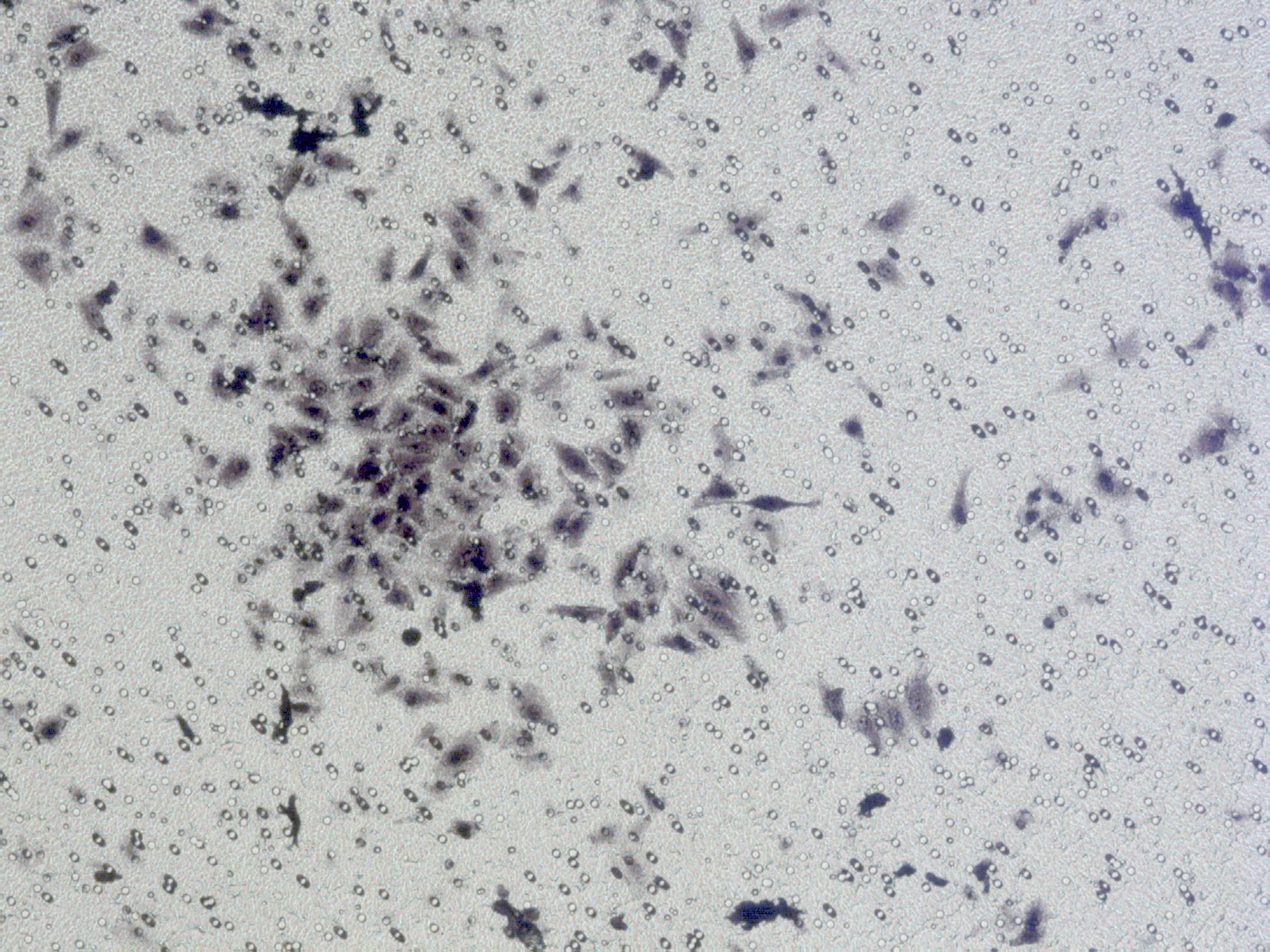

Supplement: S11 File — (ZIP) [file pone.0334639.s011.zip › S 16. File. Original FIgures. Fig.9/9d/SMMC-7721 sh-NC+DMSO .jpg]

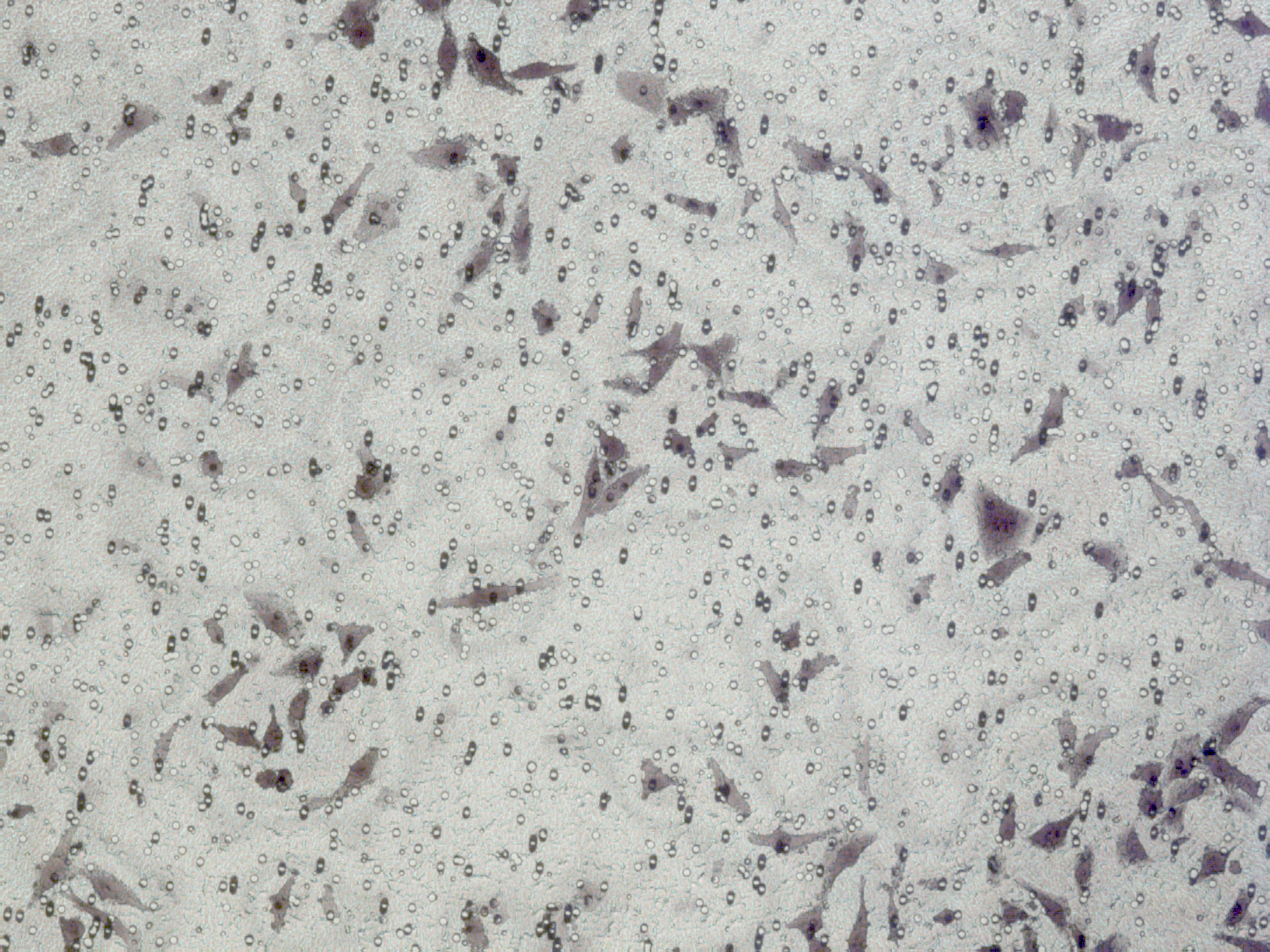

Supplement: S11 File — (ZIP) [file pone.0334639.s011.zip › S 16. File. Original FIgures. Fig.9/9d/SMMC-7721 sh-NC+MTOR.jpg]
